# Transcriptome analysis of 20 taxonomically related benzylisoquinoline alkaloid-producing plants

**DOI:** 10.1186/s12870-015-0596-0

**Published:** 2015-09-18

**Authors:** Jillian M. Hagel, Jeremy S. Morris, Eun-Jeong Lee, Isabel Desgagné-Penix, Crystal D. Bross, Limei Chang, Xue Chen, Scott C. Farrow, Ye Zhang, Jung Soh, Christoph W. Sensen, Peter J. Facchini

**Affiliations:** Department of Biological Sciences, University of Calgary, Calgary, AB T2N 1N4 Canada; Department of Biochemistry and Molecular Biology, University of Calgary, Calgary, AB T2N 4N1 Canada; Current address: Département de Chimie, Biochimie et Physique, Université du Québec à Trois-Rivières, Trois-Rivières, QC G9A 5H7 Canada; Current address: Institute of Molecular Biotechnology, Graz University of Technology, Graz, A-8010 Austria

## Abstract

**Background:**

Benzylisoquinoline alkaloids (BIAs) represent a diverse class of plant specialized metabolites sharing a common biosynthetic origin beginning with tyrosine. Many BIAs have potent pharmacological activities, and plants accumulating them boast long histories of use in traditional medicine and cultural practices. The decades-long focus on a select number of plant species as model systems has allowed near or full elucidation of major BIA pathways, including those of morphine, sanguinarine and berberine. However, this focus has created a dearth of knowledge surrounding non-model species, which also are known to accumulate a wide-range of BIAs but whose biosynthesis is thus far entirely unexplored. Further, these non-model species represent a rich source of catalyst diversity valuable to plant biochemists and emerging synthetic biology efforts.

**Results:**

In order to access the genetic diversity of non-model plants accumulating BIAs, we selected 20 species representing 4 families within the Ranunculales. RNA extracted from each species was processed for analysis by both 1) Roche GS-FLX Titanium and 2) Illumina GA/HiSeq platforms, generating a total of 40 deep-sequencing transcriptome libraries. *De novo* assembly, annotation and subsequent full-length coding sequence (CDS) predictions indicated greater success for most species using the Illumina-based platform. Assembled data for each transcriptome were deposited into an established web-based BLAST portal (www.phytometasyn.ca) to allow public access. Homology-based mining of libraries using BIA-biosynthetic enzymes as queries yielded ~850 gene candidates potentially involved in alkaloid biosynthesis. Expression analysis of these candidates was performed using inter-library FPKM normalization methods. These expression data provide a basis for the rational selection of gene candidates, and suggest possible metabolic bottlenecks within BIA metabolism. Phylogenetic analysis was performed for each of 15 different enzyme/protein groupings, highlighting many novel genes with potential involvement in the formation of one or more alkaloid types, including morphinan, aporphine, and phthalideisoquinoline alkaloids. Transcriptome resources were used to design and execute a case study of candidate *N*-methyltransferases (NMTs) from *Glaucium flavum*, which revealed predicted and novel enzyme activities.

**Conclusions:**

This study establishes an essential resource for the isolation and discovery of 1) functional homologues and 2) entirely novel catalysts within BIA metabolism. Functional analysis of *G. flavum* NMTs demonstrated the utility of this resource and underscored the importance of empirical determination of proposed enzymatic function. Publically accessible, fully annotated, BLAST-accessible transcriptomes were not previously available for most species included in this report, despite the rich repertoire of bioactive alkaloids found in these plants and their importance to traditional medicine. The results presented herein provide essential sequence information and inform experimental design for the continued elucidation of BIA metabolism.

**Electronic supplementary material:**

The online version of this article (doi:10.1186/s12870-015-0596-0) contains supplementary material, which is available to authorized users.

## Background

**Fig. 1:**
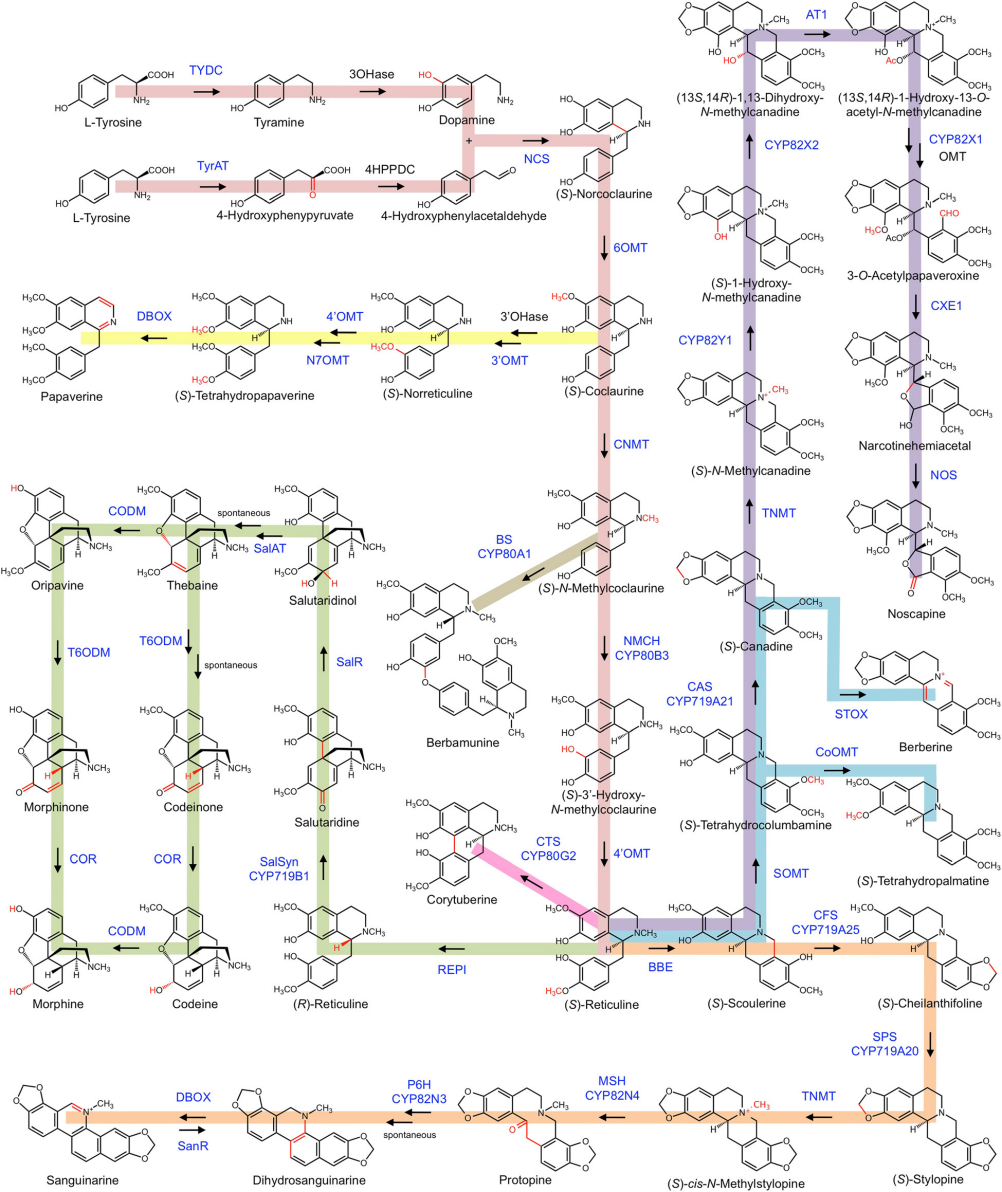
Major routes of BIA biosynthesis leading to (*S*)-reticuline (light pink), papaverine (yellow), morphine (green), sanguinarine (orange), berberine (blue) and noscapine (purple). *C-O* and *C-C* coupling reactions are shown for berbamunine (olive) and corytuberine (dark pink), respectively. Red within each alkaloid highlights enzyme-catalyzed structural changes. Solid and dotted arrows represent reactions catalyzed by single and multiple enzymes, respectively. Enzymes abbreviated in blue text have been characterized at the molecular level, whereas those in black text have not been cloned. Abbreviations: 3'-OHase, 3'-hydroxylase; 3'OMT, 3'-*O*-methyltransferase; 3OHase, 3-hydroxylase; 4HPPDC, 4-hydroxyphenylpyruvate decarboxylase; 4'OMT, 3'-hydroxy-*N*-methylcoclaurine 4'-*O*-methyltransferase; 6OMT, norcoclaurine 6-*O*-methyltransferase; AT1, 1,13-dihydroxy-*N*-methylcanadine 13-*O*-acetyltransferase; BBE, berberine bridge enzyme; BS, berbamunine synthase; CAS, canadine synthase; CFS, cheilanthifoline synthase; CNMT, coclaurine *N-*methyltransferase; CODM, codeine *O*-demethylase; CoOMT, columbamine *O*-methyltransferase; COR, codeinone reductase; CTS, corytuberine synthase; CYP82X1, 1-hydroxy-13-*O*-acetyl-*N*-methylcanadine 8-hydroxylase; CYP82X2, 1-hydroxy-*N*-methylcanadine 13-hydroxylase; CYP82Y1, *N*-methylcanadine 1-hydroxylase; CDBOX, dihydrobenzophenanthridine oxidase; CXE1, 3-*O*-acetylpapaveroxine carboxylesterase; MSH, *N-*methylstylopine hydroxylase; N7OMT, norreticuline 7-*O*-methyltransferase; NCS, norcoclaurine synthase; NMCanH, *N-*methylcanadine 1-hydroxylase; NMCH, *N*-methylcoclaurine 3'-hydroxylase; NOS, noscapine synthase; P6H, protopine 6-hydroxylase; REPI, reticuline epimerase; SalAT, salutaridinol 7-*O-*acetyltransferase; SalR, salutaridine reductase; SalSyn, salutaridine synthase; SanR, sanguinarine reductase; SOMT, scoulerine 9-*O*-methyltransferase; SPS, stylopine synthase; STOX, (*S*)-tetrahydroprotoberberine oxidase; T6ODM, thebaine 6-*O*-demethylase; TNMT, tetrahydroprotoberberine *N-*methyltransferase; TYDC, tyrosine decarboxylase; TyrAT, tyrosine aminotransferase

Benzylisoquinoline alkaloids (BIAs) are a diverse class of plant specialized metabolites that includes approximately 2500 known compounds. Although BIAs present a wide range of structural backbone arrangements, they are united in their common biosynthetic origin, which begins with the condensation of two tyrosine derivatives forming the first dedicated BIA, (*S*)-norcoclaurine (Fig. 1). Several of humanity’s most ancient medicines, poisons, hunting aids, and ceremonial preparations derive from plants accumulating BIAs, with examples found in both Old World and New World cultures [17]. Notable BIA-accumulating plants include morphine, codeine, and noscapine-accumulating opium poppy (*Papaver somniferum*), members of the berberine-accumulating barberry (*Berberis*) genus, Japanese goldthread (*Coptis japonica*), meadowrue (*Thalictrum flavum*), and species producing the antimicrobial sanguinarine, such as Mexican prickly poppy (*Argemone mexicana*) and California poppy (*Eschscholzia californica*). These plants form a core group of model species studied extensively in past decades, leading to the near-complete elucidation of major pathways at the biochemical and molecular genetic levels. Most or all enzymes responsible for the biosynthesis of papaverine, morphine, sanguinarine, berberine and noscapine have been cloned and characterized (Fig. 1) [6,17]. A restricted number of enzyme families have been implicated in BIA metabolism, which likely reflects a monophyletic origin for the pathway [34]. This feature has enabled homology-based enzyme discovery strategies, where predictions are made regarding enzyme type(s) acting at unresolved points along the BIA metabolic network. For example, *C-C* or *C-O* coupling reactions are almost exclusively catalyzed by cytochromes P450 with homology to one of CYP80, CYP82, or CYP719 families, or 2-oxoglutarate/Fe^2+^-dependent dioxygenases. Resolution of previously uncharacterized steps in sanguinarine and noscapine metabolism has been achieved through homology-based querying of transcriptome resources coupled with targeted metabolite analysis [1,6,7]. This approach was used recently for the discovery of dihydrosanguinarine benzophenanthridine oxidase (DBOX), a FAD-dependent oxidase with homology to berberine bridge enzyme (BBE) [15]. Other enzyme types found repeatedly within BIA metabolism include *O*- and *N*-methyltransferases, BAHD acylating enzymes [5] and reductases belonging to either aldo-keto (AKR) [39] or short-chain dehydrogenase/reductase (SDR) [23] superfamilies. Only the first step of BIA biosynthesis is catalyzed by a unique protein family, pathogenesis-related 10 (PR10)/Bet v1 allergens, otherwise absent within alkaloid metabolism (*i.e.* NCS; (*S*)-norcoclaurine synthase). Nonetheless, homologues of NCS appear to play this key entry-point role across different plant taxa [27].

Beyond model species, a myriad of other plants are known to accumulate BIAs. The structural diversity of these alkaloids is remarkable, yet their biosynthesis is largely or entirely unexplored. Many of these compounds have potent pharmacological activities, and plants accumulating them boast long histories of use in traditional medicine. Members of the *Cissampelos* genus, which accumulate novel bisbenzylisoquinoline, aporphine, and promorphinan-type alkaloids (Additional file 1) have been employed for centuries as hunting poisons and herbal remedies, particularly in South America and sub-Saharan Africa [45]. Trilobine, a highly crosslinked, atypical bisbenzylisoquinoline alkaloid, is thought to confer antiamoebic activity to herbal *Cocculus* preparations for the treatment of infant diarrhea [41]. Many plants of the Papaveraceae produce alkaloids featuring unique variations on the basic protoberberine and benzophenanthridine backbones, and some genus such as *Corydalis* accumulate a surprising variety of BIA types, including protopine, pthalideisoquinoline, spirobenzylisoquinoline, and morphinan alkaloids [21]. How these alkaloids are formed is poorly understood, and scarce resources are available for the non-model plants capable of producing them. To enable pathway elucidation and novel enzyme discovery, we have generated expansive datasets for twenty BIA-accumulating plants using Roche 454 and Illumina sequencing platforms. Data mining frameworks were constructed using a multitude of annotation approaches based on direct searches of public databases, and associated information was collected and summarized for every unigene, including Kyoto Encyclopedia of Genes and Genomes (KEGG) pathway maps, Gene Ontology (GO) and Enzyme Commission (EC) annotations. A comprehensive, broad-scope metabolite survey was performed in tandem with the herein presented transcriptome analysis, on identical plant tissues [18]. Used together, these unprecedented resources will allow the assembly of biochemical snapshots representing BIA metabolism in largely unexplored systems, guiding pathway elucidation and search efforts for new catalysts. Moreover, the availability of enzyme variants mined from different plant species will dramatically expand the ‘toolbox’ essential to synthetic biology efforts.

## Results and discussion

### Species and tissue selection for enrichment of biosynthetic genes

Twenty plant species were chosen for transcriptomic analysis, based primarily on alkaloid accumulation profiles, as determined by relevant literature sources and our concomitant study of metabolite content for candidate species [18]. Other considerations included taxonomic distribution, use in traditional medicine or cultural practices (signaling potential presence of pharmacologically active BIAs) and tissue availability. Priority was assigned to species for which sequence information was unavailable or lacking. We targeted four plant families within the order Ranunculales: the Papaveraceae (8 species), Ranunculaceae (4 species), Berberidaceae (4 species) and Menispermaceae (4 species) (Table 1). Although BIAs have been reported in diverse angiosperm taxa, they occur most commonly in these families [17]. Strong evidence supports the monophyletic origin of the Ranunculales, and within this order, the Papaveraceae family appears to have diverged early from the ‘core’ Ranunculales group (Additional file 2) [50]. Further evidence supports an early, monophyletic origin of BIA biosynthesis prior to the emergence of eudicots [34] suggesting that the last common ancestor of Ranunculales species was already making alkaloids. To enrich for BIA biosynthetic transcripts, analysis was restricted to alkaloid-rich organs (stem, rhizome, or root) or callus culture (Table 1). As an alternative to intact plants, cell cultures have been used for more than three decades as biosynthetic models and alkaloid production systems [54]. In vitro plant cell cultures have been instrumental in the discovery of several key enzymes and regulatory processes within sanguinarine, berberine, noscapine and morphine biosynthesis [17,44]. Recently, modest libraries (~3500 unigenes) for 18 alkaloid-producing cultures, including callus of three Menispermaceae species, were established [10]. To build on these resources, callus of *Cocculus trilobus*, *Tinospora cordifolia* and *Cissampelos mucronata* were chosen for deep sequencing.

**Table 1:** Details of plant species selected for deep sequencing analysis

#	Species	Abbrev.	Common Name	Family (Tribe)	Organ/Tissue
1	*Argenome mexicana*	AME	Mexican Prickly Poppy	Papaveraceae (Papaveroideae)	Stem
2	*Chelidonium majus*	CMA	Greater Celandine	Papaveraceae (Papaveroideae)	Stem
3	*Papaver bracteatum*	PBR	Persian Poppy	Papaveraceae (Papaveroideae)	Stem
4	*Stylophorum diphyllum*	SDI	Celandine Poppy	Papaveraceae (Papaveroideae)	Stem
5	*Sanguinaria canadensis*	SCA	Bloodroot	Papaveraceae (Papaveroideae)	Rhizome
6	*Eschscholzia californica*	ECA	California Poppy	Papaveraceae (Papaveroideae)	Root
7	*Glaucium flavum*	GFL	Yellow Horn Poppy	Papaveraceae (Papaveroideae)	Root
8	*Corydalis chelanthifolia*	CCH	Ferny Fumewort	Papaveraceae (Fumarioideae)	Root
9	*Hydrastis canadensis*	HCA	Goldenseal	Ranunculaceae	Rhizome
10	*Nigella sativa*	NSA	Black Cumin	Ranunculaceae	Root
11	*Thalictrum flavum*	TFL	Meadow Rue	Ranunculaceae	Root
12	*Xanthorhiza simplicissima*	XSI	Yellowroot	Ranunculaceae	Root
13	*Mahonia aquifolium*	MAQ	Oregon Grape	Berberidaceae	Bark
14	*Berberis thunbergii*	BTH	Japanese Barberry	Berberidaceae	Root
15	*Jeffersonia diphylla*	JDI	Rheumatism Root	Berberidaceae	Root
16	*Nandina domestica*	NDO	Sacred Bamboo	Berberidaceae	Root
17	*Menispermum canadense*	MCA	Canadian Moonseed	Menispermaceae	Rhizome
18	*Cocculus trilobus*	CTR	Korean Moonseed	Menispermaceae	Callus
19	*Tinospora cordifolia*	TCO	Heartleaf Moonseed	Menispermaceae	Callus
20	*Cissampelos mucronata*	CMU	Abuta	Menispermaceae	Callus

### Roche versus Illumina platforms: benefits of enhanced read depth

RNA was screened for sufficient quality and quantity prior to deep sequencing by either Roche GS-FLX Titanium or Illumina GA/HiSeq platforms. For Illumina-based sequencing, GA (Genome Analyzer) and HiSeq instruments were employed to generate data of essentially equal quality, permitting subsequent pooling of the data. Table 2 summarizes the results for both technologies, while Additional files 3 and 4 tabulate further details regarding Roche and Illumina-based platforms, respectively. Data for 6 of the 20 species (Table 1) were published previously, although minor errors were noted (e.g. Table 1b of [53]). Presented herein are corrected values, included for comparative purposes along with data for 14 new plant species. Multiplatform studies have highlighted certain advantages of Illumina-based sequencing over other technologies, which include lower costs ($0.06/Mb), high accuracy (<2 % error rate) and good read depth, permitting robust transcript quantification [32,40,46]. Good read depth herein is reflected as high average reads per base pair (69.6; Additional file 4) permitting nearly double the number of average unigenes per library compared with Roche technology (34,368 versus 63,886, respectively; Table 2). Conversely, advantages of Roche 454 GS FLX-based sequencing include longer average read lengths (e.g. >12-fold longer than Illumina HiSeq platforms; [32]) enabling reliable detection of splice variants. Despite longer reads, Roche-based sequencing resulted in less predicted full-length coding sequences (CDSs) compared with Illumina-based sequencing (Additional files 3 and 4). Nonetheless, using two different platforms had the inherent advantage of enhanced overall transcriptome coverage. Roche and Illumina libraries averaged ~14,000 and ~24,500 full-length CDSs respectively, with an average of ~7700 CDS intersects between the libraries as determined by conservative, Mega BLAST estimates with an *e-*value cutoff of 0 ([56]; Additional file 3). The low number of CDS intersects likely reflects the use of stringent BLAST parameters rather than inherent differences between the two libraries, and increasing the *e-*value cutoff would be expected to reveal greater concordance.

**Table 2:** Annotation summaries for Roche-based and Illumina-based transcriptomes

			Roche GS-FLX Titanium					Illumina GA/HiSeq				
No.	Abbrev.	Plant	Unigenes	Overall annotated	High-level annotated	GO annotated	EC number allocated	Unigenes	Overall annotated	High-level annotated	GO annotated	EC number allocated
1	AME	*Argemone mexicana*	25,499	22,121	17,979	21,974	3086	75,101	60,836	45,404	60,254	7653
2	BTH	*Berberis thunbergii*	41,672	33,548	23,243	33,080	4197	88,302	61,576	41,927	60,561	7289
3	CMA	*Chelidonium majus*	23,678	19,635	13,977	19,460	2368	45,005	42,057	33,449	41,956	6092
4	CMU	*Cissampelos mucronata*	35,166	27,451	19,865	27,139	3147	69,822	32,209	22,943	31,597	3314
5	CTR	*Cocculus trilobus*	34,783	26,678	18,701	26,338	3197	84,793	33,055	21,961	30,542	432
6	CCH	*Corydalis chelanthifolia*	22,511	19,161	14,633	19,024	2433	51,797	48,423	42,784	48,139	7738
7	ECA	*Eschscholzia californica*	32,150	28,430	21,403	28,194	4221	42,167	38,332	32,677	38,063	6545
8	GFL	*Glaucium flavum*	26,520	20,945	15,645	20,725	2719	31,100	31,100	19,669	31,100	3231
9	HCA	*Hydrastis canadensis*	23,809	20,443	15,491	20,230	2511	33,335	33,335	20,898	33,335	3637
10	JDI	*Jeffersonia diphylla*	38,773	24,583	16,777	24,199	2581	86,832	31,712	22,574	30,842	3118
11	MAQ	*Mahonia aquifolium*	36,429	30,209	20,624	29,805	3581	98,375	53,093	33,434	47,040	521
12	MCA	*Menispermum canadense*	36,399	31,715	24,565	31,482	4495	87,141	70,524	52,713	69,877	8924
13	NDA	*Nandina domestica*	45,387	33,501	24,308	33,010	4186	70,425	53,109	38,428	52,531	6553
14	NSA	*Nigella sativa*	50,508	36,231	25,560	35,591	4526	67,591	41,260	29,127	40,316	4807
15	PBR	*Papaver bracteatum*	46,224	33,168	24,381	32,767	4988	70,428	56,463	37,334	53,039	6793
16	SCA	*Sanguinaria canadensis*	25,652	20,493	15,938	20,301	2621	53,019	47,247	40,122	46,890	7715
17	SDI	*Stylophorum diphyllum*	43,568	34,954	26,144	34,614	5115	50,125	40,797	30,157	40,324	5276
18	TFL	*Thalictrum flavum*	21,146	17,609	12,121	17,431	2294	41,982	33,120	23,900	32,711	4123
19	TCO	*Tinospora cordifolia*	34,518	28,044	21,199	27,795	3444	81,927	35,851	24,174	34,712	3386
20	XSI	*Xanthoriza simplicissima*	42,969	33,657	22,165	33,187	3740	48,447	39,281	27,434	38,831	4642
		Average	34,368	27,128	19,736	26,817	3472	63,886	44,169	32,055	43,133	5089

### Library comparisons reveal isolated cases of low intersection

Variation in library quality between different source tissues (e.g. stem vs root, callus) was not apparent. For quality control measures, Illumina-based sequencing was performed on both stem and root of *Chelidonium majus* yielding comparable results (Additional file 5). However, library quality appeared reduced in isolated cases. For example, the Illumina-based *Cocculus trilobus* library consisted of a large number of reads, but yielded an above average number of unassembled contigs and a small number of full-length CDSs (Additional file 4). Conversely, Roche-based *C. trilobus* sequencing appeared relatively successful (Additional file 3). As Illumina- and Roche-based libraries were constructed using the same source material, we ruled out the possibility that *C. trilobus* tissue was compromised, as poor tissue quality would have affected both transcriptomes, not just the Illumina data. Another Illumina library with reduced full-length CDSs (compared to raw reads) and low intersection with Roche data included *Mahonia aquifolium*. It is possible that cross-contamination with samples derived from other plants occurred in these cases, precluding proper assembly and separation of foreign or native sequences at later stages.

### Establishment of fully annotated BLAST- accessible transcriptomes

On average, 79 % (Roche) and 69 % (Illumina) of all unigenes received a functional annotation, with high-level annotations based on more stringent criteria assigned to 57 % (Roche) and 50 % (Illumina) (Table 2). Enzyme Commission (EC) number allocation was included in the analysis to gain insight on the number of enzymes represented in each library, and enable corresponding links to KEGG pathway maps (www.genome.jp/kegg/pathway). More importantly for enzyme discovery, EC assignments can facilitate word searches based on enzyme function. On average for both Roche and Illumina libraries, about 12 % of all annotations corresponded to an EC number. Low success in EC number assignments was noted for *C. trilobus* and *M. aquifolium* Illumina libraries, likely due to poor assembly of full-length CDSs. Results for every unigene, including constituent reads, expression data, BLAST results, annotation evidence and relevant links are summarized on individual pages available through MAGPIE. A previously established MAGPIE-based BLAST portal [53] is available for public access to the assembled data of each transcriptome reported herein (www.phytometasyn.ca).

### Homology-based mining of BIA biosynthetic genes

Illumina and Roche 454-based transcriptomes were mined for candidate genes putatively involved in BIA metabolism. tBLASTn searches were performed on the basis of homology to fully characterized alkaloid biosynthetic enzymes, using a cutoff of 40 % sequence identity in most cases. Exceptions include O-acetyltransferases (OATs) and carboxylesterases (CXEs) where a search cutoff of 30 % was generally used. For OATs and CXEs, greater sequence divergence between taxonomic groups was evident, prompting more flexible search criteria. A pre-defined cutoff was not required in some cases, since tBLASTn yielded a small number of hits with relatively high identity. For example, searches using berberine bridge enzyme from *Eschscholtzia californica*, *Papaver somniferum* and *Berberis stolonifera* (EsBBE, PsBBE and BsBBE respectively) yielded a total of 18 hits with substantial (>60 %) identity. Similar results were obtained for dihydrobenzophenanthridine oxidase (DBOX)-like FAD-dependent oxidases (FADOX). In total, ~850 candidate unigenes were selected from 40 deep sequencing libraries, representing 20 BIA-accumulating plant species. Additional file 6 lists the amino acid sequences of these candidates in FASTA format.

### Gene expression for candidate selection and bottleneck identification

**Fig. 2:**
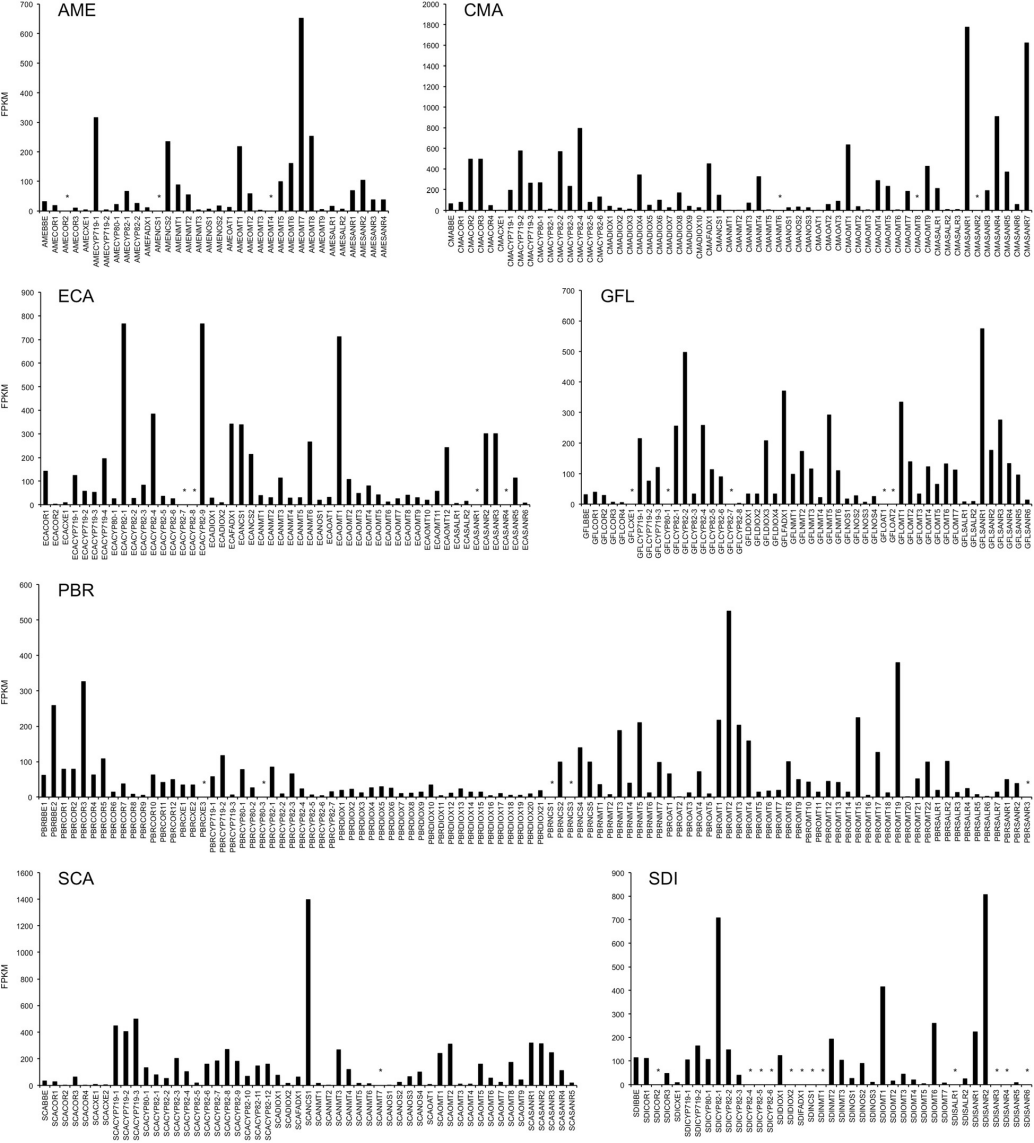
Normalized expression analysis for gene candidates potentially involved in BIA biosynthesis in Papaveraceae (tribe: Papaveroideae) species. Each candidate is labeled with respective species abbreviations (e.g. AME, *Argemone mexicana*) and the type of enzyme potentially encoded by the gene (e.g. BBE, berberine bridge enzyme). Candidates present exclusively in Roche-based transcriptomes could not be assigned an FPKM value, and are marked with asterisk. Refer to Table 1 for species abbreviations. Enzyme/protein family abbreviations: BBE, berberine bridge enzyme; COR, codeinone reductase; CXE, carboxylesterase; CYP, cytochrome P450 monooxygenase; DIOX, dioxygenase; FAD, FAD-dependent oxidase; NCS, norcoclaurine synthase; NMT, N-methyltransferase; NOS, noscapine synthase; OAT, *O*-acetyltransferase; OMT, *O*-methyltransferase; SALR, salutaridine reductase; SANR, sanguinarine reductase

Expression data were recorded for each candidate in the form of FPKM (Fragments Per Kilobase of exon model per Million mapped reads) extracted from Illumina libraries. Figure 2 summarizes results obtained for Papaveroideae tribe members (Papaveraceae). Expression results for *Corydalis chelanthifolia* (Fumarioideae tribe, Papaveraceae), Berberidaceae and Ranunculaceae species are found in Additional file 7, and results for Menispermaceae species are found in Additional file 8. Expression analyses were not performed for *M. aquifolium* and *C. trilobus* due to reduced numbers of full-length CDSs. Expression values were normalized across all Illumina libraries, permitting cross-species comparison (see methods). FPKM and related RNA-seq tools are reliable expression metrics; in fact, recent head-to-head comparison of Illumina and microarray-based data showed that RNA-seq dramatically outperforms microarray in identifying differentially expressed genes [49]. For the purpose of novel catalyst discovery, gene expression data can be used to prioritize candidates for further analysis. Genes highly expressed in BIA-synthesizing tissues can be selected over candidates with very low expression levels. For example, while 17 putative (S)-norcoclaurine synthase (NCS) candidates were identified within Papaveraceae libraries, some of these unigenes were observed only as low-read Roche contigs and were entirely absent from Illumina data (Fig. 2, Additional file 7). Lack of Illumina data could reflect a platform bias or processing error, although it is possibly the result of very low gene expression. Expression comparisons can be made across different gene families to gain insight regarding putative metabolic bottlenecks. *Papaver bracteatum* accumulates large quantities of thebaine but only trace amounts of downstream alkaloids codeine and oripavine [24], implicating a metabolic block at thebaine 6-*O*-demethylase (T6ODM) and codeine *O*-demethylase (CODM) (Fig. 1). T6ODM and CODM have been characterized in opium poppy and belong to the Fe^2+^/2-oxoglutarate-dependent dioxygenase (DIOX) family [16]. Compared with other BIA-biosynthetic genes in *P. bracteatum*, DIOX homologues are expressed at very low levels, possibly contributing to observed pathway restrictions.

### Phylogenetic analysis as prediction tool for gene function: NMT case study

**Fig. 3:**
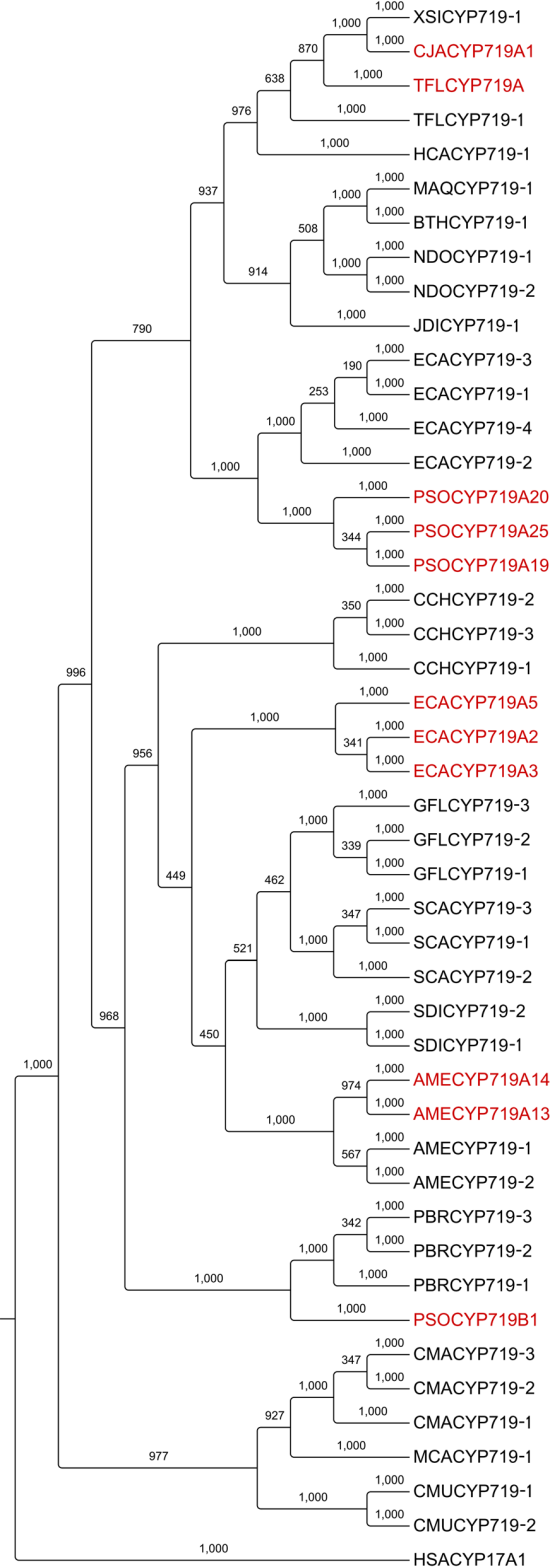
Phylogenetic analysis of CYP719 gene candidates from twenty BIA-accumulating plant species. Red text denotes characterized genes or enzymes used as tBLASTn queries for transcriptome mining. Black text denotes uncharacterized gene candidates identified through mining (>40 % identity to queries). Bootstrap values for each clade were based on 1000 iterations. Each candidate is labeled with respective species abbreviation (e.g. AME, *Argemone mexicana*; see Table 1) and candidate number (e.g. CYP719-1). Each query is labeled according to species (additional species: CJA, *Coptis japonica*; PSO, *Papaver somniferum*) with CYP719 subfamily and gene number indicated (e.g. CYP719B1, salutaridine synthase; see Fig. 1). Outgroup is CYP17A1 from *Homo sapiens* (HSA). Amino acid sequences for candidates, queries, and outgroups are found in Additional file 6

**Fig. 4:**
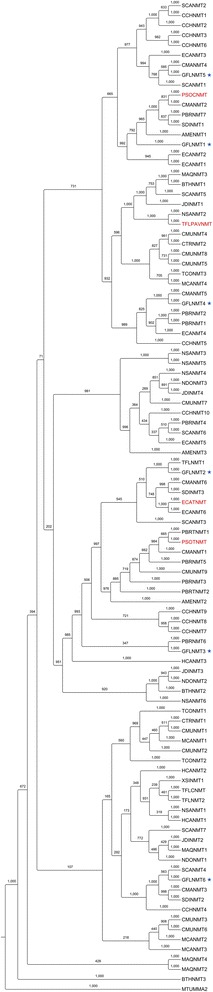
Phylogenetic analysis of *N*-methyltransferase (NMT) gene candidates from twenty BIA-accumulating plant species. Red text denotes characterized genes or enzymes used as tBLASTn queries for transcriptome mining. Black text denotes uncharacterized gene candidates identified through mining (>40 % identity to queries). Bootstrap values for each clade were based on 1000 iterations. Each candidate is labeled with respective species abbreviation (e.g. AME, *Argemone mexicana*; see Table 1) and candidate number (e.g. NMT1). Each query is labeled according to species (additional species: PSO, *Papaver somniferum*) and specific NMT function (CNMT, coclaurine *N*-methyltransferase; PAVNMT, pavine *N-*methyltransferase; TNMT, tetrahydroprotoberberine *N*-methyltransferase; see Fig. 1). Outgroup is mycolic acid synthase from *Mycobacterium tuberculosis* (MTUMMA2). NMT candidates from *Glaucium flavum* tested for catalytic activity are indicated with asterisks. Amino acid sequences for candidates, queries, and outgroups are found in Additional file 6

Amino-acid alignments and phylogenetic trees were assembled for 15 classes of protein/enzymes, representing a total of ~850 gene candidates. Figures 3 and 4 illustrate the trees built using CYP719 and *N*-methyltransferase candidates, respectively. Remaining trees are found in the Additional files 9, 10, 11, 12, 13, 14, 15, 16, 17, 18, 19, 20 and 21. Used together with the corresponding FPKM data and species-specific alkaloid profiles [18] these results represent an important resource for the discovery of new enzymes catalyzing (i) previously characterized reactions (i.e. functional homologues) and (ii) reactions uncharacterized at the biochemical and molecular levels. To test our hypothesis that phylogenetic considerations could be used to predict enzyme function, we designed an empirical case study using *Glaucium flavum N*-methyltransferase (NMT) gene candidates. Homology-based mining revealed six full-length NMT candidates in both Roche- and Illumina-based *G. flavum* transcriptomes (Fig. 2). Phylogenetic analysis revealed closer relationships between certain *G. flavum* candidates to characterized enzymes compared to others. For example, GFLNMT1 formed a six-member clade with PSOCNMT, an established coclaurine *N-*methyltransferase (CNMT) from *Papaver somniferum* [19] (Fig. 4). In contrast, GFLNMT2 formed a 6-member clade including (*S*)-tetrahydroprotoberberine *N*-methyltransferase (TNMT) from *Eschscholzia californica* (ECATNMT) [35]. On the basis of these results, it was predicted that GFLNMT1 and GFLNMT2 enzymes would exhibit CNMT and TNMT activities, respectively. Although the remaining GFLNMTs did not form similarly small clades with, or exhibit such high identity (>70 %) to known enzymes, activity with BIA substrates was anticipated owing to the >40 % identity with query sequences. All six *G. flavum* candidates were produced in *Escherichia coli* as His-tagged recombinant proteins, each of which showed a predicted molecular weight as determined by comparison with molecular weight standards (Additional file 22). Each protein was tested for NMT activity using six key alkaloid substrates (Table 3). Indeed, GFLNMT1 and GFLNMT2 exhibited CNMT and TNMT activities using coclaurine and protoberberine substrates, respectively. Further, our prediction that all *G. flavum* enzymes would accept BIA substrates proved correct. GFLNMT3 acted as TNMT using (*S*)-stylopine substrate, but unexpectedly also *N*-methylated (*S*)-reticuline. (*S*)-Reticuline *N*-methyltransferase activity was also observed for GFLNMT5. GFLNMT4 acted as CNMT with the notable distinction of carrying out subsequent *N*,*N*-dimethylation reactions to form a quaternary amine. Although GFLNMT6 did not cluster closely with characterized CNMT (Fig. 4), it accepted coclaurine substrate. These results demonstrate the general utility of phylogenetic analysis as a predictive tool, but underscore the need for empirical assay data for the purposes of gene discovery.

**Table 3:** Relative conversion of five alkaloids tested as potential substrates for six NMT candidates from *Glaucium flavum*

Substrate	Relative activity (% maximum)					
	GFLNMT1	GFLNMT2	GFLNMT3	GFLNMT4	GFLNMT5	GFLNMT6
(*S*)-Coclaurine	100 ± 1^a^	nd	nd	100 ± 10^d, e^	<1	100 ± 14^g^
(*S*)-Reticuline	nd	nd	100 ± 5^c^	8 ± 1	100 ± 5^f^	nd
(*S*)-Canadine	nd	66 ± 1	<1	nd	nd	nd
(*S*)-Stylopine	nd	100 ± 2^b^	81 ± 18	nd	nd	nd
(+/−)-Pavine	nd	<1	nd	nd	nd	nd

Values represent the mean ± standard deviation of three independent assays. For each enzyme, activity was calculated relative to the assay showing the highest conversion of substrate (i.e. the average of this assay was set to 100 %). The accompanying footnote defines 100 % conversion in pmole min^−1^ mg^−1^ protein for each enzyme

^a^5.9 pmole min^−1^ mg^−1^ protein

^b^61 pmole min^−1^ mg^−1^ protein

^c^0.4 pmole min^−1^ mg^−1^ protein

^d^Products were *N*-methylcoclaurine and *N,N*-dimethylcoclaurine

^e^2.3 pmole min^−1^ mg^−1^ protein

^f^ 0.1 pmole min^−1^ mg^−1^ protein

^g^3.3 pmole min^−1^ mg^−1^

### Functional homologue resource for synthetic biology

For the purposes of emerging synthetic biology initiatives, functional homologues - often termed enzyme 'variants' - are essential engineering tools. Assembly of alkaloid pathways in microbes using heterologously expressed plant enzymes is fraught with problems - including poor protein expression, unpredictable/off-target activities, poor interaction with other pathway enzymes, and low catalytic efficiencies [28] - which can be alleviated in some cases with variant substitution. For example, testing numerous combinations of methyltransferases from *Papaver somniferum* and *Thalictrum flavum* revealed that specific variants, and combinations of variants, ameliorated (*S*)-reticuline production in yeast [19]. Our collection of *N-* and *O*-methyltransferase candidates sourced from a wide variety of plants (Fig. 4, Additional file 18) will enable further refinement of alkaloid biosynthesis in unicellular systems.

### Candidates with putative roles in morphinan and aporphine alkaloid formation

Identification of functional homologues with roles in morphinan alkaloid biosynthesis is an important objective, as reconstitution of this pathway in microbes is an emerging goal [48]. The Illumina transcriptome of morphinan alkaloid-producing *P. bracteatum* contains three CYP719 candidates, which form a well-supported clade with opium poppy (*Papaver somniferum*) salutaridine synthase (SalSyn, PSOC719B1; Fig. 3). In addition, six *P. bracteatum* unigenes with substantial homology (up to 92 % amino acid identity) to opium poppy salutaridine reductase (SalR) were identified (Fig. 2, Additional file 14). Our study includes plant genera known to produce lesser-known morphinan alkaloids, such as *Corydalis*, *Nandina* and *Thalictrum,* which produce (+)-pallidine, sinoacutine, and (−)-pallidine respectively [21,22,47]. Significantly, these plants also produce a variety of aporphine alkaloids such as nantenine (*Nandina*; [22]), isocorydine (*Corydalis*; [14]) and corydine (*Thalictrum*; [47]). The biosynthetic pathways for these morphinan and aporphine alkaloids are not known, but likely rely on CYP-mediated *C*-*C* coupling of (*S*)- or (*R*)-reticuline. The relatively few (<10) CYP80, CYP719 and CYP82 candidates were identified in these species (Fig. 3, Additional files 16 and 17) could be tested for reticuline oxidase activity and evaluated for participation in morphinan and/or aporphine pathways.

### Potential new catalysts for phthalideisoquinoline alkaloid biosynthesis

Guided by the recent elucidation of noscapine biosynthesis in opium poppy [6,51], transcriptomes of phthalideisoquinoline-accumulating species were mined for novel catalysts. *Hydrastis canadensis* produces hydrastine, hydrastidine, and other minor constituents [26] whereas *Corydalis* species accumulate a wide variety of phthalideisoquinoline alkaloids [2]. Numerous acetyltransferase, carboxylesterase, and CYP82 candidates with possible involvement in phthalideisoquinoline biosynthesis were identified in *H. canadensis* and *C. chelanthifolia* transcriptomes. *Corydalis* species accumulate the hemiacetal egenine [52], which may require a noscapine synthase (NOS)-like enzyme for hypothesized conversion to bicuculline [12]. Six candidates were identified in *C. chelanthifolia* with up to 52 % identity to *P. somniferum* NOS, although expression was very low in some cases (Additional file 7). Three NOS-like gene candidates with possible roles as hydrastine synthases were identified in *H. canadensis* (Additional files 7 and 21).

## Conclusions

The establishment of fully annotated, deep-sequencing transcriptomes for twenty BIA-accumulating plants represents an immense resource for novel catalyst discovery. BLAST-accessible transcriptomes were not previously available for most plants included in this report, despite the rich repertoire of bioactive alkaloids found in these species and their importance in traditional medicine. The results presented herein, together with accompanying metabolite profiles [18] and relevant literature, are intended to provide necessary tools (*i.e.* gene sequences) and also inform experimental design for the continued elucidation of the BIA metabolism.

## Methods

### Alkaloids

Alkaloids used as substrates or standards were sourced as follows: (*S*)-reticuline oxalate was a gift from Tasmanian Alkaloids (Tasmania, Australia); (*R,S*)-canadine was purchased from Latoxan (Valence, France); (±)-pavine was purchased from Sigma-Aldrich (St. Louis, MO), (*S*)-coclaurine was purchased from Toronto Research Chemicals (Toronto, ON); (*R,S*)-stylopine was synthesized as described previously [33].

### Plant material

Selected tissues were harvested from *Hydrastis canadensis*, *Sanguinaria canadensis*, *Nigella sativa*, *Mahonia aquifolium*, *Menispermum canadense*, *Stylophorum diphyllum*, and *Xanthoriza simplicissima* plants cultivated outdoors at the Jardin Botanique de Montréal (Montréal, Québec; http://espacepourlavie.ca). *Jeffersonia diphylla* and *Berberis thunbergii* plants were purchased from Plant Delights Nursery (Raleigh, North Carolina; www.plantdelights.com) and Sunnyside Greenhouses (Calgary, Alberta; www.sunnysidehomeandgarden.com), respectively. *Chelidonium majus*, *Papaver bracteatum*, *Argemone mexicana*, *Eschscholtzia californica*, *Nandina domestica*, *Glaucium flavum*, *Thalictrum flavum* and *Corydalis chelanthifolia* were grown from seed germinated in potted soil under standard open air greenhouse conditions at the University of Calgary (Calgary, Alberta). Seeds were obtained from B and T World Seeds (b-and-t-world-seeds.com) with the exception of *T. flavum* and *P. bracteatum*, which were obtained from Jelitto Staudensamen (www.jelitto.com) and La Vie en Rose Gardens (www.lavieenrosegardens.com), respectively. Callus cultures of *Cissampelos mucronata*, *Cocculus trilobus*, and *Tinospora cordifolia* were purchased from Deutsche Sammlung von Mikroorganismen und Zellkulturen (DSMZ, Braunschweig, Germany; www.dsmz.de) and maintained as described [10]. All tissues were flash-frozen in liquid nitrogen and stored at −80 °C until analysis.

### Poly(A) + RNA purification, cDNA library preparation and next-generation sequencing

Total RNA was extracted from stem, rhizome, root, or callus tissue using a modified CTAB method [38]. RNA quality was based on UV absorption ratios, where only samples with ratios above 2.0 (260/280 nm) and 2.2 (260/230 nm) were used. Poly(A) + RNA purification, cDNA library synthesis, emulsion-based PCR (emPCR) and NGS was performed at the McGill University and Génome Québec Innovation Center (Montréal, Québec) as described [53]. Briefly, RNA quality and quantity was assessed using NanoDrop ND-1000 (Thermo Scientific, Waltham, Massachusetts) and BioAnalyzer 2100 (Agilent Technologies, Santa Clara, California) instruments, and Poly(A) + RNA purification was done using either a Dynabeads mRNA Purification kit (Invitrogen) or TrueSeq Stranded mRNA Sample Prep kit (Illumina, San Diego, California). cDNA synthesis was performed using either a cDNA Rapid Library kit (Roche, Basel, Switzerland) or TruSeq Stranded mRNA Sample Prep kit (Illumina) depending on the downstream NGS method. For Roche-454 GS-FLX Titanium pyrosequencing, data processing was done using GS Run Processor (Roche) to generate Standard Flowgram Format (SFF) files. For Illumina GA and HiSeq sequencing, HCS 1.4 and CASAVA 1.6-1.8 software suites (Illumina) were used to generate raw fastq reads.

### De novo transcriptome assembly, functional annotation, and GO analysis

Sequence quality control and screening was performed as described [53]. Adapter/primer sequences were clipped, all sequences were trimmed based on Phred quality scores, low-complexity regions were masked, and ribosomal RNA (rRNA) sequences were removed from each 454 database using the Scylla program of the Paracel Filtering Package (PFP) (Paracel Inc., California). Quality assessment and cleaning for Illumina reads was performed using Fast QC (www.bioinformatics.babraham.ac.uk/projects/fastqc/) and Cutadapt [37]. Cleaned 454 sequence data were assembled using MIRA (v. 3.2) [4], which produced more long (>1000 bp) contigs compared with Paracel Transcript Assembler (Paracel Inc.) or Newbler v. 2.3 [36] platforms. Filtered Illumina reads were assembled using Velvet-Oases v. 0.1.16 [55]. CD-HIT-EST [30] was used to reduce redundancy by clustering of nearly identical (>99 %) transcripts, and further assembly was achieved using CAP3 [20]. MAGPIE (Magpie Automated Genomics Project Investigation Environment) [11] was used to annotate each dataset based on sequence similarity searches against public and internal databases, including NCBI and the viridiplantae subset of RefSeq. Accelerated Hidden Markov Model (HMM) searches were also performed. Full-length coding sequence predictions were performed as described [53]. Functional descriptions based on comparison with already annotated sequences from databanks, along with domain-level contents, were assigned to each contig based on a weighted summary of all the search results. GO (Gene Ontology) annotations and EC numbers were designated for each contig as described previously [53]. Toward the goal of integrating transcriptomic with corresponding metabolomic data [18] transcript data was mapped to KEGG metabolic pathways using EC numbers.

### Gene expression analysis

As a first round, relative gene expression information for every contig in all 40 libraries (twenty 454-based, and twenty Illumina-based) was acquired based on raw read abundance. For 454 libraries, raw read counts were extracted from contig assembly files. For Illumina libraries, counts were estimated using Bowtie [25] to re-map raw reads to assembled contigs, and RSEM [29] was used for final quantifications. Relative normalization (*i.e.* within each respective library) was achieved by calculating FPKM (Fragments Per Kilobase of exon model per Million mapped reads) for each contig. To enable gene expression comparisons across different libraries, a second round of normalization was performed using Illumina data. First, contigs from all twenty Illumina libraries were compiled together and grouped into clusters based on sequence similarity. Clustering of data was performed using OrthoMCL, a program designed for the scalable construction of orthologous groups across multiple eukaryotic taxa [31]. Differences in RNA quantity between libraries (*i.e.* RNA composition bias) were accounted for by calculating a combined scaling factor for each library. This step was performed using the calcNormFactors function of edgeR v.3 (www.bioconductor.org), which determines a set of factors, later combined into a single “scaling factor”, unique to each library that minimizes log-fold changes between samples for most genes [3]. A second set of FPKM values enabling cross-species comparison were generated for contigs of interest through multiplication of the first, library-specific FPKM values by respective scaling factors.

### Alignments and phylogenetic analysis of gene candidates

Amino acid alignments of candidates belonging to individual enzyme classes were performed with the in-built Muscle alignment feature of Geneious (Biomatters, Aukland, New Zealand). The alignments were performed as a free-end gap, and computational alignments were followed by hand sorting. Maximum-likelihood phylogenetic analyses were performed using the PHYML feature of Geneious [13]. Bootstrap values for each clade were based on 1000 iterations. For P450 and NMT trees, *Homo sapiens* and bacterial (*Mycobacterium tuberculosis*) sequences were used as outgroups, respectively. Sequences from distantly related taxa are not generally applied as outgroups, as phylogenetic distance can lead to degraded alignments [43]. However, alignment degradation was not observed, which is consistent with similar reports using outgroups from distant taxa for CYP analyses [42].

### Functional analysis of N-methyltransferase gene candidates

Six gene candidates with greater than 40 % sequence identity to one or more of four query sequences encoding known *N*-methyltransferases (NMTs) with established roles in BIA biosynthesis (Fig. 4; Additional file 6) were identified in the *Glaucium flavum* Illumina-based transcriptome. Coding sequences were amplified using gene-specific primers containing attB sites using Q5 HiFi DNA polymerase (New England Biolabs) and *G. flavum* root cDNA. Recombination reactions were carried out using BP and LR Clonase II (Thermo Scientific) to generate pDONR221-GfMMT entry plasmids and pHGWA-GfNMT expression plasmids. Heterologous protein expression was carried out at 16 °C using *Escherichia coli* ArcticExpress (Agilent Technologies) grown in Studier’s autoinduction media (ZYP-5052) (Amresco, Solon, Ohio). Total soluble protein was extracted from each culture and the presence of His-tagged recombinant protein was verified by immunoblot procedure according to manufacturer's instructions (SuperSignal West Pico Chemiluminescent Substrate kit, Thermo Scientific). Five alkaloids (canadine, coclaurine, stylopine, reticuline, pavine) were screened in triplicate as potential substrates for *G. flavum* NMTs using a standardized assay under linear product formation conditions (30 μg total protein, 100 μM alkaloid, 200 μM *S*-adenosyl methionine, 100 mM sodium phosphate, pH 7). Total assay volume was 100 μL, and assays proceeded at 30 °C for either 5 or 30 min, depending on the linear range pre-determined for each enzyme. Assays were analyzed by LC-MS/MS as previously described [9]. Most products were identified by comparison with retention times and CID spectra of authentic standards. *N*,*N*-Dimethylcoclaurine was identified by comparing the reaction product CID spectrum with previously reported data [8]. Product formation was monitored relative to empty vector controls. For each enzyme, activity was calculated relative to the assay in which the most substrate conversion was observed (i.e. the latter assay being set to 100 %).

### Availability of supporting data

All sequence data discussed in this paper have been deposited in the National Center for Biotechnology Information Sequence Read Archive (http://www.ncbi.nlm.nih.gov/sra) under the accession numbers listed in Additional files 3 and 4. All phylogenetic data are available in Dryad (http://dx.doi.org/10.5061/dryad.bh276).

## Additional files

Additional file 1:
**Selected examples of BIA structural subgroups derived from the basic benzylisoquinoline subunit.** (PDF 854 kb) Additional file 2:
**Phylogenetic relationships among the Ranunculales as evidenced by molecular loci and morphological data.** Adapted from [50]. (PDF 629 kb) Additional file 3:
**Summary of results obtained using Roche-based deep sequencing platform.** Predicted full-length CDS intersects between Roche-based and Illumina-based transcriptomes are indicated for comparison purposes (see Results and Discussion). (PDF 73 kb) Additional file 4:
**Summary of results obtained using the Illumina-based deep sequencing platform.** (PDF 80 kb) Additional file 5:
**Summary of results obtained using the Illumina-based deep sequencing platform.** For comparison purposes, both stem and root of *Chelidonium majus* were subjected to transcriptome analysis. (PDF 55 kb) Additional file 6:
**FASTA file of all candidates, queries, and outgroups.** (PDF 253 kb) Additional file 7:
**Normalized expression analysis for gene candidates potentially involved in BIA biosynthesis in Papaveraceae (tribe: Fumariaceae) and Ranunculaceae species.** Each candidate is labeled with respective species abbreviations (e.g. CCH, *Corydalis chelanthifolia*) and the type of enzyme potentially encoded by the gene (e.g. BBE, berberine bridge enzyme). Refer to Table 1 and Fig. 2 for species and enzyme/protein family abbreviations, respectively. Expression analysis was not performed for *Mahonia aquifolium* due to reduced numbers of full-length CDSs (see Results and Discussion). Candidates present exclusively in Roche-based transcriptomes could not be assigned an FPKM value, and are marked with asterisks. (PDF 131 kb) Additional file 8:
**Normalized expression analysis for gene candidates potentially involved in BIA biosynthesis in Menispermaceae species.** Each candidate is labeled with respective species abbreviations (e.g. MCA, Menispermum canadense) and the type of enzyme potentially encoded by the gene (e.g. BBE, berberine bridge enzyme). Refer to Table 1 and Fig. 2 for species and enzyme/protein family abbreviations, respectively. Expression analysis was not performed for *Cocculus trilobus* due to reduced numbers of full-length CDSs (see Results and Discussion). Candidates present exclusively in Roche-based transcriptomes could not be assigned an FPKM value, and are marked with asterisks. (PDF 65 kb) Additional file 9:
**Phylogenetic analysis of norcoclaurine synthase (NCS) gene candidates from twenty BIA-accumulating plant species.** Red text denotes characterized genes or enzymes used as tBLASTn queries for transcriptome mining. Black text denotes uncharacterized gene candidates identified through mining (>40 % identity to queries). Bootstrap values for each clade were based on 1000 iterations. Each candidate is labeled with respective species abbreviation (e.g. AME, *Argemone mexicana*; see Table 1) and candidate number (e.g. NCS1). Each query is labeled according to species (additional species: PSO, *Papaver somniferum*). The outgroup is Bet V1 allergen protein from *Betula pendula* (BPE). Amino acid sequences for candidates, queries, and outgroups are found in Additional file 6. (PDF 7248 kb) Additional file 10:
**Phylogenetic analysis of berberine bridge enzyme (BBE) gene candidates from twenty BIA-accumulating plant species.** Red text denotes characterized genes or enzymes used as tBLASTn queries for transcriptome mining. Black text denotes uncharacterized gene candidates identified through mining (>40 % identity to queries). Species for which tBLASTn querying did not yield hits are not represented on the tree. Bootstrap values for each clade were based on 1000 iterations. Each candidate is labeled with respective species abbreviation (e.g. AME, *Argemone mexicana*; see Table 1) and candidate number (e.g. BBE1) where more than one hit was identified. Each query is labeled according to species (additional species: PSO, *Papaver somniferum*; BST, *Berberis stolonifera*). The outgroup is tetrahydrocannabinolic acid synthase (THCAS) from *Cannabis sativa* (CSA). Amino acid sequences for candidates, queries, and outgroups are found in Additional file 6. (PDF 1851 kb) Additional file 11:
**Phylogenetic analysis of FAD-dependent oxidase (FADOX) gene candidates from twenty BIA-accumulating plant species.** Red text denotes characterized genes or enzymes used as tBLASTn queries for transcriptome mining. Black text denotes uncharacterized gene candidates identified through mining (>40 % identity to queries). Species for which tBLASTn querying did not yield hits are not represented on the tree. Bootstrap values for each clade were based on 1000 iterations. Each candidate is labeled with respective species abbreviation (e.g. AME, *Argemone mexicana*; see Table 1). Each query is labeled according to species (PSO, *Papaver somniferum*; CJA, *Coptis japonica*; BWI, *Berberis wilsoniae*). The outgroup is tetrahydrocannabinolic acid synthase (THCAS) from *Cannabis sativa* (CSA). Amino acid sequences for candidates, queries, and outgroups are found in Additional file 6. (PDF 4025 kb) Additional file 12:
**Phylogenetic analysis of 2-oxoglutarate/iron (II)-dependent dioxygenase (DIOX) gene candidates from twenty BIA-accumulating plant species.** Red text denotes characterized genes or enzymes used as tBLASTn queries for transcriptome mining. Black text denotes uncharacterized gene candidates identified through mining (>40 % identity to queries). Bootstrap values for each clade were based on 1000 iterations. Each candidate is labeled with respective species abbreviation (e.g. AME, *Argemone mexicana*; see Table 1) and candidate number (e.g. DIOX1). The queries are derived from *Papaver somniferum* (PSO). The outgroup is anthocyanidin synthase from *Arabidopsis thaliana* (labeled ATHDIOX). Amino acid sequences for candidates, queries, and outgroups are found in Additional file 6. (PDF 20162 kb) Additional file 13:
**Phylogenetic analysis of aldo-keto reductase gene candidates with homology to codeinone reductase (COR) from twenty BIA-accumulating plant species.** Red text denotes the characterized COR1.3 from *Papaver somniferum* (PSO) used as a tBLASTn query for transcriptome mining. Black text denotes uncharacterized gene candidates identified through mining (>40 % identity to query). Bootstrap values for each clade were based on 1000 iterations. Each candidate is labeled with respective species abbreviation (e.g. AME, *Argemone mexicana*; see Table 1) and candidate number (e.g. COR1). The outgroup is deoxymugineic acid synthase (DMAS) from *Zea mays* (ZMA). Amino acid sequences for candidates, queries, and outgroups are found in Additional file 6. (PDF 12593 kb) Additional file 14:
**Phylogenetic analysis of short chain dehydrogenase/reductase gene candidates with homology to salutaridine reductase (SALR) from twenty BIA-accumulating plant species.** Red text denotes the characterized SALR from *Papaver somniferum* (PSO) used as a tBLASTn query for transcriptome mining. Black text denotes uncharacterized gene candidates identified through mining (>40 % identity to query). Species for which tBLASTn querying did not yield hits are not represented on the tree. Bootstrap values for each clade were based on 1000 iterations. Each candidate is labeled with respective species abbreviation (e.g. AME, *Argemone mexicana*; see Table 1) and candidate number (e.g. SALR1). The outgroup is porcine testicular carbonyl reductase (PTCR) from *Sus scrofa* (SSC). Amino acid sequences for candidates, queries, and outgroups are found in Additional file 6. (PDF 7467 kb) Additional file 15:
**Phylogenetic analysis of short chain dehydrogenase/reductase gene candidates with homology to sanguinarine reductase (SANR) from twenty BIA-accumulating plant species.** Red text denotes the characterized SANR1 from *Eschschotzia californica* (ECA) used as a tBLASTn query for transcriptome mining. Black text denotes uncharacterized gene candidates identified through mining (>40 % identity to query). Bootstrap values for each clade were based on 1000 iterations. Each candidate is labeled with respective species abbreviation (e.g. AME, *Argemone mexicana*; see Table 1) and candidate number (e.g. SANR1). The outgroup is a SanR-like protein from *Zea mays* (ZMASDR). Amino acid sequences for candidates, queries, and outgroups are found in Additional file 6. (PDF 11309 kb) Additional file 16:
**Phylogenetic analysis of CYP80 gene candidates from twenty BIA-accumulating plant species.** Red text denotes characterized genes or enzymes used as tBLASTn queries for transcriptome mining. Black text denotes uncharacterized gene candidates identified through mining (>40 % identity to queries). Bootstrap values for each clade were based on 1000 iterations. Each candidate is labeled with respective species abbreviation (e.g. AME, *Argemone mexicana*; see Table 1) and candidate number (e.g. CYP80-1). Each query is labeled according to species (additional species: HNI, *Hyoscymus niger*; CJA, *Coptis japonica*; BST, *Berberis stolonifera*; PSO, *Papaver somniferum*) with CYP80 subfamily and gene number indicated (e.g. CYP80A1, corytuberine synthase; see Fig. 1). The outgroup is CYP1B1 from *Homo sapiens* (HSA). Amino acid sequences for candidates, queries, and outgroups are found in Additional file 6. (PDF 13027 kb) Additional file 17:
**Phylogenetic analysis of CYP82 gene candidates from twenty BIA-accumulating plant species.** Red text denotes characterized genes or enzymes used as tBLASTn queries for transcriptome mining. Black text denotes uncharacterized gene candidates identified through mining (>40 % identity to queries). Bootstrap values for each clade were based on 1000 iterations. Each candidate is labeled with respective species abbreviation (e.g. AME, *Argemone mexicana*; see Table 1) and candidate number (e.g. CYP82-1). Each query is labeled according to species (additional species: PSO, *Papaver somniferum*) with CYP82 subfamily and gene number indicated (e.g. CYP82N3, protopine hydroxylase; see Fig. 1). The outgroup is CYP82E4 from *Nicotiana tabacum* (NTA). Amino acid sequences for candidates, queries, and outgroups are found in Additional file 6. (PDF 7660 kb) Additional file 18:
**Phylogenetic analysis of** ***O*** **-methyltransferase (OMT) gene candidates from twenty BIA-accumulating plant species.** Red text denotes characterized genes or enzymes used as tBLASTn queries for transcriptome mining. Black text denotes uncharacterized gene candidates identified through mining (>40 % identity to queries). Bootstrap values for each clade were based on 1000 iterations. Each candidate is labeled with respective species abbreviation (e.g. AME, *Argemone mexicana*; see Table 1) and candidate number (e.g. OMT1). Each query is labeled according to species (additional species: PSO, *Papaver somniferum*; CJA, *Coptis japonica*) and specific OMT function (SOMT, scoulerine *O*-methyltransferase; CbOMT, columbamine *O*-methyltransferase; N7OMT, norreticuline 7-*O*-methyltransferase; 7OMT; 6OMT; 4'OMT; see Fig. 1). The outgroup is isoflavone *O*-methyltransferase from *Medicago sativa* (MSA). Amino acid sequences for candidates, queries, and outgroups are found in Additional file 6. (PDF 13001 kb) Additional file 19:
**Phylogenetic analysis of BAHD-type** ***O*** **-acetyltransferase (OAT; D'Aura 2006) gene candidates from twenty BIA-accumulating plant species.** Red text denotes the characterized AT1 (1,13-dihydroxy-*N*-methylcanadine *O*-acetyltransferase; [6]) and SAT (salutaridine synthase) from *Papaver somniferum* (PSO) used as tBLASTn queries for transcriptome mining. Black text denotes uncharacterized gene candidates identified through mining (>30 % identity to query). Bootstrap values for each clade were based on 1000 iterations. Each candidate is labeled with respective species abbreviation (e.g. AME, *Argemone mexicana*; see Table 1) and candidate number (e.g. AT1). The outgroup is vinorine synthase from *Rauvolfia serpentina* (RSEVS). Amino acid sequences for candidates, queries, and outgroups are found in Additional file 6. (PDF 5574 kb) Additional file 20:
**Phylogenetic analysis of carboxyl esterase (CXE) gene candidates from twenty BIA-accumulating plant species.** Red text denotes the characterized CXE1 (3-*O*-acetylpapaveroxine carboxylesterase; [6]) from *Papaver somniferum* (PSO) used as a tBLASTn query for transcriptome mining. Black text denotes uncharacterized gene candidates identified through mining (>30 % identity to query). Bootstrap values for each clade were based on 1000 iterations. Each candidate is labeled with respective species abbreviation (e.g. AME, *Argemone mexicana*; see Table 1) and candidate number (e.g. CXE1). The outgroup is carboxylesterase 1 from *Actinidia eriantha* (AERCXE). Amino acid sequences for candidates, queries, and outgroups are found in Additional file 6. (PDF 6985 kb) Additional file 21:
**Phylogenetic analysis of short chain dehydrogenase/reductase gene candidates with homology to noscapine synthase (NOS;** **[** 6 **])** **from 20 BIA-accumulating plant species.** Red text denotes the characterized NOS from *Papaver somniferum* (PSO) used as a tBLASTn query for transcriptome mining. Black text denotes uncharacterized gene candidates identified through mining (>40 % identity to query). Bootstrap values for each clade were based on 1000 iterations. Each candidate is labeled with respective species abbreviation (e.g. AME, *Argemone mexicana*; see Table 1) and candidate number (e.g. NOS1). The outgroup is ben1-1D (BEN1) from *Arabidopsis thaliana*. Amino acid sequences for candidates, queries, and outgroups are found in Additional file 6. (PDF 8376 kb) Additional file 22:
**Immunoblot analysis revealing the presence of His-tagged recombinant protein in soluble extracts of** ***Escherichia coli*** **strains expressing one of six** ***N*** **-methyltransferase candidates (GFLNMT1-6) from** ***Glaucium flavum***. (PDF 203 kb)

## Abbreviations

AKR: Aldo-keto reductase
BBE: Berberine bridge enzyme
BIA: Benzylisoquinoline alkaloid
CDS: Coding sequence
COR: Codeinone reductase
CXE: Carboxylesterase
CYP: Cytochrome P450 monooxygenase
DIOX: 2-oxoglutarate/Fe^2+^-dependent dioxygenase
FADX: FAD-dependent oxidoreductase, FPKM, Fragments per kilobase of exon model per million mapped reads
OAT: *O*-acetyltransferase
OMT: *O*-methyltransferase
NCS: Norcoclaurine synthase
NOS: Noscapine synthase
SalR: Salutaridine reductase
SanR: Sanguinarine reductase
