# Supplementary material for: Transcriptome analysis of 20 taxonomically related benzylisoquinoline alkaloid-producing plants
Source: BMC Plant Biol. 2015 Sep 18;15:227. doi: 10.1186/s12870-015-0596-0 (PMC4575454; doi:10.1186/s12870-015-0596-0)
Supplement: Additional file 1: — Selected examples of BIA structural subgroups derived from the basic benzylisoquinoline subunit. (PDF 854 kb) [file 12870_2015_596_MOESM1_ESM.pdf]

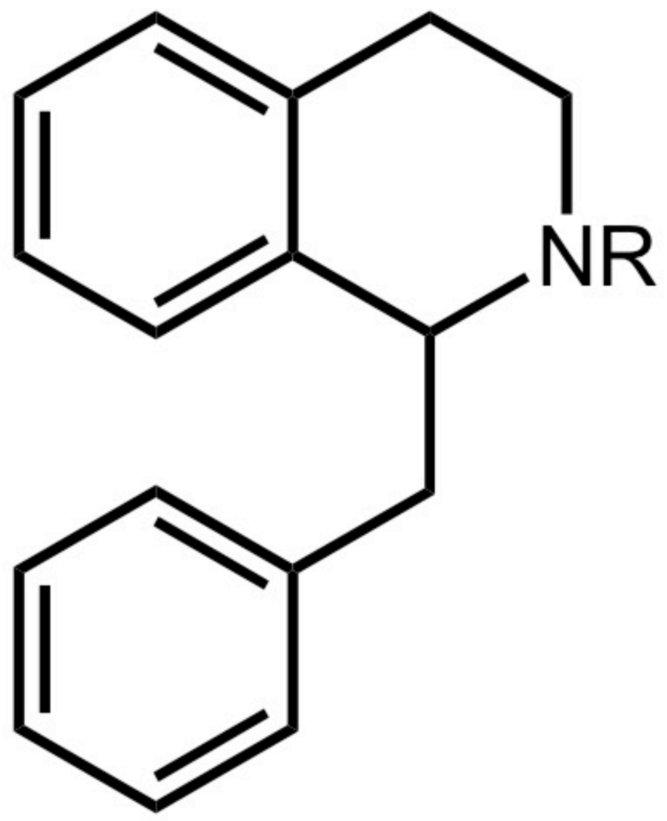

1-Benzylisoquinoline

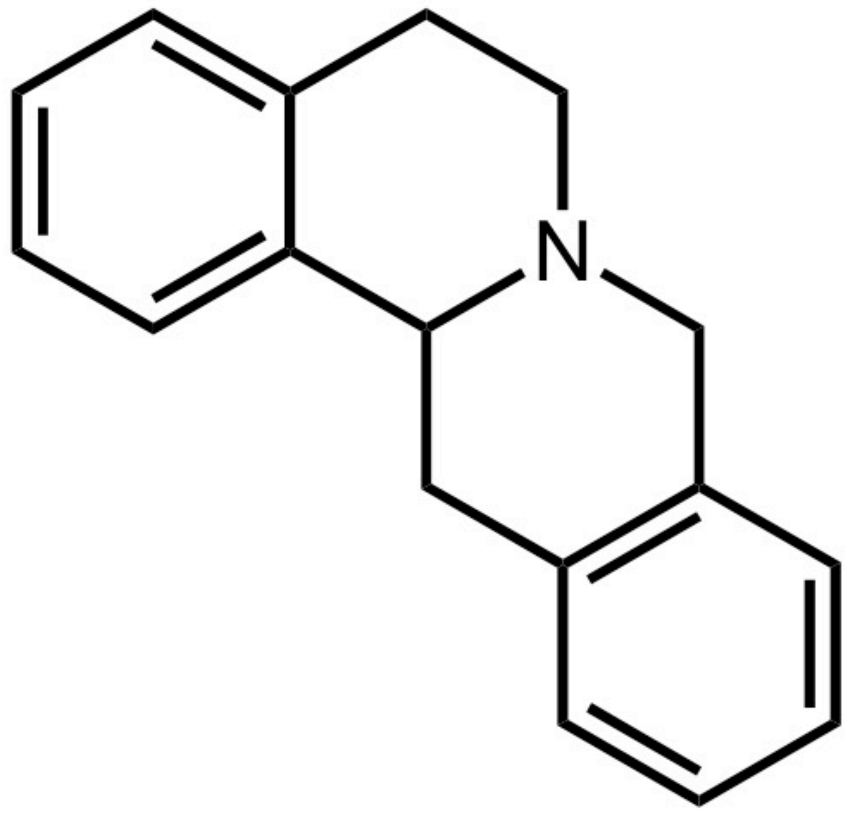

Protoberberine

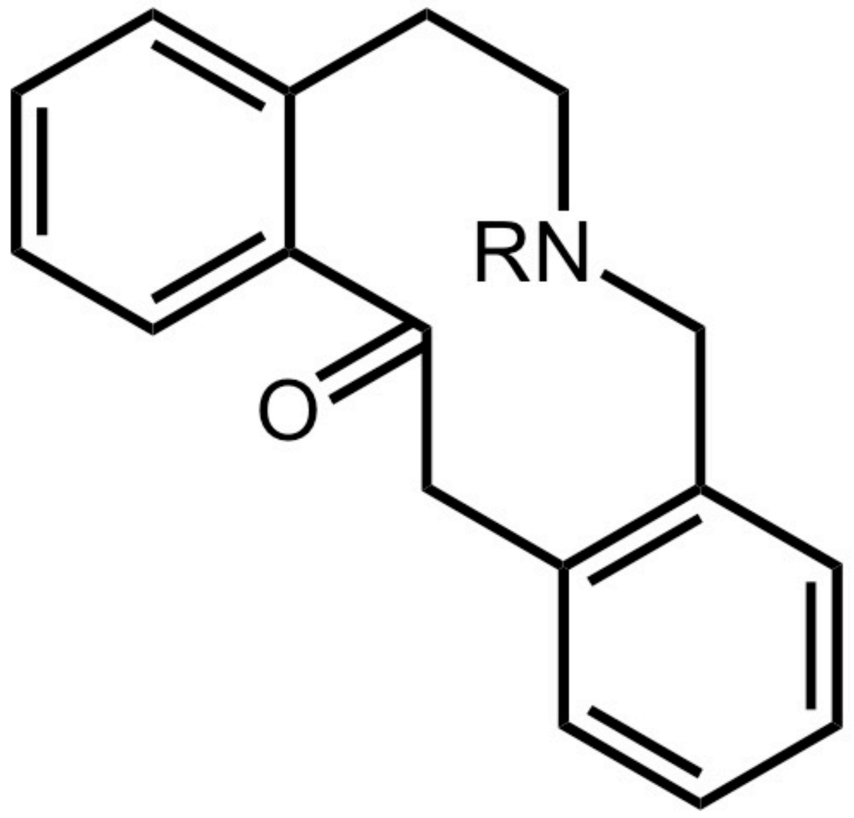

Protopine

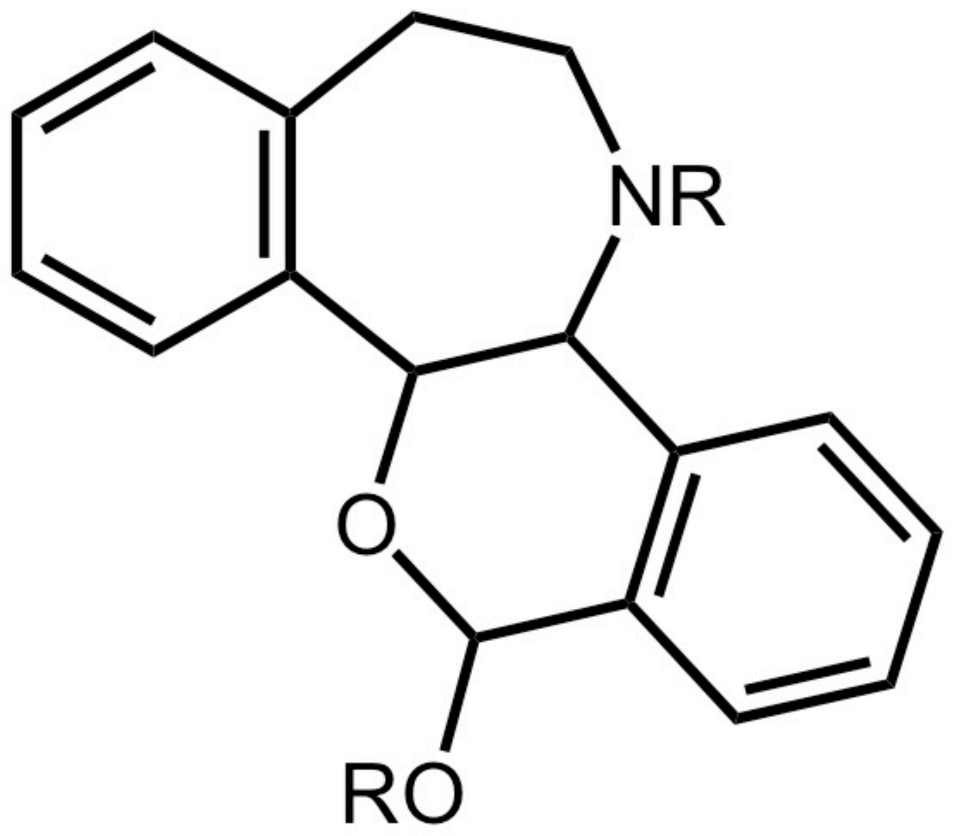

Papaverubine

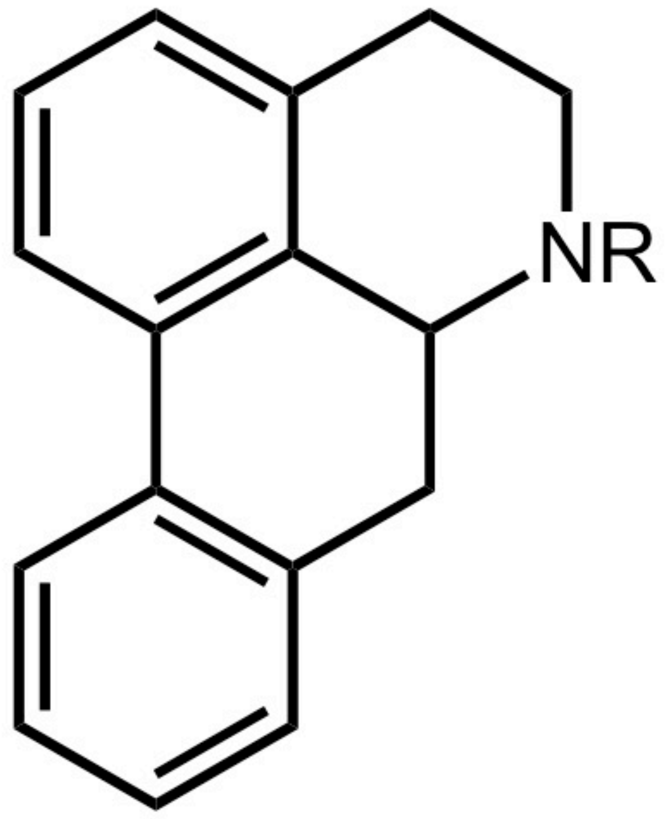

Aporphine

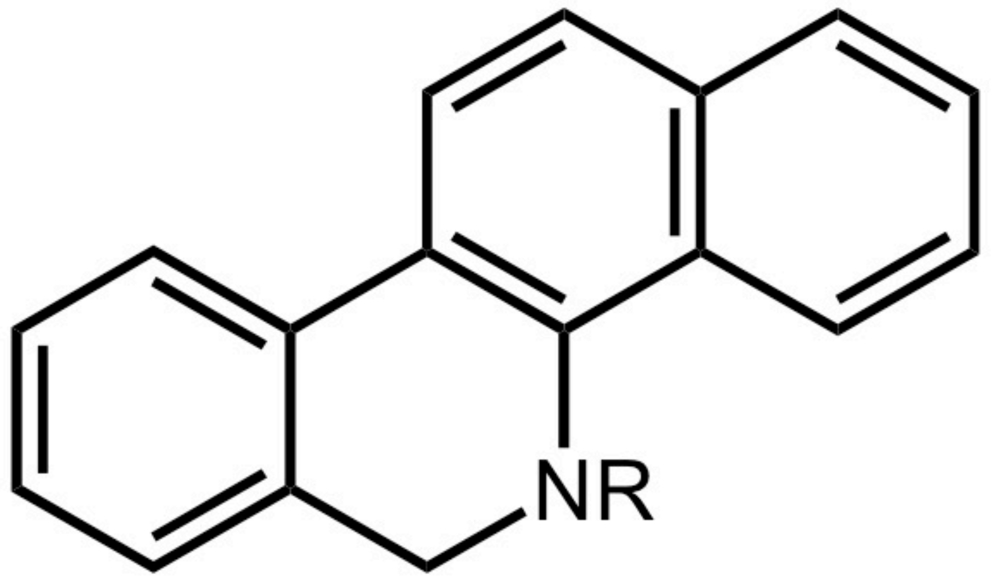

Benzo[*c*]phenanthridine

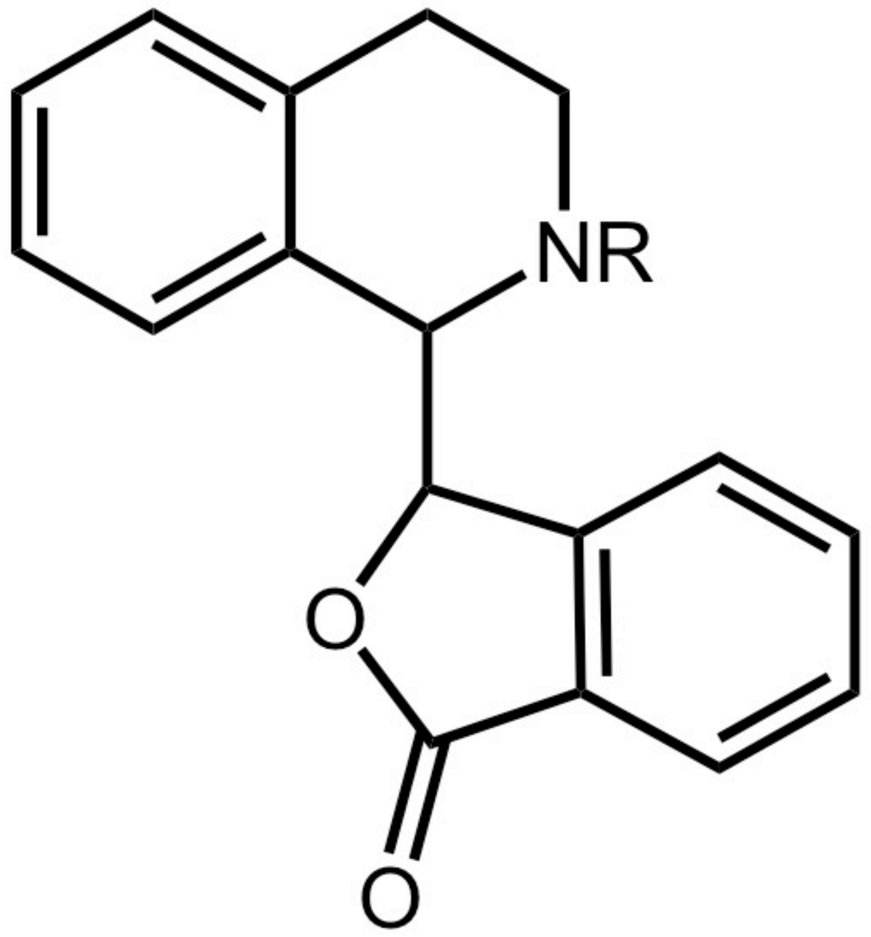

Phthalideisoquinoline

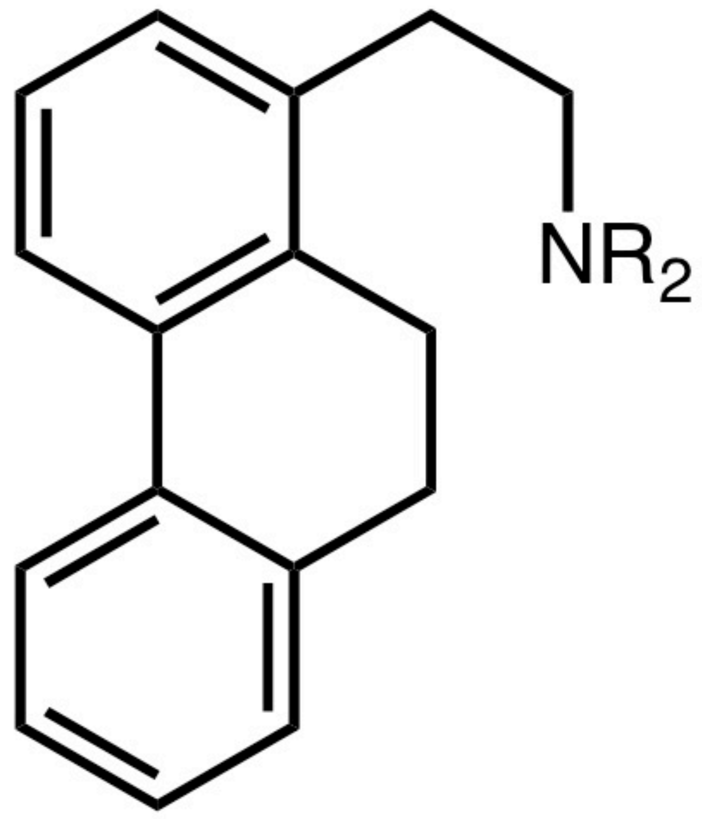

Secoisoquinoline

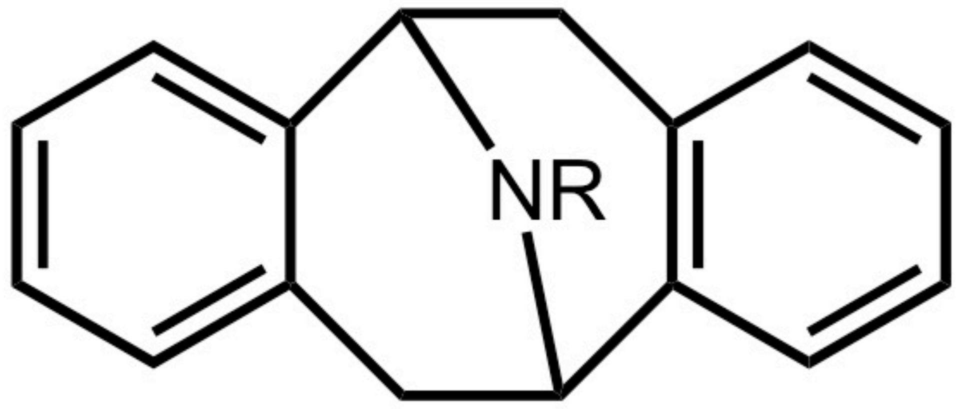

Pavine

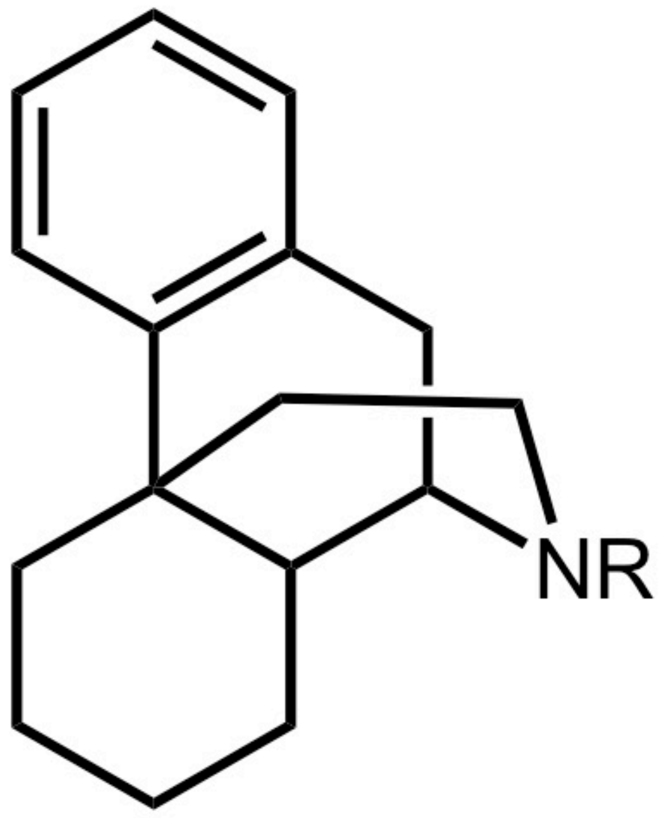

Promorphinan

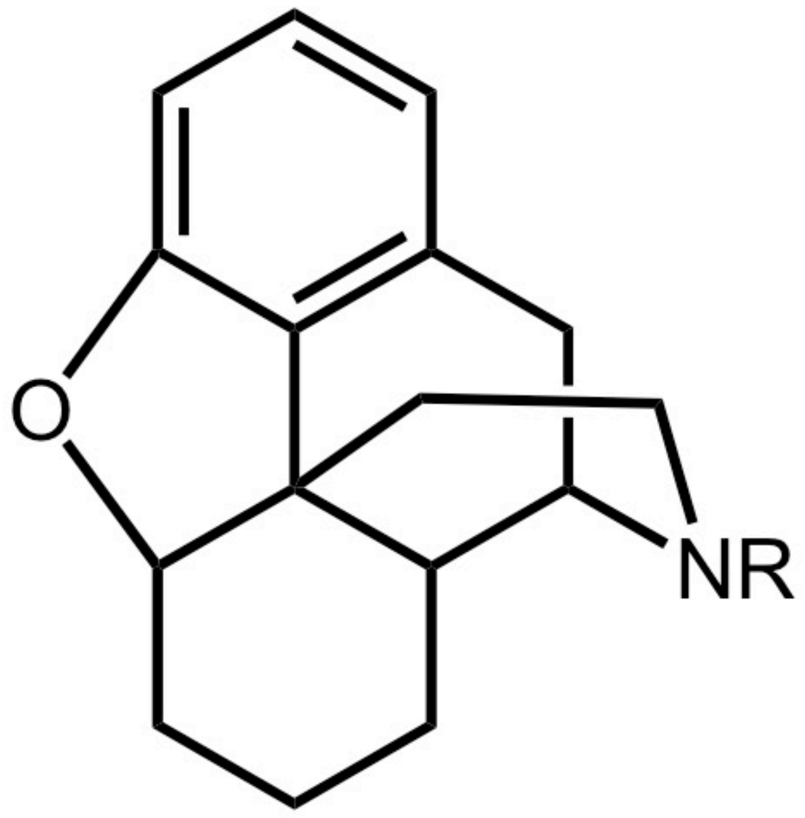

Morphinan

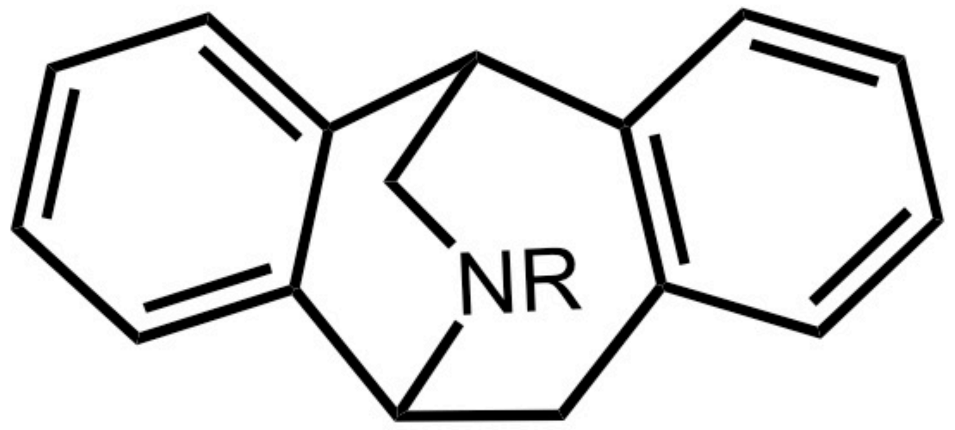

Isopavine

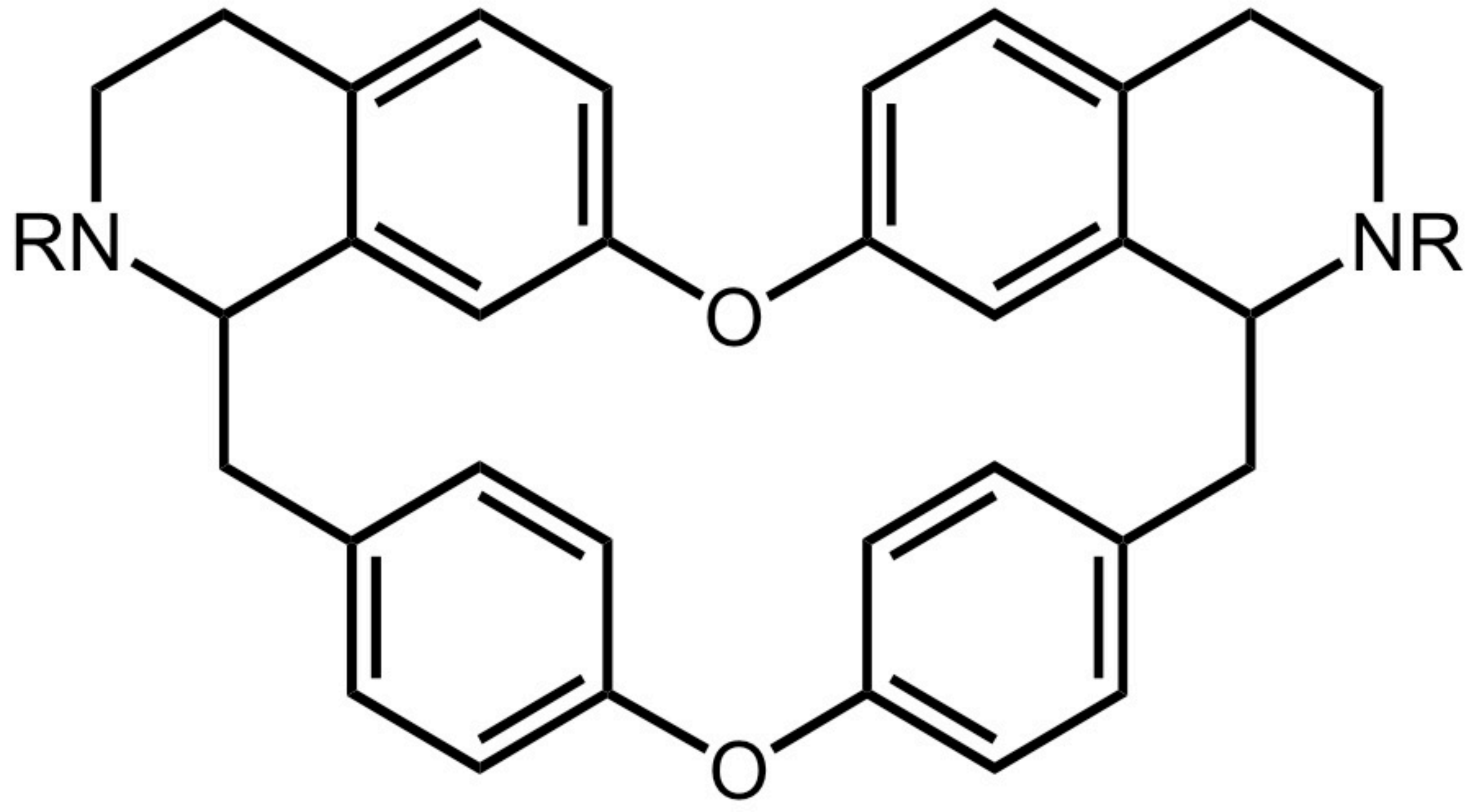

Bisbenzylisoquinoline
