# Supplementary figures and images for: Transcriptome analysis of 20 taxonomically related benzylisoquinoline alkaloid-producing plants
Source: BMC Plant Biol. 2015 Sep 18;15:227. doi: 10.1186/s12870-015-0596-0 (PMC4575454; doi:10.1186/s12870-015-0596-0)

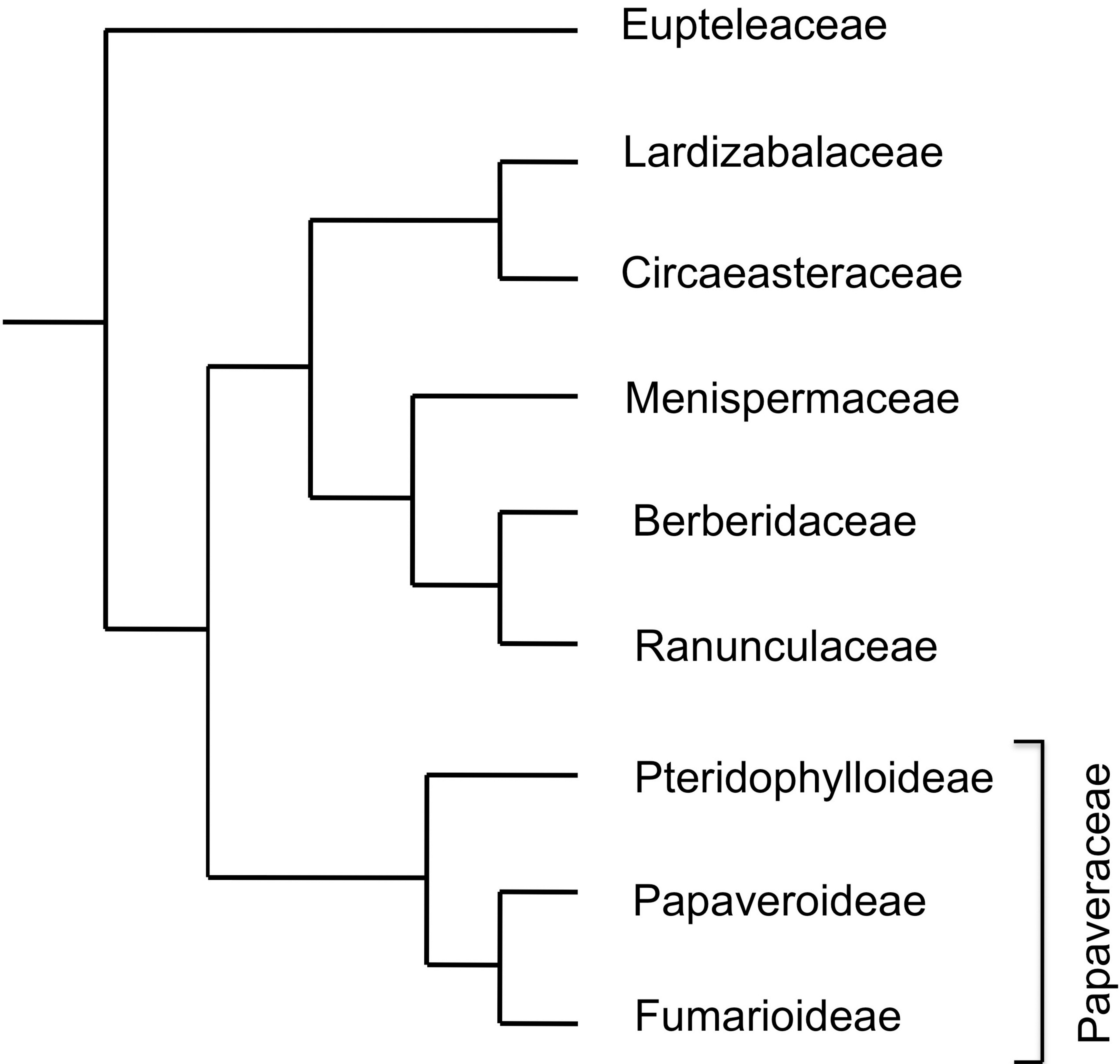

Supplement: Additional file 2: — Phylogenetic relationships among the Ranunculales as evidenced by molecular loci and morphological data. Adapted from [50]. (PDF 629 kb) [file 12870_2015_596_MOESM2_ESM.pdf]

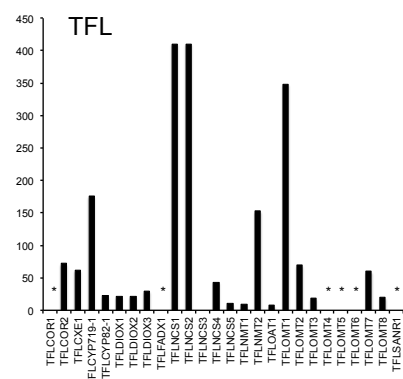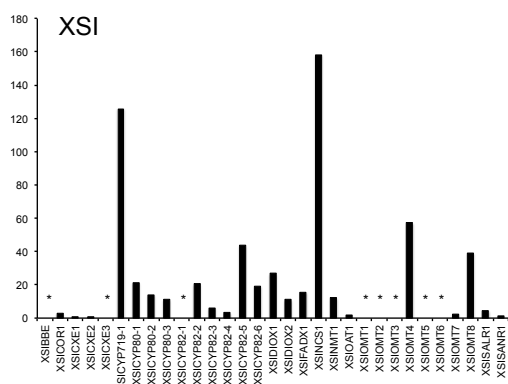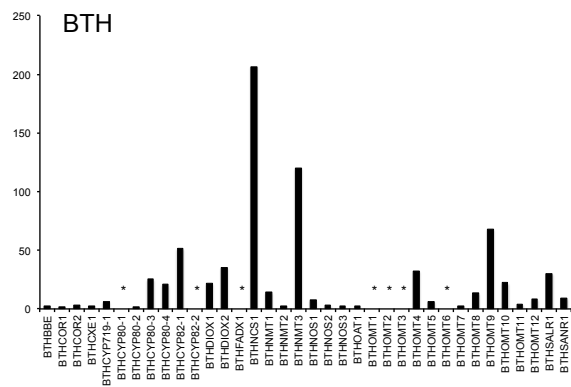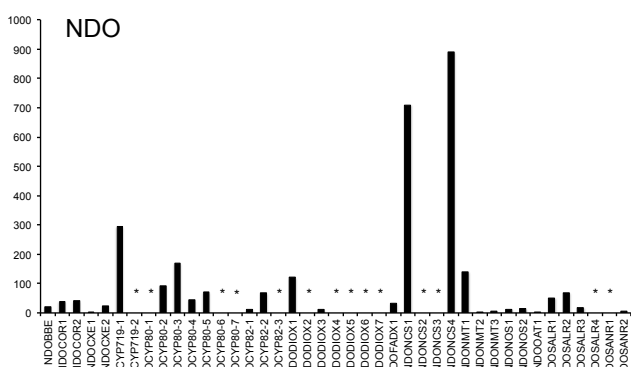

Supplement: Additional file 7: — Normalized expression analysis for gene candidates potentially involved in BIA biosynthesis in Papaveraceae (tribe: Fumariaceae) and Ranunculaceae species. Each candidate is labeled with respective species abbreviations (e.g. CCH, Corydalis chelanthifolia) and the type of enzyme potentially encoded by the gene (e.g. BBE, berberine bridge enzyme). Refer to Table 1 and Fig. 2 for species and enzyme/protein family abbreviations, respectively. Expression analysis was not performed for Mahonia aquifolium due to reduced numbers of full-length CDSs (see Results and Discussion). Candidates present exclusively in Roche-based transcriptomes could not be assigned an FPKM value, and are marked with asterisks. (PDF 131 kb) [file 12870_2015_596_MOESM7_ESM.pdf]

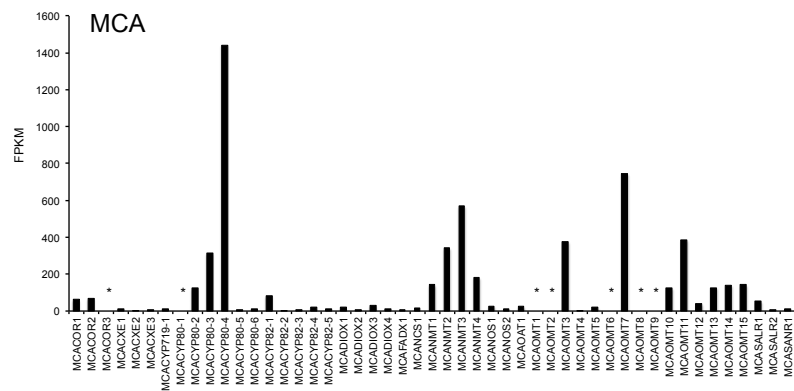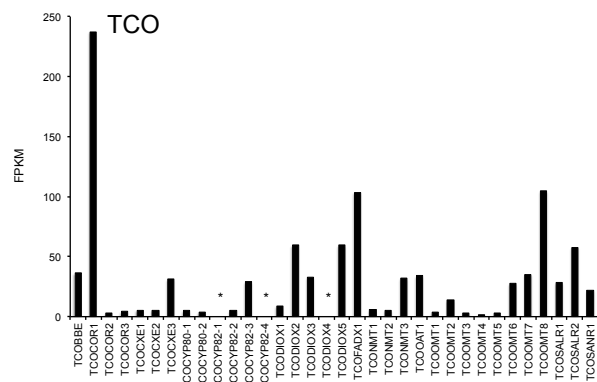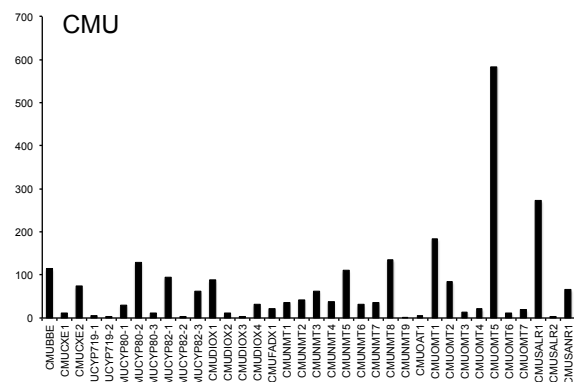

Supplement: Additional file 8: — Normalized expression analysis for gene candidates potentially involved in BIA biosynthesis in Menispermaceae species. Each candidate is labeled with respective species abbreviations (e.g. MCA, Menispermum canadense) and the type of enzyme potentially encoded by the gene (e.g. BBE, berberine bridge enzyme). Refer to Table 1 and Fig. 2 for species and enzyme/protein family abbreviations, respectively. Expression analysis was not performed for Cocculus trilobus due to reduced numbers of full-length CDSs (see Results and Discussion). Candidates present exclusively in Roche-based transcriptomes could not be assigned an FPKM value, and are marked with asterisks. (PDF 65 kb) [file 12870_2015_596_MOESM8_ESM.pdf]

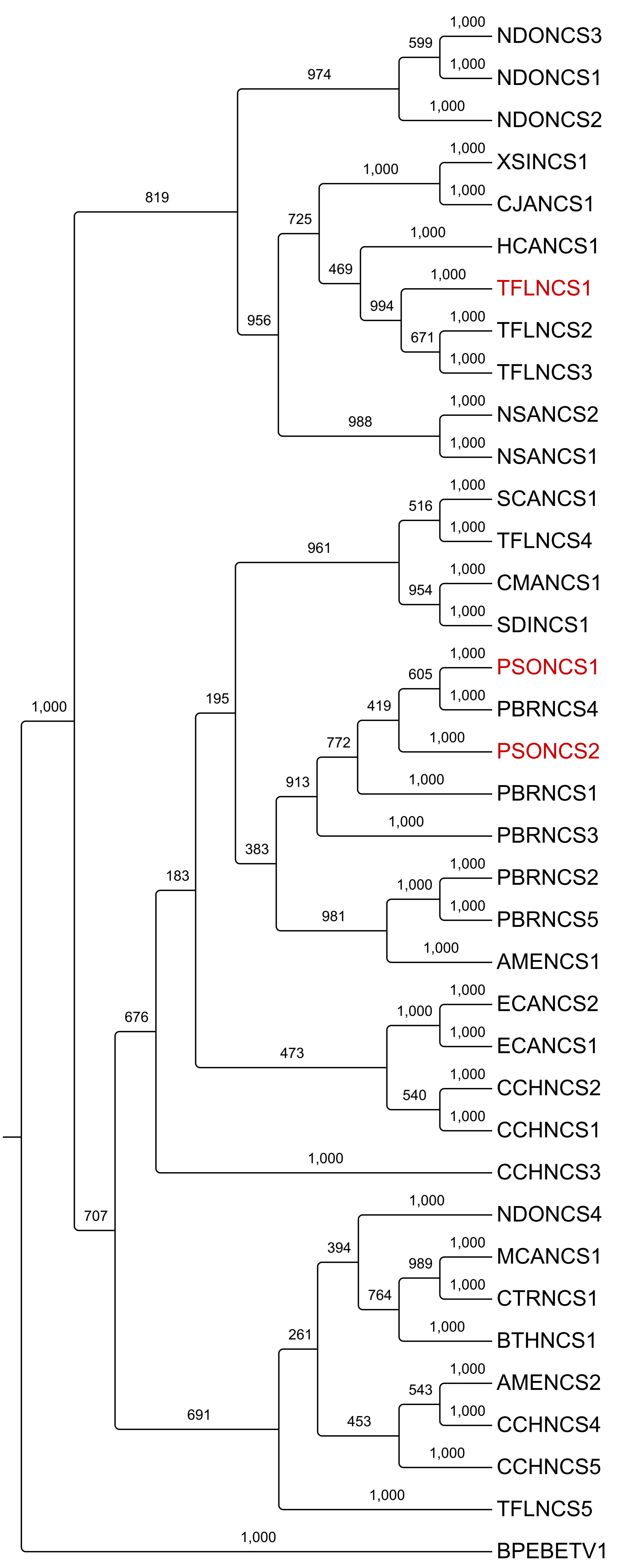

Supplement: Additional file 9: — Phylogenetic analysis of norcoclaurine synthase (NCS) gene candidates from twenty BIA-accumulating plant species. Red text denotes characterized genes or enzymes used as tBLASTn queries for transcriptome mining. Black text denotes uncharacterized gene candidates identified through mining (>40 % identity to queries). Bootstrap values for each clade were based on 1000 iterations. Each candidate is labeled with respective species abbreviation (e.g. AME, Argemone mexicana; see Table 1) and candidate number (e.g. NCS1). Each query is labeled according to species (additional species: PSO, Papaver somniferum). The outgroup is Bet V1 allergen protein from Betula pendula (BPE). Amino acid sequences for candidates, queries, and outgroups are found in Additional file 6. (PDF 7248 kb) [file 12870_2015_596_MOESM9_ESM.pdf]

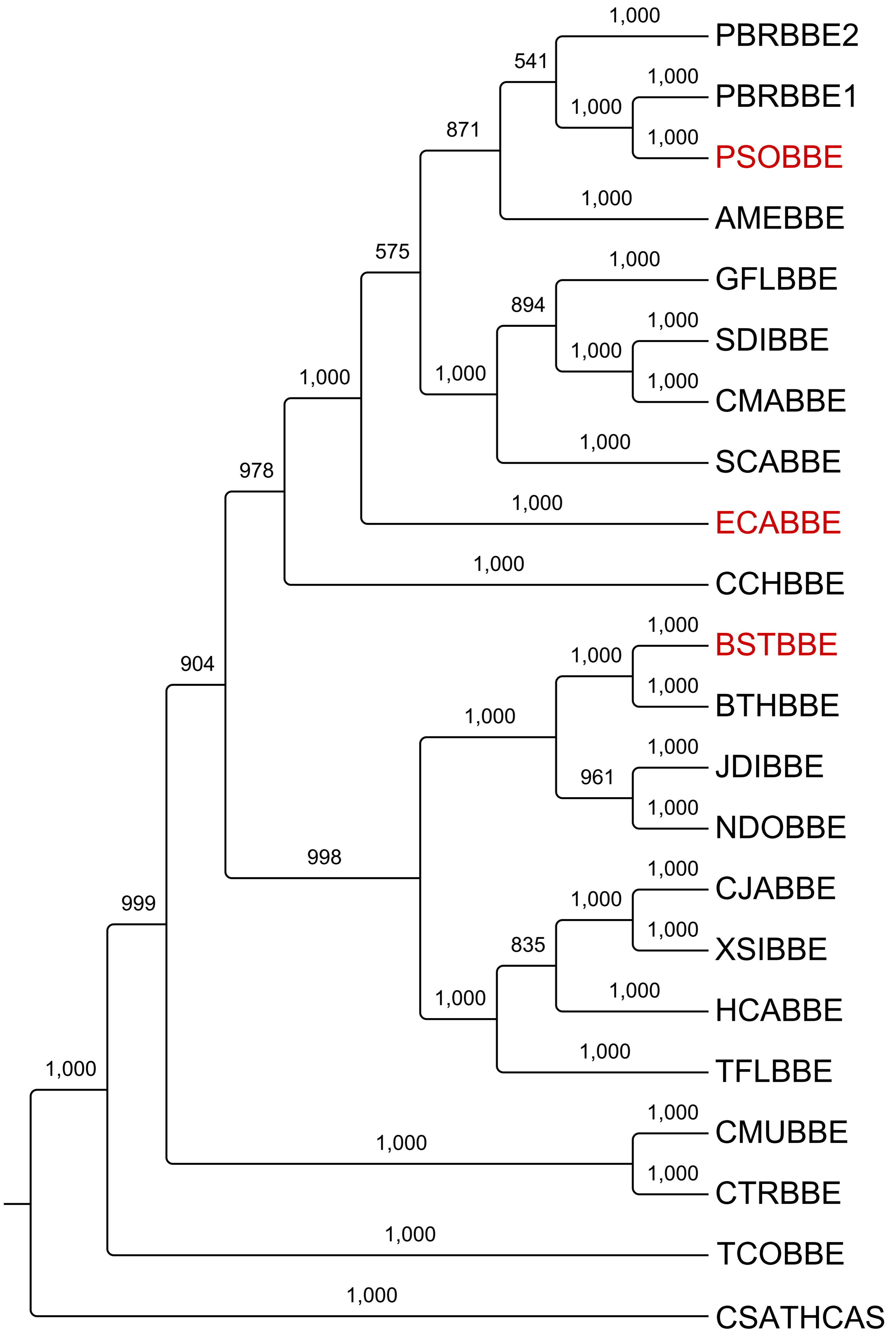

Supplement: Additional file 10: — Phylogenetic analysis of berberine bridge enzyme (BBE) gene candidates from twenty BIA-accumulating plant species. Red text denotes characterized genes or enzymes used as tBLASTn queries for transcriptome mining. Black text denotes uncharacterized gene candidates identified through mining (>40 % identity to queries). Species for which tBLASTn querying did not yield hits are not represented on the tree. Bootstrap values for each clade were based on 1000 iterations. Each candidate is labeled with respective species abbreviation (e.g. AME, Argemone mexicana; see Table 1) and candidate number (e.g. BBE1) where more than one hit was identified. Each query is labeled according to species (additional species: PSO, Papaver somniferum; BST, Berberis stolonifera). The outgroup is tetrahydrocannabinolic acid synthase (THCAS) from Cannabis sativa (CSA). Amino acid sequences for candidates, queries, and outgroups are found in Additional file 6. (PDF 1851 kb) [file 12870_2015_596_MOESM10_ESM.pdf]

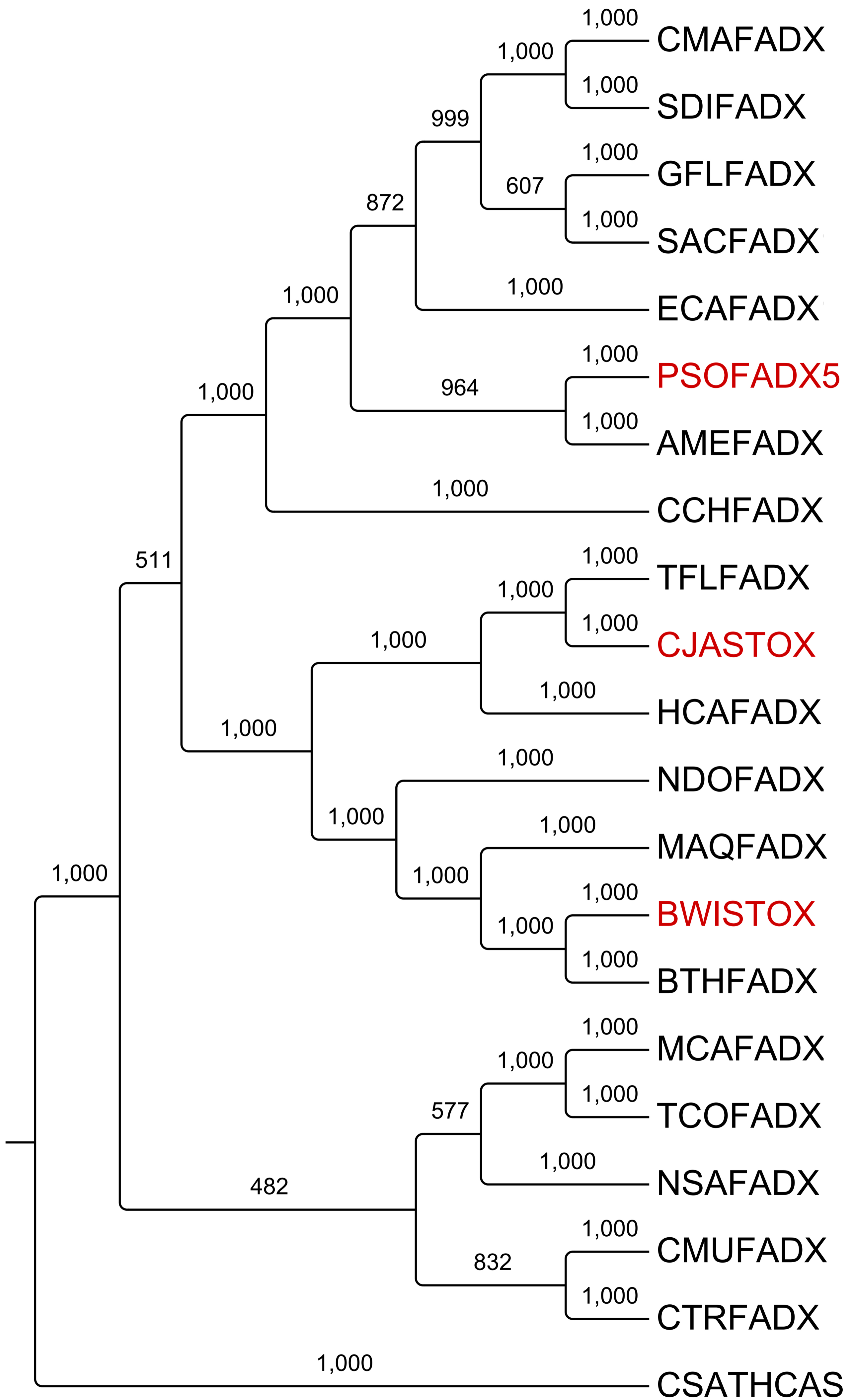

Supplement: Additional file 11: — Phylogenetic analysis of FAD-dependent oxidase (FADOX) gene candidates from twenty BIA-accumulating plant species. Red text denotes characterized genes or enzymes used as tBLASTn queries for transcriptome mining. Black text denotes uncharacterized gene candidates identified through mining (>40 % identity to queries). Species for which tBLASTn querying did not yield hits are not represented on the tree. Bootstrap values for each clade were based on 1000 iterations. Each candidate is labeled with respective species abbreviation (e.g. AME, Argemone mexicana; see Table 1). Each query is labeled according to species (PSO, Papaver somniferum; CJA, Coptis japonica; BWI, Berberis wilsoniae). The outgroup is tetrahydrocannabinolic acid synthase (THCAS) from Cannabis sativa (CSA). Amino acid sequences for candidates, queries, and outgroups are found in Additional file 6. (PDF 4025 kb) [file 12870_2015_596_MOESM11_ESM.pdf]

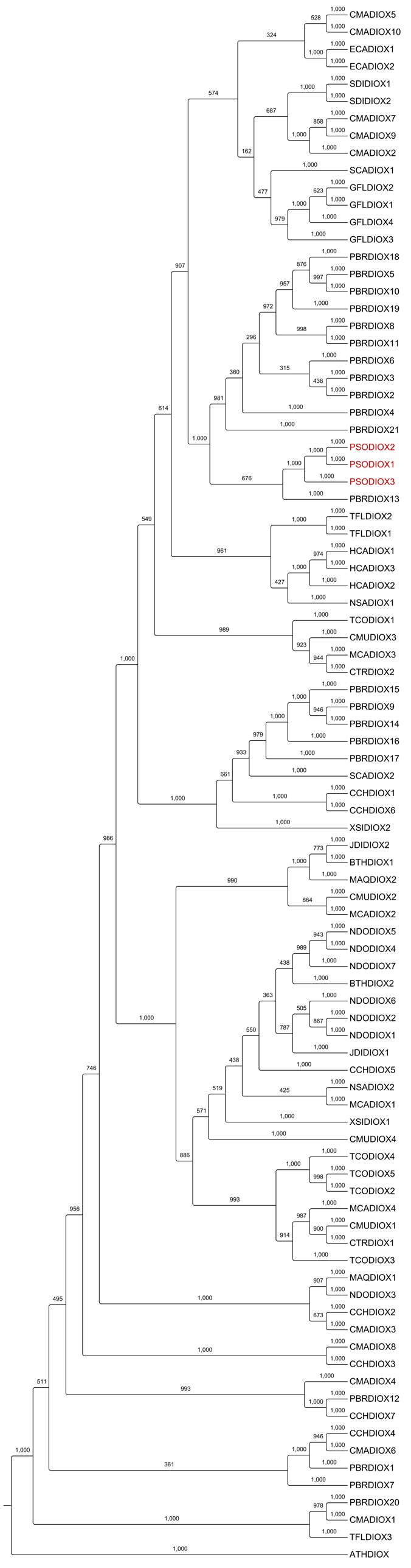

Supplement: Additional file 12: — Phylogenetic analysis of 2-oxoglutarate/iron (II)-dependent dioxygenase (DIOX) gene candidates from twenty BIA-accumulating plant species. Red text denotes characterized genes or enzymes used as tBLASTn queries for transcriptome mining. Black text denotes uncharacterized gene candidates identified through mining (>40 % identity to queries). Bootstrap values for each clade were based on 1000 iterations. Each candidate is labeled with respective species abbreviation (e.g. AME, Argemone mexicana; see Table 1) and candidate number (e.g. DIOX1). The queries are derived from Papaver somniferum (PSO). The outgroup is anthocyanidin synthase from Arabidopsis thaliana (labeled ATHDIOX). Amino acid sequences for candidates, queries, and outgroups are found in Additional file 6. (PDF 20162 kb) [file 12870_2015_596_MOESM12_ESM.pdf]

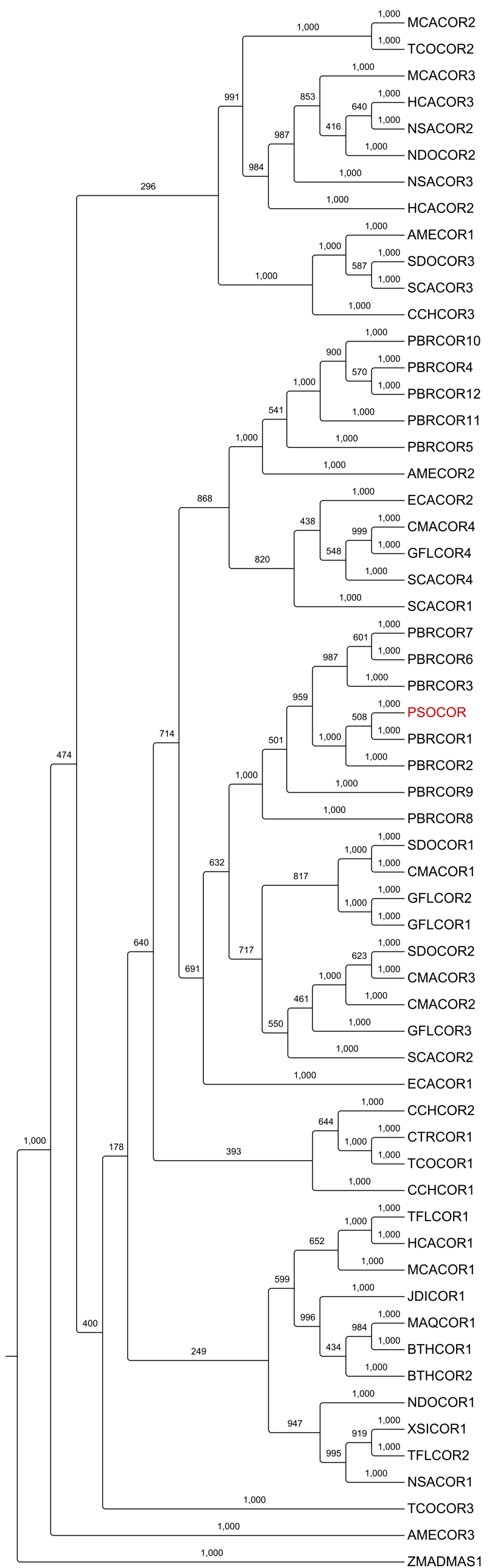

Supplement: Additional file 13: — Phylogenetic analysis of aldo-keto reductase gene candidates with homology to codeinone reductase (COR) from twenty BIA-accumulating plant species. Red text denotes the characterized COR1.3 from Papaver somniferum (PSO) used as a tBLASTn query for transcriptome mining. Black text denotes uncharacterized gene candidates identified through mining (>40 % identity to query). Bootstrap values for each clade were based on 1000 iterations. Each candidate is labeled with respective species abbreviation (e.g. AME, Argemone mexicana; see Table 1) and candidate number (e.g. COR1). The outgroup is deoxymugineic acid synthase (DMAS) from Zea mays (ZMA). Amino acid sequences for candidates, queries, and outgroups are found in Additional file 6. (PDF 12593 kb) [file 12870_2015_596_MOESM13_ESM.pdf]

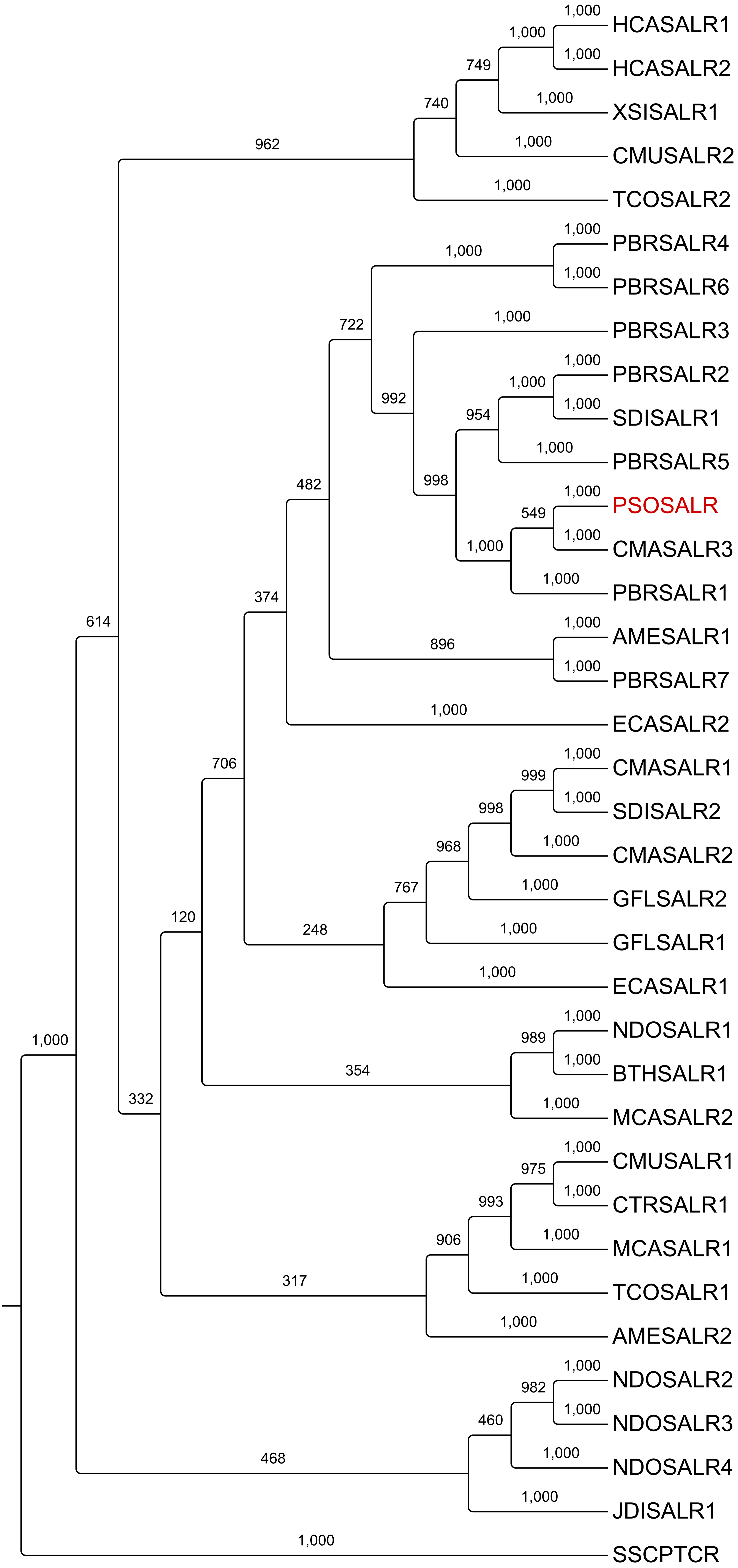

Supplement: Additional file 14: — Phylogenetic analysis of short chain dehydrogenase/reductase gene candidates with homology to salutaridine reductase (SALR) from twenty BIA-accumulating plant species. Red text denotes the characterized SALR from Papaver somniferum (PSO) used as a tBLASTn query for transcriptome mining. Black text denotes uncharacterized gene candidates identified through mining (>40 % identity to query). Species for which tBLASTn querying did not yield hits are not represented on the tree. Bootstrap values for each clade were based on 1000 iterations. Each candidate is labeled with respective species abbreviation (e.g. AME, Argemone mexicana; see Table 1) and candidate number (e.g. SALR1). The outgroup is porcine testicular carbonyl reductase (PTCR) from Sus scrofa (SSC). Amino acid sequences for candidates, queries, and outgroups are found in Additional file 6. (PDF 7467 kb) [file 12870_2015_596_MOESM14_ESM.pdf]

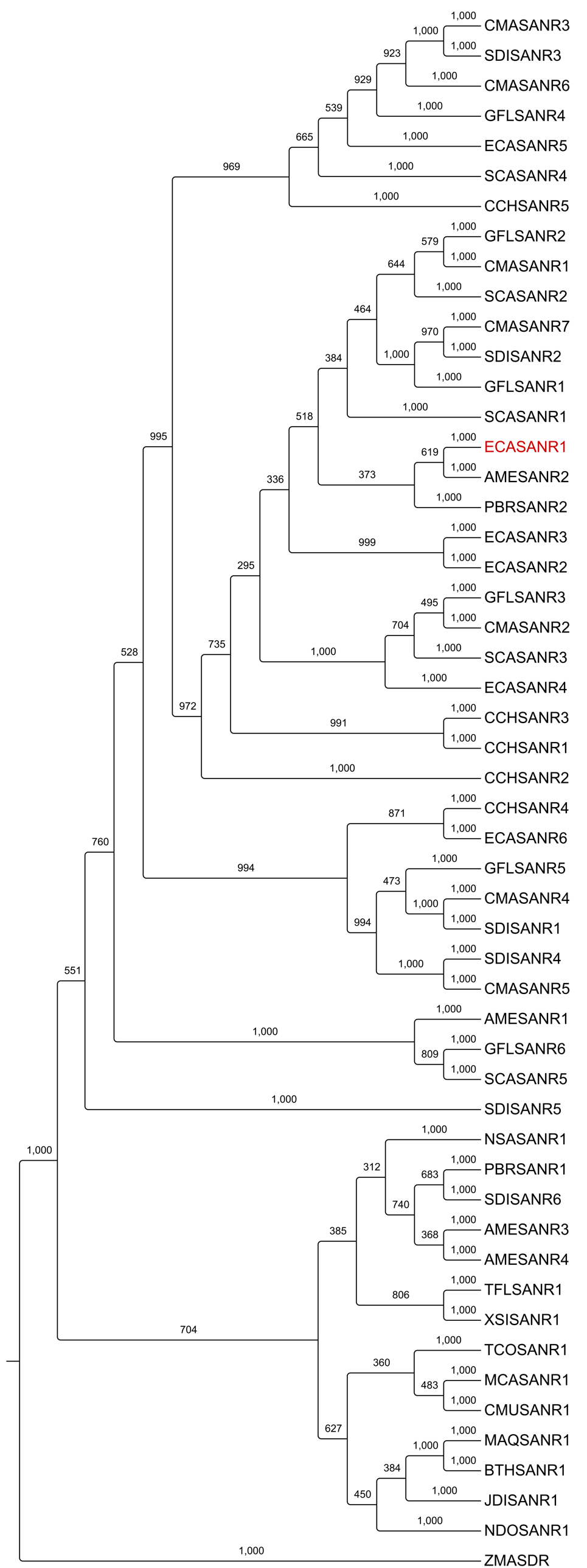

Supplement: Additional file 15: — Phylogenetic analysis of short chain dehydrogenase/reductase gene candidates with homology to sanguinarine reductase (SANR) from twenty BIA-accumulating plant species. Red text denotes the characterized SANR1 from Eschschotzia californica (ECA) used as a tBLASTn query for transcriptome mining. Black text denotes uncharacterized gene candidates identified through mining (>40 % identity to query). Bootstrap values for each clade were based on 1000 iterations. Each candidate is labeled with respective species abbreviation (e.g. AME, Argemone mexicana; see Table 1) and candidate number (e.g. SANR1). The outgroup is a SanR-like protein from Zea mays (ZMASDR). Amino acid sequences for candidates, queries, and outgroups are found in Additional file 6. (PDF 11309 kb) [file 12870_2015_596_MOESM15_ESM.pdf]

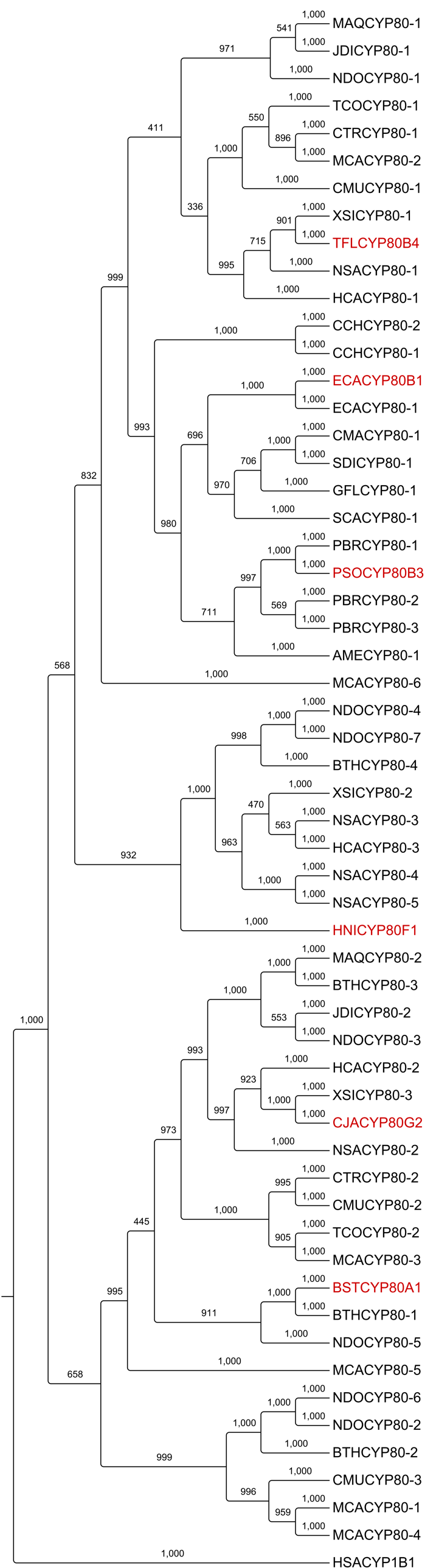

Supplement: Additional file 16: — Phylogenetic analysis of CYP80 gene candidates from twenty BIA-accumulating plant species. Red text denotes characterized genes or enzymes used as tBLASTn queries for transcriptome mining. Black text denotes uncharacterized gene candidates identified through mining (>40 % identity to queries). Bootstrap values for each clade were based on 1000 iterations. Each candidate is labeled with respective species abbreviation (e.g. AME, Argemone mexicana; see Table 1) and candidate number (e.g. CYP80-1). Each query is labeled according to species (additional species: HNI, Hyoscymus niger; CJA, Coptis japonica; BST, Berberis stolonifera; PSO, Papaver somniferum) with CYP80 subfamily and gene number indicated (e.g. CYP80A1, corytuberine synthase; see Fig. 1). The outgroup is CYP1B1 from Homo sapiens (HSA). Amino acid sequences for candidates, queries, and outgroups are found in Additional file 6. (PDF 13027 kb) [file 12870_2015_596_MOESM16_ESM.pdf]

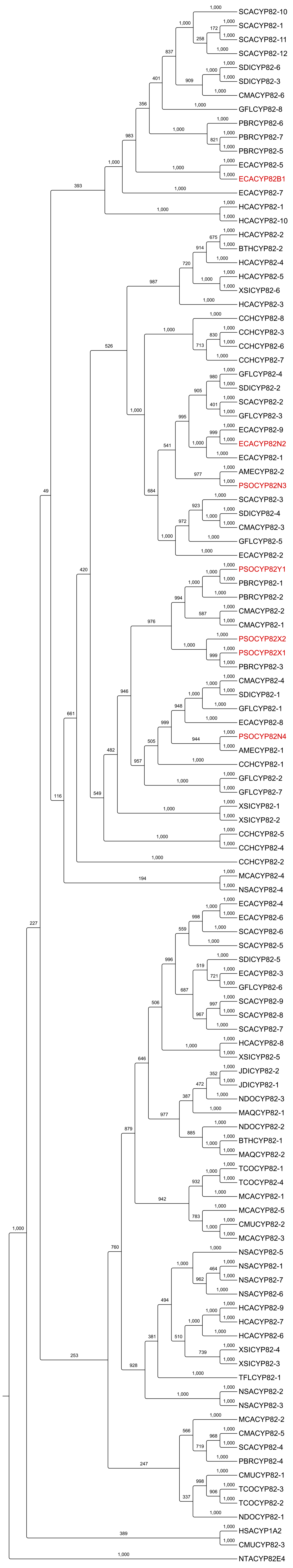

Supplement: Additional file 17: — Phylogenetic analysis of CYP82 gene candidates from twenty BIA-accumulating plant species. Red text denotes characterized genes or enzymes used as tBLASTn queries for transcriptome mining. Black text denotes uncharacterized gene candidates identified through mining (>40 % identity to queries). Bootstrap values for each clade were based on 1000 iterations. Each candidate is labeled with respective species abbreviation (e.g. AME, Argemone mexicana; see Table 1) and candidate number (e.g. CYP82-1). Each query is labeled according to species (additional species: PSO, Papaver somniferum) with CYP82 subfamily and gene number indicated (e.g. CYP82N3, protopine hydroxylase; see Fig. 1). The outgroup is CYP82E4 from Nicotiana tabacum (NTA). Amino acid sequences for candidates, queries, and outgroups are found in Additional file 6. (PDF 7660 kb) [file 12870_2015_596_MOESM17_ESM.pdf]

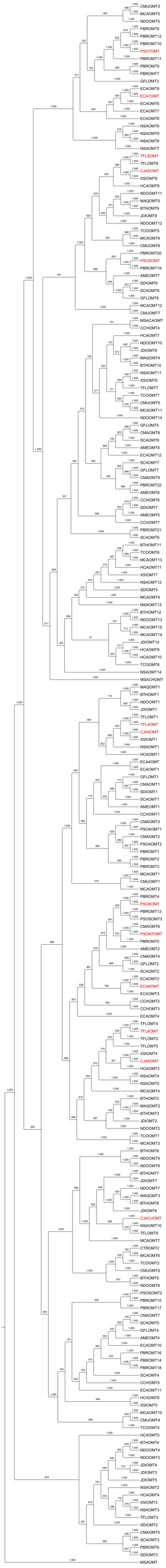

Supplement: Additional file 18: — Phylogenetic analysis of O -methyltransferase (OMT) gene candidates from twenty BIA-accumulating plant species. Red text denotes characterized genes or enzymes used as tBLASTn queries for transcriptome mining. Black text denotes uncharacterized gene candidates identified through mining (>40 % identity to queries). Bootstrap values for each clade were based on 1000 iterations. Each candidate is labeled with respective species abbreviation (e.g. AME, Argemone mexicana; see Table 1) and candidate number (e.g. OMT1). Each query is labeled according to species (additional species: PSO, Papaver somniferum; CJA, Coptis japonica) and specific OMT function (SOMT, scoulerine O-methyltransferase; CbOMT, columbamine O-methyltransferase; N7OMT, norreticuline 7-O-methyltransferase; 7OMT; 6OMT; 4'OMT; see Fig. 1). The outgroup is isoflavone O-methyltransferase from Medicago sativa (MSA). Amino acid sequences for candidates, queries, and outgroups are found in Additional file 6. (PDF 13001 kb) [file 12870_2015_596_MOESM18_ESM.pdf]

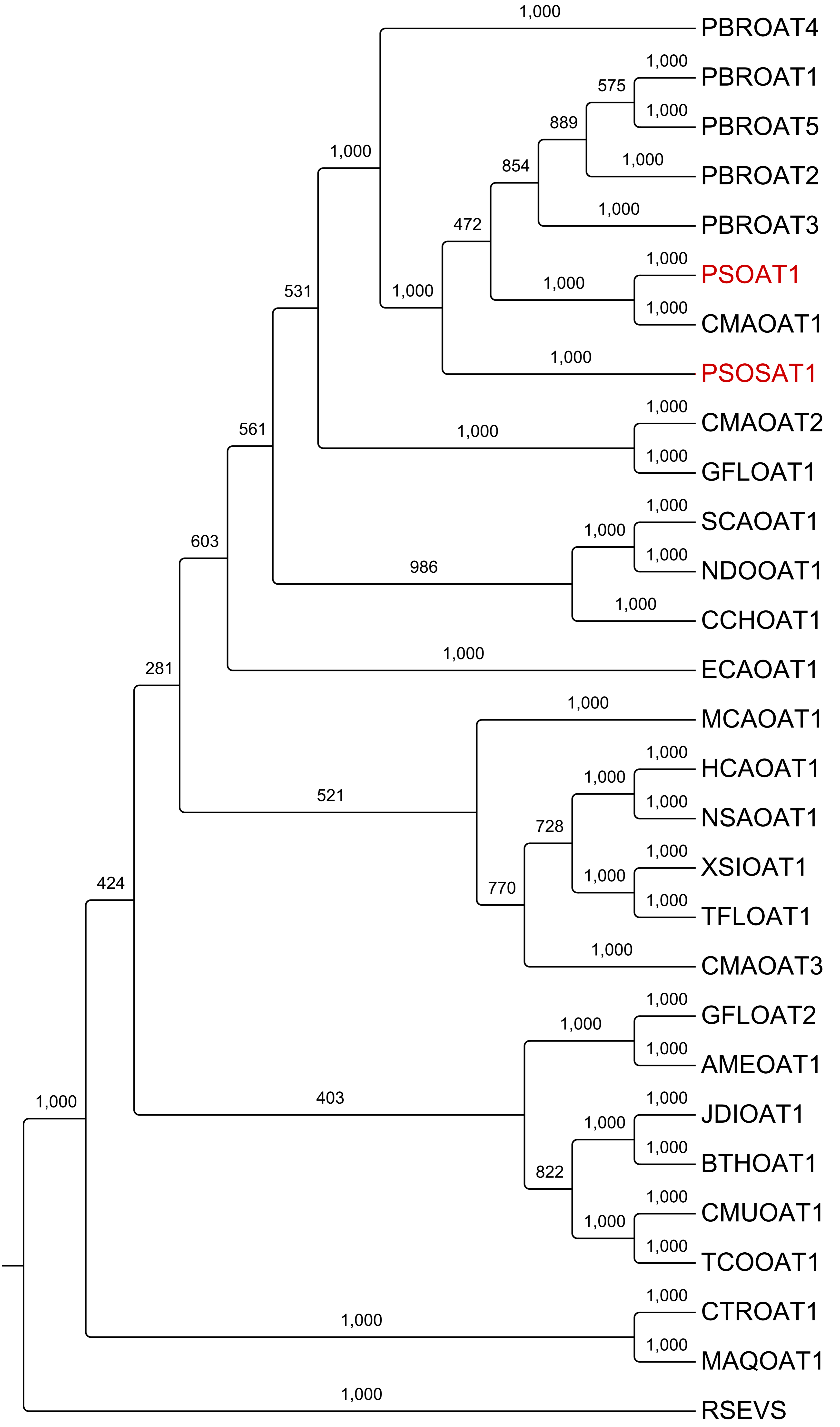

Supplement: Additional file 19: — Phylogenetic analysis of BAHD-type O -acetyltransferase (OAT; D'Aura 2006) gene candidates from twenty BIA-accumulating plant species. Red text denotes the characterized AT1 (1,13-dihydroxy-N-methylcanadine O-acetyltransferase; [6]) and SAT (salutaridine synthase) from Papaver somniferum (PSO) used as tBLASTn queries for transcriptome mining. Black text denotes uncharacterized gene candidates identified through mining (>30 % identity to query). Bootstrap values for each clade were based on 1000 iterations. Each candidate is labeled with respective species abbreviation (e.g. AME, Argemone mexicana; see Table 1) and candidate number (e.g. AT1). The outgroup is vinorine synthase from Rauvolfia serpentina (RSEVS). Amino acid sequences for candidates, queries, and outgroups are found in Additional file 6. (PDF 5574 kb) [file 12870_2015_596_MOESM19_ESM.pdf]

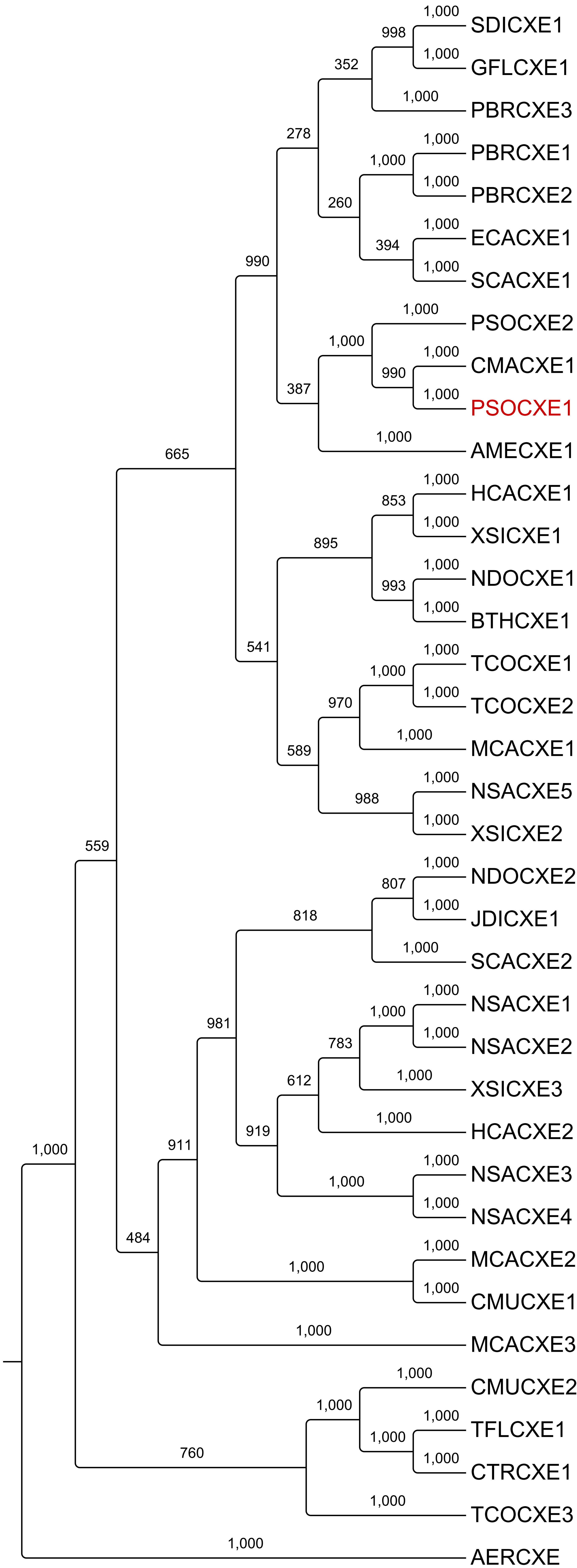

Supplement: Additional file 20: — Phylogenetic analysis of carboxyl esterase (CXE) gene candidates from twenty BIA-accumulating plant species. Red text denotes the characterized CXE1 (3-O-acetylpapaveroxine carboxylesterase; [6]) from Papaver somniferum (PSO) used as a tBLASTn query for transcriptome mining. Black text denotes uncharacterized gene candidates identified through mining (>30 % identity to query). Bootstrap values for each clade were based on 1000 iterations. Each candidate is labeled with respective species abbreviation (e.g. AME, Argemone mexicana; see Table 1) and candidate number (e.g. CXE1). The outgroup is carboxylesterase 1 from Actinidia eriantha (AERCXE). Amino acid sequences for candidates, queries, and outgroups are found in Additional file 6. (PDF 6985 kb) [file 12870_2015_596_MOESM20_ESM.pdf]

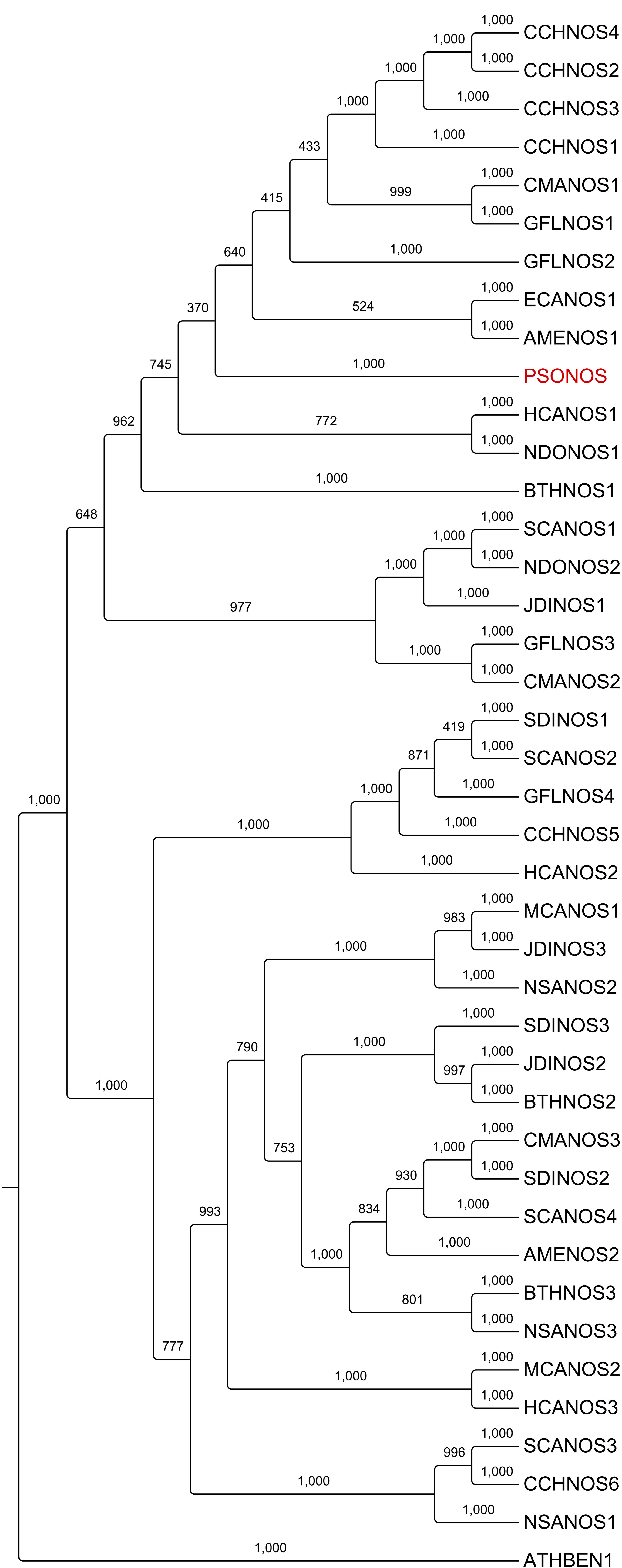

Supplement: Additional file 21: — Phylogenetic analysis of short chain dehydrogenase/reductase gene candidates with homology to noscapine synthase (NOS; [ 6 ]) from 20 BIA-accumulating plant species. Red text denotes the characterized NOS from Papaver somniferum (PSO) used as a tBLASTn query for transcriptome mining. Black text denotes uncharacterized gene candidates identified through mining (>40 % identity to query). Bootstrap values for each clade were based on 1000 iterations. Each candidate is labeled with respective species abbreviation (e.g. AME, Argemone mexicana; see Table 1) and candidate number (e.g. NOS1). The outgroup is ben1-1D (BEN1) from Arabidopsis thaliana. Amino acid sequences for candidates, queries, and outgroups are found in Additional file 6. (PDF 8376 kb) [file 12870_2015_596_MOESM21_ESM.pdf]

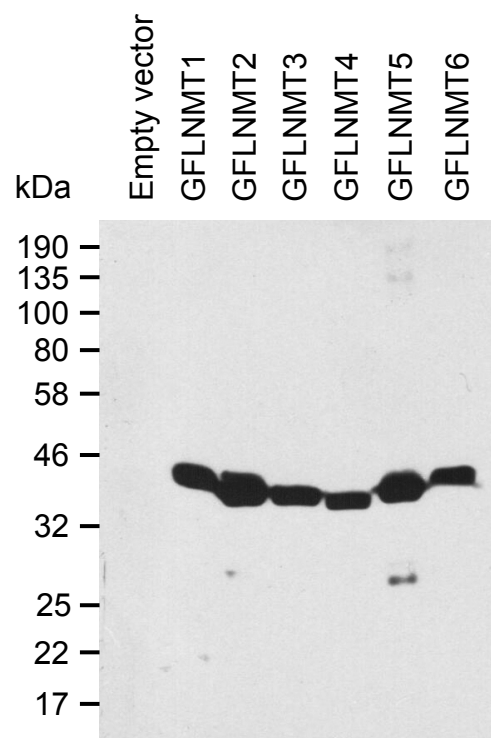

Supplement: Additional file 22: — Immunoblot analysis revealing the presence of His-tagged recombinant protein in soluble extracts of Escherichia coli strains expressing one of six N -methyltransferase candidates (GFLNMT1-6) from Glaucium flavum. (PDF 203 kb) [file 12870_2015_596_MOESM22_ESM.pdf]
