# Supplementary material for: Transcriptome analysis of 20 taxonomically related benzylisoquinoline alkaloid-producing plants
Source: BMC Plant Biol. 2015 Sep 18;15:227. doi: 10.1186/s12870-015-0596-0 (PMC4575454; doi:10.1186/s12870-015-0596-0)
Supplement: Additional file 3: — Summary of results obtained using Roche-based deep sequencing platform. Predicted full-length CDS intersects between Roche-based and Illumina-based transcriptomes are indicated for comparison purposes (see Results and Discussion). (PDF 73 kb) [file 12870_2015_596_MOESM3_ESM.pdf]

No.	Abbrev.	Plant	Tissue	SRA accession number	Number of raw reads	Number of cleaned reads	Average read length (bp)	Average transcript read depth (reads/bp)	Unigenes	Predicted full-length CDS	Intersects between 454 and Illumina predicted full-length CDS
				Roche GS-FLX Titanium							
1	AME	*Argemone mexicana*	Stem	SRX078322	579,575	511,923	406	8.0	25,499	14,446	8,010
2	BTH	*Berberis thunbergii*	Root	SRX202153	728,069	721,524	340	6.2	41,672	12,312	6,129
3	CMA	*Chelidonium majus*	Stem	SRS150402	504,849	408,742	347	7.1	23,678	10,312	8,298
4	CMU	*Cissampelos mucronata*	Callus	SRX130678	721,691	714,978	376	7.3	35,166	14,300	9,877
5	CTR	*Cocculus trilobus*	Callus	SRX130682	535082	525,283	327	5.2	34,783	11,784	127
6	CCH	*Corydalis chelanthifolia*	Root	SRX078320	502,500	431,507	360	7.6	22,511	10,912	8.716
7	ECA	*Eschscholzia californica*	Root	SRS160813	472,167	423,743	428	5.8	32,150	17,385	12,911
8	GFL	*Glaucium flavum*	Root	SRS212395	648,604	540,433	396	8.1	26,520	12,084	8,199
9	HCA	*Hydrastis canadensis*	Rhizome	SRS212407	685,239	440,083	254	6.8	23,809	10,801	7,617
10	JDI	*Jeffersonia diphylla*	Root	SRX202161	833,182	821,875	340	7.4	38,773	13,293	8,880
11	MAQ	*Mahonia aquifolium*	Bark	SRX078960	508,160	442,484	289	5.1	36,429	11,802	495
12	MCA	*Menispermum canadense*	Rhizome	SRX078321	443,738	392,619	378	5.7	36,399	8,858	3,956
13	NDA	*Nandina domestica*	Root	SRX202162	832,375	821,728	372	6.4	45,387	18,367	11,138
14	NSA	*Nigella sativa*	Root	SRX078325	1,326,498	1,203,320	318	9.1	50,508	19,551	10,457
15	PBR	*Papaver bracteatum*	Stem	SRS160814	595,176	528,920	380	4.7	46,224	18,879	14,042
16	SCA	*Sanguinaria canadensis*	Rhizome	SRS212403	653,689	571,822	417	8.7	25,652	11,787	9,514
17	SDI	*Stylophorum diphyllum*	Stem	SRX078312	646,959	576,700	353	5.5	43,568	19,631	9,836
18	TFL	*Thalictrum flavum*	Root	SRX039636	440,689	401,315	385	7.8	21,146	13,002	8,153
19	TCO	*Tinospora cordifolia*	Callus	SRX130689	647,850	641,937	381	6.8	34,518	14352	9,403
20	XSI	*Xanthoriza simplicissima*	Rhizome	SRX078324	978,176	729,767	265	6.3	42,969	15,995	7,107
		**Average**			**664,213**	**592,535**	**356**	**6.8**	**34,368**	**13,993**	**7,708**
