# Supplementary material for: Transcriptome analysis of 20 taxonomically related benzylisoquinoline alkaloid-producing plants
Source: BMC Plant Biol. 2015 Sep 18;15:227. doi: 10.1186/s12870-015-0596-0 (PMC4575454; doi:10.1186/s12870-015-0596-0)
Supplement: Additional file 4: — Summary of results obtained using the Illumina-based deep sequencing platform. (PDF 80 kb) [file 12870_2015_596_MOESM4_ESM.pdf]

				Illumina GA / HiSeq					
**No.**	**Abbrev.**	**Plant**	**Tissue**	**SRA accession number**	**Number of raw reads**	**Number of cleaned reads**	**Average transcript read depth (reads/bp)**	**Unigenes**	**Predicted full-length CDS**
1	AME	*Argemone mexicana*	Stem	SRX096074	79,936,080	50,819,736	39.1	75,101	32,940
2	BTH	*Berberis thunbergii*	Root	SRX096075	70,074,022	64,584,658	53.1	88,302	24976
3	CMA	*Chelidonium majus*	Stem	SRX096034	62,362,800	50,819,736	32.3	45,005	32,416
4	CMU	*Cissampelos mucronata*	Callus	SRX130854	232,997,416	190,922,766	137.1	69,822	21,140
5	CTR	*Cocculus trilobus*	Callus	SRX202432	220,956,972	190,682,284	69.0	84,793	3,773
6	CCH	*Corydalis chelanthifolia*	Root	SRX096066	79,161,120	69073478	38.2	51,797	42,019
7	ECA	*Eschscholzia californica*	Root	SRX096037	62,704,080	53,746,798	37.3	42,167	31,085
8	GFL	*Glaucium flavum*	Root	SRX096058	60,410,640	38,697,818	63.2	31,100	15,861
9	HCA	*Hydrastis canadensis*	Rhizome	SRX096072	71,077,680	61,254,386	87.8	33,335	18,744
10	JDI	*Jeffersonia diphylla*	Root	SRX202488	331915850	235,742,972	187.5	86,832	24,421
11	MAQ	*Mahonia aquifolium*	Bark	SRX202767	231932044	196,915,558	62.2	98,375	13,,866
12	MCA	*Menispermum canadense*	Rhizome	SRX096076	82,491,120	71,509,392	42.7	87,141	32,954
13	NDA	*Nandina domestica*	Root	SRX096070	84,144,000	70,453,764	51.9	70,425	27,337
14	NSA	*Nigella sativa*	Root	SRX096077	76517332	70,223,386	56.3	67,591	22,508
15	PBR	*Papaver bracteatum*	Stem	SRX096061	69721200	57,768,096	36.0	70,428	37,752
16	SCA	*Sanguinaria canadensis*	Rhizome	SRX096071	71,713,920	59,322,808	23.3	53,019	37,241
17	SDI	*Stylophorum diphyllum*	Stem	SRX096064	70,502,640	52,300,928	59.5	50,125	18,970
18	TFL	*Thalictrum flavum*	Root	SRX096062	66,080,640	49,761,080	79.6	41,982	15,426
19	TCO	Tinospora cordifolia	Callus	SRX202768	238,046,752	195,094,516	142.6	81,927	18,967
20	XSI	*Xanthoriza simplicissima*	Rhizome	SRX245855	67,378,080	59,166,962	93.4	48,447	16,436
		**Average**			**116,506,219**	**94,443,056**	**69.6**	**63,886**	**24,442**
