# Supplementary material for: Transcriptome analysis of 20 taxonomically related benzylisoquinoline alkaloid-producing plants
Source: BMC Plant Biol. 2015 Sep 18;15:227. doi: 10.1186/s12870-015-0596-0 (PMC4575454; doi:10.1186/s12870-015-0596-0)
Supplement: Additional file 5: — Summary of results obtained using the Illumina-based deep sequencing platform. For comparison purposes, both stem and root of Chelidonium majus were subjected to transcriptome analysis. (PDF 55 kb) [file 12870_2015_596_MOESM5_ESM.pdf]

No.	Abbrev.	Plant	Tissue	Illumina GA / HiSeq<br>SRA accession number	Number of raw reads	Number of cleaned reads	Average transcript read depth (reads/bp)	Unigenes	Predicted full-length CDS
3	CMA	*Chelidonium majus*	Stem	SRX096034	62,362,786	50,819,736	32.3	45,005	32,416
3a	CMA_RT	*Chelidonium majus*	Root	SRX096036	60,969,004	53,233,416	35.0	24,292	34,955
