# Supplementary material for: Transcriptome analysis of 20 taxonomically related benzylisoquinoline alkaloid-producing plants
Source: BMC Plant Biol. 2015 Sep 18;15:227. doi: 10.1186/s12870-015-0596-0 (PMC4575454; doi:10.1186/s12870-015-0596-0)
Supplement: Additional file 6: — FASTA file of all candidates, queries, and outgroups. (PDF 253 kb) [file 12870_2015_596_MOESM6_ESM.pdf]

COR

>ZMADMAS1
MSATGRAPCGLPRVGLGTAVQGPRPDPVRAAVLRAIQLGYRHFDTAAHYATEAPIGEAAAEAVRTGLVAS
REDLFVTSKVWCADAHRDRVLPALRRTLSNLQMEYVDLYMVHWPVTMKAGRFTAPFTPEDFEPFDMRAVW
EAMEECHRLGLAKAIGVCNFSCKKLETLLSFATIPPVVNQVEINPVWQQRKLREFCRAKGIQLCAYSPLG
AKGTHWGSDSVMDSGVLHEIAKSKGKTVAQVCLRWVYEQGDCLIVKSFDEGRMKENLDIVDWELSEEERQ
RISKIPQRKINQGRRYVSEHGPYKSFEELWAGEI
>PSOCOR
MESNGVPMITLSSGIRMPALGMGTAETMVKGTEREKLAFLKAIEVGYRHFDTAAAYQTEECLGEAIAEAL
QLGLIKSRDELFITSKLWCADAHADLVLPALQNSLRNLKLDYLDLYLIHHPVSLKPGKFVNEIPKDHILP
MDYKSVWAAMEECQTLGFTRAIGVCNFSCKRLQELMETANSPPVVNQVEMSPTLHQKNLREYCKANNIMI
TAHSVLGAVGAAWGTNAVMHSKVLHQIAVARGKSVAQVSMRWVYQQGASLVVKSFNEARMKENLKIFDWE
LTAEDMEKISEIPQSRTSSAAFLLSPTGPFKTEEEFWDEKD
>AMECOR1
MEEVIGLETVVPKVTLSSSSGDNSMPVIGFGTAASPMADHEITKAAVLVGIENGYRHFDTAAAYGTEKAV
GEAIAEALRLGLIKSRAEVFITTKLWCRSCERDLVVPSLKNSLQDLQTDYVDLFLIHWPVRLRHDAGRPP
ISRDQILTFDTKSVWEGMEECHELGLAKNIGVSNFSIKKLEELLATAKIPPVLDQVELSPTWHQKNLIEY
CKEKGIHVTAYSALGANNTHWGNNRVVESDVLGEIAKARGKTTAQIALRWVYEQGVSMVVKSFNQERMKQ
NLQIFDFELTEEESNKISQFPQHKGVSLSAVYGDHDILKEMEAEL
>AMECOR2
MENVIPRVTLSSGSVMPILGMGTASYPFVSSEEGKLAILNAIKVGYRHFDTASTYMFEEFLGEAIAEAIQ
LGLIKSRKELFITSKLWCCDAHPDLVVPSLRNSLRNLKLEYLDLYLIHFPVSLKAGRYEYPPLKEALVLM
DYKSVWAAMEECQNLGLTKSIGVSNFSCKKLQTILDYAKIPPSVNQVEMNPVWQQKKLREFCKANDIVIT
AYSPLGASGTPWGSSRVLDAQVLHEIARAKGKTHAQVCLRWVYEQGVSLLVKTFNEERMKENLRIFDWEL
TEEDKQKISKIPQHRGLTGDAFVSEHEGAPFKTAEELWDGEV
>AMECOR3
MDMNKHAVVYVPTKVIMGIPVIGFGTSADPPVDFDTTRLAIMQAIENGYRHFDTAALYNSEQPLGEAINE
AISRGLIKSRDELFITSKLWCSDTHPQHILPAIQKTLRNLNMEYLDLYLIHWPVSSRHENFEYPIKKEDF
LPMDFKKVWAAMEECHKQGLAKFIGVSNFSCKKLDNIMASATISPSVLQVEVNPCWQQKRLIDFCKANGI
FVVAYAPLGAVGTIYGTNRVMESDVLKQIAKKRGKSVAQVCLRWAYEQGIGVVVKSFNKERMKQNLEIFD
WKLSEEDTKKISEIQQDRACLGMDYTSPYGPYKTIKQLWDEE
>BTHCOR1
MEIVPKVTLNSGYQMPLLGFGTAENWPFTKSEAAKRAILCAIKNGYRHFDTASMYQSEESLGEAIVEALH
LGLIKSRNELFITSKLWCSDCHQDRVLPALRETLKNLHLDYLDLYLIHWPLSAKPGKHEYPILKDKLLPM
DYESVWGAMEECQRLGLSKSIGVSNFSSKKLEQLLKTAKIIPAVNEVEVNPVWHQNKLIEFCKAKGIVVT
GYSPLGAKGTTWGTNKVMDSEVLKEIAKTRGKTHAQVCLRWIYEQGVGLVAKSFNEERMKENLEIFDWAL
TEEESKQISQLPQYKGQNGETFLSAEGPFKTLEELWDEEVAA
>BTHCOR2
MEIVPKVTLNSGYQMPLLGFGTAENWPFTKSEAAKRAILCAIKNGYRHFDTASMYQSEESLGEAIVEALH
LGLIKSRNELFITSKLWCSDCHQDRVLPALKNTLQNLQLDYLDLYLIHFPLSSKRGKLEYPLQEDETYPM
DFGSVWEEMEKCQRVGLTKSIGVSNFSAKKIEHLLTTAKIIPAVDQVEMNPLWQQRTLREYCKTKGIVVT
AYSPLGAKGAIWGSNIVMENEVLAEIAKAKGKTHAQVCLRSVYEKGVALVTRSYNEERMKQNLNIFDWNL
SKEETKRIDNLPQKRVFLGEGFLSPLGPFKSPEELWDGEM
>CCHCOR1
MMVSVPEVTLQSSSSDKKMPIMGMGTAAYPFAESDEAKAALLTSIKLGYRHFDTASLYHNEKSVGKAISE
ALKLGLVKSRDEFYINSKLWCSDSHPDRVIPALQESLKKLGLEYLDMYLIHWPLSLKKGSYEFPIPTDEE
ILPMDFKAVWAAMEECQKLGLTKSIGVSNFSCKKLETILSTAKIPPVVNQVELNTLWQQTKLNKFCKDNG
IVLMAFSPLGAHGSPWGTNRVMECELLHEIAKAKGKTLAQVCLRWIYEQGAGILVKSYNEVRMKENLEIF
DWELSAEELKSISELPQHRGFPADSLLSAKGPFRTEEELWDGEI

>CCHCOR2
MESVACEPGVIEQQQYFIPEVTCGGGSSTTRMRMPVVGMGTASYPFAGSEGVKQALLHAIKLGYWHFDTA
SFYQTEQSLGEAISEAIQLGLIKSRDELFITSKLWCTDAHKDLVLPALQNTLRNLQLEYLDLYLIHFPIS
LKPGRYDFPVQSKEDLLPMDFKSIWAKMEECQGLGLTKSIGVSNFSCKKLENLLATAKIPPAVNQVEMNP
LWQQKKLREFCKANGITITAHSPLGAKGTNWGTNRVVECEVLHQIAKAKGKSHAQVCLRWVYEQGVSLLV
KSFNKERMKENLGIFDWELNAEDLKKISEIPQGRGLPVDGFLSPNGPFKSEEEFWDGEI
>CCHCOR3
MPVVGFGTAKFPFGDDEAIKLAVLEGIKLGYRHFDTATKYKSEKPLGETIVEAINLGLIKSRDELFITSK
LWCGSTERSLIVPSLKTSLQNLQLDYLDLYLIHWPLRFSTDETPTPVPKEQLLPFDTKSVWQAMEECQNL
GLTKSIGVSNFSCKKLEELLSTAKITPAVNQVELNTSWQQTKMREFCKGKGIHITGYSPLGSYGTKWGDN
RVVENQVLEEIGKPRGKTAVQVCLRWIYEQGASMVVKSFNKERMKANLDIFDWKLTEEELKRISQLPQHK
ASLPTASFGDHDEVRELDYEI
>CMACOR1
MESDSAAAAVVVPVTTLSSGIEMPMLGMGTVENFLPGSETVKLALLNAIKLGYRHFDTAAIYQTEECLGE
AVAEALQLGLIKSRDQLFITSKLWCSDAHPDLVIPALQNSLRKLKLEYLDLYLVHWPLSSKPGNCVFPIL
KEDLLPLDFKSVWAAMEECQKLGLTKSIGVSNFSCKKLQDLLTSAIIPPVVNQVEMNPRWQQKKLREFCK
ARDIVVTAYSPLGAKGTVYGSGAVMDCEVLHQIAKIRGKSVAQVSLRWVYQQGVTPLVKSFNEERMKENM
KIFDWELYEEDLKMIDEIPQSRVNPCYFFLSENGPFKTVEEFWDGEV
>CMACOR2
MESSNNAVLVPVITLNSGREMPIVGMGTAENLFQGSERVKLALLTAIKVGYRHFDTAAVYQTEESLGEAI
AEALQLGLIKSRDELFITSKLWLPDCHHDLVLPALQNSLRKLKLEYLDLYLIHWPVSSKPGEIKHVIPKE
ELLPMDFKSVWAAMEECHKLGLAKSIGVSNFSCKKLQDLLVTANIPPDVNQVEMNPLWQQTKLREFCKAH
GILVAAYSPLGAKGTAWGRTNGVMDSEVLQQIAKARGKSIAQVSLRWVYEQGVVLLVKSFNEERMKENLK
IFDWELSGEDLKKISEILPQRRGLPSHVFVSDDGPFKSEEELWDGEV
>CMACOR3
MESSNNAVLVPVITLNSGREMPIVGMGTAENLFQGSERVKLALLTAIKVGYRHFDTAAVYQTEESLGEAI
AEALQLGLIKSRDELFVTSKLWIPDCHHDLVLPALQNSLRKLKLEYLDLYLIHWPVSSKPGELRSLIPKE
ELLPMDFKSVWVAMEECHKLGLTKSIGVSNFSCKKLRDLLLTANIPPAVNQVEMNPLWQQKKLREFCKAN
GIVIAAYSPLGAKGTAWGRTNDGVMDSELLQKIAKARGKSTAQVSLRWGYEQGVILLVKSFNEERMKENL
KTFDWELSGEDLKQINEILPQRRGHSHEFISDNGPFKSEEELWDGEV
>CMACOR4
MGEEMIKTSSVEIPVATLSTGRTMPLLGMGTAAYPFVGSDGVVKAILHAIKLGYRHFDTAALYFTEECLG
DAVAEALRLGLIKSRDELFITSKLYCCDAHPDRVVPALQNSLRKLKMEYLDLYLIHWPASTHEGNFEFPL
QKQDILPLDFKSVWAAMEECQKLGLTKSIGVSNFSCKKLQDLLASAKIPPAVNQVEMNPIWQQNKLREFC
KANDIHITAYSPLGANGTPWGSSGVAETQVLHDIAKARGKTHAQVCLRWVYEQGVSVLVKSYNEERMKEN
LAVLDWELSEENLKKISEIPQRRGLPGDIFVSDNGPFKTVEELWDGEV
>CTRCOR1
MPVVGMGLAAYPFQESEVVKLAMLRAIEMGYRHFDTAALYQTEQSLGLAIAEALELGLIKSRDELFITSK
LWCSDAHPHLVIPALQRTLKNLGLDYVDLYLIHWPVSSNKPGKYDFPVPKEELVQMDYESVWAAMEEGHR
LGLTKSIGVSNFSCKKLENLLTIAKVPPTVNQVEMNPFWRQYKLKEFCKEKGIIITAYSPLGAKGTIWGT
NKVMESKVLEDIGIASGKTLAQVCLRWVYEQGVTLLVKSFNEERMKGNLDIFDWELSEKELKKINEIPQS
RGLPGDIFVSDDGPFKSEAELWDGEV
>ECACOR1
MGSCSIPVKSLSSGREMPVIGMGTADSNLAGSDASRIALVKAIEVGYRHFDTASIYQSEQNLGDAIAQAL
ELGLIKSRDELFITSKLWSSDSHPDRVVPALQESLRKLKLEYLDLYLIHWPASSTPGIYEFPIPKDEIFP
LEYETIWAAMEECQRLGLTKSIGVSNFSSKKLQTILNTATIPPAANQVEMNPVWQQKKLKEFCDANNILL
TAYSPLGAKGTSWGSNKVMESEVLKQIAMANGKSVAQVSLRWLYQIGATMVVKSYNAERMKENLKIFDWE
LSDEDMKAISEIPQRRNFAGDILISSNGPFKSEDELWDGEV
>ECACOR2
MSSYGSVPMKSLNSGSKIHVLGFGTAAYPFVGSDGVKNAILNAIKLGYRHFDTAALYFTEESLGEAVSEA

LQLGLIKSRDELFITSKLWISDAYPDRVLPAIQKTLRNLKMEYLDLYLIHWPISGKEGNFDLPVPKDCLQ
ELDYKPVWAAMEECQKLGLTKSIGVSNFSCKKLQIILSSATIPPAVNQVEMNPIWHQKKLREFCKANNIM
IAAYSPLGAAGTPWGSSGVVETEVLHEIARSRGKTHAQVCLRWVYEQGVIVLVKSFNEERMKENLSVFDW
ELTDEDLKKISQIPQRRGLPSGDIWVSTEGPFRSEEELWDGEI
>GFLCOR1
MEMSSAQVVPLMTLNSGKEIPVLGMGTAENHFQGSDGTKDALVNAIKIGYRHFDTAAIYNVEECLGDAIA
EALQHGLIKSRDELFITSKLWISDSHPDRVLFALQNSLRKLKLEYLDLYLIHWPLSSTPGKSVFPVPKED
FLPLDFKSVWAAMEECQKLGLTKSIGVSNFSCKKLQDLLDIANIPPAVNQVEMSPLWQQKKLREFCKVNG
ILVTAYSPLGAKGTFWGSNEIMDSHVLQEIAKARGKSVAQVTLRWVYEQGVILLVKSFNENRMKENMAIF
DWDLSIDDLKKFDEISQRRGNLGDFFVSENGPFKSVDMVWDGEV
>GFLCOR2
MESSSSSAVVVPVMTLNSGKEMPVLGMGTAENNLQGSEKTKLALLTAIKAGYRHFDTASAYNTEEALGEA
VAEALQLGLIKSRDELFITSKLWCSDAHPGLVVPALQNSLRNLKLEYLDLYLIHWPVSLKPGKFVIPFSK
EDILPLDFKNVWAAMEECQKLGLAKSIGVSNFSCKKLQDLLAIANIPPAVNQVEMSPLWQQKKLREFCKV
NGILVTAYSPLGAKGTFWGSNEIMDSDVLNQIAKARGKSVAQVSLRWVHEQGVILLVKSFNENRMKENMA
IFDWELSNDDLKKMDEISQRRGPLADIFVSENGPFKSVDMLWDGGF
>GFLCOR3
MGSSDAVVVPVKTLSSGRKMPVIGMGTAEILLVDGSEKVKLALLNAIKIGYRHFDTAAVYRTEEALGEVV
AEAIQLGLIKSRDELFITSKLWLSDSHPDLVLPALQNSLRKLKFEYLDLYLIHWPLSSKPGELKVLIPKE
ELLPLDFKSVWAAMEECQKLGLTKSIGVSNFSCKKLQDLLDIANIPPAVNQVEMNPLWQQKKLNEFCKAN
DIVITAYSPLGAKGTPWGSNRVMDSEVLNQIAKARGKSVAQVALRWVYEQGVSLVVKSFNEERMKENVTK
IFDWELSAEDLKKIEEIPQCRGVPSHVFVSVNGPFKSEEELWDGEI
>GFLCOR4
MGEEMKIKSVEIPVVTLSSGRTMPVFGMGTAAFPFVGSDGVVKAITHAIKLGYRHFDTAALYFTEESLGD
AIAEALRLGLIKSRDELFITSKLWCCDAHPDRVLPALRNSLRNLKIEYLDLYLIHWPASTHEGNLEFPIQ
KQDIHPLDYNSVWAAMEECQTLGLTKSIGVSNFSCKKLQHILAIAKIPPAVNQVEMNPIWQQKKLREFCK
ANDIHITAYSPLGANGTPWGSSGVVETQVLHDIAKARGKTHAQVCLRWVYEQGVIVLVKSYNEERMKENL
TVFDWELSAEDSMKISEIPQHRGLPGDIFVSDSGPFKSEEELWDGEV
>HCACOR1
MGSVPNVILSSGHPMPLVGFGTAGFPFGTSEGIKSAILCGIKNGYRHFDTASVYQTEQILGEAIAEALEL
GLIKSRDELFLTSKLWCSDAHQQHVLPALQKTLRTLQLDYLDLYLVHWPLSSKPGKYEYPIPKEELLPMD
FKSVWAAMEECQALGLTKSIGVSNFSCKKLEQLLSTSNIPPAVNQVEVNPIWQQNKLREFCKAKGIIVAA
FSPLGAKGTSWGTNKVMDSEVLNEIAQARGKTTAQNCLRWLHEQGVCVVVKSFNEERMKGNLKLFDWELS
KEESKKISQLPQSKGHTGDDMVSANGPFKSLEELWDGEI
>HCACOR2
MTRIPEIVLNSGWRMPVLGMGTATFPIQSPEVIESSIVNAIELGYRHFDTASVYQSESPLGRAISEAIRR
GLIESRKEVFITSKLWCTDAHHDLVIPALHKTLQNLGLEYLDLYLIHFPVRLKGDISFDIKKADLIPFDV
KGTWEAMEKCQELGLTRSIGVSNFSSKKLSELLTHATISPAVNQVEMHPFWQQKELRAFCADKGIHVSAY
SPLGGKGALWGSDILLNSKEIERIAQAKGKSIAQVCLRWAYEQGVSYLPKSYNKGRMKENMEIFDWQLSE
DELQKISHLPQGKIYTGHHFISDDGEYKSPIDLWDCEIC
>HCACOR3
MGIVREVVLNSGERMPLLGMGTATYPVAPFELVESSVIAAIELGYRHFDTASVYETEQPLGLAISEAVRQ
GLIASRDEIFITTKLWCGHAHYDLVLPALRDSLETMGLDYVDLYLIHFPARFNMKEKSLNVNKKDLLPLD
IRGTWKAMEECYELGLAKSIGVSNFSCKKLSQLLSVANIPPAVNQVEMHPLWQQQKLREFCEEKGIHVSA
YSPLGGKGTIWGSNAVLDSDQIKQIAKAKGKSIAQICLRWGFEHGVSILPKSFNKERLKENMEIFDWELS
KEELQKMNTFPQNRIFQAEHLVSPDEGLFKSVADLWDGEI
>JDICOR1
MCFCVLVTKRAMGIVPEVTLNSGHQMPLVGFGTAQFPFPEGDEAKQIIAPILCGIKSGYRHFDTASLYKT
EEALGEAIKEALRLGLIKSRDELFITSKLWCTDSHQDLVLPALKKTLQNLQMDYLDLYLVHFPVSSKPGK
PEFPPQKEDLLPMDFGSVWAAMEECQRLGLTKSIGVSNFSCKKLEQLLTTAKIIPAVNEVEMSPVCQQNK

LREFCKVKNIVVTAYSPLGGGSNAVKDNKVLKEIAKAKGKTCAQVTLRWVYEQGVALVPKSFNEGRMKEN
LDIFNWTISEEEFKQISQLPQGRVATGEWFVSVEGPFKSLEELWDEVI
>MAQCOR1
MGVVPVVTLNSGHEMPLVGFGTAVPAFGGSDAIKQAILCGIKNGYRHFDTASVYQTEKSLGEAIVEALRL
GLIKSRDELFITSKLWCTDAHQDGVLPALRETLKNLQLDYLDLYLIHWPLSAKPGKHEYQIPKDELLPMD
YESVWGAMEECQRLDLSKSIGVSNFSIKKLEELLKTAKIIPAVNEVEVNPVWHQNKLIEFCKAKGIVVTG
YSPLGAKGTTWGTNRVMDNEVLKEIAKTRGKTHAQVCLRWIYEQGVGLVAKSFNEERMKENLEIFDWALT
EEESKQISQLPQYKGQNGETFLSAEGPFKTLEELWDEEIAA
>MCACOR1
MASVPEVTLSSGHPMPLFGFGTAAWPFVSSEGAKSAILCGMELGYRHFDTASIYGTESSLGEAIAEALKL
GIIKSRDELFITSKLWCCDGHHDRVLPALQMTLKNLQLDYIDLYLEHWPLSSVPKGELEYPIPKEKLFPM
DLKAVWEAMEECQRLGLTKSIGVSNFSCKKLEDLLAFAKIPPAVNEVEMNPVWQQKKLRDFCKAKGIVLT
AYSVLGAKGTLWGTNKVMDSEVLGKIAKARGKTVGQVCLRWAYEQGACVLVKSFNEERMKENLETFNWEL
GEEDLKMISEIPQYKGLRGEDMVAPNGPYKTLEELWDGEI
>MCACOR2
MGVDKTNEFSNGNAASPVLIKEANLFKIAEVTLNSGHHMPVLGTGTASFPFPPLKELKKAIVEAMEVGYR
HFDSAALYQSEEGLGAAIAEALEKGLVKKREELFITTKLWCNNAHSDRVLPAIRESLRKLRLDYVDLYLI
HFPVRLKEDLLDMNCKKGGIFELDLKSVWAAMEQIQHLGLAKSIGVSNFTCKKLTDLLSYAKIPPAVNQV
EMHPVWQQKKLRDFCKEKGIHVSAYSPLGAKQWGFDVVLGNKILKEIAQDKGKTIAQIALRWGYEQGVIL
IPKSFNKGRLTENLRIFDWKLTEDELKKISSIQQSRVAIMPEFVFPESPFKTFEDFWDGEM
>MCACOR3
MEGGGVPKILLNSGHRMPVLGMGTASFPISPQHLVKSSILTAIELGYTHFDTASVYETEPTLSRAIRQAL
EGRHIASRDQLFITTKLWCGQAQSDLVVPALRESLQELGLDYVDLYLIHFPARFKCVEKTFNVTKEDLLP
LDMKGTWQAMEECCKLGLAKSIGVSNFSCKKLSQILTFATIPPAVNQVEMHPLWQQRKLREFCKEKGIHV
SAYSPLGGKGAKWGSNAVFECEEIKQIAEAKGKSLAQICLRWAFEQGVSFVPKSFNKERLKENMEIYGWE
LSKEELQKLSVLPQCRIFKGEWLVSADHGVFKSVEDLWDGEI
>NDOCOR1
MVSVPEVTLNSGHRMPLVGMGTACFPLPGSEVVKSAMVTAIKLGYRHFDTAALYQSEQPLGEAIAEALHL
GLIISRDELFITSKLWCTDAHQDLVLPALKTSLRNLQLDYLDLYLIHFPVSLKPGKIQLPVPKEELLPMD
FKSVWAAMEECQKLGLTKSIGVSNFSCKKLEHILSLAKIPPAVNQVELNPIWRQKKLIEFCKAKGIVVTA
YSPLGAVAPGQGSNRVMECEVLSEIAKARGKTHAQVCLRWIIEQGVSLVVKSFNEDRKKKNLEIFDWELS
EEDSKKISQIPETRGNPGDFVISVDGPYKSREELWDGEI
>NDOCOR2
MGRVPETTLSSGHNMPLLGMGTATFPLPPSELIESYILAAIEMGYRHFDTASVYDTEVPLGRAISEALRR
GLVANRDELFITTKLWCGHAHPDLVVPALHQSLMEMGLEYVDLYLIHFPARFNKKEKNFDVKKEEVIPLD
MKGTWEAMEECSKLGLAKSIGVSNFSCKKLSQLLTYATIPPAVNQVEMHPLWQQWKLREFCVANDIHVTA
YSPLGGKGALWGSNSVLDSKEIQQMAEAKGKSIAQICLRWAFEQGVSFIPKSFNVERLKENMEIFDWEMN
KNELLKMSLLPQKRTFTAEYLVSPDGAFKSVDDLWDGEI
>NSACOR1
MARVPEITLNSGHRMPLLGMGTAVVPFKEPEPVKSAILTAIKNGYRHFDTASLYRTEPALGDAIAEALQL
GLINSRDDLFITSKLWCTDNHPDLVLPALHTTLRNLKLDYLDLYLIHWPVSMTPGRIRFPGPDEKILPMD
YKSVWAAMEECQKLGLTKSIGVSNFTCKKLELLLASATIPPAVNQVEVNPMWQQEKLIEFCRTKGIVVTA
YAPLGTVGTFIGNNDIMNCELLKEIAQAKGKTNGQICLRWVHEQGVGLLVKSFTEVRMKQNLEIFDWELS
EEESMKIKQLPQRKSHQGKGFVTADGPFKSLTELWDGEM
>NSACOR2
MVYEIMLNSGYRIPLLGMGTAAYPVPPPELVESSVLAAIELGYRHFDTAHIYGTEAPLGRAISEALRKGL
IKSRDELFITTKLWPGHARPDLVLQALHQSLEALRIDYVDLFLIHFPARYNTTEIRMDIKKEDILPLDIK
GTWQAMEECCKLGLAKSIGVSNFGCKKLSQLLDSATIPPAVNQVEMHPLWQQAKLREFCAERGIHVSAYS
PLGGKGTLWGSNAVLDSEQIKQIALAKGKSVAQICLRWGFQQGVSIIAKSFNRERLKENMEIFDWELNKE
ELYKLSILPQNRISTLEYLVSPDAVFKSINDLWDEEV

>NSACOR3
MDNSVVVLNSGHRMPLLGMGTAATPLPPNEVVQSSVLAAIELGYRHFDTASVYGSEVILGRAISEALRRG
LVASRDELFITTKLWCGNGHPDLVLGALQESLRIMELDYVDLYLIHFPVRFNITEKLLNMKNVPLLPFDM
EGTWQAMEECCRLGLAKSIGVSNFSCKRLSELLLSASIPPAVNQVEMHPLWQQRNMREFCRKEGIHVSAY
SPLGGKGAAWGSNAVLDSEDIQQIAQHKEKSVAQVCLRWAFEQGVSIIVKSFNKERLKENTQIFDWELNK
QELQRINTLPQNKIFKASNLISADGPYKTFEELWDGEV
>PBRCOR1
MESNGVPMITLSSGIRMPALGMGTVETMEKGTEREKLAFLKAIEVGYRHFDAAAAYQTEECLGEAIAEAL
QLGLIKSREELFITSKLWCTDAHADLVLPALQNSLRNLKLEYLDLYLIHFPVSLKPGKIVSDIPKDQMLP
MDYKSVWAAMEECQTLGFTRAIGVSNFSCKKLQELMATANSPPVVNEVEMSPIFQQKKLRAYCKANNIMI
TAYSVLGARGAAWGSNAVMDSKVLHEIAVSRGKSVAQVSMRWVYQQGACLVVKSFNEERMKENLKIFDWE
LSAEDMEKISEIPQSRTSSADFLLSPTGPFKTEEEFWDEKD
>PBRCOR2
MEINGVPVISLSSGVRMPALGMGTAETMEKGTDRERSAFLKAIEVGYRHFDTAAAYQTEECLGEAIAEAL
QLGLIKSRDELFITSKLWCTDAHADLVLPALQNSLRNLKLEYLDLYLIHAPVSLKPGKILNEIPKDQMLP
MDYKSVWAAMEECQTLGFTRAIGVSNFSCKKLQELMATANSPPVVNEVEMSPTLHQKNLREYCKANNIMI
IAYSVLGARGTGWGSNAVMDSKVLHQIAVARGKSVAQVSMRWVYQQGACLVVKSFNEERMKENLKIFDWE
LTEEDMDKISEIPQSRTLSADFLLSPTGPFKTEEEFWDEMV
>PBRCOR3
MRNTGVPVITLSSGKGIPVLGMGTFETVGKGGERERLAFLKAIEVGYRHFDTAACYQTEECLGEAIEEAL
QLGLIKSRDELFITSKLWCTDAHPDRVLLALQNSLRNLKLEYLDLYLIHFPVSLKPGNEVTMDAAGGEIF
LMDYKSVWAAMEECQNLGFTKSIGVSNFSCKKLQELLATANIPPVVNQVEMSPVFHQKNLREYCKANNIL
VAAYSILGGKGTAWGSNSVLGSEGLNQIAIARGKSIAQVSMRWVYEQGAILVVKSFSEKNMRENLNIFDW
ELTKEDLERIGEIPQRRLIIQEFMVSSNGPFKSLEEFWDEKAD
>PBRCOR4
MENVIPAVTLSSGSVMPILGMGTAAYPLVEPEEAKLAFLNAIKIGYRHFDTAASYHCEGFLGEAIAEALQ
LGLIKSRDELFITSKLWPCDAHPDLVIPAIQNSLRNLKLEYLDLYLIHFPISTKPAAGLVFPPPKDALLP
MDYKSVWAAMEECQKLGLTKSIGVSNFSCKKLQTILDIANIPPAVNQVEMNPVWQNLKLRDFCKANNIVL
TAYSPLGARGTPWGSNAVYEERVLHEIAEAKGKTHAQVCLRWVYEQGVSLIVKSFNELRMKENMMIFDWE
LTEDELQKIGKIPQRRGLPGDFFVSEAAPFKTVEEFWDGEI
>PBRCOR5
MEVVIPKVALSSGRVMPVLGMGTSSFPPVGPEDGKAAILNAIKIGYRHFDTASVYKSEDFLGEAIAEALL
LGLIQSRDQLFITSKLYCNDAHPDLVVPALQNSLRNLRLEYLDLYLIHFPVSSKPVKYEYHLKKEHLLPM
DYESVWAAMEKCQKLGLTKSIGVSNFSCKKLQTILDTANISPAVNQVEMNPVWQNLKLRDFCKAKGIVVT
AYSPLGASGTPWGSNAVKEAQELHEIAKARGKTHAQVCLRWVYEQGVSLLVKSFKEERMKENLMIFDWEL
TKDDLFKISKITQRRGLPGYRFISKLEGSPFKTVEEFWDGEV
>PBRCOR6
MESSGVPVITLRSGKVMPVLGMGTFEKAGKGSERERLAILKAIEVGYRYFDTAAAYETEEVLGEAIAEAL
QLGLIKSRDELFISSMLWCTDAHPDRVLLALQNSLRNLKLEYVDLYMLPFPASLKPGKITMDIPEEDICP
MDYRSVWSAMEECQNLGLTKSIGVSNFSCKKLEELMATANIPPAVNQVEMSPAFQQKKLREYCNANNILV
SAVSILGSNGTPWGSNAVLGSEVLKKIAMAKGKSVAQVSMRWVYEQGASLVVKSFSEERLRENLNIFDWQ
LTKEDNEKIGEIPQCRILSAYFLVSPKGPFKSQEELWDDKA
>PBRCOR7
MGSSCIPVLTLNSGNKMPVLGMGTFETFAKGGERERLAYLKAIKVGYRYFDTGAAYGTEEVLGQAIAEAL
QLGLIKSRDELFISTMIWATDAHPDGVLPAVQRSLRNLKLDYVDLYLIPFPASLNPEGEIPNYIPENETF
LKMDYKSVWAAMEECQTLGFTKSIGVSNFSCKKLQEVMETANIPPAVNMVEMNPTFQQKYLREYCKANNI
LVNAYSVLGSTGTSWGSNAVMGSEVLKQIATDIGKSIAQVSMRWVYEQGAGFVVKSFSEERMRENLNIFD
WELTKEDLEKISQIPQCRVLPMDFLVSSDGAFKSLEDLWDGEA
>PBRCOR8
MPILGFGTAENLFEGGDKVKLAILKAIEVGYRYIDTAAVYRTEESVGEAVAEALQLGLIKSRDELFITSK

LWGVDAHPDLVLPALQNSLRKLKLEYLDLYLIHYPVSLKPGEMVDDIPKDEIFPLDYKSVWAAMEECQKL
GYTKSIGVSNFSCKKLQQLMATANIPPAVNQVEMNPTWQQKNLREYCKANNIFITAYSTLGAKDLLWGSN
AVLGSKVLNQIAVARGKSVAQVSLRWVYEQGVSLVVKSFNEERMKENLKIFDWELTTEDLKMISEIPQRR
VATADFFVSDIGPFKSLEELWDE
>PBRCOR9
MPVLGLGTAENLTKGSEREMLAILKAIEVGYRHFDTAFIYQTQECVGEAIAEALQLGIIKSRDELFITSK
LWGSDAHPDCVLLALQNSLRNLKLQYLDLYLIHYPVSLKPGTTLKDLGNKDNFLPMDYKSVWAAMEECQK
LGLTKSIGVSNFSSKKIQELMSTDSIPPAVNQVEMNPTWQQKKLREYCQANNILVTAYSPLGAKGTTWGS
NAVMGSEVLNQIALARGKSVAQISLRWVYEQGVSLVVKSFNEERMRENLKIFDWELTAEDLKKIDELPQS
RVATAEFVVSENGPFKSLEEFWDDES
>PBRCOR10
MENAIPAVTLNSGSVMPVLGMGTAAYPFVESEEAKLAILNAIKTGYRHLDTAALYQSEESLGEAVSEAIQ
LGLIKSRDELFITSKLWPCDAHPDLVIPAIQNSLRNLKLEYLDLYLIHFPISTKPAAGLVFPPPKDALLP
MDYKSVWAAMEECQKLGLTKSIGVSNFSCKKLQTILDIANIPPAVNQVEMNPVWQNLQLRDFCKDKSIIL
TAYSPLEGKGTPWGSNAVYGAQVLHEIAEAKGKTHAQVCLRWVYEQGVSLLVKSFNDQRMKENMMIFDWE
LTEDELEKISRIPQRRGLPGDFFVSEAAPFKTVEEFWDGEI
>PBRCOR11
MENAIPAVTLNSGSVMPVLGMGTAAYPFVESEEAKLAILNAIKTGYRHLDTAALYQSEESLGEAVAEAIQ
LGLIKSRDELFITSKLWPCDAHPDRVVPTLQNSLRKLKLEYLDLYLIHWPVSSNPVAGHVLSLPKDSLVT
MDYESVWAAMEECQKLGLTKSIGVSNFSCKKLQTILDIANIPPAVNQVEMNPVWQNLKLRDFCKANNIVL
TAYSPLGARGTPWGSNAVYEERVLHEIAEAKGKTHAQVCLRWVYEQGVSLIVKSFNELRMKENMMIFDWE
LTEDELQKIGKIPQRRGLPGDFFVSEAAPFKTVEEFWDGEI
>PBRCOR12
MENVIPAVTLSSGSVMPILAMGTAAYPLVEPEEAKLAFLNAIKIGYRHFDTAASYHCEGFLGEAIAEALQ
LGLIKSRDELFITSKLWPCDAHPDLVIPAIQNSLRNLKLEYLDLYLIHFPISTKPAAGLVFPPPKDALLP
MDYKSVWAAMEECQKLGLTKSIGVSNFSCKKLQTILDIANIPPAVNQVEMNPVWQNLQLRDFCKDKSIIL
TAYSPLEGKGTPWGSNAVYGAQVLHEIAEAKGKTHAQVCLRWVYEQGVSLLVKSFNDQRMKENMMIFDWE
LTEDELEKIGKIPQSRGLPGDVFVSELEAAPFKTVEEFWDGEV
>SCACOR1
MENVSVVTLNSGREMPILGMGTAAYPSVGSEEAKLAILLAIKVGYRHFDTAAAYQIESSLGEAVAEALQL
GLLKSRDELFITSKLWCSDSHPERVLPALQNSLRNLKLEYLDLYLIHWPVSLKPGNFDLTIPKEDLLPMD
YKSVWAAMEECQKLGLTKSIGVSNFSCKKLQDLLATANIPPAVNQVEMNPIWQQKKLREFCKANGILITA
YSPLGANGTPWGSSGVANTEVLHQIAKARGKTHAQVCLRWVYEQGVSLLVKSFNEERMKENLKIFDWELT
AEDLKQISEIPQHRGLPADIFVSVNGPFKTVEEFWDGEV
>SCACOR2
MEISVPITTLSSGREMPILGMGTAENLFNGSEKVKLAILDAIKVGYRHFDTAAVYQTESSLGEAVAEALQ
HGLLKSRDELFITSKLWCSDSHPDRVLPALQTSLRKLKLEYLDLYLIHWPLSSKPGSHDYPIPKEDLLPL
DYKSVWAAMEECQKLGLTKSIGVSNFSCKKLQDLLDTANIPPAVNQVEMNPLWQQKKLLEFCKGNGIIIT
AFSPLGAKGTSWGATNGVMDSEVLHQIAQARGKSIAQVSLRWLYEQGVSLVVKSFNVERMKENLKIFDWE
LSAEDLKKINEIPQRRGLPSGSFISANGPFKSEEELWDGEV
>SCACOR3
MAMETVPKLPLSSGDNSIPVIGFGTAAFPLPDDETLKSAFLNGIEAGYRHFDTAAAYGSEKALGEAITEA
LRLGLLKSREEVFITTKLWCSSCERSLVVPSLKNSLRNLQMEYVDLFLIHWPVRLSIDAQRPPIPREQIL
TFDTKSVWEGMEECHELGLAKNIGLSNFYPKKIDELLATAKIPPAVLQVELSPTWQQKNLIEYCREKGIL
VTAYSALGANGTHWGDNRVVESDVLGDIAKARGKTTAQIALRWVYEQGVCMVVKSFNKERMKLNLQIFDF
ELTEEESNKISQLPQIKGVKLSPMFGNHDVLKELEDQL
>SCACOR4
MGEEMKCGRVVPVATLNSGRTMPLLGMGTAAYPFVGSEKVKSAILHAIKLGYRHFDTAALYQTEECLGEV
VAEALQLGLLKSRDELFITSKLWCSDSHPDRVLPALQTSLRKLKLEYLDLYLIHWPLSLKPGNYGFPIPK
EDMLPMDYKSVWAAMEECQKLGLTKSIGVSNFSCKKLQDLLATANIPPAVNQVEMNPIWQQKKLREFCEA

NGILITAYSPLGANGTPWGSSGVAQTQVLHDIAKARGKTHAQVCVRWVYEQGVSVLVKSYNEERMKENLT
VFDWELDVEDLKKISTEIPQRRGLPSDIFVSVLTGPFKSAEELWDGEL
>SDOCOR1
MESDSTAVAVPVTTLSSGIEMPMLGLGTADEKLLPSSETVKLAFLTAIKLGYRHFDTAAVYQTEECLGEA
VAEALQLGLIKSRDELFITSKLWCSDAHPDLVIPALQNSLRKLKLEYLDLYLIHWPLSSKPGNCVFPIPK
EDLLPLDFKSVWAAMEECQKLGLTKSIGVSNFSCKKLQDLLAMAIIPPAVNQVEMNPHWQQKKLREFCKV
KDIVVTAYSPLGAKGTPWGSNAVMDSKVLHQIAKARGKSVAQVSLRWVYQQGVVLLVKSFNEERMKENMK
IFNWELNEEDLKMIDEIPQCRGNPSHYYVSENGPFKTVEEFWDGEV
>SDOCOR2
MPIVGMGTAENLFEGSERVKLALLTAIKVGYRHFDTAAVYQTEGSLGEAIAEALQLGLIKSRDELFITSK
LWIHDCHHDLVLPALQNSLRKLKLEYLDLYLIHWPVSSKPGELRSLIPKEELLPMDFKSVWVAMEECHKI
GLTKSIGVSNFSCKKLQDLLLTANIPPAVNQKVEMNPLWQQKKLREFCKANGIVIAAYSPLGAKGTLWGT
NGVMDSEVLQQIAKARRKSIAQVSLRWVYEQGVVLLVKSFNEGRMKENLKIFDWELSREDLKQINEILPQ
RRGLPSHVFVSDDGPFKSEEELWDGEV
>SDOCOR3
MTMEAVPKVTLSTSGKSMPIIGFGTALFPTGNDEAVKSAVLSGIEAGYRHFDTAVAYRSEKGLGEGIAEA
LRLGLILSRDEVFITTKLWCTSCERSLVVPSLKNSLRNLQMEYVDLFLIHWPLRLSADAQRPPIRNDQIL
HFDTKSVWEGMEECYELGLAKNIGVSNFYPKKIDELLATAKIPPAINQVELNPAWNQKDLIKYCKSKGIL
ITAYSALGANGTHWGDNRVVDSDVLKDIAKARGKSTAQVALRWVYEQGVSMVVKSFNKERMKQNLQIFDF
ELTEEESNKISQLPQHKGVKLTPVYGDHDAMKKIDNEI
>TCOCOR1
MVSVPEVVLSNGHLMPVVGMGMAAYPFQESETVKQAMIRAIKMGYRHFDTAALYQTEKSLGDAIVEALKL
GLIKSRDELFITSKLWCSDAHPHHVIPALQRTLRNLGLEYLDLYLIHWPVSSEPGKYEFPVRKEELILMD
FENVWADMEEGYRLGLTKSIGVSNFSCKKLESLLTMAKIPPAVNQVEMNPLWRQNKLLRFCKDKGIAITA
YSPLGAKGTIWGTNRVMESEVLNDTANAREKTLAQVCLRWVYEQEVSILVKSFNEERMKENLDIFDWKLS
DEDLEKINEIPQCRGLPGDIFVSDDGPFKSEAELWDGEI
>TCOCOR2
MVGVNKENVVSSVATVPVVTLNSGHKMPVLGTGTASFPVPPLEELKKVIMEAMEVGYRHFDTAAMYQSEE
GLGAAIKEALEKGLIKSRDELFITTKLWCNNAQPHLVLPAIRDSLRRLRLEYVDLYLIHYPVRLKEDLLS
MDCKEDEIFPIDIKSVWSAMEEIHNLGLAKSIGVSNFTCKKLTDLLAHAKIPPAVNQVELHPAWQQKKLR
EFCQEKGIQVSAYSPLGAKQWGFDVVLSNKIIKEIAHHKGKTVAQIALRWGHEQGIILIPKSFNKERLIQ
NLLIFDWELTQDELKKMASIQQSRIAIAPEFVFPGSPFKSFEEFWDGEM
>TCOCOR3
MGISMPDATLNSGHRIPLIGFGTASFPFPLEGSETATRAILEAIKIGYRHFDTAALYQTEVGLGEAIEEA
LHLGLIESRDELFITSKLWCTHGRPERVLPAIRESLKNLKLDYLDLYLIHMPMSFKSEKPWFPLPGEFEA
MDFKSVWEQMEACQRLGLARSIGVSNFSCKKLQLILTTANIPPAVNQVEMNALWQQNKLREFCKSEGIVI
TAYSPLGGKGTSWGSNRVMECEVIKEIATEKGKTHAQVCLRWLYEQGVSMVVKSFNKERMKENLEIFDWE
LTSKECEQIKKLPQSRGFTAQGLVSEDGPFKSIEDIWDGEI
>TFLCOR1
MRRPHSRLASTRDMSSIVPDVVLSSGHKMPLIGFGTVAYPIAASDSIKTAIVNGIKHGYRHFDTASVYQT
EQLLGEAIAEALQFGLIKSRQELFITSKLWCSDSHHDRVLPALQNTLKTLQLDYLDLYLVHWPISSKPGK
HEFPIPKEELLPLHFESVWTAMEECQAVGLTKSIGVSNFSSKKLEQLLSTSKIPPAVNQVEMNPIWQQYK
LREFCKSKGIVITAFSPLGAKGTSWGTNKVMDSEVLNEIAQAKGKTHAQVCLRWLHEQGICVVVKSFNEE
RMKENLEIFDWELSPEESALIQQLPQSRGNTGEDFISVDGPFKSLEELWDGEI
>TFLCOR2
MGSIPEVSLNSGHRMPLIGMGTASYPFAGSEVVKSAILSAIKLGNRHFDTAALYKTEQIIGEAIAEALQL
GLIKSREELFITTKLWVNDGHKDCILPALQTSLKNLQLDYVDLYLIHWPVCIPPGELRMPGPNEKVLPID
YASVWEAMEECQRHGLTKSIGVSNFTCKKLELILASAKIPPAVNQVEVNPLWRQEKVIRFCKDRGIAVTA
YSPLGTAGSDFMGNRNVVQSELLKEIAKATGKTHAQVCIRWVFEQGVGVIVKSFKEMRLEENIDIFDWEL
SKEDSLKISRLPESKGYPGHGFIRVEGPFKSLEELWDGEI

>XSICOR1
MGSVPEITLNSGHSMPVLGLGTASVPFAGYEVVKSAILSAIKLGYRHFDTAALYRTEQPLGDAIAEAIRL
GIIKSREELFITSRPWLNDTHHDRILPALQTTLQNLQLDYLDLYLIHWPLSIKPGNIRFPGPDEKILPMD
FESVWAAMEECQRLGLTRSIGVSNFSCKKLEKILASATIPPAVNQVEVNPLWRQEKLIQFCKSKGIVITA
YSPLGTVGSSFQGNNNIMECEVLKEIAGNRGKTHAQVCLRWVYEQGVCLLVKSFNEARLKENMEIFGWAL
SEEEANQINQLPQGRGYPGHNFITPDGPFKSEEELWDGEI

CXE

>AERCXE
MSNDHLETTGSSDPNTNLLKYLPIVLNPDRTITRPIQIPSTAASPDPTSSSPVLTKDLALNPLHNTFVRL
FLPRHALYNSAKLPLVVYFHGGGFILFSAASTIFHDFCCEMAVHAGVVIASVDYRLAPEHRLPAAYDDAM
EALQWIKDSRDEWLTNFADFSNCFIMGESAGGNIAYHAGLRAAAVADELLPLKIKGLVLDEPGFGGSKRT
GSELRLANDSRLPTFVLDLIWELSLPMGADRDHEYCNPTAESEPLYSFDKIRSLGWRVMVVGCHGDPMID
RQMELAERLEKKGVDVVAQFDVGGYHAVKLEDPEKAKQFFVILKKFVVDSCTTKL
>AMECXE1
MAENNSSIITTNVTNPYEYLKIVHNSEDDTLTRLDPVPITNPITNVQNCKDIPLNIQHKTWIRLFKPPQL
QQQKSSKKDDEFSKSLSNNQSFPLIIYFHGGGFIMNSAASNYEHVFCEFVTTRIPAFIASVEYRLAPENR
LPAAYDDAVEALNWVKNQALDTINGEQWLRNFVDFSKCFIMGCSAGGNIAYHACLRTLELDLEPIKISGF
ILNQPYFGGLRRTESELRLINNWMLPLVINDLMWELSLPIGANRDHVYCNPMVDIDNSSSTILGLVKKLG
LIRMKCLVIGCDGDPLIDRQIEFVKLLKEKGVMVESCIEEGGFHGVGFSCPEKTDELCVKLKDFILGTY
>BTHCXE1
VDQSQSQTPINPYNLLGLVHNSDDTLTRLISPTCRYEDDQSIIQDIPLNTAHKTWLRLFKPIESPRITNG
DKLPIILYFHGGGFILCSAAQTIFHDFCTSLANKLPAFILSVEYRLAPESRLPAAYNDAVQAINWVRDQA
LNLNGEPLLREFGDFSKCFLMGCSSGGNIVYHAGLQALD
LDIEPLKIRGLVLNQPFFGGTQRTKSELRLVDDKILPLSMSDMMWELALPRRVDRDHKYCNPMIHGDDDK
KNGLIKRCFVSVRSGDPLMDHQMEFVRMLERKGVHVVRWFDDNEKEGYHGIELYEPQKAEVLFAALIDFV
YSSEATE
>CMACXE1
MLPIIIYFHGGGFILFNADSTMNHDFCQSIATHIPALVVSVDYRLAPENRLPAAYDDAVDALNWVKDQGL
GKLNNSEVWLKEYGDFSKCFIMGCSSGANVAYHASLRAIEMDLEPAKINGLILHCPFFGSLERTESDSKV
INNQDLPLAVRDVMWELALPLGSTRDHVYCNPNIDHDGSSSGNMVGLIERCFVVGFYGDPLIDRQIQLVK
MLEEKGVKVETWIEQGGYHGVLCFDPMIRETFLEKLKHFILNDEFIY
>CMUCXE1
MDPYKFLSISPNPDGSLTRHTRIPTSPPTNNPTVASADFPLNPSNNTWFRLYKPTNTPTSAKVPIVIYFH
EGGFVLFSAASQPFHDCCAALASQLQALVMSVEYRLAPEHRLPCAYDDAIDAIAWVRGQALGGGNGWFGD
SVDFSRCFLMGSSSGGNIVYKAGLMSLEKGLIGGGAEHVKLVGLIMNQPFFGGVERTDSELRYADDRILP
LPAGDLLWELALPTGSDRDHEFCNPLKGVSGEIERLPRCFVNGYCRDPLIDRQRQFAKMLERNGVSVVER
FVDDGFHACELFDPDKARALALAIKDFIYDCGAGGAKSAL
>CMUCXE2
MRNKRMAALSFDNARLGHQVGQDHRQKPGVLIEEIDGLIRVYKDGHVERPPVVPNVDCIAYIESSLTCRD
VIIDKFTNVWARFYVPRTQGTKLPFLVYFHGGGFCVGSAAWSCYHEFLTKVATKAGCVIMSVNYRLSPEH
RLPTAYEDGVGALMWVRREILRGSSEQKWWWSHCNFSQVFIVGDSAGANIAHHVTTRVGQLGASSDEPNI
LSPMTLKGTILIQPFFGGEARTHSEKYMAQPPKSALTVAASDVYWRLSLPLGASRDHRWCNPIAKGAMDL
EELRVPPIMVCISELDILKDRNLEFCSAMKRAGKRVEHVVYMGVGHAFQVLNHSQLSQARAHEMMSHIKA
FINR
>CTRCXE1
MAGSNAVNESQRAVPLNTWILISNFKVAYNLLRRPDGTFNRHLAEFLDRKVPANVIPVEGVFSFDCFIDR
ATGLLSRIYRPAPADEAEILPGVIDLERPLSTEVVPVIIFFHGGSFAHSSANSAIYDTLCRRLVRNCKAV
VVSVNYRRAPENRYPCAYDDGWAALKWVHSRPWLRSGEDSKVHVYLAGDSSGGNIAHHVAMRAVEAGTEV

MGNILLNPMFGGMDRTESEKRLDGRYFVTIQDRDWYWRAFLPEGTDRDHPACNPFGPKGADLKGVKFPKS
LVVVAGLDLVQDWQLAYVEGLEKAEQEVKLIYLERATIGFYLLPNNDHFYSVMNEINKFVNSNC
>ECACXE1
PQLLILTTYFKIIIKLKYFFNLSSLSTMEDKSSSLISNINPYEALQLVYDAQADSLTCNLPVPTIESSGD
PNCKDIPLNIEKKTWVRIFRPTTITTPTTTSVTTATTTTNKLPIIIYFHGGGFILFSAASGMFHDFCKSM
AAQVQALVVSVEYRLAPEKRLPAAYDDATDAINWVRNQAMDPVNGEPWLRDYADFSKCFLMGCSAGANIS
YHVGLKACELDLEPIKISGLILNQPFFGGSKRTESEIRLRDDQILSNPVIDLMWKLSLPIGVDQDHVYSN
PMANNGGGNVKVIKRIFVNGCDGDPLFDRQVEFVKMLEDKGLKVEASMEEGGFHGMTFLDPKQTDFFCKK
LKDFIFLSQVV
>GFLCXE1
MQSSSPQIKNTNKTRKNPNPTMADQPPTTINPYEALKIVHNPDDTLTRNLPIPDTPSSDDPNCKDTSLNP
QHKTWIRIFRPTKTQSQDNDFSITNKLPIIVYFHGGGFILCSAFSTIFHDFCKNMATQLPALVLSVEYRL
APESRLPAAYDDAVDALNWVRNQALETANGEEWLRDYADFSNCFIMGCSAGGNIAYHTGTRALELDLEPI
KISGLILNQPYFGGVERTGSELRLINDKALPLAMNDMMWELGLPIGADRDHFYCNPMVKIDGESGTRDEG
KLGLRMIRCLTIAGYDDPLFDRQIELGKMLEGKGVKVVRFIDKEGYHGMVHFDQKKAEEMFVAVKDFISS
TN
>HCACXE1
MADQNSSVEPIDPYKFLNIIYNPSDDTLTRNNHFPSTPITPDVKNAKDISLNTTKKTWVRLFRPNEPSLN
RKLPIIVYSHGGGFILQSASDTVFHNFCASMANILPALVVSIEYRLAPESRLPGAYEDTIEAINWVKNQA
LDMNGEQWLKDFADFSNCYLMGSSAGGNIVYHAGLRALSLDLDPLIIKGFILNSPFFGGSKRTESELRLI
NDTLLPLAVNDLMWELSLPKGCDRGHEYCNPMVDSSYKQNIGLIEKCFVSVGGEDPLMDREMEFVKMLKE
KGVKVVVWFDKEGLHGMEITVPGKDQALFVLLKSFVY
>HCACXE2
MANPNPVTIDPFKQLIITLNPDGTLTRPTLVPIVPPTGEINSHSKTPSVISMDVPLNPSKNTWIRVFQPS
NLSSNSKHPLIIYFHGGGFVLYSAASKPFHESCCQMASELSAVILSIEYLLAPENKLPSAYEDAEDAMLW
VKKQALNCEYKFCEFVDFSKCFIMGSSSGGNITYQAGFRALKLDLEPITINGVIMNQPFFGGVERTESEL
RLINDRILPLPTCDLMWEFALPIGSNRDHEYCNPIGSHHNEIKWLPRCYVRGYGGDPLIDRQMKFVKMLE
DHGACVVKHFLDDGFHACELFDPKMAEALYVDLRDFIYSS
ESNAVVKSKY
>JDICXE1
MDPYDALNFILNPDGSITRLDSSVPTTPPTGYDYNDSSVASIDVPLNTTTNTWIRIFHPLNLPSDTKLPI
IVYFHGGGFLLFSAASLTLHDFCVHLAGETPALVLSVDYRLAPENRLPAAYEDAVDAVLWLKQQALHGEH
KFTTFVDFTQCFIMGSSSGGNIAYHAGLRALNLNISPVEIRGLILNIPFFGGVERTESELKCVDDPILPL
PVADLMWELALPVGTDRDHEYSNPMVEGSNQKDIQRLPTCYVNGYEMDPLVDREKQLVEKLKGHGVCVVE
HFFDDADAYHAVEFFDPDKGRALVMDLKDFIYSPVTAASV
DKPIE
>MCACXE1
MENQQPPLLNTTDPYETLKIVYNANDDTLTRLYPIPCTAATGDHHNLPIITKDICLNTTHKTWLRLFIPT
EPPPSPNSLPLIIYFHGGGFVLCSAANTLFHDFCARIAADLPAVVASVEYRLAPERRLPAAYEDAVEAVN
WVREHGIKEKWTRDFVDFSKCYLMGNSSGGNIAYRAGLRCGSLDLEPVKIVGLVLIQPYFGGVERSESEM
RLANDGHFPLVVNDLMWELALPRGADRDHEYCNPMVAGAQWEKIGKCFVSVRGGDPVMDRQIALARRLEG
EGVKVESWFEEEGYHGIELFDPVKASELLLKLKHFICPPEAAEYRVVEK
>MCACXE2
MDDPYKFLNIVPNPDGSLTRLTQIPTSPPSHHPEATSNKSINSSLNDHSVISMDFSLNPATNTWLRLFRP
NTTTPQHVKLPIIIYFHGGGFVLFSAASQPFHDSCAAMAADLPALVLSVEYRLAPEHRLPAAFHDAVDAI
VWVKQKALEQHWLCDLADFSKCFLMGSSSGGNIAYHAGLRALGLDLGPVKVSGLILNQPFFGGVVRTESE
LRLAGDRILPLPAGDLMWELALPVGSDRDHEYCNPIMKDSNGGDIERLPRCFVNGYGGDPLVDRQKEFVK
MLEQCGVSEVARFVDDGYHAVELFDQSKAKDLVMAIRAFI
YTTSATTTPKSTL
>MCACXE3

SDLRDPSVIDEPYQFLKIALNPDGSLTRGYEFPRVPPTPELASESANLSSRRQPALSKDIPLNTSNQTFL
RLFRPLHHSKKLPIIIDFHGGGFVILSAISAPFHDMLSRAAAFAPALIITVEYRLAPEHRLPAAYDDAME
AIMWVRDQAREINGCDPWMKELADYSNVHIMGTSAGGNI
AYQAGLRALDVDINPVQIRGLILNQPFFGGIQRTQSEEILSRNPYLPMAVSDLLWVLSLPPGSDRDHEYS
NPLDGVNQKIQCLPRCLIRGFAGDALVDRIKGLAKMLDAHGVKVVVKIEDGGFHGAEMADPSKVQELFLQ
IKDFVYSS
>NDOCXE1
PIQQTLSNSGELKLPLVLYFRGGGFILCSVANTFFHNFCTSLATNLPALILSVEYRLAPESRLPAAYDDV
IEAINWVRNQGLDMHGGEEWLRKYGDFSKFFLMGCSSGGNMAYHAGMHALGLDIKPLKIQGLILNQPFFG
GTKRTESELRLVNDQKIPLAVSDLMWELALPKGVDRDHK
YSNPMVHDDDDDQEKIELMKGCSWFVSMRGGDPLMDRQMEFVRMLEKKDVKVVKWFDDEGYHGMELFEPQ
KAEELLVALKNFIRSEALE
>NDOCXE2
MDPFKVLNLIPNPDGSFTRPPIFPYAPPTGYTDSTPPQNVATIDVPLNSMKNTWIRIFHPLNLPSVSTKL
PVILYFPGSGFVMDTARILPLNEFCIQIAGELPVVILSVQYRLAPENRLPSAYEDAVDAMLWLKQQALHG
EHKFIDSADFTKCYIMGSSSGGNMAYQAGLRALNLDLEPVRINGLIMNQPFFGGVERTGSEMKYENDKVL
PLLATDSMWELSLPIGADRDHEYCSPMVQVWDLVQIQKLPKCYVNGHGGDLLVDRQKEFVKMLEGQGVSV
IAHFFDDGCHAVELFDQEKAQALYMDLKEFIYSSTTGDGA
HGS
>NSACXE1
MADENPNSVALDPYKVINIVLNPDGSLTRPTQVPNLPTSDDPKSVISLDIPLNPSNKTWMRVFQPPNLSS
NQKLPLIIYFHGGGFILYSPSSRPFHVSCIQMATQLPAIILSVDYRLAPENKLPSAYDDAVDALLWVKNN
AMKGEYKFWEFIDLSKCFIMGSSSGANITYHVGLKALVLDLEPVKIKGLIMNQPFFGGIQRTGSELRFVN
DKVVPLVVADLMWEFCLPVGADRDHQYCNPMAEGGSEEEEIKELPRCYVRGHDGDPLIDRQKEFVEMLEG
RGVSVVKDFSDDGFHACELFDATKGQELIEKVKEFIYSSS
>NSACXE2
MADENPNSVALDPYKVINIVLNPDGSLTRPTQVPNLPTSDDPKSVISLDIPLNPSNKTWMRVFQPPNLSS
NQKLPLIIYFHGGGFILYSPSSRPFHVSCIQMATQLPAIILSVDYRLAPENKLPSAYDDAVDALLWVKNN
AMKGEYKFWEFIDLSKCFIMGSSSGANITYHVGLKALVLDLEPVKIKGLIMNQPFFGGIQRTGSELRFVN
DKVVPLVVADLMWEFCLPVGADRDHQYCNPMKGSQNQEIKRLPRCFVRGYGGDPLIDRQKEFVKMLEEEG
ASVIKYFLEDGFHACELFFPDKAQALYVDLKDFIYSSPET
NKSTL
>NSACXE3
MAAQNQNSTINPFEALSIVLNANGSLTRHPHPLSTLSLNTTKSVVCTDIPLNPNRNTSIRIFQPPIHPTT
PKIPLIIYFHGGGFLIFSAKSPPYSEFCLQIASELPALVLSVGYSLAPEDRLPSAYEDALDAVVWVKNHA
FKGEYKYDFFDYVDFSKCFIMGSSSGGNITYQLGLQALKLNLEPFRINGLIMIQPFFGKVERSGSELRLA
NDNILPLPVTDLMWELSLPVGADRDHQYCNPMAEGGSEEEEIKELPRCYVRGHDGDPLIDRQKEFVEMLE
GRGVSVVKDFSDDGFHACELFDATKGQELIEKVKEFIYSS
S
>NSACXE4
MAAQNQNSTINPFEALSIVLNANGSLTRHPHPLSTLSLNTTKSVVCTDIPLNPNRNTSIRIFQPPIHPTT
PKIPLIIYFHGGGFLIFSAKSPPYSEFCLQIASELPALVLSVGYSLAPEDRLPSAYEDALDAVVWVKNHA
FKGEYKYDFFDYVDFSKCFIMGSSSGGNITYQLGLQALKLNLEPFRINGLIMIQPFFGKVERSGSELRLA
NDNILPLPVTDLMWELSLPVGADRDHQYCNPMKGSQNQEIKRLPRCFVRGYGGDPLIDRQKEFVKMLEEE
GASVIKYFLEDGFHACELFFPDKAQALYVDLKDFIYSSPE
TNKSTL
>NSACXE5
MDTFKQLSITYNPDDDTIDRLVTIPCTTTNHHNSLPNQQIFTHDIPLNSSHKTSLRLYKPINPPLHAKLP
LVVFIHGGGFLFASPFNTIYNDFCTSMAISLGVVVLSVGYRLAPESRLPAAYEDALEALTWVRDQADNIL
LKDMVDFSNCYLMGDSAGGNIVYHAGLRALSINLEPLVIKGYIFIQPFFGGMKRTESEVRLADDKILSSV

VSDLLWELGLPKGSDRDHEYCNPMVGGGFSDKIELLTRCFLVALGGDLLVDRQKEFMKMLEEKGVEVVSW
FEDEGCHGMFLFEPEKALHLFEVIKDFIYQPMPTV
>PBRCXE1
MADQHSTTTSSTIDPYEALMVVHDPDLDTLTRNLPLQITNTTDDPNSKDILLNAQHNTWIRIFKPTIQDL
PTANKLPVIIYYHGGGFILCSTFWTIYHDYCKSKANALPAIVLSVEYRLAPESRLPAAYDDAVDALNWVK
HQASGGKPSEPWLRDYADFTNCYIMGESAGGNIAYNVSLRASELDLSPLKISGVILNQPFLGGIERTSSE
LRLINDKILSLPVSDLMWELSLPIGSNRNH
PYCNLLINEDDESLRKKCGFIKKCLVIGCDGDPLIDRQVEFVKMLEGKGVKVETFMQEGGYHGMVYFEPE
ELEIMLPRVKDFILSGASKL
>PBRCXE2
MADQHSIDPYEALMVVHDPDLDTLTRNLPLQITNTTDDPNSKDILLNAQHNTWIRIFKPTIQDLPTANKL
PVIIYYHGGGFILCSTFWTIYHDYCKSKANALPAIVLSVEYRLAPESRLPAAYDDAVDALNWVKHQASGG
KPSEPWLRDYADFTNCYIMGESAGGNIAYNVSLRASELDLGPVNITGVILNQPFMGGIERTSSELRLIND
KILSLPVSDLMWELSLPIGSNRNHPYCNLLINEDDESLRKKCGFIKKCLVIGCDGDPLIDRQVEFVKMLE
GKGVKVETFMQEGGYHGMVYFEPEELEIMLPRVKDFILSGASKL
>PBRCXE3
MIVYFHGGGFILCNANSTIFHNFCKSMANQLSAIVLSVEYRLAPENRLPAAYEDAVESLVWVQNQALDAI
NGEPWLRNYGDFSKCFIMGCSAGGNIAYNSCLGASELDLEPIKICGLILNQPYFGGVERSKSELRLVNDK
CLPSVVNDLMWELGLPVGVNRDHVYCNPFVDENLETKLRFIRRCLVTCSNEDPLVDRDIQFVKMLEGKGV
KVVSWIEEFGFHGMAHSDPVKAQELLKVVKDFIFSED
>PSOCXE1
MADPYEFLMCIHNPEEDTLTRNFPIPATPLDQNTKDISLNPDRKTSLRIFRPPTKEPPVTKNKLLPIIIY
FHGGGFILFNADSTMNHDFCQSIATHIPALVVSVDYRLAPENRLPAAYDDAVDALNWVKDQGLGKLNNSE
VWLKEYGDFSKCFIMGCSSGANVAYHASLRAIEMDLEPAKINGLILHCPFFGSLERTESDSKVINNQDLP
LAVRDVMWELALPLGSTRDHVYCNPNIDHDGSSSGNMVGLIERCFVVGFYGDPLIDRQIQLVKMLEEKGV
KVETWIEQGGYHGVLCFDPMIRETFLEKLKHFILNDEFIY
>PSOCXE2
MADPYEFLMCIHDPEEDTLTRNFPIPATPLDQNTKDISLNLDRKTSLRIFRPPTEEFCVTTNKLLPIIIY
FHGGGFVLFNADSTINHDFCQSIATHLPALVVSVDYRLAPENRLPAAYDDAVDALNWVKDQGLGKLNNSE
VWLKEYGDFSKCFIMGCSSGGNIAYHASLRAIEMDLEPVIINGLILHSPFFGSLQRTESDLKVINDQDLP
LAVRDVMWELALPLGSSRDHVYCNPNIANDGSSSGNMAGLIKRCLVIGFYGDPLIDRQIQLVKMLEEKGV
KVETWIEQEGYHGVPCFDPKIRETLLGKIKYFI
>SCACXE1
DHHQLSQLAASVNYSIDPYDRLKIVHNSDDDSLIRLVPLPSYPSTDDDDQNSCKDILLNPQHKTSIRIFR
PTTTQFQQSSKKYDFSVKNKLPPIIYFHGGGFILGSAFNIVFHDYCKSMATQLPALVLSVEYRLAPENRL
PAAYDDAMDAVNWVRNQALDAINGEPWLRDYADFSKCFLMGFSAGGNIAYHAGLRALELDLEPIKISGLI
LNQPYFGGSKRTRSELRLTNDPILPCPMSDLMWELALPRGANRDHMYCNPMVDDVTRRGNRNKVGLVIRR
CLMAGCDGDPLIDRQIEFVKMLEGKGVKVETWMEEGGSHGMVFADPKKAEILFGKLKDFIFTDIVTKGAA
N
>SCACXE2
SIDVPLNTDKNTWIRIFLPSNLPSNNSKLPIIIYFHGGGFIVFSSASLPYHEFCVRLASELPAVILSVEY
RLAPESRLPSAYEDAIDAINWLKKQALNGGEHKFVDFVEFTGCFLMGSSSGGNIAYQAGLRVADLDIEPV
KINGLILNQPFFGGVERTGSELRYANDEILPLPVSDLMWEFALPIGANRNHEYCNPMVEKSHYCKIRMLP
RCLINGNGRDPLVDHQKEFVKMLERNGVCVVAHFADDGSHAVELFDPEMARALVTNVRKFIYSSATDVGV
STTGAAKSSIPIDRLLFCK
>SDICXE1
ESLPPINPPSYKPLTMADQSSTTINPYEALNIVHNPDDTLTRNLPIPDTPSSDDPNCKDIPLNPQHKTWI
RIFRPTKTQFQDDQNKKIPVIIYFHGGGFIICSPSSTIFHDFCKSMAIQSSALVISVEYRLAPESRLPAA
YDDSVDALNWVRDQALDVINSEEWLREYGDFSNCFIMGESAGGNIAYHACLRSLELDLEPIKITGLILNQ
PFFGGVERTGSELRLINDKVISIVMSDLFWELGLPIGADRDHFYSNPMMVENNGGTSTINWLMIKRCLVI

GKMLERKGVKVVSWIEEEGCHGKVHSDQKHAEEMFKVVKDFIRA
>TCOCXE1
MEDHDDVPSINPTDFFKSLKIIYNPETDTLTRLFTVPCTNPTNTDDDHHHSPAVSSQPVLTKDLPLNSSY
HTWLRLFLPTGPPPSKKLPLIIYFHGGGFIFMSAANTMFHDFCSRVAVELPAVVLSVEYRLAPESRLPAA
YDDAVDAIHWVREQALVAGDDKGHDLEDVWLREQVDFPTCYLMGLSAGGNIAYHASLRSLHLDLDPLKIV
GIILIQPYFGGVQRTASEMRLGNDENFTVGMTDVMWELSLPKAADRDHEYCNPMVGDYGEKIGQLGRFLV
CVRGGDMLRDRQMELVKMIEEKGGKVKCWFEEEGYHGVELSDCEKASELLVKIKHFIYEK
>TCOCXE2
MEDHDDVPSINPTDFFKSLKIIYNPETDTLTRLFTVPCTNPTNTDDDHHHSPAVSSQPVLTKDLPLNSSY
HTWLRLFLPTGPPPSKKLPLIIYFHGGGFVFLSAANTMFHDFCSRVAVELPAVVISVDYRLAPESRLPAA
YDDAIDAIYWVREQALVVGDDKAHDLKNVWLREYVDFSTCYLMGLSAGGNIAYHASLRCLPLDLEPVKIV
GMVLIQPFFSGVQRSGSEMRLGNDGMFTLDMSDAMWEYALPKGADRDHEYCNPMVGDYGEKIGQLGRFLV
CVRGGDMLRDRQMELVKMIEEKGGKVKCWFEEEGYHGVELSDCEKASELLVKIKHFIYEK
>TCOCXE3
MSDQVAPFTPVPITDPYDYVDLVHHHDGTITRKKPSNFPTVPTVPDLSYPAPVLSKDIPINQSNNTWARV
YISRYKLESNSTNDLLPLIVYYPGGGFVISTTATPTCYNFCSKLALQLSAVVVSIDYRVGPEHLLPAAYD
DAVEALNWIKFAEDSWLRDHANLSNCFIMGCSAGGNIAYHVGLRVAMEDVDSFKPLKINGLILHYPFFGG
HERTESELRMANDLILPLIATDVMWKYSLPAGANRDHEYSNPAVVDRSKLFDKIKMLGWKILMSTGDKDP
GIDRQIGLLTTMKEKGVKVKGRIVEGDHHGEELLNNSHKLDSLLVALKDFIFSTFSE
>TFLCXE1
MAVPLNTWVLISNFKLAYTMLRRPDGTFNRHLAEFLDRKVPSNANPVDGVFSFDVIIDRATDLLCRVYRQ
ALATEGRPSFLQLEQPLSSDVVVPVIIFFHGGSFAHSSANSAIYDTLCRRLVRNSKAVVVSVNYRRAPEY
RYPCAYDDGQAALKWVHSRPWLRSGKDSKAHVYLAGDSSGGNIAHHVALRAVESGTEVLGNILLNPMFGG
VERTESEKKLDGKYFVTLQDRDWYWRAFLPQGADRTHPACNPFGPNAASLEGVKFPKSLVVVAGLDLLHD
WQLAYVQGLQRAGQDVKLMFLEQATIGFYLLPNNIHFFSLMDEINNFLSC
>XSICXE1
MAEQNPSILPINPYEVLGIVHNPDDTLTRQIQIPCTTTSTDDISCAKITKDIPLNACNKTWLRLFRPIEQ
PVNVKLPIILYFRGGGFILCSASNTIFHNFCASMANKFPALIVSVDYRLAPESRLPSAYEDAIEAITWVK
KQALDTNGEQWFKDLADFSKCYLMGCSSGGNIVYHVGLRAISLDLKPLLIKGLILNQPFFGGLRRTESEM
RMVNDKTLPLVVSDLMWELALPAGSDRDHEYCNPRVDGGFKENIKLLSRCFVSVRGGDPLMDHQKEFVKM
VEEEGVKVVNWFDEAGYHGMELFEPQKAEAMFVALKAFIYSSSDIE
>XSICXE2
MASQADQAHVDRYKEHNIIYNPDDTLTRLITFPCTSTTDHNSLPNQPVFTQDIPLNPINKTWMRLFKPSN
PQPPKTKLPLILYFHGSGFIIGSASNSTFHDFCTSMAIELSCVVLSLGYRLAPESRLPAAYEDAMEAIDW
VRNKALDLGSDNCLKEVVDFSKCYLMGISSGGNMVYHAGLRVLSMDLHPMIIKGLILNHPFFGGLKRTNS
ELRLANDKFSLAFTDIMWELSLPKVFDRDHEYCNPMVEGSNKDKIGLLGRCFVSGCGGDILIDRQMEFAK
MLEEKGASVVTWFEEEGCHGVELFEPEKTRALFFLLKEFISGQ
>XSICXE3
MADQNTIIDPYKHINVVLNPDGTLTRPTLVPIIPPSSDPNSNSVISLDIPLNPSKSTWMRVFQPPNLSCN
QKLPLIIYFHGGGFVLYSASSRPFHESCVKMASELPAIIISVDYSLAPENKLPSAYEDGMEALLWVNNQA
INGEFEFCKFVDFSKCFIMGSSSGANITYQVGLRALKLDLEPVKIIGLIMNQPFFGGIQRTGSELRMVND
RIIPLVVADLMWELALPIGADKDHEYCNPMAAHFNEIKQLPRCFVNGYGGDPLVDRQKEFAKMLGEHGAC
LVEHYSDDGFHACELFIPDKAQALVMNIRDFIYSPGIGSNAATSTL

CYP80

>HSACYP1B1
MGTSLSPNDPWPLNPLSIQQTTLLLLLSVLATVHVGQRLLRQRRRQLRSAPPGPFAWPLIGNAAAVGQAA
HLSFARLARRYGDVFQIRLGSCPIVVLNGERAIHQALVQQGSAFADRPAFASFRVVSGGRSMAFGHYSEH
WKVQRRAAHSMMRNFFTRQPRSRQVLEGHVLSEARELVALLVRGSADGAFLDPRPLTVVAVANVMSAVCF

GCRYSHDDPEFRELLSHNEEFGRTVGAGSLVDVMPWLQYFPNPVRTVFREFEQLNRNFSNFILDKFLRHC
ESLRPGAAPRDMMDAFILSAEKKAAGDSHGGGARLDLENVPATITDIFGASQDTLSTALQWLLLLFTRYP
DVQTRVQAELDQVVGRDRLPCMGDQPNLPYVLAFLYEAMRFSSFVPVTIPHATTANTSVLGYHIPKDTVV
FVNQWSVNHDPLKWPNPENFDPARFLDKDGLINKDLTSRVMIFSVGKRRCIGEELSKMQLFLFISILAHQ
CDFRANPNEPAKMNFSYGLTIKPKSFKVNVTLRESMELLDSAVQNLQAKETCQ
>AMECYP80-1
MDILSISVIAIVITSLLYLILRDSSPKDLPPGPKPWPIVGNLLQLGEKPHSQFAQLAKTYGDLFTLRLGT
ETVVVASTPLAANEILKTHDRILSGRYVFQSFRVKHHVENSIVWSECNDTWKNLRKICRTELFTQKMIEN
QAEVRERKTWEMIEFLKKKEGEEVKIVEIIFGTLVNIFGNLIFSKNIFELGDENSGSVEMKKHLWRMLEL
GNSTNPADYFPFLGRFDLFGQRKDVADCLQGIYDVWGVILKERKVEKKKRIDEEEKKKNDFVDILLDSGL
DDQQINSLLMELFGAGTETSASTLEWGIAELTKNPKIKTKIRSELMTVVGQNTVKESDIPNLPYLQAFVK
ETLRLHPPTPLLLPRRALDTCRVLNYTIPKECQIMVNAWGIGRDPKTWNDPLTFSPERFMNSNIDFKGND
FELIPFGGGRRICPGVPLATQFISLIIATLVQNLDWELPMGMDPSDLIMEEKFGLTLQKEPPLFIVPKFR
V
>BTHCYP80-1
MSKNSPPFLDYMSNIAQKYGPLVHLKFGLHSSIFASTKEAAMEVLQTNDKALSGRKPLPCFRIKPHIDYS
IVWSDSNSYWKNGRKILHTEIFSQKMLQAQEKNRERVAENLVNFIMTKVGNVVELRSWLFGCALNVLGHV
VFSKDVFEYSDQSDEVGMDKLIHGMIMTGGDFDVASYFPFLARFDIHGLKRKMDEQFKLLIKVWEGEVLA
RRANRNPEPKDMLDVLIANDFNEHQINAMFLETFGPGSDTSSAIIEWALAQLIKNPDKLAKLREELDRVV
GRSSTVKESHFSELPYLQACVKETLRLYPSVTIMIPHQCMETCQVMGYTIPKGMDVHVNAHAIGRDPKDW
KDPLKFQPERFLGSDIEYNGKQFQFIPFGSGRRICPGLPLAVRTIPLVLASLVHAFDWELPDGVPNEKLD
MEELFTLTLCMAKPLQVIPKVRI
>BTHCYP80-2
MGPLALFALVFIIPFLLLITLFKPSSKNLPPGPPAWPLIGTLLPKLKKQPHVELTKLAKSHGPLMLLKFG
VEPVVVASNHVAAMEVLKTHDRELSGRFAPHSVRIKGYVEHSMVWADCTDYWKLVRKIWRTDMFSTKMLD
AQACVREEKVLELVGFLKGKQGEVVKFADVVFGCILNILGAIVFSQNVYDFEDKSDNELGMKGMIRQLMI
LAAIPNLADLYPILGKSDFQGLKKASAACVTRMNESWATVVKERRRNSDHLKNDFLNVLIKAGFSDPQID
AMLLEIFGPGSDTSASTIEWAMAELLKSPEKLKNVQNELKKVIGANQVVKESDLPDLPYLNACVKETLRL
HPPVTFLLPHRALEECQVMGYTVPKGSQLMVNTYAIGRDPETWNNPLSFEPERFLSDTEIDYQSSTDMKY
IPFGAGRRICPGLSLAARLVPLVLASLIHAFDWGLPGGMDPSEIDMKDKFGLALLKDDPLLIIPKVTKV
>BTHCYP80-3
MDLNTALLYLIPVVLVYYILFKPKHKNLPPGPKPWPLIGNIPVLFGSGKPLHVTLTDMARTHGPIITLWM
GTQPTAIASTAESAMEILKTHDRLFSGRAVRMSFRLKHHIKYSLVWADCSEYWKLLRKIVRTEIFSPKML
QVQEHVREKKMEEMVEFLKGKEGEVLKLSPLVFGTLLNVLGNVVYSKDVFGYFGDDGDQMQTLMKEMLMI
GAAPNVAEFWPILEELDLQGLKKKCHERFTEVMKMWKGTVEERKVNRNAEPKDMLDVLLANDFNDDQINA
LFLETFGPGSETSSATIEWTMAELIKNPKIIAKVREELDQVVGKSLVKESHLPNLPYLQACIKETMRLHP
AAPFLLPHRSVEACQVMGYTIPKDIQVLVNAYAIGRDPKTWKDPTTFKPERFMESDIDYYGKHFEFIPFG
SGRRQCVGMPLATRTIPLIVSNLVHAIDWSLPDGKNLEELVMNETLSLSLALDPTLTVIPKARV
>BTHCYP80-4
MEEVLGESNLFFLLLLALLPFFFFILNYIKSPTLPPGPFSWPIIGNLLKMGTKPHVSLAQLAHTYGPIIS
LRLGTRLLVIGSSPSAAIEILKTHDRLLSARTIPDASPTKDPAHTKFSLAWADCTDQWKYLRTICRSELF
SIKAIESQSKMREEKVIELVKFLGTKDGQLVRIDEVVFDTMVNMLSNIFVSQDFVNMEERDSEEGLRGLV
RKFVEVAATPNIADFYPILSVLDLHGLKKRSKELFVKICDLWGVMIKERREGKTGNKDFLDFLLANGFIN
DQINCLLLELFSAGSDTSTSTIEWAMAELMKNEEAMKNVLSELEREIHTNMVKESDLPQLPYLHACVKET
LRLHPPAPLLLPHRALETCKVMNYTIPKDTQVLVNVWAIGRDPMVWNDPLAFKPERFLDSNSLDMNLSDF
RFIPFGAGRRICPGLPMATKQVALILASLINCFHWSLPHDMHPLELDMEEKFGVTLQKEQPLVLIPRVKT
>CCHCYP80-1
MEILSIAILSVVISTLIYYFFFSDSKAQKGLPPGPKPWPIVGNLLQLGDKPHSQFAEMAQIYGDLFTLKM
GTETVVVASTPAAAIEILKTHDRLLSARYVFQSFRVPNHVENSMVWSDCNEVWKMLRKVCRTELFTPKMI
EAQAHVRESKALEMVKFLKGKQGGEVKIAEVVFGTLVNIFGNLIFSQDVFELGDPSGGSAEMKEHLWRML

ELGNSTNPADYFPILGRFDLFGQRKDVAECLRLIYDVWGVMLKERKVKKAAQGNKGNDNKDFVDVLLDSG
LNDFQINALLMEIFGAGTETSSSTIEWSITELTKNPHITAKLRAELESVVGQNPVKESDIPNLPYLQACT
KETLRLHPPTPLLLPRRAIETCKVMNYTIPKDCQIMVNAWAIGRDPKIWKDALSFSPERFLNSSVDFKGN
DFELIPFGAGRRICPGVPLATQFISLIVATLVQNFEWNLPNGMDPKDLVMDEKFGLTLQKEPPLYIVPKS
RV
>CCHCYP80-2
EFFSITILSLLITSLIYYLFSDHSKTQKGLPPGPKPWPIVGNLLQLGDKPHSQFYEMAKTYGDLFTLKMG
TQTVVVASTPAAATEILKTHDRLLSARYVFQSFRIPKHVENSMVWSDCTDAWNMLRKICRTEIFTTKMIE
AQAYVRESKVLEMVKFIKGKQGSEVKVLKVVFETLVNVFGNLIFSQDVFQMGDPSGEGAVMKGHIWRMLE
LGNSTNPADYFPILGRFDLFGHRKDVEECLRQIYDVWGVMLKERKAKKAAIQEKGGNYNNSDFVDVLLDS
GLEDFQMNILLMELFGSGTETSSSTIEWAMAELTKSPDIAAKLREELSSVVGQRPVKEADIPNLPYLQAI
TKETLRLHPSTPLCIPRRAPETCQVMNHTIPKDCQIIVNVWAIGRDPKAWEDACKFSPERFLNSSIDFKG
NDFELIPFSAGRRICPGMPIAIQFVNLFVATLVQNFEWDLPNGMDPRDLVMDDKFGLTLQKEPPLYIVPK
SRV
>CMACYP80-1
MEIITVALIAVVISSILYLIFRDTSPKGLPPGPKPWPIVGNLLQLSDKPHAQFAELAQTYGDLFTLRMGT
QTVVVASTPTAAAEILKTHDRILSGRYVFQSFRVKEHVKNSIVWSECNDTWKSLRKVCRTELFTQKVIES
QAEIRERKAWEMVEYLKKKQGEEVKIVEVIFGTLVNIFGNLIFSQNIFDLGDPSSGSVELKEHLWRMLEL
GNSTNPADYFPIMGRFDLFGQRKEVEDCLQGIYDVWGAMLKKRKAAKEAGNSEGGNDFVEILLDSGLTDQ
QINSLLMELFGAGTETTASTLEWAITELTKNPNVTAKMRSELETVVGHNTVKESDIPNLPYLQAFVKESL
RLHPPTPLLLPRRALETCRVLNYTIPKECQIMVNAWAIGRDPKIWTDPLTFSPERFLNSSIDFKGTDFEL
IPFGGGRRICPGIPLATQFIGLIAATLVQNFEWDLPNGMSPKELNMEEKFGLTLQKEPPLYIIPKSRA
>CMUCYP80-1
MEVVAATIGLIAFAIFIYIFFIKDQSSKKGLPPGPKPLPIVGNLLQLGDQPHAQFAQLAQTYGDLFSLKL
GSQTVVVASTPAAATQVLKTHDRIFSGRYVFQSFRVEKHVNNSIVWSECNENWKMLRKVCRTELFSAKMI
ESQAHLREAKAMELVKFLKGREGQVVKITEVVFGVLVNIFGNLIFSQDVFDLSDPTGGSAEMKDHIWQML
EMGNSANPADYFPFLGRFDLFGQRRAVANCLQQIYDVWGAMLRERRRAAKKGDGSTPPHHDFVSILLDAG
LDDQKINALLMELFGAGTETSSSTIEWAIAELTKNPSIKEKLLLELDNVVGKEEVKESDIPRLPYLQAFV
KETLRLHPATPMLLPRRALETCQVMGYTIPKDCQIMVNAWAIARDPKIWKEPLRFLPERFLDSSLDFKGN
DFEFIPFGGGRRICPGIPLATQFIGIIVATLVQNLDWSLPNGMDPSELVMEEKFGLTLQKEPPLLIVPKA
KDLCK
>CMUCYP80-2
MEVVTLLLSLLSSIPLILLVYLLLLLKPKPNLPPSPPSWPLIGSIPAILGSSKPLHLTLTDLAAAHGALM
LVTLGTAPTVVASTRAAAMEVLKTHDKSLSGRHIRMSFRIKEMNRHSLVWSNCTDTWKLLRRLARTEIFS
PKMLQAQEPVRERKVGELVHFLREREGAVVKISEFVFGTLLNIIGNVVFSKDVFGYGDKNEIGMQSLIKE
LLVIGASPNVAEFYPILEVLDLQGLRRRCADRVARVNMLWESTVKKRRISRGGDPEDMLDVLLDNGFNDV
QINVLFLEMFGPGSETSSATIEWALAELIKNPDKLAKVREELDRVVGPTSQVNEAHIPHLPYLQACIKET
MRLHPAAPFLLPHRAVETCQVMGYTIPKDYQLLVNAYAIGRDPESWNEPSKFMPERFLESEVDYNGNHFE
FIPFGSGRRICIGMPLATRTVPLIVSSLMHNFSWSLPDGRRNEELEMNEMLSLSLAIDPSLTIIPKARIL
HA
>CMUCYP80-3
MDQLTLLSLILLSTLLICLLFSFEFKRYISKRLPPGPFVWPMIGTVFMSVSEAQDHIVLADLARKYGPLM
MVKFGLAPPLMVASNHAAAAEILKTHDVACCGRDPPHSVKLPGYIEHSMVWGDCTEHWKMVRKIWRTELF
SAKMVDSQAGIREEKVRELVGFLRGRGGEVVQLTEAIFGCIINVLGSIIFNKNVYDYEGRVDNEKGMKGM
IRELIMLAATPNLPDFFPVFGLLDLQGIGKRTGECVKRMNEYWGVIVKERRASKDHSRNDFLDVLIQAGF
TDAQIDALLLEIFGPGSDSTSCTIEWAMSELIKNPNKLLKLQEELNTVIGQNREVKESDLENLPYLHACI
KETLRLHPPVTFLLPHRATKTCEVMSYTIPKESGIFVNTYAIHRDPTVWEDSSMFKPERFLNSTVDYQGN
DFQYIPFGAGRRMCPAHNMASRTTRIMLASLIHNFEWSLPNGMAPSELDMQDTFVMVLAKAVPLSVIARE
RSVCVSS
>CTRCYP80-1

VSATIGFIFFIIFIFFFLKKQNTSTKGLPPGPKPWPIVGNLLQLGDKPHAQFAQLSQTYGDLFSLKLGSQ
TVVVASSPTAATQVLKTHDRILSGRYVFQSFRIEKHVNNSIVWSECNDNWKMLRKVCRTEVFSTKMIESQ
AHLREAKAIEMVEFLKGREGQVVKIAEVVFGTLVNIFGNLIFSQDVFDLADPTSGSADMKDHIWKMLEMG
NSANPADYFPFMGKFDLFGQRRAVAECLQQIYDVWGAMLKERRGAARGGTGANNDFVSVLLEAGLDDQRI
NALLMELFAAGTETSSSTIEWAIAELTKNPPIREKIYRELESVVGKERVKESDIPRLPYLQGFVKETLRL
HPATPMLLPRRALETCQVMGYTIPQDCQIMVNAWAIARDPKVWKDPLKFSPERFLNSSLDYKGNDFEFIP
FGGGRRICPGLPLASQVHKASLWQLLC
>CTRCYP80-2
LSLLSFIPFVIVLYLLFKPKHNLPPSPRPSWPLIGNIPAILGSKTPLHVTLTDLAQVHGPLMVVTLGTAP
TVVASTREAAMEVLKTHDRALSGRHIRMSFRIKEMNKHSLVWSNCTDTWKLLRKLARTEIFSPRMLQIQE
PVRENKVGELVEFLRGREGKVVKISQFVFGTLLNIIGNVVFSKDVFLFDDDKIGMQSLIKELLMIGASPN
LAEFYPILEVLDLQGLRRRCADRVSKVNKLWESTVKERRLGRSGDAKDMLDVLLDNGFSDVQINVLFLET
FGPGSETSSATIEWVMAELIKNPDKLAKLRLELDQVVGPTSQVKEAHIPLLPYLQACVKETMRLHPAAPF
LLPHRAVETCQVMGYTIPQDYQVLVNAYAIGRDPESWKDPSEFRPERFLESNVDYNGNHFEFIPFGSGRR
ICIGMPLATRTVPLIVSSLVHNFYWSLPDGKSPEELEMNEILSLSLAIDPSLSINPKVRALNT
>ECACYP80-1
MEVVTVALIAVIISSILYLLFGGSGHKNLPPGPKPWPIVGNLLQLGEKPHAQFAELAQTYGDIFTLKMGT
ETVVVASTSSAASEILKTHDRILSARYVFQSFRVKGHVENSIVWSDCTETWKNLRKVCRTELFTQKMIES
QAHVREKKCEEMVEYLMKKQGEEVKIVEVIFGTLVNIFGNLIFSQNIFELGDPNSGSSEFKEYLWRMLEL
GNSTNPADYFPILGRFDLFGQRKEVAECLKGIYAIWGAMLQERKLAKKVDGYQSKNDFVDVCLDSGLNDY
QINALLMELFGAGTETSASTIEWAMTELTKNPKITAKIRSEIQTVVGERSVKESDFPNLPYLEATVKETL
RLHPPTPLLLPRRALETCTILNYTIPKDCQIMVNAWGIGRDPKTWTDPLTFSPERFLNSSVDFRGNDFSL
IPFGAGRRICPGLPIANQFIALLVATFVQNLDWCLPNGMSVDHLIVEEKFGLTLQKEPPLFIVPKSRV
>GFLCYP80-1
MEIVTIAVIAVVISSIFYFIFRDTSPKGLPPGPKPWPIVGNLLQLGEKPHAQFAELAQTYGDLFTLRMGT
QTVVVASSPSAAAEILKTHDRILSGRYVFQSFRVKEHVENSIVWSECNDTWKNLRKVCRTELFTQKMIES
QAHIRERKAWEMVEFLKKNEGQQVKIVEVVFGTLVNIFGNLIFSQNIFELGDPSSGSVELKDHLWRMLEL
GNSTNPADYFPIMGRFDLFGQRKEVADCLQGIYDVWGAMLKERKAARKAGGNQGKNDFVEVLLDSGLNDQ
QINALLMELFGAGTETSASTIEWAITELTKNPKVTAKIQSELLTVVGRSSIKESDIPNLPYLQAFVKETL
RLHPPTPLLLPRRALDTCRVLNYTIPKECQIMVNAWAIGRDPKTWTDPLSFSPERFLNSSIDFKGNDFEL
IPFGAGRRICPGVPLATQFIGLIVATLVQNFEWGLPNGMKPGDLNMEEKFGLTLQKEPPLYIIPQSRA
>HCACYP80-1
MELLSIAIAFLFFFFLRIIFHDSNRKNLPPGPRPWPIIGNLLQLGEKPHAEFAKLAQTYGDLFTLKLGSQ
TVIVASSPASATEILKTHDRILSSRYIFQSFRVKDHVENSIVWSECNENWKLLRKVCRTELFSPKMIESQ
AHVREAKALDMVKFLKRKEGEVVKMVEIVFGTLVNIFGNLIFSKDVFDLEDPTGGSAEMKEHLWKLLDMG
NSTNPADYFPIMGKFDLFGQRKAVAQVLQQIYDVWGVMLKERRNRKGSENNHDFVNVLLDAGLDDQKINA
LLMEIFGAGTETSASTIEWAIAELTKNPRVLSKIASELVNVVGEETVKESHLPNLPYLQAFVKETLRLHP
PTPLLLPRRASDTCQVMNYTIPKDCQIMVNAWGIGRDPKTWNDPLTFKPERFLNSNVDYKGNDFELIPFG
AGRRICPGLPLASQFINLIVATLVQNFEWSLPQGMSPSELTMEEKFGLTLQKEPPILLVLKARVSIKS
>HCACYP80-2
MDLTIALFSFIPIILVFVLLLKPKTKNLPPGPSAWPLIGNLPTLFSNPNVPLHVTLTNLAKTHGPVMLLW
LGTQPTVIASTDEAAMEILKTHDRVFSGRHVRMSFRLKHHIKYSLVWSDCTDYWKLLRKIARTEIFSVKM
LQVQSHVREQKVAELVEFLRSKQGQAVKISQFVFGTLLNILGNVVFSKDVFIYSDEEDKEGIQPLIREML
MIGAAPNVAEFYPILEELDLQGLKRRCADRFIQVMKLWEGTVKERKQNRNEESKDMLDVLLANDFNDAQI
NALFLETFGPGSETSSATIEWVMAELIKNPNEMAKVRKELDEIVGASNVKESHLPHLPYLQACIKEAMRL
HPAAPFLLPRRAVEKCEVMGYTIPKDCQVLVNAYAIGRDPKTWKGPSTFRPERFLESDIDYHGGHYQFIP
FGSGRRTCIGMPLATRTIPLIVGSLVQTYDWSLPGGKRPEELEMKEMLSLSLAIDPSLCVVPKLRA
>HCACYP80-3
MEETFVVIRKANLFYYLLLLVLSLLFLILKHIRSPNNLPPGPFPWPIVGNILNMGNRPHISVAQLAKVHG
PLMSLRLGTQLVVVGSSPAAATEILKTHDRILSGRCVPHATPASPNDVHLSMSWSDCTDQWRLLRTLCRT

ELFSAKVIESQANVRDKKVVELVEFLATKEGKMVQIVEVVFATIFNMLSNLFVSRDFIKLDEKREDGGVK
GLIKGLVELGFTPNLADLYDIFSGFDLQGLNKKSKEMFLKICGVWEVIVKERREGKSNDTSRQRDYLDAL
LDSGLTNGHINYMFLELFLAGTDTSTSTIEWAMAELIKNPEAMKKVKIELEREINGNVVTESDLPQLPYL
HSVVKETLRLHPPAPFLVPHCALETCKVMNYTIPKDTQIFVNVWAIGRDPNIWDDPLTFKPERFVESSMD
LNSNNFRFIPFGSGRKICPGLPMAAKQVPLVLACLIKCFDWSLPHDITPSKLNMDEKFGITVQKEKPLVL
IPKVKKS
>JDICYP80-1
MELITGVVIAISFFLVYLLISNRLSRKGLPPGPRPWPIIGNLLQLGEKPHAEFAKLAQKYGDLFTLRLGT
ETVVVASSPAAAAEILKTHDRILSGRYVFQSFRVKEHVENSIVWCQCNDNWKMLRKVCRTELFTPKMIES
QAHVREAKALEMVSFLRGKQGEVVKISEVVFGTLVNIFGNLLFSQDIFDLADPTSGSVEMKDHLWKMLQL
GNSTNPADYFPFLGRFDLFGQRRAVADCLQRIYDIWGALLKERRGSNASKDHHDFVNVLINAGIDDQKIN
ALLMELFGAGTETTASTIEWAVAELTKNPHIAAKVRQELLNVLGKDPIKESDIPRLPYLQAFVKETLRLH
PATPLLLPRRALETCQVMNYTIPKDCQIMVNAWGIARDPKIWNDAMTFSPERFLNSSVDYKGNDFELIPF
GGGRRICPGIPLATQFIHLIVATLVQNFEWKLPKEMDPSDLTMEEKFGLTLQKDPPLYIVPKSTL
>JDICYP80-2
MELTTALLYLIPTIFLIIFLKPRNKNYPPGPNPWPIIGSIPALFGSGTPMHVTLADMAHTHGPLMMVWLG
TQPTAVASTAESAMEILKTHDRLFAGRAIRMSFRLKHHIKYSLVWADTSEYWKLLRKIVRTEIFSPKMLQ
VQEHVREEKVAELVEFLKGKEGQVVGVSQLVFGTLLNILGNVVFSKDVFAYVGAEGDKMQTLIKEMLMIG
AAPNIAEFWPIFEEFDFQGLKKKCDQRFKVVMQMWEGTVEERKVKRNEEPKDMLDVLLVNDFNDAQINAL
FLETFGPGSETSSATIEWAMAELIKNPNIMAKVRAELDQVVGKSTVKESDLPHLPYLQACIKETMRLHPA
APFLLPHKSMETCKVMGYTIPKGIQVLVNAYAIGRDPKTWKDPTTFKPERFLESDIDYYGKHFQFIPFGS
GRRQCVGMPLATRTIPLIVSNLVHKIDWSLPDGKRPEELVMNETLSLSLALDPTLSVIPKLRV
>MAQCYP80-1
MDLITCLAILTLFFFLYFFITDHLSRKGLPPGPRPWPIIGNLLQLGEKPHSEFAKLAQTYGELFTLKLGT
QTVVVASSPAAAAEVLKTHDRILSGRYVFQSFRVMEHVENSIVWCQCNDNWKMLRKVCRTELFTPKMIES
QAHVREGKALEMVRFLRSKQGQVVKMTEVVFGTLVNIFGNLIFSQDVFELGDPTSGSVEMKEHLWRMLEL
GNSTNPADYFPFMGRFDLFGQRRDVAECLRQIYKIWGVILEERRRSGSKQNNDFVDVLLNAGIDDKKINA
LLMELFGAGTETSASTIEWAIAELTKNPHVTKKLRNELLNVVGKATVKESDIPRLPYLQAFVKEVLRLHP
PTPLLLPRRALETCQVMNYTIPKECQIMVNAWAIGRDPKTWTDPLTFSPERFLNSSVDYKGNDFELIPFG
GGRRICPGVPLATQFIHLIVATLVQNFEWTLPKEMDAKDLTMEEKFGLTLQKDPPLLIVPTARI
>MAQCYP80-2
MDLKTAFLYLIPIVLVYYILFKPKHKNLPPGPKPWPLIGNIPVLFGSGKPLHVTLTDMARTHGPIITLWM
GTQPTAIASTAESAMEILKTHDRLFSGRAVRMSFRLKHHIKYSLVWADCSEYWKLLRKIVRTEIFSPKML
QVQEHVREKKMEEMVEFLKGKEGEVLKLSPLVFGTLLNVLGNVVYSKDVFGYFGDDGDQMQTLMKEMLMI
GAAPNVAEFWPILEGLDLQGLKKKCHERFTEVMKMWKGTVEERKVNRNEEPKDMLDVLLANDFNDDQINA
LFLETFGPGSETSSATIEWAMAELIKNPKIIAKVREELDQVVGKSLVKESHLPNLPYLQACIKETMRLHP
AAPFLLPHRSVEACTVMGYTIPKDIQVLVNAYAIGRDPKTWEDPTTFKPERFMESDIDYYGKHFEFIPFG
TGRRQCVGMPLATRTIPLIVSNLVHAIDWSLPDGKNPEELVMNETLSLSLALDPTLTVIPKARV
>MCACYP80-1
MALLALFLIFALPILLLYFLKPSSPKNLPPGPFSWPLLGTLGLKSNEKQAHVVLTNLAQTYGPLMLLKLG
VKPVIVASTHVAAMEILKTQDHVLSGRCPPHSVQVEDYIEASVVWADRNEHWKMVRKICRTELFSTKMLE
SQASIREERVSELIAFLRRREGEVVKISDVIFGCMINVLGSVIFNQSVYDFEGKKDTGMKGMIRELMILA
ATPNLPDFYPIFDRFDIQGLRSTTSACWKRMSESWAGIVKERRASRDHSRNDFLDVLIQANFTDPQIDAL
FLEIFAPGSDSTTTAMEWAMTELMRNPEKRLNVQNELKTVIGNRQVVKESDLPNLPYLHACVKESLRLHP
PVTFLLPHKATETCQVMNYTIPKGTQLMVNAYAIGRDPKTWDDPLCFKPERFLNSEVDYQGNDFSLIPFG
SGRRMCIGMPLASRVVRLIIASLIHNFDWSLPGGIKPSELDMQEMFELVLQKHVPLSIIPNARA
>MCACYP80-2
MEIVSTVAIGFIFFIIFIIFFFSNQRSTKGLPPGPKPWPIVGNLLQLGDQPHAQFAQLSQTYGPLFSLKL
GSQTVVVASSPSAATQVLKTHDRVLSGRYVFQSFRIDRHVNNSIVWSECNDNWKLLRKVCRTEVFSPKMI
ESQAHLREAKAIEMVEFLRGREGQVLKIAEVVFGTLVNIFGNLIFSQDVFDLADPTSGSAEMKEHIWRML

ELGNSTNPADYFPIMGKLDLFGQRRAVAECLQQIYDVWGAMLKERRATKGTETNNDFVNVLLEAGLDDQR
INSLLMELFAAGTETSASTIEWAIAELTKNPQIMAKIHSELESVVGKERVKESHIPHLPYLQAFVKETLR
LHPATPMLLPRRALETCNVMGYTIPKDCQIMVNAWAIARDPKVWKDPLKFSPERFLNSSLDYKGNDFEFI
PFGGGRRICPGLPLATQFISLIVGTLVQNMDWSLPNGMDPTELGMEEKFGLTLQKEPPLLIVPKSRDFLN
ETRG
>MCACYP80-3
MDLAVLLSLFVPAILIYLLLKPKNVPPGPRSWPLLGNLLTVLRSHVPLHITLTDLARTHGPLMLVTLGTQ
PTVLASTSEAAMEILKTHDRALSGRHIRMSFRLKEMNKHSLVWSNCTDTWKLLRKIARTEIFSPKMLQIQ
EHVREQKVLELVEFLRGREYLGKAVKISQFVFGTLLNIIGNVVFSKDVFGFSCDEGGDEIGMQSLIRELL
MIGASPNIAEFYPILEGLDLQGLKRRCKDRVDRVNKLWEGTVKERRLKRSGESKDMLDVLLDNGFDDVQI
NVLFLETFGPGSETSSATIEWVMAELIKNPDKLAKVRTELEQVVGLHSQVKESHLPHLHYLQACVKETMR
LHPAAPFLLPHRAVETCRVMGYTIPKDCQVLVNAYAIGRDPNIWKDPSRFKPERFLESSVDYNGNHFEFI
PFGSGRRICIGMPLATRTVPLIVSSLVHNFDWSLPDGKRPEELVMNEMLSLSLAIDPSLSIIPKVRG
>MCACYP80-4
MALLALFLLFALPILLLYFLKTSSNPKNLPPGPFSWPLIGTLVMKLNEKRPHVVLTNLARTYGPLMLLTF
GIEPIAVCSTPEAAMEMFKTQDRVMSGRYVPHSVQVKGYIEHSMVWADCNEYWKMVRKIYRTELFSTKML
EAQVSVREEKVKELMAFIKRKEGEVVKITDVVYGCILNILGSVIFNQNVYDFDGKTDNDAGMKGMVRQLM
TLAGIPNFPDFYPIFGRFDPQGLKKKTTECVKRMNESWAGIVKERRASKDHSGNDFLDVLIQANFTDPQI
DSLLLEIFGPGSDSSTSTIEWAMADLMRNPEKLLKVQDELETVIGRNREVKESDLPNLPYLHACVKETLR
LHPPVTFLLPHRATETCQMMNYTVPKGTQTTVNTYAIGRDPKAWEDPLCFKPERFLNSEVDYQGNDFHYI
PFGAGRRICPAVSLASRVSRLIIASFIHDFDWSLPNGMQPSELDMEAKFGLVLWKDDPLCIIPQNQSPI
>MCACYP80-5
MDSVLAILYLSIFFLTILLVKLFQHKSHKSNQLPPGPRPWPILGNLVSILGSKTPPHITFANLARAHGGL
MLLWLGQKPLVVVSDKEAAVEVLKTHDRVLSGRSITVSFRFREKTEYSLVWADCNDYWKLLRRILRTEIF
SPKMLKIQERVREQKVTELMELMRSSEGKEVTIRPLVFGTILNILGNAVFSKDMFELGGRGDKVGLEQLI
RELLTIGSTPNIAAFFPIFEKLDPQRLKKRTWERLVKIDKLWESIVKERRQQERSEECKDMLDVLLANDF
TDPQIDNIFLETFGPGSESSSATVEWVMSELIKNPQTFAKLRQELDNEFGQSKVITGSRLTNLPFLSAVI
KETMRLHPAIPFMLPHRAVETCEVMGYTIPKDMEIQLNAYAIGRDPKAWKDPNTFRPERFLESDIDYQGN
HFELIPFGAGRRVCPGLPLAVKNTPLIVSSLVHGFDWSLPDGLTHDQLQMNEVLSVALTKDPSLCLIPKV
RIWAS
>MCACYP80-6
SHALRLLLLLLLLFLFFFRDSSSKNLPPGPPPFPIIGNLHQLGSKPHSTLAQLAQTYGPLLSLRFGSQLV
VIASSPAAASEVLKAHDNVLSGRHIIYNARSKNYVEHSMVWAPECNEAWKNLRRICRLELFSPKAMEARA
GVRELKVEEMVGVLRGKVGEVVKVSELVFGTIFNVLGGLIFSKDVFDMRREDGVVVGDLKGHLCKMLELG
SAVSLADCFPKLGWMTGGRKVSEECRRNVFGSWEEIIRERRELSSNYGVGLGDGCDDDDDDGAKKDFLSS
LIKAGFSNDQINTLLLDIFGAAADTTTSTIEWAMVEIMKNPHVLHKLQSELQLILMETSSKKNNIITEAD
LSHLPYLQATVKETLRLHPPTPLLLPRRALQTCKVMNYTVPKNSQILVNAWAIGRDTNVWENALKFWPER
FLEDYKLGNNSFNFVPFGGGRRICPGMPLAAELTPLILGSLFHNFDWSLPMGMTPYDLTTEEKFGLTLQK
DPPLLLVPKERH
>NDOCYP80-1
MDLAILIFSFILLLFVFLVLNDHLGRKGLPPGPRPWPIIGNLLQLGEKPHAEFAKLAQKYGDLFTLRLGS
QTVVVASSPAAAAEILKTHDRILSGRYVFQSFRVKEHVENSIVWCQCNDNWKMLRKVCRTELFTPKMIES
QAHVREVKALEMVKFLKTKQGEVVKVAEVVFGTLVNIFGNLIFSQDVFDLADPTSGSVEMKEQLWRMLEL
GNTTNPADYFPFMGRFDLFGQRRAVAECLQQIYDIWGVMLKERRANSTRNVNNDFVNVLLSAGLDDQKIN
ALLMELFGAGTETTSSTIEWAIAELTKNPRIVRKIQQELLNVVGEAATATVKESHIPHLPYLQAFVKETL
RLHPPTPLLLPRRALETCQVMNYTIPKECQIMVNAWAIGRDPRTWNDALVFTPERFLNSNVDYKGNDFEL
IPFGGGRRICPGIPLATQFIHLIVANIVHNFEWSLPNGMDVGKLTMEDKFGLTLQKDPPLYIVPKARTRY
DLEHILPSINKIAGLNNISIIETTDLGGTDLFVNICIIL
>NDOCYP80-2
MVVPFVLAFFVLFIAPFLLLFLLKPSPKNLPPGPFAWPLIGTILPNEKQPHVELTNLAQTYGPIMLLKFG

VEHVVVVSTNEAAMQVLKTHDLVLSGRYAPNSVKIKGYIEYSMVWNDCNEYWKMVRKIWKTELLSTKMLD
AQACVREEKVMELVDFLRGKRGHVVKFADLIYGCLLNILGAIVFSQNVYDFDDTSNNELGMKAMIRELMV
LAANPNNLADLYPMLGGSDFQGLRKATAACVKRMNELWGSIVRERRKTNAHFKNDFLDVLIKADFSDPQI
DAMFLEIFGPGSDTSTSTIEWAMAELLRSPEKLKNVRKELNNVVLGTKQVKESDLPNLPYLNAVVKETLR
LHPPATFLLPHRAIEECQVMGYTIPKGCQLMVNTHAIGRDSKTWNDPLSFNPERFLLDTEIDYQSSTDFK
YIPFGAGRRICPGLSLAARVVPLVLASLINTFDWSLPNGMDPSELDMEEKFGAALWKEVPLLIIPKVQ
>NDOCYP80-3
MELTTSLLCFIPIILLYILFKPSNKNLPPGPKPWPLIGSIPALFGSGSPLHVTLADMARTHGPIMTLWLG
TTPTVVASTPESAQEILKTHDKHFSGRAIRMSFRLKHHIKYSLVWADCSEYWKLLRKIVRTEIFSPKMLQ
VQEHVREAKVAELVEFLKGKEGQVVRLSQLVFGTLLNILGNVVFSKDVFRYADGEKDKMQKLIKEMLMIG
AEPNLAEFWPILEELDLQGLKKRCNDRFLEVMKMWEGTVEERKVKRNEEAKDMLDVLLANDFNDAQINAL
FLETFGPGSETSSATIEWALAELVKNPKILAKVREELDRVVGESTVKESHLPYLPYLQACIKETMRLHPA
APFLLPHRSVEECNVMGYTVPKDYGILVNAYAIGRDPNSWKDPTTFKPERFIESDIDYYGKHFQFLPFGS
GRRQCVGMPLATRTIPLIVSNLVHRIDWSLPDGKPVEELVMNETLSLSLALDPTLTVVPKVRV
>NDOCYP80-4
MFMKIMEETIAMEGINFLFLLLLLVLVLVLLCFIYKYITSPTLPPGPFSWPIIGNILAIGKRPHVSLAQL
AQAHGPLFSLRLGAQLVVVGSSPAAAHEILKVHDRLLSARSIPNAAPAKNPAYTYCSVGWADCTDQWKYL
RNICGSELFSTKAIESQSKLREDKVIELVEFLGTKEGQVVNIGEVVFATAFNMLSNILVSKDFINLEGER
GGEGMKGIVRTYVEVGITPNIADFYPILGVFDLQGLKRKSMESFVKICGFWEGIIKERRESKDGDVSMRQ
RDFLDYLLSIGFNNDQINHLLLELFLAGVDTTTPTIEWAMAELIKNQKTMKNVRDELEREINKNCVNESD
LVRLPYLHSCIKETLRLHHPGPLSIPRRALETCKVMNYTIPKGSQVFVNLWAIGRDPMTWNDPLVFKPER
FLDASVESNSSGFGWIPFGAGRRSCPGQPMANKQVPLIIASLIKRFDWSLPHDMNPSELNMDETFGLTLQ
REQPLILIPKVKK
>NDOCYP80-5
MDLTSAILIIPFFAIPFILMAFKLKHKNLPPSPPGWPILGNLPTLFSHGNEPLHITLTNMAPTYGPLLLL
RLCQKRIVVASTTEAAMEILKTHDRALSGRVIPSSFRIDYHIKHSIVWSDCNDTWKNIRKILRTEIFAPK
MLQVQEKIREGKVRDMMNFIRGKQGNVVNVRQWVFGCLLNILGQAVCSKDVFVYSDDGDKVGMQTLLRDM
LINSGTPNIGEYFPILDPLDLQWLRRQCNQLFRQGVKLWEGTVKERRLCRNEESMDMLDVLLANDFNDDQ
INALFLETFGPGSETSSSTIEWALSELIRNPDKMAKLRKELDQIVGKSTVKESHLPHLPYLQACIKETMR
LYPSAPFLLPHKAVETCQVMGYTIPKGYGILVNVYAIGRDPKLWKDPLTFTPERFLECDDMNYNGTNFQF
LPFGAGRRICVGWPLAERTIPLILSSLVHGFDWDLPDGKHHEDMEMNQVLHISLNKDPPLAVIPKMKA
>NDOCYP80-6
MLLKFGVEPVVVASTNEAAMQVLKTHDLVSGRFAPNSVQIKGYIEHSMVWNDCNEYWKKVRKIWKTQMFS
TKMLDAQAFVREEKVMELVDFLRGKQGHVVKFADLIFGCILNILGAVIFSQNVYDFDDTSDNELGMKGMI
RQLMVLAAIPNLADLYPILGSSDFQGLRKASAACVKRMNEYWGSIVKERRKKNCHLKNDFLDVLLKADFS
DPQIDAMLLEIFGPGSDTSTSTIEWAMAELLRSPEKLKTVRKELNNVVLGTKQLKESDLPNLPYLNAVVK
ETLRLHPPVTFLLPHQAIEECQVMGYTIPRGCPLMVNTYAIGRDPKTWKDPLSFNPERFLLDTEIDYQSG
NDFKYIPFGAGRRICPGLSLAARVVPFVLASLINTFEWSLPNGMDPSELDMKDKFGLALLKEVPLLITTL
FFLTSMAGISTSSTKRCAVVTGANKGIGLEICRQLPSNGVLVVLTARDEKRGVEAVENLRQCGLSDVVFH
QLDLMNSTSIASLAEFIQTQFGKLDILVNNAAIHGTIIDNDALKAQSHGNAWSALTGAMVQTYELAEECI
NTNYHGTKGVTEALLPLLQLSNSARIVNVSSKIGQLENISNEWAKEMLSNVDNLTEEKIDDVLNEFLKDY
KEGLLEIKGWPIAPSAYRISKAAVNAYTRILAKKFPMISINCCTPGFVKTDMSAHIGRLSVEDGAKAVVM
IALLPDGGPSGLFFVQKEVASF
>NDOCYP80-7
KSWKKPLQRKVSISLFLLLLVLVLLCFIYKYITSPTLPPGPFSWPIIGNILTIGKKPHISLAQLAQAYGP
LISLRLGTQLVVVGSSPAAAMEILKVHDRLLSGRSIPDVIPAKNPAFTKCSLAWNDCTDQWKYLRTICRS
ELFCIRAIESQSKLREDKVIELVEYLGTKEGQVVNFGEVVFGIVFNMLSNILMSKDFINLEEEGEGEGMK
GLVRTLVELGTIPNLADLFPLLSVMDLQGLKKKSTEAFVKICGFWEGIIKERREGRDKDVLIRQRDFLDY
LLSIGFNNDQINRLLVELLLAGADTTTSTIKWTMVELMKNQDAMKKVCIELEREINENRVKESDLHRLPY
LNSCTKETLRLHPPAPLLLPHRALETCKVMNYTIPKDTQVFVNLWAIGRDPMTWDDPLVFKPERFLDSNV

ESKLNDFGWIPFGAGRRICPGQPMAMKQAVLILASLINHFEWSLPCDISSSTLNMDEKFGITLQKEQPLI
LIPIIK
>NSACYP80-1
MEFFSIAIVSSFFFLFLIFILRDSRPKNLPPGPRPSPIVGNLLQLGDKPHSEFAKLSQTYGELFSLKLGT
QTVVVASSPAAATEILKTHDKNLSSRYVFQSFRVKEHVANSIVWSDCSENWKLLRKICRTELFTPKMIES
QAYIREAKALDMVKFLKKKEGEVVKIVEVVFGTLVNIFGNLIFSKDVFDLEDPNGGSAELKQHLWKLLEM
GNSTNPADYFPFLGKFDLFGQRRAVAEVLHQIYDVWGVMLKERRAMNGTENDFVSVLLSSGLDDQKINSL
LMELFGAGTETSASTIEWAIAELTKNPSIASKLQSELLSIVGDRAVKESDIPSLPYLQAFVKETLRLHPA
TPLLLPRRAPNTCKVMKYTIPKDCQIMVNAWAIGRDPKTWDDPLSFKPERFLDSNLDFKGNDFELIPFGG
GRRICPGLPLASQFISLFIATLVQNFDWSLPQGVSSNDLSMDEKFGLTLQKDPPLLIVPKLRISSV
>NSACYP80-2
MDVQVALFSIIPAILAFLLISKFKNKNFPPGPPSWPLIGSMPILFNSVTPLHITLTNMARTYGPMMTLWL
GTQPTVIASTPESAMEILKTHDRACSGRHIRMSFRLKHHIKYSLVWADCTDYWKLLRKIARTEIFAPKML
QAQSHAREEKVGELIEYLGRKQGQAVKINQFVFGTLLNILGSVVFSKNVFEFSDDDGDKESMQNLIKQML
MIGAAPNLAEFYPFLETLDLQGLKRRCDDRFTQVMKFWEGTVKERRERRNEESKDMLDVLLANDFNDSQI
NALFLETFGPGSETSSATIEWTMAELIKNPKEMAKVRKELDNVVGRSSVKESHLPELPYLQACIKETMRL
HPAAPFLLPRRAVESCQIMGYTIPKGCQILVNAYAIGRDPKAWKEPDRFRPERFLETEVDYHGGHFQYIP
FGSGRRTCVGMALATRTLPLIVGSLVNTYDWGLPDGKKPEELEMKEMLSLSLAIDPSLLVVPTLRA
>NSACYP80-3
MEINLYYYLLLLVLPLIFLIIKHIKSATNLPPGPFPWPVIGNILSMGTKPHVSLAQLAKSYGPVMSMRLG
TQILIVGSSPAAATEILKTNDRVLSARKVVHALPASPDRLHFSVTWSDCTDQWKYLRTLIRTELFSAKVI
ESQGQMRERKVSEVVEFLRTKEGKPVKIGVIVFNTIFNMLSNLLVSRDLIDLHQENEDEGLKGFVRKFVE
LCSCPNLADYYTMLSGLDLQGLNKKARKMFQEISMIWEVIIKERRDAQSHGNNLSREPDLLDTMLDRKLT
NDHISYLFLELFLAGTDPSTSTVEWAMAELMKNPNALKIVRSELATEINGSVIKESDLSRLPYLQSVVKE
TLRLHPAAPFLLPHRALETCKVMNYTVPKDSQVFVNIWAIGRDPNVWEEPMSFKPERFLNQSHNDLNSTN
IPFIPFGAGRRICPGQPMVAKQVPLVLASLINCFEWSLPHGVHPTDLDMEEAFGIVLQKDKPLILIPQVI
LQ
>NSACYP80-4
MLEMDYYYYLLLLVLPLVFLIWKHIKPPTNLPPGPFPWPVIGNILNLGNKPHVSLAQLAETYGPVMSLRL
GTKLLIVASSPAAATEILKTHDRIFSGRTIPHAIPAYPDRIHLSMGWADCTDQWKFLRTLCRTELFSAKA
IQSQANIREDKMKELVMFLATKEGQSVKIEEVVFNNIFNTLSNLFLSKDLMSLQEGDQLRNFVRTFIQLI
FTPNLADYYSILSGLDLQGLNKKCKEMIEKICGVWGVIIRERRQEDKDSGVTRPRDFSDSLIHAGLTDEQ
INYLFMELFFAGADTSTSTIEWVMAELIKNPQVMRKVCGELESRKKETVIINDSDLPYLPYLHATVNETL
RLHPPAPFLIPHRAIETCTIMNYTIPKDAQVFVNVWALGRDSKTWEDPMSFRPERFLESGIDINSGDFRF
IPFGGGRRICPGLPLAAKQVPLLVANLINCFDWSLPLDMEPAHLCMDEKFGITLQMEKPLVLVPRKKKW
>NSACYP80-5
LYYYLLLLVLPLIFLIIKHIKSATNLPPGPFPWPIVGNILNMGNKPHVSLSQLAETHGPLMSLRLGTQLL
IVASSPSAATEILKTQDGILSGRTVPHAIPVSPDRLHLSLGWADCTAQWKFLRTLCRTELFSAKAIQSQA
NVRDDKVKELVMFLATKEGQSVKIAEVVFNNIFNTLSNLLLSNDLVSLQEGDQLRNFVRTFIQLISTPNL
ADYYSILSGLDLQGLNKKAREIFEKTCGVWEVIIRERRKEGESGGSRQRDFLDSLINSGLTNDQINYLLM
ELFSAGTDTTTSTVEWAMAELIKNPQVMRKARLELENEIKETAIIKDSDLPCLPYFRAIVNETLRLHPPA
PLLLPHRATETCKLMNYIIPKDAQVLVNAWKIGRDSKTWEDPMSFRPERFLESGMDLSSSNFKFIPFGAG
RRVCPGLPVAARQVPILVANLINFFDWSLPLDMEPGHLCMDEKFSITLQMEKPLVLIPSVKKR
>PBRCYP80-1
MEIVTAALIAIVITTFLYLIFRDSSPKGLPPGPKPWPIVGNLLQLGEKPHSQFAELAKTYGDLFTLKLGS
ETVVVASTPLAASEILKTHDRVLSGRYVFQSFRVKEHVENSIVWSECNDTWKKLRKVCRAELFTQKMIES
QAEIRESKAMEMVEFLKRNQGSEVKIVEVVFGTLVNIFGNLIFSQNIFKLGDESSGSVEMKEHLWRMLEL
GNSTNPADYFPFLGRFDLFGQRKDVADCLQGIYSVWGAMLKERKIAKLHNNSKKNDFVEILLDSGLDDQQ
INALLMEIFGAGTETSASTIEWALSELTKNPEVTANMRSELLSVVGKRPVKESDIPNMPYLQAFVKETLR
LHPATPLLLPRRALETCKVLNYTIPKECQIMVNAWGIGRDPKRWTDPLKFAPERFLNSSIDFKGNDFELI

PFGAGRRICPGVPLATQFISLIVPTLVQNFDWGLPKGMDPSQLIMEEKFGLTLQKEPPLYIVPKNRD
>PBRCYP80-2
MEIVTLAALIAIVITSFFYLILRDSSPKGLPPGPKPWPIVGNLLQLGEKPHSQFAQLAGTYGDLFTLKLG
TQTVVVASTPLAASEILKTHDRVLSGRYVFQSFRIKNHVQNCMVWSDCNETWKSLRKVCRTELFTRKMIE
SQAEVRECKAMEMVEYLRKHQGNEVKIVEVIFATLVNIFGNLIFSQNIFKLSDENSGSVEMKEQIWRLLE
LGNSTNPADYFPFLGSLDLLGQRKDVADSLQEIYGVWGPILTEKKLAKQHNTNTTNDFVDLLLDSGLNDQ
QINAFLMEIFSAGTETSATTIEWALAELIKNPELTANIRSELLSVLGKRTVKESDIPNLPYLQAFVKETL
RLHPATPLLLPRRALETCQVLNYTIPKGCQIMVNAWGIGRDPKTWTDPLKFAPERFLSSSIDYKGNDFEF
IPYGAGRRICPGMPLATQFISLIVPTLVQNFDLGLPKGMDPSELIMEEKFGLMLEKEPPLYIVPKTRE
>PBRCYP80-3
MMALENSIRERKRENKIKMDIVTLAALIAIVFTIFFFLILRDSSPKGLPPGPKPWPIVGNLLQLSDKPHS
HFAQLAKTYGDLFTIKLGTETIVVASTSIAATEILKTHDRILSGRYVFQSFRVKGQVKLSMTWSDCNDTW
NKLRRICKVELFSQKMIESQAEIRESTALEMVEYLKKKEGSEVKMSDVIFGTVMNMFGKSIFSQNIFELG
DENSGSLNMKERIRRLMELGNTKNPADYFPFLGSWDLLGQRKEVANCLQRVYDVWGVVLKERKIFNMQHN
SKKKNDFVDVLLDAGLEDGQINSLIMEIITGTETSGSTIEWAVAELTKNPQVAAKMRSELLSVVGQRPIK
EADIPNMPYLQAFSKETLRLHPPTPLLIPRRALETCKILNYTIPKDCQIMVNAWGIGRDPKTWTDPLKFS
PERFLNSSIDFKGNDFGYIPFGAGRRICPGMPVAIQFLSLIVPTLVQNFDWGLPNETDPTQLIMEEKFAL
TLQKEPPLYIVPRTRDPSA
>SCACYP80-1
MEIVTVALIAVVISSILYLLFRDSSPKGLPPGPKPWPIVGNLLQLSDKPHAQFAELAQTYGDLFTLRMGT
QTVVVASTPSAATEILKTHDRILSGRYVFQSFRVKGHVENSIVWSECNDTWKNLRKVCRTELFTQKMIES
QAHVRERKAWEMVEYLKKKEGEEVKIVEVIFGTLVNIFGNLIFSENIFELGDPSSGSVEMKEYLWRMLEL
GNSTNPADYFPIMGKFDLFGQRKAVAECLQGIYDVWGAMLKERKAAKKAGGSEGKNDFVNILLDSGLNDQ
QINALLMELFGAGTETSASTIEWAITELTKNPKVTAKIRSELLSVVGQSTVKESDIPNLPYLQAFTKETL
RLHPPTPLLLPRRALETCQVMNYTIPKECQIMVNAWAIGRDPKTWTDPLNFSPERFLNSSVDFKGNDFEL
IPFGGGRRICPGVPLATQFISLIVGTLVQNFDWDLPNGMSPRDLKMEEKFGLTLQKEPPLCIVPKSRA
>SDICYP80-1
MEIVTVALIAVVISSILYLTFRDTSPKGLPPGPKPWPIVGNLLQLSDKPHAQFAELAQTYGDLFTLRMGT
QTVVVASTPKAAAEVLKTHDRILSGRYVFQSFRVKEHVKNSIVWSECNDTWKSLRKVCRTELFTQKMIES
QAEVRERKAWEMVEYLKKKQGEEVKIVEVIFGTLVNIFGNLIFSQNIFDLGDPSSGSVELKEHLWRMLEL
GNSTNPADYFPIMGRFDLFGQRKEVEDCLQGIYDVWGAMLKKRKAAKEAGKSEGRNDFVEILLDSGLNDQ
QINSLLMELFGAGTETSASTIEWAITELTKNPNVTAKMRSELETVVGHNTVKESDIPNLPYLQAFVKESL
RLHPPTPLLLPRRALETCRVLNYTIPKECQIMVNAWAIGRDPKIWTDPLTFSPERFLNSSIDFKGTDFEL
IPFGGGRRICPGIPLATQFIGLIAATLVQSFEWDLPNGMSPKDLNMEEKFGLTLQKEPPLYIIPKSRD
>TCOCYP80-1
MEIITLSIGFIFFIIFIFYFFNNQSSKGLPPGPKPWPIVGNLLQLGEKPHAQFAQLSQEFGDLFTLRLGS
QTVVVASSPAAATQILKTHDRILSGRYVFQSFRIDKHVNNSIVWSECNDNWKMLRKVCRTEIFTAKMIES
QAHVREAKAMEMVKFLRHREGEVVKIAEVVFGTLVNIFGNLIFSQDVFDLSDPSSGSAEMKEHIWRMLEL
GNSTNPADYFPIMGKLDLFGQRRAVEECLQQIYDIWGVMLKERRARKGSENTHDFVNVLLEAGLGDQCIN
SLLMELFAAGTETSSSTIEWAIAELTKNPRITTKLRQELRDVIGEERVKESHIPSLPYLQAFVKETLRLH
PATPMLLPRRALETCKVMGYVIPKDCQIMVNAWAIGRDPKVWKDPLTFWPERFLNSSLDYKGNYFEFIPF
GGGRRICPGFPLASQFISIIVATLVQNMDWCLPNGTDPSELVMEEKFGLTLQKEPSLLLVFKSRDLK
>TCOCYP80-2
MDPTVALILCLVIPVILLISFLYKPRNLPPGPHAWPLVGSLPAVLSSGAPLHITLTNLARTHGPLMLVTL
GTQPTVLASTSEAAMEVLKTHDRALSGRHIRMSFRLKEMNKCSLVWSNCTDYWKLLRKIARTEIFSPRML
QIQEHVRERKVGELVEFLRGRKEGKAVKISEFVFGTLLNIIGNVVFSKDVFGFDDEGGDKIGMQSLIREL
LMIGASPNVAEFYPILEGLDLQGLKRRCRDRVNRVNKLWETTVMERRKKRSVAEGWESKDMLDVLLDNNF
DDVQINVLFLEMFGPGSETSSATIEWVLAELIKNPDKMAKVQKELEKLVGNKGSQVKEAHLPDLPYLQAC
VKETMRLHPAAPFLLPHRAVETCQVLGYTIPKDYQLLVNAYAIGRDPNTWKDPTSFCPERFLDSSVDFYG
NHFEFIPFGSGRRICIGMPLATRTVPLIVASLVHNFDWSLPDGKRPEELVMNEMLSLSLSIDPSLSVVPK

TRA
>XSICYP80-1
MEVLSIAIVSFLFLLFLFFILRDSRQKNLPPGPRPSPIVGNLLQLGDKPHAEFAKLAQKYGELFSLKLGS
QTVVVASSPAAAAEILKTHDKILSGRYVFQSFRVKEHVENSIVWSECNENWKLLRKVCRTELFSPKMIES
QAGIREAKAREMVKFLRSKEGEVVKIVEVVFGTLVNIFGNLIFSKDVFDLEDPTGGSAELKEHLWKLLDM
GNSTNPADYFPIMGKFDLFGQRRAVAEVLQQIYDVWGVMLKERRGRKVSESNNDFVNVLINAGLDDQKIN
ALLMEMFGAGTETSASTIEWAITELTKKPPVASKIRLELVNVVGDNTVKESDLPHLPYLQAFVKETLRLH
PPTPLLLPRRALETCTVMNYTIPKDCQIMVNAWAIGRDPKTWDDPLNFKPERFLSSDVDYKGNDFELIPF
GGGRRICPGLPLASQFISLIVATLVQNFEWSLPQGMSTSELSMDEKFGLTLQKDPPLLIVLKARASNDLS
FI
>XSICYP80-2
MEELIVVLRETNLLYYISLIALPLLFLILKQIKSPSNLPPGPFPWPIVGNILNMGSKPHISLAQLAKSHG
PLISLRLGTQLVVVGSSQAAAKEILKTHDRILSARCVPRVIPVYPDQLHLSMAWVECNDQWRYLRTLCRT
ELFSAKMIESQASVRDKKTNELVEFLANNEGKLVKIGDVVFATVFNMLSNIFVSKDFISLEEESEDGGMK
GLLRRFMELGSAPNLADFYTILSGLDLQGITKKSKEMLGQVNGAWGVITKQRRADKGNDVLRQRDFLDSL
LDSELDDDQINYLLQELFLAGADTSTSTVEWAMVELIKNPKAMRKVCAELERETSGHIVKESDLPRLPYL
HFTVNETLRLHPPAPFLLPHRALETCKVMNYTIPKDCQVLVNAWAIGRDPNTWEDPLSFKPERFLDSGMD
LNSGNFKFIPFGAGRRICPGLPMAAKQVTLILACLINCFEWSLPHSMLPSELDMDDKFGITLHKEKPLLL
IPKVRKA
>XSICYP80-3
MDLQIALFSLIPVILVFILLLKPKYKNLPPGPHPWPFIGSLPILFTNTEVPLHITLANMARTHGPLMILW
LGTQPTVMASTAEAAMEILKTHDRIFSARHIRMSFRLKHHIKYSLVWSDCTDYWKLLRKIVRTEIFSPKM
LQAQSHVREQKVAELIEFLRSKEGQVVKISQFVFGTLLNILGNVVFSKDVFVYSDESDKGGIQNLIREML
MIGAEPNVAEFYPILEELDLQGLKKKCDDRFIRVMKMWEGTVTERKANRNEESKDMLDVLLANDFNDAQI
NALFLETFGPGSETSSATIEWVIAELIKSPKEMAKVRKELDEVVGTSTIKESDLPQLPYLQACIKEAMRL
HPAAPFLLPRRAAETCEVMGYTIPKGSQVLVNAYAIGRDPKSWKDPTIFRPERFLESDVDFHGAHYQFIP
FGSGRRTCVGMPLATRTIPLIVGSLVHTYDLGLPGGKRHQELEMNEMLSLSLAIDPSLCVVPKARA
>BSTCYP80A1
MDYIVGFVSISLVALLYFLLFKPKHTNLPPSPPAWPIVGHLPDLISKNSPPFLDYMSNIAQKYGPLIHLK
FGLHSSIFASTKEAAMEVLQTNDKVLSGRQPLPCFRIKPHIDYSILWSDSNSYWKKGRKILHTEIFSQKM
LQAQEKNRERVAGNLVNFIMTKVGDVVELRSWLFGCALNVLGHVVFSKDVFEYSDQSDEVGMDKLIHGML
MTGGDFDVASYFPVLARFDLHGLKRKMDEQFKLLIKIWEGEVLARRANRNPEPKDMLDVLIANDFNEHQI
NAMFMETFGPGSDTNSNIIEWALAQLIKNPDKLAKLREELDRVVGRSSTVKESHFSELPYLQACVKETMR
LYPPISIMIPHRCMETCQVMGYTIPKGMDVHVNAHAIGRDPKDWKDPLKFQPERFLDSDIEYNGKQFQFI
PFGSGRRICPGRPLAVRIIPLVLASLVHAFGWELPDGVPNEKLDMEELFTLSLCMAKPLRVIPKVRI
>CJACYP80G2
MDLQIALFSLIPVILVFILLLKPKYKNLPPGPHPWPLIGNLPILFTNTEVPLHITLANMARTHGPIMILW
LGTQPTVMASTAEAAMEILKTHDRIFSARHIRMSFRLKHHIKYSLVWSDCTDYWKLLRKIVRTEIFSPKM
LQAQSHVREQKVAELIDFLRSKEGQVVKISQFVFGTLLNILGNVVFSKDVFVYGDETDKGGIQNLIREML
MIGAEPNVAEFYPSLEELDLQGLKKKCDERFIRVMKMWEGTVKERKANRNEESKDMLDVLLANDFNDAQI
NALFLETFGPGSETSSATIEWVIAELIKSPKEMAKVRKELNEVVGTSTIKESDLPQLPYLQACIKEAMRL
HPAAPFLLPRRAAETCEVMGYTIPKNSQVLVNAYAIGRDPKSWKDPSTFWPERFLESDVDFHGAHYQFIP
FGSGRRTCVGMPLATRTIPLIVGSLVHNYDFGLPGGNRPEDLKMNEMLSLTLAIDPSLCVVPKARA
>ECACYP80B1
MGTETVVVASTSSAASEILKTHDRILSARYVFQSFRVKGHVENSIVWSDCTETWKNLRKVCRTELFTQKM
IESQAHVREKKCEEMVEYLMKKQGEEVKIVEVIFGTLVNIFGNLIFSQNIFELGDPNSGSSEFKEYLWRM
LELGNSTNPADYFPMLGKFDLFGQRKEVAECLKGIYAIWGAMLQERKLAKKVDGYKSKNDFVDVCLDSGL
NDYQINALLMELFGAGTETSASTIEWAMTELTKNPKITAKIRSEIQTVVGERSVKESDFPNLPYLEATVK
ETLRLHPPTPLLLPRRALETCTILNYTIPKDCQIMVNAWGIGRDPKTWTDPLTFSPERFLNSSVDFRGND
FSLIPFGAGRRICPGLPIANQFIALLVATFVQNLDWCLPNGMSVDHLIVEEKFGLTLQKEPPLFIVPKSR

V
>HNICYP80F1
MYIEDTSEIFTIFFTHILLPLLSFFIIRCVISSRKKLPLPPGPFPWPIIGHLFYLGNKPHVSLAKLANVH
GPHLMSIRLGGRLVIVASSPMATAEVLKTHDRLLSGRFVSHPMRVEGSYIRNLATETLEECDENWKKVRS
MYQIVLFSHKAVESQVNIREKKVMELVKFVASKEGELVNIKGIAFVTILNILSNSTISNDLVDFEGKGIG
EGMREWIRNYTKLEGVPQLADLFPILDGCTWDFQGTYKKLKDTFERISDVWRDIINKKRMEISNKYYEGE
DFADALIRNGFEDKQINALLMELYSAGTETTITTVEWTLVELLKNPEAMKRLRNEIKKELTTIDDREIMI
VKDSNLPNLPYLEACMKETLRLHPPAPLLFPHRAVQTCEVMGYRIPQDTQIIVNVWKMARDSEYWNDDPW
SFKPDRFLDSSTDYKGHDFEFIPFGSGRRICAGQSLALRMLPMIVGSLVHNFELILPNNMNPMEMNMDDI
IDVTMAKKDPLFIIPKIRNS
>PSOCYP80B1
MIESQAEVRESKAMEMVEYLKKNVGNEVKIAEVVFGTLVNIFGNLIFSQNIFKLGDESSGSVEMKEHLWR
MLELGNSTNPADYFPFLGKFDLFGQSKDVADCLQGIYSVWGAMLKESKIAKQHNNSKKNDFVEILLDSGL
DDQQINALLMEIFGAGTETSASTIEWALSELTKNPQVTANMRLELLSVVGKRPVKESDIPNMPYLQAFVK
ETLRLHPATPLLLPRRALETCKVLNYTIPKECQIMVNAWGIGRDPKRWTDPLKFSPERFLNSSIDFKGND
FELIPFGAGRRICPGVPLATQFISLIVSSLVQNFDWGLPKGMDPSQLIMEEKFGLTLQKEPPLYIVPKTR
D
>TFLCYP80B4
MEVLSAAMVSLFFIFLFIFFILCDSRNKDLPPGPRPSPIVGNLLQLGEKPHAEFAKLAEKYGELFTLKLG
SQTVVVASSPAAAAEILKTRDKILSGRYVFQSFRVYEHVLNSIVWSECNENWKLLRKVCRTELFSPKMIE
SQAYIREAKALDMVRFLRKKENQEVKIVEVVFNTLVNIFGNLIFSKDVFDLEDPTGGSAELKEHLWKLLD
MGNSTNPADYFPIMGKFDLFGQRREVAKVLKQIYDVWGVMLKERRSITGHRENDFANVLLNAGLDDQKIN
ALLMELFGAGTETTASTIEWAITELTNNLRVISKLRAELINVVGHKTVKESNIPHLPYLQAIVKETLRLH
PPTPLLLPRRALETCKVMNYTIPKECQIMVNAWAIGRDPKTWDDPLTFKPERFMNSTVDYKGNDFELIPF
GGGRRICPGLPLASQFLSLIVATLVQNFDWSFPQGMTPNEVPMDEKFGLPLQKDPPLLIVLKARTSMKLQ
D

CYP82

>HSACYP1A2
MALSQSVPFSATELLLASAIFCLVFWVLKGLRPRVPKGLKSPPEPWGWPLLGHVLTLGKNPHLALSRMSQ
RYGDVLQIRIGSTPVLVLSRLDTIRQALVRQGDDFKGRPDLYTSTLITDGQSLTFSTDSGPVWAARRRLA
QNALNTFSIASDPASSSSCYLEEHVSKEAKALISRLQELMAGPGHFDPYNQVVVSVANVIGAMCFGQHFP
ESSDEMLSLVKNTHEFVETASSGNPLDFFPILRYLPNPALQRFKAFNQRFLWFLQKTVQEHYQDFDKNSV
RDITGALFKHSKKGPRASGNLIPQEKIVNLVNDIFGAGFDTVTTAISWSLMYLVTKPEIQRKIQKELDTV
IGRERRPRLSDRPQLPYLEAFILETFRHSSFLPFTIPHSTTRDTTLNGFYIPKKCCVFVNQWQVNHDPEL
WEDPSEFRPERFLTADGTAINKPLSEKMMLFGMGKRRCIGEVLAKWEIFLFLAILLQQLEFSVPPGVKVD
LTPIYGLTMKHARCEHVQARRFSIN
>AMECYP82-1
MDNFLLQFQPISIAAFFVALVFLYWSYGRKSNKTLKPRAPEAAGGRPITGHLHLFYGEELTHRKLGSMAD
KYGPVFNIRFGSHKTLVVSNWEIVKECFTTNDRLFSNRPGTLAIKLMFYDTDSVGYAPYGNYWRELRKIS
TLKLLSNHRIETLKHLRTSEVESCFKQLYNQWEMKNENRVEVDKLGFASVRMDNWFGDLTFNVVARIVAG
KKNFAGGAVNGDIGAQRYKVAMDESFRLMLTFAYSDVIPSLKWLDKLRGLIRDMKRCGSEIDSIVASWVE
EHRLKRNSSENNNLDLEEDFIDVCLDIIESSSLPGDDPDTVIKSTCLDMILGGSDTTTVTLTWAISLILN
NPHVLKRAKEELNSVVGKDRRVEDSDIPHLTYIHAIIKETMRLYPAGPLIERRTMEDCEVGGYHVPAGTR
LLVNVWKMQRDGDVYKDDPLVFRPERFMTSNADVDLKGQHFELIPFGAGRRICPGVSFAVQLMHLVLARL
LHEFEITTIPSEPKVDMSESGGLICHKIKPLEVLIKPRLELN
>AMECYP82-2
MDFSSILLLLNINCFTTSLVTLLVLVMVFLYNNTRFTKPKGNKKMIKPPRLPGARPLLGHLHLFGPGELP
HKVLSTMADKYGPAFTIKFGKHTTLVVSDIHTIKECLTTNDILFSNRPSSIAFDLMTYADDSVAFAHYSP

YWRELRKISTLKLLSNNRLQSIKQLRLSEVNVCFKELYSLCNNNNKSGAPVLIDMKKWFKEVTTNIVIRV
IVGKQNFGSKIVRGEDQEAANYKKIMDELSRLAKLSMFSDYAPLLSLFDYFRGNVSAMKRNGKELNAMLQ
NWLEEHKRKKNSSISNDDEQDFMDLMLSIIDETKLYGRDADTFIKAISLSMIVGGIDTVVVTLTWILALI
MKNPFALKKAQEELDFFVGKERQVEDSDLKNLVYMNAIVKETLRLYPLSCILERDTKEDCEVGGYYVEGG
TRLLINAWKLQRDPNVWTDPSEFKPERFLNENADIDVGGQHYELIPFGAGRRVCPGVSFALQFMDLVLAR
LIHGYELGTLNGEDVDLTEKTEGQINFKATPLDLIVTPRLHPNLYDY
>BTHCYP82-1
MESIEFLVTLLLSLLALFCAYAWKRKNHTKDSKIKEPPQPAGAWPIIGHLHLISRGGLPHINMGAMADKY
GPVFMIRLGVNRAVIVSNTEIVKECFTTNDKFLLNRPVGLALNLMCYNNAMFGFCRYGPYWREIHKIVML
ELLSNSRLESLKHVWDSEISTSIEELYQLCQTQNKTHEGPVLVEMKDWFADMALNVSLRMIAGKRYFGAS
AGGNKDEARKCQKTIRDFFRLVGLFIVSDALPFLRWLDLGGHQKEMKRTFEDLDYMLQGWLDEHKLNRKS
GVNGDQDFMDVMLSILDDSKFPDYDVDTVNKATCFQLIIGGTDTTTVTLTWALSLMLNHPHVLKKAQDEL
DIQVGKHRQVEESDIKNLKYLEAVIKETLRMRPVGPLLGPRETIEDCTIGGYRVRAGTRVMVNAWKVHRD
PSVWSNPDDFKPERFLKKDIDFRDQNFEFIPFGSGRRACPGISFAVEVLPMALARLLHGFELKTQLGCKV
DMTEHAGLVHAKATPLEVLVSPRLSRELYVSS
>BTHCYP82-2
GSLALIAIIILSNIVKKSSKSLSSKGGKSAPVVHGAWPVIGHLHLLMGKELPHHALAKLADKYGPAFTLN
IGAYQELVISTRDLAKECFTTKDKFFLNRPSNKAMKILTYGEASVGFAPYSPLWVEMRKVTKSNLLSHQR
VLMQNRSRASEIDASFKQLYEQCNGKSGASVEMKNWFEEVMLNVVTRNVSGKRSFGTKARLGDAEAVKYK
RVIDETARFMGNLVISDMVPSLWWLDNMLGAESAMKKLAIELDSLLGGWVEEHRGKKLSDDEEGDFISLT
WEMIDQIQLHGLAPETFIKSMCVGVLLGGSDTTSVALTWALSLMLNNREVLKKAQEELDFHVGKDRKVDD
SDLKNLVYLQAIMKETMRIYQVGPLIEREAVEDCEVGGFQIKKGNRILVNLWKLQRDPEVWSDPSEFRPE
RFLEDKSKVDLKGQNAELMPFGSGRRMCPGVWFGVHSTSLVLARLIQGFEIETPLDAKVDMTETSSVTNY
KGSPLEVIARLPGSNLSFMICSYV
>CCHCYP82-1
MTPSISQPFELLLLLMTMESLLLQWQPTSIAVLLLAITIFLYTFTKSPPKTHKKLAPDATGGRPLMGHLH
LFNSNELTHRTLGSMADKYGPAFNIRFGSHKTLVVSTWEIVKECFTTNDRSFSNRPGTLYIKLMFYNEDS
VGYAPYGAYWRDLRKLSTLKLLSNHRLESLKHLRVSEMNECFKQLYEICNKGSKSVAVRMDNWFGDLTFN
VVARIVAGKRNFASISNDVGAMRYKNAMDEAFRLMTVFSFSDVIPSLGWLDNLRGLVGDMKKAGKEIDSV
VSGWIEEHREKRKRRSSGGNNRDGELEEEDFIDITLSILETSSLPGDDPDMTIKSTCLDMILGGSDTTTV
TMTWALSLLLNNAHALKRAREELDLHVGRDRQVEDSDINNLVYIQAIIKETMRLYPAGPLIERMTKEDCE
VGGFHIPAGTRLLVNLWKMQRDPNVWEDPLEFRPERFLTNNAEIDLKGQHFELIPFGTGRRICPGVSFAL
QLMHLALARLLHSFEVASPMDAKIDMTETAGLISHKVIPLEVLMTPRLDSKLYDY
>CCHCYP82-2
THTTEKNLPPEVPGALPVIGHLHKLGGRKGLLPYHVLGAMADKYGPAFTFQFGSNRTLVISNWEMVKECF
TSTNDKLFAYRPTALFNKEVFCSDHSFGFGPYGPFWREMRKVCSLHLLSNYRLELLKHSRKSEMDECFKE
LYQLWSASNISKDKQPAVLVVEMKKWFEDAMFNVASRVVVGKGSLKSHEKAGGSDNEVARNKYKKGMDEA
RRHMGTFVISDRIPFLWWIDYLRGIHSSMKRTAKDLHAIVETWLEEHRLHCHKRRVLQLDANHEVEDMAK
EEEKDFMDVLLTMADEGAIKIFDHDLDTVIKANCLDMVIAGTDTPTVMLTWTLSLLLNNPDVLKRVRDEL
DLHVGKERQVEESDVKNLVYLQAVFKETLRLYPAVPLNERLTMEDCNVGGYDIPAGTRLLINIWKVQRDP
NVWQDPSEFRPERFLSKEKSTIDVRGLDFELIPFGTGRRMCPGISFSLQLMHLGLARLIHGFELATPMDL
DVDMSANPGAFNYKSTDLQVLVSPRFHSKFYGC
>CCHCYP82-3
MDFLALQWFPASLTALLALAVLYNFWTKQRTYKNGKTSTKQAPVAAGAWPVLGHLHLFGGGELPHKMLAT
MADKYGPAFTMKFGTHQTLVVSNTKIVKECFTTNDTLFANRPSTTAFHLMTYDNESVAFTPYGPFWRELR
KISTLKLLSNHRLQAIKDVRASEVNVCFRELYSQWRINKSEPETSDHQGPILVDMKKWFEEVSDNVVLRV
IAGKQNFGSKIVHGDKEALHYKKIMDEILRLAAVSMLSDVAPLLGWLDMFQGHLSAMKRNAKEVDTMLCN
WLEEHRTKRLSSDHDGVEQDFIDVMLSIVEENKFSGHDNDTVIKATVLAMIMGGTDTTAVSLTWIVSLLM
NNRHVLRRAQDELDLHVGKDRQVDDSDLKNLVYLNAIVKETMRLYPLGALLERETKEDCEVGGFHVKGGT
RLLVNVWKLQRDPSFWTDPTEFKPERFLADKANIDVGGQHFELLPFGAGRRVCPGVSFALQFMHLVLARL

LHGFDLATPMNADVDLTESTEGHVNQKASPLNLLVTPRLHSKLYEY
>CCHCYP82-4
MEYTFSSASLFSSSSLQQWLSTLLLLISSITISLYLTRRGKSSPPMVPGAWPLIGHLHLLGGNDPLHIVL
GTMADKYGHTFAMLFGKHPSLVVSSSDIVKECFTSTNDKLFSHRHVPTGVKYMFYNNDSFGFAPYGPYWR
EMRKMNSLSLFSHHRVEMLNRIRTSQINIWFKNLYEMCTKEKSTSTSSDGVVVEMKSWFDEMFFNVLVNM
IVKPINDDEELVKKYREVATEAGRLLSSMAVSDMVPSLGWLDHLFGIVGKMKKTAKDMDAILTTWLEQHK
IKSQQLRRSNGGEGAADQTEEEEKEGKGLIGDLLSMQESALFGHDRDTVIKSACQALIMAGSDSTSATLT
WVLSLLLNHRDALRKAREELDQVVGKDRQVDDSDIKDLVYLQAILKETMRLYPAAPILERLAVEDCIVGG
FHVQAGTTLFVNVSKLQRDPNLWTDPLEFKPERFLTGCNADVDLKGQDYELVPFGSGRRSCPAVSFAVRV
MLLVLARFIHSFEVKTREEDGILDMTESSGHTNCRSSPLEVLISPRLDSKIYC
>CCHCYP82-5
MEYYSSLPFLQQWPLSSISSSPTSIATTLLIVACFTIVFLYINTSSKSNNNLKSPPKLPGAWPFMGHLHL
LGGNQPAHVSLANIAEKYKSTPFMIRMGQHSTLVVSSVDLVKECFNNTNDKLFSNRSIARGIRDMFYDME
SFGFAPYGYYWRELRKVSALTLFSTHRVDSVIQIKTPQIDGWFKKLYEQCTNTKGGIVEIKSWLDEMMFD
VLVKMLVEPKDEGSVEKYRHVAGEALEVLGNMSIYDLVPSLGWLDHFTGLVKKYKVTGKKMDTILNSWLE
DHRGEKVAEEHKGLIGKLLSMKEGSKLLGHDGDTAIKSTVQVLILGAGDSAGATLTWAISLLLNHPHIME
KLRKELDAAVGKDRQIEDSDVKKFTYLLAILKETMRLYPVTTLLQRETMEDCAIGGYHVAAGTRLMVNVW
QVQRDPNAWSDPLEFKPERFLTECAHVDFSGQYSELIPFGTGRRICPALTLSVRLMLLTLARIIHSFDVK
IPNGASSVDMATSVGFVNLRLTPLEVELSPRLDSKIYG
>CCHCYP82-6
MDSSLLQWYTTASMAALLALAFFYKLWSKPRTSKNGKSTLQAPVAAGAWPVLGHLHLFSGGELPHKMLAA
MADKYGPAFTMKFGTHRTLVVSDTKIVKECFTTNDTLFANRPSTMAFHLMTYDNESVAFTPYGQFWRELR
KISTLKLLSNHRLQAIKDVRASEVNFCFRTLYNQWRINKSETIGTGDHQGPILVDMKKWFEEVSNNVVIR
VIVGKHNFGSKIARGKGEALHYKKVMDEVLRLAAVSMLSDVAPLLGWFDHLQGHVSAMKRNAKEVDTLLC
SWMEEHRKKRTSNGSNGVEQDFIDVMLSIVEENKFSGHDSDTVIKATVLAMIMGGTDTSAVSLTWIVSLL
MNNRHVLRRAQEELDTHVGKDRQVDDSDLKNLVYLNAIVKETMRLYPLGALLERETKEDCEVGGFHVKAG
TRLLVNVWKLQRDPNFWIDPTEFKPERFLTDNANIDVGGQHFELLPFGAGRRVCPGVSFALQFMHLVLAR
LIHGFELDTPMNANVDMTESTEGHVNHKASALDLLITPRLRSTLYDY
>CCHCYP82-7
MFKSVPSAQAILHKLSFIHQYFLLSQTNMDSLLFQWFPASMAALVAFAFLYKLWSAPKITKNGKTSTKQA
PIAAGAWPVLGHLHLFGGGELPHKMLAGMAEKYGPAFTMKFGMHRTLVVSDTKIVKECFTTHDTLFANRP
STTAFHLMTYDQESVAFTPYGPFWRELRKISTLKLLSNHRLQAIKDVRTSEVNVCFRGLYNIWQTNKNEQ
GGTVSDHPRPVLIDMKKWFEEVSNNVVIRVIAGKQNFGSKIVRGEEEAVKYKKIMDEVLRLAAVSMLSDV
APLLGWLDLFQGNLSAMKRNAKEVDNMLNAWVEEHRRKRLAHSAKVEATHVEQDFVDVMLSIMEENKLSS
VHDDDTVIKATVLAMIMGGTDTTAVSLTWTVSLLMNHRYVLKKAQEEIDQHVGKERQVEDSDLKNLVYLN
AIVKETMRMYPLGALLERETKEDCEVGGFHVKGGTRLLVNVWKLQRDPNVWIDPTEFKPERFLAENANID
VGGQHFELLPFGAGRRVCPGVSFALQFMHLVLARLIHGFELETPMNADVDLTESTEGHVNHKASPLDLLI
APRLNSKLYEL
>CCHCYP82-8
MDSLFVLLQWFSASLAAVLALAFLYNLWVKPRISDGKGNKGSTKQAPVAAGAWPVLGHLHLFGGSELPHK
MLAAMADKYGPAFTMKFGTHRTLVVSNTQIVKECFTTNDTHFSNRPSTTAFHLMTYDNDSVAFTPYGPFW
RELRKISTLKLLSNHRLQAIKDVRVSEVNVCFKDLYNQWKTNQNQRPVLVDMKKWFEEVSNNVVIRVIVG
KQNFGSKIVRGEEEAVKYKKIMDEILRLAAVPMLSDMAPLLGWLDLFQAHKSAMKRNAKEVDNMLESWLE
EHKRKRLSGVNGAEEEDFMDVMLSIMEENKFTGRDSNTVVKSTVLAMIMGGTDTTAVSLTWIFSLLMNNR
HALKKAQEELDMHVGKDRQVEDSDLKNLVYLNAIVKETMRMYPLGALLERETKEDCEVGGYHVRAGTRLL
VNVWKLQRDPSFWTDPTEFKPERFLTENAKTDVGGQHFELLPFGAGRRVCPGVSFALQFMHLVIARLIHG
FELGTPMDADVDLTESTEGHVNHKATPLDLLIAPRLDSKVYDY
>CMACYP82-1
MRSNYLKPTTTTIEDKSKNKISRKKPIAAKPPPEALGAWPVIGHFLLFTGKGLTHVTLGDMADKYGPAFL
IRFGSHRTLVVSSWEMMKECFSAPNDKIFSNRESNLLWIKSMFYGSNSYGFSPYGPYWKELRKISTQKLL

SHHRLDAMKHLQMVEVDASFKQIYNLCNKNGGSPSPSSINTTALVNMDEWLSHLMFNVIARMVSGYQSDD
VGTGATSTGERFKVSMDETMRLMAIFAVSDLVPWLACVDRLRGLTRKMNRCGKDLDSIIGRLIEEHRQKR
RLCRNNKGSKNEDGGHDDDQDFIDICLSIMEQSQLPGDNPEIAIKSIVLDMIFAGSHTTTLAMTWTLSLL
LNHRHMLKKAKEEIDAHVGNERQVDDSDIHNLVYIQAIIKESMRLYPPSPLFERMTMEDCEVGGFHIPAG
TRLFVNVWKMQRDPTVWEDPLEFRPERFLTSKREIDVKGQHYELIPFGAGRRICPGASFTLQVLHLVLAR
LIHGFEMTTPMDVKVDMASSAGLFSNKMTPLEVLITPRTA
>CMACYP82-2
MKILQEFHQLTISNIVLLLLISFIFFSLSAWFIESTNYYYSSKKKKSKKTPPPEASGAWPVIGHLHQFKP
DDLPHEALGDMADKYGPVFIVRFGSYRKLVVSNSEMVKECFTAANDRSFSNRPAVLGIRIMLYNTITYAF
APYGPYWRELRRISSQNLLSNHRVDMVKHLHVDEVNTLFKQLYELCNKNGGRPPTGTNTSTSAALVNMDD
WLSNIMFNVIARIVNGNNKSINNERTGAINEKRYKTAMDEARRLVAIFAVSDSVPWLGWLDRVRGLIRGM
NLCGKELDSIIEDIIDEHRQKRRLCTSKLGSSGDDDADNSNLEEEDNFIDACLSIEEQSQLPGENPEIVI
KALILDMFAGGSDGPHFVLTWTLALLLNHPHVLKKAREEVDAHVANDRHVDVSDISNLVYIQAILKESMR
LYPPNALIERMTTEDCEVGGYHIPAGTYLLVNVWKVQRDPTVWQDPSEFRPERFLLTNTKTEMEYEHIPF
GAGRRRCPGMRFALQVMHLVLARLIHEFEITTPVDVIKVDMTATNGLFNHKAVPLEVLLTPRTLIRG
>CMACYP82-3
MDYSVLLQYGWFAPSMAALLALAFLYNLFLASSPKATSKRTISTKKPPMAAGAWPILGHLHLFKEGELPH
QMLKSMADKYGPAFLMKFGQHRSLVVSDYRIVKECFTTNDTHFCNRPSTTAFDVMTYANESVAFTEYSPY
WRELRKISTLKLLSNNRLQAIKNLREEEVNVSFKGLYDSWKNKNNKSTGSVGDERAPVLVDMKKWFEEVS
NNVVIRVIVGKCNFGTKIVQGEKEGVEYKTIMDELLRLASLSLLSDFAPILGLLDFFQGHVRTMKRNGKK
LDVLLQRWLEEHKRKKSTPEDEQDFMDVMLSVIEESKLSGYDADTVIKATCLAMIMGGTDTSAVSLTWIV
SLLMNNRHALKKAREELDAQVGKDRQVEDSDLKNLVYLNAIVKETMRLYPLGTLLERETKEDCEVGGFHL
EGGTRLLVNVWMVQRDPSVWTDPTKFTPERFLTEKADIDVWGGNFELIPFGAGRRVCPGVSFALQFLNLV
LARLIHGYELGTPDDADVDLTESPEGHLNHKASPLELLLTPRLSNPKLYDY
>CMACYP82-4
MEFLSLEPQLISIFVLLLASSIFLYNLLKNHGRKSKTSKPPAPVASGGWPIMGHLHLFNGSELTHQTLGS
MADKYGPAFNIQLGSHQTLVVSSWEIVKECFTTNDRFFSNRPGSLAIKLMFYDADSVGYAPYGAYWRDLR
KISTLKLLSNHRVETLKHLRTSEAESCFKQLYNQWKNNKVGGDDDEFAIVRMDNWFGDLTFNVVARIVAG
KKNFAGGATSGDVGAKRYKEAMDEAFRLMTIFAFSDVVPSLGWLDKLRGLVGGMKRCGAEIDSIVGGWVD
EHRLKRASRKGGDHNDLDLEQDFIDVCLEIMEHSTLPGDDPEIVIKSTCLDMILGGSDTTTVTLTWALSL
LLNNPHVLKRAREELDTHVGKDRQVDDSDMSNLVYIQAIIKETMRLYPAGPLIERRTSEDCEVSGFHVPA
GTRLMVNLWKMQRDGSVYKEDPLEFRPERFLTSNADVDLKGQNYELIPFGAGRRICPGVSFAVQLMHLVL
ARLVHGFEMKTAGGAKVDMTESAGLISHKVTPLQVLLKPRLAIQQAL
>CMACYP82-5
MDSMNLFQQAIAVGTLVIFLYYLWKWTSIIFTRTHQAAPPEPAGAWPIIGHLHLLAGGANQLVYHILGSM
ADKYGPIFTVRLGMRRALIVSNSELAKECFTTNDRIFSTRPSSVAIKLMGYDSAMFGFAPYGPYWREMRK
IAVTELLSNRRLEILKNIRISEINMSIKDLYQLWVENKNSGVDGGGDGGEVVVGSPVLVEMKRWFEDVSF
NVVVRMVAGKRYFGRRVESDDREEEEARRWQKAMNDFMHLVGIFVVSDAIPFLGFLDFQGYEKEMKKTAV
EIDYVMGRWVEEHQRRKLERVAGGSDDQQEDFIDVMLSILKDGDQFYGYDPDTVIKSSCLSLVLGGSDTI
SVTLTWTVSLLLNNRNVLEKAQSELDIHVGRGRQVDESDINNLKYLQAIVKETMRLYPAGPLSAPREAME
DCTIAGFHVPKGTQLMLNLWKLHRDPRVWSDPLEFKPERFLTTGTHGDVDVKGQHFELLPFGAGRRICPG
ISYALQVLHLTLARLLHSFHLSTPYDIPVDMTESSGLSCPKATPLDVLLTPRLPSELYVS
>CMACYP82-6
MDLFIFFSRFQYIVGLLAFLTFFYYLWRVSITGTRIKTNQNIMNGTNMMAPEAAGAWPIVGHLPQLVGPQ
PLFKILGDMADKYGSIFMVRFGMHPTLVVSSWEMAKECFTTNDKFLASRPTSAGGKYLTYDFAMFGFSFY
GPYWREIRKISTLELLSHRRVELLKHVPYTEIGGSIKQLYKLWMETQNQNKQRDDHQVKVDMSQVFGYLT
LNTVLKLVVGKGLFNNNDMNHEQEEGRKLHETVLEFFKLAGVSVASDALPFLGWLDVDGQKRSMKRIAKE
MDLIAERWLQEHRQKRLTSNNKASSGHDDFMSVLLSILDDDSNFFNYNRDTVIKATSLNLILAASDTTSV
SLTWVLSLLVTNPGALKKVQDELDTKVGRNRHVEERDIEKLVYLQATVKETLRMYPAGPLSVPHEATQDC
TVGGYQVTAGTRLVVNVWKLQRDPRVWPNPSEFKPERFLPDGCEVGCGEAANMDFRGQHFEYIPFGSGRR

MCPGIDFAIQIIHMTLACLLHAFEFQVPSSLDKHLVPAVIDMSEGSGLTMPKVTPLEVLLNPRLPLPLYE
L
>CMUCYP82-1
MEAMGEYFQLISWTVFSALITIYFIFSRKTNGKKKAPEPGGAWPIIGHLHLFGAHDLLYRKLGAMADKLG
PVFMIRFGMHRAVVVSNYEVAKECFTVNDKILASRPRIIASKLMGYNHAAVGLSPYGPYWREARKIATLE
LLSNHRLQLLKHIRISEIDMWIKELKEFCMKSSSDGPVLVQLDGWFNALTLNIMVRNIAGKRYCGGAEAF
EDEESQRWKKVSRELMFLFGVFMVPDAFPVLEGIDVQGFERAMKRVGKEMDFFLSRWLEEHRRKKIELSE
KGNEVNDQGAQDFIDVMLNTIKDAKIFEHDADTMIKATCMALVVAGNDTTMITLSWAVSLLLNNRGVLEK
AQEELSNQIGKNRHVEEGDIESLPYLHAIVKETFRLYPAAPLSLPHEAMEDCTIAGFHVPAGTRLITNIW
KLHKDPNIWPDPLEFKPERFLTTHAHVDFKGQHFEFIPFGSGRRMCPGSSLAISVLHLTLARLLHEFELK
TPDDAPVDMTEGPGITLMRANPLHVLINPRHD
>CMUCYP82-2
MSSPIQQLLLSLTSINGGTIISAVIISLLLILTLHRLLKPINSNKTKHPPRASGAWPVMGHLHKLRGPDL
PHRTLSSMADQYGPIFTINLGPTTALVISDPAVAKECFTTNDLTFSSRPTTLATTLMCYNNTMFGFAPYG
PYWREVRKVVMAQLLSTRRLESLKNIWASEIDRWVKGLAQKAQSGSHGVVEVEMGDWLARLTMNIGLRLV
VGKSCGEMGSEESERCLGAMREYPELLGRFALEDALPWLGRFDLQGHQREMRRAARVLDEILDGWLDEHK
RNRSGLSGGDDHRDQDFMDVMLSVLEQDQDSSLSDHDADMINKSTCLNLILGMADTTMVALTWAISLLLN
NKTSLKKAQEELQAQVGNERHVDESDVKNLVYLQSIIKEVLRLYPPEPLSGPREALEDCEVAGYHVPRGT
RLIANLWKIHRDSSTWQDSMEFRPERFLTAHEHVDVWGQHFEFMPFGSGRRSCPGVSFEVRVLPLILARL
IHAFEMTTRGDVPVDMSERPGLVISKASPLEVMISPRLPLHLFE
>CMUCYP82-3
MEFHLLLQAVAATLVTFFLYELWALRKKFSRLTKTPSSKALLKAKCAPEPPGAWPIIGHLPLLVSAKQPH
RAFAALAEKYGPAFMLRMGMSPMLIVSTKEVAKECYTANDHVFATRPVTTAGKLMAYDHSVMGFTPFGTY
WREIRKIATVELFSARRIGMLKPVRQSEVSLWMKGLHEKWVHNGNSSVSVELKSQLEELTFNLLTQMVAG
KRYYGSNVAKVDEKMAGLFRHAVQQFNYHLGNSEMYDALPFMAWLDFKGDAKAMKKTQKDLDFIMQTWLD
EHRLKADQMRGDAINNTRDFLDVLVMMEKTGQFSSAIKDRDTTIKALALTQLVAGVDSMANTMVWVLALL
MNNPEMLAKAQDELDNNVGKDRLVEESDIPNLKYLQALLKETLRMYPVGPLLVPHEATEDCHVAGYFVPR
GTGLFINAWTIQRDPNVWAEPNCFNPERFLTTHAEMDVKGQNHELLPFGSGRRSCPGVGLALQVMHLTLA
RILQAFELKTQSGVGIDLEECSGILLSMMHPLQVMMAPRLPSELYD
>ECACYP82-1
MDSLILTNWFPISIASVLTLVFLYKVLLSSRTLKDKKIKTSPMANGAWPILGHLHLFGSGELPHKMLATM
ADKYGSAFRMKFGKHTTLVVSDTRIVKECFTTNDTLFSNRPSTKAFQLMTYDNESVAFTPYGPYWRELRK
ISTLKLLSNHRLQAIKDVRASEVNVCFKSLYDQCKNPSGAPILIDMKKWFEEVSNNVVMRVIVGRQNFGS
KIVQGEEEAIHYKKVMDELLRLASLSMFSDFAPLLGFLDIFQGNLSAMKQNAKKVDAILENWLEEHRKKK
NSVAESEQDFMDVMLSIANESKLSGHDADTVIKATCLAMIMGGTDTTAVSLTWIISLLMNNRHALKKARE
ELDALVGKDRQVEDSDLKNLVYMNAIVKETMRMYPLGTLLERDTKEDCEIGGFHVKGGTRLLVNVWKLQR
DPNVWVDPTEFRPERFLTENADIDVGGQHFELLPFGAGRRVCPGVSFALQFMHLVLARLIHGYDLNTLNE
ENVDLTESPEGHVNHKASPLDLILTPRLHYKLYE
>ECACYP82-2
MDTASSISLLLPWAVVVPSIATLLALLLFLFITSPKTPKRKTSSKPPPTVLPGAWPILGHLHLFKEGESP
HHMLKNLADKYGPAFVMKFGQHRSLVVSNTKIVKECFTTNDTLFSNRPSTTAFDLMTYAHDSVAFTPYSP
YWRELRKISTLKLLSNNRLKAIKKLRGEEVDVCFRGLYGLWKNKTKNGAPVLVDMKKWFEEVANNVVIRV
IVGKLSFGTKIVDGEEEAVEYKTVMDELLRLASLSLLSDMAPILGWLDFFQGSVRKMKQTGKRLDVLLEK
WLGEHREKKNLVGEDEQDFMDVMLSIVEESKLSGHEADAVIKATCLAMIMGGTDTTAVTLTWIISLLMNN
RHALEKAREELETHVGKDRQVEDTDLQNLVYLSAIVKETMRLYPLGTLLERETKEDCEVGGFHIQGGTRL
LVNIWMVQRDPTVWNDPSAFKPERFLTDKSEIDVGGQHFELIPFGAGRRVCPAVSFALQFLHLVLARLIH
GYDLGTPSNVEVDLTESLEGHVNHKASPLELLLTPRLNPKLY
>ECACYP82-3
MESEIQYQTATMASILSTNLILSSIFTAFLLYFLLKMSPFRSSKNKSRKQAPKPTGSWPIIGHLLHLQGP
NLPHINLAALADKYGSVFSFRIGLRPALVVSSWEIAKECLTTNDRVFVSRPPLIAMKHMGYDHALFGFSP

YGPYWRELRKLVNQELLSNTRIELIKHVWDTEINTFINDLYEVWAMKSNEGGGDVVVEMKQCLYDFTLNL
TLKILTGKRYFGGGGSEELREEAGRCQKAVRNFLRLVGTFTAQDVIPFLEGWLDLGGHEKEMKITGKELD
SLLQKWLEEHKVKKSSAGEEGQENRGGDEEDFMGVMLTKLRDHEKLLSYYDADTINKATCLTVILGGSDT
TMMTLVWALALLVNHPHVMKKLQDELDTHVSKERQVEESDIKNLAYLQSVMKETMRRYPGTPLYVRESIE
DCTIAGYDVPTGTRLVVNAWKIQHDPQVWSNPFEFNPERFLTTHKDIDVRGQNFELIPFGAGRRMCPGAS
LGLQVVHLTLARLVHGFEFKTPDGEPMDMTESIGLTNLKATPLEIMLTPRLSSKLYVC
>ECACYP82-4
MGLSFDLSSHNFLQFSNPTILIGLFGLLVYLLLVNLRKRISRKNEAPEVEGGWPIIGHLHHFMGGKNKLL
HVAFGAWADKYGPVYTLRMGLNKVLVVNSAEVAKECSTTNDMLFMARPYRIASEIMGYGYAMFPIAPYGP
FYLKMRKMVTQELLSNSRVDSLKHVWGSEIKNAIQELHKKVLSTKGGGPISMDMKGWVSNLTFRTAMKVI
CGGVGVDGGGGGGGATSPATSIGEHNYDEVGSFQKALKEFFVLLGEIRISDVIPFLGWLDMRTGYVEKMK
DNGKFLDNLMEEMLEEHKRKRRSLTEEEEKDGGYVEQDFMDVMISKLNDPKLLSYYDADTINKSTCLTLI
LSGSETTMVSIVWALALLVSHPHVLKKAQDELDTLVGRERQVDESDIKDLVYLQAVVKEALRLFPPAPLS
TPRVATEDCVVSGYHVPAGTQLFVNTWKIQRNPEVWPEPSEFRPERFFTTHKDFDVRGLHYDLHPFGSGR
RACPGAGFALQVVHLTLASLLHGFEIKNPSDEPIDMTESPGVTNLKATPLKVLLTPRLFSKEVY
>ECACYP82-5
MEKPILLQLQPGILGLLALMCFLYYVIKVSLSTRNCNQLVRHPPEAAGSWPIVGHLPQLVGSGKPLFRVL
GDMADKFGPIFMVRFGVHPTLVVSSWEMAKECFTSNDKFLASRPPSAASIYMAYDHAMLGFSSYGPYWRE
IRKISTLHLLSHRRLELLKHVPHLEIHNFIKGLYGIWKDHQKQQQQPTARDDQDSVMLEMSQLFGYLTLN
IVLSLVVGKRVCNYHADGHLDDGEEAGQGQKLHQTITDFFKLSGVSVASDALPFLGLFDLDGQKKIMKRV
AKEMDFVAERWLQDKKSSLLLSSKSNNKQNEAGEGDVDDFMDVLMSTLPDDDDSFFTKYSRDTVIKANSL
SMVVAGSDTTSVSLTWALSLLLNNIQVLRKAQDELDTKVGRDRHVEEKDIDNLVYLQAIVKETLRMYPAG
PLSVPHEAIEDCNVGGYHIKTGTRLLVNIWKLQRDPRVWSNPSEFRPERFLDNQSNGTLLDFRGQHFEYI
PFGSGRRMCPGVNLATPILHMTLARLLQSFDLTTPSSSPVDMTEGSGLTMPKVTPLKVLLTPRLPLPLYD
Y
>ECACYP82-6
MNLSSKLMGSFDLFSLNFLQFSKPASMVIIGICSFLVYFSLLNLVRRVLAPSCTMKNGKTEPPEVENGWP
IIGHLHHFMGKKNKLIHEIFGDMADKYGPTFTLRMGLTKVLVVSSAEVAKECLTTNDLVFIGRPPRVANS
LLGYSFAMFPFSPYGTYYSQMRKIVTHELLSTSRVESLKHVWNSEINKAIQELHHKVVSVGGGSPVLIEF
KRWFSDLTLRTTVKLICGKQYFGTDGATQASMTINGGGDDDEAGKFQEALREFFCLLGKFRVSDVIPFLG
WLDFGTGYKEKKNRMFIDSLMEEWLEEHKMKRRLLNEADKKESRIEQDFMDVMISKLDDPNLLSHYDADT
INKATCLTLILGGSDTTMVSLVWALTLLMNHPHVLKKVQDELDFHVGRERQVEESDMKNLVYLHAVMKEA
MRLNPAGTLSAPRMSTKDCTVSGYHIPAGTHLFMNTWKIQRDPNAWVEPTEFRPERFLTTHKDFDLRGQN
FELLPFGSGRRSCLGANFALQVLRQTLARLLHGFDLKTPSDEPVDMTGSAGLINMKATPLEVLVTPRLFS
SELYG
>ECACYP82-7
MNLLIFFQFLLQFQVLVGLSVLLAFSYYLWVSKNPKINKFKGKGALLAPQAAGAWPIVGHLPQLVGPKPL
FRILGAMADNYGPIFMLRFGVHPTVVVSSWEMTKECFTTNDRHLASRPSNAASQYLIYEVYALFGFSLYG
SSYWRDARKIATLELLSHRRLELLKHVPYTEIDTCIKQLHRLWTKNNKNQNNPELKVEMNQFFTDLTMNV
ILKLVVGKRFFNVDDAADHEKEEARKIQGTIFEFFKLTEGSVSAGALPLLNWLDLNGQKRAMKRTAKKMD
SIAEKLLDEHRQKRLSKEGVKGTHDHNDFMDVLLSILDADQGDYSHHPFNYSRDHVIKATTLSMILSSMS
ISVSLSWALSLLLNNRHVLKKAQDELDMNVGKDRQVEEGDIKNLVYLQAIVKETFRMYPANPLLLPHEAI
EDCKIGGFNVPAGTRVVVNAWKLQHDPRVWSNPSEFKPERFLNDQAAKVVDVRGQNFEYLPFGSGRRVCP
GISFSLQTIHMSLARLVQAFELGTPSNERIDMTEGSGLTMPKTTPLHVLLNPRLPLPLYE
>ECACYP82-8
MEFHFPLMEQFQPFFIFALLLASFIFLYKLFNFGNRITKNGKPTAPEASGGRLIMGHLHLFNGTELTHRT
LGSMADKYGPAFNIRFGSHKTLVVSSWKIIKECFTTNDRFFSNRPGSLAIKLMFYDADSVGYAPYGSYWR
ELRKISTLKLLSNHRLETLKHLRTSEVDSCFNQLMNSWAENKNRGDSDFAPVRMDDWFGDLTFNVVARIV
AGKKNFAGGAARGDAGAQRYKSAMDEAFRLMTIFAYSDVIPSLGWLDKLRGLVGDMKRCGSEIDSVVESW
VDEHRLKRRVSKKGGELDLEQDFIDVCLDIMEHSSLPGDDPEIVIKSTCLDMILGGSDTTTVTLTWALSL

LLNHPQVLKRAKEELDSQVGKERQVEDSDIPNLPLIQAIIKETMRLYPAGPLIERRTMEDCEVAGFHVPA
GTRLLVNLWKMQRDKEVWSEEPLEFRPERFLTSNTEVDLKGQHYELIPFGAGRRICPGVSFAVQLMHLVL
ARLLHGFEMTTPMGEKVDMTESAGLISHKITPLEVLIKPLRV
>ECACYP82-9
MDSFLLAYWVPISVASIIAFVFLYNLFSSRTLQNKKIRTAPMATGAWPILGHLHLFGSGELPHKMLAAMA
DKYGSAFRMKFGKHTTLVVSDTRIVKECFTTNDTLFSNRPSTKAFQLMTYDNESVAFTPYGPYWREIRKI
STLKLLSNHRLQAIKDVRASEVNVCFKTLYDQCKNPSGSAPILIDMKKWFEEVSNNVVMRVIVGRQNFGS
KIVQGEEEAIHYKKVMDELLRLASLSMFSDFAPLLGFVDIFQGNLSAMKRNAKKVDAILENWLEEHRKKK
NSVAESQQDFMDVMLSIVEESKLSGHDADAVIKATCLAMIMGGTDTTAVSLTWIISLLMNNRHALKKARE
ELDALVGKDRQVEDSDLKNLVYMNAIVKETMRMYPLGTLLERETKEDCEIDGFHVKGGTRLLVNVWKLQR
DPNVWVDPTEFRPERFLTENADIDVGGQHFELLPFGAGRRVCPGVSFALQLHAFSTCSPHPWIRFEYSKR
RKCGSDGEPRRTCEPQSIAS
>GFLCYP82-1
MEFLSLQLQPVSIFALLVASIFLYNFLIHGKKSNNKKTTKPPAPEASGGWPIMGHLHLFNENELTHRTLG
SMADKYGPAFNIRFGSHPTLVVSSWDIVKECFTTNDRFFSNRPGSLAIKLMFYDADSVGYAPYGAYWRDL
RKISTLKLLSNHRLETLKHLRTSEVESCFKELYNQWRNNKTGGGGDGFAPVRMDNWFGDLTFNVVARIVA
GKKNFAGGAASGDAGAQRYKEAMDEAFRLMTIFAFSDVVPALGWLDKLRGLVGGMKRCGAEIDSIVAGWV
DEHRLKRSSGKGSDADLEQDFIDVCLEIMEHSTLPGDDPEVVIKSTCLDMILGGSDTTTVTLTWALSLLL
NNPHVLKRAREELDTNVGKDRQVDDSDIPNLVYIQAIIKETMRLYPAGPLIERRTSEDCEVGGFHVPAGT
RLLVNLWKMQRDGSVYKEDPLEFSPERFLTSNADVDLKGQNYELIPFGAGRRICPGVSFAVQLMHLVLAR
LIHGFEMKTPEGGKVDMTESAGLISHKVTPLEVLLKPRLAIQHS
>GFLCYP82-2
MHAKLTLIKKMEFTIINSLEIQPIISTFALLTFSILLYKILLNHGRENKNNKPKTSSSSSSIPEVAGAWP
IMGHLHLFNGDELMHHKLGSMAEKYGQAFYIRFGSHKAVVVSNWEMVKTCFTTNNQIFLNRPPMLAINLL
FFPTDSLSYIPYGDHWRELRKFSNQKLLSNHRIETQKNLRKLEVDYCFKQLCNQSSKYFIINNMDDQDSK
FALVRMDTWFDDVTLNVLARIIAGKKKFISGGATSSGDDDNAEARRYMEALDEGLRLMTSFTFSDVLPWL
GWLDNLRGLAGKMKRCGAELDSVFAGWVEEHRVKRGSRKDGDDADLEQDFIDLCWESLEQVPGNDPAKII
KLICMEMILNGSGATAVTLTWTVSLLLNNPDVLKRAREELDTHVGSHRQVDESDIPNLVYIQAIIKEGMR
LYPPGPFLERSTTEDFEIDGVHVPAGTRLWVNLWKMHRDESMYQEPLEFKPERFLNSNSDVDLKGQSYQL
LPFGAGRRICPGVSFALPLMHLTLARLIHGFEMKLPVGVEKVDMTENGGIINRKATHLDVLLKPRLIAQQ
A
>GFLCYP82-3
MDTLSIQWIVPSIATLLALVFLYNLIFTSKKTTKTNNTRKAPMASGAWPVLGHLHLFGTGELPHKMLATM
AENYGTAFTMKFGNHTTLVVSDTRIVKECFTTNDTLFANRPSTKAFDLMTYANDSVAFTPYSPYWRELRK
ISTLKLLSNHRLQSIKDVRVAEVNVCFRGLHVLCKSKIYGAPVLVDMKKWFEEVSNNIVMRVIVGKQNFG
SRIVQGQEEAVYYKSVMDELLRLASVSVLSDFAPLFGWLDFFQGNISAMKRNGKKLDVILERWMEEHRQK
KISSSSSIAASGAGEDDEQDFMDVMLSIIEETKLSGRDADTVIKATCLAMIMGGTDTTAVSLTWIVSLLM
NNRHVLKKAREELDSLVGKDRQVEDSDLKNFVYMNAIVKETMRLYPFGALLERDTKEDCEVGGFHVEAGT
RLLVNVWKLQRDPNVWKDPLEFRPERFLVENVDIDVGGQHFELLPFGAGRRVCPGVSFALQFMHLVLARL
IHGYELETLNGEDVDLTESTEGHVNHKASPLDLLITPRLDSKVYNY
>GFLCYP82-4
MDSTLVLQCFVGSMAAVSALVFLYNLIFSSSKTTKGKVLRKAPMAAGAWPILGHLHLFGSGELPHKMLSK
MAEKYGPAFTLKFGKHTTLVVSDTRVVKECFTTNDTLFANRPSTTAFDLMTYANDSVAFTPYSPYWRELR
KISTLKLLSNHRLQSIKEIRVSEVNVCFRELFEMSKSKTDGAAPALVDMKKWFEEVSNNIVMRVIVGRQN
FGSKIVQGDAEAVNYKNVMDELLRLASLSMLSDFAPLLGWVDMFQGNKNAMKRNAKKVDTILEGWLEEHR
KKNKKMSSSENDEQDFMDVMLSIIEETKLSGRDADTVIKATVLAMIMGGTDTTAVSLTWIVSLLMNNRHV
LKKAREEIDAIVGKDRQVEDSDLKNFVYMNAIVKETMRLYPLGAMLERDTKEDCEVGGFQVQAGTRLLVN
VWKLQRDPNVWSDPSEFRPERFLSENADIDVGGQHFELLPFGAGRRVCPRVSFALQFMHLVLARLIHGYE
LGTQNDELVDLTESTEGHVNHMASPLDLILTPRLSNPKLYDY
>GFLCYP82-5

MFFTSSSKTTNKNTSKKPPMAPGAWPILGHLHLFKEGELPHHMLKSMADKYGPAFLMKFGQHRSLVVSDY
RIVKECFTTNDTLFSNRPSTTAFAVMTYATDSVAFTEYSPYWRELRKISTLKLLSNNRLQAIKKLRESEV
NVCFRGLYDSWRKNKSEQNGAGNSIDGGNERARPVLVDMKKWFEEVSNNLVMRVIVGKRNFGTKIVEGEK
EAVEYKTIMDELLRLASLSLLSDFAPILRLFDHFQGHIRTMKRNGKKLDVLLQRWLEEHRRKMSTPEEEQ
DFMDVMLSIVDESKLSGHDADTVIKATCLAMIMGGTDTSAVSLTWIVSLLMNNRHALAKAREELDKHVGK
DRQVEESDLKNLVYLHAIVKETMRLYPLGPLLERETKQDCEVGGFDIAGGTRILVNIWMVQRDPAVWNDA
TEFIPERFLTEKSDVDVWGGSFELIPFGAGRRVCPGVSFALQFLHLVLARLIHGYELKTPNDMPVDLTES
TEGHVNHKASPLDLLLVPRLSDLKLYDY
>GFLCYP82-6
MDSILTTVVLLSILLYFLFSWQLNKYSATKKNTKSSKQLPPEPAGSWPVIGHLHLLAKGSNLPHINLGAM
ADKYGPIFMIRIGLNPTLVVSSWEVAREIFTTNDQVFSSRPIQVSTKHLGFDTAMYGFAPYGPYWREISK
LVKREVLSNTRLEFLKPVWGSEINTSIKELYDVCVMKNKEEGGTGPIIVEMKQWFSDLALNMSVKLVAGK
RYFGASQLGNEEAARWQKALRNCFRLVGLFVVSDAIPFLRWLDVGGHEKEMKNTAKELDDLLEGWLEEHK
MKKKLSLSEVEAEKKERDRVDFMDVMLSTLEHEKASDYFPADTINKATCLALILGGTDTTTVVWVWALAL
LVNNPNVLKKAQDELDVHVGKKRQVDESDIKNLTYLQAIIKESMRLYPAATLGIRESTEDCTVAGYHIPA
GTSLIVNSWKIQHDPQVWTDPFEFQPERFLTGHMDVDIRGQSCKFLPFGSGRRSCPGTSLALQMVTLTLA
RLIHGFEFRTPSEAPTDMTESAGLTNVKATPLEVLVSPRLPSELYVC
>GFLCYP82-7
MELINSLEIQPITISILALLTVSILLYKIIWNHGSRKNNKSNKNNRKTSSSAGVVEIPGAWPIIGHLHLF
NGSEQMFHKLGSLADQYGPAPFFIRFGSRKYVVVSNWELVKTCFTAQSQIFVSRPPMLAMNILFFPKDSL
SYIQHGDHWRELRKISSTKLLSSHRVETQKHLIASEVDYCFKQLYKLSNNGEFTLVRLNTWCEDMALNVH
VRMIAGMKNYVAAPGSGEYGGQARRYRKALEEALDLLNQFTITDVVPWLGWLDHFRDVVGRMKRCGAELD
SIFATWVEEHRVKRASGKGGDVEPDFIDLCWESMEQLPGNDPATVIKLMCKEHIFNGSGTSSLTLAWILS
LIMNNPYVIKKAREELEKHVGNHRQVEESDLPNLLYIQAIIKEGMRLYTPGPFIDRNTTEDYEINGVHIP
AGTCLYVNLWKIHRDPNVYEDPLEFKPERFLKNNSDLDLKGQNYQLLPFGAGRRICPGVSLALPLMYLTV
SRLIHGFDMKLPKGVEKADMTAHGGVINQRAYPLEVLLKPRLTFQQA
>GFLCYP82-8
MDLQIFFHFQGIVGSLALLSFFYYLWRLLTTTKTSICNGTTAAPPEVSGGWPILGHLLQLVGSKQPLFKV
LGDMADKYGPIFVVRFGMYPTLVVSSWEMAKECFSTNDRVLATRPTSAASKYLTYNYAMFAFTFYGPYWR
EIRKISTIELLSHRRVEMFKHVPFMEIDTCIEQLYLLWMQNQNQNQNQPNGDPVQVNMSKVFEELTMNAV
LKLVVGKRLTDDKEGEKLHKTIQEFFKLLEVSVASDVFPFLGWLDVDGQKRKMKRVAKEMDIIAEKWLEE
HRQKRSSKLEEEEEEEEDDGGGKGDAAAADKDFMDVLLSLLEGDEGDSDQPFMNYSRDTVIKATSLNILV
AATDTTSPTLTWAISLLLNNPHVLREAQNELDMKVGRDRQVEEQDIENLIYLQAIVKETLRLYPAGPLSI
PHEAIQDCKLGGYHVRAGTRLLLNIWKLHRDPRVWSNPLEFKPERFLILSEEVCGCSRGTQNFDFKGQCF
EYIPFGSGRRMCPGYNFGIQIIHMTLARLLQSFEMQPAKAKSLNDQDGPVDMREGSGLTLPKITPLKVLL
TPRLYGQLYNH
>HCACYP82-1
MDFSMLFQWLLVTMATLLFLNSVYNVWYKSSKNTSITTTTSKGKKAPVAAGARPFMGHLHMLVGGKQLPH
QALGKLADIYGPAFIIHIGPNPELVVSSWELAKECFTTNDKYFANRPTNKAMKYLTYDEASVGFGPYGPL
WVEMRKIAKSNLLSQQRLQMHKRVRVLEIDAFFKELHELWSMKKEDGPVSVDMKQWFEELTLNVITRMVS
GKHKYATKARRGDSEAKQFKRVIGEAAHFTGNLLLSDIFPSLGFLDNMQGRVNSMKRTGKELDSILSSWV
EEHRQKKLSGEQSEDEEKDFIDLTLSMMDEIQLHSTDSETFIKAICVGVILGGSDTTSVGITWILSLLMN
HRDVLKKAQEELDQQVGMDRKVEDSDLNNLVYLRAIVKESMRLYQVGPLIERKASQDCTIGGFHVKAGTR
LLVNLSKVHKDPTVWSDPLEFQPERFLTTHSNMDLKGQHFELLPFGSGRRMCPGYLFALNEMYLVLARLI
QGFELGTPMDAKVDMTETSSVTNYRATPLQVLLTPRLSPKLYDY
>HCACYP82-2
MAFVALIVVYNIWFKSAASRNKTSYNNKTMSKTPVVDGAKPIIGHLHLLMGGDLPHHALGRLADKYGPIF
LMHIGAFPELVVSSSELAKECFTTNDKYIINRPTNKAMTYLSYEQASVGFAPYGPLWIEMRKISKSNLLS
NQRIHMQKQVRVAELDAFFKELYQLCRSNDENNNNSTSHGKVLVEMNKWFEELTLNVVTRMICGKQKMGT
KARLGDSDAKHYKKTIDEAAHFMGNLVISDVVPCLGMLDNLLGHVSAMKRTGKELDTIFGSWVEEHQQKI

RLSGYKDDAEEEEHDFIDLTLSMMKGSTDLHGLDPATFIKSICVGMILGGTDTTSVALTWILSLLLNNRH
ILKKAQEELDHQVGKERKVEDSDLNNLVFIGAIVKESMRLYPVGPLIEREAIEDCQIGGVHVKAGTRLLI
NIWKVQQDPKIWPNPSEFRPERFLDSNMDVKGQHFELIPFGSGRRMCPGMSFAINELNLVLARLLQGFEL
ETPMNAKVDMTETSSVTNYKGTPLQVLLTPRLSSKLYM
>HCACYP82-3
MEYSHLLPWLATSIAAIFAFIFLFYVRRRKNNKAFIHNHAKKAPVVPGALPFLGHLRLFTGPILPHKALG
ALADKYGPAFTIYLGSHQTLVVSGRELVKECFTTNDRLFSNRPRSKAVKYLTYDEASVGFAPYGPLWVEM
RKVSKLNFLSNQRIQMQKQALASELNFCFKDVYQLWLKNKELPVMVDMTKWFEEMTLNVITRLICGKQNY
GSKANSGESEAKRYKQVVEKAAHLTSTVVMSDVFPFLEWFDKFQGQEKVMKKVANEFDSILGSWVDEHRR
KRLLRGNNEEEEEEQDFIDLSLAMMEETQLHGVDPDTFIKSMCLGMILGGGDTTPVALTWALSLLLNNPD
IMKKAQEEIDQVIGKERKLDGSDISNLVYLQAVVKESMRLYQVGPLIERETTEDCKIGDFHVEAGTRLLV
NIWKVQQDPCVWSNPTEFQPERFLSSKSDMDLKGQHFELIPFGSGRRMCPAVASALQMMHLVLAKLIHGF
ELGTPMNAKIDMTETSSITNNMATHLQVLLNPRLNANLYDF
>HCACYP82-4
MADFTMLLQWLLLTMATLLFLNAVYKSIKSSKNTINDTSFKKGKKAPVAAGARPFIGHLHMLVGGKQLPH
QALGKLADKYGPAFIINIGPNPELVVSSWELAKECFTINDRCFTDRPSNKAMKYLTYDEASVGFAPYGPL
WVEMRKIAKSNCAFQQRLQVQKRVRVLEIDLFFKELHELWSHAGAGATGTSVIPLSIDMKKWFEELTLNV
ITRMVSGKHNYATKARKGDTEAKRFKRVISEAAHFTGRLLLSDIFPSLGFLDNMQGRVNSMKRTGKELDS
VLSSWVEEHRQKRVSGNKEQSPEDDDVEQDFIDLTLSMMEEIQLHWTDADTFIKAICVGVILGGSDTTSV
GITWILSLLMNHPDVLKKAREELDQQVGLERKVDDSDLNNLAYLRAIVKESMRLYQVGPLIERKAKQDCN
IGGFHLKAGTRLLVNLSKVHKDPTVWSDPNEFRPERFFTTHTNIDIKGQNFELIPFGSGRRMCPGYLFAL
SEIYLVLARLIHGFELRTPNDAKLDMTETSSVTNYRATPLQVLLTPRLSSKLY
>HCACYP82-5
MDYFSMPYQWLLTSLATLIVFVFIWSITAGKTSTTSNKAKKAPPIAAGAWPIIGHLHLIMGGELPHRLLS
NLADKYGPIFMLNYGSQPGLVVSSLKLAKECFVHNDRAVFKRPNSKAMKYFTYDQASFGFAPYGPLWVEM
RKVSKSNLLSNQRLQLQRNQRASEVDAFIKELYQLCKKSNNGTLMVEMNKWFEELTLNVVTRMVCGKKNI
GAKARHGGDNEAKYYKKVIDEATIFTAKLVASDFFPSLGWVDYLHGDESAIKQTAKELDSIIGSWVEEHR
QKRLLSLNKDDYAEQDFIDITLSMIDQTQHQGIDADTFVKSMCVGMIFGGSDSTSVALNWALSILMNNRH
VLKRAREEIDTLVGKDRKVNDVDVTKLVYLKAIVKESMRLCLVGPLLERVTVEDCEIGGFYVKAGSRIVV
NIWKLQHDPDLWSDDVMEFRPERFLTTNSNVELRGQHFELIPFGSGSRMCPGVTFALELMHLTLARLIHG
FELGTPMDAKVDMTETSSVTNYKATPLEILLTPRLHPKLYDF
>HCACYP82-6
MFISITQQSMDSFHQYLATIIFGVFSFVLFLIYYLPLKRSRSDKKRAAPKPVGAWPIIGHLPMLSRHQPP
HITLGNIADKYGPAFTLQLGVHRALVVSSSEVAKECFTTNDKALASRPSSVALKLMGYNNAMFGFGPYGS
YWRQMRKIVVLELLSNHRLQLLKHVRISEVSTSLKELYQVWASCTNKNDKGQVLVDMQQWFGDLTLNVSV
RMIAGKRYFGASAACDEDEARRFQKAIKDFLHLVGLFVVSDALPFLEWLDIQGHQKAMKRTFKELDRILG
KWVEEHRRNKLDGGTNVGRDFMDVMSSILDDANISDYDADTINKATCLSLILGGTDTTMVTLTWALSLLM
NNQHILKKAQDEIDIYVGKDKNVDESDIEKLVYLQAIVKETLRLYPAGPLSGPHEAIEDCTVAGYHVPRG
TRLITNLWKIQRDPRIWSSPCEFQPERFLTTQANVDVRGQHFEFLPFGSGRRSCPATSFALQVVHLALAR
VLQGFEFETQSNAPVDMTESAGLTNVKATPLEVLITPRLPLNLY
>HCACYP82-7
MESLHQYLAAVMSCIFALLLFLYYFPWKIRRSSSDNNMRSAPEAAGAWPIIGHLPMLGGHQLPHITLGNL
ADRYGPAFTIRLGVCRALVVSSSEVAKECFTTNDKAFATRPSSVAVKLMGYNYALFGLAPYTSYWREVRK
IVILELLSNRRLELLKHVRISEVNTSIKELFQVWASSNNKNEKGQVLVEMQRWFGDLTINVAVRMVAGKR
YFGASVNTCDDEEEARRFQKAIKNFFHLVGLYVLSDSLPFLEWLDFEGHQKAMKRTFKDVDCILQRWLEE
HRRDKENGAMKEERDFMDVMLSILKDANLFGYEADTVNKATSLNIILGATDTTMVTLTWALSLLLNNRHI
LKKAEDEIDIHVGKDKAVDESDIEKLVYLQAIVKETLRLYPVAPLLAAHEAIEDCIVAGYHVPRGTRLIP
NIWKIQRDPRIWSSPCDFQPERFLTTQANVDVRGQHFELFPFGSGRRMCPGISFGLQVVHLALARVLQGF
EFDTPSNKAIDMTESAGLTNLKATPLQVLITPCLPLNLY
>HCACYP82-8

MDSILLPTPLMVSLFALLLCLYYLIISRPRSSHNTSTKEAPEAAGALPIIGHLHLLGGRILPHITLGAMA
DKHGPAFMIRIGVHRALVVSSSEVAKECFTTNDKAFASRPKHTAAELMGYNYAMFGFAPYGPFWSEMRKI
IMAELLSNRRLELLKYIRDFELKASIKELYMTWENHSVTNKGQVVVEMKKWFGDLTLNVILRMIAGKRYF
GSNSTCDESEAKICQKGMRDFFRLLGEFLVEDAIPYLGWLDLQGFKKEMKNTAKELDILLQGWLEEHKKK
REFSKDVKEEQDFMDVMMTILEDANFSDFDADTINKATCLTIISGGSDTTMLTLTWALSLLLNNQHVLKK
AQDELDTHVGRDRRVDESDIKNLVYLNAIVKETLRLYPASPLLGIRVSTEDCTVAGYHVPSGTRLMVNAW
KIQRDPLVWSDPFEFRPERFLTTHVNVDVKGQNFNLIPFASGRRVCPGVAFALQMLPLVLAHLLHGFKLM
TQLGGPVDMTESTGLTNIKATPLEVVISPRLRPELYEMYT
>HCACYP82-9
MESLHQYLATILSCIFAFLLFLYYFPWKGRRSFDNNLRTAPEAAGAWPIIGHLPMLNQRDLPHVTLGNLA
DRYGPAFTIRLGVRRALVVSSSEVAKECFTTNDKAFATRPSSVAVKLMAYNYAVFGFGPYGSYWREMRKI
VILELLSNRRLELLKHVRISEVNTSIKELYQVWASNNKNEKGQVLVEMQRWFGDLTMNVVVRMVAGKRYF
GASVTCDEEEARRFQKAIRDFSHLAGLYVLSDALPNIEWLDFEGHHKAMKKTFKDLDCILQRWLEEHRRD
RENGATKGERDFMDVMLSILKDANPFDFEADTVNKATSLNMVLGGTETTTVTLTWALSLLLNNQHILKKA
QDEINIRVGKDKPVDESDIEKLVYLQAIVKETLRLYPVLPLSVPHEAIEDCTIAGYHVPRGTRLITNLWK
IQRDPHIWSSPCEFQPERFLTTQANVDVRGQHFELFPFGSGRRMCPGISFGLQVVHLALARVLQGFEFET
PSNVPMDMTESAGLTNFKTTPLEVLITPCLPLDQY
>HCACYP82-10
MDSLLQLQIIGALAALIFTYKLLKVICRSPMTDGMEAPEPPGAWPIIGHLHLLGGQDPIARTLGVMTDKY
GPILKLRLGVHTGLVVSNWELAKECFTTNDRVLASRPMGAAGKYLGYNYAIFGLAPHGPYWSEVRKIVLR
ELLSNQSLEKLKHVRISEINTCLKNLFSLNNGNTPIKVDMKQWFERPMFNVVTMMIAGKRYFSMENDNEA
MNFRKVATEFMYLTGVFVVSDALPYLEWLDLQGHVSAMKRTAKELDIHVGKWLEEHRRAKLLGETKNEDD
FVDVLLTILPEDLKDNQTYIHDRDTIIKATALALFLAASDTTAITLTWALSLILNNPDVLKRAQDELDKH
VGKEKLVKESDIINLVYLQAIIKETLRLYPAAPLLLPHEAMEDCTVGGYHVPKGTRIFVNIWKLQRDPRV
WFDPNEFRPERFLTTHANVDFKGQHFEYIPFSSGRRVCPGITFSTQIMHLTLAHLLHEFNIVTPTKSNAG
VDMTESLGITMPKATPLEVLLTPRLPSNLYNQYRD
>JDICYP82-1
MDSLQLIASVVCGLFAFLRIYYLLKKLSSNKEAPQLGGAWPIIGHLHLLGRVELPHIFFSAMADKYGPAF
MIQLGMRRALVVSSAEIAKECLTTNDKALATRPSSIAAEIMGYDYAMFGLGPYGDYWREVRKIVVLEFLS
NRRLELLKHVRVSEISLSLKELYQSWEKQNKADEPVLVHLDQWFGDMTLNLSVRMVAGKRYFGATAACGE
DESRKVQKATREFDRLLGLFVVSDYLPFLRWLDLEGHQKLMKSIGRELDSILQGWLDEHKTRRNSGGGAK
GDQDFIDVMLSVLEDGNLSNYDADKLNKATSLTMIVAGSETTMVTLTWAVCLLLNNPEVLNKAQDELDMH
IGKDRQVEESDIKNLAYLQAVIKETMRLYPVAPLLIPHEAMEDCTIAGYHVRAGTRVILNGWKLQRDPFI
WSDPCEFQPERFLNNDVDVRGRHFELLPFGSGRRACPGISLALNVVSLTLARLLHEFKFSNPSEGKVDMS
ESAGVVVAKATPLEVLIIPRLPSKFYEYYKL
>JDICYP82-2
MFSLQLLSTPLFGFFTLLAIYYLLWTKSNKTKEAPQPAGAWPIIGHLHLLARSELPHITLGAMADKYGPA
FMIRLGTRQAIVVSNSEIAKECLTIKDRIFATRPSLVAFKLMGYNSAMVGLSPYGPYWRELRKIIALKVL
SQRRLESLKDVWDSEISSSLNHLHQIWSHQTEPNGKILVEMDQWFGDLTLNVAVKMVAGKRFFSADAACD
ENESRRCQKAIREFFRLLGQFVVSDSLPFLGWLDLEGYQKEMKSTAKEVDSILQKWLDEHKQKRQSAGAD
RDQDFMDVMLSLLEDASLSQYDTDTINKATCFSMIVGGSDTTKITLTWALTLLLNNPDVMKKSQDELDIQ
VGKDRHVEESDIKNLVYLEAIVKETLRLYPAAPLLAPHEATEDCIVAGYNVRAGTRLIVNAWKIQRDPLI
WPNPSEFLPERYLNKDVDVKGQHFELIPFGSGRRACPGITFGLQVVSLTLARLLHEFEISIASGIKVDMT
ESGGLVTAKATPLKALIAPRFLYSHHHI
>MAQCYP82-1
MNSLQFLATPLLTLIALLCVYCIVWRKSFSRNGSKIKQAPWAGGAWPIMGHLHLLAWGDLPHITLGALAD
KYGPVFMMQLGMRQAIVVSSAEIARECFTTNDRILATRPSSIAVKLMAYNYAMFAFAPYGPNWREIRKTV
MLELLSTPRLESLKHVWEDEISTSVKELYELCASKTKTHGHVSVDMKQRFGDLTLNVLVKLVNGKRYFGA
NTDYTEHESRRCQKAVRDFFSLMGLFVVSDFLPFLERLDLQGYEKEMKRVAREVDGIVQGWLDEHKRKRE
SSETTHDQDFMDVMLSILKDSKFSNSDYDADTINKATCFNMILGGSDTTMSTMTWALSALLNNPHMLKKA

QDELEFHVGKGRQVEESDIKKLTYLQAIIKETLRLYPSAPLLAPREATEDCKIAGYHVPAGTRLIVNVWK
IHRDPFVWPNPNEFQPERFLNKDVDVKGQHFELIPFGSGRRACPGVSLGLQVVSLGLARLLHGFELSIPS
GSKVDMTETASLVTFKATPLEVFITPRLSPHMYVE
>MAQCYP82-2
YAWKRKNHTKDSKIKEPPQPAGAWPIIGHLHLISRGGLPHINMGAMADKYGPVFMIRLGVNRAVIVSNSE
IVKECFTTNDKFLLNRPVGLALNLMSYNNAMFGFSRYGPYWREIHKIVMLELLSSSRLESLKHVWDSEIL
TSIEELYQLCQTQNKAHEGPVLVEMKDWFADMALNVSFRMVAGKRYFGASAGGNKDEARRCQKTMRDFFR
LVGLFIVSDALPFLRWLDLGGHQKEMKRTFEDLDYVLQGWLDEHKLNRKSGGNGDQDFMDVMLSTLYDSK
FTDYDVDTINKATCFQLILGGTDTTTVTLTWALSLMLNHPHVLKKAQDELDIQVGKHRQVEESDIKNLKY
LEAVIKETLRMRPVGPLLGPREAIEDCTIGGYRVRAGTRVMVNAWKVQRDPSVWPNPDDFKPERFLKKDI
DFRDQNFEFIPFGSGRRACPGISFAVELLPMALARLLHGFELKTQLGCKVDMTEHAGLVHAKATPLEVLV
SPRLSRELYVSS
>MCACYP82-1
MDSTDHHLALVIPTLLATPVLLFSFYCLLLRPRSLKKPRTNKPPEPSGSWPIIGHLHLLSQGLPHITLGA
MADKLGPAFSIRLGTRRALVVSSWEVAKECYTTNDRAFASRPSSIAVKLMSYNNSILGFAPYGQYWRELR
KIVVLQLLSNRRLESLKNVWESEIDLSIKELYDTWASNRGGEALMVDMKQWFGDLTMNIVVRLVVEKRCF
GRSVGDDETEARRGQRALKEFLKLVGLFLVEDAFPFLSWLDVQGHQREMKRIARELDSLFQGWLEEHKRK
RSIKGEAKGDQDFMDVMLSVLEDSTISTDIEDDTVIKSTCLTVVLGGSDSTVGTLTWALSLLLNNRHELK
KAQDELDAYVGKERQVDESDIKNLVYLQAIVKEVLRLYPAGPLSGPRESVEDTVVAGYHVPKGTQLIVNL
WKIQREPSIWADPLEFQPERFLTTHKGMDVWGQHFELIPFGSGRRSCPGTAFALQVIHLTLARLLHGFEL
TTPCGAPVDMSESAGLINVKTTPVEVHVAPRLPLKLYKS
>MCACYP82-2
MILIAMGNHIELQDIILYSLGFFAATILWRIFSTYVLRRNSCSRPPEAAGKLPLIGHLHLLGANKILHHT
LGDMADRHGPIFSLNLGIKRTLVITSWEVAKECFTTQDRVFATRPKSLVGKVVGYNSTVMIFQQYGPYWR
EIRKLAMIELLSNRRLEMLKHVRESEVNLFIKELYEQWSSNENGSKVVVEMKERFGDLTTNIVVRTVAGK
KYSGTGVHGNEESRRFQKAMTDFMHLAGLFMVSDALPLLGWIDTFKGYRGKMNKTAEEIDHVLGSWLKEH
QQKRKNISINHLDEDFIHVMLSAMDGNQFPDIDTETAIKGTCLSLILGGYDTTSATLTWALSLIVNNHHV
LKKAQDEMDKYVGRDRQVKESDVKNLTYLQAIVKETLRLYPAAPLSVQHEAMEDCTVAGCNIPAGTRLVV
NLWKMHRDPKVWSDPLEFQPDRFLQKHVNVDIWGQNFELLPFGSGRRSCPGITFAIQVLHLTLAQLLHAF
QLGTVFASPIDMTESSGVTNPKATPLQVTLTPRLPPEVY
>MCACYP82-3
MDSTYQLLSALLFILCLYYLVCKPITKTKNDEAPVPAGAWPIMGHLHMLRGPNLPHITLSSMADKHGPAF
TIRLGTRRALVVSNSDLAKECFTVNDKAFSTRPSTVATKLMGYNNTMFGFAPYGPYWRELRKIVMTELLS
SRRLESLASVWASEIESSVKELFAEWAENRAKGPVVVEMGEWFGNLTLNIALRMVVGKRYLREESRKCLK
AMRDYPELFGRFLVEDAVPFLGWLDLIQGYQREMKKTARELDSVLEKWLVEHKDKRGSGEGKGEHQDFMD
VMISVLEDSKLSYGEADTVNKSTCLNLILGATDTTMVALTWALSLLLNNKQVLKRAQQELKSQIGNNKQV
SQSDIKNLLYLQATVKEALRLYPPEPLSGPREALEDCTVAGYRVRAGTRLIVNLWKIHRDPSIWQVPLEF
RPERFLTAHKDVDVWGQHFELMPFGSGRRSCPGISFVLQVVPLILARLLHGFELTTPGEALVDMSESAGL
VNAKVTPLEVVISPRLPLELFAG
>MCACYP82-4
MIMMFIDYYSSWLPQTLLLQSILLAVSLVIFINLFLTRRRSYSSKSHTNIIHPPKAAGALPVIGHLYTLF
RGLSAGVPLYRQLDAMADRYGPAFIIHLGVYPTLVVTCRELAKECFTTNDQTFATRPSTCAGKYIGYNYA
FFGFAPYGPYWREARKIATVELLSNYRLDSLRHVREAEVGRNVDELYALHASSSTNKQNMMKIDMKQWFD
QVTLNVILMMVVGKRCVTTGGNEEEVRVVKVLHEFFKHLGTLSVSDVVPYVEWMDLDGNIGRMKSTAKEL
DCILGRWLEEHRRERRSDFMDAMLAMVEGIKIPYYDSDTVIKAICLNLLNAGSDTLGITMTWALSLLLNN
RHVLKKVKDELDVHVGKNRQVEELDVKNLVYLHAVVKETLRLFPPAPLGVPHEAMEDCVVGGFHVAKGTR
LVVNVWKLHRDPSVWSDPLAFKPERFLDNNTVDVRGQHFQLLPFGSGRRGCPGITFALQVAHLTLARLLH
GFEWDTPDGAPVDMSEVSVLTTAKKNPVEVLFTPRLPAEVYTQN
>MCACYP82-5
YQLLFLQTAIAGLLFALALLVRLRFSRRNNGKKKQAPPEPAGARLIMGHIHMLNSGEQPHKTLAALADQY

GPVIKLRLGLRDAIVVSNWEMAKECFTTNDKIFLSRPQTVVSKFMSYNLKMLGFAPYGPYWREIRKIVVS
ELLSNRRLELLKNAWTSEISSWVKELFDEWGAKEARGPVVVDMRHWFGNLAFNIGMNMVAGKRFFGPRVV
SDEDGLRRVQQGLTDFFRLIGAFLLEDAVPFLSWWDSQGYQKEMKDTAREMDTLIQGLLDEHKRRRSSSS
GEAKGEKDFMDVMLNILEGSKFPSGDADNINKSTCLSLMLGLTDTTTATLTWALSLLLNDPRVLKKAREE
LDFHVGKNRLVDTSDLMNLPYIHAIVKEVLRLHPPAPLSGHRESLEDSVVGGYHVPKGTRFMVNVWKIHH
DPNKWPDPIEFRPERFLTTHKDVEVWGQNFELIPFSSGRRVCPGASLSLQVLPFTLARVLQGFEVATPGG
APVDVTESKGLSTAKETPLDVVITPRLPEKFYH
>NDOCYP82-1
MDSLISFQAIVGLFILILVSYQWLGRSRSIKTNKHNEAPEPAGRWPIIGHLHLLGGSDQLLYRTLGSMAD
KLGPAFNIRLGSRRAFVVNSWEVAKECFTINDKALASRPITVAAKLMGYNYAVFGFAPYSPFWRAMRKIA
TLELLSNRRLEMLKHVRISEVDMGLKEIYGLWSKNKDSGPLMIELNRWFENLTLNMVVRMVAGKRYFGAD
ASCDENEAQRCQKAISEFFRLIGIFVVSDAIPSLWWLDLQGHEKAMKRTAKDLDSILGGWLEQHRSRRVN
GKVPTEGEQDFIDVMLSLQEGDHLSDFEYDADIAIKSTCLALILGGSDTTAGTLTWAISLLLNHPYALKK
AQEELDLHVGKDRQVYDQDIKNLVYLQAIIKETLRLYPAGPLLGPREAMEDCTISGFDVRAGTRLVVNVW
KIQRDPNVWSNPSEFSPERFLTSHVDVDVRGQNFELMPFGSGRRSCPGASFALQVLHLTLARFLHGFGLA
NPLGKPVDMNESPGLTIPKATPLNVLLTPRLDSELYGC
>NDOCYP82-2
METFQFLGIPLFGFFALLCTYYLVLRKPSSTKIREPPRPAGAWPIIGHLHLLARGDLPHVTLGKMADKYG
PVFKLKLGVRQAIVVSDWEVAKECYTTNDRALANRPSGLGAKIMGYNYALIGSAPYGPYWRDLRKIITLE
VLSSRRLESLKHVWDSEISTSVKELYQIWADQNKAQGQVSVEMKQWFSDMTLNVAVRLAVGKKYLGATAD
CEKNKAVQCQKAIRNFFRLAGLFVVADYLPFLGWLDLGGHEKEMKHTAKELDYIAQEWLDDHKKKRTSNR
TVDEPQDFMDVMLSILEESTFTGYDVDIINKSTCFALILGGTDTVAVTLTWALCLLLNNPHVLKKAQDEL
DIHVGKDRHVNESDIKNLVYLQAMIKETLRLYPAGPLLGPRQVIEDCTIAGYHVRAGTRVIVNAWKFQRD
PSIWSNPCEFQPERFLDKDIDVKGQHFELIPFGAGRRACPGISFALQVLPLALARLLHGFELKNPSESQV
DMTETPGMVHAKTTPLEVLITPRLSPKFYV
>NDOCYP82-3
WKRPTSVKYREAPQPAGAWPIIGHLHLLARGDLHHITLGAMADKYGPAFMMRLGVHQAMVVSDSESAKEC
STTNDRVLATRPSSVAVKLMGYNYAMFGFGPYGSYWREIRKIVILEVLSNHRLESLKHVWKSEISMSTKE
LYQLWINQNKDEGPTLVELKQWLCNMTLNIGVRTVAGKRYFGASSCDANESNRIQRAIRDFFRFVGLFVV
SDFLPFLGWLDLEGYQKEMKSIARELDSILQGWLDEHTRKRQSGGTNENQDFMDIMLSVLEHSNALTHYD
ADTINKATCFTMILGGSDTTMVTLTWALSLLLNNPHVLKKAQDEIDIQVGKDRLVDESDIKNLVYLQAIV
KETLRLYPAVPIITPHEAIEDCTIAGYHVPAGTRLIVNAWKIQRDPLIWDNPGEFQPERFLYKDVDVKGK
HFELIPFGSGRRVCPGISLALQVVSLAMAHLLHEFDLAKPSEGNVDMTESVGLVNAKATPLEVLITPRLS
PKFYE
>NSACYP82-1
MGSFNQINPSVVYFAAFTIFFFFFLYKILYKRRTSAKTRVAPEPAGAWPFIGHLPLLSQQNLPHVTLGVL
ADKYGPAFTVQLGLHKAVVVSSWEVAKECFTVNDKVLATRPSSVAVKIMCYNYAVFGFGPYGSYWREARK
IVVRELLSNHRLNLLKHVLVTEISMSMNELYTVWQKNANNVSGKALVEMKQWFGDLSLNMIVKIVAGKRY
FQASANSDERDQLKRGVQDLFHLVGLFLVSDALPFLSWLDIGGHEKAMRRTAKELDNILGSWLDEHKQAK
LAGATKGEQDFMDVLMSILEDNNNLPEYEPDVVSKAICLGMILGGADTTTITLTWIVSLLLNNQHILKKA
QDEIDSKVGKDRQVNESDIEKLVYLQAIVKETLRLYPPAPLSTQHEAMEDCTIAGYHVQAGTRLITNIWK
IQRDPGVWPNPCEFQPERFLTTHANVESNGKHFEFIPFGSGRRACPGMSLGLQVVHLTLARLLQGFELET
PLSVEVDMTESAGLTNLKVTPLEVLINPRLPSNLY
>NSACYP82-2
MDLTSILICIFLFIISICFLSWKRSRSLSGTRAAPEPVGAWPVIGHLRLLMGSKTPHMTLGNLADNYGPA
FTIRLGTKKTLVVSSWEVAKECLTTNDKAFAGRHSTMAVEIMGYNYAFFSLGPSGQYWREIRKITVSELL
SNRRLELLKHIWSSEIKTSIKELYEICAAQSQYGKKQSPMVEMQQWFSHLNLNLSARMVVGKRYFADGVE
ENEGDIRRFQNAIKEFFYWAGAFIIADTIPYLRWMDFLNERSMKRTAKETDHVLEGWLQEHKRNRLNGVT
KEQDFMDVLLTELGDKDLCGYTADVVNKATCLNMIFGGTDTTKTGMIWTLSLILNNPQVLKKLQNEIDIQ
VGKDRQVDDSDIGNLVYLQATVKEAMRLYPPATIFGRESIEDCTVAGYHIQAGTRLIVNAWKIQRDPNVW

PNPDEFQPERFLTNQADVDFRGQDFELIPFGAGRRACPGISLTVRVTHLTLACLLQGFNFETPLNEPVDM
SEKYGLTNSKATPLNVILTPRLPSNLY
>NSACYP82-3
MDSIFHMYAPINILAATFVFIVISVWFLLWMKRSRKNSKSRRAPEPVGGWPLIGHLRLLVGPELPHVILG
SLADKYGPALSIRLGMRQALVVSNCEVVKECLTTNDRCFSTRPSSVAVELMGYNYASFGLGPYGGFWREV
RKIAILELLSNHRLKSLKHVWISEISGFVKELYQLCVANGNTGRQSALVEMRQWLNDLTLNVSVRMVVGK
RYFGSGSEHGGEDVKRFQKAIKDFFRLAGKFTLADAIPFLRWLDFGHERAMKKTAKELDYVLARWLDQHQ
KNKLNCETKQVDQDFMDVMLTVLGDKDLYGFKADVVNKATCLNLILGGVDTTSVTLTWALSLLLNNPNIL
NKAQEEIDVQIGKDRQVDDNDLGKLVYLEAIVKETMRLYPAGPLSGARAAIEDCMVAGYHVPKGTRLIVN
TWKIQRDPEKWDNPSEFRPERFLITTTNGHVEVWGQHFEYIPFGSGRRICPAISFSLQLVHLTLARLLQA
FSFQTPSNAAVDMTETPGLVNFKATPLQILLTPRLPSSLY
>NSACYP82-4
MLSIHDSTMVFLQLQAICGIFGFIFIITWWTRWKSSNKMKAPEVAGAWPVIGHLHLLGGGRPLYQLLGDM
SDKYGPAFTLRMGIQKALVVSSWEVAKECLTTNDRALATRPSSAGGKYMGYNNALIPFSPYGPYWRDMRK
IATLELLSNHRLEELKHVREMEINTCISDMYKLCQVEDGVEIKPISVDLSQWFADLTFNVVVMMITGKRY
IGSTDAGDMNEIRHFQAALVKFMRLLRISLLVDVFPVLQWINYGGFKGVMKSTARDIDSVLENWLQEHQR
KRLSPDFNGNHDFIDVMISTLEGTEFSDYDHNTIIKAISMAMVVGGTDTTTTTLIWAISLLLNNPNAMKK
VQEELEIHVGKERNVDGSDIQHLVYLQAVVKETLRLYPPVPLSVMHQAMEDCVIGSYNIQAGTRVLFNLW
KLHRDSSVWSDPLEFRPERFLTSHVDVDVRGQHFELIPFGSGRRSCPGISFALQVIHLTIARLFHGFNLT
TPGNSSVDMSEISGATLSKVTPLEVLVTPRLSSKLYN
>NSACYP82-5
MDFLIYVAAFAAAFFFLLFYNILSKRITGTKRRIAPEPAGAWPFIGHLPMLFGPNLPHVTLGALADKYGP
AFTIQLGLHKALVVSSWEVAKDCFTVNDKALATRPTSAAVKIMAYNCAVFSFSPYGSYWREARKIAILEL
LSHHRLDLLKHVRKSEVSTSIRELYQFWQKNATEVTKFAQLEMKQWFGDLTMNLIVRMVAGKRYASVQSD
DDGEGKRLQKAVNDFFHLVGLFVVSDALPFLGWLDIGGNLKTMKKTARELDSIMECWLKHQARRSNGEQD
FLDVMLSILREDNKLQEYDGDVVVKAMCLNMILGGADSTTITLTWTISLLLNNRHILQKAQDEIDSKVGK
DRQVNESDIEKLVYLQAIVKETLRLYPPAPLSSQHEAIEDCTIAGYHIPAGTRLITNLWKIQHDPSVWHN
PSEFQPERFLTTQANVDFRGQNFEFIPFGFGRRSCPGTSLGLQMVHLPLARLLQGFTFETPSNAAVDMTE
SAGLTNHKVTPLEVLISPRLRFDLYQESPP
>NSACYP82-6
SFNQTYTGFICLSVFASFFFFFLYSVQQKRRTGRKARYAPEPTGAWPLIGHLPILFEPNLPHIQLGALAD
KYGPVFSIKLGLRKALVVSSWEVVKECFTVKDKVLASRPSSVAVKIMGYDYAVYAFGPYGSYWREARKLT
ILELLSDHRLDLLKHVRVSEVSMSMKELLKFCQKNANNVSGKALLVDMKQWFGDLTLNVIVRMVAGKSYL
GASAKLEDGEQKRLQNAIHEFFRLAGLFVVSDALPILSWLDIGGHEKAMRRNAREIDKIMSSWLDEHRRN
KLAGVTNGEQDFMDVLLSKFENSNLPGYEPDVAIKAICLNMILGAADTTIVTLTWVVSLILNNRQILKKA
REEIENKVGKDRQVNESDIEKLVYLQAIVKETLRLYPAAPLSAHHESMEDCTIAGYHVPAGTQLITNLWK
IQRDPRVWPNPCEFLPERFLTTHANVNYKGQNFEFIPFGYGRRSCPGMSFGLQMVHLVLASLLQGFELET
PLNEAVDMTESAGLTNLKATPLEVLITPRMPLNLY
>NSACYP82-7
VTVIYLFTFAIFFYLYSILWKNPRKSAKTRVAPEPAGAWPFMGHLPMLLEPNLPHIKLGALADKYGPAFT
VQLGLHKALVVSSWEVAKECFTVNDKVLADRPSSVAVKIMGYDNAVFAFGPYGSYWREARKISTVELLSN
HRLSLLQHFRISEASMSMKELYQLCANNGKALVEMKKWFGDLSLNVIVRMVAGKRYAKASEDDERRQLQK
GLKDFFYLVGLFVVSDALPILSWLDIGGHEKAMRRTAKELDTIMERWLDEHRRAKLAGVTKGEQDFMDVL
LSILEDGKLPHYESNTVNKAICLTMILGAAHTTTVTLIWTLSLLLNNRHILNKAQDEIDSEVGKDRAVTE
SDIKNLVYLQAIVKETLRLYPAAPLPAHREVMEDCIIAGYHVPVGTRIITNLWKIQRDPRVWSNPCAFQP
ERFLTTHSNVDFRGQNFEFIPFGSGRRSCPGISLGLQMVHLALARLLQGFELETPLNLGIDMTESSGLTN
LKVTPLEVVITPRLPSNLY
>PBRCYP82-1
MAYLMIKKSFHLFSDQPTSVSTLIVLAFLLTLSPVIIYYEQKKRGLRRNRTSSSCTAITTTPLPEASGAW
PVIGHLLLFMNENDLNHATLGNMADKYGPIFSLRFGSHRTLVVSSWEMVKECFTGANDKFFSNRPSSLAV

KLMFYDTESYGFAPYGKYWRELRKISTHKLLSNQQLDKFKHLRISEVDNSFKKLHDLCSNNKLGGETTSV
ANLVRMDDWFAYLTFNVIGRIVSGFQSNAVSGATSSQEKYKLAIDEVSNLMATFAVSDVVPCLGWIDRLT
GLTGKMKKCGKKLDAVVGDAVEVHRQKKLKISRNTAGELTEHEEEDFIDVCLSIMEQSQIPGNNPEISVK
SIALDMLSGGSDTTKLIMTWTLSLLLNHPDILDKAKEEVDTYFRKKKISDNTPVVDAADVPNLVYIQAII
KESMRLYPASTLMERMTSDDCEVGGFHVPAGTRLWVNVWKMQRDPRVWNDPLVFRPERFLSNDKGMVDVK
GQNYELIPFGTGRRICPGASFALEVLHLVLTRLILEFEMKAPEGEIDMRARPGFFNNKVVPLDVLLTPRT
LD
>PBRCYP82-2
MNNYSSSPASSTETAVLCHQRQQSCALPISGLLHIFMNKNGLIHVTLGNMADKYGPIFSFPTGSHRILVV
SSWEMVKECFTGNNDTFFSNRPIPLAFKIIFYAGGVDSYGLALVPYGKYWRELRKICVHNLLSNQQLLKF
RHLIISQVDTSFNKLYELCKNSEDNQGMVRMDDWLAQLSFSVIGRIVCGFQSDPKTGAPSRVEQFKEAIN
EASYFMSTSPVSDNVPMLGWIDQLTGLTRNMTHCGKKLDLVVESIINDHRQKRRFSRTKGGDEKDDEQDD
FIDICLSIMEQPQLPGNNNPPKIPIKSIVLDMIGGGTDTTKLTTIWTLSLLLNNPHVLDKAKQEVDAHFL
TKRRSTNDAAVVDFDDIRNLVYIQAIIKESMRLYPASPVVERLSGEDCVVGGFHVPAGTRLWVNVWKMQR
DPNVWADPMVFRPERFLSDEQKMVDVRGQNYELLPFGAGRRICPGVSFSLDLMQLVLTRLILEFEMKSPG
GEVDMTATPGLMSYKVLPLDVLLTHLSAS
>PBRCYP82-3
MEFLMKLLLLLEPITFSIFLGIGSIVLLYNVFFLVINKKKKKKAPNASGAWPLIGHLNLFMNDKEALYKT
LGTMADKYGPAFNVRLGNQEILVVSNWKMVKECFNTQNDKLFSNRRTTLGVKYMLNKKTSVAFSPYGTYW
RELRKLTVQQLLSKQRLDSWKHLKIKEIDASFGRLNDLCSNNKGTGAATPIRMDSWFAELTFNVFARIVF
GYQSGERLMLSGDTASNGERYKKTLEEAFLLMSSFAVSDVFPCLEWVDRSRGLVRSMKRFGDQLNSIAGC
LIEEHRQKRSQSVSASNSTNDKGVGDEQDFIDVLLSVAELSQIPGDDPDLVIKSMILEALAGGSDTTTST
LTWVLSLLLNHPKVLKTAKEEIDMHVGRNRCVEESDIPKLVYVNAIIKESMRLYPNGSLVDRLTLEECEV
GGFHVPAEGHLFVNVWKIHRDPSVWENPLEFKPERFLSNDCKVDMDFISQKYEFIPFGIGRRICPGMLSA
LQVMHFVVARLIHGFDMEAASADGKVDMAEKPGMTCYKMTPLEVMLTARQ
>PBRCYP82-4
MYPVNQLQSQAIAVLCAVIVFIFYLGRKLFNSTHIHKPGKTAPEPAGAWPIIGHLHLLGGAKLMYRTLGS
LADEYGPVFMVRLGMRRVLVISNTASARECFTTNDKVFATRPTTVAIKLLTYNHTMFGFAPYGPYWREIR
KIATTELLSDRRLAMLKNVWISEINLCINELHQLLMDKNSRKVDHHQHVYNQNMDPISVEMNGWFADLSF
NVVARMIAGKRYFGKNTDGGEEETRRYREAMNDFMHLVVILLVSDAVPFLGGLDFQGYKKRMKKTAKEID
YFLGKWVDEHRQRLKYKNISKVDQQDDFIDVLLLNLNDQPIYGRDTDTIIKSTCLSLIAGGSDTTAVTLT
WALSLLLNNQHVLRKAQDELDIHVGRERQLEESDIKNLVYLQAIVKETLRLYPAAPLSAPRMAMEDCTVA
GFQVSKGTQLMLNVWKLHRDPHFWGPDPLEFRPERFLTADGSSTGSGHCIDIDVKGRHYELLPFGSGRRM
CPGVSFAMQVVHLTLATLLHGFHLSTPTDGPVDMTETSGLSCPKATPLEVLLTPRLPC
>PBRCYP82-5
MDVAIIVDHHYLQPFVSIAGLLALLSFFYCIWVFIIRPRIIKSNLDERKLSPSSPPEVAGAWPIVGHLPQ
LIGSTPLFKILADMSNKYGPIFMVRFGMYPTLVVSSWEMSKECFTTNDRLFATRPPSAAGKYLTKALFAF
SVYGPYWREIRKISTIHLLSLRRLELLKHGRYLEIDKCMKRLFEYWMEHHKNIISTTSSVKVNMSQVFAE
LSLNVVLKIIVGKTLFIKNGNEDYTKEEEEGQKLHKTILKFMELAGVSVASDVLPFLGWLDVDGQKKQMK
RVYKEMNLIASKWLGEHRERKRLQIIQKRGAARGSNYDDGNDFMDVLMSILDEENDDLFFGYSRDTVIKS
TCLQLIVAASDTTSLAMTWALSLLLTNPNVLQKAQDELDTKVGRDRIIEEHDIECLVYLQAIVKETLRLY
PPAPLSLPHEAMEDCTVGGYQVKAGTRLVVNLWKLQRDPRVWSNPLEFKPERFLPQSDGGFGGEEARMDF
RGQHFEYTPFGSGRRICPGIDFFLQTVHMALARLLQAFDFNTAGGLVIDMVEGPGLTMPKVTPLEVHLNP
RLPVTLY
>PBRCYP82-6
MQVDWPNILQKYYPIITCSLLTLLSFYYIWVSITKPSRNSKTKLPPPEVAGSWPIVGHLPQLVGSTPLFK
ILANMSDKYGPIFMVRFGMHPTLVVSSWEMSKECFTTNDKFLASRPPSASAKYLGYDNAMFVFSDYGPYW
REIRKISTLQLLTHKRLDSLKNIPYLEINSCVKTLYTRWAKTQSQIKQNVGGAADDFVKVDMTEMFGHLN
LNVVLRLVVGKPIFIQKDNADEDYTKDGHNKEELGQKLHKTIIEFFELAGASVASDVLPYLGWLDVDGQK
KRMKKIAMEMDLFAQKWLEEHRQKGINHDNENDFMAVLISVLGEGKDDHIFGYSRDTVIKATCLTLIVAA

TDTTLVSLTWALSLLLTNPRVLSKAQDELDTVVGKERNVEDRDVNHLVYLQAVIKETLRLYPPSPLAVPH
EAIENCNVGGYEVKARTRLLVNLWKIHRDPRVWSNPLEFKPERFLPKLDGGTGEASKLDFKGQDFVYTPF
GSGRRMCPGINFASQTLHMTLARLLHAFDFDIESNGLVIDMTEGSGLTMPKVTPLQVHLRPRLPATLY
>PBRCYP82-7
MMDLAMFIDQYFSLAKIAGLLALLSFFYYLWISTLWSPRNPKLSSVSPPEVAGAWPILGHLPQLLGSRPL
FKILADMSDNYGPIFMVRFGMHPTLVVSSWEMAKECFTTNDRFLAGRPSGAANKYLTFALFGFSTYGPYW
REIRKIATLHLLSHRRLELLKHVPDLEVTNCMKHLHRRWIDSQNQIKQNDAAAGSVKVDMGRVFGELTLN
VVLKLVAGKSIFFKNDNTRQYDSKDGHNKEEEEGKKLHKTIIDFYSLAGASVASDVLPFLGWLDVDGQKK
RMKRVAKDMDFIAAKWLEEHRHQKRQTVLSSSATLGSSNHDDAKDFMDVLMSILDGENDDLFFGYSRDTV
IKTTCLQLIAAAADTTSVTMTWALALLITNPTILRKAQDELDTKVGKDRNIEERDINDLVYLQAIVKETL
RMYPAGPLNVPHEAIADCNIGGYEVRAGTRLLVNLWKMHRDPRVWSNPSEFKPERFLPQLDGGSGGEAAN
LDFRGQDFEYLPFSAGRRMCPGIDFSLQTLHMTLARLLHGFDFNNDSAGIIIDMEEGSGLTMPKLTPLEI
YLCPRLPAKLY
>SCACYP82-1
MEFLSLQFQPITIFALLLATPIFLYNLWNYGRKLNNKNKKSRKPPAPEASGGWPIMGHFHLFNGTEVTHR
ALGSMADKYGPAFNIRIGSHPTLVVSSWEIAKECFTTNDRLFSNRPGSLAIKLMFYDADSVGYAPYGTYW
RELRKISTLKLLSNHRLETLKHLRTSEVESCFKQLHDLWRMNNKKGGSDDGSDFALVRIDNWFGDLTFNV
VARIVAGKKNFAGGAASGDAGAQRYKEAMDEAFRLMTRFAFSDVVPSLGWLDKLTGLVGGMKRCGSEIDS
IVASWVDEHRLKRISGKEGDDLEQDFIDVCLEILEHSTLPGDDPEVVIKSTCLDMILGGSDTTTVTLTWA
LSLLLDHPHVWKRAKEEVDAHVGKERQVEDSDIPNLVFIQAIVKETMRLYPAGPLIERRTMDDCEVGGFH
IPGGTRLIVNLWKIQRDGSVYKEDPLEFRPERFLTSNAEVDLKGQNYELIPFGAGRRICPGVSFAVQLMH
LVLARLIHDFEITMPMGAKVDMTESGGLISHKVTPLEVLLKPRLPSLQQAALY
>SCACYP82-2
MEYSSVLLHWFPASMAALLALVFLYNLIFNSPKTGNKKIIRRAPKAAGAWPVLGHLHLFGSGELPHKMLA
AMAEKYGPAFTMKFGKHTTLVVSDTRVVKECFTTNDTLFANRPSTKAFDLMTYANDSVAFTPYSPYWREL
RKISTLKLLSNHRLQSIKEVRVSEVSVCFRELYDICKNDGGGAPPVLVDMKKWFEEVSNNIVMRVIVGKQ
NFGSKIVCGEEEAVNYKNVMDELLRLASVSMLSDVAPLLGWLDMFQGHMSAMRRNGKKLDTILERWLEEH
RKKKSEDEQDFMDVMLSIVEETKLSGRDADTVIKATCLAMIMGGTDTTAVSLTWIVSLLMNNRYALKKAR
EELDALVGKDRQVEDSDLKNLVYLNAVVKETMRLYPLGTLLERDTKEDCEVGGFHVEGGTRLLVNVWKLQ
RDPNVWNDPLEFRPERFLTENADIDVGGQHFELLPFGAGRRVCPGVSFALQFMHLVLARLIHGYELGTQN
DEDVDLTESTEGHVNHKASPLDLLITPRLHPKLYE
>SCACYP82-3
MDYSSHLNQFYGVAPYMAALLIALVFLYNILWASPKTTNKKPIMAAGAWPILGHLHLFKDGELPHHMLKS
MSDKYGPAFLMKFGQHRTLVVSNYRMVKECFTTNDTHFCNRPSTTAFDVMTYANDSVAFTAYSPYWRELR
KISTLKLLSNNRLQAIKNLREAEVNVSFRGLYDLWKNNSDRAASVLVDMKKWFEEVSNNVVIRVIVGKHN
FGTKIVKGEKEAVEYKTIMDELLRLASLSLLSDFAPILGWVDFFQGNVRKMKRNGKKLDVVLQRWLETHR
RKKNSPEDEQDFMDVMLSIVEEGDKLSGHHADTVIKATCLAMIMGGTDTSAVSLTWIVSLLMNNRHALKK
AREELDMYVGKDRQVEDSDLKDLVYLHAIVKETMRLYPLGTLLERETKEDCKVGGFHVEAGTRLLVNIWM
VQRDPTVWSDPTKFIPERFLTEKADIDVGGQHFELIPFGAGRRVCPAVSFALQFLHLILARLIHGYELGT
PNDADVDLTESPEGHVNHKASPLELLLTPRLDPKLYI
>SCACYP82-4
MNSIAVVGVLLFFLYYLWRTLFTTHKAAPPPQPAGARPIIGHLHLLGGANQLVHQTLGSMADKYGSIFMI
RLGMRRVLVVSSSELAKECFTTNDKVFLNRPSSLALKLMAYNNAIFGFAPFGPYWRKMRKIAVTELLSNR
RLEILKNIRISEINMSIKELHQLWVDMNSGDDQVGGPPIVVEMRKWFGDLSFNVVGRMIAGKRYFGRNCD
CDEEETRRYQKAMGDFMHLVGVFVVSDAIPLLGFLDLQGYEKKMKRTAMDIDCVLGRWVDEHRRRRQDHD
EKRIMVSDDDHQGQDFIDVMLSILNDDDQFYGYDADTVIKSTCLSLIAGGHETTAVTLTWALSLLLNNRH
VLQNAQDELDIHISKERQVDESDIKNLKYLQAIVKEALRLYPALPLSALRLAMEDCTIAGFHVPKGTELM
LNLWKLHRDPHVWSDPLEFKPERFLTNSTHEEVDVGGQHYELLPFGSGRRMCPGVSFALQVLHLTLARLL
HGFHLSTPSGVPVDMTQSFGLSCPKATPLDVLLTPRLPSKLYYVA
>SCACYP82-5

MISTTPDVLATSSYSSSNKHMDSDHLYRFSTSTSIVIGLFILLFFLVLLWKPKKQSATSKTNKPTREQRA
PEPSSSWPIIGHLRLFGGPNSLPHITLGAMADKYGPVFTIRMGSRPVLVVSSWETAKECFTTNDRVFASR
PRTIAIKHMCYEHVMLGFAPYGSYWRELRKIVNRELLSNNRLELLKHVWGSEINTSIKELYELWLVNKNN
IEAEGENEGTSDRVLVEMERWFADLTLNMSIKIVVGKRYPSGVTAGGSSGCDDDEAQRCRKALRDFFKLV
GLFLVSDAIPFLRWLDLGGYEREMKTTARELDCLMEEWLEEHKMKRRLLISPSAGDHHEAAEIMINKQKE
AELQEYDFMDVMLSILDDDIEGKNLSDYDVNADTINKSTCLNLILGGSDTTMVTLTWALALLLNNPHVLQ
KAQDELDIHVGSKRQVDDSDMKNLIYLQAIVKETMRLYPAGPLSVPHESTEDCTVAGYHVPAGTRLIVNT
WKIQRDPRVWSNPSEFKPERFLTDSHVDIDVRGRNFELIPFGSGRRSCPGTSFALQVVHLVLARLIHGFE
FKTPSEAPIDMTESVGLSNFKATPLDVLLTPRLPSQLYYVS
>SCACYP82-6
MDSTLVFIGLFALLLVYPLLLRRSVLKSTSNTNKTTYKSKNQAPEAAGAWPIIGNLHQLAGGGNCLHKTL
GAMADKHGPAFTIRMGLHKALVVSSWELAKECFTTNDVVFMSRPHQVAIKHMGYSTAMFGIAPYGPYYRE
MRKIVTQELLSNRRLELLKHVWASEINNSIKQLYEKVEGGPVLIEMKRWFADLTLRTTVKVICGQQQFGG
DDDDDEAGRFQTALRDFFRLLGHFRVADVIPVLEWLDFQGYKKEMENNGRVLDNLMNKWLEEHKRKRKNG
GTEEDFMNVMISKLLDDTKMLSYYDADTINKSTCLTLILGGSDTTMVSLTWALSLLVNHPHVLKKAQEEL
DVHVGKERQVDDSDLWSLTYLQAIIKETLRLYPPGPLSAPRESTDNCTIAGYHVPAGTRLIVNTWKIQRD
PRVWPDPLEFKPERFLTTHVDVDVKGQNFELLPFGSGRRACPGASFALRVLRLSLARLIHGFDFKTPLDD
SPIDMVESGGITNVKDTPLEVLVTPRLPSSLYVCK
>SCACYP82-7
MDFDLYQFSKPTIFIGGLFAILLYFVLKKSSTPKSKSKLEQPPEPAGAWPIIGHLPLLGGPDLPHITLGK
LADKYGPAFTIRIGVHKAVVINSWEVAKECFTTNDKAFSSRPRQVAMKHMGYDYAMFGFAPYGNYWRELR
KIVNREVLSHSRIESLYHVWGTEINTSVKELYELWGKKSTGSGGAPVLVEMKRWFSDMTLNMSVMMVAGK
RYNFSGNKADDEAGRCQDGLRNFFRLVGLFVPSDALPFLAWLDIGGYEKEMKKVAKELDVLVQEWLEEHK
EKRLALTAAGKKGSENDFMDVMMNILEDQKLSEFDADTINKATCLTLILGGTDTNMVNLVWALTLLVNNQ
DALKKAHDELDFHVGKDRQVEESDVKNLVYIQAIMKETLRLYSGPLSGLRESTEDCTVAGYYVPAGTRLI
INAAKIHRDPRVWSDPTAFKPDRFLTEQKEMDVRGQDFEILPFGAGRRICPGVSFALQVLPLALARLIHG
FDFKTPTDAPIDMTESPGLTNAKSTPLEVLVSPRLTSKLYGC
>SCACYP82-8
MDSILNQFISKPTMVICSLFALLSLYFLLIKRSTTKSKLQLPPEPAGAWPVIGHLHQLGGPNLPHITLAA
MADKYGPIFTMKIGTYRALVVSSPEVAKEIFTTHDRIWATRPNQAAMKHLGYDSAMFGFAPYGPYWRELR
KLVNRELLSNTRLDLLHHVWDSEINTSIKELYDSLATKNNAKGRGTGGAGSTGPVLVEMKRWFADLTLNI
TVRMVAGKRYFGANKTSSTSTDQCDNEKGDSKAWKCQRELRNFFRLVGLFVVSDALPFLGWLDLGGHERE
MKNTARELDVLVQGWLDEHKRKRSLSVAEGGEKINGEQDFMDMMLSTIDDAKLSGYFDADTINKSTCLNL
ILGGSDTMMVNLIWPLTLLVNYPDELKKVYDELDTHVGRDRQVDESDIKNLVHLKAVIKETMRLYTGRIT
GLRQSSEDCTVAGYHVPAGTRLIVNTWKISRDPRYWSDPLEFKPERFLTTHADIELTGQNFELIPFGSGR
RSCPGASFAIQLLHLALARLVHGFWFERPSDAPIDTSECPGLTNFKATPLEVLATPRLPSKLYVG
>SCACYP82-9
MDFILNQFISKPTMVLGTLFALLSLYFLLIKRSTTKSKLQLPPEPAGALPIIGHLHLLGGPNPPHITLAN
MAEKYGPIFSIKMGLKRALIVSSSEVTKECFTTHDLNFVSRPRHAAMKLMGYNFATFGFAPYGPYWRELR
MIINRELLSQTRILSLEHVWGSEINESIKELYDLLTRKGGGGGGPVVVEMKRWFADLTLNIAVKMVCGKR
YFGVNSPSTSTTRDVQEDDEARRCQKGMRNFFRLLGQFVVSDSIPFLGWLDLGGYQREMKNTARELDDLM
QGWLEEHKKKRLERKTAAGEQDFMDVMLNILEDGKLSQYFDTDTINKSTCLSLILGGSDTTMVNLVWAFS
LLVKHQDALKKTRDELDIHVGRERQVEESDIKNLVFLQAVVKETFRLYAGPISGVREAAEDCTIAGYHVP
AGTRLLINSWKIHRDPRVWSDPLEFHPERYLEARHADIDVKGQNFELAPFGMGRRVCPGSAFGLQVLHLT
LARLIHGFEFKTPSDAPVDMTESVGMSIIKATPLEVLVTPRLPSELYV
>SCACYP82-10
MDISNLYELYYFSTPTLFVGGLFVLLLIYSLFSSRPGIKSKLQQPPEPAGAWPLIGHLPVLSGPELPHVA
LGKLADKYGPVFTIRMGVHKSLVVSDWEVIKECFTTHDKVFSSRPCQVAMKLMGTAMFGFSPYGNYWREL
RKIMNREVLSHGRIESLYHVWGSEINTSVKELYDLWAKKNNGGGPILVEMKRWFSDLTLNMSVMMAAGKR
YNFGDDSSTTNDEAARCQHALREFFRLVGLFVPSDALPYLGWLDIGGYQKQMKKVARELDDLMQVWLDEH

KKKRLAAKAEGKKGGEQDFMDVMMTILEDGKLSDYYDADTINKATCLTLILGGTDTNMLSLVWTLALLMN
NRQALKKVHEELDIHVGRERQVEESDIKNLVYLQAVIKEEMRLYSGPLSGLRETTEDCTIAGYHIPAGTR
LIINASKLHRDPKVWSDPLEFKPERFLTENVGVDVRGQNLELQPFGAGRRICPGVSFALQVLPLTLARLI
HGFELNTPGDAPIDMTESPGLQNAKLTPLEVLITPRLPSKLYV
>SCACYP82-11
MIVIGAFLALVVLVVYYNLLLRRPALLKFKNMKQQAPEAASAWPIIGHLHNLAGGIGGNGLLHEKLGAMA
DKHGPAFIIRLGLHKALVVSSWEVVKECFTTNDVALISRPHQVASKHMGYGMAMFAFASPYGPYWRELRK
IVKQELLSNSRLELLKHVWASEINTSIKQLYEKVKEGGGLPVLVEMKGWFADLTLKMTVKVICGKRHFRV
AGNVGDHVDADDDDEVIGGRFEKALRDFFRLLGDIRVVDVLPFLRWLDFGVGYKKEMENNGRVLDILMEE
WLQEHKRKRMNGGTEEDFMNVMISKLLNDTKLLSYYDADTINKSTCLTLILGASDTTMVTLTWALSLLVN
HPQVLKRAQDELDVHVGRERQVEDSDIQNLTYLQAIIKETLRICPPGPIQAPHEAKDDCTVAGYHVPAGT
RLIVNTWKIQRDPRVWPNPSEFKPERFLTTHVGVDVRGHNFELIPFGSGRRSCPGTSFALQVVHLTLARF
IHGFEFKTPSDAPIDMTESGRITNVKATPLEVLVTPRIPPSGLYV
>SCACYP82-12
MDLLIFSSQLQGTVGLLALLTFSYYVWILIIKRIKTTNTLKVMNKVAAAAAPEVAGAWPIVGHLPQLVGP
QQLYRILGDMADEYGPIFMVRFGMYPTLVVSSWEMIKECFTTNDRFLASRPSSAAGKYLTYDFAMFGFSF
YGPYWREIRKISTLELLSHRRLELFKNVPFTEIDTCIKRLYQLWRVKNNDHPAAAPVIKVDMSQLLRDLT
LNTILKLVVGKNLFNEKDHGEQEEGRKLHLHKTIVEFFKLAGVSVASDALPFLGWLDLDGQKKKMKRIAK
EIDLIAERWLQEHQEKKSLSNKKLLAAGGGKVDGHDDFMDVLLFILDDDSQFFNFSRDTVIKATSWAMIL
TAADTTSVSMTWALTLLLTNPRVLRKAQDELDMKVGRDRHVEERDIENLIYLQAIVKETLRLYPAAPLGV
PHEAIQDCTVAGYQVRAGTRVLVNLWKLHRDPRVWSNPSEFRPERFLIDDIDEQGGGGGGGGGESTAKDF
RGQHFEYIPFGSGRRMCPGINFALQIVHMTLARLLHAFELRTTTSASLMDMTEESGLTMPKKTPLVVLLQ
PRLPLPLYDHHE
>SDICYP82-1
MEFLSLQHQLISIFALLLASIFLYNLLKNHGRKSKTSKPPAPEASGGWPIMGHLNLFNGSELTHQALGSM
ADKYGPAFNIRFGSHQTLVVSSSEIVKECFTTNDRFFSNRPGSLAIKLMFYNADSVGYAPYGAYWRDLRK
ISTLKLLSNHRLETLKHLRTSEVESCFKQLYNQWKNNKVGGDHDFALVRMDNWFGDLTFNVVARIVAGKK
NFAGGATNGDVGAQRYKEAMDEAFRLMTIFAFSDVVPSLGWLDKLRGLVGGMKRCGAEIDSIVAGWVDEH
RLKRASGKGGGHTDLEQDFIDVCLEIMEHSTLPGDDPEIVIKSTCLDMILGGSDTTTVTLTWALSLLLNN
PHVLKRAREELDTHVGKDRQVDDSDMSNLVYIQAIIKETMRLYPAGPLIERRTSEDCEVGGFHVPAGTRL
LVNLWKMQRDGSVYKEDPLEFRPERFLTSNADVDLKGQNYELIPFGAGRRICPGVSFAVQLMHLVLARLV
HGFEMKTAGDAKVDMTESAGLISHKVTPLEVLLKPCLAIQQAL
>SDICYP82-2
MDSFLLIQWFAASMAALLAFVFLYNLVWSSSRTTKGKNIRKAPMAAGAWPILGHLHLFGSGELPHKMLST
MAEKYGPAFTMKFGKHTTLVVSDTRIVKECFTTHDTLFANRPSTTAFDLMTYANDSVAFTPYGPYWRELR
KISTLKLLSNHRLQSIKEIRVSEVNVCFRELYESCKSKTDATPVLVDMKKWFEEVSNNIVMRVIVGRQNF
GSKIVQGEDEAVNYKKVMDELLRLASLSMLSDVAPVLGWLDMFQGNKSAMKRNAKKVDTILEGWLEEHRM
KKSASGISSAGENDQDFMDVMLSIIEETKLSGRDADTVIKATCLAMIMGGTDTTAVSLTWIVSLLMNHRH
VLKKAREELDALVGKDRQVEDSDLKNLVYMNAIVKETMRMYPLGAMLERETKEDCEVGGFQVQGGTRLLV
NVWKLQRDPNVWTDPTEFKPERFLNENADIDVGGQHFELLPFGAGRRACPGVSFALQFMHLVLARLIHGY
ELGTQNDADVDLTESTEGHVNHKASPLDLLLTPRLNNPNLYDY
>SDICYP82-3
MTIGALALLSFIYFLRVSVIKRTKYTNTAVTATNKLENDEDEANHSKRVVAPPEVAGAWPILGHLPQLVG
LKQPLFRVLGDMADKYGPIFIVRFGMYPTLVVSSWEMAKECFTTNDRVLASRPASASGKYLTYNYAMFGF
TNGPYWREIRKISMLELLSHRRVELLKHVPSTEIDSSIKQLYHLWVENQNQNKQGDHQVKVDMSQLLRDL
TLNIVLKLVVGKRLFNNNDMDHEQDEAARKLQKTMVELIKVAGASVASDALPFLGWLDVDGLKRTMKRIA
KEIDVIAERWLQEHRQKKLTSNDKGGSNNIQGGGGDNDFMDVMLSILDDDSNFFINYNRDTVIKATSLTM
ILAGSDTTTLSLTWALTLLATNPGALRKAQDELDTKVGRDRQVDERDIKNLVYLQAIVKETLRMYPAAPL
AIPHEATQDCIVGGYHVTAGTRVWVNLWKLQRDPHAWPNPSEFRPERFLAVENDCKQQGTCDGEAANMDF
RGQHFEYMPFGSGRRMCPGINFAIQIIHMTLARLLHSFELRVPEEEVIDMAEDSGLTISKVTPLELLLTP

RLPLPLYI
>SDICYP82-4
MAALLPLAFLYNIFLASSSKATSKRTISTKKPPMVAGAWPILGHLHLFKEGELPHQMLKSMADKYGPAFL
MKFGQHQSLVVSDYRIVKECFTTNDTLFCNRPSTTAFDVMTYANESVAFTEYSPYWRELRKISTLKLLSN
NRLQAIKNLREEEVDVSFKGLYDSWKNKNNKSTAGSVGDERAPVLVDMKKWFEEVSNNVVIRVIVGKCNF
GTKIVQGEKEGVEYKTIMDELLRLASLSLLSDFAPILGLLDFFQGHVRTMKRNGKKLDVLLQRWLEEHKR
KKSTPEDEQDFMDVMLSIIEESKLSGYDADTVIKATCLAMIMGGTDTSAVSLTWIVSLLMNNRQALKKAR
EELDAQVGKDRQVEDSDLKNLVYLNAIVKETMRLYPLGTLLERETKEDCEVGGFHLEGGTRLLVNVWMVQ
RDPDVWTDPTKFIPERFLTEKADIDVGGGNFELIPFGAGRRVCPGVSFALQFLNLVLARLIHGYELGTPE
DADVDLTESPEGHVNHKASPLELLLTPRLSNPKLYDY
>SDICYP82-5
MDSDHQLLSYQFSASNMFISLFAFVLYYLLVWRPTKSNLKMKTISSEDKQAPELAGSWPVIGHLHLLHGP
NPLHVALGAMADKYGPAFTVRVGVHRTLVVSSWEVAKECFTTNDKVFVSRPRQAAGKHMGYNNAMLRFAS
DTPYWTQIRKMLKRDLLSNNRIELLNHAWHSEINTSIKELYEMNWSSGEGRRGGIRPPVSVDMKQWLGDL
TLNMSVKMIAGKRCFGSGVSACDEGEARRCQKGLKDFVRLMGQIVVSDAIPFLGWLDLDGYEGEMKRAGK
ELDRLLGGWLEEHKMKRSPGDEASAQKDWMDLMLSVLGDGKLDGYYDADTINKATCLALLQGGSIWTMLT
LLWAFALLVNHPHVMKKAHDELDNHVGRERQVEESDIKNLTYLQAIVKEAMRLYSVSMRLLESTADCTVS
GYDVPAGTRLIVNTWKIQRDPRVWSDPSEFHPERFLTHRHRDMDLYGQNFEITPFGSGRRSCPGSSLGLQ
MVQLTIARLLHGFEFKTPSDAPIDMTESVGVENMVKATPIEVLLTPRLLDEVYAFYN
>SDICYP82-6
FCQFQGIVGILLAFLTFLYYLWRASITGLRTKPKHNDFKVTKAAPEADGAWPIVGHFAQFIGPRPLFRIL
GDMADKYGSIFMVRFGMYPTLVVSSWEMAKECFTTNDRFLASRPASAAGKYLTYDFAMLSFSFYGPYWRE
IRKISMLELLSHRRVELLKHVPSTEIDSSIKQLYHLWVENQNQNKQGDHQVKVDMSQLLRDLTLNIVLKL
VVGKRLFNNNDMDHEQDEAARKLQKTMVELIKVAGASVASDALPFLGWLDVDGLKRTMKRIAKEIDVIAE
RWLQEHRQKKLTSNDKGGSNNIQGGGGDNDFMDVMLSILDDDSNFFINYNRDTVIKATSLTMILAGSDTT
TLSLTWALTLLATYPLCALRKAQDELDTKVGRDRQVDERDIKNLVYLQAIVKETLRMYPAAPLAIPHEAT
QDCIVGGYHVTAGTRVWVNLWKLQRDPHAWPNPSEFRPERFLAVENDCKQQGTCDGEAANMDFRGQHFEY
MPFGSGRRMCPGINFAIQIIHMTLARLLHSFELRVPEEEVIDMAEDSGLTISKVTPLELLLTPRLPLPLY
I
>TCOCYP82-1
MDLPQPTISTTLLAFFSILLFIICYRLLSTKSDKRSSSSRNKPLDAPGAWPVIGHLHLLNGVIQHEILGT
MADKYGPAFTIWLGTRPALVVSNWEVAKECFTTCDKALASRPPSLALKIMGYNDALFAFAPYGQYWRELR
KIVMIELLSSRQLDSLKHVWDSEIDSCIEDLYEKCKGHRETVLVNMKQWFADLMMNVVVRMVVGKLNFGK
TKEGDDEVAKAHQGQLKAMREFFKWVDVFMIEDAFPILTWLDPQGYQRKMKNIAKDLDSLVQGWLDEHKL
KQKTGDNGKKDFMDIMLSAVKDASISDRDDETICKATCLNIILGASDTTTVTLTWTLSLLLNNRHVLRKA
QEELQAQVGKHRCVEESDVKNLVYLHAIIKEVLRLYPAVPLLGPRESVADTVVAGYHIPAGTRLIVNLWK
IHRDPLNWPDPLEFRPERFLTTHKDTDVWGQNFELTPFGSGRRICPGISFALQVIPLTLARLLHGFELTT
PGSAPVDMTESTGLTNLKATPLDVFISPRLVL
>TCOCYP82-2
MESIPTAINVLLAFVSIYYLLKFRKKIITTTRDIKKIKAPELKGAWPIIGHLHLFRSDDLLHRKLGAMAD
ELGPAFMIRLGMRPALVVSNWQTAKECFTTKDRIFATRPDSVAAKHMGYENLILGFTRYGPYWREVRKIV
TLELLSSHRLHLLRHVRISEIDMSIKKLHQLCTSNDNKYSSVVVDLNQWFKALTLNIIVRIIAGKRYYGG
DLEDDEESRRWKKALNEFMHLFGIFSVSDAIPQLQGMDVQGHERVMKRTGKDIDVILSKWLEEHRRKKIR
VNREGAEKDFIDIMLDLIKENVNISGHDADNVVKATCLALVSAGSDTTMITLTWAVSLLLNNHRVLEKAQ
AELDNQIGINRVHVEEEDIKNLPYLQAIVKETLRLYPAAPFSLPHEAMEECTVAGFHVPKGTRLITNIWK
LQRDPQVWEDPLEFRPERFLTSDRRHVDFRGQHFELIPFGSGRRMCPGISFAIGVLHLTLARLLHEFHLG
TPTNAPIDMNENPGITLEKADPLHPLVSPRLTSINY
>TCOCYP82-3
MDSQLTSFQSKPSAYLIAALLALVSAYYLIIKKKSRATYQDNYGKIRAPEPKGAWPIIGHLLLFKPNDVL
YQKLSSLADELGPAFMIRLGMRSALVISNWEIAKECFTTNDRIFATRPKLMASKHMGYGGAEVGVAPYGP

YWREVRKIITVELVSNHRLNLLKDVRISEIDMSLKELYDLCMKKKKNGSGEESSRVELDLYQWFKDMSLN
ILVKLIAGKRYYGVAAAANEDEESRRWKKATEESVFLFGQFVVGDAIPLLEGVDVQGLERAMKKTGKEID
AVLSKWVEEHRMKKQQLNGARDEQDFIDVMLNIIKDGKISDHDADTIVKATCMSLVQAGSDTTMLTLTWA
VCLLLNNCNVLEKAQAELDDQIGRSKGRIVEEDDIKKLPYLQAIVKETWRLYPPAPLAVPREAMEDCTVA
GFHVPKGTRLMTNIWKLHRDPKVWDDSLKFRPERFLSSEHAHVDFRGQQHEFMPFGSGRRMCLGMPLAIG
VVHLTLARLLHEFELATPSNAPVDMSEKAGLTLVKATPLNVLIAPRLC
>TCOCYP82-4
MDSIILQSNLLTTVIIITSFGLFVLSFYYVLWKAIRTGNKRNCSNPKAPEAAGGWPILGHLHLFRGQGLL
LHKIFGAMAEKHGPAFTIRLGMRPALVVSNWEVAKECFTANDRALASRPTSLALKIMGYNNSLFAFAPYG
QYWRELRKIVMHQLLSSRRLELLKNVWLSEIDIWIKGLYENSLVNKVVDMKQWFGELMMNIVVRLVAGKR
SFGKIREGDSEEAKAHQRQIKALRDFFRLVEVFMIEDAFPFLTWLDPRGYQKEMKNIAKELDYLLEEWLE
EHKLKQQAACDDQSKDFMDVMLSEVKDSTITERDANTICKATCLNVILGASDTTTLTLTWALSLLLNNRH
VLKKAQEELNAQVGKDKQVEESDMKNLVYLHAIIKEVLRLYPAAPLSVAHESLEDTVVAGYHVSKGTQVI
FNLWKIQRDPSVWSNPLEFQPERFLTTHKHVDIWGQNFELIPFGSGRRMCPGVPFALQVVSLTLARLLHG
FELTTPGGAAVDMTETPGLTNMKATPVEVFLRPDLPHQLYA
>TFLCYP82-1
MPSQTIQIIMDFFHQCVATILFCVLASPFFFYYLLPWWKNANDHDLAKSRGKPVPEVDGAWPIIGHMHML
QPSDSFYSSLAEKYGPIFTLRIGLCKTIVINSWELAKECCTTHDRVFASSPEATAAKILGYDYAMFGRNP
YGPYWREMRKIIITELVSNRRLELLKHIRVTEISTSIQELYQLWEARSSKNVKERVVVDMQKWFGDLMLN
IGVEMVAGKRYFGASSNEDKEEARQLHKTFKDFSNLFGVPVLSDAIPFLRWLDYKGHIKAMKRCAKQVDC
ILHRWLEEHKQNKTTIAGDFMDILLSVLQDKEIFGRDADTVIKATCLNMMLGVVETNKVTVTMALISLLN
NRRILKKAQEEIDIHVGSDKQVEESDIEKLVYLQAIVKETLRLYSPGLGTREPSEDCTISGFHVPKGTEV
MVNVQQIHRDPSIWSNPLEFQPERFLTTQANIDFGGQHHEYIPFGAGRRLCPAISFAVLVVHLTLARLLQ
GFDFATPKDAPVEMNESVETLEVLITPRLPLHMY
>XSICYP82-1
MHNPIPNQWLPSSTLLTPIIVSFLVIVLFYIYKKRSSNTIRTKKAPEVVGAWPVIGHLNLFSCPKPGHIV
LGELADQYGPAFTIHFGMHPTLVVSSSELVRDCFTTNDKLFSSRLVNKAIKYMFYDQDTISFAPYGPYWR
ELRKMIALNLLNNERIKMLQQQRISEMDACLKKLYDLSAKRKDENAGVLVDMSKWFAEISFNVVTRIVAG
KHIFGPKVERYKNVMEEARRLMDVMVFSDMVPYLGWLDRLRGVDSAIKRTAKELDSVLESWVEEHRRKSV
SISAGTGGIFNIKEEEELDFIDIMLSIIAKNNLPGDDPDTLIKAIVQETYLAAWDNTTVTLTWVLCLLLN
NKQVLKRAQNELDAQVGKERQVEDSDINNLPYVQAIVKESLRLYPPGPIIERATTEDCNVGGFRVPAGTR
LWVNLWKLQRDPKVWPNDPLEFRPERFLNDNADIDLRGQNSELIPFGSGRRMCPGVSFSLQVIHLVIARI
IQGFELKAPTDADIDMSTTLGMISWKATPLEVLMNPRFPPVFYK
>XSICYP82-2
MSTLQISILTVKSPYSQMHYYPLLHQWLPASMALVSLLVIILFSIFKKRSSKMIKAKKAPEVAGAWPLIG
HLNLFSGPKLLHLVLGELTDQYGPAFMIHLGMYPTLVVSNWELLKDCFTTNDIFFSNRPVNKAIKHMFYN
KESIGFTPYGSYWRELRKMTTLKLLSNHRLDLLKPLRISEMDACFRNLYELWTKDKDGNAWLLVDMSKWF
GEISFNVVARIVAGKKNFGSKGDRYKTVMEEVVRLMGLRALSDAVPYLGWLDQLRGLDSAMKRAAKELDS
VLESWVEEHRLKRVSVSAGTGSTVKTAKEEEEEEDFIDITLSILAENQLPGDDPDTGIKSLILDMILGGS
DTSTVTLIWAMCLLLNNVHVLKRAQYELDAQIGKERQVEDSDIKNLPYIQAIIKETMRLYPAGPIIERQA
NEDCDVGGFHVPAGTRLWVNLWKLQRDPNVWKDDALEFRPERFLTDHADIDLKGQHLELIPFGSGRRICP
GISFALQVMHLALARIIHGFELKTPNDSNIDMSGTPGLLCCKATPLQVLLTPRFNPMFYK
>XSICYP82-3
MDSLRHCLGGILALLIFLGYLQWKRGTSNKCIEAPQPDGAWPIIGHLPRLMKPQIMHRTLSTMADKHGPA
FTLRLGVHKTLVVSSSWEVAKECFTTNDRVFATRPTSIAVEILGYNYALFAFGPYGSYWREARKIAILELL
SNHRLELLKHVRISEVSTSIRELYQVWKENCDIANGSALVDMKPWFGDLTLNVVVRMVAGKRYMGGSVKS
DDVEGRRFQKATKDLFDLFGLFILSDALPFMGWLDLHGHKKAMKKTAKELDSIMQRWLEEHRQSLLDDGT
KEEKKDFMYVMLSILEDKKLFQYDADIVNKAICMNMIMAGTDTQMITLTWVLSLLLNNRHILKKAQDEID
SSVGKDRQVEESDIVKLVYLQAIVKEALRLYPPAPLSAQHEAMEDCTVAGYYVPAGTRLITNIRKIQRDP
RVWSDPFEFHPERFLTNQANVDFRGQSFEFIPFGSGRRMCPGISFALQVVHLTLARILQGFDFETPMNAS

VDMTEAPGLTNVKSTPLQVLITPRLHPNLY
>XSICYP82-4
MDLLNPYFATIFGGFFALLIFLFIISQKRSRIPKIRAAPEPVGAWPIVGHLPMLLGPRLPHFVLGDLGEK
YGSAFTLRLGIHKTLVVSSWEVAKECFTTNDQVFATRPSFMAAKIMGYNYALFGLAPYGSYWRELRKISI
IELLSSHRLELLKHVRVSEVSTSMKELYEVWAENCSNGNGSVLVEMQRWFGDLTLNISVRMVAGKRYFGT
SASLDDDEARRISKATKDFFRLTGMFVVSDSVPFLWWLDFQGHEKAMKRTAKEMDDIFGGWLEDHRRNKL
AGGTKVQQDFMDVMLSILEDEKFFGYNADVVTKATCLNMLLGGTDTTMVTLTWALSLLLNNRHILKKAQT
EINTHVGKDTQVDESDIVKLVYLQAIVKETLRLYPAAPLSTPHEATKDCTIAGYHVTAGTRLITNIWKIQ
RDPRVWSNPSEFQPERFLTDQANVDVRGQHFEFIPFGSGRRSCPGISLGLLVVQLALARLLQGFDFETPS
DALVDMTESAGLTNLKATPLHVLITPRLHSSLY
>XSICYP82-5
MDSVHQGQYLPTPMVSVFVFFISLYFLIVWKTRSSSKTNTCNEVPEAPGAWPIIGHLHLLGGSELPHKTL
GAMADKYGPIFKIRIGVHQALVVSSSDIVKECFTTNDKVFASRPTSTASKILGYDYVMFGFSPYGPYWIE
LRKIIMSELLSNRRLELLKHVRDSEIDISIQELYKVWKNHDKSKGPILVDMKQWFGDLALNVILRMIAGK
RYSGSIFSSDETEARRCQKGARDFFRLLGLFIIEDALPYLSWFDLQGYKKEMKNTAKELDSVFQRWLEEH
KRTRETGELNREQDFMDILMSTLEETKISEYDNDTIIKSTCLSIVTGGNDTTMVTLTWILSLLLNNKHAL
KKVQDELDSHVGKHRHVEESDIKNLIYLQAIMKEALRLYPAGPLSGPRVADADCTLAGYHIPAGTRLLVN
TYKIQRDPLVWSEPSEFRPERFLTSHVNMDVKGLQYELIPFGTGRRACPGMLFALQVVPLVLARFLHEFE
PKTEMDMPVDMTETAGLSNAKATPLEVVITPRLQPELYSL
>XSICYP82-6
MDFTMIIQWLLTSFAILVAFVLIWSSATRSSTRKRNKTAPVAAGALPIVGHLHMLMGRELPHRLLSNMAD
KYGPAFMMKYGLQPALVVSSLKLTKECFVHNDRAVFKRPRSKALKNLTYDQASLGFAPYGPLWVEMRKVS
KTNLLSNQRLQMQRHVRASEVDAFIKELYDLWSSKSNNIKGAPLLVEMNKWFEELALNVVTRMLSGKRHI
GFKARRGEDSESMHYKKVVVDATLLTAKLVVSDFFPSLGLVDYLQGDESSIKGTSKELDAILATWVEEHR
HKKVSGSEDDEKDFIDLTLSMIDQTQHQGADVDTFIKSMCVGMIFGGSDSTSVALAWALSILMNNRPVLK
KAREEIDRQVGRDRKVNDLDVLKLDYLKAIVKESMRLCLVGPLLERVAVEDCEIGGYHVKAGSRVVVNIW
KMQHDPNLWSDPLEFQPERFLTTNANVELRGQHFELLPFGAGSRICPGITFALELIQLTLARLIHGFELG
TPMDADVDMTETSSVTNYRATPLEILLTPRLDPKLYNY
>ECACYP82B1
MEKPILLQLQAGILGLLALICFLYYVIKVSLSTRNCNQLVKHPPEAAGSWPIVGHLPQLVGSGKPLFRVL
GDMADKFGPIFMVRFGVYPTLVVSTWEMAKECFTSNDKFLASRPPSAASSYMTYDHAMFGFSFYGPYWRE
IRKISTLHLLSHRRLELLKHVPHTEIHNFIKGLFGIWKDHQKQQQPTGREDRDSVMLEMSQLFGYLTLNV
VLSLVVGKRVCNYHADGHLDDGEEAGQGQKLHQTITDFFKLSGVSVASDALPLLGLFDLGGKKESMKRVA
KEMDFFAERWLQDKKLSLSLSSETNNKQNDAGEGDGDDFMDVLMSILPDDDDSLFTKYSRDTVIKATSLS
MVVAASDTTSVSLTWALSLLLNNIQVLRKAQDELDTKVGRDRHVEEKDIDNLVYLQAIVKETLRMYPAGP
LSVPHEAIEDCNVGGYHIKTGTRLLVNIWKLQRDPRVWSNPSEFRPERFLDNQSNGTLLDFRGQHFEYIP
FGSGRRMCPGVNFATLILHMTLARLLQAFDLSTPSSSPVDMTEGSGLTMPKVTPLKVLLTPRLPLPLYDY
>ECACYP82N2V2
MDSLMLAYLFPISVASIIAFVFLYNLFSSRTLKNKKIRTAPMATGAWPVLGHLHLFGSGELPHKMLAAMA
DKYGSAFRMKFGKHTTLVVSDTRIVKECFTTNDTLFSNRPSTKAFQLMTYDNESVAFTPYGSYWREIRKI
STLKLLSNHRLQAIKDVRASEVNVCFKTLYDQCKNPSGSAPILIDMKKWFEEVSNNVVMRVIVGRQNFGS
KIVQGEEEAIHYKKVMDELLRLASLSMFSDFAPLLGFVDIFQGNLSAMKRNAKKVDAILENWLEEHRKKK
NSVAESQQDFMDVMLSIVEESKLSGHDADAVIKATCLAMIMGGTDTTAVSLTWIISLLMNNRHALKKARE
ELDALVGKDRQVEDSDLKNLVYMNAIVKETMRMYPLGTLLERETKEDCEIDGFHVKGGTRLLVNVWKLQR
DPNVWVDPTEFRPERFLTENADIDVGGQHFELLPFGAGRRVCPGVXFALQFMHLVLARLIHGYDLNTLNE
ENVDLTESPEGHVNHKASPLDLILTPRLHYKLYE
>NTACYP82E4v1
MVFPIEAIVGLVTFTFLFFFLWTKKSQKPSKPLPPKIPGGWPVIGHLFHFNDDGDDRPLARKLGDLADKY
GPVFTFRLGLPLVLVVSSYEAVKDCFSTNDAIFSNRPAFLYGDYLGYNNAMLFLANYGPYWRKNRKLVIQ
EVLSASRLEKFKHVRFARIQASIKNLYTRIDGNSSTINLTDWLEELNFGLIVKMIAGKNYESGKGDEQVE

RFKKAFKDFMILSMEFVLWDAFPIPLFKWVDFQGHVKAMKRTFKDIDSVFQNWLEEHINKREKMEVNAEG
NEQDFIDVVLSKMSNEYLGEGYSRDTVIKATVFSLVLDAADTVALHINWGMALLINNQKALTKAQEEIDT
KVGKDRWVEESDIKDLVYLQAIVKEVLRLYPPGPLLVPHENVEDCVVSGYHIPKGTRLFANVMKLQRDPK
LWSDPDTFDPERFIATDIDFRGQYYKYIPFGSGRRSCPGMTYALQVEHLTMAHLIQGFNYRTPNDEPLDM
KEGAGITIRKVNPVELIIAPRLAPELY
>PSOCYP82X1
MELFIKLPFIQPIPFSIILVTTVSIVLLYSVFFWVTDKKKKRKKAPNAAGAWPLIGHLRLLMNDKEPLYR
ALGSMADKYGPAFNIRLGNQEVLVVSNWEMVKQCFGNQNDKLFSNRQTTLAAKYMLNQTTSSGFAPYGPY
WRELRKIMVQQLLSKQSLESWKHLKIKEMDASFSKLNELCNNNGTGTATLIRMDEWFAELTFNVIARNVF
GYQSGGRSTALTNGDTESKGERYKKTLEEALHLMSIFAVSDIFPSLEWVDRLRGLIRNMKRFGDELNSIA
GCLIEEHRQKRLQSVSKSDKGVGDEQDFVDVLLSVAEKSQLPGDDPDLVIKSMILEIVSGGSETTSSTLT
WALCLLLNHPHVLKKAKEELDTHVGKDRHVEESDTPKLVYINAIIKESMRLYPNGAMLDRLALEECEVGG
FHVPAGGRLFVNVWKIQRDPSVWENPLEFKPERWFLSNGEKMDVDYKGHNHEFIPFGIGRRMCAGMLWAS
EVIHLVLPRLIHGFDMKAASANGKVDMAEMAGMVICFKKTPLEVMVNPRE
>PSOCYP82X2
MKSLMMNKLLFLQRITDSPSTTIISTFIVTIISIVFLYTVLLIRTTKNKQKIAAPKASGAWPFIGHLKLF
MKQDTQFYRTLGTMSDKYGSVFTLRLGNQAILVVSNWEMVKECFTTNDKSFSNRPSTLSTKYMLNDTNSV
VFSPYGTYWREMRKILVQKLLISNQRSEALKNLKTKEIDNSFVKLNDLCNNDVSGGGTKVRMDEWLADMM
FNIIARITFGYQSGGGDAPGASTTSKNVERYKKTLDEMFVVLATRFAVSDIFPSLEFIDRLRGLVKDMKI
LGDELNSIAGCFIEEHRQKRRESLSSLLSLSNESVGDEQDFIDVLLSIMDQSRLPGDDPDFIIKIMILEA
FAGGTDSLSATLTWVLSLLLNHPNVLKRAREEIDRHVENGKQVEVSDIPKLGYIDAIIKETMRLYPVGAL
SERYTTEECEVGRFNVPAGTRLLVNIWKIHRDPSVWENPSDFQPERFLCSDKVGVDLYGQNYELIPFGAG
RRVCPAIVSSLQTMHYALARLIQGYEMKSASLDGKVNMEEMIAMSCHKMSPLEVIISPREPRRS
>PSOCYP82Y1
MAYLMIKKSIYLFFDQPTAVGTLILAFLLTLSPVIIYYEQKKRGLRRNRTAITTTPLPEASGAWPVIGHL
LLFMNENDLNHVTLGHMADKYGPIFSLRFGRHRTLVVSSWEMVKECFTGTNDKLFSNRPSSLAVKLMFYD
TESYGFAPYGKYWRELRKISTHKLLSNQQLEKFKHLRISEVDNSFKKLHELCSNNKQGGDTTYVASLVRM
DDWFAYLTFNVIGRIVSGFQSNAVAGATNSQEKYKLAIDEVSNLMATFAVSDVVPRLGWIDRLTGLTGKM
KNCGKKLDAVVGDAVEDHRQKKLKISRNNTGALTEHEEEDFIDVCLSIMEQSQIPGNHPEISVKSIALDM
LSGGSDTTKLIMTWTLSLLLNHPDILDKAKEEVDTYFGKKKISDNTPVVDAADVPNLVYIQAIIKESMRL
YPASTLMERMTSDDCDVGGFHVPAGTRLWVNVWKMQRDPRVWKDPLVFLPERFLSNDKGMVDVKGQNYEL
IPFGTGRRICPGASFALEVLHLVLTRLILEFEMKAPEGKIDMRARPGFFHNKVVPLDVQLTPRTLD
>PSOCYP82N4
MRTESIKTNRPMDLLLQYLQPISVALVVIALVWNYGRRNPTKKLAPEASGGRPIMGHLHLFNDGELTHRK
LGAMADTYGPVFNIRFGSHKTLVVSDWEIVKECFTTNDKLFSNRPGTLGIKLMFYDADSVGYAPYGAYWR
DLRKISTLKLLSNHRIDTIKHLRSSEVESCFESLYSQWGNGEKSGEFAPVRMDSWLGDLTFNVVARIVAG
KKNFSANGDVGAQRYKAAMDEAMRLMRFFAFSDVIPSLSWLDNLRGLVREMKKCASEIDSIMATWVEEHR
VKRNSGGNSQLEHDFIDVCLDIMEHSSLPGDDPDLVVKSTCLDMILGGSDTTTVTLTWAMSLLLNHPQVL
QKAKEELETQVGKNRQVDDSDIPNLPFIQAIIKETMRLYPAGPLIERRTMEDCEVAGYQVPAGTRLLVNV
WKMQRDGNVYKGDPLEFRPDRFLTSNADVDLKGQHYELIPFGAGRRICPGVSFAVQLMHLVLARLLHEFE
ITTVEPETKVDMAESGGLLCYKIMPLEVLIKPRLEI
>PSOCYP82N3
MDFSSLLLLLLNTWISAYSMAALLALVLVYNLRMTKSSSSKTTSLKGKKIITRPPAVTGAWPVFGHLHLF
GSGEHPHEMLSKLAEKYGPSFTMKFGKHTTLVVSDTRVVKECFTTNDTLFSNRPSTIAFDLMTYATDSIA
FTPYSPYWRELRKISTLKLLSNNRLESIKQLRTSEVSVCFKELYDLTNKKNDNGAPVPIDLKRWFDEVSN
NVIMRVIFGKQNFGSKIVLGEDQEAVHYKKIMDELSRLSSLTMLSDMVPLLGWLDYFKGDLRAMKRNGKE
LNSILQKWLEEHKSKKSSDARQDFMDVMLSISKDTQLYGHDQDTFIKATCLAMIMGGTNSTEVALTWILS
LLMNNRCALHKAREEIDLLVGKDRQVEDSDVKNLTYMNAIIKETMRLYPLGFLLERDTKEDCEVSGFNIK
GGTRLLINVWKLQRDPNVWTDPMEFKPERFLTENADIDVGGQHFELLPFGAGRRVCPGVSFALQFMHLVL
ARLIHGYDMETLNGEDVDLSVSSGGHVNIKSTPLELILTPRLHPELYDCET

CYP719

>HSACYP17A1
MWELVALLLLTLAYLFWPKRRCPGAKYPKSLLSLPLVGSLPFLPRHGHMHNNFFKLQKKYGPIYSVRMGT
KTTVIVGHHQLAKEVLIKKGKDFSGRPQMATLDIASNNRKGIAFADSGAHWQLHRRLAMATFALFKDGDQ
KLEKIICQEISTLCDMLATHNGQSIDISFPVFVAVTNVISLICFNTSYKNGDPELNVIQNYNEGIIDNLS
KDSLVDLVPWLKIFPNKTLEKLKSHVKIRNDLLNKILENYKEKFRSDSITNMLDTLMQAKMNSDNGNAGP
DQDSELLSDNHILTTIGDIFGAGVETTTSVVKWTLAFLLHNPQVKKKLYEEIDQNVGFSRTPTISDRNRL
LLLEATIREVLRLRPVAPMLIPHKANVDSSIGEFAVDKGTEVIINLWALHHNEKEWHQPDQFMPERFLNP
AGTQLISPSVSYLPFGAGPRSCIGEILARQELFLIMAWLLQRFDLEVPDDGQLPSLEGIPKVVFLIDSFK
VKIKVRQAWREAQAEGST
>AMECYP719–1
MEEKIMTNNSPWILTSSTTTTTTILLSLLFTIFIILRRNKSSSSKMVWPTGPKTLPIIGNMNILGGTALH
VVLHNLAKTYGNVMTIWIGSWRPVIVVSDIDRAWEVLVNKSSDYSARDMPEITKLATADWKTISSSDSGP
FWTNLRKGLQNVALSPQNLSSQSKFQERDIIKTIQNLKEEAKMNNGIVKPLDHLKKAMVRLISRLIYGQD
FDNDEYVEEMHHTIEELIRVSGYARLAEAFYYAKYLPSHKKAVREVLQANQRVQNLVRPLLSLNSPTNTY
LHFLRSQNYEDEVIIFAIFEAYLLGVDSTSSTTAWALAYLIREPNVQEKLYEELKNFTNDNDRKMVKFED
LNKLQYLQAVVKETMRMKPIAPLAIPHKACRETSLMGRKVNQGTRVMVNIYALHHNQNVWKEPYKFNPER
FLQKNQDGVDGKAMEQSLLPFSAGMRICAGMELGKLQFSFALANLVNAFKWSCVSDGVFPDMSDQLGFVL
LMKTPLEAGIVPRM
>AMECYP719–2
MPDITKIISANWKNISCSDSGPFWHNLRKGLQGVALTPLNVASQYHLQERDMKNLINSMYKDASQKNGIL
KPLDYLKEETVRLLSRLIFGQDFQDENLVVGMHHALDDLVRISGYASLADAFKFCENLPSHKKSIREVHE
VKKRVENLIRPHIVSNPPTNTYLHFLKTQDFNEDIIISAILEVYDLGVDSTASTTVWALTFLVREQEIQE
KLYREIVNVTGGKRSVKVEDVNKMPYLQAVMKETMRMKPIAPMAIPHKTSKETSLMGKKINKGSVIMVNL
YAIHHNPKVFPEPYKFMPERFLKDVNSDESLGNIKTMESSLLAFSAGMRICAGMELGKLQLAFGLASLVH
EFKWSCSVDGKLPDLSEDHCFILLMKNPLEAKITRRIH
>BTHCYP719–1
MEFTVWVVGFNVLVVVAMTMFLLRTKSSSSTTKWPIGPKKLPIIGNLHQLGGDVLHVALAKLAKVHGGIM
TIWIGSWRPVIVVSDIDKAWEVLVNKSSDYSARDMPEITKIASASWHTISSSDSGPFWQNLRKGLQNGAL
GPANISAQSQLQERDMKRMIGDLQKEAKINGGIVKPLDHIKKATVRLLSRLIFGHMFDDDSFIESMHHEI
EDLIRISGYARLAEAFSYAKYLPSHKLAVKEAYEVKHRVEQLVRPLLTSSPPANSYLNFLLSQDYSEEVI
IFCIFEIYLLGVDSTSSTTTWALSFLIHEQQVQEKLYQSIKGVTSDDDGLVKAEDLSKLQYLQAVMKETM
RIKPIAPLAIPHKASKDTKLMGTKVAKGTRVMVNLYALHTKENIWSDPEKFKPERFLHGDEGANVKAMEQ
CFLPFSAGMRICAGMELGKLQFSIALANLVKAFKWTSVVDGELPDMSEELSFVLLMKTPLEARIIPRHL
>CCHCYP719–1
MVNAVAEILTEKPWLLPATLLAVIAIAKFFLGKSSTMKWPIGPKTLPIIGNMHQLGGTDLQVVLANLAKT
YGTIMTIWVGSWRPMIVLSDIEKAWEVLVNKSSDYSGRAMPEITEIISAKGKTISTSDSGPHWSNLRKGL
QNVALSPHNIAAQSRFQESDITKMIKTLKQEAASNNGIVQPLDHLKKSTVRLISRLIFGQDFDDDEYVEA
MHLAVEELIRVSGYARLAEAFYYAKYLPSHKRAVSEVWEANRRVKRIVKPRLSANPPPNCYLHFLSSQDY
SEEMIIFAIFEAYLLGVDSTSSTTAWALGFLIREPKVQERLYQELKNCAGENGLIKVEDINKLPYLQAVL
KETMRMKPIAPLAIPHKAVRETSLAGNKVEPGTRVMVNLYAIHHNPKVWIDPHKFKPERFLQGEENGGGN
LKLMEQSLLPFSAGMRTCAGMELGKLQFGFSLANLVNAFQWDCAKKGMFPDMSDLLGFVLLMKTPLQAKI
VPRGSPSINGY
>CCHCYP719–2
MVAAVVEILKEKAWLLPTTLVAIIAIAKLFLAKSSTVKWPTGPKTLPIIGNMHQLGGTELQVTLTRLAKT
YGNIMTIWVGSWRPMIVVSDIEKAWEVLVNKSSDYSGRAMPEITQIGSANWKTISTSDSGPHWSNLRKGL
QNVALSPHNIAAQSRFQESDISKMIKALKQAAVANNGIVLPLDHLKKTTVRLISRLIFGQDFDDDEYVEA
MHLAIEELIRISGYARLAEVFYYAKYLPSHKRAVDSAWEVNRTVKRIVKPLLLVKPPTNCYLHFLTSQDY

SEEVIIFAILEVYLLGVDSTSSTTAWALGFLIREPRVQEKLYQELKNFAGENGMIKVEDINKLPYLQAVL
KETMRMKPIAPLAIPHKAVRETSLAGNKVEPETRVMVNIHAIHHNPEVWMDPYKFKPERFLQGEDINGGG
NLKLMENSFLPFSAGMRICAGMELGKLHFGFSLANLVNAFQWDCAKKGKLPDMSDLLGFVLFMKTPLEAK
IVPRGSSSNINGY
>CCHCYP719-3
MEESFWIVSATIVVVFVIATMFRKSSSISSKTEWPAGPKKLPVIGNLHQLGGDVLHVVLANLAKVYGTVM
TVWVGSWKPMIVISDIDRAWEVLVNKSNDYSGRDLPEITKIISANWKNIMTADAGPYWTSLRKGLTGHTL
APTNVASQSHLQEKDMNNLINRMKNQAASNNGIIKPLDHLKEETVRLLSRLIFGQHFEDEHFVEGIHQAL
DDLVRISGYASLADAFKFCENLPSHKKSISGVHEILSRVRNLVRPYIVPNPPTNTYLHFLQSQKFTEEVI
IACILEVYDLGVDSTAATTVWALTFLVREPEVQEKLYREIQTVIGDRGTVKVEDISKMTYLQAVMKETMR
MKPIAPMAIPHKAVRETTLMGNKIDKNTVVMVNLYAIHHNPKVFPEPYKFRPERFLAGGDGKFGNLKAME
QSLLPFSAGMRICAGMELGKLQYGFALASLVNAFNWTCTADGKLPDMSEDHCFILLMKNPLVARIIPRVN
>CMACYP719-1
MEEINVEWISTAATTIGIIVVVFATVSMMFGRRRKSSMKWPNGPKTLPIIGNMHQLGGTALQVVLHKLAD
VYGSVMTIWIGSWRPVIVVSDIDKAWEVLVNKSSDYSARDMPEITRINTANWSTISTSDSGPHWSNLRKG
LQNVALSPLNVAAQSQFQEKDINQVIKNLQEESALNGGIVKPLDHLKKATVRLLSRFIYGQNFDDDTYVE
NMHNEIEELIRISGYARLAEAFYYAKYLPSHKKVEKAVEEAHYRVKALISPFLTRNPPPNSYLHFLRSQN
YPEEVIIFCIYEVYLLGVDSTSSTTAWALAYLVRESAVQEKLYQELNNFIGDRQQMGSAIKVEDVNKLQY
LQAVMKETMRMKPIAPLAIPHKACKDTSLGGNKVDKGTKVMVNLHAIHHNPKVWNEPNRFKPERFLQLKD
KKEMEQSYLPFSAGMRTCAGMELGKLQFGFALANLVYAFQWSCVADGKPPDMSDLLGFVLFMKTPLEARI
VPRVR
>CMACYP719-2
MEESFWLVAATVLVVFVVAKLLFRKSSSISTMEWPAGPKTLPIIGNLHQLGGAALHVVLANLAKVYGSVM
TIWVGAWRPMIVISDIDKAWEVLVNKSSDYSARSLPEITRIISANWKNIMTSDSGPFWQNLRKGLQGGAL
SPHNVMSQYQLQERDMQNLIKTMRVEASKNNGRIKPLDHLKQETVRLLSRLIFGQDFNDEKLVVGMHHAL
DDLVRISGYASLADAFKVAENLPSHKKSIREVHELKRRVENLVRPHIVSNPPTNTYLNFLLSQNFSEDLI
ISAILEVYDLGVDSTASTTVWALTFLVREQKVQEKLYQEIKNLTGGRSTVKVEEVSKMPYLQAVMKETMR
MKPIAPMAIPHTAARETSLMGKKIDKGTVVMVNLYAIHHNPNIFPEPYKFMPERFLHGEEQNGGNIKEME
QSLLPFSAGMRICAGMELGKLQLGFALASLVNAFKWECAADGKLPDLSEDHCFILLMKNPLEAKITPRTH
>CMACYP719-3
MEGSLWIILSILVGAVMILVQNLWKRWSKTSEDMILPPGPRKIPIIGNLYQLNRGGELIHVALAKLAQEH
GKMMTIWFGGGQPSIVVSHHEVAWEVLVTKAADYSSRTLPFMSRVTSADWHTLATSNLGPFWQTLRKGLR
STTLNPHTISAQVQLQEEDVTRMILSFKEQACLNDGVVKPLIPLRRTTVQLIGRFCFGPEFKNDDDFVEE
MDIVIEDVISLTGHGRLIDIFEFTRYIPGLNLPFKQAYKVKQRIIELIRPYIDYHKSSKSPCPNCYLHFL
LSHDYDEEVITFNLFELFLLAVDSTSNATAWALAYLIHDQKIQQKVYDEVSEFGRKEMVTIEEVSKLKYV
NAVVKEIIRMKPIAPLAVPHQADRDSKLMGIQVKKGTPVLVNLYAMHYDPSIWIEPSRFMPERFLVGHPQ
NVHCGEDHMAVMERSLIPFGAGRRICAGMDLAKLQVALTIANLVNSFQWSSAVEGQLPDLTEDLTFILRM
KTPLAARIIPR
>CMUCYP719-1
MDQTNILLAVAAALFIGLLTKLLASNSKHHLPPGPKPLPIIGNMLELSKGGELLHVSLAKLAERYGEIMS
FWFGGFEPSIVVTSHELVWEVMVTKANDFSDRALPWLTKILTSNWQTLATCDLGPYWYSIRKGLQSTTLN
HQTIMSQTQLQERDIADLLTSLEQEASSNNGIVKPYPMLRKLMVRLVSRFCFGPDFPNDEEFVERMDSTL
DESIHQSGHTRLADLFEFTRYVPGLWGPFKESESLKRRIVNLLTPHLKAASSPNCYSSLLLSKGLSEEVV
VLNLYELFELAVDATSNTTAWALAFIIHNKDAQEKVFKEVRYNQVGERRKIIRVEELGEMEYVQAVAKET
IRMRPIAPVMPHKAARDGELKGVKVRGGTPVLMNFYAVLHDEKVWREPYKFTPERFTTKGVDAEAMERSY
VSFGVGRRSCPGMELAKIQIAVTVANLVNRFEWCSAAEGELPDLSEGLTFVLMMKTPLVARIVPRSY
>CMUCYP719-2
MDVHYSTLLIALATLASLLLLSKLLLRSINTSKHHQWPPGPNKLPLIGNLHQFRGDLLHQNFAELAQKYG
KLMTVWIGSQQPFIVVTDPDLAWEVLVTRAVDYWSRQMLYLSRIISAGNRTLATSDGGPYWETLRRGLQS
TALSPQTISSQTKLQEQDIAHMIASLQQEASLNDGLVKPLPHIRKLAIRLLARLCFGADFPNDEHFVERM

DELLEDDMRLLTAAGLVDVFEFTRHIPGLTPRLKEIEDHMEGIKGLIRPCLAAAMKKKGNSISLGNTHMN
FLISQGFTEEIIILNLFEVFVFGVDSTSISLSWALGFLIDGRETQEKVLKEIVSKLGGSRRVVGVGDVSG
MEYVHAVVKETLRMRPVAPMAVPHKAARDSELMGFKVKEGTPFFVNLYALHHNEKAWKDANKFVAERFVK
GDGAEEYLKKMERYYIPFGAGRRACPGMELAKVHLAMVVANLVNSFEWQSAVEGQPLDLSETLNPLLVMK
TPLVARLVPRFA
>ECACYP719-1
MEEMKILMMNNPWILTATATTLLISIFLFFTRKSSKMVWPAGPKTLPIIGNMHLLGGTALQVVLHNLAKV
HGSVMTIWIGSWRPVIVVSDIERAWEVLVNKSSDYSARDMPDITKIISADWKTISTSDSGPHWTNLRKGL
QNVALSPHNLAAQFQFQEKDMTKMIQTLEEEARNNNGIVKPLDHMKKATLRLISRLVFGQDFNNDKYVDD
MHLAIEELIRVSGYARLAEAFYYAKYLPSHKKAVREVEEAQRRVQNLVSPFLSLNPPTNTYLHFLRSQKY
DDEVIIFAIFEAYLLGVDSTSLTTAWALAFLIREPNVQEKLYQELESFASKNDRKILKVEDINKLQYLQA
VIKETMRMKPIAPLAIPHKACRDTSLMGKKIDKGTRVMVNIFALHHNKNVFNDPFKFMPERFMKVDSQDA
NGKAMEQSLLPFSAGMRICAGMELGKLQFSFALANLAYAFKWSCVADGVLPDMSDQLGFVLLMKTPLEAR
INRRN
>ECACYP719-2
MEEIKILIMNNPWILTATATTLLISMFLFFTRKSSKMVWPQGPKTLPIIGNMHLLGGTALQVVLHNLAKV
HGSVMTIWIGSWRPVIVVSDIERAWEVLVNKSSDYSARELPDIIKYNTADCRTIATCDSGPHWSNLRKGL
QNVALSPNNLAAQFQFQEKDVIKMIDILEQEAINKNGIVKPLDHLKKATIRLISRLVFGQDFNDDKYVED
LHQAIEELIRMSGYAQLAEAFYYAKYLPSHKKAIRHAELTKQIVTNLVRPFLSLNPPTNSYLHFLQSQKY
DEEMVIFAIFETYLLGVDSTSSTTAWALAYLVREPNVQDRLYNELNNYAKYNDRKILKVEDINKLQYLQA
VTKETMRMKPIAPLAIPHKACRDTSLMGTKIDKGTKVMVNLHALHHNENVFTDPFKFMPERFLKVNNEDV
NMKAMEQSLLPFSAGMRICAGMELGKLQFSFALANLIFAFKWSCVDDGVLPDMSDELGFVLLMKTPLKAR
INPRN
>ECACYP719-3
MEESLWVVTATVVVVFAIAKLLKKSSSISTMEWPKGPKKLPIIGNLHQLGGEAFHVVLANLAKIHGTVMT
IWVGAWRPMIVISDIDKAWEVLVNKSSDYAGRDFPEITKIISANWKNISCSDSGPFWQNLRKGLQGGALA
PLNVISQYQLQERDMKNLITSMQEKASKNNGILKPLDYLKEETIRLLSRLIFGQSFNDENFVKGVHLALD
DLVRISGYASLADAFKFCENLPSHKKSIREVHEVNERVVNLVKPYLVKNPPTNTYLYFLNSQKFSDEVII
SAVLEVYDLGVDSTASTAVWALTFLVREPRVQEKLYKEIIDLTGGERSVKVEDVSKLPYLQAVMKETMRM
KPIAPMAIPHKTSRDTSLMGKKVNKGTSIMVNLYAIHHNPKVFPEPYKFIPERFLQGQESKYGDIKEMEQ
SLLPFSAGMRICAGMELGKLQYGFSLASLVEAFKWTCAVDGKLPDLSEDHCFILLMKNPLEARITPRTQL
>ECACYP719-4
MEEMKFLIMNNPWVLFATSATLLISIFLFFRRKSPNMAWPEGPKTLPIIGNMHLLGGTALQVVLYNLAKV
HGRVMTIWIGSWRPVIVVSDIEQAWEVLVNKSSDYSARDMPDITKIVTADWRTISTSDSGPHWSNLRKGL
QNIAISPNNLAAQFQFQEKDIIKMIQILQQEAKDNNGIVKPLDHLKKATIRLISRLVFGRDFEEDKYVED
MHHAIEELIRISGYARLAEAFYYAKYLPSHRKAVRYVEELKQIVKNLIRPFLSLNPPTNTYLHFLRSQNY
DEEVVIFAIFETYLLGVDSTSSTTAWSLAYLVREPNVQDRLHQELDHFAKQNDRKILKVEDMNKLQYLQA
VIKETMRMKPIAPLAIPHKACKDTSLMGNKINKGTRVMVNLYALHHNKNVFNDPFKFMPERFLKVDNQDA
KGKAMEQSLLPFSAGMRICAGMELGKLQFSFALANLIFAFKWSCVDDGVLPDMSDELGFVLLMKTPLKAR
INPRN
>GFLCYP719-1
MEMKSSVVFSYPAMEELFKSNPWVITGTTLAVLILMTTMFRKKSLSMKWPKGPKTLPIIGNMHLLGGTNL
QVILHNMSKVYGNVMTIWIGSWRPVIVVSDIERAWEVLVNKSSDYSGRDMPEITKIISADWKTISTSDSG
PHWTNLRKGLQNVALSPHNLAAQFQFQERDICKLIETLKEEAKLNNGIVKPLDHLKKATIRLISRLVFGQ
DFDNSKYVEDMHHAIEELIRVSGYARLADAFYYAKYLPSQKKAVRDVEEAQRRVQRLVRPFLSLNPPTNC
YLHFLRSQNYSDEVIIFAIFEAYLLGVDSTSSTTAWALAFLVREPNVQEKLYQELKNFTAKNGREMLKVE
DINKLPYLQAVIKETMRMKPIAPLAIPHKACRDTSLKGRKIDKGTQIMVNIYALHHNENVWKEPFKFMPE
RFLQTDQDVNGKAMDQSLLPFSAGMRICAGMELGKLQFSFSLANLLNTFQWSCVADGVLPDMSDQLGFVL
LMKTPLEARITPRC
>GFLCYP719-2

MVEISVQWILTTSVVVLVFAIASKMFANGRKSSMKWPEGPKTLPVIGNMHQLGGTALQVVLHKLADVYGS
VMTIWIGSWRPVIVVSDIDRAWEVLVNKSSDYSAREMPEITRINTANWRTISTSDSGPHWSNLRKGLQNV
ALSPLNVAAQSQFQERDINQVIKDLQEEAALNNGIVKPLDHLKKATVRLLSRFIYGQNFHDDKYVESMHY
EIEELIRISGYARLAEAFYYAKYLPSHKKLEKAVEEAHYRVKALISPLLTSDPPPNSYLHFLLSQNHPEE
VIIFCIYEVYLLGVDSTSSTTAWALAFLVREPAVQEKLYQELSNFVGDRQQMGCAVKVEDVNKLQYLQAV
MKETMRMKPIAPLAIPHKACKDTSLGGNKVDKGTRVMVNLHALHHNPKVWKEPNTFKPERFLELKDKEME
QSFLPFSAGMRTCAGMELGKLQFGFALANLVNAFQWTCVADGKLPDMSDLLGFVLFMKTPLEARIVPRVR
>GFLCYP719-3
MGRFLAPDIEMEESFWLVTATVIVVFAVAKFFTRSSSISKMEWPAGPKKLPIIGNLHQLGGNVFHVVLAN
LAKVHGSVMTIWVGSWRPMIVISDIEKAWEMLVTKSSDYSGRSFPEITKIISANWKNISCSDSGPFWTNL
RKGLQGGALAPLNVISQYHLQEKDMENLVKSMRVEASKNNGNIKPLDHLKQETVRLLSRLIFGQDFHDEK
LVVGMHHALDDLVRISGYASLADAFKIAENLPSHKKSIREVHEVNERVKKLVRPHIVSNPPTNTYLYFLQ
SQKFSEDVIISAILEVYDLGVDSTASTTVWALTFLVREQEIQEKLYQEIKSVSGGRSTVKVEEVSKMPYL
QAVMKESMRMKPIAPMAIPHKAQRETTLMGKKVDKDTVLMVNLYAIHHNPNVFPEPFKFMPERFLQGEDH
HNGGNIKAMEQSLLPFSAGMRICAGMELGKLQLAYGLASLVNAFKWDCAVDGKLPDMSEDHCFILLMKNP
LEARITPRTH
>HCACYP719-1
MAEIELGLWLGGAVVAIVLSTILFRSSSSSSSFSSQTKWPAGPKKLPIIGNMHQLGGDVLHVALDKLSKI
HGGVFTFWIGSWRPVIVISDIDKAWEVLVNKSSDYSARDMPEITKIGSASWHTISTSDPGPFWQNVRKGL
QGGAMGPLNMSAQTPFQEKDMKNLIETMKIEAENNNGIIKPLDHIKKNTVRLLSRLIFGQMFEDDKFIES
MHYEIEDIIRISGYARLAEAFYYAKYLPSHKRAEKEAFLVKCRVEDLVRPLLKSNPPSNSYLYFLLSQNY
PEEVIIFCIFELYLLGVDSTSSTTTWALAYLIREQAVQEKLYQDIKVTIGNANLVKVEDVNKLKYLQGVV
KETMRMKPIAPLAIPHKSSKDTTLMGTKVAKGTRLMVNLYALHHNQNIWSEPFKFMPERFLEDGNNNKAM
EQSFLPFSAGMRICAGMDLGKLQFAFALANLVNAFKWSCVEEGKFPDMSEEFSFVLLMKNPLEARITPRK
AH
>JDICYP719-1
MTKSSSSWSTMKWPAGPKTLPIIGNLHQLQGDIFHKALAKLAGVHGGVMTIWLGSWSPVIVVSDIDHVWE
VLVSKSFDYSARPKPDLIKIVAGDDINISDSGTFWYNLRKGLQNVPLGPVNICAQTNYQEGDIEQMIGDM
QEEASSNGGIVKPLYHLKRASTRLLCRLIFGHTFHDDSFVESINNAVEKMIRLGENVHLAEAFSYTKYLP
SHKRAVRRSYELKLAIQELVRPFLVSTPPPNTYLYFLLSQHHPEEVIISCIFELFVLGVDSTSSTTTWAL
AFLIHDQVVQEKLYQEIIQVSNGDCLLRSKDLSKMQYLQAVMKETMRMKPIAPLAIPHITSKHTTLMGTK
VAKGTSVIVNLYALHHNPNIWVDPYKFMPHRFLQGQDDVNVKAMEQSFLPFSGGMRICAGMELAKLQFSL
ALANLVRTFKWTTVLDGNLPDMSEELSFITLMKTPLEARIICRHH
>MAQCYP719-1
MEFTVWVVSFNVLVVVAMSMFLLRTKSSSSTTKWPIGPKKLPIIGNLHLLGGDVLHVALAKLAKVHGGIM
TIWIGSWRPVIVVSDIDKAWEVLVNKSSDYSARDMPEITKIASASWHTISSSDSGPFWQNLRKGLQNGAL
GPANISAQSQLQERDIKRMIGDLQKEAKINGGIVKPLDHIKKAAVRLLSRLIFGHTFDDDSFIESMHHEI
EDLIRISGYARLAEAFSYAKYLPSHKLAVKEAYEVKHRVEQLVRPLLTSSPPANSYLNFLLSQDYSEEVI
TFCIFEIYLLGVDSTSSTTTWALSYLIHEQQVQEKLYQSIKGVTSDDDGLVKAEDLSKLQYLQAVMKETM
RIKPIAPLAIPHKASKDTKLMGTKVAKGTRVMVNLYALHTKENIWSDPEKFKPERFLHGDEGVNVKAMEQ
CFLPFSAGMRICAGMELGKLQFSIALANLVKAFKWTSVVDGELPDMSEELSFVLLMKTPLEARIIPRDL
>MCACYP719-1
MGEGSLWIVATTLVMVGVLIFMMEQSLLWRRRSTQKWPPGPTKFPFIGNLYQLNKGGELVHVTLTKMAQQ
YGRIMTIWFGSQEPTIVVSDHELAWEVLVTKSSDYSSRTLPYLSRVTSADFHTLATCDLGPYWQTLRKGL
QAFTINPHNISSQIHLQEKDIADLVLSLEEEASLNNGVVDPLPKLRRLLILLIRRFCFGPDREFNDDLFV
ERMDCALEDVIRLFGHARLIDVFEFARYIPGIGLGPFQEVKRLKQRIKELIRPYILAHKSSNSACQNCHL
NFLLSQGFTEDVVILNLFELFMLGVDSTSAAIAWALAFVIHEEETQQKLVDEVINKLGRGRRMVGVEEVS
ELEYVQAVVKETMRMKPIAPLPVPHRAVRDSELKGTKVREGTQVLVNLYAVLTDGRVWKEPNRFMPKRFL
QSQRGEDMRAMIMGMERSFIPFGAGRRICAGMELAKPQVALTLANLVYKFQWCSEVEGQLPDLSEDLTFV
LRMKTPLVA

>NDOCYP719-1
MDPMSLWAAIAIVLVMTLLVLLQTRTKSLPSSSTQWPTGPKTLPIIGNLHQLEGDVFHVALAKLAKMHGD
IMTIWIGSWRPVIVISDYDKAWEVLVNKSSDYSARDMPEITKIASAGWRTISLSSSGPFWHTLRKGLQNG
PFGPLNVSAQILSQERDMRRMIRDMQEEADINGGIVKPLGHLKKATVGLLTRLIFGQNFDDDSFIESIHH
ETEELIRISGYARLAEAFSYAKYLPSHRRAVKESYECKHRVEELVKPLLSSNPPKNSYLNFLQSQDYPEE
VIIFCIFEIYLLGVDSTSSTTTWALAFLIHEPAVQEKLYQEVKCITNGDGGLVKAGDLSKFQYLQAVMKE
TMRMKPIAPLAIPHTTSKDTTLMGTKVAKGTRIMVNLYALHHNPNIWSEPFKFMPERFLQEQFNGVGGDA
NVKAIEQSFLPFSAGMRICAGMDLGKLQFSFALANLVNAFKWTTVADGKFPDMNEELSFVLLMKTPLEAR
IVPRKP
>NDOCYP719-2
MEAISLWTVSASFLTLLALAIILPRTRNSSSIINWPPGPKTLPIIGNLHQLGGQLLHVALTNLAKVHGGV
MTIWIGSWRPIIVISSPEEVWEVLVKKSPDYAARDMPEITKLITGYWHTISSSHSGQFWSNLRKGLQNGP
LGPLNITTQMSFQERNIKRMISDIQEEATFNGGIVKPLEHLKKATLRLLSHIIFGQSFDDDQFIDSMHHQ
IEELIRINGFAPLVDAFSYAKYLPSHKRAIKETYRVKNQIEQLVRPVIASSPPANCYLHFLLSQGYSEEV
VIYSIVELYSLGVDSTASTTTWALAFLIHDQTVQDKLYQDIKHIAATDALVKIEDLSALHYLEAVMKETM
RMKPIAPLAIPHKTSKDTTVRGTKVAAGTCVLVNLYALHHDHKIWAKPHKFMPERFLEGNVNGGDSSGAN
VKVIEQSFLPFGAGMRICAGMELGKLQFSFALANLVNAFKWTSVVDGKLPDMSEELKFIMLMKTPLEARI
IPRNSLN
>PBRCYP719-1
MIMSNSWILTLISTILSVVFAAVLIIFRRRISASTTEWPVGPKTLPIIGNLHILGGTALHVVLHKLAEVY
GSVMTIWIGSWKPVIIVSDFDRAWEVLVNKSSDYSAREMPEITKIGTANWRTISSSDSGPFWATLRKGLQ
SVALSPQHLASQTAHQERDIIKLIKNLKDEAALNSGTVKPLDHLKKATVRLISRLIFGQDFDDDKYVEDM
HDVIEFLIRISGYAQLAEVFYYAKYLPSHKRAVTGAEEAKRRVIALVRPFLQSNPATNTYLHFLKSQHYP
EEVIIFAIFEAYLLGVDSTSSTTAWALAFLIREPSVQEKLYQELKNFTANNNRTMLKVEDVNKLPYLQAV
VKETMRMKPIAPLAIPHKACKDTSLMGKKVDKGTKVMVNIHALHHTEKVWKEPYKFMPERFLQKQDKAME
QSLLPFSAGMRICAGMELGKLQFSFSLANLVYAFKWSCVSDGVLPDMSDLLGFVLFMKTPLEARVVPRL
>PBRCYP719-2
MEENSWFITATITVIVVFVLAKFLASKIFSPTTMEWPAGPKTLPIIGNLHQLKGDLFHVVLQNLAKVHGG
VFTIWIGSWNPVIIVSDFNIAREVLVSKSLDFSARSVPDYVKIVSAGGKTISESDCGPFWHGLRKGLQGV
ALNPLHLMSQSHLQERDMQNLIKSMQESASQEDGVIQPLDYIRKATIRLLSRILIGQDFSNEDFVDSMNH
TLHKLVSIGGFASLADAFKIGEYLPTHKKFIRELKDVSEEAANLILPYIVSKPSKNTYLHFLVSQEYSED
VIVSAVLEIFGMSSDSTAAATVWALAFLVRHPDIQEKLYQEIKNVTGGNRPVQIVDVKKMPYLQAVVKET
LRMKAVGPMGISHKASKNTSLMGKKIDKGTQVMVNHYAIHHNSDIFPEPYNFKPERFFKDVNSDASLGDR
EKMESSLLPFGAGMRVCAGTDIAKLIISFGIASLVNEFKWSCVSDGKLPDLSEDLSFILLMKNPLEARIA
PRVD
>PBRCYP719-3
MAPINIEENDFWMIACTVIIVFALVKFMFSFYQSANTTEWPEGPKTLPIIGNLHQLGGGVPLQVALANLS
KVYGGAFTIWIGSWVPMIVISDIDNAREVLVNKSADYSARDVPDILKIITANGKNIADCDSGPFWHHLKK
GLQSCINPSNVMSLSRLQEKDMQNLIKSMQERASQQNGILKPLDHAKEASIRLLSRVIFGQDFSNEDLVI
GVKDALDEMVRISGLASLADAFKIAKYLPSQRKNIRDMYATRDRVYNLIQPHIVSNLPANSFLHFLTSQD
YSDEIIYSMVLEIFGLGVDSTAATAVWALSFLVGKQEIQEKLYREINNLTGGQRPVKVADLKELPYLQAV
MKETLRMKPIAPLAVPHVAVKDTTFKGRRIVKGTKVMVNLYAIHHDPNVFPAPYKFIPERFLKDVNSDGR
FGDINTMESSLIPFGAGMRICGGVELAKQMVGFALASMVNEFKWDCVSEGKLPDLREAVSFILYMKNPLE
AKITPRDARSYKTFGQ
>SCACYP719-1
MEFEKIMMSNPWILAATTAVVFISITAIFFRRKSSSMEWPTGPKTLPIIGNMHLLGGTALHVVLHNFAKI
YGNVMTIWIGSWRPVIVVSDIDRAWEVLVNKSSDYSARDMPEITKIISADWKTISTSDSGPFWTNLRKGL
QNVALSPHNLAAQARFQERDISKMIETLKEEAALNNGIVKPLDHLKKATVRLISRLIYGQEFDNNKYVED
MHHAIEELIRVSGYARLAEAFYYAKYLPSHKKAVRDVEEANRRVKNLVRPFLSSNPPTNCYLHFLRTQNY
PEEVIIFAIFEAYLLGVDSTSSTTAWALAFLVREPNVQEKLYQELKNFTTNNDRKMLKVEDINKLQYLQA

VIKETMRMKPIAPLAIPHKACKETSLMGSKVDKGTRVMVNIYALHHNQKVWKEPYKFMPERFLETDNQDG
NVGKAMEQSLLPFSAGMRICAGMELGKLQFTFSLANLVNAFKWSCVSDGVLPDMSDQLGFVLLMKNPLEA
RIVPRS
>SCACYP719-2
MEEMISLQWILTAVIVVVVVAMASKIFSNRRKSSMKWPDGPKTLPIIGNMHQLGGTAGLQVVLHKLADVY
GSIMTIWIGSWRPVIVVSDIDKAWEVLVNKSSDYSARDMPEITRINTANWRTISTSDSGPFWSNLRKGLQ
NVALSPHNLAAQSQFQERDINRLIKNLKEEAALNDGIVKPLDHLKKATVRLLSRLIYGQNFNDDKYVETM
HNEIEELIRISGYARLAEAFYYAKYLPSHKRVEKAVEEAHYRIKALISPLLSWNPPLNSYLHFLRSQNYP
EEVIIFCIYEVYLLGVDSTSSTTAWALAFLVREPAVQEKLYQELNNFTHDRQMSAVKVEDVNKLPYLQAV
MKETMRMKPIAPLAIPHKTCKETSLGGNKVDKGTRVMVNLHAIHHNPKVWHEPYRFLPERFLQVNKETIN
NGGGGGGTAKEMEQSFLPFSAGMRICAGMELGKLQFGLALANLVNAFQWSCVADGKLPDMSDLLGFVLFM
KNPLEARIVPRIR
>SCACYP719-3
MEESFWLVTVSVIVLFAVAKFFRKSSSIPKMEWPAGPKKLPIIGNLHQLGGDVFHVVLANLAKVYGSVMT
IWVGSWRPMIIVSDIDKAWEVLVSKSSDYSARDMPDITKIISANWKNISCSDSGPFWHNLRKGLQGGALA
PLNVISQYHLQERDMQNLINSMQQEASKNNGILKPLDHLKQETVRLLSRLIFGQDFHDEKLVVGMHHALD
DLVRISGYASLADAFKFAENLPSHKKSIREVHEVKERVENLVRPHIIPNPPTNTYLHFLQSQGFSEDIVI
SGILEVYDLGVDSTASTTVWALTFLVREQKIQEKLFEEIKKVTGGRSSVRVEEVSKMAYLQAVMKETMRM
KPIAPMAIPHKTSKETSLMGRKVDKGSVIMVNLYAIHHNPEVYPEPYKFKPERFLQGSENIKAMEQSLLP
FSAGMRICAGMELGKLQLAFGLASLVNAFKWDCAVDGELPDMSEDHCFILLMKNPLQARITPRTH
>SDICYP719-1
MESLIDFSNPAKMEELMSNPWIITAITLAVVIIMMTIFRRKSSSMKWPKGPKTLPIIGNMHLFGGTDLHV
VLYNIAKVYGNVMTIWIGSWRPVIVISDIERAWEVLVNKSSDYSARDMPEITKLISSDWKTISTSDAGAF
WTNLRKGLQNVALSPHNLASQFQFQESDIKKLIETIKEEGAVNNGIVKPLDHLKKAMVRLISRLIFGQDF
DNDKYVEGMHHAIEELIRVSGYARLAEAFYYAKYLPSHKKAVRDVEEANRRVQKLVRPFLSLNPPTNTYL
HFLRSQNYSEEVIIFAIFEAYLLGVDSTSSTTAWALAFLIREPNVQEKLYQELKNFTVKNGRDMLKVEDI
NKLPYLQATIKETMRMKPIAPLAIPHKACRDTSLMGSKIDKGTRVMVNIYALHHNEKIWKEPFKFMPERF
LQTEGEVNGKAMEQSFLPFSAGMRICAGMELGKLQFNFALANLVNAFKWSCVADGVLPDMSDQLGFVLVM
KTPLEARITPRC
>SDICYP719-2
MEESFWLVTATVVVVFVIAKLQFRKSSSISTMEWPAGPKTLPIIGNLHQLGGAALHVVLANLAKAYGSVM
TIWVGSWRPMIVISDFDKAWEVLVNKSSDYSARSLPEITRIISANWKNIMTSDSGPFWQNLRKGLQSGAL
SPHNVMSQYQLQERDMQNLIKTMRVEAFKNNGRIKPLDHLKQETVRLLSRLIFGQDFNDEKLVVGMHHAL
DDLVRISGYASLADAFKFAENLPSHKKSIREVHELKKRVENLVRPHIVSNPPTNTYLNFLQSQNFSEDLI
ISAILEVYDLGVDSTASTTVWALTFLVRELKVQEKLYQEIKNLTGGRSTVKVEEVSKMPYLQAVMKETMR
MKPIAPMAIPHTAARETSLMGKKIDKGTVVMVNLYAIHHNPNIFPEPYKFMPERFLHGEEQNGGNIKEME
QSLLPFSAGMRICAGMELGKLQLGFALASLVNAFKWECAVDGKLPDMSEDHCFILLMKNPLEAKITPRTH
>TFLCYP719-1
MEVNLWLVSATFATLLAITTLIRMFKSSSQMKWPSGPKTLPIIGNMHQLGGDLLHVVLAKLAKVHGGVMT
IWIGSWRPIIVVTDIDKVWEVLVSKSADYAARDFPEFTNFVTASRHTISSSDLGAFWQTLRKGLQNGALG
PINIAAQSQFQERDMKQMTQAMVDEAGRNNNIVKPMEHIKKNSVRLLTRLIFGETFDDDEFVDSMLYDVD
DVIRIGGFARLAEAFYYAKYLPGHRKAVREANLLKLRVEKLIRPFFSSNPPKNSYLYFLLSQNIPEEVII
FCIFELYCLGVDSTSSTTTWALAYLVHEQAVQEKLYQEIRMTLGDVDQVRIEDVSKMKYLQAVVKETMRM
KPIAPFAIPHMTAKDTTLMGTKVAKGTSIMVNLYALHHNPDIWTEPYKFIPERFMQGEDGSATNKAMERS
FLPFGAGMRTCAGMDLGKLQFAFVLANLVNAFKWSCVEEGKLPDLSEDLAFVLLMKTPLEAKITPRKS
>XSICYP719-1
MEMSPTLLVCATVAIVFTITTIIRNLFSSSSFSQMKWPTGPKKLPVIGNLHQLGDDVLHVALAKLAKVHG
SVMTIWIGSWRPVIVISDIDKAWEVLVNKSADYGARDMPEITKIASASWHTISTSDAGPFWQNVRKGLQS
GAMGPLNVAAQNQFQERDMKRLVNAMSDEALNSKGIVKPLDHIKKNTVRLLTRLIFGQTFDDDKFIESMH
YEIEDIIRISGYARLAEAFYYAKYLPSHKKAEREAFLVKCRVEKLVRPLLSSNPPSNSYLYFLLSQNFEE

EVIIFCIFELYLLGVDSTSSTTTWALAYLIREQGAQEKLYQDIRMTLGDVDLVKIEDVSKLKYLQGVIKE
TMRMKPIAPLAIPHKTAKETTLVGTKVAKGTRIMVNLYALHHNENIWPEPYKFMPERFLEGEAKTAYNKA
MEQSFLPFSAGMRICAGMDLGKLQFAFALANLVNAFKWSCVEEGKFPDMGEELSFVLLMKTPLEARIAPR
NV
>AMECYP719A13
MEEKIMTNNSPWILTSSTTTTTTILLSLLFTIFIILRRNKSSSSKMVWPTGPKTLPIIGNMNILGGTALH
VVLHNLAKTYGNVMTIWIGSWRPVIVVSDIDRAWEVLVNKSSDYSARDMPEITKLATADWKTISSSDSGP
FWTNLRKGLQNVALSPQNLSSQSKFQERDIIKTIQNLKEEAKMNNGIVKPLDHLKKAMVRLISRLIYGQD
FDNDEYVEEMHHTIEELIRVSGYARLAEAFYYAKYLPSHKKAVREVLQANQRVQNLVRPLLSLNSPTNTY
LHFLRSQNYEDEVIIFAIFEAYLLGVDSTSSTTAWALAYLIREPNVQEKLYEELKNFTNDNDRKMVKFED
LNKLQYLQAVVKETMRMKPIAPLAIPHKACRETSLMGRKVNQGTRVMVNIYALHHNQNVWKEPYKFNPER
FLQKNQDGVDGKAMEQSLLPFSAGMRICAGMELGKLQFSFALANLVNAFKWSCVSDGVFPDMSDQLGFVL
LMKTPLEAGIVPRM
>AMECYP719A14
MDETIWLIISTVIIVLGIAKFLLGKSSSSSLSTMEWPVGPKKLPIIGNLHQLGGDVFHVVLANLAKVYGS
VFTIWVGSWRPMIIVSDIDKAWEVLVNKSSDYSARDMPDITKIISANWKNISCSDSGPFWHNLRKGLQGV
ALTPLNVASQYHLQERDMKNLINSMYKDASRKNGILKPLDYLKEETVRLLSRLIFGQDFQDEKLVVGMHH
ALDDLVRISGYASLADAFKFCENLPSHKKSIREVHEVKKRVENLIRPHIVSNPPTNTYLYFLKTQDFNED
IIISAILEVYDLGVDSTASTTVWALTFLVREQEIQEKLYREIVNVTGGKRSVKVEDVNKMPYLQAVMKET
MRMKPIAPMAIPHKTSKDTSLMGKKINKGSVIMVNLYAIHHNPKVFPEPYKFMPERFLKDVNSDESLGNI
KTMESSLLAFSAGMRICAGMELGKLQLAFGLASLVHEFKWSCSVDGKLPDLSEDHCFILLMKNPLEAKIT
CRIH
>CJACYP719A1
MEMNPLLVCATVAIVFATTTIIRILFSSSSLPQMKWPSGPRKLPIIGNLHQLGDDVLHVALAKLAKVHGS
VMTIWIGSWRPVIVISDIEKAWEVLVNKSADYGARDMPEITKIASASWHTISTSDAGSFWQNVRKGLQSG
AMGPLNVAAQNQYQERDMKRLIKAMSDEAANNNGIVKPLDHIKKNTVRLLTRLIFGQAFDDNKFIESMHY
EIEDIIRISGYARLAEAFYYAKYLPSHKKAEREAFLVKCRVEELVRPLLSSKPPTNSYLYFLLSQNFEEE
VIIFCIFELYLLGVDSTSSTTTWALAYLIREQGAQEKLYQDIRMTLGDVDLVKIEDVNKLKYLQGVVKET
MRMKPIAPLAIPHKTAKETTLMGTKVAKGTRIMVNLYALHHNQNIWPDPYKFMPERFLEGETGTAYNKAM
EQSFLPFSAGMRICAGMDLGKLQFAFALANLVNAFKWSCVEEGKLPDMGEELSFVLLMKTPLEARIAGRN
V
>ECACYP719A2
MEEMKILMMNNPWILTATATTLLISIFLFFTRKSSKMVWPAGPKTLPIIGNMHLLGGTALQVVLHNLAKV
HGSVMTIWIGSWRPVIVVSDIERAWEVLVNKSSDYSARDMPDITKIISADWKTISTSDSGPHWTNLRKGL
QNVALSPHNLAAQFQFQEKDMTKMIQTLEEEARNNNGIVKPLDHMKKATLRLISRLVFGQDFNNDKYVDD
MHLAIEELIRVSGYARLAEAFYYAKYLPSHKKAVREVEEAQRRVQNLVSPFLSLNPPTNTYLHFLRSQKY
DDEVIIFAIFEAYLLGVDSTSLTTAWALAFLIREPNVQEKLYQELESFASKNDRRILKVEDINKLQYLQA
VIKETMRMKPIAPLAIPHKACRDTSLMGKKIDKGTRVMVNIFALHHNKNVFNDPFKFMPERFMKVDSQDA
NGKAMEQSLLPFSAGMRICAGMELGKLQFSFALANLAYAFKWSCVADGVLPDMSDQLGFVLLMKTPLEAR
INRRN
>ECACYP719A3
MEEMKFLIMNNPWVLFATSATLLISIFLFFRRKSPNMAWPEGPKTLPIIGNMHLLGGTALQVVLYNLAKV
HGRVMTIWIGSWRPVIVVSDIEQAWEVLVNKSSDYSARDMPDITKIVTADWRTISTSDSGPHWSNLRKGL
QNIAISPNNLAAQFQFQEKDIIKMIQILEQEAKDNNGIVKPLDHLKKATIRLISRLVFGRDFEEDKYVED
MHHAIEELIRISGYARLAEAFYYAKYLPSHRKAVRYVEELKQIVKNLIRPFLSVNPPTNTYLHFLRSQNY
DEEVVIFAIFETYLLGVDSTSSTTAWALAYLVREPSVQDRLHQELDHFAKQNDRKILKVEDMNKLQYLQA
VIKETMRMKPIAPLAIPHKACKDTSLMGNKINKGTRVMVNLYALHHNKNVFNDPFKFMPERFLKVDNQDA
KGKAMEQSLLPFSAGMRICAGMELGKLQFSFALANLIFAFKWSCVDDGVLPDMSDELGFVLLMKTPLKAR
INPRN
>ECACYP719A5

MEESLWVVTATVVVVFAIAKLLKKSSSISTMEWPKGPKKLPIIGNLHQLGGEAFHVVLANLAKIHGTVMT
IWVGAWRPMIVISDIDKAWEVLVNKSSDYAGRDFPEITKIISANWKNISCSDSGPFWQNLRKGLQGGALA
PLNVISQYQLQERDMKNLITSMQEKASKNNGILKPLDYLKEETIRLLSRLIFGQSFNDENFVKGVHLALD
DLVRISGYASLADAFKFCENLPSHKKSIREVHEVNERVVNLVKPYLVKNPPTNTYLYFLNSQKFSDEVII
SAVLEVYDLGVDSTASTAVWALTFLVREPRVQEKLYKEIIDLTGGERSVKVEDVSKLPYLQAVMKETMRM
KPIAPMAIPHKTSRDTSLMGKKVNKGTSIMVNLYAIHHNPKVFPEPYKFIPERFLQGQESKYGDIKEMEQ
SLLPFSAGMRICAGMELGKLQYGFSLASLVEAFKWTCAVDGKLPDLSEDHCFILLMKNPLEARITPRTQL
>PSOCYP719A19
MIMSNLWILTLISTILAVFAAVLIIFRRRISASTTEWPVGPKTLPIIGNLHILGGTALHVVLHKLAEVYG
SVMTIWIGSWKPVIIVSDFDRAWEVLVNKSSDYSAREMPEITKIGTANWRTISSSDSGPFWATLRKGLQS
VALSPQHLASQTAHQERDIIKLIKNLKDEAALNSGMVKPLDHLKKATVRLISRLIYGQDFDDDKYVEDMH
DVIEFLIRISGYAQLAEVFYYAKYLPSHKRAVTGAEEAKRRVIALVRPFLQSNPATNTYLHFLKSQLYPE
EVIIFAIFEAYLLGVDSTSSTTAWALAFLIREPSVQEKLYQELKNFTANNNRTMLKVEDVNKLPYLQAVV
KETMRMKPIAPLAIPHKACKDTSLMGKKVDKGTKVMVNIHALHHTEKVWKEPYKFMPERFLQKHDKAMEQ
SLLPFSAGMRICAGMELGKLQFSFSLANLVNAFKWSCVSDGVLPDMSDLLGFVLFMKTPLEARIVPRL
>PSOCYP719A25
MEVTFWLITCGVVVAFALAKLLFGKKSSMSTMEWPSGPKTLPIIGNLHQLGGEAFHVCLANLAKVYGGVF
TIWVGSWRPFIVISDVDKAWEVLVNKSSDYSARDMPDITKIISANWKNISHGDSGPFWHNLRKGLQGVAL
TPFNVASQYHLQERDMQNLIKSMKKKASQKNGILKPLDYVKEETVRLLSRLIFGQDFVDEDFVVGMHQAL
DELVSISGYASLADAFKFCENLPSHKKTVRAVHAIKIRFDNLIRPHIVSNPPTNTYLHFLLSQDFSEDVI
ISAILEVYDLGVDSTASTTVWALTFLVREQKIQEKLYREINNVTGGKKPVKVEDLNKLPYLQAVMKETMR
MKPIAPMAIPHKASKDTSLMGKKINKGAVVMVNLYAIHHNPAVFPEPYKFMPERFLKDANSDGSLGDIKK
MESSLLAFSAGMRICAGMELGKLQLAFGLASLVNEFKWDCFAEGKLPDLSEEHCFILLMKNPLEAKITPR
IH
>PSOCYP719B1
MAPINIEGNDFWMIACTVIIVFALVKFMFSKISFYQSANTTEWPAGPKTLPIIGNLHQLGGGVPLQVALA
NLAKVYGGAFTIWIGSWVPMIVISDIDNAREVLVNKSADYSARDVPDILKIITANGKNIADCDSGPFWHN
LKKGLQSCINPSNVMSLSRLQEKDMQNLIKSMQERASQHNGIIKPLDHAKEASMRLLSRVIFGHDFSNED
LVIGVKDALDEMVRISGLASLADAFKIAKYLPSQRKNIRDMYATRDRVYNLIQPHIVPNLPANSFLYFLT
SQDYSDEIIYSMVLEIFGLGVDSTAATAVWALSFLVGEQEIQEKLYREINNRTGGQRPVKVVDLKELPYL
QAVMKETLRMKPIAPLAVPHVAAKDTTFKGRRIVKGTKVMVNLYAIHHDPNVFPAPYKFMPERFLKDVNS
DGRFGDINTMESSLIPFGAGMRICGGVELAKQMVAFALASMVNEFKWDCVSEGKLPDLSEAISFILYMKN
PLEAKITPRTKPFRQ
>PSOCYP719A20
MEKTIFSNPWILTTSTLVVITIITMLMVFKRKKSSTMVWPTGPKTLPIIGNMHVLGGTALQVVLHNLAKI
HGNVMTIWIGSWRPVIVVSDIDRAWEVLVNKSSDYSARDMPEITKIISADWKTISTSDSGPGWSNLRKGL
QNVALSPHNLAAQSQFQEKDITKMIQSLKKEAASNNGIVKPIDHLKKATLRLISRLIFGQDFDDDKYVDD
MHHAIEELIRVSGYAQLAEAFYYAKYLPSHKKAVRDVEEANQRVKKLVRPFLSLNPPTNTYLHFLNSQKY
DEEVIIFAIFEAYLLGVDSTSLTTAWALAFLIREPVVQDKLYQELKNLTAKNDCEIVKVEDLNKLQYLQA
VLKETMRMKPIAPLAIPHKACRDTSLKGNKIDQGTQVMVNIYALHHNEKVWKEPFKFMPERFLQTQDVVN
GKGMEQSLLPFSAGMRICAGMELGKLQFSFSLANLVNAFEWSCVSDGVLPDMSDQLGFVLLMKTPLQARI
VPRV
>TFLCYP719A1
MELVSLWLVSATLALVVAITILIPMVTSSSSFSQMKWPTGPNKLPIIGNLHQLGGDVLHVALAKLAQVHG
SVMTVWIGNWRPIIVISDIDKAWEVLVNKSSDYGARDMPEITKIASASWHTISTSDAGAFWQTVRKGLQS
GAMGPLNIAAQSQFQERDMKRLIQALRDEAAQNNNIVKPLDHIKKNTVRLLTRLIFGQTFDDDKFVESMH
FEIDDIIRISGYARLAEAFYYAKYLPSHKKAEREAYLVKCRVEDLVSPLLSSNPPTNSYLYFLLSQRFSE
EVIIFCIFELYLLGVDSTSSTTTWALAYLIREQAIQEKLYQDTRMTLGDVDLVKIEDVSKLKYLQAVVKE
TMRMKPIAPLAIPHKTAKDTTLMGSKVAKGTRIMVNLYALHHNQNIWTNPYKFMPERFLQGEDGSASNKA
MEQSFLPFSAGMRICAGMDLGKLQFAFALANLVNAFKWSCVEEGKFPDMSEELSFVLLMKTPLEAKITAR

KF

DIOX

>ATHDIOX
MVAVERVESLAKSGIISIPKEYIRPKEELESINDVFLEEKKEDGPQVPTIDLKNIESDDEKIRENCIEEL
KKASLDWGVMHLINHGIPADLMERVKKAGEEFFSLSVEEKEKYANDQATGKIQGYGSKLANNASGQLEWE
DYFFHLAYPEEKRDLSIWPKTPSDYIEATSEYAKCLRLLATKVFKALSVGLGLEPDRLEKEVGGLEELLL
QMKINYYPKCPQPELALGVEAHTDVSALTFILHNMVPGLQLFYEGKWVTAKCVPDSIVMHIGDTLEILSN
GKYKSILHRGLVNKEKVRISWAVFCEPPKDKIVLKPLPEMVSVESPAKFPPRTFAQHIEHKLFGKEQEEL
VSEKND
>BTHDIOX1
METGLVAPIPLSVQNVQDLSTKNLDVIPSRYIRPELANDIISIDEGSKIPIIDLSKLTDEQFCQDELERF
HLACKDWGFFQLINHGVQQDALENVKREVTEFFKQPFEVKKEFSQQPGEVEGYGQAYVFSDEQKLDWGDM
LYLITQPDVARNMKLWPTSPPTFRETIVKYSEELQKVKMCVMASMAKNLEIEPEKFTNMFEKGIQSMRFN
YYPPCVEDDKVLGISPHSDATGITFLIQVNHVHGLQLRHKGNWVPVVLTTDAILVNIGDVLEILSNGKYK
SIEHRVVINKTKERLSVAAFHEVNDNVMIGPLPELIKQGEDKYYKSLSFVDYTKLLNSEKLDGKTLIEQM
KLKN
>BTHDIOX2
MEVDEIRSYGASLPVENVQALAAKDLKEIPERYIRPELEFDVVVSIENSGEIPVIDLSRLLDEHFAHDEL
AKLHSACEEWGFFQLINHGASEEVIEKMKTDTQEFFRLPLEEKKAYTQVSNGIEGYGQAFVFSEEQKLDW
GDMLFLFALPINQRNFKFWPTNPTSFRETTEKYSLEVHKVAICLLGLIAKNLGIEPEKFTKVFEDGMQHF
RLNYYAPCPHAHKVLGLSPHSDGSALTLLTQVNDDVDGLQIKKNGNWMPVKPISGAFIVNIGDVIEITSN
GKYKSIEHRAIIDPKKERLSIAAFHNQNFRTTIGPFPELVKGNSTEKYKTVSYQDYLRNITSTGLDGKNR
LEFMKL
>CCHDIOX1
MEEVKEELSPKALVNSGSSLLVPSVKELAKEQLTEIPSRYIRFDQESPVTTFQPTCLPSVPVIDIHSLLF
QESEAATASELNRLHSACKNWGFFQIINHGVSSSLIEKIKLEIKAFFELPMEEKQKLWQQPCDQEGFGQL
FVVSEEQKLDWCDMFFIRTLPTTIRKPHLFEKLPVPLRETLENYSLEMKKLAKTVLEQMAKALKMDAEEM
RLLFDDGFQEMRLNYYPLCSQPEKTIGFTPHSDASALTILLQLNETEGLQIRNEGKWVPVKPLPNAFVVN
IGDVMEIVSNGIYHSIEHRAVVNSTMERLSIATFYRTNLEAEIGPARSLIGPHKPAVFKRVMTEKYYKDF
FAREIKGKSYLDSMRINGTGEEKSS
>CCHDIOX2
MAPAPISSINVGHIDDVQELRKIQPTKIPERYVRDIAERPTKTHARSPMQIPVIDLSKLMRNDKEMAQNE
LSKLAYSCENWGFFQVINHGIDTELIECIEEVAKKFFMLPLEEKQKFPMAPGTLQGYGHAFVFSEDQKLD
WCNMLALGVEPFFIRNPKLWPTNPAEFSETLDLYSANIRKLCKILLTFIAMSLGLNDDMFNDMFGTAVQA
VRMNYYPPCSRPDLVLGLSPHSDGSALTVLQQAKGSSVGLQIRKDDKWVPVKPVPNALVINIGDTIEVLT
NGKYKSVEHRAVTNKEKDRLTIVTFYAPSYDIELGPLPECIDENRPCMYKKYNHGEYSKHYITSKLEGKK
TLDFXXXXXXXXXXXXXTKI
>CCHDIOX3
MEEIHLVSTVADDHISSIVNQRVQDFAINVKEPPSRYIRKAINDDIDFGLNQTTTVDXXKDDSLSSSSVP
IIDLQRLSSSSIEEKKAEMEKLRSALASWGLFQAVGHGIPNTLLDEVRNISKQFFELPSEEKQKYSKLGG
VDTFDDLEGYGNDPVTSGDQVLNWNDRLYLLVKPEEQRKLNFWPEHPNHFRDTLHEYSVKSSIAAEFILK
TMSELLGLKEKYLLNQLGEQAPVYVRFNYYPPCPRPDLVYGMTPHSDVGGITIILQDEVEGLQVLKDEKW
INVPTIPHALLVNMADLMEMMSNGQFKSPVHRVITNAEKERISLAMFFSPELDKEIQPAAELIDDDKPRL
FKKVIVKEYFKYFFPRYLEGKRAIDWAKV
>CCHDIOX4
MEVKTLAESRLPHVPPQYIQPVENRPAIDSCREKSSDFGIPVIDLFNFDPQCSSGACQELGRACRDWGAF
QIINHGVPKRLLDEMMTAGLSFFNECSSAEKRKYSCDPNSAASEGYGSRMLVKEDTVLDWRDYFDHHTLP
LSRRNPNRWPQSPSSYRGVVVEYSDHIKSLAVKLMSMISESLGLPGSCIEDIVGEIYQNITISYITISYY

PPCPQPELTLGLQAHSDIGAITLLIQDDVGGLEVLKGEEWVTVQPLSGAIVVILADQTEIITNGKYRSSV
HRAVTNSHRARLSVGTFHDPSKSRKICPATGLISKDSPAKYREVVYGDYISAWYSKGPEGKRNIDALLIH
E
>CCHDIOX5
MKNEMIIEKGLGGSLPVESVQALASKDLKDIPARYIRPELESDVVNTDGSLEIPVIDLSRLLDQQFMYEE
LKKFHSACEDWGFFQLINHGVPEEVVDKMKVDVEEFFKLPLEEKKPYSQLPGVLEGYGQAFVVSEEQKLD
WGDMLFLHAQPVVGRNMTLWPKHPTSFRATLDKYSSELQRVTVCLIECMAKNLGFVDPEKLTSMFEIGWE
GVRMNYYPPCVVNADKVLGISPHSDATGLTLLIQVNEVQGLQIRKNGNWVPVKPIPGAFIVNIGDMIEIM
SNGKYKSIEHRAVIDPLKERMSIAAFHSPEPKTIGPLPEMVKMSGGVEKYKTLNYVEYLKLLYSSKLDGK
NLLEFMKLDQGM
>CCHDIOX6
MEEEKEELSPKALVNFGSSLLVPSVKELAKKQLTEIPSRYIRFDQESPVTTFQPTCLPSVPVIDIHSLLF
QESEAATASELNRLHSACKNWGFFQIINHGVSSSLIEKIKLEIKAFFELPMEEKQKLWQQPCDQEGFGQL
FVVSEEQKLDWCDMFFIRTLPTTIRKPHLFEKLPVPLRETLENYSLEMQKLAKTVLEQMAKALKMDAEEM
RQLFDDGFQAMRLNYYPLCSQPDKVLGFTPHSDSCALTILLQLNETEGLQIRNEGKWIPVKPLPNAFVVN
IGDIMEIVSNGVYRSIQHRAVVNSMKERLSFVTFYSTNIDAEIGPACSLIEPHKPALFKRVMTEKYYKDF
FAREIKGKSYLDSMRINVSGES
>CCHDIOX7
MGFVKNLSESTILTSIPSDYDFSTINSDYSPSVTTEEESIPIIDFSLLTSSSPQQRSQIVQDLGNACLQW
GFFMVVNHGIPERLKEEVFQSCQDFFDLSEDEKLGYTAGKHVLDPIRCGTSFNASVEKVFFWRDFLKVFV
HPEFHSPKKPIGFSGISNEYSKRVREIARELLKGISESLGLEASYIEKAMELQSGLQIFVANLYPPCPQP
ELAMGMPPHSDHGLLTLLMQNDVGGLQVKHKGKWVHVNPLPNSILINTGDHLEILSNGKYKSVLHRATVN
NNTTRISIAVAHGPSLDTLVTPAPGLVISEIHPPRYHGMKYKEYLELQQSNQLDGKSCLDRVRVLNV
>CMUDIOX1
MTTWVPSLPVPNVQDLAAKKLEEVPDRYIQREAESENVAIELSDQVPVIDLQRLLHHDPSIASDEMAKFH
MACKDWGFFQLINHGMDEELMRKMVNNLEEFFNLPLEEKKAYAQTPESFEGYGQAFVASKEQKLDWSDML
GLQTLPVNRRAMRVWPKKPLSFSETIDKYAFGMQKVTFCLLSLIAKNLGVEPQKLTSIFEDGIQGTRMNY
YSPCPQSDKVLGLSPHSDANLITLLIQNNDLPGLQIKIDGKWIPIKPISGAFIVNIGDSIEILSNGLYKS
IEHRAVIHADRARISIAGFQAPRSGVVIEPLPELLEKENVVANYKSVDYEEYLRQYFSAKLNGKSMLDLI
KLKIHKDEERA
>CMUDIOX2
MESLSIDASSVLDLVSKNPETIPSRYIRPENDHQIVVTSIHNEVPVIDLSKLIFDANNGGVSDEEMKNLH
LACQEWGFFQLVNHGVDEEAMKKIVEDVREFFELPLKLKKECSSEMEGYGQAFVLSEEQKLDWGDMFFLF
TGPDSIKNYEVWPAHRPFTFRETINKYSSEMEKIKIILVGLMAKNLGIDQDKFINLFKDGVQSMRMNYYP
PCPDPSKVLGISPHSDATGITILLQLNEVDGLQIRHGGNWVPVKMLPGALLVNIGDIIEILSNGIYNSIE
HRVVINKEKERMSVATFHETHNSVDIGPLPELVINGETKYKSVAWDEFSELFLSQKLDGKSLLDALKLN
> CMUDIOX3
MLREKMTIEAKEKLISIHSISEEDQLISMDHQVPLTLGSSLLVPSVQEMARKSALDAIPKRYIRDDLRQE
LMNVTRSDETDRLLPSLPVIDMKALLDERDVELEKLHSACKDWGFFQLVNHNVDENLVEKVKGVIQDLFN
HPIEEKQKYWQKPGDLEGFGQAFVVSDEQKLDWADMFYMVTQPEHSRKPHLFPLLPHPIRETFEEYSIEL
KNLALKVLKMMAKALKMDVEEMNSLFAEGKQAFRINYYPPCPQPEKVIGLTPHSDAVGLTILLQVNDVEG
LQIRKDGMWVPVKPLPNGFIVNVGDILEMVTNGNYRSIEHRAVVNSSKERLSIATFYSPGVDAEIGPAPS
LINSNSPPIFKRINGEDYFKGLFARELAGKSYLDCMRI
>CMUDIOX4
MATEQEEYFPSLPVPNVQDLSSNLANDIPDRYIRPEFQSEFVCDDKSFAIPVIDFSRILDEKFRDHEMTK
LHAACQEWGFFQLINHGSEEAIEEIKSHVEEFFKQPLAAKKAYTQERNSIEGYGQAFVQSEDQKLDWGDM
FLVRAFPVRKRNMRFWPNYPSTFRGTLDRYSSEVRKLAIGLLGLMAENLGLDAEKLTGMFGDLQQSLRMN
YYPPCPQADKVVGISPHSDPLPLTLLIEANNVQGLQIRKNSIWVPVHAIPGAFIINIGDMLEIWSNGKYK
SIEHRVVVNPYRERLSVAAFHAPNEDSIVGPLQDLVNKEGAKYKSIDANSYYKLKNTIQGKMHANEMKLV
TPTTN

>CMADIOX1
MSCSCPQVDWPEPIVRVQSISDSGSPVIPDRYIKPPLERPSKLGLARTSITSTNDQDVLNIPLIDLGGLL
IQDEDNLILPDSILNQICMACQNWGFFQIVNHGVSPDLLHRVREVWREFFHLPYEHKQVYANSPKTYEGY
GSRLGVEKGAILDWSDYFYLHYLPQCLKDPNKWPALPTSCREVVDEYCCQLVKLSKNLLKILSITLGLGE
SYLENAFGGEEIGACMRVNFYPKCPQPDLTLGLSSHSDPGGMTLLLPDNQVVGLQVRKDDNWVTVKPAPN
AFIVNIGDQVQVLSNAIYKSVEHRVVVNSVEERVSLAFFFNPRGDLPLQPAKELVTSDRPALYLPMTFNE
YRLYIRTQGPRGKVYMESLKSPR
>CMADIOX2
METPKPVTLGGSLPVPSVQELAKQPLLQVPPRYIRSDQDLPIISESDDVSATFQTVPVIDLKSLLCGDTE
LERLHSACREWGFFQLVNHGVNTSLVEKVKSEIQDFFKLPMEEKKKYWQKEGDVEGFGQDFVISEEQKLD
WADIFFMITLPRHARKSHLFPELPLPLRETLESYSSEMKNLAMSLFEVMSKALDVETQLVTELFEDGMQN
MRMNYYPPCPQPELVVGLTPHSDAVGLTILLQLNEMEGLQIKREEKWVPIKPLPNAFIVNIGDILEIVTN
GIYNSVEHRAIVNSKKERLSIATFHSIKLGGEIGPIPCLVTPKTPALFRTVGVAEYYKHRYSRTLHGKSY
LDFMRIEGNHDS
>CMADIOX3
MAPVPISSIRVGQIDDVQELRKSRPKSIPERYVRDIAERPRLAKLQPSLPMQIPVIDLSMLDETNTYQSQ
KELSKLAASCRDWGFFQVINHGIDTDLLKTIEKVATEFFMLPLAEKQKYPMLPGTVQGYGHAFVFSEDQK
LDWCNMLALGVEPFFIRNPKLWPTNPAKFSETLETYSTNIRKLCKNLLRFIAMTLGLNQDTFNKMFGEAV
QAVRMNYYPPCPRPDLVLGLSPHSDGSALTVLQQGTGSSVGLQILKDDKWVPVQPLHNALIINIGDTIEV
LSNGIYKSVEHRAVTHKKKDRLSIVTFYAPSYEIEIGPLPELIGQNQPCMYRRFNHGEYSKHYINNKLEG
KKTLDFAKIQTKLSS
>CMADIOX4
MTMEPTILETKTEEKSISESTTYQKGVKHLCECGVSKVPNKYIFPVSERKGEEPKNSGSNLKLPVIDFDQ
LKGSNRCQVLNSLSKACEDFGFFQVINHGIPDDIIQNMIETSRGFFELPLEERSKYMSTDVRAPVRYGTS
FNQINDSVYCWRDFLKLACHSLPDSVPHWPSSPADLRDAASTYAEENKSLFKMIMEAILESLGLIRTTET
DDDNDVLKEFEDGSHLTVINCYPSCPQPELTLGMPPHSDYGFLTLLLQDEVEGLQIQHKGEWVTVEPIPN
AFVINIGDHLEIFSNGRYKSVLHRAIVNSTKCRTSIASLHSMPMMNMVKPSPKLINEENPRRYKDTNFAT
FIDFIASNDLKEKSFLDSRKLT
>CMADIOX5
MTPLMETPPKVISLGSSLPVPSVQELVKQPLERVPPRYVQQQDQDSSRLVISSDDSCLVLPTVPVIDLQN
LLSPESIIGDLELEKLDSACKEWGFFQVVNHGITNTLVEKVKLEVQDFFKLPVEEKQKYEQIRGEIEGFG
QAFVISKDQKLDWADLFFMVTHPLHLRKPHLFPKLPLPLRDTLESYTTELRNLTMTLLESMAKALRLETK
EMTDLFEDGRQSMRINYYPPCPQPELVIGIRPHSDATGLTILLQLNETEGLQIRKEGIWIPIKPLPNAFI
VNIGDMLEIVTNGVYRSVEHQVTVNKMKERLSIATLYSPGLEREISPLPSLITPQTPTLFKKIEVEKYLK
ELFNRELNGKSFLDVMRTEGGGKE
>CMADIOX6
MEFSLSGGTSVGVQTLAESGLDHVPSRYIQPPENRPVTSFSCRKTSDSGIPVIDLFNFEPKRSDDVLQEL
RRACRDWGAFQVRNHGVPKRLLEDMIKVGLSFFKESSVEEKCKYSCDPNSAASEGYGSRMLVKEDTVLDW
RDYFDHHTFPLSRRNPNRWSESPSNYRGVVEEYSDHIKVLALKLMSMISESLGLQSSCIEDIVGEVYQNI
TISYYPPCPQPELTLGLQAHSDIGAVTLLIQDDVGGLQVLKDDEWVTVQPLSDAIVVILADQTEIMTNGQ
YKSSQHRAITNSHRARLSVGTFHDPSKTRKIAPAAPLIRKDSPAKYKEVIYGDYVSSWYTKGPEGKRNID
ALLLHQ
>CMADIOX7
METPPKQQVTLGVSLPVPSVQELAKQPLSQVPPRYVRSDQDPPIIYESHHDVSSSTTTVQNVPIIDLKSL
LSQGPIGDSELERLHFACREWGFFQLVNHEVNTSLVEKVKTEIQDFFKLPLEEKKKYWQKEGDVEGFGQA
FVISEEQKLDWADLFFMITLPRDARKSHLFPELPLPLRETLESYSSEMKNLAMTILEVMSKALHIETKLM
TDLFEDGMQNMRMNYYPPCPQPELVIGLTPHSDSVGLTILLQLNEMEGLQIKREGKWVPIKPLPNAFIVN
IGDILEIVTNGIYNSVEHRAIVNSKKERLSIATFHSIKLGGEIGPIPCLVTPKTPALFRTVGVAEYYKHR
YSRTLHGKSYLDFMRIEGNNDS
>CMADIOX8

MEGSHLPAEETSSLEKRVQELAINCKEPPPRYICKTDNEEENQRPADDHLSYSVPIIDLSRFSSRSSITE
KEEEMEKLRSALNSWGLFQAIGHGIPKNLLDDVHNVTKQFFELPIEEKQKYTKADSEIVDLQGYGCDRIV
SEDQVLDWSDRLYLLVDPEEQRKLKLWPEPPSNFRNTLHEYSAKSSSVAELILKVMAESVGLEENNFLSQ
IGEQVLTYARFNYYPPCSRPDRVYGIKAHADGGALTILLQDKEVEGLQVLKDDRWINVPSIPYALLVNVA
DLMEIMSNGLFKSPVHRVVTNTERERISLAMFYSPAFDKEVEPAAGLIDESKPRMFRKVNVKNYLEVFFP
RYLQGKRAIDWAKV
>CMADIOX9
METPKPVTLGVSLPVPSVQELAKQPLSQVPPRYVRSDQDPPIIYESHHDVSSSTTTVQNVPIIDLKSLLS
QGPIGDSELERLHFACREWGFFQLVNHEVNTSLVEKVKTEIQDFFKLPLEEKKKYWQKEGDVEGFGQAFV
ISEEQKLDWADLFFMITLPRDARKSHLFPELPLSLRDTLESYSSEVKNLAMTILEVMSRALHIETKLMTD
LFEDGMQTMRMNYYPPCPQPELVIGLTPHSDSVGLTILLQLNEMEGLQIKKEGKWLPVKPLSNAFIVNIG
DILEILTNGIYNSVEHRAVVNSMKERLSIATFHNVKLDGAVGPIPCLVTPETPALFKTVGAAEYFKHLFS
RKLHGKSYLDSMRI
>CMADIOX10
MTAKLQRDSREREREERVRKEASMDQTTKSVRIGTCLLVPSVQELAKQHFVKVPPQYICPNQDPAPIFSQ
TLETHSLFVPTVPIIDLQRLLSQEPHGDLELGRLDSACKDWGFFQLVNHGINSSLIEQVKSEVQDFFRLP
MEEKKKFWQNEGDIEGFGQKFVVSKEQKLDWSDMFFMITLPLHSRKPHLLPKLPLPLRVTIESYSEELKR
LTMTLVELIAKSLQIEPKLMTELFEDGRQAMRMNYYPPCPQPELVMGIPPHSDSVGLTILLELNEIEGLQ
IKKEGMWVPIKPIPGALLVNIGDMLEIVTNGIYQSVEHRATVNSLKERLSLATFYSPGLEREISPAPSLI
TPQTPALFKSIGVGNYLTKTRFNKDQLHGKLLHSLRNVTG
>CTRDIOX1
MKVGSLPVPNVQDLASKKLKEVPDRYIQREPESEHVSFEMSDQVPVIDLERLLDDEPFIYSDEMAKFHLA
CKDWGFFQLINHGVDEELLKKMVSNVEEFFKLPLEEKKAYAQLPDDLEGYGQAFVVSEEQKLEWSDILLL
RTLPVSIRNLRFWPEKPSSFSETLDKYAFAMEKVTFLLLNLIAKNLGVEPKMVTSIFEDGIQGVSSNYYP
PCPAPEKVLGISPHSDASMITLLIQNNELPGLQIRKNGNWVPIKPIPGAFVVNIGDAIEIMSNGLYKSIE
HRAVIHPDKERISIAGFQDPRSGVLVGPLPELLKKKNVGANYKTVNFDEYLRQYISAKLDGKTMLDLVKL
EKPEIQEGA
>CTRDIOX2
MDQKAQVTVTLGSSLLVPNVQEMARKALNTIPERYIRDDLEPHFVTISDPTLLPSVPIIDMKALLEGELV
DSELEELHSACKDWGFFQLVNHSVSGSLVEKVKVGIQEFFNLPIEEKQKYWQKPGEVEGFGQAFVVSEEQ
KLDWGDLFFVLTQPAYLRKPHLFPLLPQPFRGILEEYSIELKNLANKVLEMMAKALKMEDEEMKLLFEDG
MQSMRMNYYPPCPQPEQVIGLTPHSDAVGLTILLQVNEMEGLQIRKDGMWVPVKPLPNAFIVNVGDVLEI
VTNGIYRSIEHRAVVNSSKERLSVATFYNPRIDGEMGPAPSLIKSDSLALFRRISVQDYFKGLFARELVG
KSYLDSMRI
>ECADIOX1
MEITQKEIKLGKSLLVPSVQEMVKQPIAEIPPRYLQPDQNTPNTSSDVSLPQLSVPIIDLESLLTPGSVG
DLELDRLDSACKEWGFFQLINHGVSSSLVEKVQLETQDLFRLPMIEKKKYWQTEEEVEGFGQAFVVSEEQ
KLDWADIFYLTTLPIHGRKPHLFPKLPLPFRDTLDSYSLELKSLAMTLIKVMAKALQIETKVIEELFEDG
LQSMRMNYYPPCPQPDLVIGLKSHSDSVALTILLQLNEVEGLQVIKDGKWVPIKPLSNAFIVNIGDILEI
LTNGIYRSVHHRAIVNSTKERLSIATFLNPNPEGEISPIGSLITPSTPALFKTVGVKQYLQDLFTRELDG
KSYLDCMKIDGNTT
>ECADIOX2
MDISQKEVKFRSLLVPSVQEMVEEPIGEIPSRYLQPGQNTPIITTDVSLSVPIIDLESLLTPGSIGDLEL
EKLDSACKEWGFFHLINHGVSSSMVERVQLETQHFFRLPVVEKKKYWQSEEEVEGFGQTFVVSEEQKLDW
ADVFYLTTLPVHARKPHLFPKLPLPFRDTLESYSLELKSLAMTLLRVMAKALQVETKELEELFDEDGFQS
MRMNYYPPCPRPDLVVGLRPHSDSTALTILLHVNEVEGLQVIKDGKWVPIKPLPNAFLVNIGDVLEILTN
GIYRSVEHRATVNSTKERLSTATFLNPKLEGEISPIKSLITPNNPALFKTIGVKQFYQDLVMRELNGKSH
LDFMKIESNNTA
>GFLDIOX1
METAKKPIIIGGSLLVPSVQELAKQPLVQVPTRYVRSDQDSPTLSDDISTIPTVPVIDLQCLLSPEAIGD

SELEKLHSACQEWGFFQLVNHGVSPSLVEKVKMEIKDFFRLPMEEKNKYWQEERQVEGFGQAFVVSKEQK
LDWADLFFMVTLPLHARKPHLFPKLPLPLRETLEFYSSELKILTMTLLKMMCKALQIGTKEMTELFEDGM
QSMRMNYYPPCPQPELVIGLTPHSDSTGLTILLQLNEMEGLQIRKDGTWVPVKPLPNAFIVNIGDALEIV
TNGIYRSVEHRAMVNSMKERLSVGTFHGPRLEDEIGPLPNLINPDRPAMFRRVRVEEYFKKLFTRALDGK
SFIDSMRIEMGCAEAKPA
>GFLDIOX2
METAKKPIIIGGSLLVPSVQELAKQPLVQVPTRYVRSDQDSPTLSDDISTIPTVPVIDLQCLLSPEAIGD
SELEKLHSACQEWGFFQLVNHGVSPSLVEKVKMEIKDFFRLPMEEKNKYWQEERQVEGFGQAFVVSKEQK
LDWADLFFMVTLPLHARKPHLFPKLPLPLRETLEFYSSELKILTMTLLKMMCKALQIGTKEMTELFEDGM
QSMRMNYYPPCPQPELVIGLTPHSDSSGLTILLQLNEMEGLQIRKDGRWVPVKPLPNAFIVNIGDILEIV
TNGIYRSVEHRATVNSMKERLSIATFHSPGLEDEIGPIANLITPDRPAMFRSVRAEEYFKKRFTRALDGK
SFIDSMRIEAGCGEGKPT
>GFLDIOX3
METTKMPSSYVPVPSVQELATQIDDLVQIPARYVRSDQESSTIFSDEISTNIPSVPVIDMQCLLSLETIN
GVSDELDKLHFACQEWGFFQLVNHGVSTALVEKVKSEIQDFFKLPMEEKKKYSQKDGGVEGFGQVCVVSE
EQKLDWADIFFMFTLPLHARHLFPELPLPLRETLESYSSELKSITMTLLRMMAKALQIETKDMTKLLEDG
VQSMRMNYYLPCPRPELVIGLTPHSDATGLTILLQLNEMEGLEIRKDGRWIPIKPLPNAFIVNIGDILEI
GTNGIYRSVEHRATVNSTKERLSIATFHNPSLQDEICPLPNLITSDRPAMFRSIMTEEYNTKLLTRALDG
KSLLDSLRIEAKCEDDGN
>GFLDIOX4
METAKKPIIIGGSLLVPSVQELAKQPLVQVPTRYVRSDQDSPTLSDDISTIPTVPVIDLQCLLSPEAIGD
SELEKLHSACQEWGFFQLVNHGVSPSLVEKVKMEIKDFFRLPMEEKNKYWQEERQVEGFGQAFVVSKEQK
LDWADLFFMVTLPLHARKPHLFPKLPLPLRETLEFYSSELKILTMTLLKMMCKALQIGTKEMTELFEDGM
QSMRMNYYPPCPQPELVIGLTPHSDSTGLTILLQLNEMEGLQIRKDGTWVPVKPLPNAFIVNIGDMLEIA
TNGIYRSVEHRATVNSLKERISIATFHNPNPEDEICPLSNLITSDRPAMFESIRAEDYTKKLLTRALDGK
SLLDSLED
>HCADIOX1
MEEPKLSYGSSILVPSVQELAKQALASPPPRYIKPHQDPPIISYSSLIPPIPVIDMQKLLYGEDMDSELE
TLHSASKEWGFFQLVNHDVNTILVEKVKHEIQEFFQLPLEEKKAYWQKEGEVEGFGQAFVVSNEQKLDWG
DMFFMLTLPIYARKPHLLPQLPLPFRDTMDTYSLESKNLAMKILEIMEKALKMEVNEMTNLFEDGMQSMR
MNYYPPCPQPELVIGLTPHSDTAALTILLQVNEVEGLQIRKEGMWIPIKPLPNAFIVNIGDMLEIITNGI
YRSIEHQATVNSEKERLSIATFYSPRHNVEMGPAQSLVTPQTPALFRRIGVEEYSKGLFSRVLDGKSYLD
VMRTENGKNNSN
>HCADIOX2
MEEPKLSYGSSILVPSVQELAKQALASPPPRYIKPHQDPPIISYSSLIPPIPVIDMQKLLYGEDMDSELE
TLHSASKEWGFFQLVNHDVDTILVEKVKHEIQEFFQLPLDEKKFFWQREGELEGFGQAFVVSDEQKLDWG
DMFFMFTLPIYARKPHLFPQLPLPFRDTMDTYSLELKNLAMKIIGMMEKALKMEVNEMTNLFEDGTQSMR
MNYYPPCPQPELVIGLTPHSDSVGLTILLQVNEMEGLQIRKEGMWIPIKPLPNAFIVNIGDILEMVTNGK
YCSIEHQATVNSVKERLSIATFYSPRHNVEIGPAQSLVTPQTPALFKRIGMEGYLKGLFTRQLDGKSYLD
VMRIENGKNNSN
>HCADIOX3
MEEPKLSYGSSILVPSVQELAKQALASPPPRYIKPHQDPPIVSNSTLIPTIPVIDMQKLLYGEDMDSELE
TLHSASKEWGFFQLVNHDVDTLLVEKVRHEIQEFFQLPLDEKKFFWQREGELEGFGQAFVVSNEQKLDWG
DIFFMLTLPIYARKPHLFPQLPLPFRDTMDAYSLESKNLAMKILEIMEKALKMEVNEMTNLFEDGTQSMR
MNYYPPCPQPELVIGLTPHSDTAALTILLQVNEVEGLQIRKEGMWIPIKPLPNAFIVNIGDMLEIITNGI
YRSIEHQATVNSEKERLSIATFYSPRHNVEMGPAQSLVTPQTPALFRRIGVEEYSKGLFSRVLDGKSYLD
VMRTENGKNNSN
>JDIDIOX1
METEMINTWEFGGSLPVENVQALAAKELKDIPDRYIRPELQSDHVSIDESREIPVIDLSRLLDEQVSLDE
LAKFHSACEGWGFFQLINHGVSEEINKMKIDVQDFFSLPLEEKKACSQVATSIQGYGQAFVVSDEQKLD

WADMLFILPLPVRERNMRFWPNTPTSFRETLDKYSLELQRVTVSLIGLMARNLAVEPEKLTNMFENGLQG
VRINYYPPCPQADKVLGLSPHSDAVALTLLIQVNDVEGLQIRKRGNWVPVKPIPGAFIVNIGDIMEIISN
GKYKSIEHRAIINPKKERLSIAAFHSPNLSTLIGPLADLVNESGEHYKTIMFEDYLKHIVSSKLDGKSTL
DYLKLD
>JDIDIOX2
MGTEVPMPMSVENVQDLATKILDVIPSRYIRPELMTDVVSIDEESMIPIIDLSKLTDDHVSQAELEEFHL
ACQNWGFFQLINHGLKEDVLEKLKMDVTEFFNLPIEAKKEFSQEPGQMEGYGQAFVFSDEQKLDWGDMLY
LVTQPEAARRMKLFPTCPSTFRDTIANYSLESERVKMCVLGVMAKNLGIEREKFTNTFEGGIQSMRFNYY
PPCLEEDKVLGISPHSDATGITLLVQVNQVHGLQIRHEGRWVPVVLATDAILVNIGDVLEILTNGKYKSI
EHRVVINKNKERLSVAAFHEVNENATVGPIPELVKDGEECYQSFSFDEYAELLIGEKLDGKSLIERMRLK
K
>MAQDIOX1
MAPTPISPLQVGHIIDVQELRKVRTKTIPRRYIRAVAERPISAATSTMHIPVIDLSKLIEGNSDQSQMEL
SKLASACEQWGFFQVINHGIDISMMENMEKVGKEFFMLPVEEKQKYPMAPGTIQGYGQAFVFSEDQKLDW
CNMLALGIEPHFIRNPKLWPTNPANFSETLETYSEEVRKLCQNLLRFIATSLSLRDETFNDMFGVPVQAV
RMNYYPPCQRPDLVLGLSPHSDGSALTVLQQAAGSSVGLQILKDDKWVPVQPVPNALVVNIGDTLEVLTN
GKYKSVEHRAVTHKDKDRLTIVTFYAPDYEIELGPLPEFVSENRPCMYRKYNHGEYSRHYVTNKLQGKKT
LDFARIQTEFSA
>MAQDIOX2
MEVGLAETLQYNVQLLASKKLDVIPSRYIRPELAADIICMDKASVIPVIDFSKLCDDTFSQDELEKFHLA
CQDWGFFQLVNHEVPKEVLEKVKTDLTEFFNLPIEEKMACQQPGQEEGYGLVVMFSDEQKLDWGDMIGIV
TQPETTRNVNVWPNSPLTFRETIAQYSLELQKIKTCILGLMAKNLGIEPEKFTNMFEGGMQSMRINYYPP
CQKDDKVLGLSPHSDSNGITFLIQVNDVQGLQIRHEGCWVPVVLSTDAILVNIGDVLEILTNGRYKSVEH
RVVINKNKERLSVAAFHELNENETIEPLQELIKEGEDKCYRSLSFAEYQKLESSHYLSHYLDGKTLIEKM
KLKK
>MCADIOX1
MSSNTGHFGGSLPVENVQALASQELKEVPSRYVRPELAFDVVSIDESLQIPVIDLFRLTDQRYARDELAK
LHSACEDWGFFQLINPGVPHDMIENMKADTEEFFKLPLEVKKAYSQLPNSIEGYGQAFVVSEEQKLDWGD
MLFFFCQPLNERNMRIWPNHPDSFRETLDKYSAELQRVALCLLGFLAKNLGLDPEKFINMFENGMQGVRM
NYYPPSPQAEKVLGISPHSDADSLTLLLQVNEVQGLQIRHNGIWVPIEPIPGALIVNIGDVIEIMSNGKY
KSIEHRSIVNTKKERLSIAAFHTTKAGTTIGPISDLVKENGANYRTVSDEDFLRQVITNKLDGKSVLDHM
KLK
>MCADIOX2
MSSLETMRESTVPLAVENVQVLASKNLKEIPLRYIRPEHELEISSINEITEASSSDVPVINLSRLTDDDE
QFYQEEMGKFHFACQEWGFFQLVNQGVAEDVMEKMIRDVSEFFKLPFEEKKACSQDADQMEGYGQSFVFS
EEQKLDWGDMLFLFTQPLLSRNFKESVSRYSSEMERIKTILVGQMGKNLGINQEEFTNMFNNGVQSMRFN
YYPPCPQASKVLGVSPHSDATGLTVLLQLNQVQGLQIRHDGQWVPVNILPDALLVNIGDILEILSNGRYK
SIEHRVVINKEKERMSVAVFHETNKSAETGPLPELLLNNEGETDKYRSVSFEGFVNLFSSRKLDGKSLVD
FMKLKK
>MCADIOX3
MADEKAKEAVPVTLGSSLLVPSVQEMARKAISTVPERYVRNDLDPPFSTNPTLLPTLPTIDMNSLLDGES
ADSELEKLHLACKDWGFFQLLNHGVSASLVEKVRIGIQDFFNMTIEEKQKFWQKPGEVEGFGQAFVVSEE
QKLDWGDLFFCTTQPAYQRKPHLFPMLPQPFRETLEEYSLELKNLAMTLIEMMAKALKMDIEDMKLLFED
GMQAMRMNYYPPCPQPDQVIGLTPHSDAVGLTILLQVNEMEGLQISKDGMWVPVKPLPNAFIVNIGDILE
IVTNGIYRSIEHRATVNASKERLSIATFYNPKFDGEMGPAPSLIKKDSPPFFIRMRVEEYFKGLFARELV
GKSYLDSMRIRYEKSGIV
>MCADIOX4
MTVGPLTVPNVQDLASNKLKDIPHRYIQREPESEHVLFEISDEVPLIDLQKILHESFSSDEMAKLHMACK
DWGFFQIINHGVDEELIKRMLNDVEEFFKLPLEEKKAYAQLPNSFQGYGQELVVSQEQKLDWSDMLVLHT
LPISRRNMKFWPINPASFRETLDKYTMETERVTLCLLSLMAKNLGVEAEKVTSIFEDGIQAVRMNYYPPC

SQADKVLGISPHSDASIITLLIQNNQVPGLQIRKDGNWVPIKPIPSAFIVNVGDVIEIMSNGLYRSVEHR
AVIDPEKERISIAGFQDPRNGVLVGPLPELMEGNKGANYKTVSYDEYLRQYLSAKLNGKSVLDLLKLEKC
EIKERV
>NDODIOX1
MGSEQMNDEQRVFGGSLPVDYVQVLAAKKLKEIPDRYIRSELEFEVVSIDLSNKIPVIDLGKLLDEQFRR
DEMAKLHSACEDWGFFQLINHGVSEEIIQKMKTNSHEFFNLPLEEKKVYANLPSSNEGYGQAFVVSEEQK
LDWVDMFFVTTLPLHERITRLWPNNPTSFRETLDQYSLELQRVTISLAGFMANNLGVEPEKLTNMLENGS
QGITMNYYPPCPHADKVIGLSPHSDACAITLLIQVNEVEGLQIRKNGNWVPIKPIAGAFIVNIGDTMEIL
SNGKYKSNVHRAIVHPQKERISVAAFHRPNLSAMIDPLSELVKESGKHYKTILFQDYLKHYFASKLDCKS
ALDLIKLDN
>NDODIOX2
MESKQVYKSRMLGGSLPVENVQALASKKLQEIPDRYIRPELEFDVVSVNLSNEVPVIDLRKLLDEQFGCN
EMAKLHSACEDWGFFQLINHEVSEEIIEKMKIDLQGFFSLPLEEKKAYGQLANSNEGYGQAFVISEEQKL
DWADRLYIVTLPVEERNMRFWPNTPTSFRETLDKYSVELQRVAVCLVGLLASNLGVEPKNLTNMFDKGFQ
AVRMSYYPPCPCADKVIGLSPHSDAVALTLLIQVNEVEGLQIRKNGNWVPIEPIPGAFIVNIGDAMEIIS
NGKYKSIEHRAVIHPQKERISVAAFHGPNLNAMIGPLSNLGKESGEHYXEVGGGQNMGKTVAIVVGGVAA
VGFALICLMFLRSLMKKHDDY
>NDODIOX3
MAPAAIPSLKVGHIHDVQELRKARRRTIPKRYVRDIAERPITSVTSTMHIPVIDLSKLIQGDIDQSQTEL
LKLASACKDWGFLQVINHGIDVSLLESIEKVTKEFFMLPLAEKQKYPMAPGTVQGYGQAFVFSEDQKLDW
CNMLALGIEPHFIRNPKLWPTKPAEFSETLEMYSTEIRKLCKNLLRFIAMSLGLKDDVFNGMFGTPVQAV
RMNYYPPCPRPDLVLGLSPHSDGSALTVLQQAAGSSVGLQIYKDDTWVPVQPVPNALVINIGDTLEVLTN
GIYKSVEHRAVTHRDKDRLTLVTFYAPSYEMELGPLPEFIDENRPCMYRKYNHGEYSRHYVTNKLEGKKT
LDFAKIQTKCRLKSPPRFNLTL
>NDODIOX4
MESANELGGSIPVENVQALAAKDLKEIPHRYIRPELESDVVSIDKSREIPVIDLGKLLDEQFAPEELAKL
HSACADWGFFQLINHGASEEIIEKMKHDIQEFFRQSLEVKNAFSQLPNSVEGYGQAFVVSEEQKLDWADM
LYLETLPISQRKVNLWPTTPTSFRETLDKYSLELQRLTICLIGLIAKNMEIEPQKMTNIFENGTQGVRMN
YYPPCPCANQVLGLSPHSDGSGLTLLIQVNEVAGLQIRKNRDWVPVKPIAGAFIVNIGDAVEIFSNGKYK
SIEHRAVINPEKERLSIAAFHDPNLSTMIGPLPELVKENGERYKTIKYKDYIKQVMASKLDGKSSLDSLK
LQK
>NDODIOX5
MESEMNKAIEVGGSIPVDNVQALATTELKEIPHRYIRPEFESDVVSNDKSGKIPVIDLCKLLDEQFSHDE
LAKLHSACEDWGFFQLINHGAAEEIIEKMKIDIQEFFRQPLEVKKAYSQLPNSMDGYGQAFVASEEQKLD
WADMLFLTCLPISQRYVTLWPTIPTSFRETLDKYSLELQRVTTCLIGMMAKNLGIEPQKLTNIFENGLQG
VRMNYYPPCPQANKVIGLSPHSDGSGLTLLIQVNEVEGLQIRKNGDWVPVKPIAGAFIVNIGDAVEILSN
GKYKSIEHRAVINPEKERLSIAAFHNPNLSTMVGPLPELVKENGERYKTMKYEDYVKQVIASKLDGKSSL
DAVKLEK
>NDODIOX6
MESETKEPKPLGGSLPVENVQALATKELKEIPHRYIRPEIESSVVSVDKSREIPVIDLGRLVSEQFSSDE
MAKLQSACEDWGFFQLSNHGVSEEIIEKMKIDLRDFFNLPLEEKKACSQLPNNTEGYGQAFVVSDEQKLD
WSDMLFIMTLPVPERNMRFWPKNPSSFRETMDKYSTELHRVTVCLTSLMAKNLGVEPEKLANMFESGSQS
VRMNYYPPCAHADKVIGLSPHSDAVGLTLLIQANEVEGLQIRKNGNWIPVKPIAGAFVVNIGDVLEIISN
GKYKSIEHRAVAHPEKERLSIAAFHGPNFNAMIGPLSDLVKESREHYKTIIHEDYVKQIFASKLDGKSTL
DFMKLDK
>NDODIOX7
MNKAKELGGSISVDNVQALATKELKEIPHRYIRPELESDVVSIDESREIPVIDLCKLLDEEFASDELATL
RSACEDWGFFQLVNHGASEEIIAKMKINIQEFFRQPLEVKKSFSQLPNSMEGYGQAFVVSEEQKLDWADM
LFLVSSPISERNFTFWPTNPTSFRETLSKYSLELQRVTVRLVRFMAKNLGVEPDMLANMFANDGSQTIRM
NYYPPCPQRDKVLGLSPHSDGSGLTLLIQVNEVEGLQIRKNGSWVPVKPVAGAFIVNIGDVIEIMSNGKY

KSIEHRAVIDPVKERLSIAAFHGPNLNTMTGPLPDLVKKNEQRYMTVNYEDYVEQLVSSKLDGKSALDSM
KLEK
>NSADIOX1
MATSNPVKYGSSKLVPCVQELAKQPMVTPPARYIRLDQEPSFVSALPLDAVPVISMQKLLYGDSMELELQ
RLHSACKEWGFFQLANHGVPEILVENIKKEIQEFFELPFEEKKKFWQSENEVEGFGQAFVVSDEQKLDWA
DIFFINTQPINSRKPHLFPNLPLPFRRTLDDYSLELKNLAMKILGWMEKALKIEGKEITELFKEGVQSMR
MNYYPPCPESDKVIGLTPHSDSIGLTILLQVNEMEGLQVRKEGKWIPIKPLPNAFIVNIGDILEIVTNGV
YHSIEHRATVNKVKERLSIATFYSPRFEAEMGPAKSLVTDQTPAMYRRIMVHEYFRQFFSRELGGRSFIE
DMKIENED
>NSADIOX2
MGNKTNMREFGGSLPVQNVQALASQPLNQIPPRYLRPELLSDTVVTATDSDESLKIPVINFRRLLLDQQQ
QADDDGSYNEMDKFHAACRDWGFFQLINHGVADEVIEKMKMGTEGFFRLPLEEKNVYSQLPNNIEGYGQA
FVVSEDQKLDWGDMLFFLSQPLHDRTLRFWPNTPSSYRETLDKYSAELQKLAVSLVEMMASNLGIIPEKL
TDMFGIDGSQGLRMNYYPPCPHADKVIGLSPHSDGTGITLLLQVNQVQGLQIRKNGNWLPITPIPGAFIV
NIGDIIEIMSNGAYKSIEHRAVINPEQERLSIAAFHGPRLGTPIGPLPCIVKEDEMKYKTVSHEDYLKQM
INQKLDGKSVLDLMKL
>PBRDIOX1
MFKNQPRNIFLVPDPPSIVFSNSPLFHSINQSMEVFVSGVQTLSETGVTHVPSQYIQPPQNRPHQPTITS
SSDSLNIPIIDLSNFNPQHSNDVRTELGRACSDWGAFQITNHGISNSLIKDIIDVGLTFFNECSITEKSK
YSCDPNSAASEGYGSRMLVNEDSVLDWRDYFDHHTFPLSRRNPNRWPDFPTNYRGVVEEYSNQIKELALK
LMSMISESLGLQSSCIEDIVGEVYQNLTISYYPPCPQPELTLGLQSHSDIGAITLLIQDDVGGLEVLKDD
KWVTVQPVSDAIVVILADQFEIMTNGKYRSAVHRAVTNSHRARLSVGTFHDPGKIRKIAPAAQLVDKDTL
ARYKEVVYGEYVSNWYSKGPEGKRNIDSLLLNQQ
>PBRDIOX2
METPKSIKLGGSLLVPSVQELAQQSFAEVPARYVRDDLEPLTDLSGVSMIDQTIPVIDLQKLQSPVPIIR
ELESEKLHSACKEWGFFQVVNHGVDILLVEKTKSEIKDFFNLPMDEKKKFWQEEGDIQGFGQAFVQSEDQ
KLDWADIFLMVTLPRHTRNPRLFPKLPLPLRNTMDSYSSKLSKLASTLIEMMGKALHMETSVLAELFEDG
RQTMRINYYPPCPQPKDVIGLTPHSDGGGLTILLQLNEVDGLQIRKEKIWIPIKPLPNAFVVNIGNILEI
MTNGIYRSVEHRATIHSTKERLSVAAFHNPKVGVEIGPIVSMITPESPALFRTIEYDDYGKKYFSRKLDG
KSSLDFMRIGEGDEENKAT
>PBRDIOX3
METPKLIKLGGSLLVPSVLELTKQSPAEVPARYIRNDLEPMTDLSSASLTDQTIPVIDLQNLLSPEPELE
LEKLHSGCKEWGFFQVMNHGVDILLVEKVKSEIQGFFNLPIDEKNKFWQEEGDLEGYGKAFVHSEDEKLD
WADMFFILTQPQYMRKPRVFPKLPLRLRETIESYSLELSKLGLTLLDLMGKALQIETGVMSELFEDGRQT
MRMNYYPPCPQPEHVIGLTPHSDGGALTILLQLNQVDGLQIRKEEIWVPIKPLPNAFVVNIGDILEIMSN
GVYRSVEHRATINSSKERLSVAIFQSPKHGTEIGPILSMITPEAPALFKTIPYEDYLRKFFSRKLGGKSF
VDSMRIGESDEDNNTA
>PBRDIOX4
METQKQENFGASLSVPNVQELAKQSPEQVPDRYIRSDQDSSTNISCPSMTDQIPVIDLQSLLSPDPIIGE
LELERLHSACKEWGFFQVVNHGVDNLLVEKVKSEIQGFFNLPMDEKKKFWQEEGDFEGFGQAFVFSEDQK
LDWGDVFFILTQPQHMRKPRLFPKLPLPFRKTIESYSLETNKLSMTLLELMEKALKIETGVMTELFEGGI
QRMRMTYYPPCPQPKHVIGLTPHSDPDALTILLQLNEVDGLQIRKEKIWVPIKPLSNAFVVNIGDILEIM
SNGIYRSVEHRATVNSTKERLSVATFHSPRKDTEIGPILITPETPALFRTSGFEDYFRKFFAHKLNGKSF
LSSIRIGETDEGNNAT
>PBRDIOX5
MEAPKLIMLGGSLFVPSVQELAKQSLAEVPVRYVRDDQDTLGNNINITPMSMIDQSIPVIDLEKLLSPEP
IVGELELERLHSACKEWGFFQVVNHGVDSLLVEKVKSEIEGFFKLPMDEKTKFWQEEGDIEGFGQVFVHS
QDQKLDWGDMFLMQTLPRHTRKPRLFPNLPLPLRQTIESYSSELSKLVLTLVDLMGKALQMESGVLTELF
ENGIQRMRMNYYPPCPQPEQVIGLTPHSDVGGLTILLQLNEVDGLQIKKDKVWVPIKPLANAFVVNVGDA
LEIMSNGIYRSVEHRATINSTKERLSIATFHNPRADREIGPIPSMISPETPALFKTTGYEEYFKKFFSRK

LEGKSFLDSLRIREGDEHCGRLDVKGPCN
>PBRDIOX6
MEIPNPIKIGSSLLVPSVQELAKQSFAEVPARYIRNDVDPLITKLSDVSLIDQTVPVIDLQKLLSPEPIV
GELELERLHSACKEWGFFQVVNHGVDNLLVEKVKSEIQGFFNLPMEEKKKFWQEEGDFEGFGQMFVQSEE
QKLDWGDMFFILTQPQHMRKPRLFSKLPLPLRETIESYSLELIKLGLTIIKLMEKALQIDAGVMAELFED
GIHTMRMNYYPPCPQPEHVIGLTPHSDGGGLTILLQLNEVDGLQIRRENIWVPIKPLPNAFVVNIGDILE
ILSNGIYRSVEHRSTVNATKERLSVATFQNPKQESVIGPNMITPERPALFRKIVYKDYMKKLFSRKLDGK
SFLDSLRIGEGDERP
>PBRDIOX7
MGEVDAAFVQALEHRPNPNNIVEAGGIPLIDLSPLNDFCHSNDENSAHDSARHTDLVTKIGRACKEWGFF
QVFNHGVSSDLQQGVEAAVKKFFDLSSEEKRKVKRDEVNPMGYYDTEHTKNVRDWKEVFDFTVKNPTLLP
ASHHLEDQELQELRNQWPDYPPELREVCEEYASALEKLSFELLKLIALSLGLPEKRLNGYFNDDHTSFIR
FNHYPPCPEPHLALGVGRHKDAGALTVLAQDDVGGLDVKRKSDGEWVRVKPIPGSYIVNVGDIIQVWCND
KYESAEHRVTVNSKRERFSIPFFFNPSHHVMVKPLEELVDYQNPSKYKEYNWGKFFKTRKISNFKKLDAE
NIQIYHFKRSLV
>PBRDIOX8
METLKTVKPGGSLFIPNGQELAKQSLEEVYVGNDQDTMLLIGQTIPVIDLQKLLSPEPITGDMELDKLHS
ACKEWGFFQVVNHGVDILLVEKVKSEVHDFFNIPMDEKKPFWQEEGDLEGFGQVFITSEDQQLDWGDMFF
MVTLPKHMRKPRLFLKLPLPLRETIESYSLKLSKLGVTLVELMGKALQMEDRIMSELFDDGRQTMRMNYY
PPCPQPEQVIGLTPHSDPGGLTILLELNEVNGLIRKENIWVPIIPLPNAFIVNIGDILEIMSNGIYHSVE
HRATINSTKERLSVAMFNSPKVDTEIGPIHSMITPETPALFRTIGYDEYLKIFFSRKLDGKSLLESMKI
>PBRDIOX9
MSYESLPPKVVVPSVHELIKEKITEIPNPYIRTSCVDHEDHHTDQSTADHNIPVIDVQGLLSGESVLADS
EFEKLHSACQDWGFFQVVNHGISSLVIANLKSEMRNLFELPLEERKKICQEPGDPMAEGFGQLFIVSDDQ
KRDWSDTFYLTALPTKLRKPQLFAGIPVLMRESLEAYSSGLKDVTMILLGKMAKAIKMDSDEMEELFNDC
LQRMGLNYYPPCPKSEQVIGLSPHSDAGALSIVLQLNETEGLQIRKDGKWVTVKPIPGALIVNLGDMIEI
LSNGAYPSIEHRVMVNPAIERLSIATFHATNPDAEFGPALSLIDPPHKPALFRRETVEKFYTNFFANKLN
GKTTNLDFMRIQNGDQSSRLISGYRP
>PBRDIOX10
MEAPKLIMLGGSLFVPSVQELAKQSLAEVPVRYVRDDQDTLGNNINITPMSMIDQSIPVIDLEKLLSPEP
IVGELELERLHSACKEWGFFQVVNHGVDSLLVEKVKSEIEGFFELPVDEKKKFWQEEGDIEGFGQIFVHS
EDQKLDWADMFYMLTLPPNMRKPRLFPNLPLPLRQTIDSYSSELSKLVLTLVDLMGKALQMESGVLTELF
ENGIQRMRMNYYPPCPQPEQVIGLTPHSDVGGLTILLQLNEVDGLQIKKDKIWVPIKPLRNAFVVNVGDA
LEIMSNGIYRSVEHRATINSTKERLSIATFHNPRADREIGPIPSMISPETPALFKTTGYEEYFKKFFSRK
LEGKSFLDSLRIGEGDEHCGRLXVKGXCN
>PBRDIOX11
METPKLMKLGGSLFVPSVQELAKQSLAEVPARYVRDDRDMVGNIINVTPMSMIDQSIPVIDLEKLLSPDL
IVGELELERLHSACKEWGFFQVVNHGVDSLLVEKVKSEIEGFFELPMDEKKKFWQEEGDAEGFAQFFVQS
EDQKLDYSGDMFFMLNLPQHMRKPRLFLKLPLPLRETIESYSLKLSKLGVTLVELMGKALQMEDRIMSEL
FDDGRQTMRMNYYPPCPQPEQVIGLTPHSDPGGLTILLELNEVNGLIRKENIWVPIIPLPNAFIVNIGDI
LEIMSNGIYHSVEHRATINSTKERLSVAMFNSPKVDTEIGPIHSMITPETPALFRTIGYDEYLKIFFSRK
LDGKSLLESMKI
>PBRDIOX12
MGRTVKDLTDTSDLISIPSEYVFPTNPEDLEEAEQQIPIIDYYLLTSGSPEQRSLIIENLRSACLEWGFF
MVINHGVPQSLINEMMNLCLGFFDLSPNEKSDYVGKHVLNPIRCGTSFNTSVEKIYFWRDYLKILVHPEF
HAPLKPHAFRDISYEYCKRSREVAKELLKAISESLGLEEDYIEKALETKSGLQILIVNLYPPCPQPELAM
GMPPHSDHGLLTLLAQNEIGGLQIKHKGKWIAANALPNSFMVNIGDHMEILSNGKYKSILHRVAVNTKAT
RVTVVTANGPSLDAVVTPAPDLVDCENYPPLYRGMKYRDYVELQQSNQLDGKSCLDRILV
>PBRDIOX13
METPKLRDFGSFLPVPSVQELAKQVLTEIPPRYIRTDLEALNKLSCASNTDQTVPIIDMQCLLSAEPEME

LEKLHSACKEWGFFRVVNHGVDNLESVKSEIESFLNLPVNAKNKYGQKQGDDQGFGSRFVLSEEQKLDWG
DFFYMVTRPLYLRKPHLFPELPLPLRETIESYSSEVSKLAMALFEMMGKALKIETGVMTEIFEGGMQAMR
MNYYPPCPRPDLVIGLNAHSDFGGLTILLQLNEVEGLEIRNKGEWVSVKPLANAFVVNVGDVMEILTNGI
YHSVEHRATINSSKERLSVATFHYPKLETGIGPLPCMITPKTPALFGRIERYELLLRKYYARKLNGKSTL
DCMRIGNGFEDDNTA
>PBRDIOX14
MSYESLPPKVVVPSVHELIKEKITEIPNPYIRTSCVDHEDHHTDQSTADHNIPVIDVQGLLSGESVLADS
EFEKLHSACQDWGFFQVVNHGISSLVIANLKSEMRNLFELPLEERKKICQEPGDPMAEGFGQLFIVSDDQ
KRDWSDTFYLTALPTKLRKPQLFAGIPVLMRESLEAYSSGLKDVTMILLGKMAKAIKMDSDEMEELFNDC
LQRMGLNYYPPCPKSEQVIGLSPHSDAGALSIVLQLNETEGLQIRKDGKWVTVKPIPGALIVNIGDMLEI
LSNGAYPSIEHRVVVNPTMERFSIATFHSTNPDAEIGPAHSLIDPPHKPALFRRETLKKYYQNFFAHKLN
GKTTNLDFMRIRNGDLSS
>PBRDIOX15
MSSSPMVVPLVRELVKENITEIPNRYIRPTSDLDHEYHHSDDDSTAAKYNIPVIDVEGGLLSGESVLADS
ELHKLHSACQHWGFFQVVNHGISSLVIANLKSEMRNLFELPLEERKKICQEPGDPMAEGFGQLFIVSDDQ
KRDWSDTFYLTALPTKLRKPQLFAGIPVLMRESLEAYSSGLKDVTMILLGKMAKAIKMDSDEMEELFNDC
LQRMGLNYYPPCPKSEQVIGLSPHSDAGALSIVLQLNETEGLQIRKDGKWVTVKPIPGALIVNIGDMLEI
LSNGAYPSIEHRVVVNPTMERFSIATFHSTNPDAEIGPAHSLIDPPHKPALFRRETLKKYYQNFFAHKLN
GKTTNLDFMRIRNGDLSS
>PBRDIOX16
MSSSPMVVPLVRELVKENITEIPNRYIRTTCLDHEEDHSHRDDYDSAAAKYDIPVIDVQGGLLSGESVLA
DSELEKLHSACQHWGFFQVVNHGISSLVIDKLKLEIHNLFELPLEEKKKIWQEPGDMEGFGQQFVVSNEQ
KLDWSDMFYITALPTKMRKPQLFSRIPLSLREALEAYSWGLKNLNMILLGKMATALKMDSDEMEDLFSDC
LQRMKMNYYPPCPKSQQVIGLSPHSDAGALSIVLQLNETEGLEIRKHGKWVPVKPIPGALVVNVGDMIEI
LSNGAYTSIEHRVMVNPTIERLSIATFHSINPDAEFGPACSLIDPPHKPALFRRETVKKYYKNFFANKLN
GKTTNLDFMRIQNGDQSSRLISG
>PBRDIOX17
MSSFGSSLIVPSVQELVKKEITEIPDRYIRTSSSNLSLDNEIHYSDSAAHEIPVINIGRLLSGEQTLADN
ELDKLHSACKDWGFFQVVDHGVSLSTIEKIKLETHNLFELPLEEKKKLWQTPGDHEGFGQLFVVSDEQKL
DWSDMFFITMLPTILRKPHLFAGMPSPLREALEDYALGLEKLTMTILEQMAKALKMDSNEMRELFSGCYQ
TMRLNYYPPCPKAEQVVGISPHSDADALTIVLQLNETEGLQIRKDGKWIPVKPIHNAFVVNIGDIMEIVS
NGVYRSIEHRAVVNSTKERLSVAAFHSANLDAEIGPAHSLISPPHKPALFRRESVETYFKNFFARKLDGK
SYLASMRIDKVHGEQSS
>PBRDIOX18
MEAPKLIMLGGSLFVPSVQELAKQSLAEVPARYVRDDQDTLGNNINITPMSMIDQSIPVIDLEKLLSPEP
IVGELELERLHSACKEWGFFQVVNHGVDSLLVEKVKSEIEGFFELPVDEKKKFWQEEGDIEGFGQIFVHS
EDQKLDWADMFYMLTLPPNMRKPRLFPNLPLPLRQTIDSYSSELSKLVLTLVDLMGKALQMESGVLTELF
ENGIQRMRMNYYPPCPQPEQVIGLTPHSEVGGLTILLQLNEVDGLQIRKEKIWVPIKPLSNAFIVNIGDI
LEIMSNGIYRSVEHRATVNSTKERLSVATFHSPRKDTEIGPILITPETPALFRTSGFEDYFRKFFAHKLN
GKSFLSSIRIGETDEGNNAT
>PBRDIOX19
MSMIDQSIPVIDLEKLLSPEPIVGELELERLHSACKEWGFFQVVNHGVDSLLVEKVKSEIEGFFELPVDE
KKKFWQEEGDIEGFGQIFVHSEDQKLDWADMFYMLTLPPNMRKPRLFPNLPLPLRQTIDSYSSELSKLVL
TLVDLMGKALQMESGVLTELFENGIQRMRMNYYPPCPQPEQVIGLTPHSDVGGLTILLQLNEVDGLQIRK
EKIWVPIKPLSNAFIVNIGDILEIMSNGIYHSVEHRATINSTKERLSVAMFNSPKVDTEIGPIHSMITPE
TPALFRTIGYDEYLKIFFSRKLDGKSLLESMKI
>PBRDIOX20
MMSAQDSWPEPITRVQAISESGSSVIPDRYVKPPSERPNTSTTSSTELLGLSIDTETSSNVDASDLNIPL
IDFQGLLDDDEDNHLVLPDTILDQVCMASKNWGFFQIVNHGVSPELMDQVRDVWRQFFHLPFEQKQVYAN
TPATYEGYGSRLGVEKGAILDWNDYFYLHYLPQSLQDPNKWPALPLSCREVIAEYCKQLVKLSKKIMKIL

SITLGLEEDYLQNAFEGGEEFGACMRVNFYPKCPQPDLALGLSSHSDPGGMTLLLPDDDVVGLQVRKDDT
WITVKSAPNSFIVNIGDQIQVLTNAIYKSVEHRVTVNSMNERLSLAFFYNPRGDLIIQPAKELVTLERPA
LYSPMTFNEYRRFIRTMGTRGKLQLESMKSPR
>PBRDIOX21
METPKLVKSSGSSLFLSTSVQELAKQSLPEVPARYIRTNLEPLSNVSGDSQSVPVIDLQKLLSSEPIIGE
LELDKLHSACKEWGFFQVVNHGVDNLVMEKIKTEIQGFFNLSLDEKQKFWKKEGDAEGFGQNFIESEDQK
LDWGDTFGMFTLPIHMRNPRLFPELPLPLRETIESYSLDVRKLALALIGLMEKALKIKTSAMSELFEDGG
QAMRMNYYPPCPQPEHVIGLTPHSDAGGLTILLQLNEVDGLQIKKDKIWVPIKPLPNAFVVNIGDILEIM
TNGIYRSVEHRATINSSKERLSVAAFHSPKGDTLIGPMVSLITPETPALFRTIGYQDYMKKFMSRKLDGK
SLVNSMRIGEGDEDK
>PSODIOX3
METPILIKLGNGLSIPSVQELAKLTLAEIPSRYTCTGESPLNNIGASVTDDETVPVIDLQNLLSPEPVVG
KLELDKLHSACKEWGFFQLVNHGVDALLMDNIKSEIKGFFNLPMNEKTKYGQQDGDFEGFGQPYIESEDQ
RLDWTEVFSMLSLPLHLRKPHLFPELPLPFRETLESYLSKMKKLSTVVFEMLEKSLQLVEIKGMTDLFED
GLQTMRMNYYPPCPRPELVLGLTSHSDFSGLTILLQLNEVEGLQIRKEERWISIKPLPDAFIVNVGDILE
IMTNGIYRSVEHRAVVNSTKERLSIATFHDSKLESEIGPISSLVTPETPALFKRGRYEDILKENLSRKLD
GKSFLDYMRM
>PSODIOX2
METAKLMKLGNGMSIPSVQELAKLTLAEIPSRYICTVENLQLPVGASVIDDHETVPVIDIENLISSEPVT
EKLELDRLHSACKEWGFFQVVNHGVDTSLVDNVKSDIQGFFNLSMNEKIKYGQKDGDVEGFGQAFVASED
QTLDWADIFMILTLPLHLRKPHLFSKLPLPLRETIESYSSEMKKLSMVLFEKMEKALQVQAVEIKEISEV
FKDMTQVMRMNYYPPCPQPELAIGLTPHSDFGGLTILLQLNEVEGLQIKNEGRWISVKPLPNAFVVNVGD
VLEIMTNGMYRSVDHRAVVNSTKERLSIATFHDPNLESEIGPISSLITPNTPALFRSGSTYGELVEEFHS
RKLDGKSFLDSMRM
>PSODIOX1
MEKAKLMKLGNGMEIPSVQELAKLTLAEIPSRYVCANENLLLPMGASVINDHETIPVIDIENLLSPEPII
GKLELDRLHFACKEWGFFQVVNHGVDASLVDSVKSEIQGFFNLSMDEKTKYEQEDGDVEGFGQGFIESED
QTLDWADIFMMFTLPLHLRKPHLFSKLPVPLRETIESYSSEMKKLSMVLFNKMEKALQVQAAEIKGMSEV
FIDGTQAMRMNYYPPCPQPNLAIGLTSHSDFGGLTILLQINEVEGLQIKREGTWISVKPLPNAFVVNVGD
ILEIMTNGIYHSVDHRAVVNSTNERLSIATFHDPSLESVIGPISSLITPETPALFKSGSTYGDLVEECKT
RKLDGKSFLDSMRI
>SCADIOX1
MEKTQPILGTSLIVPSVQELAKQPLVEVPPRYLRPDQDPPIISQSDDSLVPITTVPVIDLQCLLSPEPIG
DSELEKFHSACKEWGFFQLINHGVNSSLVEKVKSEIQDFFRLPIEEKNKYWQEEGEVEGFGQAFVVSTEQ
KLDWGDMFFMATLPLHLRKPHLFPKLPLPLRDTLESYSSELKKLTMALLVLMARALQIETKVTTELFEDG
MQSMRMNYYPPCPQPEHVIGLTPHSDSSGLTILLQLNEMEGLQIRKEGRWVPVKPLPNAFIVNIGDILEI
VTNGIYPSVEHRATVNSMKERLSVATFHSPNLTKETGPAPSLITPYRPALFRRVGNEEFWRERFRRELNG
KSFLDFMKIGGGDGNTA
>SCADIOX2
MNSSSDSSVPKQQQQEEEVVKFGRTLLVPSVQELAKEQNTEIPHRYIRSGLDDEDCPANIVCDPTSSSFP
TVPVIDIQTLLSGDISADSELETLHSACKDWGFFQVVNHGVSSSLIEKIKLEIHDLFELPFEEKRKLWQQ
PDDLEGFGQMFVVSEEQKLDWSDMFFIFTRPTTIRKSDLYTKLPLSLRETLEAYSLELKELAITILGRMA
EALKMDSTEMRGLFNNGFQTMRLNYYPPCSQADKVLGFTPHSDADALTILLQLNETEGLQIRKDGKWIPV
KPIPNAFVVNVGDIMEIVSNGVYRSIEHRAVVNSTKERLSIATFFSTNLDAELGPAHSLIDPPHKPALFK
RVTVVKYYEDFFSQKLDKKSYIDFMRIENGQDNHSN
>SDIDIOX1
MEALPKPPVLGNSMIVPSVQELASKSLVKVPPQYIQMLDHQDPPFIINEVLTVTSALPIIDLQTLLSHPE
TNNNNFDSELVKLHSACKKWGFFQLVNHGVNTSLLDKVKSEIESFFKLPMEEKKKYWQDEENFEGFGQAF
VFSEEQKLDWADMFYMITLPRQARKHHLFPKLPGALRDALESYTSELQNIAMKLVEFIGRALEIETKVMN
EFFKDGLQTMRMNYYPPCPQPELVMGLASHSDAIGISILFQLNEIEGLQIKNEGIWIPVKPLPNTFVVNL

GDILEVTAYSNKWALS
>SDIDIOX2
MEALPKPPVLGNSMIVPSVQELASKSLVKVPPQYIQMLDHQDPPFIINEVLTVTSALPIIDLQTLLSHPE
TNNNNFDSELVKLHSACKKWGFFQLVNHGVNTSLLDKVKSEIESFFKLPMEEKKKYWQDEENFEGFGQAF
VFSEEQKLDWADMFYMITLPRQARKHHLFPKLPGALRDALESYTSELQNIAMKLVEFIGRALEIETKVMN
EFFKDGLQTMRMNYYPPCPQPELVMGLASHSDAIGISILFQLNEIEGLQIKNEGIWIPVKPLPNTFVVNL
GDILEILTNGLYRSVEHRVTVNSMKERLSVATFYSPKLEEVIGPLPCLINPHNPALFKTVGVEEYFKKRY
SLKLNGKLTIDFMRIEGDTA
>TCODIOX1
MDKAEGRAPVTLGKSLLVPSVQELARKPLATVPQRYVREDHQDDPSLLKHTCAPTSPLPSLPVIDLKCLL
DSQSAGSELEKLHFACKDWGFFQLVNHGVSTWLVEKVKSQTQDFFNLSIEEKQKFWQQPGEIEGFGQAFV
VSEEQKLDWADMFFMTTLPKYLRKPHLFPMLPLHFRETMEEYSLELKNLAMTILEMMAKALKIETEELKL
LFDDGLQAMRMNYYPPCPQPEQVIGLTPHSDAVGLTILLQVNEMEGLQIRKDGMWVPVNPLPNAFVVNIG
DILEVTTTYIYLYIYNIIII
>TCODIOX2
MTSKAGEHGSLPVPNVQELASNDLKGVPSRYIRPELETHQVLSDESLEIPVINLQKLLNQQQMGTSDADG
DDDNEMCRLHKACRDWGFFQLINHGVPEEIIEKMKSDTVEFFKLPLEEKKAYAQEPGNIFGYGQTFVVSE
DQKLDWSDILVLLTLPVSDRIMKFWPTKPSSFRGTLDKYAKEMEKLTICLLDLIEKNLGIESGKLRNVYK
GGLQRVRMNYYPPCPVADKVLGLSPHSDASLITLLTQVNEVEGLQIKKNGHWVSIKAIPGAFIVNLGDLL
EILSNGMYKSIEHRGVIHPEKERLSIAGFQGAKSGVVIGPFPELLGENGANYRAIEHDAYLKQFFSSKLD
GKRVLDLMKL
>TCODIOX3
MKSLSLPVPNVQELASKSVKDEVPDRYIRRELESEHVSDESSDEIPVIDLQKLFSSDDEMTKLHIACKDW
GFFQLINHGVEELIKKMVKNVEEFFHLPLEEKKAYAQQPGDSQGYGQAFVLSDDQKLDWSDMLSLTTLPI
SERNLRVWPKNPPSFSETLEKYVTEMQQLTFKLLSLIAKNLGVEEKKVTSVFDEGLQGVRSNYYPPCPRA
DKVIGISPHSDASIITLLIQNNEVPGLQIRKCGNWVPVKPIQGAFVVNVGDVLEILSNGLYRSIEHRAII
HPEKERISIAGFQDPNLGTVIGPIADLLDDKNFANYKTIRFDDYWGHYFSAKLDGKSVLNLMKLENNHA
>TCODIOX4
MTSKAGEHGSLPVPNVQELASNDLKGVPSRYIRPELETHQVLSDESLEIPVINLQKLLNQQQMGTSDADG
DDDNEMCRLHKACRDWGFFQLINHGVPEEIIKKMKDDTVEFFKLPLEEKQAFAQEPESFHGYGQAFVVSE
EQKLDWSDMLVILTLPVSDRVMKFWPTNPPFRGIIEKYAEEMEKLTICLLGLIETNLGIEGGKLINTYKD
GGLQWMRMNFYPPCPEADKVIGISPHSDASLITLLIQVNEVEGLQINKSGKWVPIKAIPGAFIVNFGDAI
EVLSNGMYKSIEHRAVIHPEKERLSIAGFQDPKRGIVVGPFPELLEDGAKYKTVEYDEYLTQYFSRKLDG
KSALDLIKLSEM
>TCODIOX5
MEGKVTGKIGEYRSLPVPNVQEFAKNNLKEIPSRYIRPELETHQVSNIDESLEVPVINLLKLLDQQLLGT
NDDDDEMGRFHKACRDWGFFQLINHGVPEEIIEKMKSDTVEFFKLPLEEKKAYAQEPGNIFGYGQTFVVS
EDQKLDWSDILVLLTLPVSDRIMKFWPTKPSSFRGTLDKYAKEMEKLTICLLDLIEKNLGIESGKLRNVY
KGGLQRVRMNYYPPCPVADKVLGLSPHSDASLITLLTQVNEVEGLQIKKNGHWVSIKAIPGAFIVNLGDL
LEILSNGMYKSIEHRGVIHPEKERLSIAGFQGAKSGVVIGPFPELLGENGANYRAIEHDAYLKQFFSSKL
DGKRVLDLMKL
>TFLDIOX1
MEGSKQVSFASKLVPNVQELAKQPIDSIPPRYVRTDLDNPTIVSVDPASTPTIPVIDMEKLLDGDNMDSE
TDKLHTACKDWGFFQLVNHGVDTTLVEKAKHEIQEFFGLPIEEKRKFWQKENEVEGFGQVHVVSDEQKLD
WGDMFFMYALPINARKPHLFPKLPLPFRDIIDAYSQEMKLLAMKILGMIEKVLKMDAKEITQLFEDGRQS
MRMNYYPPCPQPEKVIGLTPHSDAGGLTILLQINEIEGLQVRKNGIWIPIKPLPNAFIVNIGDILEIGTN
GIYRSVEHRATVNSTKERLSIATFHNPSLQDEICPLPNLITSDRPAMFRSIMTEEYNTKLLTRALDGKSL
LDSLRIEAKCEDDGN
>TFLDIOX2
MEGSKQVSFASKLVPNVQELAKQPIDSIPPRYVRTDLDNPTIVSVDPASTPTIPVIDMEKLLDGDNMDSE

TDKLHTACKDWGFFQLVNHGVDTTLVEKAKHEIQEFFGLPIEEKRKFWQKENEVEGFGQVHVVSDEQKLD
WGDMFFMYALPINARKPHLFPKLPLPFRDIIDAYSQEMKLLAMKILGMIEKVLKMDAKEITQLFEDGRQS
MRMNYYPPCPQPEKVIGLTPHSDAGGLTILLQINEIEGLQVRKNGIWIPIKPLPNAFIVNIGDILEIVTN
GICRSIEHRATVNKLKERISVATFYSPKFDREMGPAHSLVTEKNPALFRRIVVEDYMKGFFARKLVGKSN
LDVMRIDN
>TFLDIOX3
MSTTVMQAEWPEPIVRVQSLAESGLSVIPERYVKPPSQRPKLDLVVDHEINIPIIDLSGLEGDDDQLRKT
ILNQISVACRDWGFFQVVNHGVKPKLMHEAREAWRDFFHLPVEEKQIYANSPKTYEGYGSRLGVEKDAIL
DWCDYFFLHYRPQLLKDHNKWPAFPVSLRNLIEEYNSELVKLSGRLMKILSVNLGLDEEYLQNAFGGENI
GVCMRVNFYPKCPQPDLALGLSSHSDPGGMTLLLPDHSVSGLQVRKGDKWVTIQPAPDAFIINIGDQIQV
LSNAIYKSIEHRAIVNSVKERISIAFFYNPKSDLLLEPASELVTQDQPALYLPMTFDQYRLEIRTRGPRG
KAYMESLKSPR
>XSIDIOX1
MTKNLNNLGGSLPIDNVQSLAGTELKDVPERYVRPELEYDNDVVSTDKEDVEIPVIDLSRLLNQKFASDE
LAKFHSACLDWGFFQLINHGVSEEVIEKMKVDMEDFFRLPLEEKNVYGQLPNSTEGYGQSFVKSDEQKLE
WADMHLLITKPVQERNMRFWPNTPTSFRATVDKFSTDVQKVAMCLIGMMAKNLGLESQVLTKPFENCIQC
VRMNYYPPCPNFDKVLGLTPHSDASSLTLLLQVNQVNGLQIKKHGKWFPIKPILGAFVVNIGDILEIMTN
GIYKSIEHRVVINPDKERLSIAIFHDPEFAAIIGPVQDLVRKYGARYKSISHADYLNQSINNKLDSKTFL
GKFKLINNERE
>XSIDIOX2
MEAREVVSFGSSLIVPSVQELAKEQIDLISPRYIRSDQQDHPIFLDPSLLSTVPVIDMKILLAHDSAESI
HSELEKLHYACKHWGFFQVVNHGIDSILIEKMKSEIQDFFKLPYDEKKKLWQQPGNHEGFGQLFVVSEEQ
QLDWSDMFYITTLPNSLKQPSLFAKIPLPLRETLEAYSLELKTLARTILGKMAEALEMDTEEMKELFSDG
VQSIRFNYYPLCAQPEQVLGFTPHSDADALTILLEVNETEGLQIRKDGKWIPVKQVPNALVVNVGDIMEI
VSNGVYRSIEHRAIVNSVKERLSVATFYSTNVDAEIRPAPSLITAKNPASFRTVPVEKYFKELYARKLDG
KSYLDFMRIENC

FADOX

>CSATHCAS
MNCSAFSFWFVCKIIFFFLSFHIQISIANPRENFLKCFSKHIPNNVANPKLVYTQHDQLYMSILNSTIQN
LRFISDTTPKPLVIVTPSNNSHIQATILCSKKVGLQIRTRSGGHDAEGMSYISQVPFVVVDLRNMHSIKI
DVHSQTAWVEAGATLGEVYYWINEKNENLSFPGGYCPTVGVGGHFSGGGYGALMRNYGLAADNIIDAHLV
NVDGKVLDRKSMGEDLFWAIRGGGGENFGIIAAWKIKLVAVPSKSTIFSVKKNMEIHGLVKLFNKWQNIA
YKYDKDLVLMTHFITKNITDNHGKNKTTVHGYFSSIFHGGVDSLVDLMNKSFPELGIKKTDCKEFSWIDT
TIFYSGVVNFNTANFKKEILLDRSAGKKTAFSIKLDYVKKPIPETAMVKILEKLYEEDVGAGMYVLYPYG
GIMEEISESAIPFPHRAGIMYELWYTASWEKQEDNEKHINWVRSVYNFTTPYVSQNPRLAYLNYRDLDLG
KTNHASPNNYTQARIWGEKYFGKNFNRLVKVKTKVDPNNFFRNEQSIPPLPPHHH
>PSOFADX5
MMMSSSNILPLVTFLVLVFFSSGSWAANNSLNGDFLQCIKKNEYSSIPIPIFTPDNSSFTTIFRSSARNL
RFLTPNSTQTPQFIITPTHESHVQSAVVCSQKHGFDLKVRSGGHDVEGLSYVSDTPYVLVDLINFRNIIV
DLKEKTAWIQAGASLGEVYYQAANKSNNTLGFPAGFCPTVGVAGHISGGGFGALVRKYGLASDQVIDARI
VTVDGKIYTKETMGKDLYWAIRGGGANNFGVLLSWKVKLVPVTPVVTVATISRTLEQGATDLVHKWQFVA
DRLHEDVYIGLTFSVANSSRAGGKTVSVQFAFLFLGGSDRLLELMEESFPELGLKRNETTEMKWVESHVY
FYARGRPIELLWDRDHATKSFLKIKADYVREPISKSGLEAIWRRFVGGDSPAMLWTPFGGRMNEISEFET
PYPHRAGNIYNIMYVGNWMNETESEKQIDWMRRFYNSMARYVSKNPRSAYINYKDLDLGVNRNNVSEAVG
YVQARSWGRKYFKSNFERLVKVKSMVDPGNFFKNKQSIPPVSTWGKQ
>CJASTOX
MMGISSLVVFPFLVFIAIANSLVPLASSSSEYEGFLQCLDLRSNSSIPIYNPSSTSYTSILHASIYNLRF
ISPTTPKPNFIITPMRESHVQATVVCSREHGLLIRTRSGGHDFEGSSFVATVPFVLLDLIHLRTISVNIE

DETAWVQTGATIGELYYRIAEKSRTHAFPAGLCPSVGVGGHISGAGYGILMRKYGLSADHVIDARLVDVN
GRILDKESMGEDYFWAIRGGGGASFGVILAWKIRLVPVPPTVTTFVVPRVLEQGATALVHKWQIIADKLD
DDLFLGLSVQALHLDPDHPEKKTIVISFLGFYLGAPEKTLQLMEESFPELGLMKEDCIEMSWIKSALYFG
IFQLETDLSLLLERKNPIPPKNRYMSKSDYVHEPVSEAVLEGIWKRFNEVDEPEIIMSPYGGKMNEIEES
AIAFPHRKGNMYKINYLVSWKELGEEAEKKHASWIRELYNYMTPYVSKNPRCSYLNFKDNDLGHKKNGTA
TYLEAKVWGSKYYKNNFDRLVSVKTKVDPDDFFTNEQSIPPLRSLAEAKK
>BWISTOX
MSKMASSIFATFSLLSSLLPTSLASSDANYEDFLQCLDLYSQNSIPVYTRNTSSYTSILESTIKNLVFLS
PTTPKPNFIVTPMQESHVQTSVICCRMHGLQMRIRSGGHDFEGLSYVSNVPFVVLDLIHLKTINVDIEEN
SAWVQTGATIGELYYRIAEKVGVHAFPAGLCPTVGVGGHISGAGYGVLMRKYGVSADHVIDARIVNVDGE
ILDRESMGEDLFWAIRGGGGASFGVILAWKIRLVPVPPTVTIFIVPKTLEEGATALLHKWQFIGDNVHED
LFIGLSMRSVIISPKGDKTILVSFIGLFLGGSDKLVQHMEQSFPELGVKPHDCIEMSWIKSTVVFGVFSN
DASLSVLLDRKNPFPPKSYHKVKSDYVTEPLPISVLEGICHRFLKNGVNKAEIIMSPYGGRMNEISESEI
AFPHRKGNLYKINYIAEWEEAGSMENHLSWIRELYRYMTPYVSKSPRSSYLNFKDIDLGQTKNGTATYSQ
AKAWGSKYFKNNFKRLMQVKTKVDPNNFFCNEQGIPPFSS
>AMEFADX1
MKKMIPNSSSSSILSVLVLLLFSTSSWAANSIHEDFLNCLSIYKSSFPIPIYTSKNSSFNTLFRSSARNL
RFLSPNSTQKPEFIITPTLESHVQTTVVCSKKHGLDLKVRSGGHDVEGLSYVSDSPYVMIDLVDFRNITV
NVKNATAWIQAGSSLGEVYYKVGNESKNTLGFPAGFCPTVGVGGHISGGGFGSLVRKYGLASDQVIDARI
VTVNGEILNKKTMGKDLYWAIRGGGANNFGVLLSWKVKLVPVTPIVTVATIDRTLEQGATNLVHKWQFVA
DRLHEDVYIGLTMVTANTSRAGEKTVVAQFSFLFLGNTDRLLQIMEESFPELGLKRNDTTEMSWVESHVY
FYRRGQPIEFLWDRDHLTKSFLKVKSDYVREPISKLGLEGIWKRYVGGDSPAMLWTPFGGRMNQISEFES
PYPHRAGNIYNIMYVGNWLNENESEKQLNWMRSFYSYMGRYVSKNPRSAYLNYKDLDLGVNDNNVSEYIR
YLKARSWGRKYFKNNFEKLVKVKSMVDPDNFFKNRQSIPPIRSWGKELEAINIVI
>CMAFADX1
MMRISSSVTLSFLSFFVLLFSTSSLAATSNSDHGDFLQCLALHKPSTPIPIYTPSNSNFTTLFRSSARNL
RFLSPNSTQPQFIITPTHESHVQAAVVCCRKHGLDLKVRSGGHDVEGLSYWSDAPFVIVDLVNFRNITVD
VKAKTAWVQAGASLGEVYYKAANASNTLGFPAGFCPTVGVGGHISGGGIGALVRKYGLASDQVIDARIVT
VDGKILDKKTMGKDLFWAIRGGGASNFGVILSWKVKVVDVTPIVTVGTVSRTLEQGATDLVYRWQFIADR
LHEDLYLASTMTVANGSRAGGKTVLVNFFFVFLGDTDRLLRLMEESFPELGLNRSDCNEMSWVESHVFLY
APGRPVEYLWDRDHETKSFLKIKTDYVREPISKAALEGIWIRFMGGQSPAMLWTPMGARMNEISESELPY
PHRAGNIYNIMYVGNWQQESESEVMLDWMRRFYRHMGRYVSKNPRSGYLNYKDLDLGQSESGDSEAMSYI
RARSWGRKYFKGNFERLVKVKSMVDPGNFFKNKQSIPPIKSWGKN
>CCHFADX1
MGISRIRVVVVVSSLLSILVLSNISLATSAASVNENFLECLSLQKTPIPIPTYSPNNFSYSTIFNSSARN
LRFLSPTSEKPRFVITPLHESHVQAAVVCCKKHHLELKVRSGGHDLEGLSYTSETPFVIADLVNYRNISI
NVEDKSAWIQAGAQLGEVYYRIAEKSKTLGFPAGFCPTVGVGGHISGGGFGGLVRKYGLASDQVIDARMV
DANGNILDKESMGEDLFWAIRGGGASNFGVILSWKVKLVPVTPNVTVAIVDKTLEQGATSLVYKWQQIAD
KLHEDLYISPTMSTVNASRGGQRTVLVQFSILFLGGIDRLLQLTQQSFPELGLQRKDCTEMSWVESHVYF
YARGRPLELLLDRDHVTKSFLKVKTDYVKEPISITGLEGIWRKFLEGDAPVMLWTPFGARMNEISESESP
YPHRAGNMFNIMYIANWQDDRESQKHIDWIRRFYSYMGSYVSKFPRAAYLNYKDLDLGEHRYGPLINSLT
ARNWGNKYFKSNFERLVYVKSKVDPSNFFKNKQSIPTF
>ECAFADX1
MKMGISSSPSSSSTLSLLSLLLVFFISLTSSWATHSNSLHGDFIQCVLSQHLYSSIKVPIYTPQSSNFTS
LFRSSARNLRFLSPNLTKPQFIITPTHESHIQAAIICCKKHGLDLKVRSGGHDVEGLSYVSDTPFVIVDL
VDFRNITVDVKSKTAWVQAGASLGEVYYAVANKSKTLGFPAGFCPTVGVGGHISGGGFGALVRKYGLASD
QVIDARFINAEGKILDKRTMGDDLFWAIRGGGAANFGIIVSWKVKLVDVTPIVTVATVNRPLEQGATNLV
HRWQFIADRLHEDVYIGLTLIVANGSRAGGKTVIAQFSFMFLGRTDRLLSLMEESFPELDLKRTDCNEMS
WVQSHVYFYAPNRTIEFLWDRDHVTKSYLKIKADYVREPISKAGLEGIWKIFMTGDSPAMLWTPMGARMN
EISEFETPYPHRAGNIYNIMYVGNSAQATESENQVNWMRRFYQYMTPYVSKNPRSAYLNYKDLELGQNRK

NDSEAMAYIRARNWGRKYFKGNFERLVKVKSMVDPSNFFKNKQSIPPLHSWGKH
>GFLFADX1
MGISSFATLSFLSFLVLFSIGSSAANSNSVHGDFLQCLSSLNQTSTPITIYTPSNSNFTELFRSSARNLR
FLSPNSTQPQFIITPTHEYHVQAAVLCSRKHGLDLKVRSGGHDVEGLSYWSDVPFIIVDLVNYRNITVDV
KAKTAWVQAGASLGEVYYQVANASKTLGFPAGFCPTVGVGGHISGGGFGALVRKYGLASDQVIDARIVTV
DGRILDKRNMGKDLFWAIRGGGASNFGVILSWKVKLVEVTPIVTVATVGRTLEQGATDLVHKWQFIADRL
HEDIYIGLTMTVANGSRAGEKTVLAQFSFMFLGGTDRLLRLMHESFPELGLNRSDCSEMSWVESHVYFYA
PGRPVEYLWDRDHVTKSFLKIKADYVREPISKSGLEGIWRRFMGGQSPAMLWTPMGARMNEISEFELPYP
HRAGNIYNIMYVGNWVEENESEKQIDWMRRFYNYMGRYVSKNPRSAYLNYKDLDLGQNENSDSEAMRYIK
ARTWGRKYFKGNFERLVKVKSMVDPGNFFKNKQSIPPIKSWGIEAGD
>SACFADX1
MGISSSATLSFISFLVLLFSSTASLAANSTSVHGDFLQCLSIHNSSVPIPIYTPNNSNFTTLFRSSARNL
RFLSPNSTQPQFIITPTRESHVQAAVVCCRTHGLDLKVRSGGHDVEGQSYWSDVPFVIVDLVDYKNITVD
VESKTAWVQAGASLGQVYYEVANKSKTLGFPAGFCPTVGVGGHISGGGFGALVRKYGLASDQVIDARIVT
VDGKILDKETMGKDLFWAIRGGGASSFGVILSWKVKLVDVTPIVTVATVGRTIEQGATDLVHRWQFIADR
LHEDVYIGLTMTVANGSRAGEKTVLAQFSFMFLGGTDRLLGLMGESFPELGLNRSDCKEMSWVESHIYFY
APGRPIELLWDRDHLTKSFLKIKADYVREPISKDGLERIWIRFMGGQSPAMLWTPMGARMNEISESELPY
PHRAGNIYNIMYVGNWLQESESEQQIDWMRRFYNSMGRYVSKNPRSAYLNYKDLDLGQNENGASEAMRYI
KARTWGRKYFKSNFERLVKVKSMVDPDNFFKNKQSIPPIKSWGKN
>SDIFADX1
MGISSSATLSFVSFLVLIFSTSSLAANSSVHDDFLQCLSLHKPSTPTPIYTPSNSNFTTLFRSSARNLRF
LSPNSTQPQFIITPTHESHVQAAVVCCRKHGLDLKVRSGGHDVEGLSYWSDAPFVIVDLVNYRNITVDVK
AKTAWVQAGASLGEVYYKVANASNTLGFPAGFCPTVGVGGHISGGGIGALVRKYGLSSDQVIDARIVTVD
GKILDKKTMGKDLFWAIRGGGASNFGVILSWKVKVVDVTPIVTVGTVSRTLEQGATDLVYRWQFIADRLH
EDLYLASTITVTNGSRAGGKTVLVNFFFMFLGDTDRLLRLMEESFPELGLNRSDCNEMSWVESHVYFYAP
GRPVEFLWDKDHETKSFLKIKTDYVREPISKAALEGIWERFMGGQSPAMLWTPMGARMNEISESELPYPH
RAGNIYNIMYVGNWQQESESEQQIDWMRRFYRHMGRYVSKNPRSGYLNYKDLDLGQSESGDSEAMSYIRA
RSWGKKYFKGNFERLVKVKSMVDPSNFFKNKQSIPPIKSWGKN
>TFLFADX1
MKISKFQLLSIIMFMTTCSISLLDLASSSPNYESFLQCFDTYSNFSIPIHSPTSSSYTSILHASIYNLKF
KSPTIPKPKFIITPMHESHVQVTVVCCRQHGLQIRTRSGGHDFEGLSYVSDVPFVLLDLIHLRKISVNIE
DNTAWVQTGATLGELYYRIAEKSRTHAFPAGLCPSVGVGGHISGAGYGILMRKYGLSADHVVDARLVDVN
GRILDKESMGEDLFWAIRGGGASSFGVVLSWKIKLVPVPQTVTFFIVPRTLEQGGSALVHKWQFIGDKLT
EDLFLGLSIQAMHLDGDHNGKKTILISFLGHYLGPMEKLLQLMEENFPEMQLKKEDCIEMSWIKSAVYFG
VFTLESNLNILLERKNPFPPKGRYLTKSDYVQEPMSENALEGIWKRFHEIDEPEIIMSPYGGRMNEIEES
AIAFPHRKGNMYKINYIALWKEEGDEAERKHASWIRELYSYMTPYVSKSPRCAYLNFKDIDLGHKRNGTA
TYLEAKVWGNKYFKNNFDKLVNVKTRIDPDDFFTNDQGIPPLPSLAEDK
>HCAFADX1
METSSFLMISFLVFTTFCVPSASIHHENFLQCLDLHSNSSIPIYTPNSPSYLSILHASIYNLKFKSPSTP
KPNFIITPMQESHVQAAVVCCKKYNLQLRTRSGGHDFEGLSYVSYVPFVLLDLIHLRSINVNIEDNSAWV
QTGATLGELYYRIAEKSRTHTFPAGLCPTVGVGGHISGAGYGALLRKYGLSADHVIDARLVDVNGRILDK
ESMEEDLFWAIRGGGGASFGVILAWKLRLLPVPPTVTIFILPKTLEQGATSLVHKWQFVADNLHEDLFLG
LSIQTMNIDDDRRKGEKTILVSFFCLYLGGVENLVQMMKQSFPELGLKKDDCLEMSWIRSAVYFGVYSNE
RSLSVLLERKNPVPPKMHYMVKSDYVKEPFSENVLEGIWKFFYEVDMPEIIMSPYGGKMSEISETAIAFP
HRKGNLYKINYLVSWNEEGDEVSEKNANWIRELYKYMTPYVSKNPRASYLNFKDIELGHKKNGSATYLEA
KVWGSKYFMKNFDRLVSVKTKVDPDDFFTNEQGIPPLSS
>XSIFADX1
MGISSFVVLFPFLVFIALASSSSSQDEGFLQCLDLHSNSSIPVYNPSSSSYTSILHASIYNLKFISPATP
KPYFIITPMRESHVQATVVCCREHGLQIRTRSGGHDFEGLSFVAHVPFVLLDLIHLRTISVDIEDETAWV
QTGATLGELYYRIAEKSRTHAFPAGLCPSVGVGGHISGAGYGILLRKHGLSADHVIDARLVDVNGRILDK

ESMGEDYFWAIRGGGGASFGVILAWKIRLVPVPPTVTTFIVPRVLEQGATALVHKWQFIADKLDDDLFLG
LSVQAMHLDPDHPEKKTVVISFLGFYLGAAEKVLQLIDESFPELGLMKADCIEMSWIRSAVYFGIFQLEN
NLSLLLERRNPIPPKNRYMTKSDYVQEPVSEDVLEGIWKRFYEVDEPEIIMSPYGGKMNEIEESAIAFPH
RKGNMYKINYLVSWKELGEEAEKKHASWIRELYNYMTPYVSKSPRCSYLNFKDIDLGHKKNGTATYLEAK
VWGSKYFKNNFDRLVSVKTKVDPDDFFTNEQSIPPLPSLAEPKK
>NSAFADX1
ATSSMVAVLALLLTFSLATSASVHESFLQCLGSEPSTPLYTPNTSSYTSVLQFSVRNFRFSTSSTPKPDL
IVTPLDESQVQAVVVCSRKHGLMMKIRSGGHDYEGLSYVSDVPFIILDLINLDSISVDANKGTAWVQSGA
SIGQLYYRIAEKSRTYGFPAGVCTTMGVGGHFSGGGYGSLMRKYGVAADHIIDARIVNVDGKILDRRTMG
KELFWAIRGGGGSSFGVVLSWKIRLVPVPPTLTVFRLFKTLEQGATTLLERWQYVAHKLPREIFFQVLVR
TENSTDGKNTRRIQASFQSVFLGKAEKLVPIMEQHFPELGLKQDDCIEMSWIQSTLWLLGLPADGSLEVL
LNRSSLTFDTFKAKTDYVTEPISVAGIEGVWKFVLEQNRPLLLLTAYGGRMSKIPESSIPFPHRKGNLYK
LAQVVYWEGEGTEEINWIRRLNRYLTPYVSKSPRATYVNYNDLDLGQSKNGTSSYSAARAWGNKYFKNNF
DRLVKVKTKVDPDNFFRNAQSIPTSVK
>MAQFADX1
PKPNFIVTPMQESHVQTSVICCRKHGLQMRIRSGGHDFEGLSYVSNVPFVVLDLIHFRTIRVDIEDNSAW
VQTGATIGELYYRIAEKSRVHAFPAGLCPTVGVGGHISGAGYGVLIRKYGVSADHVIDARIVNADGEILD
RESMGEXLFWAIRGGGGASFGVILAWKIRLVPVPPTVSIFIVSKTLEEGANALLHKWQFIGDNVHEDLYI
GISMRREIISPKGDKTILVSFIGHFLGGSEKLVQHMEQSFPELGVKPNDCVEMSWIKSAVVFGVFSNDAL
LNVLLDRKNPFPPKSYHKVKSDYVAEPLPVTVLEGILKRFLKNGIDKPEIILSPYGGRMNEISESEIAFP
HRKGNLYKINYIAEWEEAGSMEKHLSWIRELYEYMTPYVSKSPRSSYLNFKDIDLGQMKNGTATYSQAKA
WGSKYFKNNFKRLVQVKTKVDPNNFFFNEQGIPPFHHRAN
>NDOFADX1
MGSSNFATLSFLCIFLLLVSTSSCADEENFLQCLDFHSQNSIPVYTPNTSSYRSILESPIYNLRFLSSTF
RKPLFIVTPMQESHVQTTVTCCKKHGLQLRVRSGGHDFEGLSSISTVPFVILDLVHHRSINVDIEDNSAW
VQAGATIGELYYRIAEKSKVHAFPAGLCPTVGVGGHISGAGYGVLIRKFGVSADYVLDARIVNADGEILD
RKSMGEDLFWALRGGGGASFGVILAWKIQLVHVPPTVTIFIIPKTLEQGATSILHKWQFIADNVDEDLFI
GISIRPEDKVDPKEGKTILVSFLGIFLGGPEELLQHMDHSFPELGLKRHDCIEMSWIKSAVVFGVFSTEV
PLNALLDKKNPFPPKSFHRVKSDYVKEPLSVTVLEEIWRRFLEMGVHKPEIIMSPYGGRMNEISESAIAF
PHRKGNIYKINYIIEWFDEETNTAKKHTSWIRDLYKYMTPYVSKSPRASYLNFKDIDLGHTKNGTASYSE
AYKAWGSKYFKNNFKRLTQIKAKVDPNNFFWNEQGIPLLSS
>BTHFADX1
MFVVVASSIFTTFSLLSLLLPTSLASSDANYEDFLQCLDLYSQNSIPVYTRNTSSYTSILESTIKNLVFL
SPTTPKPNFIVTPMQESHVQTSVICCRKHGLQMRIRSGGHDFEGLSYVSNVPFVVLDLIHLKTINVDIEE
NSAWVQTGATIGELYYRIAEKSRVHAFPAGLCPTVGVGGHISGAGYGVLMRKYGVSADHVIDARIVNVDG
EILDRESMGEDLFWAIRGGGGASFGVILAWKIRLVPVPPTVTIFIVPKTLEEGATALLHKWQFIGDNVHE
DLYIGLSMRSVIISPKGDKTILVSFIGLFLGGSDKLVQHMEQSFPELGVKPHDCIEMSWIKSTVAFGVFS
NDAPLSVLLDRKNPFPPKSYHKVKSDYVTEPLPISVLEGICERFLKNGVDKPEIIMSPYGGRMNDISESE
IAFPHRKGNLYKINYIAEWEEAGSMENHLSWIRELYKYMTPYVSKSPRSSYLNFKDIDLGQTKNGTATYS
QAKAWGSKYFKNNFKRLMQVKTKVDPNNFFSNEQGIPPFSS
>JDIFADX1
MAFSDSSTVTLLSMILTFTISWAASDSSHENFLQCLDVHSQNSIPVYKPNTTSYTSILKSSVYNLKFLSS
TTAKPLFIITPMHEAHVQTTVVCCRKHRLHIRIRSGGHDFEGLSYVSSAPFVILDLFHLKSIDVDIEDNS
VWVQAGANIGELYYRIAEKSKVHAFPAGLCPTVGVGGHISGAGYGVLVRKYGVSADYVVDARIVNVDGEI
LDRKSMGEDLFWAIRGGGGASFGVILAWKLQLVHVPANVTIFIVPKTLEQNATALLHKWQFIGDNIHEDL
FIGISIRPEEVAPNGEKTILVSFLALFLGGTEKLLPLMEESFPELGLKPEDCIEMSWIKSAVTFGVFSNE
ASLNVLLDRKNPFPPKSFHKIKSDYVKEPLPIAALEEIWRKFLEVGVEKPEIIMSPYGGIMNEIPESAIA
FPHRKGNIYKINYIVEWLEEGTEATNKHETWIRELYSYMTPYVSKSPRASYLNFKDIDLGQTKNGSASYS
QAKGWGNKYFKNNFKRLVHVKTKVDPDNFFWNEQGIPPQSS
>MCAFADX1

MSTSIPSIFTLFCVLLISISRASSSSAHQNFLQCLSLHSDQSPIPVYTPNTSSYSSILQYSIQNLRFITP
TTPKPQLIVTPSHDSHVQAAVICCRKHGLQIRVRSGGHDYEGLSYMSDVPFVIVDLITFQSIDVDLEDGS
AWVQAGATIGQVYYRIAEKTRAYGFPAGGCPTLGVGGHFSAGGFGSLFRKYGLAADNIIDARLVNADGQI
LDKESMGEDLFWAIRGGGAASFGVILSWKIKLVPVPPTVTIFSIGKSLEQGATALVHRWQYIAYELPKDL
LLAVFMRRVNASQEREGTVQATFLSVFQGRTEQLLHIMDQRFPELGVKSEDCMEMSWIQSSLYLAGFPID
GPADVLLQRNQSRRFFKAKSDFVKEPIPETALEGIWKRFYEVETPEMILVPYGGRMSEIPESELPYSHRK
GNMYSIQYLAWWEQEGPKADTKNVNWVRRWYRYMAPYVSKSPRSAYLNYRDLDLGQSKNGTASYSQARVW
GIKYYNNNFDRLVQVKSKVDPDNFFRNEQSIPSVPARKEK
>CTRFADX1
ISSTLLVSIFHILVFSISFSASNSPNYESFLSCLSNHSLDHSHIPIYLPNTSSYNSILNSSIRNLRFSSP
QTPKPHLIIKPLHESHVQAAVICSKKHSFQIRTLSGGHDYEGLSFTSHLNLPFVILDLFNFQSINVDVKD
ETAWVQSGATLGQLYYKIAEKSLIHGFPAGICTTIGVGGHFSGGGYGTLKRKYGLAADNIIDARIVNSNG
EILDRESMGEDLFWAIRGGGAASFGVVLSWKIKLVQVPPTVTVFAISKTVEQGAISLXHKWQYIAHKLXE
DLMLLVQLRRANSTTQASFRSLFLGRKEQLLQIMQXSFPELGLKREDCIETSWIRSTLFFFDQPFNGSXS
ILLDRNSIPKVFAKIKSDFVKKPISEAGLQGIWRRYSEVDDPVTLITPYGGKMVEIAESAIPFPHRNGNI
YLIGYEVDWTKGGAEASTKHIGWIRNLYEYLTPYVSKSPRASYLNYRDLDLGENRIDGXASYEEAKVWGV
KYFKSNFDRLVEVKSKVDPDNFFSYEQSIPPLPNKGASTAYXTFVYLCXKX
>TCOFADX1
MILLSHIFLFSISWWSSVLAQENFLQCLSLHSDQPPIPVYTPNTILLNSINPPIIPIQNLRFISSDTPKP
QVIITPSHESHVQAAVICCRKHGLQIRVRSGGHDYEGLSYTSDVPFVVVDLANFESIVVDVEDRSAWVQA
GATIGQVYYRIAEKTSAYGFPAGACPTVGVGGHFSAGGYGGLFRKYGLAADNIIDARIVTVDGKISDKES
MGEDLFWAIRGGGGGSFGVILSWKIRLVPVPPVMTLSTVSRRLEQGATELVYKWQNIAHKLPEDLVFAVF
IRRVNGSEETNDKIQAIFLSVFQGRTKQLLQIMEQKFPELGLKSEDCIEMSWIQSALYLAEFPFDGPRDV
LLNRNQRRRLFKAKSDYVKETIPEIGLKGIWTFFNEVDIPEMILSPYGGRMSEISESALPFSHRKGNLFK
IQYIAWWEKAGAEESKKNINWIRRLYKYMTPYVSKSPRSAYLNYRDLDLGHSENGTASYDQARVWGIKYF
GNNFDRLVHVKSKVDPENFFRNEQSIPSVPEMIMNTES
>CMUFADX1
MGISIWRLSLFQILLFIISSAASAGRENIFQCLSNHSTQSPIPLYVSNTPSYSLILQSSIQNLLFLSPKT
PKPLFIIAPLHESHVQAAVICCRKHGLQIKIRSGGHDFEGLSYISYVPFVILDLFNLKSISIDVEDQTAW
VQAGATLGELYYKIAEKTPTLGFPAGICPTVGVGGHLSGGGYGYLMRKYGLAADNIVDAQIVNVEGEVLN
RESMGEDLFWAIRGGGGANFGVILSWKIKLVPVPPTVTIFRVPKTLDQGANGLVHKLQYIAHKLPKDLML
EVLTGRANASQAGKKTILIIFQALFLGGREQLLRIMEQSFPELGLKVEDCAEISWFQSALNFSGLPMNAT
LSTLAVNPFKGSYKIKSDYVKDPISESGLQGIWKNLLEADRLNVLITPYGGRMSEIAASETPFPHREGNI
YQISYLVSWNKEEAAEKSIGWLRKLYKYMEPYVSKSPRSAYLNYRDIDLGHEFKNGTSSYARAKVWGSSY
FGNNFDRLVQVKSKVDPYNFFRNEQGIPPVN

NCS

>BPEBETV1
MGVFNYETEATSVIPAARMFKAFILDGDKLVPKVAPQAISSVENIEGNGGPGTIKKINFPEGFPFKYVKD
RVDEVDHTNFKYNYSVIEGGPVGDTLEKISNEIKIVATPDGGCVLKISNKYHTKGNHEVKAEQVKASKEM
GETLLRAVESYLLAHSDAYN
>PBRNCS1
MMRKVIKYDMEVATSADSVWAVYSSPDIPRLLRDVLLPGVFEKLDVIEGNGGVGTVLDIAFPPGAVPRSY
KEKFVNIDRVKRLKEVIMIEGGYLDMGCTFYLDRIHVVEKTPNSCVIESSIIYEVKEEFADKMAKLITTE
PLQSMAEVISGYVLKKRLQVFGFEIKPNLRFNLLLCLIICLVIAGGMLIGRVP
>TFLNCS1
MMKMEVVFVFLMLLGTINCQKLILTGRPFLHHQGIINQVSTVTKVIHHELEVAASADDIWTVYSWPGLAK
HLPDLLPGAFEKLEIIGDGGVGTILDMTFVPGEFPHEYKEKFILVDNEHRLKKVQMIEGGYLDLGVTYYM
DTIHVVPTGKDSCVIKSSTEYHVKPEFVKIVEPLITTGPLAAMADAISKLVLEHKSKSNSDEIEAAIITV

>PSONCS1
MSKLITTEPLKSMAEVISNYAMKQQSVSERNIPKKQSLLRKEITYETEVQTSADSIWNVYSSPDIPRLLR
DVLLPGVFEKLDVIAGNGGVGTVLDIAFPLGAVPRRYKEKFVKINHEKRLKEVVMIEGGYLDMGCTFYMD
RIHIFEKTPNSCVIESSIIYEVKEEYAGKMAKLITTEPLESMAEVISGYVLKKRLQVFGFEIKPKLRFNL
LLCLIICLVIAGGMFVAGVPL
>PSONCS2
MSKLITTEPLKSMAEVISNYVIQRESFSARNILNKNSLVKKEIRYDLEVPTSADSIWSVYSCPDIPRLLR
DVLLPGVFQKLDVIEGNGGVGTVLDIVFPPGAVPRSYKEKFVNINHEKRLKEVIMIEGGYLDMGCTFYMD
RIHIFEKTPNSCVIESSIIYEVKEEYAGKMAKLITTEPLESMAEVISGYVLKKRLQVFGFEIKPKLRFNL
LLCLIICLVIAGGMFVAGVPL
>CJANCS1
MRMEVVLVVFLMFIGTINCERLIFNGRPLLHRVTKEETVMLYHELEVAASADEVWSVEGSPELGLHLPDL
LPAGIFAKFEITGDGGEGSILDMTFPPGQFPHHYREKFVFFDHKNRYKLVEQIDGDFFDLGVTYYMDTIR
VVATGPDSCVIKSTTEYHVKPEFAKIVKPLIDTVPLAIMSEAIAKVVLENKHKSSE
>PBRNCS2
MMRKVIKYDMEVATSADSVWAVYSSPDIPRLLRDVLLPGVFEKLDVIEGNGGVGTVLDIAFPPGAVPRSY
KEKFVNIDRVKRLKEVIMIEGGYLDMGCTFYLDRIHVVEKTPSSCVIESSIVYEVEEEYADAMSKLITTE
PLKSMAEVISNYVIQKESVSARNIFNRQSVVKKEIHYDLEVPTSADSIWAVYSNPDIPRLLRDVLLPGVF
EKLDVIEGNGGVGTVLDIVFPPGAVPRCYKEKFVTMDHQKRLKEVIMIEGGYLDMGCTSYLDRIHVIEKT
SKSCIIKSSVVYEVKQECAEAISKLITTEPLKSMAEVISNYVLKKQSVSDTNNIAKKQSVLRKEITYETE
VQTSADSIWNVYSSPDIPPTT
>PBRNCS3
MDIIEGDGGVGTVLDVVFQPGAVPQSYKERFETVDHEKRILEVRIIQGGYLEMGCTSYLNRMHVIEITSK
SCVIKSSVIYDVKEECADAMSKLITTIQLESMAKVVADYVLKKQSASDTSIPKKQSLMRKEITHEMEVQT
SADSIWDIYSSPDIPRLLRDVLLPGAFEKLHVIQGNGGVGTVLDIALPLGAVPRNYKEKFVKINHEKRLK
EAVMIEGGYADMGCTFYMHRIHVLEKTPKSCVIESSIVYEVKEEYADKMSKLITTEPLQSMAEAISSYVL
KKQFQVFGLEVKPKLVLSLFLCLIIFLAIVGGFLIGGLKA
>PBRNCS4
MIEGGYLDMGCTFYLDRIHVVEKTPSSCVIESSIVYEVKQECAEAISKLITTEPLKSMAEVIANYVLKKQ
SVSDTNIPKKQSVLRKEITYETEVQTSVDSIWNVYSSPDIPRLLRDVLLPGVFEKLDVIAGNGGVGTVLD
IAFPLGAVPRRYKERFVKINHEKRLKEVVMIEGGYLDMGCTFYMDRIHVFDKTPNSCVIESSIIYEVKEE
YADKMAKLITTEPLQSMAEVISGYVLKKRLQVFGFEIKPNLRFNLLLCLIICLVIAGGMLIGRVPL
>PBRNCS5
MMRKVIKYDMEVATSADSVWAVYSSPDIPRLLRDVLLPGVFEKLDVIEGNGGVGTVLDIAFPPGAVPRSY
KEKFVNIDRVKRLKEVIMIEGGYLDMGCTFYLDRIHVVEKTPSSCVIESSIVYEVEEEYADAMSKLITTE
PLKSMAEVISNYVIQKESVSARNIFNRQSVVKKEIRYDLEVPTSADSIWAVYSNPDIPRLLRDVLLPGVF
EKLDVIEGNGGVGTVLDIVFPPGAVPRRYKEKFVNINHEKRLKEVIMIEGGYLDMGCTFYLDRIHVVEKT
SKSCIIKSSIVYEVKQECAEAISKLITTEPLKSMAQVIANYVLKKQSVSDTNIPKKQSVLRKEITYETEV
QTSVDSIWNVYSSPDIPRLLRDVLLPGVFEKLDVIAGNGGVGTVLDIAFPLGAVPRRYKEKFVKINHEKR
LKEVIMIEGGYLDMGCTFYMDRIHVLEKTPNSCVIESSIIYEVKEEFADKMAKLITTEPLQSMAEVISAY
VLRKRFEVFGLEIKQKLRYNLLLCLIICLVIAGGMLIGRVPL
>SCANCS1
MRKELTHEMEVPASADAIWAVYSSHDIPRLLKEVLLPGVFEKLDVIAGDGGVGTVLDIAFPPGAVPRRYK
EKFVKINHEKRLKEVEMIEGGYLDMGCTFYMDRIHVVEKGPNSCVIESAIIYVVKDECADVVVPLITTEP
LASMAEVISNYVLRKQIRLFGYVIKPKLGLSILLSLILCLVILGVLLIGGVPF
>CMANCS1
MIEGGYLDMGCTFYMDRIHVVKKGPNSCVIASAIIYEVKEEFVDVVVPLITTEPLASMAEVISNYVLKKQ
RRVRKELTYEMEVPTSADSIWAVYSSHDIPRLLKEVLLPGVFEKLDVIEGDGGVGTVLDIAFPPGAVPRT
YKEKFVKINHEKRLKEVVMIEGGYLDMGCTFYMDRIHVLEKSPNSCVIESSIIYEVKEEFADVVGPLITT
EPLASMSEVISNYVLKKQIRMFGYVIKPKLGLSLLLCFILCLVLLGVLLIGGVPL

>SDINCS1
MRKEVRYEMEVPTSADSIWAVYSSHDIPRLLKEVLLPGVFEKLDVIEGDGGVGTVLDIAFPPGAVPRTYK
EKFVTINHEKRLKEVIMIEGGYLDMGCTFYMDRIHVLEKGPKSCIIASAIIYEVKEEFADVVVPLITTEP
LASMAEVISNYVLKKQRRVRKELTYEMEVPTSADSIWAVYSSHDIPRLLKEVLLPGVFEKLDVIEGDGGV
GTVLDIAFPPGAVPRTYKEKFVKINHEKRLKEVVMIEGGYLDMGCTFYMDRIHVLEKGPNSCVIESAIIY
EVKEEFADVVVPLITTEPLASMAEVISNYVLKKQIHVFGYVIKPKLGLSLLLCFILCLVLLGVLLIGGVP
L
>ECANCS1
MIGGFLDMGCTFYMDRIHVVAKGPNSCIIKSTLIYEVKEEYADAMASLITVEPLASMAEVVANYVLHQQV
RVLGSVKRKELTHELEVAAPADAIWGVYSSPDIPRLLRDVLLPGVFEKLEVIQGNGGVGTVLEIVFHPGA
IPRRYKEKFVTINHKKRLKEVVMIGGYLDMGCTLYMDRIHVVSKGPNSCVIKSTLIYEVKAESADAMAST
ITIDPLASMAQVISNYVLKNQMQVLGSVKRRELTHELEVAASADAIWGVYGSKRYSKASQGCFASWCFRK
VRSH
>ECANCS2
MIGGYLDMGCTLYMDRIHVVEKGPNSCVIKSTLIYEVKAEYADAMASLITVDPLASMAQVISNYVLKNQG
QVLGSIKRRELKHELEVAVSADAIWGVIGSKDIPRLLRDVLLPGVFEKLDVIEGDGGVGTVLEIVFPPGA
VPRKYREKFVKVDHEKRLKEVIMIGGYLDMGCTFYMDRIHVVAKGPNSCIIKSTLIYEVKEEYADAMASL
ITVEPLASMAEVVANYVLHQQVRVLGSVKRKELTHELEVAASADAIWGVYSSPDIPRLLRDVLLPGVFEK
LEVIQGNGGVGTVLEIVFHPGAIPRRYKEKFVTINHKKRLKEVVMIGGYLDMGCTLYMDRIHVVSKGPNS
CVIKSTLIYEVKAESADAMASTITIDPLASMAQVISNYVLKNQMQVLGSVKRRELTHELEVAASADAIWG
VYGSKDIPRLLRDVLLPGVFEKLEVIEGDGGVGTVLQIVFPPGAIPRRYKEKFVKVDQKLRLKEVIMIGG
YLDMGCTFYMDRIHVVPKGLNSCVIKSTLIYEVKDEYADAMSSLITVEPLASMAEVVSNYVLNKKKLMIT
RKELTHELEVAASADAIWSVYSSPDIPRLLRDVLLPGVFEKLEVVQGNGGVGTVLEIVFPKGSVPRRYKE
KFVKINDEKKLKEVIMIEGGYLDLGCTFYMDKIHVLPKGPNSCVIESSLIYEVKEENAKAMASLITVEPL
ASMAEVVANYVLKKQIRVLGYVVKPRVGYSVLVGLLLCLVLLGVLLLSGVNI
>AMENCS1
MRKEVVYELEVPTSADSIWAVYSSPNIPTLLRDVLLPGVFEKLDVIEGNGGVGTVLNIVFPPGAVPRXYK
EKFINIDNKKRLKEVIMIEGGYLDMGCTFYMDRIHVIAETPNSCVIKSSIIYDVKKEYAEAMSKLITTIP
LKSMSEVIANYVLKNQSVIRKEVTYELQVPTSD
>AMENCS2
MKFELVNELEVPASANDVWAIYSSPDFPKLLTKLVPGILESVEYVEGDGHLGTVIHLVYVPGSVPLSYKE
KFVTIDHEKRLKEAVHVEGGFLEMGVTFYMNSFEIIEKGSDCCIIRSMTKCEIEDKEIMNLISHISVANV
TVLAMTISKYVQQHKK
>TFLNCS2
MKMEVVFVFFMILGTINCQKLILTGRPFLNRQGIINQVSTVTKGVHHELEVAASADDIWSVYSWPGLAKH
LPDLLPGAFEKLEIIGDGGVGTILDMTFTPGEFPHEYKEKFILVDNEHRLKKVQMIEGGYLDLGVTYYMD
TIQVIPTGTNSCVIKSSTEYHVKPEFVKIVEPLITTGPLAAMAEAISKLVLEHKYKSNSDEIDASKNNLK
MVINM
>TFLNCS3
MKMEATVFVFLMFLGTINCQKLIMAGRPFLHHQGIINQEFTVTKVLHHELEVAASADDIWGVYSSPHLVF
HLTDLLPGAFEKVQVIGDGGVGTILDMTFAPGEFPHEYKEKFIVVDNEHRLKKVQMIEGGYLDLGVTYYM
DTIQVVPTGTNSCVIKSSTEYHVKPELLKIVEPLITTGPVAAMAEAISKLVLEYKYKSHSDEIHAGLNNN
LKMVINNI
>TFLNCS4
MRKELTHEMEVPASADAIWAVYGSPDIPRLLKEVLLPGVFEKLDVIEGDGGVGTVLDIAFPPGAVPRAYK
EKFMKVNHEKRLKEVEMIEGGYLDMGCTFYMDRIHVVEKGPNACVIESAIIYEVKDEFADVVVPLITTEP
LASMAEVISNYVLKNQFRVFGYVIKPKLGLSLLLCFILCLVLLGGLLIGGVPL
>TFLNCS5
MRKELTNEMEVAASADEIWAVYSSPNLPKLIVQLLPAVFERIYILEGDGGVGTVLYILSPPGSVPRSYKE
KFITIDHEKRLKEVQEIEGGYLDMGVTFYMDTFYILEKGPDSCIIKSMTTYEIKDELADKVASLISIDSL

VGMAKAITKYVLDQKKAAVDSSA
>BTHNCS1
MVVAASADDVWAIYSSHDLPKLIVKLLPSVFKSIEIVEGDGGLGTVLDVKYPPGSIPLHYREKFITIDNE
KRLKEVRQIEDGLLALGCTFYMDSFHILEKDCHHEFFHIHEKNCHKKCIIKSTTVYEVPDELAYKIEPLV
TIDSLVGMAHAISKYVLDKSC
>CCHNCS1
MYYFLEFFEKLDVIEGNGGVGTVLDIAFPPGAVPRSYKEKFVKVDHKNRLKEVVMIEGGYLDLGCTFYMD
RIHVLPKGANSCVIKSTLIYEIPDELVDSVGSLMSTEPLASMAKVISDYVLKQRKMTANKILRKELKTEM
EVATSADSIWAVYGSPDIPRLLRDVLLPGVFEKLDVIEGNGGVGTVLDIAFPPGAVPRTYKEKFVKVDHK
NRLKEVVMIEGGYLDLGCTFYMDRIHVLPKGPNTCVIKSTLIYEVPDEFADAVGSLISVEPLASMAEVIS
GYVLKQKKEAKKILRKELTHELEVPTSADSIWAVYGSPDIPRLLRDVLLPGVFEKLDIVEGNGGVGTVLD
IAFPPGAVPRSYKEKFVKVDHDKHLKEVVMIEGGYLDLGCTFYMDRIHVLPKGPNSCVIESSLIYEVREE
LADVVGSLISIEPLASMAEVISSYVLKQQLRVFGVVVQPRVGLSLLLCLILCLVILGGLLIGGVSI
>CCHNCS2
MRKELRHELEVATSADSIWAVYGSPDIPRLLRDVLLPGVFEKLDVIQGNGGVGTVLDIAFPPGAVPRTYK
EKFVKVDHKNRLKEVVMIEGGYLDLGCTFYMDRIHVLPSGPNTCIIKSTLIYEVPDELAYSVASLISVEP
LASMAEVISGYVLRQRKMTTNKILRKELTTEMEVPTSADSIWAVYGSPDIPRLLRDVLLPGVFERLDVIE
GNGGVGTVLDISFPPGAVPRSYKEKFVKVDHKNRLKEVVMIEGGYLDLGCTFYMDRIHVIPKGPNSCVIK
STLIYEIPGELVDSVGSLMSTEPLASMAAVISDYVLKQRKMTANQILRKELTTEMELATSADSIWSVYGS
PDIPRLLRDVLLPGVFERLGCH
>CCHNCS3
MMRKELVHEKEVCASADAVWGVYSSPNIPTLLRDKLLPGMFKRLEILEGDGGVGTILLLEFNNPAIIPHT
YLEKFMKLDHEKRLLEVEVVKGGYLDLGCTFYMSRIHILEKGPNSCVIESTLIFEAPEELMEYVSQYANL
ESLISMAEVISKYVLEQQFRVFGVVVKKLKLGLSTIVLLCIFIFLVIVLGGLWIEGVSI
>CCHNCS4
MRKHLVNELEVVVPADTLWAIYSTTQFPKLIVQLLPIVVQNIEIDGDGSLGTVLNVIFVPGSVPLSYKEK
IVTIDHEKRLKEVVQIEGGYLDLGCSFYMSSFQILEKGRDSCIIKSMVTYELAKDADPSVADLVTIAAHA
AIAQVISKYVLDKQVAAAP
>CCHNCS5
MRKELTNELEVAAPADAVWAVYSSPDLPKIIVELLPSVFEKIEIVEGDGGVGTVLYVVFPPGSVPLTYKE
KFVTIDHEKRLKEVLQIEGGYLDLGCTFYMDSFHILEKDCDSCIIKSITAYEVRDDVVDNVSSLISIDSL
ANMAEAISKYVLEKQEAATKHGHGDDRERTGLCWPFNCLG
>NDONCS1
MRKGIVFLFLVFLGCEVSQGRQLLESRLFRKSTIQKVLHHELPVAASAQEVWDVYSSPELPKHLPEILPG
AFEKVVVTGDGGVGTVLEMVFPPGEVPRSYKEKFVLIDDEQLLKKVEMIEGGYLDMGCTFYMDTIQIVPT
GPDSCIIKSSTEYYVKPEFADKVVPLISTIPLQAMAEAISNIVLANKAKNKSIIIEI
>NDONCS2
MVFPPGEVPRSYKEKFVLIDDEQLLKKVEMIEGGYLNDLDCVHIKRTSHVQISTFNHFDMGCTFYMDTIQ
IVPTGPDSCIIKSSTEYYVKPEFADKVVPLISTIPLQAMAEAISNIVLDKTKDQRNKEVINTNTKNNKIH
HRYVATIVIIR
>NDONCS3
MRSGIVFLVLFFLGCEISQGRQLLESRLFRKSTIRKVLHHELPVAASAQEVWDVYSSPELPKHLPEILPG
AFKKVVVTGDGGVGTVIEMVFPPGVVPHRYKEKFVLIDDEKFLKKVEMIEGGYLDMGCTFYMDTIQIVPT
GPDSCIIKSSTEYYVKPEFADKVVPLISTVPLQAMAEAIAKIVLEFKAKHKGFIEI
>NDONCS4
MEVAASAGDIWAVYSSPDLPRLIVQLLPTVFEKIDIVEGDGGVGTVLHITFPPGSVPLTYKEKFVTIDNA
NRLKEVLQIEGGYLELGCTFYMDSFQIFEKGIDSCIIKSMTTYEVPDELADKVAPLISIDSLVPMAEAIS
KYVIEKRH
>CTRNCS1
MIKKELKHEMRVAASADDIWAVYSSPDLPNLILRLLPSVFDNIEIVEGNGGVGTVLHLTFPPGSVPLSYK

EKFVTINGNKRLKEVKQIQGGYLDMGCTFYMDSFHIEEKGCDSCVIVSKTEYEVPNEEIANQVELYISID
SLASMAQGHLGLCS
>HCANCS1
MKMAILFVFLMFLGKMNSEGLHLSGRPLLRAIISDKPNVIKVLKHELAVPASADKVWAVYSAPTLAFHLS
DLLPGAFEKVEVFGDGGVGTIIDMTFAPGEFPHEYKEKFILIDGKQRLKKVQMIEGGYLDLGVTYYMDTI
HVVPTGSNSCIIKSSTEYHVKPEAAKLVEPLITTEPLAAMAEVITKIVLENKSKSSEENQSSEAI
>NSANCS1
MVQFSRESKQISIISDEEEGGEEETKEKKMMKVQVALAFLLILGAASCQELILQGRPLLGGARAWGTKSI
KKELKHEFKVAASADEVWSVYSAPELCKHLTDLLPGAFEDVEIIGDGGVGTILHMIFPPGEFPHEYKEKF
VVIDDKQRLKKVEMIEGGYLDIGVTYYMDTIHVVPTGSDSCVIKSSTEYHVKPEFEKIVEPLITTVPLAA
MAEAIAKIVLDNKTHSITI
>NSANCS2
MVKIQLVLACLLLVVGAVNCQKLILQGRPLLGAWACGTIKKVLKHEFKVAASADEVWSVYSSPELCKHLT
DLLPGAFQDLEIIGDGGVGTILHMTFPPGEFPHEYKEKFVLIDDKRKLKKVEMIKGGYLDIGVTYYMDTI
HVVPTGSDSCVIKSSTEYHVRPECEKIVEPLITTEPLAAMAEAVSKIVLDAKIHSIITI
>MCANCS1
MIKKELKHELEVATSADEIWEVYSSPDLPILIVKLLPSVFEKIEILEGDGGVGTALRLTFPIGSVPLTYK
EKFVTINDWKRLKEVKQIEGGYLDMGCTFYMDSFHILRKGPKSCVIVSKTEYEVPNKEIASKVEPYISID
SLRKMATAISDYVLNRATRKEVKHELEVAASADDVWEGYRSPDVGSLICPHVFEKIELVEGDGGVGTILQ
ITHPPGYVPHTYKEKYVTLDDHKRLLEVEQIEGGYLEMGCTFYMDSIHVVKKGDNSCVIVSKAKYEVPKE
LASQVEPYIAADAVANMARIISNNVLEKKKS
>XSINCS1
MRMEVVLVVFLLFIGTVNCERMIFSGRPLLHRVTNEETVILYHELEVPASVDELWSVEGSPELGKNLPDL
LPGIFADFKITGDGGEGSILDMTFPPGQFPHHYREKFVFFDHKNHYKLVQMIDGDFFDLGVTYYMDTIRV
VATGPDSCVIKSSTEYHVKVEFAKIVKPLIDTVPLAIMSEAIAKVVLEKKYKRSE

NMT

>MTUMMA2
MVNDLTPHFEDVQAHYDLSDDFFRLFLDPTQTYSCAHFEREDMTLEEAQIAKIDLALGKLGLQPGMTLLD
IGCGWGATMRRAIAQYDVNVVGLTLSKNQAAHVQKSFDEMDTPRDRRVLLAGWEQFNEPVDRIVSIGAFE
HFGHDRHADFFARAHKILPPDGVLLLHTITGLTRQQMVDHGLPLTLWLARFLKFIATEIFPGGQPPTIEM
VEEQSAKTGFTLTRRQSLQPHYARTLDLWAEALQEHKSEAIAIQSEEVYERYMKYLTGCAKLFRVGYIDV
NQFTLAK
>AMENMT1
MCLFFAEKMGLMAEANNQQQLKKEDLLKNMELGLIPDEEIRKLIRVQLEKRLNWGYKSTHEQQLSQLLHL
VHSLKKMKIATEMENLDLKLYEAPFSFVQIQHGSTIKESSGLFKDESTTLDEAEIAMLDLYTKRAKIEDG
QSVLDLGCGLGAVTLYVAQKFKNCYVTGITSSVEQKDFIEGRCKELKLSNVKVILADITTYETEEKYNRI
FAVELIEHMKNYELLLRKISEWMKQDGLLFIEHVCHKTLAYHYEPLDEEDWYTNYIFPAGTLTLSSATLL
LYFQDDVAVVDQWTLSGKHYSRSHEEWLKRIDGNIEEVKEIMKSITKSEEEAKKLLNFWRIFCMCGAELF
GYKNGEEWMMTHILFKKK
>AMENMT2
MDNCKVEGMKKETRAAEEILGRLLKGEIKDEELRKLIKFQFQKRLEWGYKSTHHQQLSFNLDFIKSLKNM
DMSGEIETMNKETYELPTEFLEAAFGKTMKQSGCYFKHESTTIDEAEEASHELYCERAQIKDGQTVLDIG
CGQGGLVLYIAEKYKKCHVTGLTNSKAQVNYIEMQAKKLGLSNVDVILADVTKYDSDKTYDRLLMIEAIE
HMKNIQLFMKKLSTWMTKDSLLFVDHICHKTFSHPFEAVDEDDWYSGFIFPPGCVTILSSDALLYFQDDV
SVVDHWVINGMHMARSVEAWRKKLDKNMKAAKEILLPGLGGNHEAVNQIVTHIRTFCMGGYEQFSYNNGE
EWMVAQMLFKKK
>AMENMT3
MALKKIMEMPYAATVRVMLASLERNLLPDAVIRQLTRLLLSTRLRLGYAPSAHLQLAQLLQFAHSLRDMP

IAIKTEDPKVQHYELPTSFFKFVLGENLKYSCCYFNDTSSTLDDAEKAMLETYCERSGLEDGHTVLDVGC
GWGSLSIFIAKKYSKCKITGICNSTTQKAFIEERCRDLDLHNVEIIVADISMFDMEASYDRIFSIEMFEH
MKNYKELLKMISRWMKPDSLLFVHHFCHKTFAYHFEDINEDDWITRYFFSGGTMPSANLLLYFQDDVSVV
NHWLVNGKHYAQTSEEWLKRMDKNMSSVRPIMESTYGKDSSVKWTVYWRTFFIAVAELFGYSNGEEWMVA
HFLFKRK
>BTHNMT1
MEVKQAGKEGVTELLVKRMELGLVPEEEIRRLMRIQIQKRLDWGYKPTHEEQLAHLTKFIQNIRGMKMAD
EIDALDAKMYEIPLPFLQTICGKTLKFSPGYFKDESTTLDESETLMMDLYCERAQVKDGQSILDLGCGHG
GFVLHLAQKYRNSVVTGVTNSVSETEYIKEQCKKLGLSNVEIIIADVTKFEPEVTYDRVFAIALIEHMKN
YALVLNKISKWVAQDGYLFVEHHCHKVFPYKYEPLDEDDWYTNYIFPGGTLILPSASILLYFQEDVTVLN
HWSLSGKHPSRGFIEWLKRLDENIDVIMGIFEPFYGSKEEATKWINYWRVFCMTHSEMYAYGNGEEWMLS
QVLLKRK
>BTHNMT2
MDSKQAKKASVVELLERLEEGLVSDEELKKLVHVQLEKRLQWGYKPTHQEQLAFHLDFIKSLKQMDIAGD
MEMMNSESYELPISFLKTMFGKTLKQSACYFESESVTIDEAEIAMNALHCERARLKDGQRILDIGCGQGS
LIFHIANKYKNCLLTGITNSCTQKTYIEEQCKNLKLSNIKVILADVTKYETEDKFDRIVIIEALEHMKNI
ELFLKRTSKWMKEDGLLFVDHVCHKVFPDKFEELDEEDWYSEYIFPKGSVIVNAASTLLYFQDDVSVIDH
WIINGKNMARSHEEWMKNLVKNVDVAKDILKVAMGSEEAAERCIIHCRNFHLSMSELFSYQNGEEWMVSH
VLFKKR
>BTHNMT3 TRANSLATION OF A FRAGMENT OF BTMT3
MGGALIEVSGQKLSKAELLKNIEEGLIPDDELRRLMRGELAKRIQWGYKPTHEQQVAQAINFAHSLRKMK
IANEAETLVEEVYEMPMSFLRLMLGPTLKLSCLYFKNEFTTLEQAEIDTMDMYCKRAGIQDGMSILDLGC
GQGSLTLHIAHKYKNCRITALTNSASQKGNIEDQCKLQNLPNVKVILADIANFDMDETFDRVMVIELFEH
MKNYALLLKKMAKWMSSDGLLFVDTLCQKTFPYHFDPIDEDDWYTNLLFTPGTSILPSASFLLYFQDDVS
IANHWTISGKHFSRTNEEWLKRIDGQVNEIRAVLKSFLGTEKGVEKLINYWRGFCLSGMEMFGYDNGEEW
MTAHYLFRKK
>CCHNMT1
MATSDQEVKTSKMEMIADLLKRLEAGLVPDDEIRSLIRVELERRLKWGYKSTHQEQLDQLLNLAHSIKKM
KIASTEMDGLTSTMYEVPISLVQIQLGSHLKESCLYFKDETTTVDEAEIAMMDLYLERAQIKDGQSILDL
GCGLGAVSFHIAQKYTSCNITAVTNSVRQKEFIEEKSKTLNVPNVKVLLADITTLEMEHTFDRLFAISLI
EHMENYELLLRKLSEWMKQDGLLFIEHLCHKTLSYHFEPMDEDDWYTNLLFPAGTLTLVSASFLLYFQDD
LSVVNQWVMSGKHFSRANEEWLKNMDAKMDEMREIFESITDSEEEVVKLINHWRIFCISSAEMFAYNDGE
EWMNSHVLFKKKKQIQ
>CCHNMT2
MAGSGANKEMIADLLKRLEVGLVPDEEIRSLIRFQLKRRLKWGYKTTHQEQLEQLLSLAHSIRKMKIATE
MDALNSTMYEVPISFMQIVFGSTLKESCLYFKDEATTVNEAEIAMMDLYLERAQIKDGQSILDLGCGMGS
LCFHIARKYTNCNITAVTNSVSQKEFIEEKSKTLNLPNVKVILADITTLEMDDTYDCLFAIGLIEHMKNY
ELLLRKLSNWMKQDSLLFIDHVCHKTLAYHYEPIDEDDWYTNLLFPAGTLTLVSASFLLYFQDDLSLVDH
WSMSGKHFSRTNKEWLKNIDGKMDKIREIVKSITDSEEEVVKLINHWRMLCINSSEMFGFNDGEEWMNSH
VLFKKKKQI
>CCHNMT3
MIKKSKIMAFSDHHHEVVKNHSKKEMIADLLKRLEAGLVPDEEMRNLFRFELERRLQWGYKSIHQEQLSQ
LLKLAHSTKEMTIVAEMDALNSSMYELPISFLQIQLGSNLKQSSLYFKDELTTVDEAEVAIMDLYLERAQ
IEDGQSILDLGCGLGAFSFHVARKYTNCNITAVTNSLTQKEFIEKKSKILNIQNVKVIFADVTTVEMETT
FDRVFAIGLIEHMQNYELFLKKLSKWMKQDGLLFIEHFCHKTLAYHYKPIDEDDWFTNLLYPNGTVISSS
LLLYFQDDVSVVDHWSLSGKHFSRASEESLKRMDAKMDEMKEIFESITDSKEEAMKLINQWRIFCISCAE
MFGYNNGEEWMTSHFLFKKKL
>CCHNMT4
MAVEEGGIGATTEISTKKPEIAELLRKLELGLIPDEEIRRLIRIELGRRLKWGYKPTYEQQLAEVQKLAH
SLRDMNIAKEIDTLDEQMYEVPISFLQIMFGSTIKGSCCYFKEDSMTLDEAEIAMLDLYCERAQIKDGDS

VLDLGCGQGALTLHVAKKFKNSRVTAITNSVSQKEFIEEQARNLNLTNVTVTLADITKHEMEDRFDRILV
IELFEHMKNYELLLRKISNWMTKDGFLFIEHLCHKTFAYNYEPIDEDDWYTEYIFPAGTMIIPSASFFLY
FQDDLTIANHWTLSGKHFSRTKEEWLKRLDANKDAVMAIMEEFSGSKEDAVKWTNYWRGFCFSGMEMYGY
NNGEEWMACHVLFKKD
>CCHNMT5
MEVVATSSARNPKKEIVDLWKRMELGLIPDEEIRDLMKIGLEKRLKWGYKPTHEQQLSQLLHFAKSLRSM
KMASEMETLDDQMYETPTAFQQLMCGSTIKESAGFFKDESTTLDEAEIKMLDLYCEKARIEDGQKILDLG
CGHGAVMLHIAQKYKNCNVTGVTNSISQQQFIVQRSKELNLSNVNMILADVTMLEMDATYDRIFIIGLIE
HMKNFELFLRKISKWITKEGLLFLEHYCHKTFAYQCEPVDEDDWYNMFIFPPGTLILPSASFLLYFQDDL
IVVDRWTLNGNHYARTQEEWLKRIDANVDGVKQMFESVCDGNKEEAVKLMNFWRIFCISGAEMLAYNNGE
EWMISHYLFKKRN
>CCHNMT6
MGSSTASDHEMVIMENDSKNKQVVIADLLKRLVGGLVPDEEMRNMFRFELEKRLKWGYKSTHQQQLSQLL
NLVELNKGIAKIAPEMDALNSAMYEVPIPYLKLMLGSTLKQSCLYFKDESTTLDEAEIEMMDLYLERADI
QDGQSILDLGCGLGGLGFHIAQKYISCNITALTNSLTQKEFIEEKCKTLNIPNVKVILADVTTVEIETTF
DRLFAIGLVEHMENYELFLRKLSKWMKQDGLLFIEHLCHKTLAYHYKPIDEDDWYSNLLYPTGTLTSASF
LLYFQDDLSVVDHWSLSGKHFSRATEEWLKMIDANMDKIREIYESVTESKEEATRSINQWRIFCISCAEM
FGYNDGEEWMISHFLFKNKKQIE
>CCHNMT7 CCHRT1PF_ASSEMBLED CCHRT1PF_REP_C216
MGINFLERLGKREVSDEELKKQIRIIWEKRLQWGYRSTHEDQLKSNLDFVKSLKEMKMSGDIDTFNNESY
ELPSEFLESMFGKTIKQSACYFKHESMTIDEAEDASHELYCERAQIKDGQTVLDIGCGHGGLVLYIAQKY
KNCHVTGLTNSRAQVNYIEMQSKKLNLTNVEAILADVTKHEFKTEKTFDRIILIEAIEHMKNIELFMKKL
STWMTEESLLFVDHICHKTFNHHFEAMDEEDWYSGYIFPKGCVTILSSSALLYFQSDVSVVDHWVVNGNH
MARSVQEWLKKLDDNMETVRGILEPAMGGSKEAVDQVITHSRTFCMGAYEQFSFNNGEEWMVAQILFKKK
>CCHNMT8
MAVVNILERFRKREIGDEEFKQHIRSIWVKRLQWGYRSTHEEQLNSNLDFIKSLKKMDMSGDIDKYNTQE
YELPSEFQQIIFGKSIKQSGCYFKEESKTKTLDEATDDSHELYCERAQIKDGQTVLDIGCGNGGLVLYIA
QKYKNCHVTGLTNSKAQVKHIELQTKKLNLTNVEVILADVTKHVFKTEKTFDRIILIQVIEHMKNIELFM
RKLSTWMTEESLLFVDHICHKTFNYHFEPMDENDWYSGHIFPEGCVTILSSSSLLYFQKDVSVLDHWVVN
GKHMARSAQEWLKSLDDNMEKVREILEPSMGGNKEAVDQVLTQSRFFCMGAYEQFSNNNGEEWMFAQLLF
KKK
>CCHNMT9
MEMEKGSSSGVKILERLKKGEISDEELKKHIRVHWEKRLQWGYKPTFQEQLQSNLDFVKSLKEMEMSGEV
EIPLNIGESDEVSNYQKKRKSYELSSEYLGAMFGKTLKQSACYFKDESMTLDEAEEAAYELYCERAQIKD
GQTVIDIGCGHGGLVLYIAQKYKTCHVTGLTNSKTHVNYIEMQCKKLQLSNVDVILADVTKYDTEKTFDR
IVVVGTIEHMKNIGLFMKKLSTWMKKEGLLFVDHTCHTSFNYHFEAMDEDDWYSGYIFPKGSVTFLSSSA
LLYFQEDVSVVDHWLLSGMHMARSMQEWMKKIEQNMDTLRSLLEQTMEGGSNEAVNQVVTHLRTFCIGAY
ETFSYNNGEELMMAQFLFKKK
>CCHNMT10
MAGEKREMERLFQLPYDATVRMMLNALERNLLPDLVIRKLTRLLLGSRLRWGYKSISDLQLSHLLQFAHS
LEEMPIAIKTDAAKTQHYELPTSFFKLVLGKNMKYSCCQFDKSNTTLEDAEIAMLELYCERAQIKDGHTV
LDIGCGWGSLSLFIAKKYSKCKITGICNSTTQKTHIQDQCRDMNLQNVEIIVADISTFDMESTYDRLVSI
EMFEHMKNYKELLKKISKWMKQDSLFFVHHFCHKTFAYHFEDTNEDDWITRYFFTGGTMPSANLLLYFQE
DVSVVNHWLVNGKHYALTSEEWLKRMDKNLDSIKPIMESTYGKDSAVKWTVYWRTFFISVAELFGYNNGD
EWMVAHLLFKKK
>CMANMT1
MGSIDEVKKESAGETLGRLLKGEIKDEELKKLIKFQFEKRLQWGYKSSHQEQLSFNLDFIKSLKKMEMSG
EIETMNKETYELPSEFLEAVFGKTVKQSMCYFKHESATIDEAEEAAHELYCERAQIKDGQTVLDIGCGQG
GLVLYIAQKYKNCHVTGLTNSKAQVNYLLKQAEKLGLTNVDAILADVTQYESDKTYDRLLMIEAIEHMKN
LQLFMKKLSTWMTKESLLFVDHICHKTFAHFFEAVDEDDWYSGFIFPPGCATILAANSLLYFQDDVSVVD

HWVVNGMHMARSVDIWRKALDKNMEAAKEILLPGLGGSHEAVNGVVTHIRTFCMGGYEQFSMNNGDEWMV
AQLLFKKK
>CMANMT2
MEDQQTLLCVFLCKLKKMQLMAKEELLQNMELGLIPDQEIRQLIRVELEKRLQWGYKETHEEQLSQLLDL
VHSLKGMKMATEMENLDLKLYEAPMEFLKIQHGSNMKQSAGYYTDESTTLDEAEIAMLDLYMERAQIKDG
QSVLDLGCGLGAVALFGANKFKKCQFTGVTSSVEQKDYIQGKCKELKLTNVKVLLADITTYETEERFDRI
FAVELIEHMKNYQLLLKKISEWMKDDGLLFVEHVCHKTLAYHYEPVDAEDWYTNYVFPAGTLTLSSASML
LYFQDDVAVVNQWTLSGKHYSRSHEEWLKNMDKNIVEFKEIMRSITKTEEEANRLLNFWRIFCMCGAELF
GYKNGEEWMLTHLLFKKK
>CMANMT3
MGVEITESSNAKKAEVAELLRKLELGLLPYDEIRRLMRIELGRRLQWGYKPTYEEQLAQVVKLARSLRSM
NIATEIDTLDEQMYEVPIPFLQLMFGSTIKGSCCYFKDESTTLDEAEIAMLDLYCERAQIQDGQSVLDLG
CGQGALTLHVANKYKNCRVTAVTNSVSQKEFIEEKSRKLNLKNVEVMLADITTHEMEDTFDRILVIELFE
HMKNYELLLRKISKWMSNDGLLFIEHICHKTFAYHYEPIDEDDWYTEYIFPAGTMIIPSASFFLYFQDDL
SVVNQWTLSGKHFARTKEQWLKRLDANVDEVMKIMESFSGTKEGAVKWTNYWRGFCLSGMEMYGYNNGEE
WMASHVLFKK
>CMANMT4
MASGKVVDLLKRLDSGLVSDEELRRVIRFELERRLKWGYKPTHEQQLAELLNLAHATKQMEIATKIDTLN
STMYEVPNSFLEIQLGSTLKESCLYFKDESTTVDEAEIAMMDLYLERAQIKDGQIILDLGCGLGALAFHI
AQKYTNCNVTSVTNSVKQKEFIEEKCKILNVSNVKVILTDICTLEMEATFDRIFAIGLIEHMKNYELLLR
KFSAWMKQDGLLFIEHLCHKTLGYHNEPIDEDDWYTAYFFPAGTLTFIPSSFLLYFQDDVSVVNHWTLSG
KHFSRSNEEWLKRMDNKIDEVKEIYKAAASETKDDDIMKLIRLWRFLSISAAEMFGYKDGEEWMISQVLF
KKK
>CMANMT5
METGKNNQNMKTTIDDLWNQMMLGIVPDKEIRRLMKIELKKRLDWGYRPTHQQQLSQLLDFAKGLCNYCW
TALRCMKMSAEFDTLDSKVYETPKSFQQIMCGTTIKESSGLFMNESTTLDQAQISMLDLYFDKAKIKDGQ
SILDLGCGHGALILYLAQKYQNCNITGVTNSLSQKEFIVEKCKKLGLSNVEILLADVTKLEMEDMFDRVF
VIGLIEHMKNFELFLRKISEWMKPDGLLFLEHYCHKSFAHQWEPIDEEDWFSKYIFPPGTVIIPSASFLL
YFQEDVKVIDHWTLSGNHFARTQEEWLKGIDGHIDEVEKTFESFYGISKEEAVKLINFWRVFCLSGVEMF
GYNNGEEWMISHLLFKKK
>CMANMT6
MGSSEAPSVIGSSKAGEIMGRLMKGEIGDEELSKLVRHQWERRLQWGYKPTHEQQLACNFDFIKSLKDMD
MSGEIDTMNEETYELPSAFLEATFGKTIKQSGCYFKDETTTLDEAEEASHELYCERAQIKDGQTILDIGC
GQGGLVLYIAQKYKNCHVTGLTNSKAQMNYILMQAKKLQLSNVDVISADVTKFDNDKTYDRILVIEAIEH
MKNIQLFLKKLSTWMKKDSLLFVDHISHKKFNHHFEALDEEDWYSGFIFPKGCVTILSSSTLLYFQDDVS
VVDHWVVNGMHMARSMEEWRKKLDKNMEAAREILEPGLGSKEAVNQVITHIRTFCLGGYEQFSFNNGEEW
MITQMLFKKK
>CMUNMT1
VDPVEQKNDKKAELLRKLELGQVPYDEIKRLIKVELDRRIQWGYKPTFEQQTADVLSFAHSLRKLNIATA
VDTLDSEMYEVPISFLKIMFGNTIKGSCCYFKDDSVTLDEAEIAMLDLYCERSQIKDGQRVLDLGCGQGA
LTMHVARKFRNCHVTGITNSVSQKEYIEEQCKINNLMNVEIKLADITTHEMEDRYDRILVIELIEHMKNY
ELLLRKISEWLTPDGLLFIEHICHKTFPYHYEPLDEDDWFTEYIFPAGTMIIPSANFLLYFQDDVTVVNH
WTLSGKHYSRTHESWLKNIDANAEAVKKIMVSFTGSEDAALKQMNYWRGFNLSGMELYKYRNGEEWMASH
VLFKKK
>CMUNMT2
MAAGGGEEFGDRVEQKLNKKGEVAELLRKLELGELPDDEIRRLIKLQLDRRIRWGYKPSHEQQVAYLLDF
ANSLRKLNIATAVDALDSEMYEVPIPFFKFMFGSTVKASCCYFKDDFVTLDEAAIAMLDLYCERSQIEDG
QRVLDVGCGVGALTMHVARKYKNCHVTGVTNSVAQKDYIEEQCKINSLTNVEIKLVDISSHEMEEKYDRI
LAIAFIEHMKNYELLLRKISKWLAPEGLLFIDHVCQKTFPYHYEPIDEEDWYTEYIFPAGTMIIPSANLL
LYFQDDVTITNHWVLSGKHFSRTHEMWLKNLYANAKAMKEIMVSFTGSEEAAAKQMNYWRGFALSGMELF

GYENGEEWMASHLLFKKK
>CMUNMT3
MANMGGFNGEAPSLSNRGEVAELLRKLGLGLIPDEEIRNLMRVQVQRRLQWGYKPTFEQQLAQVVHFAHS
LKQMPISLEAEAMESQVYEIPNSFMKLLHGSSMKASWCYFINDSTTLDEAEIAMLELYCERSQIRDGDRV
LDLGCGFGALSTYIARKYPNCQVTGVTNSTFQKEFIEDQCKKDNLVNVEVILADVTTHEMDREFDRIMAI
GVIEHMKNYKLLLRKISKWMKQDGLLFVDHICHKAFAYHFEPLGAEDWIEEYIFPGGVMTIPSANLLLYF
QDDVYVVNHWMVNGKHYSRTNEEWLKRLDGNANVARAILEGSIGSKEEAMKMLNYWRTFCLYGIELCKFN
NGEEWMSSHVLFKKK
>CMUNMT4
MEVKQSKGDELRSRVAELLERPELGLVPDEEIRRLAKARLEKRLKWGYKATHGEQLSSLLQFVESLPSLN
MASEDDSPKAWLYETPTSFLQLIYGDIIKESGSYYKDESTTLEEAMIHNMNLCCERANIKEGQSVVDLGC
GYGAFILHVAQKYKTCRVTGITSSISQKHYIMEQCKKLNLSNVEVILADVATIKLDATFDRVFAAGMFEH
VNDYKSFLRKITNWMKPDGRLFVEHLCNKTFPYQNKPLDDGDNWGEYVFPSGGLIIPSASLLLYFQEDVS
IVNHWTFSGKHAANKFEELLKRIDAKIDAIKRIFNECYGSKDSIRFINYWRVFLITAAEMFGYNNGEEWM
GVHLLFKKK
>CMUNMT5
MEAKQHESNNNIDEELKNRVNIGEQEERPGFEDEEIRRLAKAQLAKRLKWGYKPTHEQQLSHLLQFLQSL
PSLNMASEDESSKAWLYETPTSFLQLLFGNVIKFSGYYYKHESSTFEESMIHNMDLCCERANIKEGQNVI
DLGCGYGAFVLHVAQKYKSCSVTGITCSITQKHHIMEECKKLNLCNVKVILADVATIELGTAFDRVFAFG
MFEEINDYKLILRKISNWMKPDGLFFVEHLCHKTLAYQNKLIDDQDWYEEYIFPSGGLIVPSASLLLYFQ
DDLSVVYHWTYNGKHGARSFEKMLERTDANIDTIKDMFTEFYGSKEKAIKFINYWRVFFITAAEMFAYND
GEEWMCSQLLFKKK
>CMUNMT6
MTSEALKATKAEVLEKLGLGLIPDEEAKRLIRDQLERRVQWINTHDCEQRLAHLHNYVQSLRQADLDTTN
GFNPDTYEMPIAFMKLIQGRLMKLSTCYFKNKSTTLEDAEEEMLDLYCERAQIRDGDQILDLGSGYGSLA
IYVARKYPHCHVTAITNVKSQTKFVKEQCKNYKLNNVEVVLGDITKVELNKEFDRVMAIEVFEHIKNYEL
LLKKISKWMKKDGLLFVENMCHKNHSYQMKPLHEDDWIEEIIFPDEIVTIASADLLLYFQKNVSIANHWV
LNGKHISRSSEAWLNRLDDNANAAKAVLKDFLGSEDEAVKWINQFRLSFIHGIMQGEFNNGEEWMIAHFL
FKKK
>CMUNMT7
MEALFQVPYNVTVKMLLGSLERALLPDMVVRRLTRLLLAARLRQGYKPSSQLQLSDLLHFAQSLQDMPIA
IRTDKPKEQHYELPTSFFKLVLGKNMKYSCCYFLDKSSSLEDAENAILELYCERAHLKDGHTVLDVGCGW
GSLSLYIAQKYPNCRVTGICNSTTQKACIEERCRELQVNNVEIIVADISTLEMEGTFDRVFSIEMFEHMK
NYKELLKKISKWMTQEGLLFVHYFCHKTFAYHFEDLSDDDWITRYFFTGGTMPSANLLLYFQEDVSILNH
WLVNGKHYAQTSEEWLKRMDCNLASIKPIMESTYGKAEAVKWTVYWRTFFIAVAELFGYNNGEEWMVAHF
LFKKK
>CMUNMT8
MEHKIEDIRKLKSRVEEQLERPELGLVKDEDIKTLAKAKLEKRLKWGYKPTYAEQLSNLLQFAQSLPSLK
MENVDDQGSSKQWLYGVPSEFLQIIYGGIIKMSGSYYEDESTTLEESMIKDMDSCCEKANVKEGHSVLDI
GCGYGSLIIHIAKKYRTCNVTGITNFVEQKQYIMEECKKLNLSNVEVIVGDGTTINLNTTTFDRVFVTGM
LEEINDYKLFLKSVSDWMKPDGLLLVTHFCHKTFAYQNNKALDDEDWHNEYIFPSGNLIVPSASLLLYFQ
EDLSVVSHWATNGTHTGRTCKKLVERIDANIEKIKEIFSEFYGSKEDAIRMINYWRVLCITGAEMYTCKD
GEEWMDVYYLFKKK
>CMUNMT9
SEFLEAVFGKTVKQSMCYFKHESATIDEAEEAAHELYCERAQIKDGQTVLDIGCGQGGLVLYIAQKYKNC
HVTGLTNSKAQVNYLLKQAEKLGLTNVDAILADVTQYESDKTYDRLLMIEAIEHMKNLQLFMKKLSTWMT
KESLLFVDHICHKTFAHFFEAVDEDDWYSGFIFPPGCATILAANSLLYFQDDVSVVDHWVVNGMHMARSV
DIWRKALDKNMEAAKEILLPGLGGSHEAVNGVVTHIRTFCMGGYEQFSMNNGDEWMVAQLLFKKK
>CTRNMT1
MAVGAGDNRDLVEPKNDKKEEVAELLRKLELGHVPDDEIKRLIKVELDRRIQWGYKPTYEQQTADVVNFA

HSLRKMSIATEMDTLDSEMYEVPISFFKLMFGSTIKASCCYFKDDSTTLDEAEIAMLDLYCERSQIKDGQ
RLLDLGCGQGALTMHVGRKYKNCRVTGVTNSVSQKEYIEEQCKINNLTNVDVILADITTHVMDDRYDRIL
VIGLFEHMKNYELLLRKMSEWMTPEGLLFIEHACHKTFAYHYEPLDEDDWFTEYIFPSGTMIIPSANFLL
YFQDDVALVNHWTLSGKHYSHTHEWWLKNIDANAEAVKKIMESFTGSEEAAVKQLNYWRGFNLSGMELFK
YRNGEEWMASHVLFKKK
>CTRNMT2
GLKSSVAELLERPELGLVPDGEIRKLTKTRLAKRLEWGYKATHEDQLSHLLRFIHSLPSLNMASEDDSPK
AWLYETPTSFLQLIYGDIIKESGTYYKDESSTLEEAIIHNMDLCCERARIKEGQSVLDLGCGYGAFTLHV
AQKYKSCSVTGITSSISQKDYIMEQCKKLNLSNVEVILADVATIKMNTTFDRVFALGMFEHINDYKLFLR
RISNWMKHDGLLFVEHLCNKTFAYQNKPLDDGDDWFNEYVFPSAGLIIPSASLLLYFQEDVSIVHHWTFS
GKHAAYKFEELLERIDAKIEAIKEIFIECYGSKEDAIRFINYWRVFLITAAEMFAYRDGEEWMGSHVLFK
KK
>ECANMT1
MVDLKVEKEELLKSMELGLVPDEDIRKHIRSQLEKRLKWGYKPNHEQQLAQLLDVIHSLKKMKISKEYES
FDLRLYEAPFDFHKIQLGTHLKESCSYYKDESTTLDEAEGAMLDLYTQKAKIEDGQSILDLGCGVGAVTL
FVANKYKNCKVTGITSCQWQKDFIENKCKELNLTNVRVIIGDVTAYEMEETFDRIFAIELIEHMKNYELL
LRKISKWMKDDGLLFIEHVCHKILAYPYEPIDEEDWFTEYIFPGGTLTLSSASLLLYFQDDVSVVEHSSL
NGKHYSRSHGEWLKNIDANIDEVKGIMRSITKTEEEAVRLVNFWRIFCMCGIELFGYNNGEEWMVSHILL
KKK
>ECANMT2
MAADLVVKKWNNKKELIDEMELGLVGDEEIRELIRNDLEKRLKWGYKSNHEQQLAQLLHFVHSLRGMKIA
ADEVESFNIKVYEAPFSFNKIQLGSSLKESSCYYKHDETTLDEGEIAMMELYTEKAQIKDGQSVLDLGCG
LGSLTLYVANKYPNCKVTGTTASLWHKDFIESKCKEQELTNVKIVLGDATTHEMEERFDRILAIGLIEHL
KNYGLLLGRISKWLKDDGFLFIQHVCHKTLAYPLVPVDEEDWIGEYIFPGGTLTMPSASLLLYFQDELSV
VDHSTLNGKHFSRTHEEWLKNIDAKIDEVKEILKSVTKTEEEVVRLTNFWRIFCMFGVEMFGYNEGEEWM
LSQILFKKK
>ECANMT3
MASLVEEGSFVNNKESVKERVSELVKRLKNGLVSDEELRKLMRVELEKRLEWGYKSTHEQQLSQLIDLAH
SMKKMEIAMEIDALNSTVYEVPLSFLQIIHGTTIKESCLYFKDESTTVDEAEIAMMDLYLERAQIKDGQS
ILDLGCGLGGFSFHIASKFTGCNITAVTNSVKQKEFIEEKCKTLNVPNIKVILADICTTEIENVFDRIIA
IGLIEHMKNYELLLKKFSKWMTQDGLLFIEHLCHKTFGYHNEPLDEDDWYTTYFFPAGTLTFIPSSFLLY
FQDDVSVVDHWTLNGKHFARSNEEWLKRMDEKMDEVKQIFRSNLKSENEVTKTIGEWRFLSMSAAEMFGY
NNGEEWMVSQLLFKKK
>ECANMT4
MALEQEDSMSVPERNEGVADLIKRMELGLVNDEEIRRLMRIQIENRLKWGYKPTHDQQLAQHLHFINSLK
EMKMATEMDSLDSQVYESPNSFQQIMCGRSMKESAGLFMDDVTTVEEAHIRMMDLYCDKATFEDGQKILD
LGCGHGSVVLHVAQKYKGCQVTGVTNSSAQKQYILEQCKKLDLSNVEIILADVTTLEMEEKFDRVIIIGL
IEHMKNFKLFFQKVSKWMKEGGLLFLENYFHKDFAYHCEKIDEDDWYDGYIFPPGSLLMPSASTLLYFQE
DLTVADHWVLPGTHFAKTFEEFLKKIDLRIEEVREIFEAFYGISKEEAMKLSNYWRNFCISAMEIFNYNN
GQEWMISHLLYTKK
>ECANMT5
MDALIQVPYNATVKVMLSSLERNLLPDVVIRKLTKLLLASRLRLGYKPTSDLQLSDLLQFLHSLEEMPIA
IKTDTAKTQHYELPTSFFNLVLGSHMKYSCCYFTDESKSLEDAEEAMLELYCERAQIKDGHTVLDVGCGW
GSLSLYIAQKYSNCKITGICNSVTQKAHIEEQSRELNLNNVEIIVADISTHEMEASYDRILSIEMFEHMK
NYKELLKKISNWLKQDSLLFVHHFCHKAFAYHFEDTSEDDWITRYFFTGGTMPSANLLLYFQDDVSVVNH
WLVNGKHYAQTSEEWLKRMDKNLNSIKPIMESTYGKESAVKWTVYWRTFFISVAELFGYNNGEEWMVAHF
LFKKK
>ECANMT6
MGSSAGEIMGRLMKGEIEDEELKKLIRHQWDRRIEWGYKPTHEKQLAFNLDFIKGLKEMVMSGEIDTMNK
ETYELPTAFLEAVFGKTVKQSCCYFKDENSTIDEAEEAAHELYCERAQIKDGQTVLDIGCGQGGLVLYIA

EKYKNCHVTGLTNSKAQANYIEQQAEKLELTNVDVIFADVTKFDTDKTYDRILVVETIEHMKNIQLFMKK
LSTWMTEDSLLFVDHISHKTFNHNFEALDEDDWYSGFIFPKGCVTILSSSTLLYFQDDVSALDHWVVNGM
HMARSVEAWRKKLDETIEAAREILEPGLGSKEAVNQVITHIRTFCIGGYEQFSYNNGEEWMITQILFKKK
>ECATNMT
MGSSAGEIMGRLMKGEIEDEELKKLIRHQWDRRIEWGYKPTHEKQLAFNLDFIKGLKEMVMSGEIDTMNK
ETYELPTAFLEAVFGKTVKQSCCYFKDENSTIDEAEEAAHELYCERAQIKDGQTVLDIGCGQGGLVLYIA
EKYKNCHVTGLTNSKAQANYIEQQAEKLELTNVDVIFADVTKFDTDKTYDRILVVETIEHMKNIQLFMKK
LSTWMTEDSLLFVDHISHKTFNHNFEALDEDDWYSGFIFPKGCVTILSSSTLLYFQDDVSALDHWVVNGM
HMARSVEAWRKKLDETIEAAREILEPGLGSKEAVNQVITHIRTFCIGGYEQFSYNNGEEWMITQILFKKK
>GFLNMT1
MDLMATSKQVKKKEELLKNMELGLVPDEEIRRLIRIELEKRLKWGYKPTHQQQLAQLLDLVHSLKKMKIA
TEMESLDLKLYEAPFSFVQIKHGSTIKESSSYFKDESMTLDEAEIAMLDLYVERAQIEDGQSVLDLGCGL
GAVTLHVAKKYKNCHVTGLTNSVEQKDFIEGKCKELNLSNVKVILADVTSHEMEDKFDRIFAVELIEHMK
NYELLLRRISKWMKDDGLLFIEHVCHKTFAYHYEPIDEDDWYTEYIFPAGTLTLSSASLLLYFQDDVSVV
NHWTLSGKHYSRSHEEWLKRIDGNMDAVKEIMKSITKTEEEAVKLINFWRIFCMCGAELFGYKDGEEWMM
SHVLFKKKQLLQQC
>GFLNMT2
MGSNEAQVKKESIGEIMGKLMQGEIGDEELSKRIKEIFGKRLQWGYKPTHQQQLAFNLDFIKSLKEMDMS
GEIDTMNEETYELPSAFLEAAFGKTIKQSGCYFKDETTTIDEAEEASHELYCERAQIKDGQTVLDIGCGQ
GGLVLHIAQKYKNCHVTGLTNSKAQKNYILMQAEKLQLSNVDVILADVTKHESDKTYDRILVIETIEHMK
NIQLFMKKLSTWMTEDSLLFVDHICHKTFSHHFEAIDEDDWYSGFIFPKGCVTILSASALLYFQDDVTIL
DHWVVNGMHMARSVDAWRKKLDKNMELAREILLPGLGSKEAVNGVITHIRTFCMGGYEQFSYNNGEEWMV
AQMLFKKK
>GFLNMT3
MDSKEVGIMKKESAEEILGRVMKGQIEDEELRELIKFQFERLLQWGYKPTLQQQLAFNLDFLKSLKELEM
SGETEAMNKETYELPTAFLEAIFGKTIKISACYFKDELMTLDEAEEACHELYCERAQIRDGQTILDIGCG
QGSLILHIAQKYKNCHITGITNSNGQKNYITTQAEKLQLSNVDVILADVTKFDMEKTFDRVSVIGTIEHM
KNMALFMKKVSSWMKEDGLLFVDHVCNKTFSHHYEALDEDDWYSGYKFPKGSVTILWANALLYFQDDVSV
VDHWLLNGMHMARTQVEWGKKLNKNIEAVKEILEAGLGSKEAANQVITHMRTCGIGGYEQFSYNNGEEWM
ISHVLFKKK
>GFLNMT4
MTMEANNAKKEAIENLWEQMMMGLVPDHEITRLMKSELQKRLNWGYKPTHQQQISQLLDFAKSLRRMEMS
LDFDNLELDTKMYETPESFQLIMSGTTLKESSGLFTDETATLDQTQIRMMDLYLEKAKIKDGQSILDLGC
GHGALILHVAQKYRNCNVTGVTNSIAQKEFIFKQCKKLGLSNVEMVLADVTKCEMKATFDHIFVIGLIEH
MKNFELFLRKVSEWMKSDGLLFMEHYCHKSFAYQWEPMDDDDLFSKYVFPPGSAIIPSASFLLYFQDDLT
VVDHWTLSGNHFARTHQEWLKRIDSQSDEIKGIFESFYGISKEEAVKLINYWRVFCLFGVEMFGYNNGEE
WMISHLLFKKK
>GFLNMT5
MGSNETNGELKTKEMVPDLLKRLESGLVADEELRKLIRFELERRLKWGYKPTHEQQLAELLKLAHSTKQM
KIATETDSLNSTMYEVPIPFLQLQFGSAIKESCCYFKDESTTLDEAEVAMMDLYLERTQIKDGQSILDLG
CGLGALAFHIVQKYPNCNVLAITNSVEQKEFIEEKCKIRKVENVKVSLADICTLEMKTTFDRIFAIGLLE
HMKNYQLLLKKFSNWMKQDGLLFIEHLCHKTLAYHYEPLDEDDWYTEYFFPAGTLTIISSSFLLYFQDDV
SIVNHWSLSGKHFSRSNEEWLKRMDMKIDEVKEILEAAFENKDHDITKLINHWRFLAINATEMFGYNNGE
EWMVSQVLFKKK
>GFLNMT6
MGVEITESSSTTMANKKAEVGELLRKLEHGLLPYDEIRRLMRIELGRRLQWGYKPTHEQQLSQVLKLARS
LRTMNIATEIDTLDEQMYEVPIPFLQLMFGSTIKGSCCYFKDDSTTLDEAEIAMLDLYCERAQIKDGQSV
LDLGCGQGALTLHVANKYKNCRVTAVTNSVSQKEFIEEQSRRLNLKNVEVMLADITTHEMEDTYDRILVI
ELFEHMKNYELLLRKISKWLSKDGFLFIEHICHKTFAYHYEPIDEDDWYTEYIFPAGTMIIPSASFFLYF
QDDLTVAKQWTLSGKHFARTKEEWLKRLNANVDEVMKIMESFSGSKEGAVKWTNYWRGFCLSGMEMYGYN

NGEEWMACHVLFKK
>HCANMT1
MAVEAQQPKKAAIVELLKKLELGLVPYDEIKKLIRIQLERRLQWGYKSTYEEQVADVLDLAHALRKMTIA
REVETLDSQLYEVPVEFLKIMNGNTLKGSCCYFKDDSTTLDEAEIEMLDLYCERAQIKDGQSVLDLGCGQ
GALTLHVAQKYKNCRVTAVTNSVSQKEYIEEQSRNQNLSNVEVILADITTHKMDDRFDRILIIELFEHMK
NYELLLRKITEWMAQDGLLFVEHICHKTFAYHYEPLDEDDWFTEYVFPAGTMIIPSASFFLYFQDDVSVV
NHWTLSGKHFSRTNEEWLKRLDANIDTIKPMFESLTGSEEEAMKLINYWRGFCLSGMEMFGYKNGEEWMA
SHLLFNKK
>HCANMT2
MSEIAMEGKQAKKESIVELLKKLELGLVPDNEIKQLIRIELARRLQWGYKSTYEEQVAQVLNLAHSLRHM
KIANEVDTLDSQVYEVSMSFLNIIFGSTMKESCCYFKDDSVTLDDAEVAMLDLYCERAQLQDGQRVLDLG
CGQGTVTLFVAEKYKNCHVTAIPNSVSQKEYIDEQCRIRNLVNVEVILADITTFEMDDTFDRIFVIGLFE
HMKNYELLLKKISKWMTQDGLLFVDHICHKTLAYHYDPLDEDDWFTEYVFPSGTTIIPSASFLLYFQDDV
SVVNHWILSGKHLSRTHEEWLKRLDANIGAIRIMFKSLLGKEEAVLKLVNYWRGFCLSGIELFGYNNGEE
WMVSHVLFKKK
>HCNMT3
MESKQIKKIGSAMEILERLDKGLIPDEELRELVKIQWGRRLKWGYKSTHVQQLEFNLDFVKSLKQMDMSG
KMEVINSESYELPVAFLRAMFGKTLKQSCCYFEDESMTLDEAETAAHELYCERAQIKDGQRVLDIGCGQG
SLIIHIAQKYKNCHVTGITNSNAQKNIIEEECRNLKLSNVEVILADVTQYDTKDTFDRIILIEAVEHMKN
IDLFLKKISKWMKDDGLMFIDCICHKTFGHHFEAIDEDDWYSGYIFPKGSVTLQAASTLLYFQEDVAVVD
QWAVSGKHMARTVEEWLKKLDKNIDVAREILEPSLGSKEAVEKVITHSRTFCIGTSEQFSYNNGDEWMIS
HVLFKKK
>JDINMT1
MSKGVAKLVERMELGLVSDDEVRRLMRILIEKRLKWGYKPTHEEQLTYLTNFIQGLKGMKIAEEIDALDA
KMYEIPIAFMQILCGYSLKFSPGFFEDESTTLDESETIMMDLYCERAQVQDGQSILDLGCGHGGFVLHVA
QKYKNCKVTGVTNSVSETEYIMEQCKKLGLSNVEIIIADVTKFEPEVTYDRVFAIALIEHMKNYELVLQK
LSKWVAQDGFLFVDHHCHKVFPYKYEPIDEDDWYTQYIFPGGTLVLPSASILLYFQEDVSIVNHWTLSGN
HPARGFKEWLKRLDDNMDEIKAIFEPFYGSKEEAMKWITYWRVFCITHSEMYAYNGGEEWMISQVLFKRK
>JDINMT2
MDVGEANKKESKNAELLKKLELGLVPDNEIKKLIGIQLERRLKWGYKPIYEQQIAQVLNLAHSLQNMNIA
DEIETLDEHMYEVPISFLKIMNGSMLKGSSSYFRDDSMTLDEAEIAMLDLYCERAQIKDGQSVLDLGCGQ
GALTLHVAQKYKNCHVTAITNSVSQKEYIEDQCKSCNLSNVEVILADITKHESDETYDRILVIELFEHMK
NYALLLRKISKWISKDGLLFVEHICHKTFAYHYQPLDEDDWFTDFVFPAGTMIIPSASFLLYFQDDVSVV
NHWTLSGKHFSRTNKEWLKRLDAEIDTVKEIFKSSLGTEEAAVKFINYWRGFCFSGMELFGYNNGEEWMT
SHLLLKKK
>JDINMT3
MDSNQPKKNESVIELLERLDGGQVSLHELKKLVLIQLQKRLQWGYKPTFEQQQAFHVDFIKSLKQMDISG
DMERINAEAYELPISFLKTMFGKTLKQSACYFKDESPTLDEAEMVMNNLICERARLKDGQTILDIGCGQG
SLVFHIAKKYKNSLVVGMTNSSEQKNYIEEQCRKLSLSNIKVILADVTKYEMEDRFDRVFVIEAIEHMKN
IQLFLNKVSKWMKEDGILFVEHVCHKVFPEHFEELDNEDWYAEYIFPKGSVTVHAASTLLYFQDDVTVLE
HWIMNGKHMGRTHEKWHKNLVKNIDIAKDVLQLAFGSKEAAEKCINHCLNLFAAMSEQFLYNNGEEWMIS
QIMFKKS
>JDINMT4
MEGLIEMPYNASVKMMLSLLERNLLPDTMVRKLTKLLLAGRLRSCYKPSSELQLSDLLHFVHSLQDMPIA
IKTDEPKSQHYELPTSFFQLVLGQNMKYSCCYYLDKSNTLEDAEKAMLELYCERAQIKDGHTVLDIGCGW
GSLALFIAQKFNNCKITGICNSTTQKGYIEEQCRHRQLQNVEIIVADITTFEMEASFDRIFSIEMFEHMK
NYKELLKKISKWMKQDSLLFVHHFCHKAFAYHFEDTSDDDWITRYFFTGGTMPAANLLLYFQDDVSVVNH
WLVNGKHYSLTSEEWLQRMDQNMETIKPIMVLTYGKDSAVKWTAYWRTFFISVAELFGYNNGEEWLVTHF
LFTKK
>MAQNMT1

MAAVEAETNYNNTTTTCDDTKQQEQHKDELLKKWENGSIPDHEIKQLIRAQLERRLRWGYKPTYEQQIAQ
LLDLAHSLRKMKIADEVDTLDSQMYEVPISFLRIMNGARLKGSCSYFKDDSTTLDEAEIAMLDLYCERAQ
IQDGHSVLDLGCGQGAFTLHVAQKYKNCRVTAITNSVSQKEFIEDEAKKSNLSNVEVLLADITKHETNDT
YDRIVVIELFEHMKNYELLLRKISKWISEDGFLFIEHICHKTFAYHYEPIDEEDWFTTFVFPAGTMLIPS
ASFFLYFQDDVSVVKHWTLSGKHFSRSCETWLKRIDANLDTVKAIFESFLGNKEAATKYLNYWRGFCFSG
MEMFGCKNGEEWMTTHLLLKKK

>MAQNMT2

MKKAEMVKRLEDGLIPDEEIKKLIKVELKRRLELGYKPTHEQQLAQLLHFAHSLRKMEIANEIEALDSHV
YEIPVSFLEIFHGPALKQSSCYFKDESMTLEQAEIAMLDLYCERAEIQDGMSVLDLGCGLGSLALHIAQK
CKNCRVTATTNSMSQKQYIENRCKSHHLSNVEVILADVATQNMDDTFDRIMVVGLFEHMKNYELLLRKMS
KWLSQDGLLFVDHACHKAFVSHFQPLDDDDWFTEYVFPAETFIIPSASFLLYFQDDVSVVNHWILSGKHF
SRSHEEWLKKLDTEVDAVKLLFKSFLESEEAVTKLINYWRGFCFFGMELFAYNNGEEWMSSHVLFKKNE

>MAQNMT3

MELGLVPEKEIRRLMRIQIQKRLEWGYKPTHEEQLAHLTKFIQNIRGMKMADEIDALDAKMYEIPLPFLQ
TICGKTLKFSPGYFKDESTTLDESETLMMDLYCERAQVKDGQSILDLGCGHGGFVLHLAQKYRNSIVTGV
TNSVSETEYIKEQCKKLGLSNVEIIIADVTKFEPEVTYDRVFAIALIEHMKNYALVLNKISKWVAQDGYL
FVEHHCHKVFPYKYEPLDEDDWYTNYIFPGGTLILPSASILLYFQEDVTVLNHWSLSGKHPSRGFIEWLK
RLDENIDVIMGIFEPFYGSKEEATKWINYWRVFCITHSEMYAYGNGEEWMLSQVLLKRK

>MAQNMT4

MDKANERELKRAELFKKLEDDLVTYDEIKQVMRTELAKRLEWGYKPTHQQQLAHLLDFAHALEGMKIANE
VETLASEVYETPLPFXEIVLGPAKKXSSCLFEDESTTLEQAEIAMLDLYFERAQIRXGMSVLDLGCGXGS
VGLHIARKYKNCXVTCITNSISQKQYIENQCKLYNLSNVKIILADIVAHDTDDTFDVVLVIGVIEHMKNY
ALLLNKISKWMAKDGLLFVEHLCHKTFPYHFEPLDEDDWYSNFVFPTGTLTMPSVSFLLYFQADVSILNH
WILSGKNFSRTXEEFLKRIDANVDAIKDGLKPSLGSEGVAKLISYWRGFCLTGMEMFGYNNGEEWMVSQV
LFKNK

>MCANMT1

MAVGSGDMEDKKARVAELLKKLELGLVPYDEIRRLIKVELERRLRWGYKPTYEQQTADVVNFARSLRKMS
IATEIDTLDSQMYEVPISFLKLMFGNTIKGSCCYFKDDTVTLDEAEIAMLDLYCERSQIKDGQRVLDLGC
GQGALTMHVARKFRNCRVTGVTNSVAQKEFIEEQCKINNLPNVEIVLADITTHKMDDRFDRILVIELFEH
MKNYELLLRKISEWMTPDGLLFIEHICHKTFPYHYEPLDEDDWFTEYIFPAGTMIIPSANFFLYFQDDVS
VVNHWTLSGRHYSRTHEAWLKNIDANEDAVKAIMESFTGSEEAAVKLMNYWRGFNLSGMELYKYKNGEEW
MASHVLFKKK

>MCANMT2

MASVDGKALKPKKAEIVELLKKLELGVVPDEEIKHLVRIQLERRLEWGYKSTYEQQISHVLNLVHSLRQM
SIMTEEVETFDSEFYELPISFWKIKLGRAMKQSCCYFKDELTTLDEAEEAMLELYCERAQIEDGQRVLDL
GCGYGSLTLYIARKYPNCHVTGLTNSISQKQFIEEQCKDSNLSNVNIILVDVATHETDETFDRIMVVGLL
EHMKNYELFLRKLLKWMKQDGFLFIDHVCHKALAYIYEPVDEDDWLPECTFPSGTIVIPSAHFLLYFQDD
ASVVNHWIINGKNYRRTSEEWLKRIDANANALKAIMGSVSRKEDEAVRLTNYWRTLLLFHIELFKYNNGE
EWMVAHILFKKK

>MCANMT3

MAEEAKSAIEVVELLKKVELGLIPDDEMKSFIRIELERRLQWGYKPTHHQQLSQVLNFADSLRGMDIAMD
VSSVDWQLYNAPISFLNIIHGRIIKESCCYFEDDSVTVEEAAVAMMDLYCERAKIEDGQRVLDLGCGLGA
LTMHIAGKYNNCRVTGITTSPSQKQFIEDQCLKHNLLNVEIILADVITLEMDQTFDRILAIELFEHMKNY
ELLLRKISKWMTSDGFLFVEHLCHKAFPCHFEPLDETDWITNYAFPAGTITMPSAHFLLYFQDDVSIANH
WTISGNHCSRTHEELLKRMDEKANAVEAIIESFLGSKESARKWISYCRVLHLLSIELFKYNNGEEWMISH
VLFKRKTVHSNM

>MCANMT4

MDAKEAKKESCVAELLERPELGLVPDEEIRKLTRTQLEKRLRWGYKATHEDQLSHLLQFIQCLPSLNMES
EGDKPKSWLYETPTSFLQLIYGDTIKESSTYYKDEWSTLEEAITHILDLCCERAKIQEGQRILDLGCGYG
ALTVHVANKYKSCSVTGVTTSISQKQYITEQCKKLNLSNVEVILEDVATMKMETTFDRIFALGIFEHMND

YKLFLGRISKWMKQDGLLFVEYLCNKTFAYQNKPLDDGDDWYNEYVFPSGGLIIPSSSFLLYFQDDVSIL
NHWTFSGKHSARTFEKLLKRIDSKIDAIQEIFNECYGSKEDAIKYINYWRVFLITGAEMFSYNDGEEWMG
SHFLFKKK
>NDONMT1
MAVMEDGNSDKTVISNKNKAELIKKLELGLVPYDEIKRLMGIEMERRLRWGYKPTHQEQLAQLLNLAHSL
RKMKIADEVETLDSQMYEVPISFLRIMNGSRLKGSCSYFRDDSTTLDEAEVAMLDLYCERAQIKDGQSVL
DLGCGQGALTLHVAQKYKKCRVTAITNSISQKKYIEDQCKYNNLLNVEVILADITKHETNDTFDRIMVIE
LFEHMKNYELLLRKISQWMSQDGFLFIEHICHKTFAYHYEPLDEDDWFTDYVFPAGTMIIPSASFFLYFQ
DDLSVINHWTLSGKHFSRTNEEWLKRLDANVDTVKAIFEESLGSEEAAMKFINYWRGFCLSGITMFGYNN
GEEWMTSHLLLKK
>NDONMT2
SSKLHRPKKEEILEKLDGGLVSEEELKKLVHLQLQKRLQWGYKPSNQQQLAFHLHFIKSLTQMDISGDME
IINSESYELPISFLKTVFGKTLKQSACYFKNESMTIDDAEIAMNDLHCERAMLKDGLRILDIGCGQGSLI
FHIAKRYRNCLVTGITNSFVQKNFIEEQCRNLKLSNIKVILADVTKYEMEDTFDRVLIIEALEHMKNVRL
FLNKISKWMKADGILFVDHVCHKIFPEHFVGLDEEDWYSEYIFPKGSVTIHAASTLLYFQDDVKVMDHWI
VNGKNMARTHEKWLKNLVKNMDVARDILPIALGSKEAAERCINHFRNFHLAMMEQFSYNNGEEWMIS
>NDONMT3
MESVTKVPYNASVRMMLSLLERNLLPDTIVRRLTRLLLAARLRLGYKPSSDLQLSDLLHFLHSLQDMPIA
IKTDKPKAQHYELPTSFFQLVLGKNMKYSCCYFLHKSDTLEDAEKAMLNLYCERAEIKDGHTVLDVGCGW
GSLCLFIAQKYRNCQITGICNSLTQKAYIEEQCRHLQLQNVEIIVADMSTFEMDASFDRILSIEMFEHMK
NYKELLKKISKWMKQDSLLFVHHFCHKAFAYHFEDTSEDDWITRYFFTGGTMPSANLLLYFQEDVSVVNH
WLVNGKHYALTSEEWLKRMDKNLGTIKPIMELTYGRDSAVKWIAYWRTFFISVAELFGYNNGDEWMVTHF
LFRKK
>NSANMT1
MEVERKQAKKANIAELLKKLEQGLVPYDEIKRLMRVELEKRLQWGYKPTYEQQLAHLLQLAHSLRHMSIA
HEVDTLDSQLYEVPIEFLKIINGSRLKGSCCYFENDSTSLDEAEVAMLELYCERAQIKDGQSVLDLGCGQ
GALTLYFAQKYKNCRVTAVTNSVSQKEYIEEESKKGGLTNVEVVLADITTHEMAHKYDRILIIELFEHMK
NYELLLRKISKWMAKDGLLFVEHICHKTFAYHYEPLDEDDWFTEYVFPAGTMIIPSASFFLYFQDDVSVV
NHWTLSGKHFSRTNGEWFNRIDANLDVIKPMFEALTGSKEEAMKLINYWRGFCLSGMEMFGYKDGEEWMA
SHVLFKRK
>NSANMT2
MEATQITKKQGVAELIKRIENGQVPDEEITRMMKIQIQKRLKLGYKSTHEQQLAQLLHFVHSLQKMEMAE
EVDTLDSELYEIPLPFLHIMCGKALKFSPGYFKDESTTLDESEVNMLDLYCERAQIEDGQTILDLGCGHG
SLTLHVAKKYRGCKVTGITNSVSQKDFIMEECKKLNLSNVEIILEDVTKFETGTTYDRIFAVALIEHMKN
YELFLKKVSAWMAQDGLLFVEHHCHKVFAYKYEPIDDDDWYTEYIFPTGTLVMSSSSILLYFQEDVSVVN
HWTLSGKHPSLGFKQWLKRIDDNIDEIKEIFESFYGSKEKATKFITYWRVFCIAHSEMYATNGGEEWMLS
QVLFKRK
>NSANMT3
MGILQSLHEANLIPDVILRKRISSSLSSWLRDACKPTFQQQLSDELNFARSLEKMPIAAVSDKPNSQQYE
APTELFLLAFGKQAKFSCSYFADESTPLDDAEEAMLELYCERAQIKDGHTILDLGCGLGSLCLYIAQRYK
NCKITGLSYSTTQKAYIEQICRDRMLDNVEIIHADISTYEMEATFDRVFSIEMFEHMRNYKALLKKIAKW
MKDDSLLFVEHCCHKSFSYSFEGTNEEKDISLLESHAHEWYGRYFFTGGTFPSANLLLYFQENVSVKDHW
IVNGKHFSKTCNEWLKKVDKNSSAMEPIMRSLYGKDLAIKWTVYWRTYLIFMGELFGYKNGEEWMIAHYL
FKKN
>NSANMT4
MTMIDGLVQAPYNATVRVMLSSLERNLLPDAVVRKLTRLLLSARLRSGYKPSTEEQLSDLLQFAHSLQDM
PIAIKTDEPKAQHYELPTSFFNLVLGKHMKYSCCYFLDNSNTLEEAEKAMLDLYCERAQIKDGHTVLDVG
CGWGSLALFIAQRYSNCKVTGICNSTTQKAYIDEQCRDRKLQNVEIIVADISTYEMDASFDRVFSIEMFE
HMKNYKELLKKISKWMKSDSLLFVHHFCHKSFAYHFEDTSEDDWITRYFFTGGTMPSANLLLYFQEDVSV
VNQWLVNGKHYAQTSEEWLKRMDKELATIKPIMVTTYGKDLALKWTAYWRTFFISVAELFGYNNGEEWMV

VHFLFKKK
>NSANMT5
MGIHDLVQAPQNAITRLLLSFHEANRTPDMVLRRAMRSSLSAWLNECYKPSYQAQISDLLSFAHSLESMP
MSTVADEANSQQYELPTEFFELMLGKYLKYSCNYFVDNSATLDDAEEAMLELYCERAQIKDGHTILDLGC
GWGSLCLYIAQKYKNCKITGISYSKTQKAYVDQLCRDRMLDNVEIVFADICTYETEATFDRIFAVELLED
MRNYKALLKMISKWMKEDGLLFVHHCGHKAFSYYFEETNKETNISLSETNAREWVGRYFFTGGTLPSANT
LLYFQEDVSIVDQWIVSGNHFARTCKEWLKRMDNNLGNIKPMIQRVYGEDSTIKWTAYWRSFLLFLQEMY
GFNNGEEWMITHVLFKKK
>NSANMT6
MGSEQTKKESVAELLERLDRGVVPDEEFIELVKIQLDKRLQWGYKPTAEEQVAFHIGFIDALKKMEISGD
MEKINSECYEVPISYHSLVFGAALKQSACYFKHDSMTIDEAEIAMHDLYCERSQIKDGQKILEIGCGQGG
LLFHIAHKFKNCNVTGVTNSISQKEFIEEKCRKLEVQNIEVILADITKYETEDGFDRILIIGTVEHMKNV
QLFLKKVSKWMKDDGFLFVDHVCHKLFAEQFEALDSDDWYAESLFPKGSVIIHAASTLLYFQDDISVANH
WVLNGKHMAYSHERWLKNLLNNRDAAREVLQLSLGSEEAAEKFINHCRMFHLAMKEQFSYNNGEEWMVSH
LLFGRK
>PBRNMT1
MVKGDQFQTTTMEETKISQENDLWTNMELGLIPDEEVRRLMKIEIEKRIEWGMKPTQHQQLAQLLDFTKS
LRGMKMATELDKLDSKLYETPHSFNQIVNGSTLKESSGLYTDVTTTMDEASIKMMDLYCERANIKDGQTI
LDLGCGPGPLVLHIAKKYSNCKITGVTNAFSQREYILEECKKLSLSNVEIILADVTSLDLETTFDRVFVI
GFIEHMKNFELFLRKISKWMKDDAVLFLEHFCHKSFSYHGEPLSEDDWYAKNFFAPGTLVIPSATCLLYF
QEDLAVIDHWFLSGNHFARTNEEMLKGIDGKIEEIKDIFMSFYGINEAEAVKLINWWRLFCITGAEMFSY
NNGEEWFISQLLFKKK
>PBRNMT2
MCTTMDTTKISQQDDLWKNMELGLISDEEVRRLMKIETEKRIKWGTKPTQQEQLAQLLDFNKSLRGMKMA
TEVHALENHKIYEIPDSFNQIIGGKESAGLFTDEATTTIEEANTKMMDLYCERAGLKDGQTILDIGCGAG
LLVLHLAKKYKNCKITGVTNTSWHKEHILEQCKNLNLSNVEVILADVTTVDIERTFDRVFVIGLIEHMKN
FELFLRKISKWMKDDGLLFLEHLCHKSFSDHWEPLSEDDWYAKNFFPSGTLVIPSATCLLYFQEDVTVKD
HWLLSGNNFARSNEAILKRIDSKIEEVKDIFMSFYGIGEEEAVKLINWWRLLCITANELFKYNNGEEWLI
SQLLFKKKLMTCI
>PBRNMT3
MGSIEEVRKESAEETLGRLLRGEINDEELKKLIKYQLEKRLQWGYKSSHQEQLSFNLDFIKSLKKMEMSG
EIETMNKETYELPSEFLEAVFGKTVKQSMCYFKHESSTIDEAEEAAHELYCERAQIKDGQTVLDIGCGQG
GLVLYIAQKYKKCHVTGLTNSKEQVNYILKQAEKLGLRNVDVMLADVTQYESDKTYDRLLTIELIEHMKN
IQLFMKKLSTWMTEDSLLFVEHVCHKTFSHFFEAIDEDDWYSGFIFPPGSVTILAANSLLYFQDEVSVVD
HWVVNGMHTARSFDAWRKKVDKNMEVAKEILLPGLGGSHEAVNAVVTHIRTFCMGGYEQFSLNNGDEWMV
AHLLFKKK
>PBRNMT4
METLIQVPYNATVRVMLSSLERNLLPDVVIRKLTKLLLASRLRLGYKPSSEIQLSDLIQFAHSLEEMPIA
IKTESAKEQHYELPTSFFKLVLGKYMKYSCCYFSDKTATLEDAEKAMLELYCERAQIKDGHTVLDVGCGW
GSFSLYIAQKYKSCKITGICNSKTQKEHIEEQCRELQLQNVEIIVADISTFEMEGSYDRIVSIEMFEHMK
NYKELLKKISKWMKEDSLLFVHYFCHKAFAYHFEDTSEDDWITRYFFTGGTMPSANLLLYFQGDVSIANH
WLVNGKHYAQTSEEWLKRMDKNISTIKPIMESTYGKDSAVKWTVYWRTFFISVAELFGYNNGEEWMVAHF
LFKKK
>PBRNMT5
MGSIEEVRKESAEETLGRLLRGEINDEELKKLIKFQLEKRLQWGYKSSHQEQLSFNLDFIKSLKKMDISG
EIQTVNNETYELPTEFLEAAFGKSIKSSGCYFKHESATIDEAEEAAHELYCERAQIKDGQTVLDIGCGQG
GLVLYIAQKYKKCHVTGLTNSKAQVNYLLKQAEKLGLTNVDAILADVTQYESDKTYDRLLMIEAIEHMKN
LQLFMKKLSTWMTEESLLFVDHICHKTFAHFFEAVDEDDWYSGFIFPPGCATILAANSLLYFQDDVSVVD
HWVVNGMHMARSVDNWRKALDKNMEAAKEILLPGLGGSHEAVNGVVTHIRTFCMGGYEQFSMNNGDEWMV
AQLLFKKK

>PBRNMT6
LKKRGKILERLVNGDIGDEELKKLIKIRFEKILQWGYKPTLQDQLTSNIDFIKSLKEMEMSGDLETMNSE
TYELPTAFLEAALGNTLKQSACYFEDESMTLDEAEIAAYELNSERAKIEDGQTILDIGCGFGGLVLHIAQ
KYKNCHVTGIANSTAQRNYILSQIEKLKLWNVDIILADVTKFDFVTEKKFDRILVIEAIEHMKNIQLFMK
KISKWMKDEGSFLFVEHLCHTAFNHHFEALDEDDWYTSYVLPKGSVTILSLTALLYFQDDVSVVDHWLLN
GMHMARSQEEWAKKLDKNLDVAKKELKQGLGSEEAANQVITHLRTFFVGGAVQFSFNNGEEWMISQLLFK
KK
>PBRNMT7
MQLMAKEELLRNMELGLIPDQEIRQRIRIELEKRLQWGYKETHEEQLSQLLELVHSLRGMKMATEMENLD
LKLYEAPMEFLKIQHGSNMKQSAGYYTDESTTLDEAEIAMLDLYMERAQIKDGQSVLDLGCGLGAVALFG
ANKFKNCQFTGVTSSMEQKDYIERKCKELKLTNVKVLLADITTYETEERFDRIFAVELIEHMKNYQLLLK
KISEWMKDDGLLFVEHVCHKTLAYHYEPVDAEDWYTNYVFPAGTLTLSSASMLLYFQDDVSVVNQWTLSG
KHYSRSHEEWLKNMDKNIVKFKEIMKSITKTEEEANKLLNFWRIFCMCGAELFGYKNGEEWMLTHLLFKK
KLRTPGK
>PBRTNMT1
MGSIDEVKKESAGETLGRLLKGEIKDEELKKLIKFQFEKRLQWGYKSSHQEQLSFNLDFIKSLKKMEMSG
EIETMNKETYELPSEFLEAVFGKTVKQSMCYFKHESATIDEAEEAAHELYCERAQIKDGQTVLDIGCGQG
GLVLYIARKYKKCHVTGLTNSKAQVNYLLKQAEKLGLTNVDAILADVTQYESDKTYDRLLMIEAIEHMKN
LQLFMKKLSTWMTEESLLFVDHVCHKTFAHFFEAVDEDDWYSGFIFPPGCATILAANSLLYFQDDVSVVD
HWVVNGMHMARSVDIWRKALDKNMEAAKEILLPGLGGSHEAVNGVVTHIRTFCMGGYEQFSMNDGDEWMV
AQLLFKKK
>PBRTNMT2
MGSIEEVKKESAEETLGRLLRGEINDEELKKLIKYQLEKRLQWGYKSSHQEQLSFNLDFINSLKKMGMSG
QVEAFTNEVYELPTECFEAAYGKSMKLSGCYFKHESSTIDEAEEASHELYCERAQIKDGQTVLDIGCGQG
GLVLYVAQKYKNCHVTGLTNSKEQVNYILKQAEKLGLRNVDVILADVTQYESDKTYDRILVIGVVEHMKN
MQLFIKKLSTWMAEDSLLFVDHSCHKTFNHFFEALDEDDWYSGYIFPPGCATFLSADSLLYFQDDVSVVD
HWVVNGMHFARTVDAWRKKLDKNMEAVKEILLPGLGGNHEAVNGVITHIRTCCVGGYVQFSLNDGDEWMN
AQLLFKKK
>PSOCNMT
MQLKAKEELLRNMELGLIPDQEIRQLIRVELEKRLQWGYKETHEEQLSQLLDLVHSLKGMKMATEMENLD
LKLYEAPMEFLKIQHGSNMKQSAGYYTDESTTLDEAEIAMLDLYMERAQIKDGQSVLDLGCGLGAVALFG
ANKFKKCQFTGVTSSVEQKDYIEGKCKELKLTNVKVLLADITTYETEERFDRIFAVELIEHMKNYQLLLK
KISEWMKDDGLLFVEHVCHKTLAYHYEPVDAEDWYTNYIFPAGTLTLSSASMLLYFQDDVSVVNQWTLSG
KHYSRSHEEWLKNMDKNIVEFKEIMRSITKTEKEAIKLLNFWRIFCMCGAELFGYKNGEEWMLTHLLFKK
K
>PSOTNMT
MGSIDEVKKESAGETLGRLLKGEIKDEELKKLIKFQFEKRLQWGYKSSHQEQLSFNLDFIKSLKKMEMSG
EIETMNKETYELPSEFLEAVFGKTVKQSMCYFTHESATIDEAEEAAHELYCERAQIKDGQTVLDIGCGQG
GLVLYIAQKYKNCHVTGLTNSKAQVNYLLKQAEKLGLTNVDAILADVTQYESDKTYDRLLMIEAIEHMKN
LQLFMKKLSTWMTKESLLFVDHVCHKTFAHFFEAVDEDDWYSGFIFPPGCATILAANSLLYFQDDVSVVD
HWVVNGMHMARSVDIWRKALDKNMEAAKEILLPGLGGSHETVNGVVTHIRTFCMGGYEQFSMNNGDEWMV
AQLLFKKK
>SCANMT1
MASDHEVSNKELKKKKEVITELLKRLESGLVSDEELRGLIRFELERRLRWGYKPTHEQQLAQLLNLAHSM
KQMKIATEIDALNSTMYEVPIPFLQIQLGSTLKESCCYFKDESTTVDEAEIAMMDLYLERAQIKDGQSIL
DLGCGLGALAFHIAQKYTNCNITAITNSVRQKEFIEEKCKILNVSNVKVSLADICTLEMEATFDRIFAIG
LIEHMKNYELLLKKFSEWMKQDGLIFIEHLCHKTLAYHYEPLDEDDWYTEYFFPAGTLTLISSSFLLYFQ
DDVSVVDHWTLSGKHFSRSNEEWLKRMDEKIDEVKEIFESVSDSKDDDVTKLINHWRFFCISSAEMFGYN
NGEEWMISQVLFKKK
>SCANMT2

MEMIADLLKRLEAGLVPDDEIRSLIRVELERRLKWGYKSTHQEQLDQLLNLAHSIKKMKIASTEMDGLTS
TMYEVPISLVQIQLGSHLKESCLYFKDETTTVDEAEIAMMDLYLERAQIKDGQSILDLGCGLGSVCFHIA
RKYTSCNITAVTNSVSQKEFIEEKSKTLNVPNVKVLLADITTLEMDDTFDCLFAIGLIEHMENYELLLRK
LSDWMKQDGLLFIDHVCHKTLSYHFEPMDEDDWYTNLLFPAGTLTLVSASFLLYFQDDLSLVDHWSMSGK
HFSRTNKEWLKNIDGKMDKIREIVKSITDSEEEVVKLINHWRMLCINSSEMFGFNDGEEWMNSHVLFKKK
KQI
>SCANMT3
MGGSNEEGKKKINGGLSARAGEIMGRLMKGEIGDDELKKLIRNMWERRLQWGYQPTHHQQLSFNLDFIRS
LKKMDMSGEIETMNEETYELPTAFLEAAFGKTIKQSACYFKDKSTTLDEAEEASHELYCERAQIKDGQTV
LDVGCGQGGLVLHIAQKYKNCRVTGITNSKAQMNYIVMQAEKLQLSNVDVILADVTKFDSDQTYDRILVI
ETIEHMKNIQLFMKKLSTWLKKDSLLFVDHICHKTFSHHFETLDEEDWYSGFIFPKGCVTLPSASALLYF
QDDVSVVDHWVVNGMHMARSVEEWGKKLDKNMEAAREILEPGLGSKEAVNQVITHIRTFCIGGYEQFSFN
NGEEWMVAQMLFKKK
>SCANMT4
MSTVEITETSPPLAKKAEVAELLRNLELGLLPYDEIRRLMRIELGRRLQWGYKPTHEQQLSQVLKLARSL
RTMNIATEIDTLDEQMYEVPIPFLQLMFGSTIKGSCCYFKGDSTTLDEAEIAMLDLYCERAQIKDGQSVL
DLGCGQGALTLHVANKYKNCRVTAVTNSVSQKEFIEEQSRKLNLKNVEVMLADITTHEMEDTFDRILVIE
LFEHMKNYELLLRKISKWMSKDGFLFIEHICHKAFAYHYEPIDEDDWYTEYIFPAGTMIIPSASLFLYFQ
DDLSIANHWTLSGKHFARTKEEWLKRLDANEEEVMKIMEPFSGSKEGAVKWINYWRGFCLSGMEMYGYNN
GEEWMASHVLYKK
>SCANMT5
MGGVADLLKKMELGLVPEEEIRRLMRIIIEKRLEWGYKPTHAEQLDHLTNFIQCLRGMKMADEIDALDAK
MYEIPLPFMQTICGSTLKFSPGYFKDESTTLDESEIHMMDLYCERAEVKDGHSILDLGCGHGGFVLHVAQ
KYKNSIVTGVTNSVAEKEFIMTQCKKLCLSNVEIILADVTKFEPETTYDRVFAIALIEHMKNYELVLEKL
SKWVAQDGFLFVEHHCHKVFPYKYEPLDEDDWYTEYIFPGGTIVLPSASILLYFQKDVSVVNHWSLNGKH
PARGFKEWLKRLDENMDAVKAIFEPFYGSKEEAMKWITYWRVFCITHSEMYAYNNGEEWMLSQVLFKRK
>SCANMT6
MAALIQVPYNATVRLMLTSLEKNLLPDVVIRKLTKLLLASRLRLGYKPTSELQLSDLLQFAQSLEDMPIA
IKTDKPKVQHYELPTSFFKLVLGKYMKYSCCYFSDKSKSLEEAEQAMLELYCERAQIKDGHTVLDVGCGW
GSLSLYIAQKYSNCKITGICNSATQKAHIEEQCRDLQLQNVEIIVADISTFAMEATYDRIVSIEMFEHMK
NYKELLKKISKWMNHDSLLFIHHFCHKAFAYHFEDTNEDDWITRYFFTGGTMPSANLLLYFQEDVSIVNH
WLVNGKHYALTSEEWLKRMDQNLDSIKPIMESTYGKDSALKWTAYWRTFFISVAELFGYNNGEEWMVTHL
LFKKK
>SCANMT7
MDRSSEWISEKRISAKEMEKKAEMIRKLELGLIPDDEIKRLLGIQMENRLRWGYKPTHEQQLAQLLDFAH
SLRKMKIADEIESLESQLYEMPVSFMKIMNGSKLKISSSYFKDDSMTLDEAEVAMLDLYCERAQIKDGQT
ILDLGCGMGAFTLHVAEKYKSCRVTSITNSVSQKEYIEDQCRSNNLLNVEVILADITKYEMYDTYDRIMA
IGLLEHMKNYELLLSKISKWMSQDGFLFIDHICHKTLAYQYEPLNEDDWFTDYIFPPGTFMIPSATFLLY
FQDDVSVVNHWTLSGKHFERTHEEWLKRIDANIDTVKVILEEALGNKEAAMKFINYFRNLCLFGITMFGY
NNGEEWMMSHLLLKK
>SDINMT1
MQLMAKEELLRNMELGLIPDQEIRQRIRIELEKRLRWGYKETHEEQLSQLLELVHSLRGMKMATEMENLD
LKLYEAPMEFLKIQHGSNMKQSAGYYTDESTTLDEAEIAMLDLYMERAQIKDGQSVLDLGCGLGAVALYG
ANKFKNCQFTGVTSSMEQKDYIERKCKELKLTNVKVLLADITTYETEERFDRIFAVELIEHMKNYQLLLK
KISEWMKDDGLLFVEHVCHKTLAYHYEPVDAEDWYTNYVFPAGTLTLSSASMLLYFQEDVSVVNQWTLSG
KHYSRSHEEWLKNMDKNIVKFKEIMKSITKTEEEANKLLNFWRIFCMCGAELFGYKNGEEWMLTHLLFKK
K
>SDINMT2
MGVEITESSNAKKAEVAELLRKLELGLLPYDEIRRLMRIELGRRLQWGYKPTHEEQLAQVIKLARSLRSM
NIATEIDTLDEQMYEVPIPFLQLMFGSTIKGSCCYFKDESTTLDEAEIAMLDLYCDRAQIQDGQSVLDLG

CGQGALTLHVANKYKNCRVTAVTNSVSQKEFIEEKSRKLNLKNVEVMLADITTHEMEDTFDRILVIELFE
HMKNYELLLRKISKWMSNDGLLFIEHICHKTFAYHYEPLDEDDWYTEYIFPAGTMIIPSASFFLYFQDDL
SVVNQWTLSGKHFARTKEQWLKRLDANVDEVMKIMESFSGSKEGAVKWTNYWRGFCLSGMEMYGYNNGEE
WMASHVLFKK
>SDINMT3
MGSNEAPSVNGSSNAGKIMERLMKGEIGDEELSKLVRHMWERRLQWGYKPTHEQQLAFNFDFIKSLKHMD
MSGEIDTMNEETYELPTAFLEAAFGKTIKQSGCYFKDETTTLDEAEEASHELYCERAQIKDGQSVLDIGC
GQGGLVLHIAQKYKNCHVTGLTNSKAQMNYILMQAEKLQLSNVDVISADVTKFDTDKTYDRILVIEAIEH
MKNIQLFMKKLSTWMKEDSLLFVDHISHKTFNHHFEALDEEDWYSGFIFPKGCVTILSSSTLLYFQDDVS
VVDHWVVNGMHMARSVEEWRKKLDKNMEAAREILEPGLGSKEAVNQVITHIRTFCLGGYEQFSFNNGEEW
MITQMLFKKK
>TCONMT1
MAVTKREKVEAILKKLELGLIPDGEIRKMIRVELERRLEWGYKPTHEQQTAEVVNFAQSLRKLSIAMEMD
TLDTEMYEVPISFLKIMFGNTIKGSCCYFKDENTTLDEAEVAMLDLYCERSQIKDGQRVLDLGCGQGALT
MHVARKYKNCYVTGITNSVSQKEYIEEQCRINNLPNVEVILADITTHETEDKFDRILVIELIEHMKNYEL
LLRKFSQWLTPDGFLFIEHICHKTLAYQYDPVDEDDWFTEYVFPSGTMIVPSANFLLYFQDDVTVVNHWT
LSGMNYSRTHEAWLKNIDANAEAVKEIMVSFTGSEEAAVKQMNYWRGFNLSGMELYKYKNGEEWMACHIL
FKK
>TCONMT2
MAPGANKGEIAADLRKKMDMGLIPDEEIRRVIKMELGKRLQWGYKPTYQEQTAQLVDFAHSLRKMSMAIE
IDALDSKMYEVPISFYKFTSGSMIKQSCCYFKDDFMTLDEAEIAMLDLYCERAQIQDGEKVLDLGCGAGA
LVFYVAQKYSNCEVTGVTNSVSQKEFIEEQCRNNNISNVKIILADVTTHETDKKFDRIMAIGLIEHMKNY
ELFIRKISQWMTPNGLLFIEHYCHKVIPYHYEPLDEDDWLTEYIFSVGTLTFPSANLLLYFQDDVSVVSH
WIVNGKHPSRTHEEWLKKIDANVGTMKEIMTRFTGSDEAAMKQVNYWRALNLFGIELFKYNNGEEWMVSH
LLFKKK
>TCONMT3
MEDNNNLLQEEMNVVELLQRPELGLVPDEKIRKLTRLQLQKRLKWGYKPTHEAQLSHLFQFIHSLPSLNM
ESEDENPKSWLYETPTSFLQLLYGDCIKESDTYYKEDTATLEEAVINMLELYCERARITEGLSVLDLGCG
YGALTLHVAQKYKSCKVTGVTSSISQKQYIMEKCKKLNLTNVEIILADVATIEIEAASYDRIFALGIFEH
VNDYKLFLGKLSKWMKQDGLLFVEYLCHKTFPYQNKPLDKGDKWYNEYVFPSGGLIIPSASFILYFQNDV
SVVRQWTQGGQHSARTFEELLKRIDGNIDKIKEIFIESYGSKEDAVRFINYWRVFLITGVEMFSYNDGEE
WMGAHFLFKKKFIMQE
>TFLCNMT
MAVEGKQVAPKKAIIVELLKKLELGLVPDDEIKKLIRIQLGRRLQWGCKSTYEEQIAQLVNLTHSLRQMK
IATEVETLDDQMYEVPIDFLKIMNGSNLKGSCCYFKNDSTTLDEAEIAMLELYCERAQIKDGHSVLDLGC
GQGALTLYVAQKYKNSRVTAVTNSVSQKEFIEEESRKRNLSNVEVLLADITTHKMPDTYDRILVVELFEH
MKNYELLLRKIKEWMAKDGLLFVEHICHKTFAYHYEPIDEDDWFTEYVFPAGTMIIPSASFFLYFQDDVS
VVNHWTLSGKHFSRTNEEWLKRLDANVELIKPMFVTITGQCRQEAMKLINYWRGFCLSGMEMFGYNNGEE
WMASHVLFKKK
>TFLNMT1
MGSNEAQVKKESIGEIMGKLMQGEIGDEELSKRIKEIFGKRLQWGYKPTHQQQLAFNLDFIKSLKEMDMS
GEIDTMNEETYELPSAFLEAAFGKTIKQSGCYFKDETTTIDEAEEASHELYCERAQIKDGQTVLDIGCGQ
GGLVLHIAQKYKNCHVTGLTNSKAQKNYILMQAEKLQLSNVDVILADVTKHESDKTYDRILVIETIEHMK
NIQLFMKKLSTWMTEDSLLFVDHICHKTFSHHFEAIDEDDWYSGFIFPKGCVTILSASALLYFQDDVTIL
DHWVVNGMHMARSVDAWRKKLDKNMELAREILLPGLGSKEAVNGVITHIRTFCMGGYEQFSYNNGEEWMI
SHVLFKKK
>TFLNMT2
MIVGGKQELFKKLELGLIPNDEVKKLMRIQLARRLQWGYKSTYEEQIAQVLDLTHSLRHMNIAMEVDTLD
SNMYEVPLDFLKIMNGSALKMSSCYFKDDSTTLDEAEIAMMDLHCERAQIKDGHSVLDLGCGHGSLTFYV
AQKYKKSHVTAVTNSVSQKEYIEEEARRRNLSNVEVLLADIATHEMADTYDRILVVGLFEHMKNYGLLLK

KISEWMAKDGLLFVQHVCHKTFAFHCEPIDEDDWLSEYAFPAGTMIIPSASFFLYFQDDASVVNHWTLSG
KHFSRTTEEWLKRLDANVKVIIPMFATITGSEEEAVKLLNYWRGLCFFGVELYGYNNGEEWMESHVLFKK
K
>TFLPAVNMT
METKQTKKEAVANLIKRIEHGEVSDEEIRGMMKIQVQKRLKWGYKPTHEQQLAQLVTFAQSLKGMEMAEE
VDTLDAELYEIPLPFLHIMCGKTLKFSPGYFKDESTTLDESEVYMMDLYCERAQIKDGQSILDLGCGHGS
LTLHVAQKYRGCKVTGITNSVSQKEFIMDQCKKLDLSNVEIILEDVTKFETEITYDRIFAVALIEHMKNY
ELFLKKVSTWIAQYGLLFVEHHCHKVFAYQYEPLDEDDWYTEYIFPSGTLVMSSSSILLYFQEDVSVVNH
WTLSGKHPSLGFKQWLKRLDDNIDEVKEIFESFYGSKEKAMKFITYWRVFCIAHSQMYSTNNGEEWMLSQ
VLFKKK
>XSINMT1
MLKKLELGLVPYDDIKQLIRSELARRLQWGYKPTYEEQIAEIQNLTHSLRQMKIATEVDTLDSQLYEIPI
EFLKIMNGSNLKGSCCYFKEDSTTLDEAEIAMLDLYCERAQIQDGQSVLDLGCGQGALTLHVAQKYKNCR
VTAVTNSVSQKEYIEEESRRRNLLNVEVTLGDITTHEMAETYDRILVIELFEHMKNYELLLRKISEWIAK
DGLLFLEHICHKTFAYHYEPLDDDDWFTEYVFPAGTMIIPSASFFLYFQDDVSVVNHWTLSGKHFSRTNE
EWLKRLDANLDVIKPMFETLMGNEEEAVKLINYWRGFCLSGMEMFGYNNGEEWMASHVLFKKK

NOS

>ATHBEN1
MVREEQEEDDGGGERKLLVADETVPSLLDETGLVCVTGGSGFVASWLIMRLLQRGYSVRATVRTNSEGNK
KDISYLTELPFASERLQIFTADLNEPESFKPAIEGCKAVFHVAHPMDPNSNETEETVTKRTVQGLMGILK
SCLDAKTVKRFFYTSSAVTVFYSGGNGGGGGEVDESVWSDVEVFRNQKEKRVSSSYVVSKMAAETAALEF
GGKNGLEVVTLVIPLVVGPFISSSLPSSVFISLAMLFGNYKEKYLFDTYNMVHIDDVARAMIFLLEKPVA
KGRYICSSVEMKIDEVFEFLSTKFPQFQLPSIDLNKYKVEKRMGLSSKKLKSAGFEFKYGAEEIFSGAIR
SCQARGFL
>PSONOS
MHGQKNISERYQKFKEMEGTGKIVCVTGGAGYLASWLIMRLLERGYSVRTTVRSDPKFREDVSHLKALPE
ATEKLQIFEADLENPESFDDAINGCVGVFLVAQGMNFAEEYTLEKIIKTCVEGTLRILQSCLKSKTVKKV
VYTSSADAAMMISNLKAVKEIDETIWSEVDNFISKPEQVIPGLPSYVVSKVLTERACLKFSEEHGLDVVT
ILPPLVVGPFITPHPPPSVSIALSIISGDVSMMLGVRLENAVHIDDVALAHIFVFECEKAKGRHICSSVD
FPMHDLPKFISENYPEFNVPTDLLKDIEEQEPVHLSSDKLLSMGFQFKYDFAEIFGDAIRCAKEKGFL
>AMENOS1
MEGEKGIVCVTGGAGYLASWLIKRLLQHGYTVRTTVRSDPQFKEDISHLKSLPEASKNLQVYEADLDKPK
SFDDVINGCIGVFHVAHPIDFEGNKMSQDKAIKISIEGTLGILKACLKSKTESRTAVKRVVYTSSVAAAV
FISNKKDVEEINENIWTEVETLKKNEFTRNGYLISKTLTERSALKFAEEHDLDLVTLLPSMIVGPFLTSN
LPFSLTISQALVLDNKENCKILKNLNNAVHIDDVASAQIYLFECPEAKGRHICSSVDITIHDIAKLMSIK
YPELQLPTDLLMEIEEEKPVHISSKKLLNLGFKFKYGFEDMYSDAIQCCKDKGFL
>AMENOS2
MAEYCVTGGTGFIASYIVKALLEKGHKVRATVRDPDNVEKVGYLWEMPGAKERLKLVKADLMVDGSFDDA
VNGVDGVFHTASPVLVPYDNDVQATLIDPTIKGTRNVLKSCSKASSVKRVVYTSSCSAVRYRYDVQQVSP
LNESHWSDPQYCKNHNLWYAYAKTLAEEEAWKIAKESGIDLVVVNPSFVVGPLIAPQPTSTLLLILGIVK
GLRGEYPNLTVGFVHIDDVVAAHILAMEETKAAGRLICSSSVAHFSEIIGMLKSKYPSYPYENKCGDQKG
DNNPHSMSSNKIQQLGLPPLKNLNEMFEDCIKSFQNQGFL
>BTHNOS1
MENGGEKGSVCVTGGAGYLASWLIMRLLENGYSVRATARLDPKFNEDVTHLKAFPHASEKLQIFDADLNK
PDSFNDVIEGCVGVFHVAHPIDLEGKRPEASVTKTAVDGALGILKACLQAKTVKKVVYSSSMAAAVISKL
NVQEIDESQWTDVDFCRTLDVPGISYVISKTVTERESIKFAKEHGLQLVTMLPALIVGPFSSPRIPESVA
IALSLFNGDPEKPLPAWNTNLVHVDDVVAAQIFLFESPKAEGRYVCCSTNTTVPELAKFITGKYPELKVR
PELLSDVGSENIVRLSSNKLLGLGFEFKHGLEGIFDGAIQCCKEKNLL

>BTHNOS2
MDVKGKVCVTGASGYMASWLIKRLLLSGYHVCGTVREPGNSKKVAHLWQLEGAKERLQLLKADLMEDGSF
DDAVMGCHGVFHTATPVLTPNMVKSDPKAKILAPAIKGTLNVLRSCKKNPYLKRVVLTSSSTAIRVRDEV
DPNIPLDEMSWSSVERCQRLQIWYALSKVLAEKAAWEFAEEEGIDLVTVLPSFIIGPSLPLDLSLTASDI
LGLLNGVSERFTQLGRMGYVHIDDVALCHILVYEESSAQGRYLCSSVVLDNNDLASFLALRYPSLPIPKR
FEQLDRPSFDFNTSKLKSLGFNFKGIEEMFDDCVASLKEQGHISQT
>BTHNOS3
MPEFCVTGGTGFIAAYLVKALLEKGYNVRATVRDPEDVEKVKFLWEMNGAKERLKLVKADLMVDGSFDDA
IQGVDGVFHAASPVLVPQDDHIQETLIDPIIKGTINVLSSCSKSRTVKRVVLTSSCSSIRYHYDVQNVSP
LNETHWSDPEYCKHYNLWYAYAKTLGEKEAWRLAEEKGINLVVINPSFVVGPLIAPQPTSTLLMILRIIK
GKLGEYPNTALGFVHIDDVVSAHMLAMEESEASGRFICSSKVAHWSDIIKMLKAKYPSYPIETKCAKKKG
DDKLHSMDTSKIEQLGLRAFKTLPQMFDDCIKSFQDKELL
>CCHNOS1
MEGGGEKEKGTVCVTGGAGFLASWIIMRLLQHGYTVRTTFRSDPEFKEDVSHLNALPGASEKLEIFEADL
GKPGSFTEAINGCIGVFHVAHPNDFTGKTTQDQMVKLSVEGTLGILKACLDSKTVKRVVYTSSIAAAGLM
NDAKDVKEMTEQIWTEVDAFRTLNIPGISYAVSKTLTERAAIEFAEENGLDLVTLLPSMVVGPFMGSNLP
TSVSLGLSLVFGDEELLKAIKPSCSSVHVDDVASAEIFLFECPEAKGRYICSAAENTIHDLVKFLTVKYP
ELQLHTHLLKEIGEAKPGHVSSKKLMSMGFKFKYGFEETYDEAIKCCKAKGLI
>CCHNOS2
MGVQRERGIVCVTGGAGFLASWIIKRLLERGYSVRTTIQSNPDFKEDVSHITSLPGASKKLQIYEADLGK
PDSFSEAINGCIGVFHVAHPTDFTGKTSQEEMIRISVEGTLGILRSCLDSKTVKRVIYTSSTVAAGLGTS
IEDTKVINEQMWTNVDAFKSTLNAPGISYAVSKTITERAAIDFAHERGLDLVTLLPSMVIGPFLGSYLPM
SVSFALSLIFGNEELLKHMKPTSVVDIDDVASAQIFLFESSEAKGRYICSAGEKTIHEVARFLQKKYPEL
QMQTEILKEIEEEEPVHTSSKKLTSLGFNFKYGFEEMYDNTIKCCKQKGLL
>CCHNOS3
MGVQLEGGIVCVTGGAGFLASWIIKRLLEQGYSVRTTIQSDPEFKEDVSHITSLPGALEKLKIFEADLGK
PDSFSEAIDGCIGVFHVAHPIDLSGKTSQEQMIKLSVEGTLGILKSCLDSKTVKRVIYTSSTLAAGLGTS
FEDTKEINEQMWSNIDALINSTDNAPGTSYAISKTLTERAAIDFAEEHGLDLVTILPSMIVGPFICPNLP
NSIAYALSFVFGNEELLKYLTPTSVVHVDDVASAQIFLLECSEAKGRYICSVGEKTIHEVTKFVQTKYPE
LKVQTQVLKEIEDEQPVHTSSKKLISMGFKFKYGLEELYDETIQCCKEKGFL
>CCHNOS4
MGGQREREIICVTGGAGFLASWIIKRLLEHGYSVRTTVQSNPEFKEDVSHITSLPRASKKLKIYEADLEK
PDSFFDAINGCTGVFHVAHPTDFTGKTSQEQMIKISVDGTLGILKSCLDSKTVKRVIYTSSTVAAGLGLS
INDTEAINEQMWTDVDTFKNTLNAPGISYAVSKTITERAAIDFAREHGLDLITLLPSMVIGPFLGSNLPM
SVSFALSLIFGNEELLKHMKPTSVVDIDDVASAQIFLFECSEAKGRYICSNGEKTIHEVARFLQKKYPKL
KIQTEILKEIEEEEPVHTSSKKLLSLGFKFKYEFEEMYDETIKCCEENGFL
>CCHNOS5
MGATVIDQENTDDQEMVMRKGPVVVTGAAGYIGSWLVMRLLQHGYTVRATVRDPTNVGKTKHLVELPGAK
ERLTIWKADMDDEGSFDDVVRGCTCFFHLATPMDFESNDPENEVIKPAINGVLNVMRSCKKSTTLKRVVF
TTSAGTVNVEEVQKPSYDETNWSDIDFCRRTQMTGWMYFVSKTLAEKAAWDFAKENNMDLITIIPPLVVG
PFIMNSMPPSMITALALITGNEAHYSILKQIQLIHLDDLCNAHIFLYEHPKAEGRYICSSADPTIYELAK
MLKEKYPEYNIPTKFEGIDESIKRVHFSSKKLTDLGFQFKYTMEDMFDGAIQTCREKNLLPLQS
>CCHNOS6
MSITRKKTVCVTGGTGYIAGVLIKKLLEKGYAVNTTVRNPDDEKKISPLLKLQGKYNDLKIFKADLTIEG
SFDEAIACCHFVFHVATPIHVHSEDPENDMIKPAVQGTLNVLNSVTKAETVKRVVLTSSATTLSINKLNG
VGYVIDEKSWTDVEFLTSEKPPNWGYAAMKTLVEKAAWKFAEENKIDLITIILPLVTGQSLTPEHEPPSM
PFAMALLTGNNDLINVMKDLQMLLGSVAMIHLEDAVECHIFLAENESAYGRYNCCPINTGLVELGKFFEN
RYPQYNVHTDFGDFPAQAKLSISSEKLINAGFKFQHIGLEEIYDESVEYYIALGLLQK
>CMANOS1
MEGEKGTVCVTGGAGFLASWLIMRLLQYGYSVRTTIRSDPKFKEDISHLKARPKAQEKLQIFEADLEKPE

SFDEAIDGCVGVFHVAHPIDFEGKISQDKMIKTSVEGTIGILKSCLKYKTVKRVVYTSSMGAAMFVSNIL
DVKEVDENMWTEVDNFKNIPLPGVPYAMSKTITERAAIEFAEEHGLDLVTLLPGMIVGPFLGPNLPISLS
LALSMVFGNDEYTKIMRQTNVVYIDDIACAQIFLLECPNAKGRHICSALDITIHDVAKFISFKFPELQLP
ISLLKEIQEEKPIHVSSEKLLSLGFKFKYGFEDMYDGAIKCCKEKGFLR
>CMANOS2
MEGGSDEKKGTVCVTGGAGYIGSWLIMRLLERGYSVRATVRLDPGLNRDISHLTNLPGASDKLQIFNADL
KNPQSFDTAIEGCIGVFLVAYPGNMDQIEIDDVANEMLEEVTLELLRACLKSKTVKRVVYTSSAAAVLVN
DKGLMEMDESSWSDVEFCRNVKLRGSSYIITKTLIEQAALKFAEENGLDLVSIIPPMVVGPFICPHLPDS
VSMALSIILGNRHPYFAVMQMVHIDDIVSGHIFLFECPNAKGRYICSSMETSVHDIAKFFTTHYPEFELP
MNLISEMKEEKPIHLSPKKLLSLGFKFKYDFKDMFDGAIQSCKEKGFL
>CMANOS3
MAEYCVTGGTGFIASYLVKALLEKGHRVRATVRDPENVEKVGFLWEFTGAKERLKLVKADLMMEGSFDDA
VNGVDGVFHTASPVNVPYDNDVQATLIDPSIKGTMNVLKSCAKASSIKRVVLTSSCSAVRYRYDVQQVSP
LNESHWSDPEYCKSYNLWYPYSKTLAEEHAWKWAKESGIDLVVVNPSFVVGPLIAPYPTSTLLMILGIIK
GMQGEYPNFRLGFVHIDDVVAAHILAMEESKASGRLICSSSVAHFSEIIEMLKSKYPSYPFENKCCDRKG
DDNPHSMDTSKIRQLGLPAFKTIPEMFEDCVKSFQEKGFL
>ECANOS1
MEGEKKVVCVTGGAGYLASCLIMKLIQRGYYVRATIRSDPKFNEDVSHLKAIPGAQEKLEIFDADLEDPE
SFDNIMEGCVGVFHVAQPIAFGGSKEILEKIIKTSLEGTLGILKACLKSKTVKKVVYTSSKAAALDFKNE
AKEIDENTWTDVETLRKNEVGDGWYPISKTLSENAALDFAEEHGLDLVTILPSMIVGGFLSSTLPLALRL
SLALVLGTEVECRLLAQAGNVVHIEDVVSAHIFLFERPEAKGRYICSSNDITIHDVAKFISVKYPELKLP
TELLEEFGKEKAIHYSSKKLLDLGFKFKYGFEDMYTDAIECCKEKGFL
>GFLNOS1
MEGGGGEKGTVCVTGGAGFLASWLIKRLLQHGYSVRTTVRSDPKFKEDITHLQVLPGAQEKLQIFEADLE
KPDSFDNAIDGCVGVFHVAHPIDFEGKISQDTMIKTSVEGTLGILKSCLKAKTVKRVVYTSSMGAAMFIR
NIKDVKEIDESMWTEVDIFKHFEGYGMSYAVSKTLTERAAIEFAEEHGLDLVTILPGMVVGPFDGPNLPV
SLSMALALILGNEELAKLMRQANVVHIDDIASGQIFLMECPEAKGRHICSATDITIHGVAKLISIKFPEI
QLPIDLLKEIEEEKPIHVSSDKLLSLGFKFKHGFEDMYEGAIRCCKEKGFL
>GFLNOS2
MHIYKNEPSRYTSSQYEKGERRMEGEKGIVCVTGGAGHLASWLIMRLLQHGYFVRTTVRSDPKFKEDISH
LKSLPEAAEKLEVFEADLEKPESFSEAINGCIGVFHVAHPMEYEQKVSQETIIKISVEGTLGILKSCLKS
KTVKRVVYTSSIAAAISMSNDIKEINENVWTEVDIVKNLEGPTVSYAVSKTLTERAALEFAEEHGLDVVT
ILPSIIVGPSISNNLPVSTALALSSILDQEEYKTLKKAPKPPAAVHIDDIVSAEIFLFESPKAKGRYICS
TVDITARDVAKFIATKYPELLLSTDLSKEFKEEEEEKPVHISSDKLLSIGFKFKYGFEDMYDDAIQYCKE
KGFL
>GFLNOS3
MEGGRDDEKGIVCVTGGTGYIGSWLIMRLLERGYTVRATVRLDPGHNRDISHLTSLPGALEKLQIFNADL
QNPQSFNAAIDGCVGVFLVAYPINLDQIEVDDAANGKIVLGTLELLKACLKSKTVKRVVYTSSAATLMVN
DKGLMELDESSWSDVEFCRTVKAYGSSYIIPKTLIEQAALKFSEENGLDLVSIIPPMVVGPFICSHLPGS
ISMAMALILGNRYPRVPITQMVHIDDIVSGHIFLFECPEAKGRYICSSLETSIHDVAKFFTIHYPEFEMP
MNLLSEMKEQKPFHLSPKKLLSLGFKFKYGLKDMFDGAIQSCREKGYL
>GFLNOS4
MGSLSTTSEDQKMMSKGPVVVTGAAGYCGSWLVMRLLQQGYTVRATVRDPTKIEKTRHLLDLPGAEERLT
IWKADLEDEGSFDEVIDGSIYVFHLATPMDFESIDPENEVIKPTIHGVLNVMKSCKKAKTVKRVVFTTSA
GTVNVEEHQKPVYDETSWSDIEFCRRVKMTGWMYFVSKSLAEQAAWDFAKKNNIDLISVIPTLVVGPFIM
NSMPPSMLTALALVTGNTAHYSILKQIQLVHLDDLCNAHIFLAEHPNAKGRYICSSEDPTIFQLANMLKD
RYPEFDIPTKFEGIDESIKPVHFSSKKLKDLGFQFQYTMEDMYDGAIQSCREKKLFPPGLEKDRQNEKKE
VTSKVVKEENEKITSVHAVSHAKPAIMA
>HCANOS1
MEEEKGIVCVTGGAGCVASWLIMRLLQHGYSVRATVRSDPMFKEDTSHLLNLPEAPEKLQIFDADLENPE

SFEAAINGCIGVFHLSHPMDFEGKIPLDTLIKTSIEGTLGILKACLKSKTVKRVVYTSSISAAMCISNMN
ETGQIDESMWSDLDMVRSHNFPAVSYIVSKTLTESAVLEFSKEHGLDVVTILPSAIAGPFLTSYLPVAFS
QSLPLIFGKEVQLINMTNTNLVHIDDVASAQIFLFECPIAEGRHICSTIDLSIHGLAKLLSNKYPELELQ
IPTDDLSKMEETKPLHLSSKKLLNLGFKFKYDLEEMFDGTIQCCKEKGFL
>HCANOS2
MVAEGDSVCVTGAAGFIGSWLVMRLLERGYMVKATVRDPDNIKKVKHLLDLPNAKTSLTLWKADLVDEGS
FDDAIKGCTGVFHVATPMDFESKDPENEVIKPTIEGMLNVMRSCVKAKTVRRLVFTSSAGTVNVHQHPQQ
PKYDENCWTDVEFCRNKKMTGWMYFVSKTLAEKAAWEFAKDNNLDFISIIPTLVVGPFLMSSMPPSLITA
LSPITGNEAHYSILKQIQLVHLDDLSNAHIYLFEHPEANGRYICSSYDSTIIDVANVLRQKFPEYNVPNK
FKDVDENLKAVAFSSKRLIDLGFKYKYSKEDMFVEAIVSCREKGLLPLCSETQVNDKSMECEKREARSEG
GSD
>HCANOS3
MERVCVTGAGGFQASWLVNLLLSKGYMVHGTVRNPDDEKNAHLKELEKASENLKLFKADLLDYDSLFAAI
NGCSGVFHVASPVPSGAVPNPEVELIEPAVTGTSNVLKACSMAKIKKVVYVSSIAAVMLNPNWPKDKLMD
ESCWSDKEFCKTTENWYCLSKTEAESQALDYAKKSGLEVVTVCASIIIGPMLQSKMNASSLLLLRMLKDG
LETLDNKIRMFVDVRHVAEALLLVYKKPEAEGRYICSAFTLTMKDLNEKLKTMYPNYNYPKQIVGVDEAW
DISSEKLLNLGWKYRPLEDTLIDSIKNYQEKGLLHKV
>JDINOS1
MEGKTGTMCVTGGAGYLGSWLVMKLLKRGYSVRATVRLDPEHKKDLSHLKNLPGASEHLKFFNADLNKPD
SFDAAIEGCDGVFHVAHPLDFQGNEADPARISMAVEGTLGILKACVNSKTVKRVVYTSSVAAVVLNTTGL
MEMDENSWTDLELVETIKAPGSSYIVPKTLAEQAAIKFAQEHGLDLITVAPSLIVGPFICPHFPTSVFLA
LAVLMGKTDKFQYISITSMVHIEDIVAAHIFLLECPNAKGRFICSALDTTIHDLAKFLSSKYEDFEMPID
SLKEIEERKPLHLSSKKLLDLGFTYKYGLDEIYDGAIQCCKEKGLM
>JDINOS2
MPKKAAVDQKMDFKGKVCVTGASGYLASWLVKRLLLSGYHVKGTVRDPGNSKKLAHLWQLDGAKERLELV
KADLMEDGSFDDAIMGCCGVFHTASPVLGPESDPKAEILDPAVNGTLNILRSCKRNPGLKRVILTSSSAA
VRVRDDFDPNIPLDETSWSSVEICERFQIWYALSKILAEKAAWEFAKENGIDLVTILPSFLIGPSLPHDL
CSTASDVLGLLTGGPNKFSFHGRMGYIHIDDVALCHILLFEEAKAHGRYLCSSFVMDNNDLASFLAQRYP
YLPITKRFEPLDRPCYEFNTSKMKSLGFKFKGIEEMFDDCVASLKEQGYILQK
>JDINOS3
MSIGAGKVVCVTGASGYIASWLVKLLLERGYTVKASVRDPNDYNKTVHLRALSRANERLQLFKANLLEEG
SFHSVIDGCEGVFHTASPFFHNVTDPQAELIDPAVNGTLNVLGSCAKIPTVKRVVVTSSAAAVVYNGKPR
TPEVVVDETWFSDAELCKNLKLWYVVSKTLAEESAWKFAEKNGIDMVAINPAMVIGPLLQPTLNTSAEAI
LNIIKGGETFPNRTLGWVHVKDVANAHILAFENSSATGRYCLFESVAHYSELVRILRELYPGLKLPEKSE
DDKPFAPTYQVSREKAKGLGIDFIPLEIGLKETVESLKEKKFVSFEV
>MCANOS1
MSSGAKKVVCVTGGSGYIASWLVKLLLERGYTVKASVRDPNDPKKIGHLLALPGANERLKLLKADLLEEG
SFDSVIDGCEGVFHTASPFFHAVTDPQTELIAPAVKGTLNVLSSCAKTPSVKRVVVTSSMAAVLYYGKPR
TPEVVVDETWYSDPEFCKESKLWYVVSKTLAEEAAWKFAKENGLDIVTINPAMVIGSLLQPTLNTSAAAV
LNLINGSETFPNYTFAWVHVKDVATAHILAFETPSASGRYCLVERVAHYSDVVKVLHDLYPNLSLPQKCA
DDKPFVPTYQVSIEKAKGLGIDFIPMEVSLKETVEGLKEKNMISF
>MCANOS2
MEGANERVCVTGAGGFVASWLVKLLLSKGYTVHGTVRDPRDKKNAHLMKLKEGYEKLQLFQADLMNYSSL
CAAITGCNGVFHVATPVPPGSDAVLEAELVEPAIVGTLNVLKACSEAKVKRVVVVSSSAAVEMNPNWPKD
QVMDETCWSDKEYCKQTKNWYFFSKTVAENEALEYSKGTGLDVVTVCPSIVIGPMLQSTVNGSSLVLIKI
LKDGIDRVENKVRMLVDVREVAEALLLTYKKPEAQGRYLCVSYMIRMKDLVEKLRSVYPNYNYPKNFTEV
EEEDKLSSAKLQSLGWKFRPLEETLADSVKSYQEAGILDKD
>NDONOS1
MEGGDKGTVCVTGGAGFLASWLITRLLRHGYSVRATVRSDPKFKEDTTHLMNLPEAQEKLAIFDADLEKP
GTFEPVVEGCIGVFHVAHPMDMEGKTPQETITKISVEGTLGLLNACLKSKTVKRVVYTSSTAAAMCVSNL

KELQEIDENMWSDVEMLKGLSLPGLSYSISKTLTERAAIEFGAKNGLDVVTLLPAAIVGPFHTPNIPIGV
ALALALVFGNELACQLAKQLNIVHIDDVASAQIFLLECPKAVGRHICSSADTTIHGLAKFFSARYPEFQM
PIDLLSKMEEEKPIHLSSKKLLDLGFKFNNGIEKMYDGAIESCKQKGFL
>NDONOS2
MEMEGEKGSGSRVVCVTGGGGYIASWLIMRLLQLGYSVRATARLDPKLKKDLSHLTNLPRASQNLKIFNA
DLNQPDSFTAAIEGCDGLFHVAHPLDLEDCEVDDAKVNMLVEGTLGVLKACVKSKTVKRVVYTSSVATVV
ANKTGLMEMDESIWSDIDFVKAYKFPGSSYVAAKTLSEQAAIKFAEEHGLELVTVISSMVAGPFICPSLP
SSVCMALALIMGKSDQYINLVKANLVHIDDIVAAHIFLLECPKAKGRYICSAIDTTIHELAKYLSFKYPE
FEMPMDSLKGVPEMQPIHVSSKKLMDLGFKFKYGLDEIFDGAIQSCKEKGFM
>NSANOS1
MADEEQPSTKLRACVIGGTGYLASFLIKHLLQKGYHVNSTAKDPGNPETISHLLELQNLGELKIFRADLT
DEGSFDEAVSGCLVVFHVATPVHFDSEDPENDMIKPAIQGTLNVLKACTKAQTVKRVVMTSSAAAVSINN
INNTDPVMDEEWWTDVQYLSSEKPFAWGYPVSKALAEKAAWKYAENNEMDLITVIPSVIAGPSLTPNVPN
SVSLAMSLLTGNESLINGLKGMQITSGSISLLHIEDVVRAHIFVAEKQSATGRYICSAVSTSVPELAKFL
SKRYPQYVVLSERACSCEVVSGGGVL
>NSANOS2
MSSSTTTEGAGKTVCVTGGSGYIASWLIKLLLQRGYTVKATVRDPNDPRQTMHLRELCGAGERLSLFKAD
LLSEGVFDSIVNDCDGVFHTASPCYFEAKDPQAEIVEPAVKGTLNVLRSCTKSPSVKRVIVTSSIAAVLN
NGKLRTPDVVVDETWFSDPEVCKELKLWYPLSKTLAEESAWKFAKDHGIDMVSINPAMVIGPLLQPTLNS
SSSIILNLINGAKTYTNAAYGLVDVRDVANAHILAFEVPSATGRICLAEKVCHFSEMVEILHELYPDIQL
PTKCATDEPFVQPYQLCTEKARSLGINFTPIGVSLKETVESLKAKKFVNL
>NSANOS3
MPEYCVTGGTGFIAAYIVKELLHKGYNVRATVRDPGDAEKVGFLLEMDGAKERLKLVKADLLEDGSFDDA
VQGVDGVFHTASPVLVPYDENIQETLIDPCIKGTVNVLKSCSKASSVKRVVLTSSCSSIRYCDEAQKAPL
NESHWSDPEYCKSYNLWYAYAKTLGEKEAWRLSKELGIDLVVVNPSYVVGPLIAPQPTSTLLMILGIITG
VTDEYPNTMIGFVHIDDVVRAHILAMEESRAAGRLICSSSVVHWSEIIKMLKDKYPSYPMPTKCSDKKGD
DTPHSMDTSKINELGLPAFKTVPQMFDDCIKSFQEKGFP
>SCANOS1
MEMEGEKGSGSRVVCVTGGGGYIASWLIMRLLQLGYSVRATARLDPKLKKDLSHLTNLPRASQNLKIFNA
DLNQPDSFTAAIEGCDGLFHVAHPLDLEDCEVDDAKVNMLVEGTLGVLKACVKSKTVKRVVYTSSVATVV
ANKTGLMEMDESIWSDIDFVKAYKFPGSSYVAAKTLSEQAAIKFAEEHGLELVTVISSMVAGPFICPSLP
SSVCMALALIMGKSDQYINLVKANLVHIDDIVAAHIFLLECPKAKGRYICSAIETTIHELAKYLSIKYPE
FEMPMDSLKGAPEMQPIHVSSKKLMDLGFKFLMVWMKYLMEQFKVAKKKGLCRCLLVLELP
>SCANOS2
MGSTTHDQDDQMMVMIKGPVVVTGAAGYCGSWLVMRLLQQGYTVRATVRDPTKIEKTKHLLDIPGAKERL
TIWKADLEDEGSFDEVIEGSIYVFHLATPMDFESTDPENEVIKPTIDGVLNVMKSCKKAKTVKRVIFTTS
AGTINVEKQQKPIYDETSWSDLEFCRQTKMTGWMYFVSKTLAEQAAWDFAKENNIHFISVIPTLVVGPFI
TNNMPPSMLTALALITRNEAHYSILKQIQFVHLDDLCNAHIFLAEHPKAEGRYICSSEDPTIFQLANMLS
ARYPEFDIPTKFEGIDESIKPVHFSSKKLKDLGFQFKYTMEDMFDGAIQSCREKKLFPSDLEKNNQHFDY
QKEIIDLAAAKEKIQISSVPAPEKPTIMA
>SCANOS3
MDWKVIGKKTVCVTGGTGYVASLLIKNLLEKGTYAVNTTARNPEDQKKVAHLLILQADHSDLKIFRADLT
DEGSFDTAIAGCDIVFHVATPVNFNSQDPENEMINPAIHGTLNVLNACVKSKTVKRVVLTSSAAAVSINN
QNGTGFVMDEGCWTDVEFLASAKPPIWGYPVSKTQAEKAAWKFAEENKIDLITVIPSLMLGPSLTLAVPS
SIFLGMSLLTGNNELINTLKGMQMLSGSISITHVEDVVNAHIFLAEKESASGRYICCNISSSLPELAKLL
SNRYPQYKVPTDFRDFPTKAKLSLSSEKLINEGFSFKYGIEEIYDQTMEYYKSVELLQK
>SCANOS4
MAEEYCVTGGTGFIAAYIVKALLEKGHRVRATVRDPDKVEKVGFLWEFTGAKERLKLVKADLMVEGSFDE
AIDGVDGVFHTASPVLVPYDTDIQATLIDPSIKGTMNVLRSCSKSSSLKRVVLTSSCSAVRYRYDVQQVS
PLNESHWSDPEYCKSYNLWYPYAKTLAEEQAWKLANESGIDLVVVNPSYVVGPLIAPYPTSTLLMILGII

KGVQSEYPNLAVGFVHIDDVVAAHILAMEESKASGRLICSSSVAHFSEIIEMLKSKYPSYPIVNKCNDKK
GDDIPHSMDTSKIRQLGFPAFKTIPEMFEDCIKSFQEKGFL
>SDINOS1
MGSINIDPTMMKKGPVVVTGATGYCGSWLVMRLLQEGYIVRATVRDPSKIEKTKHLLGLPGAKERLTLWK
ADLEDKGSFDEVIEGSIYMFHLATPMDFESTDPENEVIKPTINGVLNVMKSCKKSKTVKRVIFTTSAGTV
NVEEHLKPVYDETSWSDVEFCRRIKMTGWMYFVSKTLAEQAAWDFAKENNIHLISVIPTLVVGPFIMNSM
PPSMITALALITGNKAHYSILKQIQLVHLDDLCNAHIFVATHPKAEGRYICSSEDPTIFQLANMIRDRHP
EFDIPTKFEGIDEAIKPIHFSSKKLKDLGFQFKYTMEDMYDGAIQSCREKKLFPPELEKNKKQYDHQKEV
LVKEKKISTAVPNAVLQEIEKNIN
>SDINOS2
MAEYCVTGGTGFIASYLVKALLEKGHRVRATVRDPENVEKVGFLWEFTGAKERLKLVKADLMMEGSFDDA
VNGVDGVFHTASPVNVPYDNDVQATLIDPSIKGTMNVLKSCAKASSIKRVVLTSSCSAVRYRYDVQQVSP
LNESHWSDPEYCKSYNLWYPYSKTLAEEHAWKWAKESGIDLVVVNPSFVVGPLIAPYPTSTLLMILGIIK
GMRGEYPNFRLGFVHIDDVVAAHILAMEESKASGRLICSSSVAHFSEIIEMLKSKYPSYPFENKCCDRKG
DDNPHSMDTSKIRQLGLPAFKTIPEMFEDCIKSFQEKGFL
>SDINOS3
MAKGTVCVTGASGFLASWLIKRLLLSGYHVVGTVRDPGNDTKLAHLWNLQGAKERLRLVKAELTEEGSFD
DAIKGCEGVFHMASPVLGQPTDPQDILKPAIDGTLNVLRSCKKNPSLRRVVLTSSSSTIRVREKLDPKNP
LDESSWSSVELCSKLKIWYVLSKTMAEKAAWEFCNENGINLVTVSPSFVVGPSLPPALCSTASDILGLLQ
GETERFYWHGRMGYVHIDDVALCHILVYEREEAEGRYLCSSTVVDNDELVSILSARYPTLPIPKRFEQHE
RPYYDFNTNKIKSLGFTFKSIPEMFDDCITSLVAQGHLSIP

OAT

>RSEVS
MAPQMEKVSEELILPSSPTPQSLKCYKISHLDQLLLTCHIPFILFYPNPLDSNLDPAQTSQHLKQSLSKV
LTHFYPLAGRINVNSSVDCNDSGVPFVEARVQAQLSQAIQNVVELEKLDQYLPSAAYPGGKIEVNEDVPL
AVKISFFECGGTAIGVNLSHKIADVLSLATFLNAWTATCRGETEIVLPNFDLAARHFPPVDNTPSPELVP
DENVVMKRFVFDKEKIGALRAQASSASEEKNFSRVQLVVAYIWKHVIDVTRAKYGAKNKFVVVQAVNLRS
RMNPPLPHYAMGNIATLLFAAVDAEWDKDFPDLIGPLRTSLEKTEDDHNHELLKGMTCLYELEPQELLSF
TSWCRLGFYDLDFGWGKPLSACTTTFPKRNAALLMDTRSGDGVEAWLPMAEDEMAMLPVELLSLVDSDFS
K
>AMEOAT1
MSDIMKVEVVSREIIKPLSPTPNHLKTFNLSFLDQLSPPIYVPIILFYPIDCKNDCKSTIESIRGNLKKT
LAHTLTKYYPLAGRIKDGSFVECNDDGVDYVEANVYDINGGISEIIQNPDGDILKKLLPFDPYGVEGYNT
KVLLSIRINVLEENCGGIVIGICISHKIGDGSSLTTFINDWAAAARKNPSEQIKGPQFDLPSLFPLRDLK
GYIPPSAGAEMVEEIVTKKFVFEGSKITELKKKGIVNGGNNDDDDVKKYPSRVEAVSAFIWRRVIGLDQA
KKQGGDSNNEAPVTVYGSLHAVNMRSRMNPPLPSNSFGNMYTTTMAFSIINNHADEEEKLGHYQLPNLVE
KVKESIKKIDSEYVKKVQTTDELLNSMKHLATGHQMVMLSFSSWCRFPIYEADFGWGKPIWVTTSTFPFK
NVVVFMDTKSGDGIEAWVNMTKDDMAAFERDQELLQYVS
>BTHOAT1
MKMKVQVISRESIKPSSPTPPYLKTFNLCFLDQIAPPFYIPIFLFYADNSPNDIENAGLHCRQKRSLSET
LTSFYPLAGRINGNTSVDCNDEGANYVEARVSGLLSEVIVQPNVADVLKMFSPCDEPHFDDADTAHGPKF
GSKVLLAIQVNVFDCGGIMICVCLSHKISDAYSMSKFINCWAAKTHNCKEEILPISELSSIFPQRDLLGY
IPSAFIHRGKGKIVTKRFVFDASKIASLRATGANSSHVSYPTRIEAVSALIWRRFMESTRAKNGLLKEYV
AIHAVNFRRRMDPPLKVEGLENTLGNVFGSTFTASQDLDGKKEPHILVSKLRDAISKIDGKYLRRILCGN
AYLESFLGGLAGVAKGETGLAIFSSWCRFPWYETDFGWGKPTWVSISGFPCRDLIILMDTRTGDGIEAWV
NMLDEDMAEFEQDEKLLEYVSTTPSA
>CCHOAT1
MEGSSPITTASKAGNKLEMADSKSSSVVEVISKETIKPSIPTPEHLKTIKISLLDQLSPPLYVPIILFYT

```
AAAATDDDDKEIYENLEYKSNILKKSLSETLTHFYPLAGRMKDNMVIDCNDEGVDYFEARVSGNMSEFMK
PDAVPQFLPYDVSCNYSGNGKIQLGVQVNKFDCGGIAIGVCISHKTADASSMSAFINGWASTARTGATES
PTFDLVHLYPPSCVQIPSENSFTTQVEGEKVVTKRFVFDASKIANLRAKLGDDDDDKKTITRVQAVTGLI
WKCAMSKWAHTSSSTSSKLSIANLAVNIRWRMDPPLDQLSYGNLLTLATAASTTEGGSLEELVGQLKVAL
SKIDAETIKYIQGEGGFMRLQELLGRVHEMFAKGDEVEVYMFSSWCRFPVYEADFGWGRPTWVSCSSQPY
KNTVILMDTRSGDGIEAWVNLVEDDMTLFQQNQELLEFVSYTDI
>CMAOAT1
SFDSTSLFPPSEQLASQVSYPTQDSTSVDKLVSKRFVFDAAKITSAREKLQSLMHDKYKCHRPTRVEVVS
ALIWKSAVKSAPPGSISTVTHAMNFRKKMDPPLQDASFGNLCVVVTAVLPATTATTNPATKKVSSTSNE
EQVALDELSDFVALLRREIDKVKGDKGCMEKIIQKFIYGHDASVAKDSDVEDKVTALFMTSWCKFGFYEA
DFGWGTPVWVTTVPLIEPKYKNMVFMNDMKCGEGIEVWVNFLEDDMTKFEHHLREILQLF
>CMAOAT2
MIKMDPKIKVVLISNETIKPSSPTPHHLRNFNFSLIDQFSSPIYIPIILFYDQVIDSNIDDDHRSNRLKR
SLSETLTRFYPVAGRINGNVSIDCNDEGVDYFETRIQGKMSDFMVRPELYNHLLPSHIVSNIFATDAQLA
VQVNVFDCGGLAICFCMSHKIADASSMTTFINDWAATTRGSMAESVGPIFESASLFPARDILKTSIEPVP
DTAGVDVVTKRFVFDASMIAALRAKIQGTTVTSVGKYPTRVEAVSGLIWKSTIEMSKVKLESGSSTKPLK
AMVVHHSVNLRKRMDPPLADVSFGNIITLTSAVASSTMIMSQEVELEELVGKLRAAISRINISDYINKML
GCDDDDEEFVRLLKVSNRFAKEVNNDDHVDGEDNTDTDADSNADDDDVYWMSSWCRFPVYESDFGWGKPT
FVCTKQSYKNVVILMDTRCGGGIEAWVSLVEDDMAIFERNPNLLQFVSTIN
>CMAOAT3
MVLKVEIISKETIKPSSPTPIHLRSFKLSFLDQITPPIPFNILFFYPASDGSFDNVKRSDRLKKSLSETL
TYFYPLAGTIGDNITVQCDDNGVQFLETQVNCQLSKVLKHPEAQVMKQFLPDPGMMCNELGKGLQLAIQV
NMFDCGGMVIGMTVLHKIIDAGSLSTFINAWARTARGTDETLGPRFDSASLFPPNEVPGTTFLPMKMNPN
SAEKIVTKKFVFDASNIAALRAKASHSEHVKNPTRVESVSALLWKCAWNAKSKSSVANVIVNFRSRIVPP
LPENSFGNLIGMSTTKITNHEEAELHYLARNLRDAIRKINNDYMKELQNDGSLRSTLEAPHEEFNNDEVE
VYTMTSWCRFPFYEVDFGWGKPIWISTVNLLVKNGFMLMDTRCGSGIEAWVTLDEQKMAEFESDLDRASQ
IHFSQNQ
>CMUOAT1
MEVQIISRENISPSTPVDPHRENSLKLSLLDHLATSNYVTIILFYPSGCGVGNHEDHISKRSDQLKTSLS
ETLTHFYPVAGRIRDGAAVDCDGQGVEFSEAKAGVRLAEFLKRPNIDDLDKLLPCAPYVTNEELPAKALL
GVQANYFDCGGMALGVCFSHKIADGAAMATFIRFWADAANGSSKNALPNPSFDLPSLFPAKDSDGATPSG
RGGVGEGSNIVTRRFVFKGEKVAALRGTEVEHPTRVEAVSAFIWERAIAATRAKAGPERASIAVHAVNLR
GRMVPPLPSNAFGNASRPTITKCVEFGSDTRSDRHVLVKHLRDAMRRVDGDYVRKLQCGEEGFMNSIEEI
QKQFSKGEVVAMNFNSWCRFPFYDANFGWGRPIWVSTTKTPGKNLVILIDSKFGDGIEAWLNMVEEDLVE
LEKDEEFLSFVSNT
>CTROAT1
MSTLIKKNLSSSFWFQVVELGESVAMDQKSSNGNVFELSVKVGEPTLVPPAEDTPKGLYFLSNLDQNIAV
IIRTVYCFKSEEKGNERAGEVLKDALAKVLVHYYPMAGRLTISSEGKLIVHCTGEGGVFVEAEADCKMED
IGDITKPDPVTLGKLVYDIPGAKNILEMPPLVAQITKFKCGGFVLGLCMNHCMFDGLGAMEFVNSWGETA
RGEPLTVPPFLDRSILKARIPPKIEFTHQEFAEIEDISXTSSLYEEEMLYRSFCFDTQKLEQLKKKAMED
NVIAKCTTFEALSAFVWKARCEALKMKPDQQIKLLFAVDGRPRFEPPLPKGYSGNGIVLTNSLCNAGEVL
ENPLSFTVGLVQRAVKMVTDSYMRSAIDYFEVTRARPSLAATLLITTWSRLSFHITDFGWGEPILSGPVT
LPEKEVILFLSHGKERKSINVLLGLPASSMKIFQELMEI
>ECAOAT1
MAVMMDKLVRVISKEIIKPSSSTPSHLSPYKLSLLDQVIYPVYVPVVLFFESNHTNSFESESNYHHLLKK
SLSNVLTRYYPLAGRIKDNIFVECNDLGVDYFEAKVNGNITEFRSADLLNQLIPTLDMASRTPLIIQVNM
FDCGGIAISMCMSHKLFDASSIATFINCWAATARGETEFVIPNFDAHSLFSVRQVSFPVPDTPVELKDGT
IENKLVAKRFVFDAANIASLRARIAKTSNGKYPTRVEAVSALIWKSLMNPEAESMSILNQAVNLRRKMDP
PLTQESFGNIVTLATTTLIPKNSYSLDELVGHLRVSIHKIDEDYVRKLRGDSGEEFIKWVTKEAGLMNED
GKNMYMMSSWCRFPFYESDFGWGKPVWVTYSVGQALAKNGIVLMDTRDGEGIEAWVKLEEKDMTAFECNE
```

NLLEFAYVTK
>GFLOAT1
MTYAALPYPLRPCPFTPLDQFSSPHYVPIILFYAAAAPLGDCPTEGNQVDDIDHRSNRLKRSLSETLTQF
YPVAGRIKDSSSVDCNDEGIDYYETRIHGKMSAFMTRPREHHHLLPCHIASNILAKDAQLAVQVNVFACG
GTAISFCMSHKIADASSMTTFINDWAATNRGSTKTLSPTFKSAFLFPPREILNTLAVPGPDTHGIDNRTI
GDVNKAVVTKRFVFDASMIAALRAKIQNTSTPRVGYFPSRVEAVSALIWKSFMEMSKVKKLKIESSSLRK
PVKCMVVHHTVSLRKRMDPLLADRSFGNMITLATAVASPTMLMAKQVDVEELVRQLRAAISKINGDYIKK
LQSGGDDDEEFLRLVNESGRFAKKVNNGDEVTDHNDDSDDDHEVYWMGSWCRFPLYESDFGWGKPVMVSS
YQSYKNVVIMIDTSCGDGIEAWVSLVEDDMAVFERDAQLLEFASTIC
>GFLOAT2
MKMKVEVVSREMIKPSSPTPHHLRTFNLSFLDQLAPPIYVPLVLFYKSDEYDNDNNSHFPDIIKKSLSAS
LTRFYPLAGRIQDEGSVDCNDEGVDYLETKVTNGELSQIIQHPEVHVLQQFLPCDSYSTNQSTPVTSKVL
LAVQVNVFEDCGGIVIGICISHTLADASSLSTFINDWAACARGATDQIMDPCFELPTLFPKVDLMGYNTL
GSIKKNKQQIVTKRFVFEASKITELKKQSVVETNATNINGDGHRECPTRVEALSAFVWKRFMKLNQAKEE
ASARVYRVTQAVNLRKRMVPQLPTNAFGNMYSPTFALLVDAEGKGDHPYPTLIVKIGEAIKRIDGDHVRK
LQHTDAYFNFYKKQMENAASNNQTSNVMLHFSSWCRFGIYEADFGWGKPTWAATAPLPFKNVVVFMDTRS
GDGIEAWVNMTKEDMDEFECDKEFLKFAS
>HCAOAT1
MDQTQQFHLLRVTFQKILGKMTLNVEVISRETIKPSSPTPHHLKNFKLSHLDQYTPPIGVPIILFYSANH
FEKTKMSDRLKKTLSKTLTDFYPLGGKIKDNNSIDCDDSGVEYLETQVKAQLYQFLNCPEADELNQLIPL
NLNCTGMSSEIQLAIQVNVFECGGIAIAACISHKVADASSLCTFLSNWAANARGANHVVFPQFDSAPLFP
PREVSSLQLSTEGPTHNVVTKRFIFDSSNIAALKAKSANYVENPTRVEAVATFLWRCFMNVASLKPGSIK
SFMVNIAVNLRSRMVPPLPDHSFGNLIMAAMPSSTADSKAELHCLVGQLREAIRRYDGDYMKNLQDGDGV
SMFYDSMRQAIELFAKGEMGFYTITSWCRFPFYEIDFGWGKPIWASSLNMVHKNVVILMDTKYGDGIEAL
VTLDEEDMARFECEEELLAFTSPKNIAYVQELHDFTNPIKMAYEQEVLASTSPNGMAYEKRSLISLG
>JDIOAT1
MSIQVEIMSRESIKPSTPTPPHLRTFKLGLFDLLAGFDRVSILLFYDNKDTKKIEFSDISRRLKTSLSET
LSSFYPIAGRMNGYIAIECNDEGVDYVEARVKAQLEDVIKHRNVDQVLELLSPGDDQEPQLSLKVLLAIQ
VNLFECGGIVISVCLSHKISDAYSMITFINSWAAKTSDSNQLIHPISQQLSSLYPPSGLDLNSAPFQSTI
PNLVTKRFVFDSSNIASLRAEVINNNSSCVPSPTRVELVSALLWRCFMELTRKKNGFLKECAAMHIVDLR
KRMDGKGLENALGNLCAPTFTTSRELASENELYVLVGELRDAIRKMDSEFVTKIRRDGEYIQHLEDEYSK
GISTGKTGLIMFTSWCRFPLYKANFGWGKPVWATGISARNDFKNVVALMDTRSGDGIEALVRMAEEDMAE
FEQDEQLLSFLSSTHCVDC
>MAQOAT1
MIVNIKETTMVRPSEPTPKRSLWNSNVDLVVPRMHTPSVYFYRPNGSSNFFDANVLKEALSKALVPFYPM
GARLKRDEDGRIEIDCDGQGVLFVEAETVSVIDDFGDFAPTLELRQLIPTVDYSGDISSYPLLVLQITHF
KCGGVSLGVGMQHHVADGASGLHFINTWSDMARGLDLTIPPFIDRTLLRARDPPRPAFEQVEYLPPPAMK
TPLSTPTDSTSVAIFKITREQLNILKAKSKEGGNTINYSSYEMLAGHVWRSACKARGLPYDQETKMYIAT
DGRSRLRPALPPGYFGNVIFTTTPIAVSGDLSAKPLTYAASVIHEALGRMDDEYMRSALDYLELQPDLAA
LVRGAHSFRCPNIGITSWSRLPIHDADFGWGRPIFMGPGGIAYEGLAFLLPSPINDGSLSLAISLQSEHM
KVFEKILYEF
>MCAOAT1
MKKMMKYMKVQVVSKETIKPSSPTPPHLRTYNFSLIDQHIPQLLYIPFILFYSPNIHHKDHDDDHHRQLI
NIQTQSLDRLKTSLSETLTLYYPLAGRIKNDSIICCEDEGVEFVHTKVIDSPLLELVNKAEPENLAQLLP
RHDHQRNEKPPLLSIQFNAFECGGMAIGLWFSHKVADSSSLCTFLHNWASIARGSLSSTEILCPRFDCAT
HFPPKQLMSPLQHVKTKGEVVSRRYIFDASMIAALKAKASTTINRNSSSSISPSRVLAVIAFLWRTVIAM
DREKQLGSIRPLNMGIAVSLRSKMELTLPELSFGNLVAIGMTSAFHEQEVVERIGDLFGKFNRDYVKKLQ
SGEEYLKLMESLGELEEQICRGEAERYGVTSWCRFPFYEVDFGWGKPVWANVAAFEDNNFFVLMDTRFGE
GIEVWVSLGEDDMARFQRQLDRELLVLFNSFSSVGSA
>NDOOAT1

MKVEKISRDFIKPSTPTPDHLRTFRLSLLDQLAHPVFVPIILFYSSPTTTLDDDHQLGVSNQLKKSLSET
LTLFYPLAGRIKDKLLVDCSDEGIEYFESRVNGKISDFMKRDELHHLLPYDFQSTLNVIVGNDSHLIVQV
NLFDCGGIAISVSISHKLADAATLTTFINTWASITRQAIPESIVSPTFDSAILYPPREGMSSPFSGAINK
HLTSKVQAGQAEKRVTRRFVFDGTKIATLRARYGNSNDEKKKKKNLPTRVEAVTALIWKTAMAATSSPKP
SIANIIVDMRRRLDPPLPEQSFGNLATTVARVAPSITKSTGEMDLLGPMRESIRKINSDSLKVLQGDGGF
IKFQEFMKMALETNGNELELYRFTSWCRFPVYEADFGWGKPNWVAVASLPFKNTSLLMDDRTGDGIEAWI
CLLEEDMARFERDQELLDFVTLLD
>NSAOAT1
MTPKVSVLSRETIKPSLPTPPHLRTFRLSLLDQYSFPVRVPVILFYFNDGENDHTKISKLKTSLSDILTH
FYPLAGRIVGDRLINCDDSGVQYLEAQANTQLETFLKYPKADELNQLLPLKLHHNDESSETQLQIQVNTF
ECGGIAIAASISHMVADTATLCSFINTWAAKSCGCSKTMPPCFDSVALFPPKEIPSFQLPMGDQTQRVIS
KRFVFHSAKLAALRAKSVSKLEVENPTRVEVVSALVWKCLMNAATRRQGMSFTVNMAVNLRSRMVPPLSD
NSFGNLIIGALPSAERGEALHCLVGKFRKMIKRDYSKLPENNGISIFHDGLRKTMEAAARGKIGMYTITS
WCRFPFYEADFGWGKPIWATTVNVDHKNIVMLMDTKDGDGIEALVTLDERDMALFECQQELLDFAIPNSM
VS
>PBROAT1
MILYSWDYMEALSSVDVEVISKETIKPTTPTPYQLRNFNLSLLDQYCPIVYVPIILFYPAAVTNSSGSKH
HDDLNLLKSSLSETLGQFYPLAGRMKDNIVVDCNDEGIDFIEVKIKSKLCDFMMNPDVRLSLLLPSGVVS
MNFVKEAQVIVQVNIFDCGGAAICLCISHKIADACTMSTFTRYWAATTNTARLGGAIGPPNTNPLFLPSF
DSASLFPPNEQLASQSAMPSVTYPTEDSRRHIVVSKRFVFDAVKLKSVREKLQVLMHDKYRSRKPTRVEV
VSALIWKAAMKSAPSGASSTVNHAVNFRKKMDPPLQDVSFGNLCQVVAALLPATTPETTENSDNKTVNST
SNEVQVALDELNDFVAQLRGEIDKVKGDKSCMERMFLNFMNGYDASETKDSDVEDEVIAFWMTSWCNFGL
YDADFGWGKPIWVTTDPFIEPNKNIIYMIDTKCGEGIEVWVNFLEDDMAKFEHHLSEILELF
>PBROAT2
MILYSWDYMEALSSVDVEVISKETIKPTTPTPYQLRNFNLSLLDQYCPIVYVPIILFYPAAVTNSSGSKH
HDDLNLLKSSLSETLGQFYPLAGRMKDNIVVDCNDEGIDFIEVKIKSKLCDFMMNPDVRLSLLLPSGVVS
MNFVKEAQVIVQVNIFDCGGTAISVSISHKIADAATMSTFTRYWAATTNTARLGGAIGPPNTNPLFLPSF
DSASLFPPNEQLASQSAMPSVTYPTEDSRRHIVVSKRFVFDAVKLKSVREKLQVLMHDKYRSRKPTRVEV
VSALIWKAAMKSAPSGASSTVNHAVNFRKKMDPPLQDVSFGNLCQVVAALLPATTPGASPTVNHAVNLRK
KMDPPLQDESFGNLCKAVTAILPATTTTTTTKNEDNKTINSTSNEVQVAVLHELNDFIAQLRSEIDKVKG
DKDCMEKIIQNFMNGYEYATTKKKNDVEDEVITLWMTSWCNMGLYETDFGWGKPIWVTTDPNIKPNKNII
YMNDTKCGEGIEVWANFLEDDMAKFEHHLSEILELF
>PBROAT3
MILYSWDYMEALSSVDVEVISKETIKPTTPTPYQLRNFNLSLLDQYCPIVYVPIILFYPAAVTNSSGSKH
HDDLNLLKSSLSETLGQFYPLAGRMKDNIVVDCNDEGIDFIEVKIKSKLCDFMMNPDVRLSLLLPSGVVS
MNFVKEAQVIVQVNIFDCGGAAICLCISHKIADACTMSTFTRYWAATTNTARLGGAIGPPNTNPLFLPSF
DSASLFPPNEQLASQSAMPSVTYPTEDSRRHIVVSKRFVFDAVKLKSVRDLLPSFDAATLFPPSEQLVCP
SEVPPTPVSHHNDDSGGEKSVSKRFVLDAVKINSVREKLQGLMHDNYKCTSRPTRVDVVTALVWKAAMKS
APRDGFLPMVNHVMSFRKKMDPPLPDASFGNLCAVVSTAVSPGATATSTNDEVLEEQLAELVAQLRGEKY
KVKGDKGCIEKIFLSFVGGDNALWMASWSKFGFYDADFGWGKPVWVTTDLSVVEPNQNLICMIDTKCGEG
IEVSAKFLENDMAKFEFHLSEILKLF
>PBROAT4
MNQFVILKQPTMSNIIVEVISKETIKPSTPTPHQFKNHSLSLIDQYIVPSFVPLIFFFPAVPGLYDHTAG
NKQHGDNILLLKRSLSETLSHFYPLAGRVKDNIVVDCNDQGIEFIEAKVSGSMSEFLKKPDDHLSGLLPS
KVVCMNYVTNTQVFVQVNSFECHSTAICLCVSHKVADVATITTFIRCWAQTNISVSRTTSIGSKLHPTFD
SASLFPPIKQLVSPTMVTPTELDSTPLEESKSISKIISKRFVFDAKMINSVREKISATMVDKYKSRRLSR
VEIVSALIWKSFVKLATPGSSSVIVRHAVNLRRRIDPPLPDVSFGNILEFTKLVVGSASTTTQGTSKLHE
DLNEFVCQLRESISKMNKGDHDFDMENTENEGRNLWMSSWGSYGSYDIDFGWGKPIWVTTVGPMFAFSDV
FYMNDTRCGQGIEVWGNLVEEDMDNFQRNLSKLLERI
>PBROAT5

MFYFSLQRGLTQHIKSGNVLVNNNQEDMATMSSAAVEVISKEIIKPATPPPYQLTNFNISLFDQYMPPSY
TPVILFYPAAVADSTGGSKLHDDLGGLLKRSLSQTLVHFYPMAGRLKDNMVVDCNDQGVDFYQVKIKAKM
CDFMMKPDEFPLSLLLPADVVSVNFVKEAQVIVQVNMFDCGGTAISLCISHKIADACTMSTFTRNWAATT
NTARLGGAIGPPNTNPLFLPSFDSASLFPPNEQLASQSAMPSVTYPTEDSRRHIVVSKRFVFDAVKLKSV
REKLQVLMHDKYRSRKPTRVEVVSALIWKAAMKSAPSGASSTVNHAVNFRKKMDPPLQDVSFGNLCQVVA
ALLPATTPETTVNSDNKTVNSTSNEVQVALDELNDFVAQLRGEIDKVKGDKGCMERMFLNFMNGYVTKDS
DVEDEVIAFWMTSWCNFGLYDADFGWG
>PSOOAT1
MATMSSAAVEVISKETIKPRNPTPYQLRNYNMSLLDQYSSLVYVPIILFYPAASDANSTGSKHHDDLHLL
KRSLSETLVHFYPMAGRMKDNMTVDCNDEGIDFFEVRIKGRMCDFMMKSDAHLSLLLPSEVASTNFVKEA
QVIVQVNMFDCGGTAICFCISNKIADACTMITFIRSLAGTTNIARRGSSIAAPTTNQNLVPSFDSTSLFP
PSEQLASQVSYPTQDSTSVDKLVSKRFVFDAAKITSAREKLQSLMHDKYKCHRPTRVEVVSALIWKSAVK
SAPPGSISTVTHAMNFRKKMDPPLQDASFGNLCVVVTAVLPATTATTTNPATKKVSSTSNEEQVALDELS
DFVALLRREIDKVKGDKGCMEKIIQKFIYGHDASVAKDSDVEDKVTALFMTSWCKFGFYEADFGWGTPVW
VTTVPLIEPKYKNMVFMNDMKCGEGIEVWVNFLEDDMTKFEHHLREILQLF
>PSOSAT1
MATMYSAAVEVISKETIKPTTPTPSQLKNFNLSLLDQCFPLYYYVPIILFYPATAANSTGSSNHHDDLDL
LKSSLSKTLVHFYPMAGRMIDNILVDCHDQGINFYKVKIRGKMCEFMSQPDVPLSQLLPSEVVSASVPKE
ALVIVQVNMFDCGGTAICSSVSHKIADAATMSTFIRSWASTTKTSRSGGSTAAVTDQKLIPSFDSASLFP
PSERLTSPSGMSEIPFSSTPEDTEDDKTVSKRFVFDFAKITSVREKLQVLMHDNYKSRRPTRVEVVTSLI
WKSVMKSTPAGFLPVVHHAVNLRKKMDPPLQDVSFGNLSVTVSAFLPATTTTTTNAVNKTINSTSSESQV
VLHELHDFIAQMRSEIDKVKGDKGSLEKVIQNFASGHDASIKKINDVEVINFWISSWCRMGLYEIDFGWG
KPIWVTVDPNIKPNKNCFFMNDTKCGEGIEVWASFLEDDMAKFELHLSEILELI
>SCAOAT1
MNSILWATPTPDHLRTFRLSLLDQLAHPVFVPIILFYSSPTTTLDDDHQLGVSNQLKKSLSETLTLFYPL
AGRIKDKLLVDCSDEGIEYFESRVNGKISDFMKRDELHHLLPYDFQSTLNVIVGNDSHLIVQVNLFDCGG
IAISVSISHKLADAATLTTFINTWASITRQAIPESIVSPTFDSAILYPPREGMSSPFSGAINKHLTSKVQ
AGQAEKRVTRRFVFDGTKIATLRARYGNSNDEKKKKKNLPTRVEAVTALIWKTAMAATSSPKPSIANIIV
DMRRRLDPPLPEQSFGNLATTVARVAPSITKSTGEMDLLGPMRESIRKINSDSLKVLQGDGGFIKFQEFM
KMALETNGNELELYRFTSWCRFPVYEADFGWGKPNWVAVASLPFKNTSLLMDDRTGDGIEAWICLLEEDM
ARFERDQELLDFVTLLD
>TCOOAT1
MEIQIISRDKVSPSTPTPDHLKSFKLSYLDQQATSNYVTIILFYPPYSTDEDKNQVEHLKRSDQLKKSLS
ETLTHFYPVAGRIVNGVTLDCNDKGVEFLEARVKNTRLSDFLIRPNIDELSKFLPCDPYVTTNEALCSNS
LLAIQTTYFDCGGIALGFCFSHKIADGASMATFINCWASTAINGSTESIPNLFFDLSSLFPAKDSNGLIA
PSGVGMVDNIVTRRFVFDGKKIDALRGEIIFNNSKKNSKEEYPTRVEAVSAFIWKHAIRAARARAGPLRA
SAALHAVNLRGRMVPASPNHRFGNASRPAITEIVNLESETESHALVKHLRDAIRGMDGDYVRKLQCGEEG
FVKSFEEARKNFSKEELLVLNFNSWCRFPFYEADFGWGKPVWVSTTKTLGRNLIILIDTRCGGGIEAWLN
MLEEDMDELEHDQEFLSFVSF
>TFLOAT1
ELPSLCLLEIFIRNKMTLNVEVISEETIKPSSPTPNHLKTFQLSLVDQYAPPVHVPVLLFYSANEHNKVE
NTAHTNRLKESLSEVLTQFYPLAGRITGHEAIDCSDEGVSFSEARISGELKQVLNNPIPQELDQLLPRID
EFYGLGPKVLLSIQVNIFSCGGMILAVCTSHKLLDGSSMSVFLNNWASLSRGDSKLLHPKFNMTSLFPPK
DIPSPMPPIGSPHKVVSKRFIFDASNIAALRAITKDAIENPTRVEVVSALIWRCAMNAVRMRTKSLRSSA
SVVNFAVNLRGRMDPPLSNDFVGNLMIGKSVESSTDIQQKFHSLVSQLRKGLRSIDSGYVKKLQSDNGLS
ELYDSTIRFFEMDVKGELEAYVITSWCRFPFYEADFGWGKPSWLTSVNLVAKNIIMLMDTNCGNGIEAWV
SLIEEDMASFECEPDIITFTSAKSGLELN
>XSIOAT1
MTLDINVLSQETITPSSSTPNHLRNFKLSLVDQYAPPVHISVILFYPANINDRVKIDERTHRLKRSLSEI
LTHFYPLGGKINNNQFIDCTDEGVNFFETRVNGRQLSQVLNHPIPEELEQLLNKDDFYRLGPKALLSIQA

NIFDCGGMALGVCISHKAADGSSFGLFLNNWAAITREDNGIPQPILGQTSLFPPKDDVFSYVPAGVSSIP
KVVNKIFVFNAGNIAALKAKASHKLYVEHPTRVEAVSALLWRCARNALRTRPGSANSASVATLAMNLRGN
MVPALSNLSFGNLMIGTIATSNTESAPELPCLVGELREAKMKVDVDYVRKLQVDDGVSELWNSFMDLNAR
HIKGELELYTISSWCGFPFYEADFGWGKPMWITSVNLFAPNIFILMDSKCGNGVEACVTLDEEVMARFEC
EPELIAFTSTTRSAA

SALR

>SSCPTCR
MSSNTRVALVTGANKGIGFAIVRDLCRQFAGDVVLTARDVARGQAAVKQLQAEGLSPRFHQLDIIDLQSI
RALCDFLRKEYGGLDVLVNNAAIAFQLDNPTPFHIQAELTMKTNFMGTRNVCTELLPLIKPQGRVVNVSS
TEGVRALNECSPELQQKFKSETITEEELVGLMNKFVEDTKNGVHRKEGWSDSTYGVTKIGVSVLSRIYAR
KLREQRAGDKILLNACCPGWVRTDMGGPKAPKSPEVGAETPVYLALLPSDAEGPHGQFVTDKKVVEWGVP
PESYPWVNA
>PSOSALR
MPETCPNTVTKRRCAVVTGGNKGIGFEICKQLSSNGIMVVLTCRDVTKGHEAVEKLKNSNHENVVFHQLD
VTDPIATMSSLADFIKTHFGKLDILVNNAGVAGFSVDADRFKAMISDIGEDSEELVKIYEKPEAQELMSE
TYELAEECLKINYNGVKSVTEVLIPLLQLSDSPRIVNVSSSTGSLKYVSNETALEILGDGDALTEERIDM
VVNMLLKDFKENLIETNGWPSFGAAYTTSKACLNAYTRVLANKIPKFQVNCVCPGLVKTEMNYGIGNYTA
EEGAEHVVRIALFPDDGPSGFFYDCSELSAF
>AMESALR1
MAEIMSYPETKRCAVVTGANKGIGFEICRQLASNGIMVVLTSRDKDKGFEAVEKLKSSGLSDVIFHQLDV
MNPTSISSLADFIKTHFGKLDILVNNAGIGGVIVDADRFKALNLGAGELNDNPKLKEVMTETFESTEECL
KTNYYGVKAVTEALIPFLQLSNSPIIVNVSSSMGMLKNISNGKALEVLCDVDGLTKERIDEVLNMFLKDF
KEGLLETKGWAARLSAYIITKACVNAYTRILAKEFPTFRINCVCPGYVKTDINFNTGVLSVEEGAKSPVD
LALLPHNGPSGLFFVRSEISSF
>AMESALR2
MATSVNSSTTKRYAVVTGANKGLGLGICKQLAADHSVNIVLTARNEQRGLEAVEKLKESAGLSDDQINFH
QLDVADPASVSSLADFVKTKFGRLDILVNNAGIGGVAVYENGFLDQVSKIKNNGTGEVKWDEILTESYEL
TEQCVQTNYYGAKRMTEALIPLLQLSDSPRIANVSSSMGKLKNLPNEWAKGILNDEQNLTEVRVDEVLNE
YLKDYKEGSIQEKGWPRFMSAYVISKAAMNAYTRILAKKYPTFCINAACPGYVKTDINMNTGKLSVEEGA
KSVVKLALLPNGGPSGLFFFEGNISSFE
>BTHSALR1
MAEANSTRPTKRVAVVTGSNKGIGYEICRQLACHGGVVVVLTARDTKRGLEAVEKLKESGLSDVFFHQLD
VMDSTSIASLAAFIKTQFGKLDILVNNAGIGGATLDSDALKAMRLEGKNAKPNWKEIMTETYEGAVECLK
TNYYGVKQVTEALIPLLQLSNSPRIVNVSSSMGLLKNISHDEAKGVLSDADCLTEERVDNMLNDFLTDFK
EGLLESKGWPIFLSAYTISKASLNAYTRILAKKLPTFRINCVCPGYVKTDMNANTGSLTVEEGALGPVTL
AFIPDDGPSGVFFSGTKEATF
>CMASALR1
MAAEIRSDPETRKRCAVVTGANKGIGFEICRQLASNDVLVVLTSRDTKRGTDAVQSLKDSGVSGVVYHQL
DVTDPTTVASLADFIKTQFGKLDILVNNAGIGGIEVDYDGLIALTRGDGELEDNPKFKEVMTQTFELAEE
CLKTNYYGVKAVTESLIPFLQLSDSARVVNVSSSMGQMKNISNEKAIEILSDVAGLTEERIEELMNEYLK
DFKEDLIETKGWPTKLSAYAISKAALNAYTRILAKKFPTYRINCVCPGFVKTDINYNTGVLTVEEGAASP
VRLALLPDDGPSGLFFFRAEVSDF
>CMASALR2
MAAEIRSDPETRKRCAVVTGANKGIGFEICRQLASNDVLVVLTSRDTKRGTDAVQSLKDSGVSGVVYHQL
DVTDPTTVASLADFIKTQFGKLDILVNNAGIVGVKIDYEGLTNLIKAGEYNNNDKLKKLMTQTFELAEEC
LQTNYYGVKSVTEALIPFLQLSDSARVVNVSSSTGKLKNVSNENAKKVLSDVDDLTEERIDEVLNVFRKD
FKEDMLETNGWPERLAAYTLAKAALNAYTRVVAKKFPTFRINSVCPGVVKTEINNYTGVLPVEEGAKAPV
RLALLPDDGPTGVFFFREEVTDF

>CmaSALR3
MAKTCPNTVVTKRRCAVVTGGNKGIGFEICKQLSSNGIMVVLTCRDVTKGLEAVEKLKNSNHENVVFHQL
DVTDPVTTMSSLADFIKTHFGKLDILVNNAGVAGFSVDADRFKAMISDIGEDSEELVKIYEKPEAQELMT
ETYELAEECLTINYYGVKSVTEVLIPLLQLSDSPRIVNVSSSTGSLKYVSNETALEILGDGDALTEERID
MVVNMLLKDFKENLIETNGWPSFGAAYTTSKACLNAYTRVFAKKIPKFQVNCVCPGLVKTEMNYGIGNYT
AEEGAEHVVRIALFPDDGPSGFFYDCSELSAF
>CMUSALR1
MAETGATKRCAVVTGGNKGLGLEVCRQLASNGVFVVLTARDEKKGSEAVESLKASGVSDVVFHQLDVADP
ASVDSLAHFIKNRFAKLDILVNNAGISGSVPTVDDAFEKEKLPEEPGQRMVYMMQSITGTYESSKECLVT
NYYGTKRVTEALLPLLQLSNSATIVNVSSSAGLLQGIGHKRAIELLDDIDENTDKKVEEVLNEFLEDYKE
GSPEAKGWPSRSLGAYMLSKAAMNAYTRFLAKKFPSFRINCVCPGYVKTDMNFNTGLLTVEEGAEKIVKL
ASLPDDGPTGLFFRNDEVHAF
>CMUSALR2
MEETTPTLAEKRYAVVTGGNKGIGFEICRQLASKGIVVILTARYEKRGIEAVEKLESLGVSNVVFHQLDT
MDPTSIALLANFVETNFRKLDILVNNAGCNGLVMYSEAYTAFIDAGNHVTDDKVGSLKGIVEQTYEKAEE
CIETNYFGTKRVTEALLPLLQLSDSPRIVNISSIYSQLKFIANEKIRAELNNVECLKDERLDEVVQQFLK
DFKEDQLLQNGWPLTLSAYKVSKAAINAYTRILARKFPKFRINSNHPGYVITDITCNRGLLTPEEGAKRP
VMLALLPDNGPSGLYFDQMEVSSF
>CTRSALR1
MAETPVSSAATKRCAVVTGGNKGIGLELCRQLASNGVLVVLTARDEKRGTEAVEKLKGSGLSDVFFHQLD
VTNPASVASLADFIKTHFGKLDILVNNAGISGATADFDALNKENLPEEAGPRLFAMMQALSENFEMTKEC
LETNYHGTKRVTEALLPLLQLSNSARIVNVSSSAGLLQGISHERAKEVFSNIDELTEKNVDELLNEYLKD
YKEGSPETKGWPARSLAAYMLSKAAMNAYTRFLANKFPSFRINSVCPGYVKTDINFNTGLLSVEEGADSL
LRLALLPNDGPSGLFFRNKEVSIF
>ECASALR1
MADTRRRYAIVTGANKGIGLEICKQLASNGIFVVLTSRDENRGIEAVGKLKNSGISDVVFHQLDVMNPSS
ISSLADFIKTQFGKLDILVNNAGISGVTVDADGLKKMEEYDARKENPGFEEIMIETYEMAEECLKTNYYG
VKAITKALIPFLEQSNSARIVNVSSTSGKLKNIPDEKVKEIFSNIDELTEERIDEVLNLFLKDFKEGLLK
TKGWPTWSAYTISKAALNAYTRILARKLPSFRVNCVCPGYVKTDITYGTGLLTVEEGAKDPVMAALLPDD
GPSGVFFFRGKGF
>ECASALR2
MAMETAETKKRFAVVTGANKGIGFEICRQLASNGVFVVLTSRDRNRGMEAVEKLKNSGLSDVVFHQLDVM
NPTTISSLADFITTQFGKLDILVNNAGVGGIIVDVEAFKGMREGHDEHKDKSLKDVMTETYEVVEECLKT
NYYGAKAVTEALIPLLELSNSARIVNVSSSMGQLKNIHNKEAKEILGDAEGLTEEKIDEVLNTFLKDFKK
DSLESKEWPVFSGYTFSKAALNGYTRILAKKFPTFLVNSVCPGYVSTDINFNTGILTVEEGAKSPVRLAL
LPDDGPSGLFFIRAEVSEV
>GFLSALR1
MAEIRSGSERKRCAVVTGANKGCGLEICRQLASNGVLVVLTSRDIKRGTEAVENLKNSGLPLVVFHQLDV
TNPTSIASLADFIKTQFGKLDILVNNAGIIGGIADAEGFRAMAEAYGKPHHKQVMTEVMAETNELSEECL
KTNYYGAKAVTEALLPFLQISDSARIVNIASITGKLENISNEMAKKVLSDIDGLTEEKIDEVLNAYVKDL
KDNLLETKGWPANGCAYRLSKAALNAYTRILAKKFPTFHVNSFCPGHVKSDINCNTGVLTAEEGVRKPVS
LTLLQDDGPSGLFFVHGEVSDF
>GFLSALR2
MAEIRSDAERKRCAVVTGANKGIGFEICRQLASNGFLVILTSRDTKRGTDAVEKLKDSGITDVVFHQLDV
MDPTSIASLAEFIKTQFGKLDILVNNAGIGGVEVQHDELRALKQAHGEVKDFSKLGEIMTNVNELTEECL
KTNYYGVKTLTDALIPFLQLSDSARIVNVSSSLGNLKNIPNEKAKDVLSDVDGLTEQRINELLNEFFKDF
KEDLLETKGWPVRISAYILSKAALNAYTRILARKFPTFRINCVCPGYVKTDITYNTGAFTAEEGAESPVR
LALLPDDGPTGLFFYRAEVADF
>HCASALR1
MEEIVPSNPSSKRYAVVTGGNKGIGLEICRQLASNEILVILTARDEKRGNEAVENLKRSGLSDVIFHQLD

VKDPSSIASLANFIKTHFRKLDILVNNAADGGVIIDGEAFRGLRSGFDTVSDERPELLKGIMEQTNEKAE
ECLKTNYYGTKGVTEALLPFLQLSDSARIVNVTSVYGQLTFISNEKVKSELNNVECLREERLDEILQWFL
KDFKEDKVQVNGWPITVSAYKVSKAAVNAYTRILARKFPNMRINCVHPGYVKTDITCNTGFLTAEEGARP
LVKLALLPDNGPSGHYFNRMDMSTF
>HCASALR2
MEEIVPSNPSSKRYAVVTGGNKGIGLETCRQLASNEIFVILTARDEKRGIEAVENLKRSGLSDVIFHQLD
VKDPSSIASLANFIKTHFRKLDILVNNAADIGVIIDGEAWSGFGDVSGDERRELLRGIMKETNEKAEECF
KTNYYGTKGVTEALLPILQLSDSARIVNVSSIYGLLKIIPNEKVKSELNNVDCLTEERLDEILQGFLKDF
KENKLEANGWPITVSAYKVSKAAVNAYTRILARKFPNMRINCVHPGYVKTDITCNTGFLTAEEGARPLVK
LALLPDNGPSGHYFNRMDMSTF
>JDISALR1
MYAVVTGANKGIGFEICRQLASNGVVVVLTARDEKRGAEAVENLKESGLSNVVFHQLDLMNPTGLSSLAD
FIKAKYGKLDILVNNAAISGSIINTDALRVDSQGQSNLMDTLIQTYELSEECLKTNYYGTKAVTEALLPL
LQLSSSPRIVNVSSRLGQLENIPNTWAKGVLSDVDILTEEKIDEVLNEFLKDLREGSLETKNWSTAFSAY
RLSKAALNAYTRILAKKLPKINMYCVCPGYVKTDMNSHTGILTVEEGAEGPVKLALNPNGGSSGLFFAHH
EVVSF
>MCASALR1
MAETVITSPATKRCAVVTGGNKGIGLELCRQLASNGVLVVLTARDEKKGIEAVENLKGCGLPDVVFHQLD
VTDSASVASLADFIKTHFGKLDILVNNAGIGGFSVDNDALKTTKLSDAVEERAQSFFRAIRETYELAKEC
LETNYYGTKRVTEALVPLLQLSNSATIVNVSSSAGLLKNISNERAKELLSNIDELTEEKVDELLNEFLKD
YKDGLSESKGWPVTGSAYKMSKAAMNTYTRFLASKFPSFRINSVCPSYVKTDINFNTGILTVEEGAGSIL
RLALLPSDGPTGLFFSKMEVSPF
>MCASALR2
MAETISSVAATMKCAVVTGANKGIGFEICRQLASNGVLVVLTARDQNKGLEAVEKLRASGLSDLVFHQLD
VMDSASIAALADFIKTHFGKLDILVNNAGIGGVIVDWDALIALKAEVEQKGEQLNLREVTTDGYELAEEC
LRTNYYGVKELTEALIPMLQLSSSPRVVNVSSSYGQLKIVSNERAKEILSEVDYLTEERVDEVLNGFLKD
FKEGLLETKGWPTVLSAYSVSKAALNAYTRILARKHPTFYINCVCPGHVKTETNRNSGMLSTEEGAESLV
MLALLPDGGPSGRFFNRKELSSF
>NDOSALR1
MAETNNKNPATKRYAVVTGSNKGIGLEICRQLACHGVFVVSTARDPKRGIEAVEKLKESGVSDVVFHQLD
VTDPISIASLASFIKAQFGKLDILVNNAGIMSAAIVDWDALAATLGESKDEKPNYKEMMEQTYELAEECL
KINYYGVKQVTEALIPLLELSDSPRIVNVSSSLGLLKNIPNEEVKKVLSDADSLTEEKVDTLLHAFLNDF
KEDLLEPKGWPVILSAYTVSKAALNAYTRILAKKFPTFRVNSVCPGYVKTDINGNTGTLTVEEGAESAVR
LAFLPNDGPSGVFFDRKEESSF
>NDOSALR2
MAETVSNSGTKRCAVVTGANKGLGLEICRQLASKGVLVVLTARDEKRGVEAVENLRQCGVSDVVFHQLDL
VNPTSIASLAEFIKDQFGKLDILVNNAGISGSVINNDALRAHGQVIISSIHVLDAMVESFESAEECLKAN
YYGTKQVTEELLPLLQLSNSATIVNVSSSIGQLKNITNEWAKEMLSDVDGLTEEKIDEVLNEFVKDYREG
LLESKSWAPALPAYTLSKAAVNAYTRILAKKFPTIRINCVCPGFVKTDLNSNIGTLTVEEGAEGPVGLAL
MLDDRSSGQFFSKKEVVSF
>NDOSALR3
MLLSWFLLFRSSPAFLMPTYLFRCAVVTGANKGIGLEICRQLASNGVLVVLTARDEKRGVEAVENLRQCG
LTDVVFHQLDLLNPTSIASLAEFIKAQFGKLDILVNNAAINGIMINTDPLRVQSDWQTIVREAVVQTYEL
AEECLKANYYGTKGVTEALLPLLKLSNSARIVNVSSRYGQLKNICNKWAEEVLSDVDSLTEGKIDEVLNE
FLKDYREGLLESKSWPAAPSAYIVSKSVINAYTRILAKKFPTISINCVCPGFVKTDLNSNIGTLTVEEGA
EGPVGLALMLDDRSSGQFFSKKEVVSF
>NDOSALR4
MAGISTSSTKRCAVVTGANKGIGLEICRQLPSNGVLVVLTARDEKRGVEAVENLRQCGLSDVVFHQLDLM
NSTSIASLAEFIQTQFGKLDILVNNAAIHGTIIDNDALKAQSHGNAWSALTGAMVQTYELAEECINTNYH
GTKGVTEALLPLLQLSNSARIVNVSSKIGQLENISNEWAKEMLSNVDNLTEEKIDDVLNEFLKDYKEGLL

EIKGWPIAPSAYRISKAAVNAYTRILAKKFPMISINCCTPGFVKTDMSAHIGRLSVEDGAKAVVMIALLP
DGGPSGLFFVQKEVASF
>PBRSALR1
MAETCPNTVTKMRCAVVTGGNKGIGFEICKQLSSSGIMVVLTCRDATKGLEAVEKLKNTNHENVVFHQLD
VTDPITTMSSLEDFIKARFGKLDILVNNAGVAGFSVDADRFKAMISDIGEDSEEIEKIYEKPEAQELMSE
TYELAEECLKINYYGVKSVTEVLLPLLQLSDSPRIVNVSSSTGSLKYASNETALEILGDGDALTEERIDM
VVSMLLKDFKENLIEANRWPSFGVAYTTSKACLNAYTRVLAKKIPKFQVNCVCPGLVKTEMNYGIGNYTA
DEGAKHVVRIALFPDDGPSGFFYDCSELSAF
>PBRSALR2
MASKMRCAVVTGGNKGIGFEICKQLASNGITVVLTSRDIKKGLEAVEKLKICNKNVVFHQLDVVNPITIS
SLADFIKAHFGKLDILVNNAGVSGVSIDVDRFREMTSGIGEGSEETEKLLEQLEKPEMKELLTETYELAE
ECLKTNYYGVKSVTEVLIPLLELSDSPRIVNITSINGSLKNITNETALEILGDVDALTEERIDMVVNMFL
KDFKEDLIETKGWPSYVTAYKISKTCLNAYTRILARKYATFRVNCVCPGFVKSDFNCNIGIFTVEEGAKH
AVTIALLPDGGPSGFFYERAQLSAF
>PBRSALR3
MLSNSETKIRCAVVTGGNKGIGFEICKQLALNGILVVLTSRDIKKGLEAVENLRSCTAKNVVFHQLDVLN
PLTISSLADFIKTRFRKLDILVNNAGVTGFSINDIDRYVAMQQSIIGEKQKEESSFDGLKGELEKQEIND
LFIESFELAEHCLKTNYYGVKSVTEILIPLLQLSDSPRIVNLSSMYGNIKNISNKKAMEVLGDVDLLTEE
RIDEVANMFLKDYKEDALETNGWPGSSSAYKISKACLNAYTRILAKKFSTFCVNSVCPGWVDTDMNYNIG
IFTAEEAAKNAITLALLQDNGRPSGQFFDREVATAF
>PBRSALR4
MLNQKIRCAVVTGGNKGIGFEICRQLASKGITVVLTARDVTKGLKAVEKLNNSFGLANVVFHQLDVMNPI
IISSLSDFIGSQFGKLDILVNNAGVSGVITDVAGFTAVVQALIEKDQNRENANLKQMIKNRKANLKQMIK
ETYESAEECLQTNYYGVKSVTKALIPLLQLSNSPRIVNVSSLAGQLKNVCNEEALGILSDGDGLKEESVD
KTVSMFLEDFKDDLLDIKGWPAYLPAYTLSKASLNAYTRILAREFPTFRINCLCPGYVNSDFNCNTGVVT
VTEGAKRVVDLALVPDDGPSXLYFIDGEVSPY
>PBRSALR5
MCAVVTGGNKGIGFEICKQLASNGIMVVLTSRDIKKGLEAVEKLKRSNGICSQNVFFGQLDVVNPETISS
LADFIKTQFGKLDILVNNAGVSGVSIDVDRFKVLTSGIGKDSEETEKLLEQLQTPEIKELLTETYELAEE
CLNTNYYGVKSVTEILIPLLQLSDSPRIVNISSIVGSLKNITNETALEILGDVDALTEERINMVAKMFLK
DFKQGLIETKGWPNYVTAYKISKACLNAHTRMLAKKFAKFYVNCLCPGLVKSDFNYNTGIFTAEEGAENA
VTLALLPDDGPSGFFYERSELSAF
>PBRSALR6
MDSETKNRCAVVTGGNRGIGFEICRQLASNGISVVLTARDVTKGLEAVEKLKNSFGLSNVVFHQLDLMNL
VTLPSLVDFIGSHFGKLDILVNNAGILGAITDAASFKALVEAVEMDKNEEKPNLKQVITETYESAVECMQ
TNYHGVKSVTKALIPLLQLSESPRIVNVSSALGKLKFISNQMALEILNDGDGLKEESVDEIVNMFLKDFK
EDILEIQGWHAYLPAYTISKICLNAYTRILAREFPTFRINCLCPGYVNSDFNCNTGVVTVTEGAKRVVDL
ALVPDDGPSGLYFIDGEVSPY
>PBRSALR7
MNMAETLLNPEVKRCAVVTGANKGIGFEICRQLASNGIFVVLTSRDRNKGLEAVENLKKSGLSNVTFHQL
DVMNLTSVSSLAEFIQTHFGKLDILVNNAGIGGVTIVDEDQLRVLMLEDEKYLENPKLKQIWTEPYDEAE
KCLQTNYYGVKAVTEAFIPLLELSDSRIIVNVSSAMGMLKNVGNEKAFEVLSNADCLTEERIDVVVNTFL
NDLKEGCLEAKGWPTLLSAYTISKASVNAYTRILAKKFPTFRINCVCPGFVKTDMNFNSGVLTVEEGAKS
PVRLALLPDSGTSGLFFVREEVSSF
>SDISALR1
MASKMRCAVVTGGNKGIGFEICKQLASNGITVVLTSRDIKKGLEAVEKLKICNKNVVFHQLDVVNPITIS
SLADFIKAHFGKLDILVNNAGVSGVSIDVDRFREMTSGIGEGSEETEKLLEQLEKPEMKELLTETYELAE
ECLKTNYYGVKSVTEVLIPLLELSDSPRIVNITSINGSLKNITNETALEILGDVDALTEERIDMVVNMFL
KDFKEDLIETKGWPSYVTAYKISKTCLNAYTRILARKYATFGVNCVCPGFVKSDFNCNIGIFTVEEGAKH
AVTIALLPDGGPSGFFYERAQLSAF

>SDISALR2
MAAEIRSDPETRKRCAVVTGANKGIGFEICRQLASNGISVVLTSRDTKRGTDAFQSLKDSGVSGVVYHQL
DVMDPTSVASLADFIKTQFGKLDILVNNAGIGGIEVDYDGLIALTRGDGELEDNPKFKEVMTQTFELAEE
CLTTNYYGVKAVTESLIPFLQLSDSARVVNVSSSTGKLKNISNEKAIEVFSDVDGLTEGRIDEVLNEYLK
DFKEDLIETKGWPTKLSAYTISKAALNAYTRILAKKFPMFRINCVCPGFVKTDINYNTGVLTVEEGAASP
VRLALLPDDGPSGLFFFRAEVSDF
>TCOSALR1
MAEMSSVFNGSPAAAKRCAVVTGGNKGIGFEVCRQLASKGVLVVLTARDEKRGVEALQKLGGSGNADVAF
HQLDVTNSATIASLVDFIKTQFGKLDILVNNAGISGAIINYDLLKLEEARDEEVGRQLEMLLKAHEETSE
LVEECVKTNYYGTKTVTEALLPLLQLSNSARIVNVSSSLGLLQQISNERAKEVFNNIDEHTEERIDEVLN
EFLIDHKKGSLQSKGWSNDISAYKVSKAAMNAYTRILARKFPSFCINCVCPGYVKTDITCNTGIFTAEEG
AESIVELALLPIDGPSGLFFSRNQVSGF
>TCOSALR2
MEENTFSSAEKRCAIVTGANKGIGFEICRQLVSNGVMVILTARNEDRGAKAVETLRESGLSNVVFHQLDI
KDTTSIAALVSFIESQFKKIDILVNNAAENGVILDFEGFKAYALEYNGMIDANPEKLSQLTVETYEMAEE
CLKTNYYGTKAVTEALLPFLQLSDSPRIVNISSISGQLNGFSNEKVKAELNNVDCLIEERIDELLQRFLK
DFKENMLKANGWPLVPSAYRVSKAALNAYTRILARKLPSFRINCVHPGFVETDMSHHTGALNAEQGAKAP
VMLALLPQDGPSGCYFDQMEMSTF
>XSISALR1
MAEERCAVVTGGNKGIGLEICRQLASNGILVILTARDEKRGTEAVENLKESGFSNVVYHQLDVKDPSSIA
SLANFINTRFRKLDILVNNAGVGGVVIESDAFRALKNGFEDVSDENPDKLKGVMEQTYEKSEECLKTNYY
GTKRVTEALLPALQLSNSARIVNVSSSYGKLMFIHNESVKLELSNFETLTEERIDELILWFLKDFKEDKL
QENGWPITVSAYKLSKAAVNAYTRMLARKFPNMLINCVHPGYVKTDINCNTGLLTAEEGAKGPVKMALLP
DNGPSGHYFNQVNMSTF

SANR

>ZMASDR
MADSATSRPTVLVTGAGGRTGQIVYNKLKERSEQFVARGLVRTEESKQKIGAAGDVYVADIRDADRLAPA
VQGVDALIILTSAAPKMKPGFDPSKGGRPEFYYEDGMYPEQVDWLGQKNQIDAAKAAGVKHIVLVGSMGG
TNPNHPLNSMGNGNILVWKRKAEQYLADSGIPYTIIRPGGLQDKDGGVRELLVGKDDELLQTDTKSIPRA
DVAEVCVQALQYEEAKFKAFDLASKPEGVGTPTKDFRALFSQITARF
>AMESANR1
MAGLVRKVLVTGTAGLTGQIVYKKLRERTEQYIPRGLVRSEERKNKMGGSDDIFIGNIKDVKSLVPAIQG
IDALIILTSALPKIKPKPGFIPSKGERAENYVREDGSFDEMPECYFEDGGSPEEVDWIGQKNQIDVAKAA
GVKQIVLVGSVGGTIPNHPLNSLGNGNQLVWKRKAEQYLADSGVPYTIIRAGGLEEKPGGLRELLVGKDD
ELLQQPELVVVSRADVAEVCIQALQFEEAKFKAFDLGSKPEGIGSPTKDFRDLFSQVTSRF
>AMESANR2
MANSSEKLTVLVTGASGLTGHLAFKKLKERSDKFVVRGLVRSEASKQKLGGGDDIFIGDVMDPKSLEPAM
HGIDALIILTSAIPKIKPGSLPGADGKRAEDVIDSSFDGPMPEFYYEEGQYPEQVDWIGQKNQIDTAKAC
GVKHIVLVGSMGGSDPNHFLNYMGNGNILIWKRKAEQYLADSGIPYTIIRAGGLDNKEGGRELLVGKDDE
LLPTENGYISRADVAEACVQALQIENVKFKAFDLGSKPEGVGEPTKDFKALFASVTTPF
>AMESANR3
MASTVIVTGAGGRTGQIVYKKLKERSDQYLARGLVRTEESKEKIGGADDVFVGDIRDAESILPAIKGVDA
LIILTSAVPKMKPGFDPSKGGKPEFYFEDGAYPEQVDWIGQKNQIDAGKAAEVKHIVLVGSMGGTNLNNP
LNSIGNGNILIWKRKAEQYLADSGIPYTIIRAGGLQDKEGGIRELVVGKDDELVQTEIRTITRADVAEVC
IQALQFEEAKFKALDLASKPEGTGEPTKDFKTLFSQISTRF
>AMESANR4
MASTVIVTGAGGRTGQIVYKKLKERTDQYVARGLVRTEESKEKIGGADDVFVGDIRDAESIAPAIQGVDA
LVILTSAVPKMKPGFDPSKGGRPEFYFEDGAYPEQVDWIGQKNQIDAAKAAGVKQIVLVGSMGGTNLNHP

LNSLGNGNILVWKRKAKQYLADSGVPYTIIRAGGLQDKEGGLRELVVGKDDELLQTDIRTIARADVAEVC
IQALQFEEAKFKAFDLASKPEGCGEPTKDFKTLFSQISTRF
>BTHSANR1
MANSQTVLVTGAAGRTGQIVYKKLKERSDQYVARGLVRTEESKEKIGGKDDVFVGDIRDAGSIVPAIQGI
DALIILTSAVPKMKPGFDPTKGGRPEFYYEEGAMPEQVDWIGQKNQIDAAKAVGVKQIVLVGSMGGTNPD
NPLNKIGDGNILVWKRKAEQYLADSGIPYTIIRSGGLQDKEGGVRELIVAKDDELVATDTRSIPRADVAE
VCIQALQFEEAKFKAFDLASKPEGAGEPTKDFKALFSKITTRF
>CCHSANR1
MATCSQKKTVLVTGASGLTGQIVYKKLKERSDEFVVRGLVRSEASKQKLGGGDDIYIGDINKPETLVPAL
EGVDALIIVTSAIPKIVPGSCAVAGERAEDVIDSSFEGPMPDFYYEEGQYPEQVDWIGQKNQVDAAKAAG
VKHVVLVASMGGLDPNHFLNYMGNGMILIWKRKAEQYLADSGLPYTIIRSGGLDNKAGGRELLVEKDDVL
LPTESGYIARADVAETCVQAVRHEDLTKFKAFDLGSKPEGTGTPTKDFRALFAPITTTF
>CCHSANR2
MACKTCCSKIPCFRQKKTVLVTGAAGLTGNMVYKKLKERSDLFVARGLVRSEGSKKKIGGHDDVFIGDVM
DPKSLAPAMKGIDALVILTSAVPKIKPGSGPNTGKRAEDVIDSSFQGPMPEFYFEEGQYPEQVDWIGQKN
QIDAAKAAGVKHIVLVGSMGGCDPNHFLNSLGNGNILVWKRKSEQYLADSGVPYTIIRAGGLENKAGGRE
LLVGKNDELLPTEAGYISRADVAEACVQAVQFEEVTKFKAFDLGSKPEGTGTPTKDFKALFSLVTARF
>CCHSANR3
MVTCSCTQKKTVLVTGASGLTGSIVYKKLKERSDKFVVKGLVRSEASKQKIGGGDDDVCFGDIMKPETLI
PAMEGVDALIILSSAVPKIVPGTCAIAGERAEDVIDSSFEGPMPDFYYEEGQYPEQVDWIGQKNQVDAAK
AAGVKHVVLVASMGGMDPNHFLNYMGNGMILIWKRKAEQYLADSGLPYTIIRAGGLDNKVGGRELVVGKE
DELLPTESAYIARADVAEACIQALQFEEYVKFKAFDLGSKPEGMGTPTTDFGALFTPITTTF
>CCHSANR4
MAESDRKTTVLLTGAAGATGKFVFKKLKERSEKYVVRGLVRSIESKKKLGENVNDDDVYLGDIQKPETLI
PALQGVDALIILTSAIAKVKPGQNPTRGDRAENYFNEDGSLAEDMPEFHFPPGSIPEEVDWIGQKNQIDA
AITAGVKHIIVVGSICGTIPEHPLNTIGNGDILLWKRKAEKYLTESGVPYTIIRAGALDDKEGGVRELLV
GKDDEVVYQMENIYISRDDVAETCIQALQFEEAKFKAFDLTSKPEGTGTPTKDFKALFSQATARF
>CCHSANR5
MDDSYRKTVLVTGASGATGQIAYNKLKERPEQFVARGFVRSEASKKKIGGGDNVFLGNVMDADSIIPAVE
GIDALIILTSAIPKMKPGVGFSNEGERAEDVIDGSFEGPMPEFYFEEGQFPEQIDWIGQRNQIDAAKAAG
VKHIVLVGSMGGSDPNHPLNTLGNGNILVWKRKAEQYLADSGVPYTIIRAGGLINKPGGLRELLIGKNDE
LLPTESGYVPREDVAEVCVQALQFEEYVKFKAFDLGSKPEGTGTPTKDFKALFSQVTRRF
>CMASANR1
MEGSSQKITVLVTGASGLTGNLAFSKLKERSDKFVVRGLVRSEASKQKLGGGDEIYIGDIKDAESLVPAM
TGIDGLIILTSAIPKIVPGSMPDTEGKRSEDVIDGSFEGPMPEFYYDEGQYPEQVDWIGQKNQIDTAKSL
GVKHVVLVASMGGQDPNHFLNYMGNGNILIWKRKAEQYLADSGLPYTIIRAGGLDNKQGGRELLVDKDDV
LLPTENGYIARADVAEACVQALQCEETKFKAFDLGSKPEGTGTPTTDFKALFAPITTSF
>CMASANR2
MACKSCCSKVLTACSTPKKTVLVTGASGLTGRFAFNKLKERPDKYVARGLVRSEASKQKLGGGDEIYIGD
VMDQKSLEPAMKGVDALIILTSATPKMVPGQFPEGSGERGEDLIDGSFEGPMPEFYFDEGQTPERVDWIG
QKNQIHAAKAAGVKHIILVSSMGSGDPNHPLNSLGNGNILAWKRKSEEYLANSGVPYTIIRAGGLDNKDG
GKRELLVGKNDELLPTEKGYIARADVAEACVQAVLFFDELKNKAFDLGSMPEGTSTPTKDFKALFAPITT
SF
>CMASANR3
MDDSRRKTVLVTGASGATGKLAYDKLKERPDQFVARGFVRSENSRKKIGGGDGVFIGDVQNAESIVPAVQ
GIDALIILTSAIPKIKPGVVPDEGQKAEKYIDSSFESMPEFYYDEGQYPEQVDWIGQKNQIDAARAAGVK
HIILVSSMGSGDPNHPLNSLGNGNILIWKRKAEQYLADSGVPYTIIRAGGLIDNPGGMRELLIGRNDELL
PTEDGYVPRADVAEVCVQATQFEETKFKAFDLGSKPEGKGTPTKDFKALFSKLTDRF
>CMASANR4
MADSDRKKILVTGAAGSTGNLAYKKLKERTEKYVARGLVRSEESKKRIGGADDVFIGNIRDAQTLVPAVE

GIDGLIILTSSLLKIRPGSAAPKGDKAEYYMLEDGSFDPDMPEFYYEESPEQVDWIGQKNQIDAAKAAGV
KHIVLVGSMGGTDPNQPLNSLGDNGNFLIWKRKSEQYLADSGIPYTIIRCGGLDDKEGGKRELLVSKDDE
MFSQIELIYISRDDVAEVCIQAFECEEAKFKAFDLTSKPEGTGTPTRDFKALFAQVTTRF
>CMASANR5
MDNSDRKTVLVTGASGVTGNLAYKKLMQRTDKYVGRGFVRSEASKEKIGGTDNVFIGNINDPQTLVPAVQ
GIDALIILTSSMLKIKPGSAAPKGDKPDNYFNEDGSFDPDMPEFYYEDGNSPEEVDWIGQKNQIDAAKAA
GVKHIVLVGTMCGTFPNHPVNILGNGNMLVWKRKSEQYLADSGIPYTIIRAGGLDDKEGGLREIVFSKDD
EMFADINMIYISRDDVAEVCIQALECDDAKFKAFDLTSNPEGTGTPTTDLKALFSQVTTRF
>CMASANR6
MDDSRRKTVLVTGASGATGKLAYDKLKERPDQFVARGFVRSENSRKKIGGGDGVFIGDVQNAESIVPAVQ
GIDALMILTSAVPKIKPEFLIPTKGERAENYVTEGASFESVPEFYYDEGQFPEQVDWIGQKNQIDVARAA
GVKHIILVGSVGGTDPNHPLNSLGNGNILIWKRKAEQYLADSGVPYTIIRAGGLLDSPGGMRELRIGRND
ELLKTEDGSVPREDVADVCVQATQFEETKFKAFDLGSKPEGKGTPTKDFKALFSQLTDRF
>CMASANR7
MAESQKKMTVLVTGASGLTGQLAFKKLKERSDKFVVRGLVRSEASKQKLGGGDEIFIGDILNEESLVPAM
TGIDGLIILTSAIPRIVPGSMPDTEGKRAEDVVDGSFEGPMPEFYYEEGQYPEQVDWIGQKNQIDTAKSL
GVKHIVLIGSMGGQDPNHFLNYMGNGNILIWKRKAEQYLADSGVPYTIIRAGGLDNKEGGRQLLVGQNDE
LLPTEDGYIARADVAEACIQALQIEESKFKALDLGSKPEGVGELTKDFKPLFASVTTRF
>CMUSANR1
MADLPRTTVLVTGAGGRTGQIVYKKLKERRDQYAARGLVRTEESKEKIGGADDVFVGDIREAERIVPAIQ
GIDALIILTSAVPKMKPGLDPSKGGRPEFYFEDGAFPEQVDWIGQKNQIDAAKDAGVKQIVLVGSMGGTN
PNHPLNSIGNGNILVWKRKAEQYLADSGVPYTIIRAGGLQDKEGGVRELLISKDDELLSTETRTIARADV
AEVCIQALQYEESKFKAFDLASKPEGTGEPTKDFKALFSKITTRF
>ECASANR1
MADSSKKLTVLVSGASGLTGSLAFKKLKERSDKFEVRGLVRSEASKQKLGGGDEIFIGDISDPKTLEPAM
EGIDALIILTSAIPRMKPTEEFTAEMISGGRSEDVIDASFSGPMPEFYYDEGQYPEQVDWIGQKNQIDTA
KKMGVKHIVLVGSMGGCDPDHFLNHMGNGNILIWKRKAEQYLADSGVPYTIIRAGGLDNKAGGVRELLVA
KDDVLLPTENGFIARADVAEACVQALEIEEVKNKAFDLGSKPEGVGEATKDFKALFSQVTTPF
>ECASANR2
MEGSCQKITVLVTGASGLTGQLAYKKLKERSEQFEVRGLVRSEASKQKLGGGNEIFIGDIMDAKTLEPAM
KGIDALIILTSAIPKMKPSDEFPAESCSTGRAEDMIDGSFDGPMPEFYFEDGQYPEQVDWIGQKNQIDTA
KALGVKHIVLVGSMGGSDPNHFLNSLGNGNILIWKRKAEKYLADSGLAYTIIRAGGLDNKEGGIRELLIG
KDDVLLPTENGYIARADVAEACIQALKIEEVKFKAFDLGSKPEGTGVPTKDFGALFSPITERF
>ECASANR3
MEGSCQKMTVLVTGASGLTGQLAYKKLKERSEQFVVRGLVRSEASKQKLGGGDDIFIGDIMNCQTLEQAM
KGIDGLIILTSAIPKMKPTDEFPAESLGSGRAEDMIDGSFDGPMPEFYFEEGQYPEQVDWIGQKNQIDTA
KALGVKHVVLVGSMGGSDPKHFLNSLGNGNILIWKRKAEQYLADSGLAYTIIRAGGLDNKEGGIRELLVG
KDDLLLPTENGYIARADVAEACVQALQFENVKFKAFDLGSKPEGNGEPTKDFGALFAPISERF
>ECASANR4
MACKTSCSKLMATCCSSKKTVLVTGASGLTGQFAFNKLKERSDKFVVRGLVRSEGSKKKLGGGDEIYIGN
VMDPKTLEPAMKGVDALIILTSAIPKMKPGQFPENSNGGGRAEDMIDGSFEGPMPEFYFDEGQFPEQVDW
IGQKNQIDVAKACGVKHIVLISSMGSGDPNHPLNSLGNGNILAWKRKSEEYLSNCGVPYTIIRAGGLDNK
DGGIRQLLVGKNDELLPTEKGYIARADVAEACVQAVQFEELVKFKAFDLGSMPEGTGTPTKDFKALFTPI
TARF
>ECASANR5
MDDSRPKTVLVTGASGATGQIAYNKLKERPEQFIARGFVRSEQSKKKIGGGDDVFLGNIMDSNTIVPAVQ
GIDALIILTSAIPKIKPGVFPNEGEKAEKYIDSSFESMPEFYFDEGQYPEQIDWIGQKNQIDAARAAGVK
HIILVGSVGGSDPNHPLNSLGNGNILVWKRKAEEYLAESGVPYTIIRAGGLIDNPGGMRELLVGKDDEFL
PTENGYVPRADVAEVCVQAVKFEEAKFKTFDLGSKPEGKGKPTTDFKALFSQVTTRF
>ECASANR6

MAASDLGGKKTVLVTGAAGLTGRLVFKKLKERSDKYVVRGLVRSEESKKKIGSGLSDDEEEIFIGDIKDS
QTLIPAMKGIDALIILTSALPKVKPGPAANRGDRAEYYVNEDGSFPDPMPEIYYEQGNYPEQVDWIGQKN
QIDTAIAAGVKQIVLIGSMCGTDPDNFLNKYGNGNILVWKRKSEKYLSESGVPHTIIRAGGLDDLEGGLR
ELQVSKDDEMMAHIENIMISRADVAEVAVQALEHEEAKFKAFDLTSKPVGTGTPNKDFKALFAQTTVPF
>GFLSANR1
MAESQEKMTVLVTGASGLTGQLAFKKLKERSDKFVVRGLVRSEASKQKLGGGDDIFIGDILNAESLVPAM
TGIDGLIILTSAIPKIVPGSFPDTDGKRAEDVVDGSFEGPMPDFYYEEGQYPEQVDWIGQKNQIDTAKSL
GVKHIVLIASMGGQDPNHFLNYMGNGNILIWKRKAEQYLADCGVPYTIIRAGGLDNKEGGRELLVGRNDE
LLPTENGYISRADVAEACVQALQIEESKFKALDLGSKPEGVGEPTKDFKSLFASVTDRF
>GFLSANR2
MDSSSQKMTVLVTGASGLTGSLAYNKLKERSDKFVVRGLVRSEASKQKLGGGDDIFIGDIMNAESLVPAM
TGVDGLIILTSAVPKIVPGSFPNADGKRAEDVLDGAFDGPMPDFYYEEGQLPEQVDWIGQKNQIDTAKAL
GVKHVVLIGSMGGQDPNHFLNYMGNGMILIWKRKAEQYLADSGLPYTIIRAGGLDNKQGGRELLVDKDDV
LLPTENGYIARADVAEACIQALQCEETKFKAFDLGSKPEGTGTPTTDFKALFAPITARF
>GFLSANR3
MACKSCCSKLVAACSSKKTVLVTGASGLTGQFAFNKLKERSDKYVVRGLVRSEGSKKKLGGGDEIYIGNV
MDQKSLEPAMKGVDALIILTSAIPKMKPGQFPEGSGGRAEDMIDGSFEGPMPEFYFDEGQYPEQVDWIGQ
KNQIEAAKAAGVKHIILVSSMGSGDPNHPLNSLGNGNILAWKRKAEEYLAGSGVPYTIIRAGGLDNKQGG
IRELLVGKNDELLPTEKGYIAREDVAEACVQAVLYDEVKFKAFDLGSMPEGTGTPTKDFKALFAPITTSF
>GFLSANR4
MDDSRRKTVLVTGASGATGQIAYNKLKERPDQFIARGFVRSENSKQKIGCGEGVFIGDIQKAESIVPAVQ
GIDALIILTSAIPKIKPGLVPDKGEKAEKYIDGSFESMPEFYFDEGQYPEQVDWIGQKNQIDAARAAGVK
HIILVGSAGGTDPNHPLNSLGNGNILIWKRKAEQYLAESGVPYTIIRAGGLIDNPGGMRELVVGRNDELL
PTENGYVPRADVAEVCVQATQFEEAKFKAFDLGSKPEGKGTPTKDFKALFSQVSDRF
>GFLSANR5
MVESGRKTVLVTGAAGLTGKLAYKKLKERTEKYVGRGFVRSEESKKRIGGADDVFIGNIKDAQTLIPAVQ
GIHGLIILTSCSAKVKPGCAAPKGDRPENYILEDGSLDPDIPEFYFEDGASPEEVDWIGQKNQIDAAKAA
GVKHIVLVGGMGGMDPYSPLSNFGNGNVLVWKRKAEQYLADSGIPYTIIRAGGLEDKEGGRRELLVSKDD
EMFSQMEMAYISRDDVAEVCIQALECEEAKFKAFDLTSKPEGTGKPTRDFKALFSQVTTRF
>GFLSANR6
MVGLLRRTVLVTGAAGLTGQIVYNKLRERVTQYIPRALVRSEERKRKIGGSDDVFIGDITDAKSIIPAIQ
GIDALIVLTSAVPKIKPGYVPTKGERAENYIEDDGSFDEMPEFYFEDGGEPEQVDWIGQRNQIDAAKAAG
VKQIVLVGSMCGTIPNHPLNSLGNGNILVWKRKAEQYLADSGVPYTIIRAGGLEDKAGGLRELIVGQDDD
LLQQPVLADITRADVAEVCIQALQFEEAKFKAFDLGSKPEGTGFPTNDFKALFCQVTAHF
>JDISANR1
MATLPTVLVTGAGGRTGQIVYKKLKEKVDQYVARGLVRTEESKEKIGGADDVFVGNITDAESIVPAFQGI
DALIILTSAVPKMKPGFDPSKGGRPEFYFEDGAYPEQVDWIGQKNQIDAAKAVGVKQIVLVGSMGGTNLD
NPLNSLGNGKILVWKRKAEQYLADSGIPYTIIRSGGLQDKEGGVRELLVGKDDELVKTDTRTIARADVAE
VCVQALQFEEAKFKAFDLASKPEGTGEPTKDFKGLFSKITTTF
>MAQSANR1
MADLQTVLVTGAAGRTGQIVYKKLKERSDQYVARGLVRTEESKEKIGGKDDVFVGDIRDAGSIVPAVQGI
DALIILTSAVPKMKPGFDPTKGGRPEFYFEEGAMPEQVDWIGQKNQIDAAKAVGVKQIVLVGSMGGTNPD
NPLNKIGDGNILVWKRKAEQYLADSGIPYTIIRAGGLQDKEGGVRELIVAKDDELVTTDTRSIPRADVAE
VCIQALQFEEAKFKAFDLASKPEGAGEPTKDFKALFSKITTRF
>MCASANR1
MANLTRITVLVTGAGGRTGQIVYRKLKERADQYLARGLVRTEESKDKIGGADDVFVGDIRDAESIYPVIQ
GIDALIILSSAVPKMKPGYDPSTGGRPEFIFEDGANPEQVDWIGQKNQIDAAKDAGVKQIVLVGSVGGTN
PNYSLNSMGNGNILVWKRKAEQYLADSGIPYTIIRAGGLQDKEGGVRELLVGKDDELLNTETIKIARADV
AEVCIQALQFEEAKYKAFDLASKPEGTGEPTRNFKALFSQITSRF
>NDOSANR1

MAGLPTVLVTGAGGRTGQIVYKKLKERADQYAARGLVRTEESKEKIGAADDVFVGDIRDASSIVPAIQGI
DALIILTSAVPKMKPGFDPSKGGRPEFYYEDGAFPEQVDWIGQKNQIDAAKAVGVKQIVLVGSMGGTNLD
HPLNNIGNGKILVWKRKAEQYLADSGIPYTIIRAAGLQDKEGGVRELVVGKDDELLQTETRAIARADVAE
VCIQALQFEEAKFKAFDLASKPEGTGQPTKDFKALFSKITTRF
>NSASANR1
MAADSSRSTVLVTGAGGRTGQIVYKKLKEKTDQYVARGLVRTEESKEKIGGADDVFVGDIRDAESLAPAI
EGIDALVILTSAVPKMKPGFDPSKGGRPEFYFEDGAFPEQVDWIGQKHQIDAAKAAGVKQIVLVGSMGGT
NLNHPLNSIGNGNILVWKRKAEQYLADSGIPYTIIRAGGLQDKEGGLRELIVSKDDELLQTETRTIARAD
VAEVCVQALQFEEAKFKAFDLASKPEGTGEPTKDFKSLFSQIVSRF
>PBRSANR1
MASTVIVTGAGGRTGQIVYKKLKERTDEFVARGLVRTEESKEKIGGADDVFVADIRDAESIVPAIQGADA
LVILTSAVPKMKPGFDPTKGGRPEFFFEDGANPEQVDWIGQKNQIDAAKAAGVKQIVLVGSMGGTNLNHP
LNSIGNGNILVWKRKAEQYLADSGIPYTIIRAGGLQDKDGGVRELVVGKDDELLETDIRTIARADVAEVC
IQALLLEEAKFKALDLASKPEGTGEPTKDFKTLFSQISTRF
>PBRSANR2
MAESNQKITVLVTGASGLTGEIAFKKLKERSDKFVVRGLVRSEASKQRLGGGDEIFLGDVMDKKSLETAM
QGIDALIILTSAVPKIVPGSYPGADGKRAEDVFGESFDFNGPMPEFYYEEGQYPEQIDWIGQKNQIDTAK
SCGVKHIVLVGSMGGTDPNNFLNHMANGNILVWKRKAEQYLADSGIPYTIIRAGGLDNKVGGRELLVGKD
DELLSTENHFIARADVAEACVQALQIEESKFKAFDLGSMPEGVGEPTKDFKALFAPITTRF
>SCASANR1
MADSRQKLTVLVTGASGLTGQIAFKKLKERSDQFVVRGLVRSEASKQKLGGGDEIYIGDVMNAESLVPAM
TGIDALIILTSAIPKIKPSESFPVVEGQRSEDVVDGSFSGPMPEFYYEEGQYPEQVDWIGQKNQIDTAKA
LGVKHIVLVGSVGGTDPNHFLNSMGNGNILIWKRKAEQYLADSGIPYTIIRAGGLDNKEGGRELIVGKDD
ELLSTENGYIARADVAEACVQALQIEEAKCKALDLMSKPEGVGEPTKDFKGLFASVTTRF
>SCASANR2
MADSRQKFTVLVTGASGLTGHFAFKKLKERSDQFVVRGLVRSEASKQKLGGGDQIYIGDVMNAESLVPAM
TGIDALIILTSAIPKIKADESFPVAEGQRAEDVIDETFSGPMPEFYYEEGQYPEQVDWIGQKNQIDTAKA
LGVKHVVLVASMGGQDPNHFLNYMGNGNILIWKRKAEQYLADSGLPYTIIRAGGLDNKQGGIRELLVDKD
DVLLPTANGYIARADVAEACVQALHCEEVKFKAFDLGSKPEGTGTPTKDFKALFAPITTSF
>SCASANR3
MACKSCCSKIMACSTPKKTVLVTGASGLTGQFAFNKLKERSDKFVARGLVRSEGSKKKLGGGDEIYIGDV
MDQKSLEPAMKGIDALIILTSAIPKMKPGQFPEGGGGGGRAEDLIDGSFEGPMPEFYFEEGQYPERVDWI
GQKNQIDAAKAAGVKHIVLISSMGSGDPNHPLNSLGNGNILAWKRKSEEYLANSGVPYTIIRAGGLDNKE
GGIRELLVGKNDELLPTEKGYIARADVAEACVQAVLFEEVKFKAFDLGSMPEGTGTPTKDFKALFAPITT
HF
>SCASANR4
MDDSRRKTVLVTGASGATGQIAYNKLKERPDQFVARGFVRSEHSKQKIGGGDGVFIGDILDPESIAPAVQ
GIDALIILTSAIPKIKPGQFPDTGGKAESYISQSFESMPEFYFEEGQYPEQVDWIGQKNQIDAAKAAGVK
HIVLLGSMGGTDPNHPLNVLGNGNILIWKRKSEKYLADSGLPYTIMRAGGLIDNPGGMRELLVGRNDELL
PTENGFVPRADVAEVCVQALLFEEAKFKAFDLASKPEGKGTPTKDFKALFSQVTDRF
>SCASANR5
MARLLQSTVLVTGAAGLTGQIVYKKLRERATQYIPRGLVRSEERKKKIGGSDDVFIGDITDAKSIIPAVQ
GIDALIILTSAIPKIKPGFIVPTKGERAENYIGEDGSFDEMPEFYFKDGGSPEQVDWIGQKNQIDAAKGA
GVKQIVLVGSMCGTIPNHPLNNLGNGNILVWKRKAEQYLADSGVPYTIIRAGGLGDKPGGLRELLVGKDD
ELLQQPELVDVSRADVAEVCIQALQFEEAKFKAFDLGSKPEGTGTPTNDFKALFSQVSTTF
>SDISANR1
MADSDRKTILVTGAAGSTGNLAYKKLKERTEKYVARGLVRSEESKKRIGGADDVFIGNIRDAQTLVPAVE
GIDGLIILTSSLLKIRPGSAAPKGDKAEYYFLEDGSLDPDMPEFYYEESPEQVDWIGQKNQIDAAKAAGV
KHIVLVGSMGGTDPNHPLNILGDNGNFLIWKRKAEQYLADSGIPYTIIRCGGLDDKEGGKRELLVSKDDE
MFSQIELIYISREDVAEVCIQAFECEEAKFKAFDLTSKPEGTGTPTRDFKALFAQVTTRF

```
>SDISANR2
MAESQQKMTVLVTGASGLTGQLAFKKLKERSDKFVVRGLVRSEASKQKLGGGDEIFIGDILNTESLVPAM
TGIDGLIILTSAIPRIVPGSMPDTEGKRAEDVVDGSFEGPMPEFYYEEGQYPEQVDWIGQKNQIDTAKSL
GVKHIVLIGSMGGQDPNHFLNYMGNGNILIWKRKAEQYLADCGVPYTIIRAGGLDNKEGGRQLLVGQNDE
LLPTEDGYISRTDVAEACIQALQIEESKFKALDLGSKPEGVGEPTKDFKPLFASVTTRF
>SDISANR3
MDDSRRKTVLVTGASGATGKLAYDKLKERPDQFIAKGFVRSENSRKKIGGGDGVFIGDVQNAESIVPAVQ
GIDALIILTSAIPKIKPGLVPDEGQKAEKYIDSSFESMPEFYYDEGQYPEQVDWIGQKNQIDAARAAGVK
HIILVSSMGSGDPNHPLNSLGNGNILIWKRKAEQYLADSGVPYTIIRAGGLIDNPGGMRELLIGRNDELL
PTEDGYVPRADVAEVCVQATQFEETKFKAFDLGSKPEGKGTPTKDFKALFSKLTDRF
>SDISANR4
MANSDRKTVLVTGASGVTGNLAYKKLMERTDKYVGRGFVRSEASKEKIGGTDNVFIGNISDPQTLVPAVQ
GIDALIILTSSMLKIKPGSAAPKGVKPDDYFNEDGSFDPDMPEFYYEDGNSPEEVDWIGQKNQIDAAKAA
GVKHIVLVGSMCGTYPNHPVNILGNGNMLVWKRKSEQYLADSGIPYTIIRAGGLDDKEGGLREIVFSKDD
EMFADMNMIYISRDDVAEVCIQALECEDAKFKAFDLTSNPEGTGTPTTNLKALFSQVTTRF
>SDISANR5
MAESNRSTVLVTGAGGRTGQLVYKKLKERSSEYIARGLVRSEESKSKIGGANDVFIGDVTKPETLAPAIE
GIDALVILTSGVPKMKPGFDPTKGGRPEFYFEDGQYPEQVDWEGQKSQIDAAKAAGVKQIVLVGSMGGTN
LNHPLNSLGNGNILVWKRKAEQYLADSGIPYTIIRAGGLQDKEGGIRELLIGKDDELLQTETKTVARPDV
AEVCVQALQIEEAKFKAFDLASKPEGTGTPTKDFKALFSQITTRF
>SDISANR6
MTTIIVTGAGGRTGQIVYKKLKERTDQYVARGLVRTEESKEKIGGADDVFVGDIRDADSIVPAIQGADAL
VILTSAVPKMKPGFDPTKGGRPEFYFEEGAYPEQVDWIGQKNQIDAAKAAGVKQIVLVGSMGGTNPNHPL
NSIGNGNILVWKRKAEQYLADSGIPYTIIRAGGLQDKEGGVRELVVGKDDELLETEIRTIARADVAEVCI
QALQIEEAKFKALDLASKPEGTGEPTKDFKSLFPQVSTRF
>TCOSANR1
MADLPRTTVLVTGAGGRTGQIVYKKLKERADQYVGRGLVRTEESKEKIGGADDVFVGDIRDAESILPAIQ
GIDALIILTSAVPKMKPGFDPSKGGRPEFYFEEGSYPEQVDWIGQKNQIEAAKNAGVKQIVLVGSMGGTN
PNHPLNSLGNGNILVWKRKAEQYLADSGIPYTIIRAGGLQDKEGGVRELLVGKDDELLQLDTRSIAREDV
AEVCIQALQFEEAKFKAFDLASKPEGAGEPTKDFKALFSKISTRF
>TFLSANR1
MSATDRSTVLVTGAAGRTGQIVYKKLKERVDQYVARGLVRTEESKEKIGGADDVFLGDIRDAGSITPAIQ
GIDALVILTSGVPKMKPGFDPSKGGRPEFYFEEGAYPEQVDWIGQKNQIDAAKEAGVKQIVLVGSMGGTN
LNHPLNSLGNGNILVWKRKAEQYLADSGIPYTIIRAGGLQDKEGGLRELIVGKDDELLQTETRSITRADV
AEVCIQALQFEEAKFKAFDLASKPEGTGEPTRDFKALFSQVATRF
>XSISANR1
MAPSTVLVTGAGGRTGQLVYKKLKERVDQYVARGLVRTEESKEKIGGADDVFVGDIRDTASIAPAIQGID
ALVILTSAVPKMKPGFDPSKGGRPEFFFEDGAYPEQVDWIGQKNQIDSAKAAGVKQIVLVGSMGGTNPNH
PLNSLGNANILVWKRKAEQYLADSGIPYTIIRAGGLQDKEGGLRELIVGKDDELLQTETRGIPRAEVAEV
CIQALQFEEAKFKALDLASKPEGTGEPTKDFKALFSQVTTRF
```
